# Koningipyridines A and B, two nitrogen-containing polyketides from the fungus *Trichoderma koningiopsis* SC-5

**DOI:** 10.1007/s13659-024-00429-z

**Published:** 2024-01-11

**Authors:** Weiwei Peng, Qi Huang, Xin Ke, Wenxuan Wang, Yan Chen, Zihuan Sang, Chen Chen, Siyu Qin, Yuting Zheng, Haibo Tan, Zhenxing Zou

**Affiliations:** 1 Xiangya School of Pharmaceutical Sciences, Hunan Key Laboratory of Diagnostic and Therapeutic Drug Research for Chronic Diseases, Central South University, Changsha, 410013 People’s Republic of China; 2 National Engineering Research Center of Navel Orange, Gannan Normal University, Ganzhou, 341000 People’s Republic of China; 3 Key Laboratory of National Forestry and Grassland Administration on Plant Conservation and Utilization in Southern China, State Key Laboratory of Plant Diversity and Specialty Crops, Key Laboratory of South China Agricultural Plant Molecular Analysis and Genetic Improvement, Guangdong Provincial Key Laboratory of Applied Botany, South China Botanical Garden, Chinese Academy of Sciences, Guangzhou, 510650 People’s Republic of China; 4 Department of Pharmacy, Xiangya Hospital, Central South University, Changsha, 410013 People’s Republic of China

**Keywords:** *Trichoderma koningiopsis*, Endophytic fungi, Secondary metabolites, Koningipyridine, Cytotoxic activity

## Abstract

Two novel koninginin derivatives, koningipyridines A and B (**1** and **2**), along with four known compounds (**3˗6**) were isolated from the EtOAc extract of the endophytic fungus *Trichoderma koningiopsis* SC-5. Among them, koningipyridine A featured an unprecedented pentacyclic ketal skeleton with the formation of a fascinating 6/6/5/6/5 fused ring system and shared a characteristic pyridine core, which represents the first example of nitrogen-containing koninginin-type natural product. Moreover, koningipyridine B was the first member in the koninginin family sharing a unique 6/6/5 dihydropyridine skeleton, and it was suggested to be the critical biosynthetic precursor of koningipyridine A. The structures of **1** and **2** were elucidated by the interpretation of 1D and 2D NMR spectroscopy, HRESIMS data, as well as theoretical calculations of ^13^C NMR and electronic circular dichroism (ECD). Moreover, all isolates were screened for antimicrobial activities against *Staphylococcus aureus*, MRSA, and *Escherichia coli* as well as the cytotoxic effects against three cancer cell lines (A549, Hela, and HepG2).

**Graphical Abstract:**

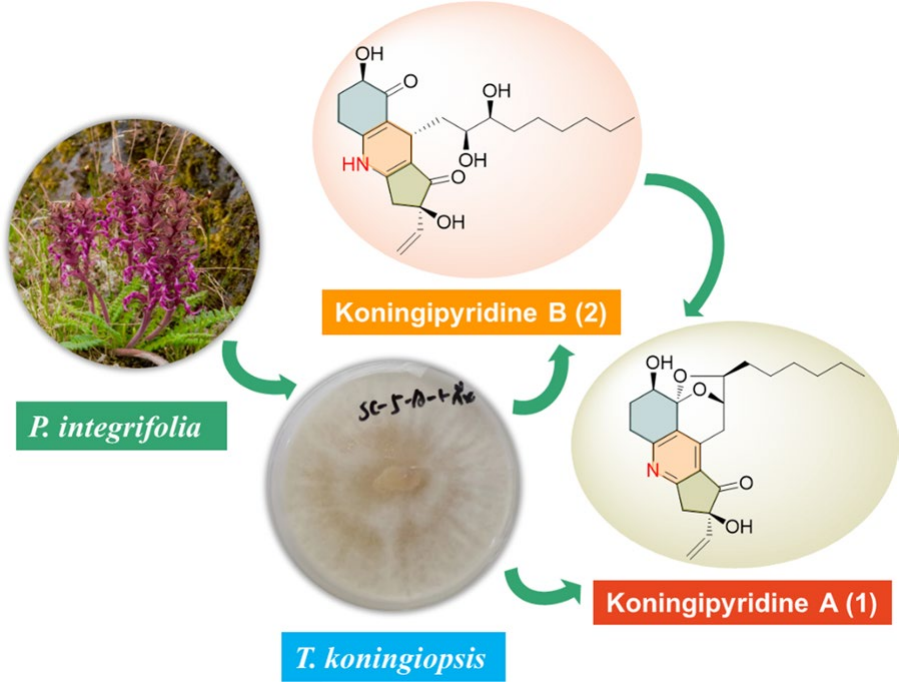

**Supplementary Information:**

The online version contains supplementary material available at 10.1007/s13659-024-00429-z.

## Introduction

Endophytic fungi have been well respected as an essential bioresource of structurally intriguing and biologically diverse active natural products [1, 2]. Genus *Trichoderma* is one of the most well-investigated fungi, which are broadly known due to their outstanding production capability to generate pharmaceutically bioactive lead molecules, including a series of polyketides [3–5], peptaibols [6], and terpenoids [7, 8]. A number of secondary metabolites derived from *Trichoderma* had been evidenced to show significant antibacterial [9, 10], antifungal [3, 11], and cytotoxic activities [6, 12–14]. Many of them had been widely applied in the biological control industry as agricultural agents [15–18].

Koninginins are mainly produced by the species of the *Trichoderma* genus and are considered as their main characteristic chemical constituents [11, 19, 20], and they have successfully attracted broad research attention from researchers in the fields of medicinal chemistry and pharmacology due to their novel structural skeletons and significant biological activities [19, 20]. Nowadays, more than 50 koninginin derivatives have been subsequently discovered [4, 8, 21–31]. Among the reported koninginin derivatives, most of them shared an intriguing bicyclic pyran skeleton with a typical hemiketal or ketal moiety. Moreover, the koninginins with structurally unprecedented and complex tricyclic pyran [21, 26] and tetracyclic pyran skeletons were also occasionally found in related fungi in recent years [4, 8]. Notably, the koninginins have been also disclosed to be promising lead pharmaceutical agents for the therapeutic treatment of fungal infection, thus serving as a type of significant lead natural products for drug development [15–18].

**Fig. 1:**
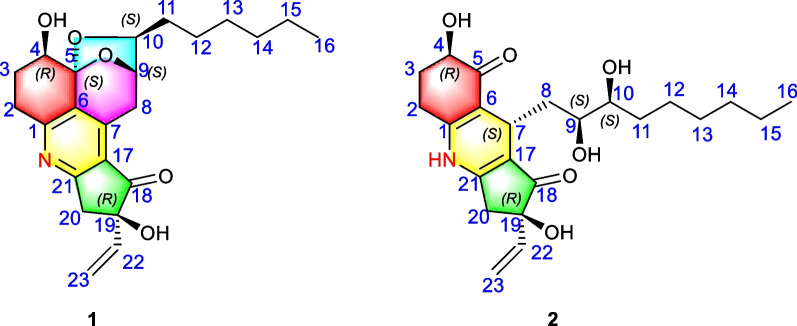
Structures of compounds **1** and **2**

In our ongoing research program focusing on the discovery of biologically meaningful natural products with unique structures from plant-originated fungi [32–40], the strain of *Trichoderma koningiopsis* SC-5 derived from *Pedicularis integrifolia* was experimentally revealed to produce abundant structurally diverse secondary metabolites as referring to the TLC and HPLC–DAD analyses. The further detailed chemical investigation of *Trichoderma koningiopsis* SC-5 had led to the isolation of two fascinating koninginin derivatives – koningipyridines A and B (**1** and **2**), which featured an unprecedented pentacyclic ketal skeleton with the formation of a complex 6/6/5/6/5 fused ring system and a unique 6/6/5 dihydropyridine skeleton, respectively (Fig. 1). Herein, the isolation, structural elucidation, and biological evaluation of these two isolates were described.

## Results and discussion

Koningipyridine A (**1**) was isolated as a yellow oil. It had the molecular formula of C_23_H_29_NO_5_ responsive for 10 degrees of unsaturation referring to the positive HRESIMS ion peak at *m/z* 400.2117 [M + H]^+^ (calcd for C_23_H_30_NO_5_^+^, 400.2118). The ^1^H NMR spectrum coupling with heteronuclear singular quantum correlations (HSQC) spectra of **1** showed a set of characteristic vinyl signals at *δ*_H_ 5.94 (1H, dd, *J* = 17.4, 10.8 Hz, H-22), 5.41 (1H, dd, *J* = 17.4, 0.6 Hz, H-23a), 5.23 (1H, dd, *J* = 10.8, 0.6 Hz, H-23b), three oxygenated methines at *δ*_H_ 4.57 (1H, dd, *J* = 4.8, 1.8 Hz, H-9), 4.08 (1H, dd, *J* = 4.2, 1.2 Hz, H-4), and 3.96 (1H, td, *J* = 7.2, 1.8 Hz, H-10), as well as a marginal methyl group at *δ*_H_ 0.89 (3H, t, *J* = 6.6 Hz, H-16). Moreover, the ^13^C NMR (Table 1) coupling with the DEPT 135 spectra disclosed 23 carbons consisting of one carbonyl group at *δ*_C_ 204.9 (C-18), nine methylenes at *δ*_C_ 28.2 (C-2), 26.8 (C-3), 33.5 (C-8), 36.1 (C-11), 26.5 (C-12), 30.5 (C-13), 33.1 (C-14), 23.8 (C-15), and 45.1 (C-20), three methines at *δ*_C_ 69.3 (C-4), 78.2 (C-9), and 83.1 (C-10), seven aromatic or olefinic carbons including a vinyl group at *δ*_C_ 116.0 (C-23) and 139.3 (C-22), together with five quaternary carbon atoms at *δ*_C_ 163.4 (C-1), 131.0 (C-6), 145.8 (C-7), 125.7 (C-17), and 170.8 (C-21), two oxygen-bearing quaternary carbons at *δ*_C_ 104.5 (C-5) and 81.1 (C-19), as well as one methyl group at *δ*_C_ 14.6 (C-16).

**Table 1:** ^1^H (600 MHz) and ^13^C (150 MHz) NMR data of** 1** and **2**

No	**1**^a^		**2**^b^	
	*δ*_H_ (*J* in Hz)	*δ*_C_	*δ*_H_ (*J* in Hz)	*δ*_C_
1		163.4		153.9
2	3.17^c^ (1H, m); 2.91 (1H, dd, *J* = 19.2, 6.6)	28.2	2.58 (1H, m); 2.54 (1H, m)	25.7
3	2.25 (1H, m); 2.18 (1H, m)	26.8	2.10 (1H, m); 1.75 (1H, m)	29.3
4	4.08 (1H, dd, *J* = 4.2, 1.2)	69.3	3.93 (1H, dd, *J* = 12.6, 4.8)	71.4
5		104.5		198.5
6		131.0		111.5
7		145.8	3.52 (1H, t, *J* = 6.0)	25.3
8	3.51 (1H, dd, *J* = 19.8, 5.4); 3.17^c^ (1H, m)	33.5	1.43 (1H, m); 1.35^c^ (1H, m)	41.1
9	4.57 (1H, dd, *J* = 4.8, 1.8)	78.2	3.29 (1H, m)	70.2
10	3.96 (1H, td, *J* = 7.2, 1.8)	83.1	3.25 (1H, m)	73.3
11	1.58 (2H, m)	36.1	1.35^c^ (1H, m); 1.24^c^ (1H, m)	32.7
12	1.40 (1H, m); 1.31^c^ (1H, m)	26.5	1.24^c^ (2H, m)	26.2
13	1.31^c^ (2H, m)	30.5	1.24^c^ (2H, m)	29.4
14	1.31^c^ (2H, m)	33.1	1.24^c^ (2H, m)	31.9
15	1.31^c^ (2H, m)	23.8	1.24^c^ (2H, m)	22.6
16	0.89 (1H, t, *J* = 6.6)	14.6	0.86 (1H, t, *J* = 7.2)	14.4
17		125.7		115.1
18		204.9		201.0
19		81.1		78.4
20	3.40 (1H, d, *J* = 18.0); 3.17^c^ (1H, m)	45.1	2.72 (1H, dd, *J* = 18.0 1.8); 2.62 (1H, m)	40.3
21		170.8		162.5
22	5.94 (1H, dd, *J* = 17.4, 10.8)	139.3	5.76 (1H, dd, *J* = 17.4, 10.8)	140.4
23	5.41 (1H, dd, *J* = 17.4, 0.6); 5.23 (1H, dd, *J* = 10.8, 0.6)	116.0	5.28 (1H, dd, *J* = 17.4, 1.2); 5.08 (1H, dd, *J* = 10.8, 1.2)	114.0

^a^Record in CD_3_OD, *δ* in ppm; ^b^Record in DMSO-*d*_6_, *δ* in ppm; ^c^Overlapped

**Fig. 2:**
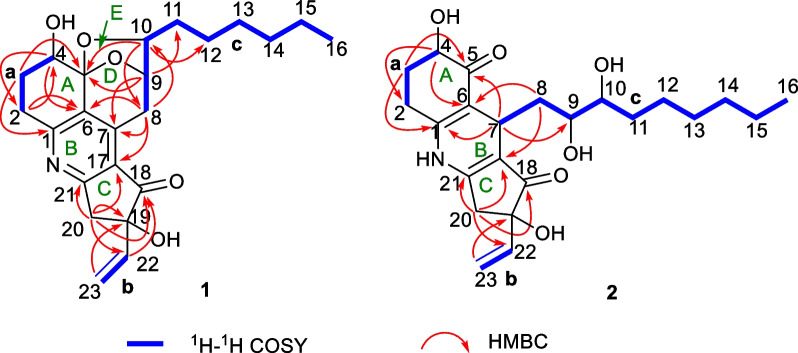
Key ^1^H-^1^H COSY and HMBC correlations of compounds **1** and **2**

The aforementioned NMR features pointed to the conclusion that compound **1** should possess a complex pentacyclic fused ring system, which could be further constructed through the chemo-logical interpretation of ^1^H–^1^H COSY and HMBC spectra. The ^1^H–^1^H COSY cross peaks of H_2_-2/H_2_-3/H-4, H-22/H-23a/H-23b*,* and H_2_-8/H-9/H-10/H_2_-11/H_2_-12/H_2_-13/H_2_-14/H_2_-15/H_3_-16 of **1** suggested the existence of three independent fragments: **a** (C-2/C-3/C-4), **b** (C-22/C-23), together with **c** (C-8/C-9/C-10/C-11/C-12/C-13/C-14/C-15/C-16) as depicted in Fig. 2. On basis of the fragment **a**, the critical HMBC correlation signals from H_2_-2 to C-4/C-6, H_2_-3 to C-1/C-5, and H-4 to C-2/C-6 indicated the presence of cyclohexene ring A. Secondly, the obvious HMBC correlation signals from H_2_-8 to C-6/C-7/C-9/C-10, H-9 to C-5/C-7, as well as H-10 to C-5/C-8/C-9 and ^1^H–^1^H COSY cross-peaks of H-8/H-9/H-10 further constructed the rings D and E. Besides, as referring to the critical ^1^H–^1^H COSY fragment **c**, the side aliphatic chain was verified to be located at C-10 position of ring E with the aid of the key HMBC correlation signals from H-10 to C-12 and H-9 to C-11 (Fig. 2). The linkage for the three core rings A, D, and E was constructed as a 6/6/5 fused tricyclic pyran-ketal bridge skeleton, which was closely similar to the sub-structure of koninginin A [41] and koningiopisin C [21], two secondary metabolites also derived from the fungi of genus *Trichoderma*.

In addition, the existence of an unprecedented cyclopentanone ring C in **1** could be confirmed through the obvious HMBC correlation signals from H-20 to C-17, C-18, C-19, and C-21. The terminal double bond was linked at the C-19 position of the C ring by the obvious HMBC correlation signals from H-22 to C-18 and C-20 as well as H-23 to C-19. Finally, by the comprehensive consideration of chemical formula, molecular unsaturation, together with NMR shifts for C-1 and C-21 of compound **1**, the connection of rings A and C was deduced to be linked through C-1-*N*–C-21 bond with the construction of a penta-substituted pyridine ring B. Then, the planar architecture of **1** had been completely constructed to feature an unprecedented 6/6/5/6/5 fused pentacyclic skeleton with a natural rarely-occurring penta-substituted pyridine ring system, and it was exampled as the first member with a nitrogen-containing skeleton reported in the koninginin family.

**Fig. 3:**
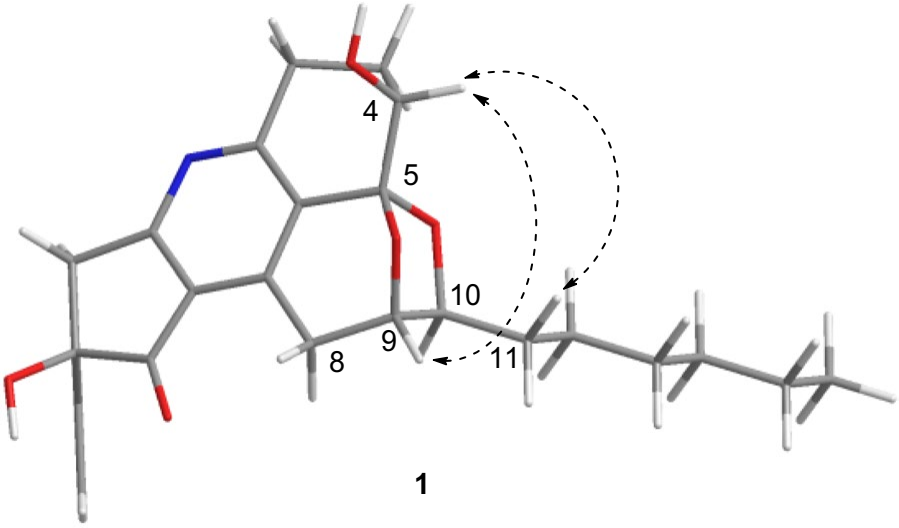
Key ^1^H-^1^H NOESY correlations of compound **1**

The relative configuration of **1** was tentatively determined with aid of the NOESY spectrum. In the NOESY spectrum, the cross peak between H-4 and H-9 revealed that the protons of H-4 and H-9 ought to be assigned on the same side as *α*-orientation, whereas the C-5/*O*/C-9 should be on the other side (Fig. 3). Moreover, the cross peaks between H-4 and H-11, H-12 were also disclosed, and it strongly suggested that the side chain (C-10 to C-16) should be *β*-orientation, when H-4 is *α*-orientation. However, the determination of the relative configuration of C-19 terminal vinyl moiety was seemed to be an intractable challenge, because it was far away from the chiral centers in rings A, D, and E, which thus resulted in the lack of the NOESY correlation.

**Fig. 4:**
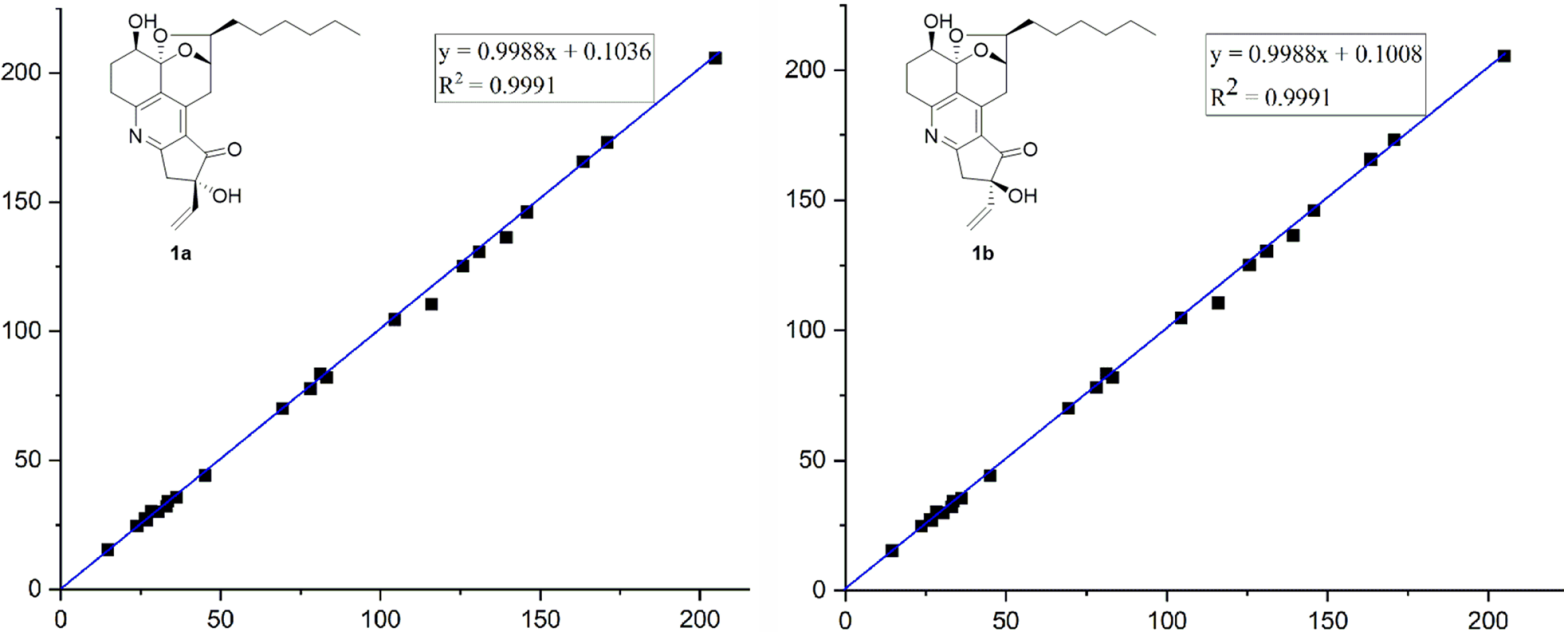
Regression analyses of experimental and calculated ^13^C NMR chemical shifts for **1a** and **1b**

In order to solve this bleak problem of the relative configuration at C-19, the ^13^C NMR calculation was applied. The GIAO-DFT ^13^C NMR calculations [42, 43] for the two probable candidate diastereoisomeric structures **1a** and **1b** were performed at the *ω*B97x-D/6-31G* (IEFPCM, CD_3_OD) level [44, 45]. As a result, both of the correlation coefficient (*R*^2^) values for **1a** and **1b** were 0.9991 (Fig. 4), these results indicated that the previous inference of the planar structure and partial relative configuration (C-4/C-5/C-9/C-10) of compound **1** could be significantly trusted. Moreover, the resulting *P*_rel_ value of **1b** was 90.48%, while that of **1a** was only 9.52% (Table 2), which collectively pointed to that the diastereoisomeric **1b** or its enantiomer is more likely to be the correct relative structure for **1**.

**Table 2:** Calculated ^13^C chemical shifts (CD_3_OD) of structures **1a** and **1b** fitting to the experimental data of **1**

No	Exptl. ***δ***	**1**			
		**1a**	abs dev^c^	**1b**	abs dev^c^
1	163.4	165.50	2.10	165.62	2.22
2	28.2	30.31	2.11	30.28	2.08
3	26.8	26.81	0.01	26.81	0.01
4	69.3	70.10	0.80	70.09	0.79
5	104.5	104.43	0.07	104.54	0.04
6	131.0	130.57	0.43	130.43	0.57
7	145.8	146.12	0.32	146.06	0.26
8	33.5	34.15	0.65	34.33	0.83
9	78.2	77.71	0.49	77.99	0.21
10	83.1	82.24	0.86	81.95	1.15
11	36.1	35.58	0.52	35.52	0.58
12	26.5	27.36	0.86	27.32	0.82
13	30.5	30.00	0.50	29.95	0.55
14	33.1	32.16	0.94	32.15	0.95
15	23.8	24.62	0.82	24.62	0.82
16	14.6	15.27	0.67	15.26	0.66
17	125.7	124.99	0.71	125.01	0.69
18	204.9	205.68	0.78	205.54	0.64
19	81.1	83.59	2.49	83.51	2.41
20	45.1	44.19	0.91	44.15	0.95
21	170.8	173.09	2.29	173.19	2.39
22	139.3	136.35	2.95	136.40	2.90
23	116.0	110.49	5.51	110.56	5.44
		MAE^a^	1.56	MAE^a^	1.54
		RMS^b^	2.20	RMS^b^	2.14
		*P*_mean_	7.14%	*P*_mean_	7.87%
		*P*_rel_	9.52%	*P*_rel_	90.48%

^a^Mean absolute error; ^b^Root mean square; ^c^Absolute deviation of calcd *δ*_C_

**Fig. 5:**
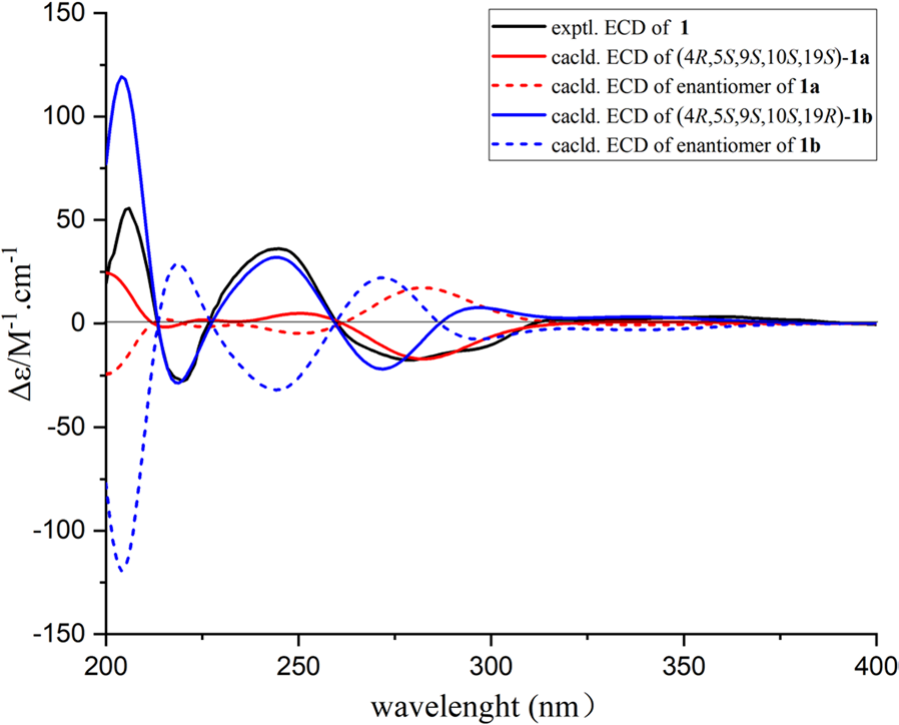
Experimental and calculated ECD spectra of compound **1** (in MeOH)

With the hope to verify the relative configuration of compound **1** and determine its absolute configuration, the TDDFT-ECD theoretical calculations in Gaussian16 involving the diastereoisomers **1a** and **1b** together with their corresponding enantiomers were applied. Experimentally, geometric optimizations of the probable isomers of **1a** and **1b** were conducted to discover the desired conformers with minimum energy, and the TDDFT methodology was then implied at the *ω*B97x-D/TZVP theory level. Furthermore, the related conformers were Boltzmann averaged to get calculated ECD spectra of **1a** and **1b**. As the results showed in Fig. 5, the experimental ECD curve of **1** had been disclosed to be much more consistent with the calculated one of **1b** by comparing the experimental and calculated ECD curves. Notably, this result was also perfectly matched with the conclusion of the ^13^C NMR DFT calculations. Therefore, as referring to the aforementioned reliable results, the absolute configuration of **1** was finally deduced to be 4*R*,5*S*,9*S*,10*S*,19*R* and named koningipyridine A.

Koningipyridine B (**2**) was purified as a yellow oil. Its chemical formula C_23_H_33_NO_6_ was determined on the basis of the positive HRESIMS ion peaks at *m/z* 420.2394 [M + H]^+^ (calcd for C_23_H_34_NO_6_^+^, 420.2386) and 442.2224 [M + Na]^+^ (calcd for C_23_H_33_NO_6_Na^+^, 442.2206), which indicated the existence of eight degrees of unsaturation in **2**. A further careful inspection and comprehensive elucidation of the NMR spectra for **2** revealed its chemical structure with a certain similarity to that of **1**. Moreover, the 1D NMR spectral data also showed a set of vinyl group signals at *δ*_H_ 5.76 (1H, dd, *J* = 17.4, 10.8 Hz, H-22), 5.28 (1H, dd, *J* = 17.4, 1.2 Hz, H-23a), 5.08 (1H, dd, *J* = 10.8, 1.2 Hz, H-23b) as well as *δ*_C_ 114.0 (C-23) and 140.4 (C-22) together with four non-proton olefinic carbons at *δ*_C_ 153.9 (C-1), 111.5 (C-6), 115.1 (C-17), and 162.5 (C-21).

Besides, two keto-carbonyl functional groups at *δ*_C_ 201.0 (C-18) and 198.5 (C-5) were observed. The aforementioned established functional groups accounted for five degrees of unsaturation, which suggested that compound **2** ought to be the ketal ring-opening product of **1** with a tricyclic system by the consideration of the existence of the two hydroxyl groups in the side aliphatic chain and a keto-carbonyl moiety in the cyclohexene ring A.

The planar structure of **2** could be further evidenced by analysis of its ^1^H–^1^H COSY and HMBC spectra. There were three independent spin fragments from the ^1^H–^1^H COSY spectrum: **a** (H_2_-2/H_2_-3/H-4), **b** (H-22/H_2_-23), and **c** (H-7/H_2_-8/H-9/H-10/H_2_-11/H_2_-12/H_2_-13/H_2_-14/H_2_-15/H_3_-16) as shown in Fig. 2. A close comparison of the ^1^H–^1^H COSY correlations of **2** with those of compound **1** could readily find the same fragments **a** and **b**. Moreover, the HMBC correlations in the rings A–C of **2** were also almost the same as those of **1**. The aforementioned results indicated that compound **2** might share a similar 6/6/5 skeleton (rings A–C) as that of **1**.

However, the chemical shift of C-5 at *δ*_C_ 104.5 in **1** was downshifted to *δ*_C_ 198.5 in **2**, it suggested that the ketal functionality in **1** was replaced by a carboxyl unit in **2**. In addition, the non-proton olefinic carbons in **2** were one less than those in **1**, which might be attributed to the change of olefinic C-7 carbon (*δ*_C_ 145.8) in **1** to a methine one (*δ*_C_ 25.3) in **2**. All these aforementioned facts indicated that compound **2** was a D/E ring-opening derivative of **1**. Notably, the HMBC correlations from H-7 to C-5/C-9, H_2_-8 to C-6/C-17 and a lack of HMBC correlations from H-9 and H-10 to C-5 confirmed the aforementioned conclusion. Therefore, the planar structure of **2** was established (Fig. 2), and it was tentatively suggested to be a critical biosynthetic precursor for **1**.

Similarly, we tried to determine the relative configuration of **2** by analyzing its NOESY spectrum, whereas there were not any obvious diagnostic NOESY correlations being observed. Thus, the determination of the relative configuration of compound **2** through the NOESY experiment seemed to be bleak. Therefore, an extensive combination of chemical transformation, Mo_2_(OAc)_4_-induced electronic circular dichroism (Mo-ICD) spectrum, GIAO DFT ^13^C NMR calculations, and theoretical ECD calculation were applied to solve the stereochemistry of compound **2**.

**Fig. 6:**
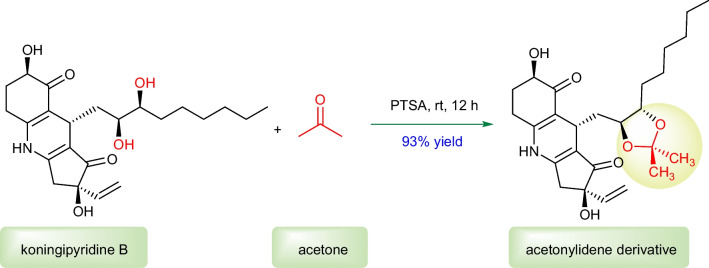
Chemical transformation of **2**

**Fig. 7:**
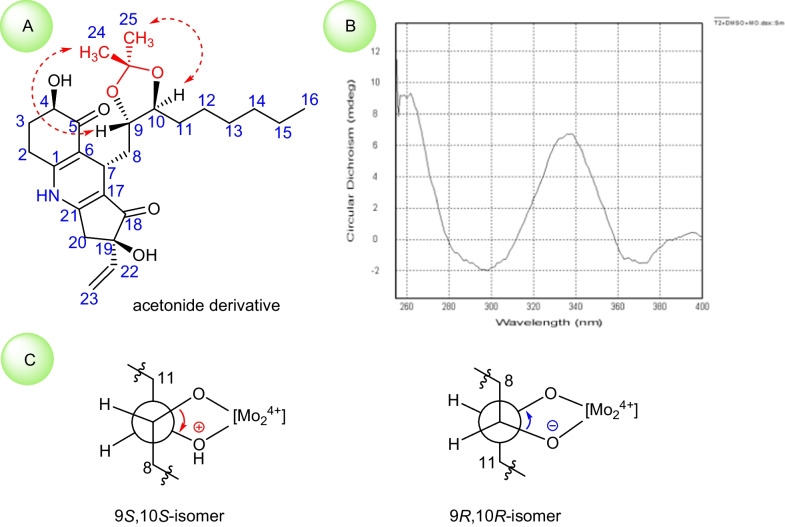
Configuration determination of the 9,10-diol in **2**

Firstly, with the consideration of the presence of 9,10-vicinal diol in **2** from a biogenetic synthetic perspective, the absolute configuration of C-9/C-10 of **2** was tentatively deduced to be the same as that of compound **1**. In order to provide much more direct evidence, the chemical transformation of the 9,10-vicinal diol with acetone to generate the five-membered ketal ring was tried to determine the relative configuration. The treatment of compound **2** with acetone in the presence of a catalytic amount of PTSA (*p*-toluenesulfonic acid) obtained its acetonylidene derivative (Fig. 6), which possessed a *trans*-acetonylidene configuration on the basis of the critical NOESY correlations of H-9/H_3_-24 and H-10/H_3_-25 (Fig. 7A). Therefore, compound **2** was revealed to be a *threo*-9,10-diol.

Then, Snatzke and Frelek’s methodology was applied to establish its absolute configuration with the aid of the Mo-ICD spectrum [46–53]. As the results compiled in Fig. 7B, the obvious positive Cotton effects at 338 and 400 nm originated from the positive (Mo–*O*)-C–C-(*O*–Mo) torsion angle. The careful analyses of the locally favored conformations for the two isomers of the *threo*-9,10-diol, it could be readily found that the 9*S*,10*S*-isomer featured an unambiguously positive torsion angle, whereas the 9*R*,10*R*-isomer owned a negative one (Fig. 7C). Therefore, the 9*S*,10*S* configuration of **2** was assigned.

**Fig. 8:**
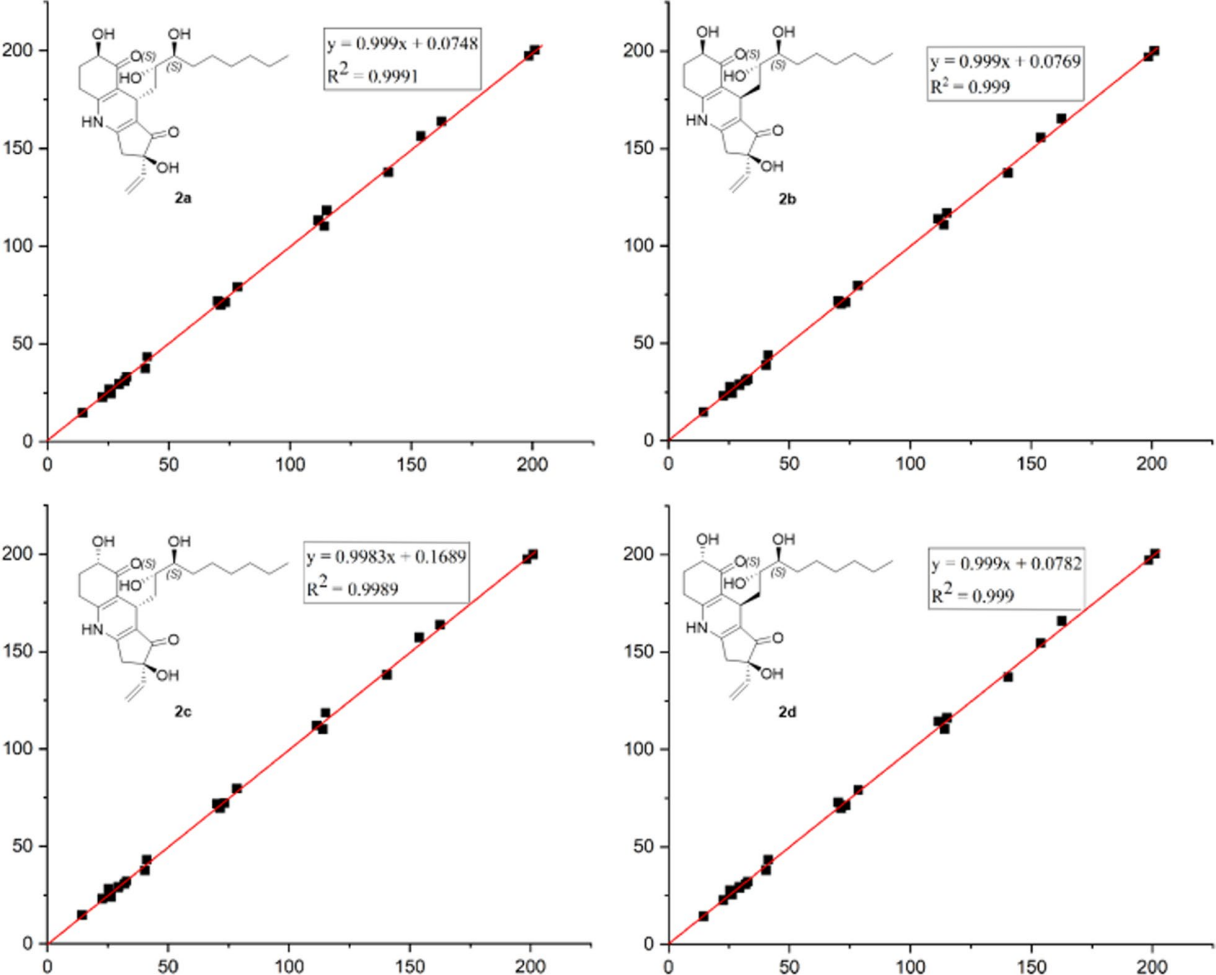
Regression analyses of experimental and calculated ^13^C NMR chemical shifts for **2a**-**2d**

Although the absolute configuration of 9,10-vicinal diol was fortunately determined by chemical transformation and Mo-ICD methodology, the remaining three unassigned chiral centers still led to eight possible related diastereoisomers. In order to further confirm the relative configuration of **2**, the GIAO-DFT ^13^C NMR calculations towards four candidate structures **2a**–**2d** were also attempted. Unfortunately, the relative configuration of **2** could not be completely addressed by ^13^C NMR calculations due to the *P*_rel_ values of **2a** (48.20%) and **2d** (43.36%) as shown in Table 3. Moreover, the *R*^2^ values for the linear regression of ^13^C NMR data were revealed to be 0.9991 for **2a** and 0.999 for **2d** (Fig. 8), which strongly indicated that both **2a** and **2d** or their enantiomers might be the correct structure for **2**.

**Table 3:** Calculated ^13^C chemical shifts (DMSO-*d*_6_) of structures **2a**–**2d** fitting to the experimental data of

No	Exptl. ***δ***	**2**							
		**2a**	abs dev^c^	**2b**	abs dev^c^	**2c**	abs dev^c^	**2d**	abs dev^c^
1	153.9	156.28	2.38	155.61	1.71	157.15	3.25	154.71	0.81
2	25.7	25.99	0.29	25.97	0.27	25.68	0.02	25.67	0.03
3	29.3	29.56	0.26	29.11	0.19	29.80	0.40	29.53	0.23
4	71.4	69.99	1.41	70.14	1.26	69.76	1.64	69.86	1.54
5	198.5	197.26	1.24	196.99	1.51	197.33	1.17	197.09	1.41
6	111.5	113.25	1.75	113.80	2.30	112.00	0.50	114.46	2.96
7	25.3	26.93	1.63	27.81	2.51	28.29	2.99	27.64	2.34
8	41.1	43.47	2.37	43.89	2.79	43.12	2.02	43.36	2.26
9	70.2	71.92	1.72	71.73	1.53	71.95	1.75	72.77	2.57
10	73.3	71.25	2.05	71.12	2.18	72.31	0.99	71.36	1.94
11	32.7	33.04	0.34	31.72	0.98	32.30	0.40	32.19	0.51
12	26.2	24.73	1.47	24.28	1.92	24.09	2.11	25.40	0.80
13	29.4	29.33	0.07	28.66	0.74	29.37	0.03	29.06	0.34
14	31.9	31.07	0.83	30.89	1.01	30.91	0.99	30.90	1.00
15	22.6	22.95	0.35	23.13	0.53	23.21	0.61	22.67	0.07
16	14.4	14.75	0.35	14.80	0.40	14.90	0.50	14.53	0.13
17	115.1	118.23	3.13	117.13	2.03	118.58	3.48	116.15	1.05
18	201.0	200.32	0.68	200.04	0.96	199.85	1.15	200.57	0.43
19	78.4	79.16	0.76	79.70	1.30	79.80	1.40	79.24	0.84
20	40.3	37.52	2.78	38.87	1.43	37.75	2.55	38.21	2.09
21	162.5	164.02	1.52	165.45	2.95	163.63	1.13	165.94	3.44
22	140.4	137.86	2.54	137.43	2.97	138.04	2.36	137.24	3.16
23	114.0	110.22	3.78	110.84	3.16	110.16	3.84	110.56	3.44
		MAE^a^	1.47	MAE^a^	1.59	MAE^a^	1.53	MAE^a^	1.45
		RMS^b^	1.78	RMS^b^	1.82	RMS^b^	1.89	RMS^b^	1.83
		*P*_mean_	14.31%	*P*_mean_	13.14%	*P*_mean_	12.36%	*P*_mean_	14.25%
		*P*_rel_	48.20%	*P*_rel_	6.78%	*P*_rel_	1.65%	*P*_rel_	43.36%

^a^Mean absolute error; ^b^Root mean square; ^c^Absolute deviation of Calcd *δ*_C_

**Fig. 9:**
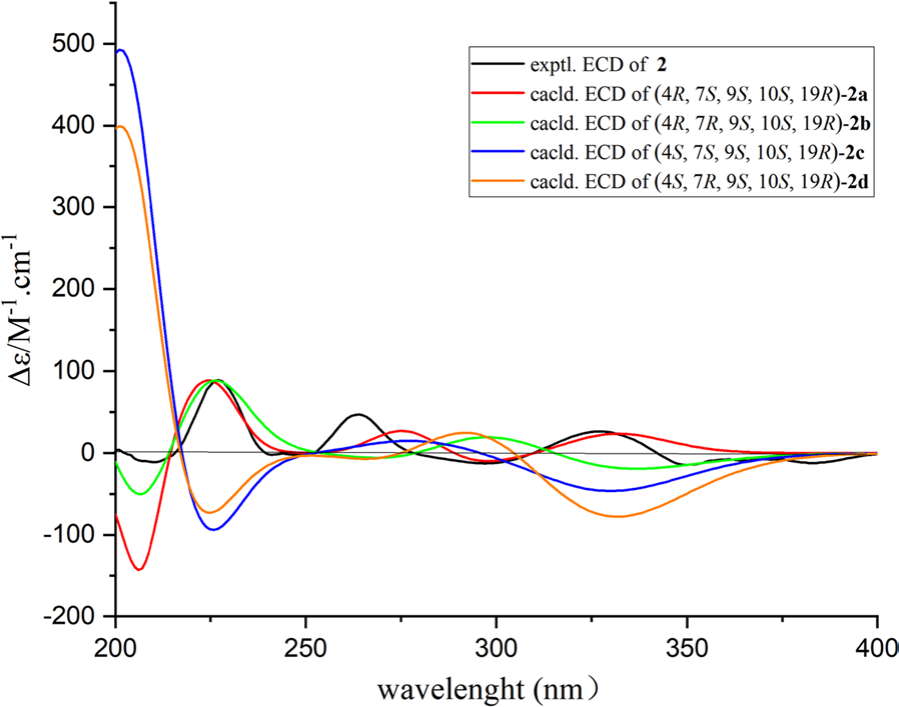
Experimental and calculated ECD spectra of compound **2** (in MeOH)

Subsequently, theoretical ECD calculations for **2a-2d** were further applied to determine the absolute configuration of **2**, it is delightful to find that the theoretical ECD spectrum of 4*R*,7*S*,9*S*,10*S*,19*R*-**2a** was well matched with the experimental one (Fig. 9), and both curves showed obvious positive cotton effects at 230, 260, and 330 nm together with a series of negative cotton effects at 210, 250, 300, and 350 nm. Moreover, the theoretical ECD calculations also supported the two potential probable structures **2a** and **2d** deduced by the GIAO DFT ^13^C NMR calculations. Collectively, the aforementioned experimental results thus disclosed the absolute structure of **2** as 4*R*,7*S*,9*S*,10*S*,19*R*.

Interestingly, the extensively deduced absolute configuration of **2** was consistent with that of its derivative **1**. From the perspective of biosynthesis, the consistent configuration of compounds **1** and **2** further proved the reliability of the aforementioned results. Therefore, compound **2** was identified as the first member in the koninginin family sharing a unique 6/6/5 dihydropyridine skeleton with an absolute configuration of 4*R*,7*S*,9*S*,10*S*,19*R*, and it was given the trivial name koningipyridine B.

Moreover, four koninginin derivatives were also isolated from the endophytic fungus *T. koningiopsis* SC-5 and identified as koninginin D (**3**) [29], koninginin E (**4**) [30], koninginin F (**5**) [31], and 7-*O*-methylkoninginin D (**6**) [3] by a careful comparison of their spectral data with the published data.

**Scheme 1:**
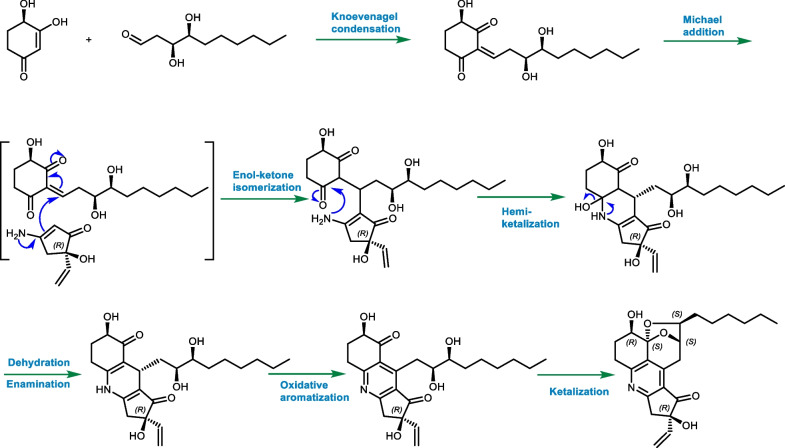
Plausible biosynthetic pathway of **1** and **2**

The novel scaffold of **1** was evidenced to feature an unprecedented pentacyclic ketal skeleton with the formation of a fascinating 6/6/5/6/5 fused ring system and shared a characteristic pyridine core, which represents the first example of nitrogen-containing koninginin type natural product. While, koningipyridine B was revealed to be the first member in koninginin family sharing a unique 6/6/5 dihydropyridine skeleton, and it was probably a critical biosynthetic precursor of koningipyridine A as proposed in the plausible biosynthetic pathway shown in Scheme 1.

Subsequently, all isolates were tested for antibacterial activities towards *S. aureus*, MRSA (Methicillin-resistant *S. aureus*), and *E. coli* by the microbroth dilution method [54], whereas they did not exhibit any notable antibacterial effect at the concentration of 100 μg/mL. In addition, compounds **1˗6** had been screened for cytotoxic activity towards three human cancer cell lines (A549, Hela, and HepG2) by MTT assay [55] as shown in Additional File 1: Table S1, and all of them showed weak activities.

## Conclusion

It is undeniable that endophytic fungi have played a great role in the discovery of fascinating secondary metabolites with novel structures and significant biological activities in recent years, there are numerous endophytic fungi that grow in special environments, which still have great potential in the exploration of the pharmaceutically meaningful lead compounds. This research carried out a systematic chemical study on the endophytic fungus *T. koningiopsis* SC-5 from *Pedicularis integrifolia*, and led to the isolation of two novel nitrogen-containing polyketides koningipyridines A and B (**1** and **2**), which featured an unprecedented pentacyclic ketal skeleton with the formation of a complex 6/6/5/6/5 fused ring skeleton and a unique 6/6/5 dihydropyridine scaffold, respectively. Notably, both koningipyridines A (**1**) and B (**2**) shared a characteristic pyridine core, which had been reported in the koninginin family for the first time. The current study did not only enrich the structural diversity of bioactive koninginin derivatives, but also provide useful theoretical references and experimental guidance for the related research in the future. Biologically, all the tested isolates only showed weak cytotoxicities to the selected three human cancer cell lines. Therefore, more biological activities including antifungal, anti-HIV, immunoregulation, and so on will be appealed in the future due to the fascinating structures of koningipyridines A and B.

## Experimental

### General experimental procedures

See the Additional File 1.

### Fungal material

The strain of *Trichoderma koningiopsis* SC-5 was isolated from *Pedicularis integrifolia*, which had been collected in July 2021 in Li County, Sichuan Province. The sequence analysis of the rDNA* ITS* region was used to identify this endophytic strain. Its rDNA *ITS* sequence was uploaded to GenBank with the accession of OP646773. The sample of *T. koningiopsis* SC-5 was preserved in the Department of Medicinal Chemistry, Central South University.

### Fermentation, extraction, and isolation

*T. koningiopsis* SC-5 was fermented in autoclaved rice solid medium (120 × 500 mL Erlenmeyer flasks, each containing 100 g grain and 120 mL water). The fungal culture was carried out in a static condition at 28 °C for four weeks. Then, they were extracted with EtOAc for three times and concentrated to obtain the crude products (110.0 g).

The crude extract was transferred to flash column chromatography (FCC) with the use of macroporous resin and eluted by EtOH-H_2_O (v/v, 20:80–0:100) to afford three fractions (Fr. 1 to Fr. 3). The silica gel FCC with CH_2_Cl_2_-MeOH (v/v, 100:1–0:100) as the eluent was used to purify the Fr. 2 (45 g) to further obtain seven subfractions (Fr. 2–1 to Fr. 2–7). Fr. 2–4 (8.5 g) was purified by use of reversed-phase ODS FCC with MeOH-H_2_O (v/v, 5:95–100:0), thus giving rise to eleven fractions (Fr. 2–4-1 to Fr. 2–4-11). Fr. 2–4-7 was separated through prep-HPLC with CH_3_CN-H_2_O (0–35 min, 45–65%, 2 mL/min) to provide **1** (18.2 mg, t_*R*_ = 25.2 min). Fr. 2–4-2 was isolated through Sephadex LH-20 column with MeOH-H_2_O (v/v, 80:20) to generate four fractions (Fr. 2–4-2–1 to Fr. 2–4-2–4), and Fr. 2–4-2–1 was prepared by prep-HPLC by using CH_3_CN-H_2_O (0–30 min, 15–30%, 2 mL/min) as eluent to give **2** (27.6 mg, t_*R*_ = 27.2 min). Moreover, Fr. 2–4-5 was separated by prep-HPLC eluting with CH_3_CN-H_2_O (0–40 min, 30–50%, 2 mL/min) to provide compounds **3** (12.4 mg, t_*R*_ = 26.8 min), **5** (4.6 mg, t_*R*_ = 28.6 min), and **6** (16.7 mg, t_*R*_ = 34.8 min), Fr. 2–3 (4.2 g) was purified through reversed-phase ODS FCC eluting with MeOH-H_2_O (v/v, 5:95–100:0) to offer 9 fractions (Fr. 2–3-1 to Fr. 2–3-9), Fr. 2–3-6 was further separated with prep-HPLC by CH_3_CN-H_2_O (0–30 min, 25–35%, 2 mL/min) to afford **4** (5.3 mg, t_*R*_ = 27.8 min).

Koningipyridine A (**1**). Yellow oil; [*α*]20 D + 6.9 (*c* 0.9, MeOH); UV (MeOH): *λ*_max_ (log *ε*): 215 (5.08), 246 (2.81), 299 (2.14) nm; IR (KBr): 3418, 2929, 2858, 1725, 1579, 1514, 1426, 1384, 1286, 1121, 1074, 1019, 729, and 562 cm^−1^; ECD (0.87 mg/mL, MeOH): *λ*_max_ (Δ*ε*) 206 (+ 55.63), 220 (− 27.65), 245 (+ 36.07), 278 (− 17.71), 361 (+ 3.21) nm; ^1^H (CD_3_OD, 600 MHz) and ^13^C (CD_3_OD, 150 MHz) NMR spectral data, see Table 1; HRESIMS: *m/z* 400.2117 [M + H]^+^ (calcd for C_23_H_30_NO_5_^+^, 400.2118).

Koningipyridine B (**2**). Yellow oil; [*α*]20 D − 9.2 (*c* 1.9, MeOH); UV (MeOH): *λ*_max_ (log *ε*): 241 (4.26), 369 (3.82) nm; IR (KBr): 3399, 3065, 2929, 2857, 1726, 1633, 1485, 1384, 1259, 1198, 1121, 1038, 1020, 921, and 631 cm^−1^; ECD (1.89 mg/mL, MeOH): *λ*_max_ (Δ*ε*) 211 (− 11.06), 227 (+ 89.28), 249 (− 2.44), 264 (+ 47.33), 297 (− 12.57), 327 (+ 26.45), 350 (− 14.56) nm; ^1^H (DMSO-*d*_6_, 600 MHz) and ^13^C (DMSO-*d*_6_, 150 MHz) NMR spectral data, see Table 3; HRESIMS: *m/z* 420.2394 [M + H]^+^ (calcd for C_23_H_34_NO_6_^+^, 420.2386), 442.2224 [M + Na]^+^ (calcd for C_23_H_33_NO_6_Na^+^, 442.2206).

### ^13^C NMR and ECD calculations methods

The conformation search and optimization, DFT GIAO ^13^C NMR, and ECD calculations of compounds **1** and **2** were performed as previously described [40]. The detailed computational method, DFT optimized geometry data, relative energies, and conformational population of all calculated structures were attached in the Additional file 1.

### Antibacterial and cytotoxic activity assay

See the Additional file 1.

### Supplementary Information

**Additional file 1.** Additional section, figures and tables.

## Data Availability

All relevant data are within the manuscript and its Additional files.

## References

[1] Aly AH, Debbab A, Proksch P (2011). Fungal endophytes: unique plant inhabitants with great promises. Appl Microbiol Biotechnol..

[2] Liu JJ, Liu G (2018). Analysis of secondary metabolites from plant endophytic fungi. Methods Mol Biol.

[3] Song FH, Dai HQ, Tong YJ, Ren B, Chen C, Sun N (2010). Trichodermaketones A-D and 7-O-methylkoninginin D from the marine fungus Trichoderma koningii. J Nat Prod.

[4] Sun Y, Tian L, Huang J, Ma HY, Zheng Z, Lv A (2008). Trichodermatides A-D, novel polyketides from the marine-derived fungus Trichoderma reesei. Org Lett.

[5] Shi XS, Li HL, Li XM, Wang DJ, Li X, Meng LH (2020). Highly oxygenated polyketides produced by *Trichoderma koningiopsis* QA-3, an endophytic fungus obtained from the fresh roots of the medicinal plant *Artemisia argyi*. Bioorg Chem.

[6] Chavez JR, Raja HA, Gra TN, Gallagher JM, Metri P, Xue D (2017). Prealamethicin F50 and related peptaibols from *Trichoderma arundinaceum*: validation of their authenticity via in situ chemical analysis. RSC Adv.

[7] Miao FP, Liang XR, Yin XL, Wang G, Ji NY (2012). Absolute configurations of unique harziane diterpenes from *Trichoderma species*. Org Lett.

[8] Chen SC, Li HH, Chen YC, Li SN, Xu JL, Guo H (2019). Three new diterpenes and two new sesquiterpenoids from the endophytic fungus *Trichoderma **koningiopsis* A729. Bioorg Chem..

[9] Shi XS, Meng L, Li X, Wang DJ, Zhou XW, Du FY (2020). Polyketides and terpenoids with potent antibacterial activities from the *Artemisia argyi*-derived fungus *Trichoderma koningiopsis* QA-3. Chem Biodivers.

[10] Song YP, Miao FP, Fang ST, Yin XL, Ji NY (2018). Halogenated and nonhalogenated metabolites from the marine-alga-endophytic fungus *Trichoderma asperellum* cf44-2. Mar Drugs.

[11] Li MF, Li GH, Zhang KQ (2019). Non-volatile metabolites from Trichoderma spp. Metabolites.

[12] Zhou P, Wu ZD, Tan DD, Yang J, Zhou Q, Zeng FR (2017). Atrichodermones A-C, three new secondary metabolites from the solid culture of an endophytic fungal strain *Trichoderma atroviride*. Fitoterapia.

[13] Hu X, Gong MW, Zhang WW, Zheng QH, Liu QY, Chen L (2014). Novel cytotoxic metabolites from the marine-derived fungus *Trichoderma citrinoviride*. Heterocycles.

[14] Ding G, Wang HL, Li L, Chen AJ, Chen L, Chen H (2012). Trichoderones A and B: two pentacyclic cytochalasans from the plant endophytic fungus *Trichoderma gamsii*. Eur J Org Chem.

[15] Reino JL, Guerrero RF, Hernández-Galán R, Collado IG (2008). Secondary metabolites from species of the biocontrol agent *Trichoderma*. Phytochemistry.

[16] El-Hasan A, Walker F, Schone J, Buchenauer H (2009). Detection of viridiofungin A and other antifungal metabolites excreted by *Trichoderma **harzianum* active against different plant pathogens. Eur J Plant Pathol..

[17] Stoppacher N, Kluger B, Zeilinger S, Krska R, Schuhmacher R (2010). Identification and profiling of volatile metabolites of the biocontrol fungus *Trichoderma **atroviride* by HS-SPME-GC-MS. J Microbiol Methods..

[18] Mukherjee M, Mukherjee PK, Horwitz BA, Berg CG, Zeilinger S (2012). *Trichoderma*-plant-pathogen interactions: advances in genetics of biological control. Indian J Microbiol..

[19] Khan RAA, Najeeb S, Hussain S, Xie B, Li Y (2020). Bioactive secondary metabolites from *Trichoderma* spp. against phytopathogenic fungi. Microorganisms.

[20] Vinale F, Sivasithamparam K, Ghisalberti EL, Ruocco M, Wood S, Lorito M (2012). *Trichoderma* secondary metabolites that affect plant metabolism. Nat Prod Commun..

[21] Liu K, Yang YB, Miao CP, Zheng YK, Chen JL, Chen YW (2016). Koningiopisins A-H, polyketides with synergistic antifungal activities from the endophytic fungus Trichoderma koningiopsis. Planta Med.

[22] Wang YL, Hu BY, Qian MA, Wang ZH, Zou JM, Sang XY (2021). Koninginin W, a new polyketide from the endophytic fungus *Trichoderma koningiopsis* YIM PH30002. Chem Biodivers.

[23] Cutler HG, Cutler SJ, Ross SA, Sayed KE, Dugan FM, Bartlett MG (1999). Koninginin G, a new metabolite from *Trichoderma **aureoviride*. J Nat Prod.

[24] Hu M, Li QL, Yang YB, Liu K, Miao CP, Zhao LX (2017). Koninginins R-S from the endophytic fungus *Trichoderma **koningiopsis*. Nat Prod Res.

[25] Liu K, Yang YB, Chen JL, Miao CP, Wang Q, Zhou H (2016). Koninginins N-Q, polyketides from the endophytic fungus *Trichoderma **koningiopsis* harbored in Panax notoginseng. Nat Prod Bioprospect..

[26] Lang BY, Li J, Zhou XX, Chen YH, Yang YH, Li XN (2015). Koninginins L and M, two polyketides from *Trichoderma koningii* 8662. Phytochem Lett.

[27] Zhou XX, Li J, Yang YH, Zeng Y, Zhao PJ (2014). Three new koninginins from *Trichoderma **neokongii* 8722. Phytochem Lett..

[28] Shi XS, Wang DJ, Li XM, Li HL, Meng LH, Li X (2017). Antimicrobial polyketides from *Trichoderma **koningiopsis* QA-3, an endophytic fungus obtained from the medicinal plant *Artemisia **argyi*. RSC Adv.

[29] Dunlop RW, Simon A, Sivasithamparam K, Ghisalberti EL (1989). An antibiotic from *Trichoderma **Koningii* active against soilborne plant pathogens. J Nat Prod.

[30] Parker SR, Cutler HG, Schreiner PR (1995). Koninginin E: isolation of a biologically active natural product from *Trichoderma **koningii*. Biosci Biotechnol Biochem..

[31] Ghisalberti EL, Rowland CY (1993). Antifungal metabolites from *Trichoderma **harzianum*. J Nat Prod.

[32] Kang FH, Lu XX, Zhang S, Chen DK, Kuang M, Peng WW (2021). Diaportones A-C: three new metabolites from endophytic fungus *Diaporthe foeniculina* BZM-15. Front Chem.

[33] Lu XX, Zhang YJ, Zhang WG, Wang H, Zhang J, Wang SS (2021). Cyclohexanone and phenolic acid derivatives from endophytic fungus *Diaporthe foeniculina*. Front Chem.

[34] Zhang S, Chen DK, Kuang M, Peng WW, Chen Y, Tan JB (2021). Rhytidhylides A and B, two new phthalide derivatives from the endophytic fungus *Rhytidhysteron* sp. BZM-9. Molecules.

[35] Zhang S, Wang WX, Tan JB, Kang FH, Chen DK, Xu KP (2021). Rhytidhyesters A-D, 4 new chlorinated cyclopentene derivatives from the endophytic fungus *Rhytidhysteron* sp. BZM-9. Planta Med.

[36] Zhang WG, Lu XX, Huo LQ, Zhang S, Chen Y, Zou ZX (2021). Sesquiterpenes and steroids from an endophytic *Eutypella** scoparia*. J Nat Prod.

[37] Zhang WG, Lu XX, Wang H, Chen Y, Zhang J, Zou ZX (2021). Antibacterial secondary metabolites from the endophytic fungus *Eutypella scoparia* SCBG-8. Tetrahedron Lett.

[38] Peng WW, Kuang M, Huang YT, Li MF, Zheng YT, Xu L (2022). Pseudocercones A-C, three new polyketide derivatives from the endophytic fungus *Pseudocercospora* sp. TSS-1. Nat Prod Res.

[39] Kuang M, Peng WW, Huang YT, Li MF, Qin SY, Zheng YT (2022). Two new chromone derivatives from the rhizosphere soil fungus *Ilyonectria** robusta*. Nat Prod Res.

[40] Chen Y, Wang H, Ke X, Sang ZH, Kuang M, Peng WW (2022). Five new secondary metabolites from an endophytic fungus *Phomopsis* sp. SZSJ-7B. Front Plant Sci.

[41] Cutler HG, Himmelsbach DS, Arrendale RF, Cole PD, Cox RH (1989). Koninginin A—a novel plant-growth regulator from *Trichoderma-**koningii*. Agr Biol Chem Tokyo..

[42] Ditchfield R (1972). Molecular orbital theory of magnetic shielding and magnetic susceptibility. J Chem Phys.

[43] McWeeny R (1961). Perturbation theory for the fock-dirac density matrix. Phys Rev.

[44] Chai JD, Head-Gordon M (2008). Long-range corrected hybrid density functionals with damped atom-atom dispersion corrections. Phys Chem Chem Phys..

[45] Li J, Liu JK, Wang WX (2020). GIAO ^13^C NMR calculation with sorted training sets improves accuracy and reliability for structural assignation. J Org Chem.

[46] Snatzke G (1979). Circular dichroism and absolute conformation: application of qualitative MO theory to chiroptical phenomena. Angew Chem Int Ed Engl..

[47] Frelek J, Snatzke G (1983). Circulardichroismus-LXXX: bestimmung der absoluten konfiguration von 1-substituierten glycerin-derivaten und anderen aliphatischen vic-glykolen im mikromaßstab. Z Anal Chem.

[48] Snatzke G, Wagner U, Wolff HP (1981). Circulardichroism-LXXV1: Cottonogenic derivatives of chiral bidentate ligands with the complex [Mo_2_ (O_2_CCH_3_)_4_]. Tetrahedron.

[49] Frelek J, Klimek A, Ruskowska P (2003). Dinuclear transition metal complexes as auxiliary chromophores in chiroptical studies on bioactive compounds. Curr Org Chem..

[50] Politi M, De-Tommasi N, Pescitelli G, Di-Bari L, Morelli I, Braca A (2002). Structure and absolute configuration of new diterpenes from *Lavandula **multifida*. J Nat Prod.

[51] Gao Y, Duan FF, Liu L, Peng XG, Meng XG, Ruan HL (2021). Hypothemycin-type resorcylic acid lactones with immunosuppressive activities from a *Podospora* sp. J Nat Prod.

[52] Di-Bari L, Pescitelli G, Pratelli C, Pini D, Salvadori P (2021). Determination of absolute configuration of acyclic 1,2-diols with Mo2(OAc)4 1 Snatzke's method revisited. J Org Chem.

[53] Jo MS, Lee S, Yu JS, Baek SC, Cho YC, Kim KH (2020). Megastigmane derivatives from the cladodes of *Opuntia **humifusa* and their nitric oxide inhibitory activities in macrophages. J Nat Prod.

[54] Zhao LY, Liu HX, Huo LQ, Wang MM, Yang B (2018). Structural optimization and antibacterial evaluation of rhodomyrtosone B analogues against MRSA strains. Medchemcomm..

[55] McCauley J, Zivanovic A, Skropeta D (2013). Bioassays for anticancer activities. Methods Mol Biol.

