# Supplementary material for: Koningipyridines A and B, two nitrogen-containing polyketides from the fungus Trichoderma koningiopsis SC-5
Source: Nat Prod Bioprospect. 2024 Jan 11;14(1):8. doi: 10.1007/s13659-024-00429-z (PMC10784257; doi:10.1007/s13659-024-00429-z)
Supplement: Supplementary file 1 — Additional file 1. Additional section, figures and tables. [file 13659_2024_429_MOESM1_ESM.pdf]

Supporting Information

Koningipyridines A and B, two nitrogen-containing polyketides from fungus *Trichoderma koningiopsis* SC-5

Weiwei Peng[1,3†], Qi Huang[1,4†], Xin Ke[1,3†], Wenxuan Wang[1], Yan Chen[1,3], Zihuan Sang[1,3], Chen Chen[1,3], Siyu Qin[1], Yuting Zheng[1], Haibo Tan[1,2,3,*] Zhenxing Zou[1,*]

[1]Xiangya School of Pharmaceutical Sciences, Hunan Key Laboratory of Diagnostic and Therapeutic Drug Research for Chronic Diseases, Central South University, Changsha 410013, People's Republic of China.
[2]National Engineering Research Center of Navel Orange, Gannan Normal University, Ganzhou 341000, People's Republic of China.
[3]Key Laboratory of South China Agricultural Plant Molecular Analysis and Genetic Improvement, Guangdong Provincial Key Laboratory of Applied Botany, South China Botanical Garden, Chinese Academy of Sciences, Guangzhou 510650, People's Republic of China.
[4]Department of Pharmacy, Xiangya Hospital, Central South University, Changsha 410013, People's Republic of China.

[*] **Correspondence:** tanhaibo@scbg.ac.cn (H.-B. Tan), zouzhenxing@csu.edu.cn (Z.-X. Zou)
[†]These authors contributed equally to this work.

Contents

Experimental section..........1

1.1 General experimental procedures..........1

1.2 Antibacterial assay..........1

1.3 Cytotoxic assay..........2

Table S1. Cytotoxic activities of compounds **1-6**...........2

1.4 ^{13}C NMR and ECD calculations..........2

Table S2. Geometry data of conformers of structure **1a**...........3

Table S3. Geometry data of conformers of structure **1b**..........153

Table S4. Geometry data of conformers of structure **2a**...........311

Table S5. Geometry data of conformers of structure **2b**..........357

Table S6. Geometry data of conformers of structure **2c**...........490

Table S7. Geometry data of conformers of structure **2d**..........592

Attached figures of compounds 1-6..........709

Figure S1. HRESIMS spectrum of compound **1**..........709

Figure S2. UV spectrum of compound **1**..........709

Figure S3. IR spectrum of compound **1**..........710

Figure S4. ^{1}H NMR spectrum (600 MHz, CD_3OD) of compound **1**..........710

Figure S5. ^{13}C NMR spectrum (150 MHz, CD_3OD) of compound **1**..........711

Figure S6. DEPT 135 spectrum of compound **1** recorded in CD_3OD..........711

Figure S7. ^{1}H-^{1}H COSY spectrum of compound **1** recorded in CD_3OD..........712

Figure S8. HSQC spectrum of compound **1** recorded in CD_3OD..........712

Figure S9. HMBC spectrum of compound **1** recorded in CD_3OD..........713

Figure S10. NOESY spectrum of compound **1** recorded in CD_3OD..........713

Figure S11. CD spectrum of compound **1**..........714

Figure S12. HRESIMS spectrum of compound **2**..........715

Figure S13. UV spectrum of compound **2**..........715

Figure S14. IR spectrum of compound **2**..........716

Figure S15. ^{1}H NMR spectrum (600 MHz, DMSO-d_6) of compound **2**..........716

Figure S16. ^{13}C NMR spectrum (150 MHz, DMSO-d_6) of compound **2** 717

Figure S17. DEPT 135 spectrum of compound **2** recorded in DMSO-d_6..................... 717

Figure S18. ^{1}H-^{1}H COSY spectrum of compound **2** recorded in DMSO-d_6 718

Figure S19. HSQC spectrum of compound **2** recorded in DMSO-d_6........................... 718

Figure S20. HMBC spectrum of compound **2** recorded in DMSO-d_6.......................... 719

Figure S21. NOESY spectrum of compound **2** recorded in DMSO-d_6 719

Figure S22. ^{1}H NMR spectrum (500 MHz, $CDCl_3$) of acetonylidene derivative of **2** . 720

Figure S23. NOESY spectrum of acetonylidene derivative of **2** recorded in $CDCl_3$ ... 720

Figure S24. CD spectrum of compound **2** 721

Figure S25. Mo-ICD spectrum of compound **2** 722

Figure S26. ^{1}H NMR spectrum (600 MHz, $CDCl_3$) of compound **3** 722

Figure S27. ^{13}C NMR spectrum (150 MHz, $CDCl_3$) of compound **3** 723

Figure S28. ^{1}H NMR spectrum (600 MHz, CD_3OD) of compound **4** 723

Figure S29. ^{13}C NMR spectrum (150 MHz, CD_3OD) of compound **4** 724

Figure S30. ^{1}H NMR spectrum (600 MHz, $CDCl_3$) of compound **5** 724

Figure S31. ^{13}C NMR spectrum (150 MHz, $CDCl_3$) of compound **5** 725

Figure S32. ^{1}H NMR spectrum (600 MHz, CD_3OD) of compound **6** 725

Figure S33. ^{13}C NMR spectrum (150 MHz, CD_3OD) of compound **6** 726

References 726

Experimental section

1.1 General experimental procedures

HRESIMS data were obtained with Agilent 6500 series Q-TOF mass spectrometer analyser (Agilent, Singapore) with positive ion mode. 1D and 2D NMR spectra were measured on a Bruker AVIII-600/500 spectrometer (Bruker, Karlsruhe, Germany) and using TMS as an internal standard. Optical rotations were measured with an Anton Paar MCP-500 spectropolarimeter (Anton Paar, Graz, Austria). Experimental ECD spectra were acquired from Applied PhotoPhysics Chiascan circular dichroism spectrometer (APL, Britain). The reversed-phase preparative HPLC was conducted using an Agilent 1100 (Agilent Technologies, California, USA) instrument connected to an Innoval ODS-2 column (5 μm, 10 mm × 250 mm) with a VWD detector. The UV spectra were recorded with the use of a UV-2600 spectrophotometer (Shimadzu, Kyoto, Japan). The IR data for the tested compounds were recorded on the PerkinElmer FT-IR spectrometer (PerkinElmer, Massachusetts, USA). Silica gel (80-100 and 200-300 mesh, Qingdao Marine Chemical Factory, Qingdao, China), macroporous resin (HPD-100, Hebei, China), Sephadex LH-20 (Toyopearl Tosoh, Tokyo, Japan) and C_{18} reversed-phase silica gel (50 μm, Fuji, Kasugai, Japan) were used for column chromatography. All solvents were of analytical grade.

1.2 Antibacterial assay

The antibacterial effects for all the tested isolates against *S. aureus*, MRSA, and *E. coli* were evaluated by the use of the broth microdilution methodology referring to CLSI guidance on 96-well microplates. The positive control was vancomycin, and the negative control was DMSO. The MIC value was considered as the lowest inhibitory concentration for the visible growth of the tested bacteria.

None of the compounds exhibited antibacterial activity at the maximum concentration (100 ug/mL) designed for the experiment.

1.3 Cytotoxic assay

The cytotoxicities of all isolates were screened by MTT assay.90 μL suspended cells (1 × 10^5 cells/mL) were transformed to every well in the 96-well microplate and incubated at 37 °C under 5% CO_2 atmosphere. 24 h later, 10 μL tested isolates with a concentration of 500 μM in DMSO were added. After culturing for another 48 h, 15 μL MTT agent (5 mg/mL) in DMSO was then added and cultured for 4 hours. Finally, the formazan crystals was dissolved by the use of 100 μL DMSO after removing the supernatant from each well, and the absorbance was measured at 490 nm to calculate the inhibitory rates. The experiments were carried out in triplicate.

Table S1. Cytotoxic activities of compounds **1-6**.

Compounds (50 μM)	Inhibition rate		
	A549	Hela	HepG2
**1**	18.37%	8.01%	26.99%
**2**	11.28%	19.35%	19.88%
**3**	9.43%	6.02%	11.42%
**4**	15.42%	3.72%	19.03%
**5**	13.20%	1.45%	18.60%
**6**	18.47%	6.15%	38.71%
Adriamycin (25 μM)	83.50%	83.20%	94.50%

1.4 ^{13}C NMR and ECD calculations

Conformational search was performed by Crest (Pracht et al., 2020), followed by optimization on GFN2-xTB (Bannwarth et al., 2019) level with 4 kcal/mol energy window to remove high energy conformers. Optimization and frequency calculation of each conformer was performed on B3LYP-D3(BJ)/TZVP (Tsuzuki et al., 2020; Grimme et al., 2011) level of theory by using Gaussian16 software package with keyword g09defaults (Frisch et al., 2016). DFT GIAO ^{13}C NMR calculation was calculated on the ωB97x-D/6-31G* level (Chai et al., 2008), and the data processing followed the reported STS protocol (Li et al., 2020). The calculated shielding tensors were Boltzmann averaged based on Gibbs free energy. The low energy conformers obtained in aforementioned ^{13}C NMR calculation section were further calculated by TDDFT on ωB97x-D/TZVP level of theory, and were Boltzmann averaged

according to Gibbs free energy. SpecDis v1.71 (Bruhn et al., 2013) was used to simulate the ECD curves of compounds 1 and 2, with sigma/gamma value of 0.30 eV and 0.20 eV, respectively. The averaged calculated ECD curves of compounds 1 and 2 were adjusted by blue shifting for 16 nm and red shifting for 2 nm, respectively.

Table S2. Geometry data of conformers of structure **1a**.

1a-c82, ΔG = 0.0000 kcal/mol, population = 6.21 %

C 0.416225 -3.653290 0.817081
C 0.905864 -3.040807 -0.484714
H 1.975432 -3.224325 -0.621452
C 0.709603 -1.532023 -0.435321
C -0.688129 -1.123559 -0.006705
C -1.510079 -1.985224 0.732907
N -2.740534 -1.633337 1.150778
C -3.163994 -0.422559 0.819667
C -2.423669 0.485402 0.053787
C -1.139850 0.142926 -0.372065
C -0.273237 1.038212 -1.216195
H -0.216318 2.035638 -0.777893
H -0.740783 1.160112 -2.197327
C 1.120643 0.439138 -1.372029
C 1.913725 0.393056 -0.043010
C 3.402332 0.632181 -0.203859
C 4.183771 0.489502 1.101686
C 5.695150 0.671960 0.937673
C 6.110747 2.075529 0.490269
C 7.624266 2.311634 0.503663
C 8.396381 1.446748 -0.493405
H 8.017960 1.592761 -1.509060
H 8.310902 0.384243 -0.258049
H 9.458919 1.699274 -0.493763
H 7.817900 3.366252 0.285912
H 8.005481 2.132046 1.514882
H 5.630818 2.807839 1.148820
H 5.731785 2.273973 -0.517321
H 6.181309 0.451898 1.894061
H 6.066349 -0.072737 0.226832
H 3.985682 -0.498975 1.522692
H 3.806461 1.219588 1.826962
H 3.527158 1.639367 -0.610414
H 3.789883 -0.067239 -0.951972
H 1.491985 1.094222 0.682019
O 1.675418 -0.956274 0.437737

H 1.677785 0.920798 -2.170919
O 0.992266 -0.958795 -1.702820
C -3.203008 1.704250 -0.133895
O -2.858860 2.782499 -0.578297
C -4.645715 1.424933 0.387794
C -5.521772 1.163711 -0.814759
C -6.553578 1.914227 -1.175974
H -6.854265 2.782045 -0.602492
H -7.139518 1.670734 -2.052902
H -5.244433 0.292542 -1.401544
C -4.490319 0.149438 1.245039
H -4.457752 0.432587 2.298950
H -5.302997 -0.563108 1.110095
O -5.088450 2.513785 1.177771
H -4.861444 3.326008 0.701962
C -1.073845 -3.388849 1.047049
H -1.352687 -3.619375 2.076285
H -1.669721 -4.053027 0.414189
O 0.156774 -3.601196 -1.561455
H 0.412048 -3.137557 -2.369304
H 0.605507 -4.727043 0.804655
H 1.007615 -3.228526 1.630150

1a-c5, ΔG = 0.0922 kcal/mol, population = 5.31 %
C 0.685536 3.390567 -0.958164
C 0.952518 2.946888 0.470588
H 2.004500 3.091988 0.732501
C 0.658003 1.458819 0.597013
C -0.693855 1.064040 0.030135
C -1.345965 1.852576 -0.928096
N -2.526715 1.504174 -1.472545
C -3.071285 0.372174 -1.052022
C -2.508256 -0.453424 -0.071966
C -1.276114 -0.115608 0.489365
C -0.596506 -0.923378 1.562194
H -0.546747 -1.974075 1.272706
H -1.202042 -0.883481 2.472064
C 0.800400 -0.376401 1.839501
C 1.772092 -0.558826 0.648408
C 3.206704 -0.832314 1.056346
C 4.170636 -0.926084 -0.124179
C 5.608965 -1.205031 0.307900
C 6.581862 -1.324396 -0.863628
C 8.022500 -1.595164 -0.431814

C 8.986780 -1.724344 -1.609552
H 8.691239 -2.544565 -2.269147
H 10.007225 -1.918755 -1.272337
H 9.001564 -0.808715 -2.206812
H 8.356776 -0.788185 0.228753
H 8.051240 -2.512078 0.166455
H 6.550202 -0.402801 -1.456268
H 6.248344 -2.128256 -1.529978
H 5.945222 -0.405247 0.977364
H 5.637492 -2.129318 0.896258
H 4.135476 0.006130 -0.694512
H 3.833534 -1.718921 -0.801374
H 3.220157 -1.773922 1.615108
H 3.528391 -0.045000 1.745396
H 1.412615 -1.339472 -0.027162
O 1.689847 0.715629 -0.043218
H 1.206476 -0.765820 2.769161
O 0.724921 1.059089 1.957294
C -3.384444 -1.597798 0.154458
O -3.178395 -2.617717 0.783682
C -4.716212 -1.330161 -0.611983
C -5.740016 -0.869228 0.398337
C -6.867570 -1.508794 0.677337
H -7.142176 -2.424679 0.169255
H -7.559107 -1.123075 1.415298
H -5.489486 0.053335 0.914296
C -4.357884 -0.192265 -1.593210
H -4.193545 -0.618565 -2.584804
H -5.133793 0.567699 -1.674951
O -5.107492 -2.496038 -1.314527
H -5.010026 -3.244625 -0.708185
C -0.776386 3.176290 -1.356104
H -0.902479 3.278770 -2.434816
H -1.400787 3.950619 -0.900849
O 0.107919 3.693602 1.344409
H 0.218258 3.336947 2.235119
H 0.949933 4.443002 -1.065300
H 1.345664 2.820932 -1.614692

1a-c20, ΔG = 0.1010 kcal/mol, population = 5.24 %
C 0.967946 4.086410 1.189222
C 0.317772 3.867335 -0.166617
H -0.554001 4.516454 -0.288181
C -0.171999 2.429583 -0.260419

C 0.888251 1.411788 0.121737
C 1.973986 1.756653 0.939406
N 2.911786 0.866814 1.314156
C 2.778882 -0.372210 0.864267
C 1.746979 -0.792460 0.017465
C 0.758637 0.113474 -0.368246
C -0.375680 -0.233729 -1.294160
H -0.875204 -1.143989 -0.959065
H 0.030395 -0.451953 -2.285893
C -1.364832 0.923733 -1.375476
C -2.094097 1.186101 -0.033049
C -3.542807 1.619796 -0.172248
C -4.487043 0.493918 -0.607887
C -4.623785 -0.643230 0.405659
C -5.655664 -1.691680 -0.006865
C -5.785976 -2.838622 0.994148
C -6.820171 -3.882767 0.577170
H -6.890891 -4.690835 1.308499
H -6.560993 -4.327470 -0.387338
H -7.812325 -3.434301 0.478555
H -4.809566 -3.318978 1.117505
H -6.052261 -2.430405 1.974868
H -5.385153 -2.098808 -0.987986
H -6.631830 -1.209727 -0.133852
H -3.658843 -1.139101 0.549202
H -4.900979 -0.224302 1.379585
H -4.161801 0.084569 -1.570339
H -5.474519 0.928873 -0.786613
H -3.585038 2.445156 -0.888455
H -3.870152 2.017401 0.792390
H -2.010508 0.316173 0.619903
O -1.326434 2.267560 0.555524
H -2.050172 0.808878 -2.210271
O -0.631189 2.149821 -1.574244
C 1.922271 -2.207799 -0.289124
O 1.158245 -2.983348 -0.830833
C 3.322566 -2.634789 0.247926
C 4.267307 -2.672880 -0.930396
C 4.880797 -3.762689 -1.371207
H 4.752240 -4.722022 -0.886056
H 5.544593 -3.721858 -2.225198
H 4.417197 -1.718732 -1.427556
C 3.705158 -1.502541 1.226799
H 3.509433 -1.836194 2.247780

H 4.752065 -1.209384 1.160573
O 3.219756 -3.874794 0.924125
H 2.672348 -4.457077 0.377380
C 2.180447 3.174961 1.393827
H 2.484116 3.165299 2.441637
H 3.035072 3.563804 0.832661
O 1.284736 4.127047 -1.182588
H 0.887235 3.898071 -2.032438
H 1.268176 5.130556 1.281704
H 0.215342 3.895452 1.956187

1a-c37, ΔG = 0.2993 kcal/mol, population = 3.75 %
C 0.848729 -3.144917 1.794269
C 0.925836 -3.281867 0.282881
H 1.909817 -3.647579 -0.024237
C 0.734430 -1.913171 -0.354214
C -0.481342 -1.171844 0.172061
C -1.039193 -1.472929 1.422637
N -2.095468 -0.807075 1.925225
C -2.610659 0.156080 1.175348
C -2.138710 0.497088 -0.097332
C -1.033840 -0.171730 -0.625584
C -0.461370 0.105625 -1.989729
H -0.288077 1.175002 -2.118751
H -1.196903 -0.180287 -2.746898
C 0.831880 -0.676548 -2.196147
C 1.980854 -0.206523 -1.271567
C 3.355306 -0.286451 -1.911710
C 4.514878 -0.004391 -0.954214
C 4.511260 1.402396 -0.355389
C 5.740106 1.686237 0.506457
C 5.746970 3.088967 1.112847
C 6.981358 3.364996 1.969203
H 7.896879 3.266849 1.379790
H 7.046864 2.659275 2.801617
H 6.960974 4.373460 2.388008
H 5.687826 3.828057 0.306759
H 4.843745 3.222958 1.717480
H 6.644777 1.549955 -0.097214
H 5.795614 0.945014 1.312145
H 4.457941 2.140264 -1.164370
H 3.612466 1.547223 0.252339
H 5.452169 -0.156321 -1.498150
H 4.502360 -0.743433 -0.148391

H 3.377013 0.431504 -2.738278
H 3.470582 -1.282604 -2.348694
H 1.781221 0.799770 -0.899397
O 1.905529 -1.129501 -0.153784
H 1.123815 -0.699430 -3.242628
O 0.628417 -2.037622 -1.764301
C -2.948659 1.584043 -0.636108
O -2.761527 2.274572 -1.619368
C -4.167355 1.781215 0.317108
C -5.364694 1.128665 -0.332215
C -6.459528 1.770987 -0.716027
H -6.574923 2.837152 -0.566615
H -7.282472 1.241976 -1.179156
H -5.274604 0.056880 -0.484835
C -3.757777 1.032072 1.604478
H -3.419637 1.759523 2.345048
H -4.569325 0.453600 2.044043
O -4.352837 3.161543 0.575761
H -4.283262 3.626080 -0.270947
C -0.512586 -2.613568 2.248603
H -0.476228 -2.301084 3.293183
H -1.258216 -3.412209 2.194746
O -0.100287 -4.174945 -0.146018
H -0.097378 -4.183842 -1.111730
H 1.042414 -4.113763 2.255494
H 1.645322 -2.469107 2.110249

1a-c3, ΔG = 0.3025 kcal/mol, population = 3.73 %
C 0.636728 4.062363 0.792535
C -0.131943 3.535762 -0.408102
H -1.130445 3.979694 -0.454511
C -0.312325 2.030981 -0.266832
C 0.978868 1.300322 0.052614
C 2.067313 1.958507 0.642241
N 3.210262 1.324851 0.964118
C 3.278621 0.031009 0.687195
C 2.254088 -0.694319 0.068549
C 1.056618 -0.056120 -0.256933
C -0.096493 -0.739803 -0.941502
H -0.351301 -1.665616 -0.423795
H 0.211637 -1.026633 -1.950882
C -1.305430 0.187883 -1.008630
C -1.911951 0.502375 0.381104
C -3.419429 0.673541 0.387035

C -4.172248 -0.652248 0.281230
C -5.688647 -0.468633 0.262481
C -6.458263 -1.785854 0.187401
C -7.974962 -1.601083 0.161486
C -8.738264 -2.922805 0.098254
H -8.508383 -3.549516 0.964013
H -8.471138 -3.487460 -0.799022
H -9.818221 -2.761064 0.081040
H -8.283278 -1.042667 1.051727
H -8.246138 -0.979840 -0.698617
H -6.186194 -2.413118 1.044064
H -6.146979 -2.337556 -0.707209
H -5.995730 0.079111 1.160448
H -5.964214 0.161348 -0.590534
H -3.893641 -1.293397 1.124444
H -3.865935 -1.186564 -0.624542
H -3.699396 1.338972 -0.435608
H -3.698321 1.179474 1.315269
H -1.611505 -0.252853 1.111934
O -1.286010 1.761992 0.736867
H -2.047650 -0.173343 -1.714982
O -0.871073 1.489151 -1.453802
C 2.676055 -2.081121 -0.094339
O 2.024492 -3.054234 -0.421629
C 4.189863 -2.160139 0.273750
C 4.972744 -2.217490 -1.016330
C 5.744996 -3.229775 -1.386920
H 5.879599 -4.101151 -0.758492
H 6.279790 -3.207659 -2.327692
H 4.859414 -1.348746 -1.658482
C 4.459624 -0.838706 1.027210
H 4.482560 -1.040456 2.099899
H 5.402870 -0.369448 0.750685
O 4.418351 -3.274736 1.117821
H 3.952348 -4.030197 0.731005
C 2.031201 3.440269 0.893039
H 2.473878 3.639958 1.869905
H 2.696351 3.900981 0.156897
O 0.608559 3.836216 -1.589657
H 0.158719 3.415478 -2.333524
H 0.717628 5.147464 0.722288
H 0.054617 3.837025 1.687903

1a-c257, ΔG = 0.3194 kcal/mol, population = 3.62 %

C 0.338465 4.109877 0.394668
C -0.332526 3.436675 -0.791164
H -1.356716 3.799824 -0.915465
C -0.412336 1.938024 -0.538057
C 0.911184 1.331598 -0.106924
C 1.919212 2.112405 0.476192
N 3.086581 1.592632 0.898720
C 3.261020 0.290371 0.728873
C 2.324802 -0.554213 0.121706
C 1.102922 -0.034285 -0.307157
C 0.039658 -0.852193 -0.990135
H -0.170319 -1.757143 -0.418204
H 0.416628 -1.182461 -1.962218
C -1.229487 -0.027025 -1.173046
C -1.911328 0.339258 0.169788
C -3.427433 0.363010 0.122204
C -4.035179 -1.038509 0.076258
C -5.561913 -1.026128 0.017800
C -6.164178 -2.430709 -0.022311
C -7.689092 -2.462146 -0.163986
C -8.439086 -1.861872 1.025692
H -8.155574 -2.361246 1.956412
H -9.519072 -1.970500 0.904856
H -8.227489 -0.797413 1.143320
H -7.975215 -1.934371 -1.080262
H -8.006966 -3.500452 -0.297913
H -5.876170 -2.971873 0.886585
H -5.719276 -2.978935 -0.859425
H -5.946144 -0.477778 0.882741
H -5.885835 -0.470015 -0.869353
H -3.710872 -1.600547 0.958820
H -3.649935 -1.583566 -0.791991
H -3.742925 0.945876 -0.748698
H -3.788054 0.891059 1.008925
H -1.568649 -0.326478 0.966302
O -1.413292 1.674451 0.439888
H -1.913798 -0.494020 -1.875719
O -0.873162 1.266999 -1.700728
C 2.854400 -1.913239 0.086456
O 2.293028 -2.955479 -0.191168
C 4.350180 -1.846963 0.523065
C 5.194240 -1.924738 -0.727165
C 6.058622 -2.893956 -0.995178
H 6.229798 -3.708721 -0.302983

H 6.633578 -2.890748 -1.912214
H 5.043809 -1.111207 -1.430984
C 4.481072 -0.460483 1.191742
H 4.456852 -0.585995 2.276034
H 5.402071 0.059394 0.931221
O 4.625410 -2.881900 1.450016
H 4.228047 -3.693698 1.102637
C 1.766902 3.602330 0.603354
H 2.148062 3.910950 1.577753
H 2.431606 4.051291 -0.140475
O 0.440441 3.699378 -1.960881
H 0.058145 3.189179 -2.686347
H 0.345810 5.189349 0.241017
H -0.268322 3.911244 1.279881

1a-c2, ΔG = 0.4418 kcal/mol, population = 2.94 %
C 0.542139 3.331465 -1.052730
C 0.825389 2.938274 0.387773
H 1.871228 3.130064 0.644266
C 0.586090 1.444134 0.557442
C -0.749177 0.983379 0.001848
C -1.428067 1.718053 -0.978879
N -2.593788 1.308478 -1.515295
C -3.094204 0.169331 -1.063172
C -2.502082 -0.606311 -0.059400
C -1.287362 -0.205029 0.494548
C -0.580429 -0.953049 1.593222
H -0.493081 -2.010386 1.338891
H -1.187731 -0.903993 2.501495
C 0.795732 -0.347867 1.852397
C 1.772085 -0.529604 0.664934
C 3.215504 -0.744014 1.076882
C 4.179516 -0.845302 -0.102990
C 5.625724 -1.071072 0.333899
C 6.599341 -1.204261 -0.835501
C 8.046851 -1.425158 -0.398253
C 9.012102 -1.571317 -1.573182
H 10.037489 -1.729311 -1.231947
H 9.003750 -0.676708 -2.201610
H 8.735431 -2.420640 -2.203472
H 8.362565 -0.588293 0.233662
H 8.098617 -2.320160 0.230884
H 6.543974 -0.303699 -1.457983
H 6.284832 -2.037609 -1.474305

H 5.941986 -0.240873 0.975490
H 5.679699 -1.974158 0.952648
H 4.117060 0.067898 -0.701171
H 3.863774 -1.667472 -0.755052
H 3.259932 -1.666690 1.664847
H 3.513652 0.074244 1.739939
H 1.438600 -1.340676 0.012092
O 1.645704 0.721520 -0.061248
H 1.216344 -0.694984 2.792260
O 0.667563 1.086665 1.928639
C -3.339206 -1.775956 0.195949
O -3.099226 -2.776360 0.845037
C -4.682035 -1.558227 -0.560581
C -5.696928 -1.158689 0.488058
C -6.122775 0.068749 0.757119
H -5.781818 0.939794 0.211379
H -6.835943 0.245611 1.552120
H -6.050048 -1.996309 1.083289
C -4.353774 -0.467114 -1.589771
H -4.151413 -0.946153 -2.549700
H -5.153744 0.254730 -1.740857
O -5.081731 -2.760466 -1.212217
H -4.943202 -3.487922 -0.588186
C -0.910119 3.049924 -1.444145
H -1.038791 3.116999 -2.525317
H -1.564014 3.812414 -1.010849
O -0.046291 3.678007 1.240812
H 0.077800 3.352076 2.141418
H 0.766016 4.389945 -1.188732
H 1.224145 2.769811 -1.693556

1a-c36, ΔG = 0.4462 kcal/mol, population = 2.92 %
C 0.753646 3.348146 -0.944421
C 1.062652 2.850321 0.457829
H 2.127590 2.958262 0.682796
C 0.731351 1.367818 0.546763
C -0.653708 1.029965 0.025734
C -1.319740 1.866888 -0.880609
N -2.531115 1.569653 -1.386577
C -3.092336 0.441232 -0.977953
C -2.515125 -0.430681 -0.047533
C -1.251321 -0.147317 0.471565
C -0.550581 -1.010365 1.486678
H -0.545720 -2.051502 1.160877

H -1.113654 -0.983739 2.423868
C 0.873283 -0.513923 1.717775
C 1.783550 -0.681309 0.476750
C 3.226385 -1.015460 0.803716
C 4.131500 -1.064455 -0.424920
C 5.579153 -1.412947 -0.082794
C 6.491091 -1.443135 -1.309422
C 7.935219 -1.859674 -1.012913
C 8.692590 -0.885011 -0.110165
H 8.690206 0.122052 -0.536580
H 9.733324 -1.191833 0.014453
H 8.246734 -0.824273 0.884384
H 7.934334 -2.855212 -0.555658
H 8.473450 -1.959414 -1.960274
H 6.491509 -0.453557 -1.781069
H 6.067101 -2.133705 -2.046107
H 5.955552 -0.686676 0.643734
H 5.611162 -2.389754 0.413868
H 4.100585 -0.096526 -0.932592
H 3.736788 -1.800619 -1.134242
H 3.238326 -1.987129 1.308332
H 3.601497 -0.276800 1.519216
H 1.367194 -1.423285 -0.209758
O 1.713366 0.620500 -0.162582
H 1.309849 -0.947511 2.613408
O 0.845316 0.918145 1.888136
C -3.416162 -1.554930 0.181857
O -3.215947 -2.599272 0.771684
C -4.769402 -1.224797 -0.519799
C -5.737447 -0.764048 0.544056
C -6.871916 -1.378989 0.849378
H -7.192648 -2.271288 0.326519
H -7.522624 -0.995705 1.624777
H -5.439576 0.135232 1.075679
C -4.415868 -0.069412 -1.482470
H -4.303396 -0.471462 -2.491313
H -5.171075 0.714835 -1.511998
O -5.224820 -2.357479 -1.238024
H -5.122696 -3.126440 -0.658442
C -0.728182 3.186931 -1.290783
H -0.892281 3.326215 -2.360250
H -1.312286 3.963706 -0.788571
O 0.273531 3.590463 1.387228
H 0.409395 3.203827 2.261710

H 1.043413 4.395973 -1.028489
H 1.371192 2.782537 -1.644389

1a-c31, ΔG = 0.4537 kcal/mol, population = 2.89 %
C -0.865644 3.300096 0.836775
C -1.294028 2.590717 -0.436942
H -2.382850 2.600953 -0.540243
C -0.860887 1.132858 -0.369700
C 0.595005 0.957972 0.019039
C 1.295627 1.956037 0.710247
N 2.578491 1.810302 1.090787
C 3.174113 0.670902 0.771022
C 2.559718 -0.361649 0.052982
C 1.225287 -0.233533 -0.333975
C 0.480584 -1.274122 -1.126573
H 0.582917 -2.253951 -0.657730
H 0.938146 -1.358160 -2.116272
C -0.992024 -0.896681 -1.262312
C -1.761614 -0.943501 0.079205
C -3.210954 -1.369583 -0.055879
C -3.980182 -1.316378 1.263663
C -5.425767 -1.809289 1.157789
C -6.326174 -0.940200 0.279075
C -7.783514 -1.400558 0.270757
C -8.680112 -0.531303 -0.608999
H -8.337787 -0.540421 -1.647225
H -8.674364 0.507446 -0.268160
H -9.714734 -0.880929 -0.595892
H -7.829502 -2.439473 -0.072969
H -8.166145 -1.400855 1.296888
H -5.952460 -0.932889 -0.749554
H -6.276798 0.097305 0.630232
H -5.432124 -2.837245 0.777594
H -5.854770 -1.852854 2.164198
H -3.972717 -0.290797 1.643137
H -3.448525 -1.924683 2.002884
H -3.224736 -2.393599 -0.444476
H -3.683880 -0.733323 -0.808192
H -1.241707 -1.576733 0.802745
O -1.696027 0.430651 0.545115
H -1.482598 -1.473562 -2.041538
O -1.086796 0.497731 -1.618775
C 3.512115 -1.450751 -0.134238
O 3.324893 -2.580629 -0.542780

C 4.909782 -0.939562 0.332274
C 5.702547 -0.586924 -0.903976
C 6.831543 -1.177911 -1.270842
H 7.278969 -1.965193 -0.677326
H 7.350477 -0.878239 -2.172208
H 5.275292 0.206940 -1.509859
C 4.586115 0.323337 1.161001
H 4.632733 0.072133 2.222448
H 5.273575 1.147953 0.977231
O 5.535123 -1.923336 1.137020
H 5.423126 -2.775495 0.691082
C 0.654535 3.281769 1.012772
H 0.930111 3.580430 2.025187
H 1.113505 4.014621 0.342773
O -0.676557 3.238400 -1.547787
H -0.877897 2.721089 -2.338084
H -1.225613 4.329125 0.816631
H -1.351290 2.801657 1.677598

1a-c8, ΔG = 0.4782 kcal/mol, population = 2.77 %
C 0.020763 3.774355 0.780343
C -0.740882 3.056763 -0.321374
H -1.804826 3.307897 -0.284950
C -0.625707 1.553171 -0.114512
C 0.799542 1.084949 0.108762
C 1.794748 1.957624 0.567622
N 3.058138 1.555973 0.805366
C 3.336308 0.282456 0.572824
C 2.411718 -0.647295 0.082684
C 1.096236 -0.252078 -0.153988
C 0.033007 -1.164941 -0.703927
H -0.008201 -2.091247 -0.129040
H 0.305673 -1.448098 -1.724485
C -1.326479 -0.471758 -0.701274
C -1.873808 -0.199622 0.720660
C -3.384911 -0.276570 0.837126
C -3.910952 -1.712714 0.806274
C -5.426140 -1.816215 1.001259
C -6.251301 -1.187018 -0.121542
C -7.755540 -1.398656 0.047234
C -8.577453 -0.758037 -1.069686
H -9.647114 -0.926174 -0.927060
H -8.301712 -1.169796 -2.044156
H -8.411514 0.321888 -1.108086

H -7.965123 -2.472796 0.087398
H -8.068959 -0.989314 1.013523
H -5.932328 -1.609037 -1.081933
H -6.050923 -0.112701 -0.177392
H -5.696117 -2.873751 1.086028
H -5.699213 -1.352028 1.955598
H -3.410017 -2.288279 1.591092
H -3.641781 -2.188694 -0.142805
H -3.821699 0.318549 0.031517
H -3.676837 0.196834 1.778763
H -1.399715 -0.860578 1.450642
O -1.443273 1.161001 0.984191
H -2.041720 -0.994824 -1.329593
O -1.173230 0.862483 -1.227190
C 3.070709 -1.942209 -0.066605
O 2.589987 -3.039645 -0.277182
C 4.597881 -1.707866 0.120375
C 5.198715 -1.730155 -1.268369
C 5.535875 -0.680463 -2.006560
H 5.426832 0.339344 -1.658710
H 5.933892 -0.810420 -3.004850
H 5.302261 -2.732773 -1.673943
C 4.677753 -0.350763 0.835196
H 4.799022 -0.529476 1.905365
H 5.505234 0.274581 0.506436
O 5.160383 -2.738278 0.926792
H 4.782887 -3.578207 0.626748
C 1.509672 3.420550 0.765710
H 1.993578 3.749043 1.686560
H 2.008853 3.953907 -0.048559
O -0.178743 3.427494 -1.578825
H -0.609252 2.896117 -2.260749
H -0.107100 4.851164 0.666151
H -0.426910 3.493141 1.735204

1a-c23, ΔG = 0.5026 kcal/mol, population = 2.66 %
C 0.191609 -3.781893 0.884568
C 0.719507 -3.205079 -0.418360
H 1.775836 -3.454657 -0.553211
C 0.616408 -1.686913 -0.373921
C -0.753532 -1.191713 0.050609
C -1.628725 -1.999496 0.790004
N -2.836076 -1.571377 1.203397
C -3.183222 -0.337413 0.868479

C -2.386026 0.521669 0.103339
C -1.124460 0.099861 -0.317941
C -0.200727 0.937936 -1.160236
H -0.080740 1.929300 -0.721127
H -0.658494 1.089983 -2.141751
C 1.152848 0.251878 -1.315768
C 1.945631 0.157525 0.011010
C 3.450695 0.273897 -0.141652
C 3.907736 1.710858 -0.398927
C 5.411159 1.842816 -0.657073
C 6.292826 1.448865 0.528110
C 7.781407 1.683499 0.272932
C 8.660745 1.283957 1.456378
H 8.396151 1.854077 2.350868
H 9.717936 1.462023 1.247934
H 8.541142 0.223360 1.692784
H 8.085741 1.120544 -0.615893
H 7.943386 2.740352 0.035932
H 6.138899 0.393827 0.774997
H 5.982972 2.019217 1.411784
H 5.682026 1.234849 -1.527592
H 5.629062 2.880691 -0.928473
H 3.630246 2.334972 0.457600
H 3.368363 2.117157 -1.260211
H 3.773345 -0.379941 -0.958434
H 3.907372 -0.109090 0.772979
H 1.578942 0.893219 0.731459
O 1.614428 -1.168340 0.499260
H 1.735165 0.694552 -2.118876
O 0.937526 -1.136295 -1.642320
C -3.087328 1.786436 -0.087775
O -2.675879 2.840226 -0.533615
C -4.545656 1.598645 0.431750
C -5.433753 1.391420 -0.772654
C -6.413065 2.207462 -1.137849
H -6.656135 3.093952 -0.565814
H -7.011964 2.001942 -2.015713
H -5.211649 0.502762 -1.356572
C -4.472199 0.316410 1.290278
H -4.424557 0.597549 2.344150
H -5.327601 -0.343839 1.153650
O -4.920817 2.713858 1.219919
H -4.641857 3.509969 0.744534
C -1.279943 -3.426246 1.110600

H -1.573927 -3.634494 2.140393
H -1.914472 -4.055480 0.479660
O -0.061602 -3.721723 -1.494186
H 0.223557 -3.278686 -2.303572
H 0.315435 -4.865160 0.876684
H 0.806072 -3.390380 1.697182

1a-c6, ΔG = 0.5729 kcal/mol, population = 2.36 %
C 0.782755 -3.378085 0.641015
C 1.377155 -2.411521 -0.369823
H 2.467847 -2.391733 -0.292078
C 0.876885 -1.004698 -0.074423
C -0.628578 -0.926492 0.097748
C -1.385679 -2.051998 0.450119
N -2.716447 -1.998446 0.645281
C -3.301420 -0.821995 0.475312
C -2.627293 0.341691 0.087059
C -1.245103 0.307132 -0.102047
C -0.429496 1.495189 -0.538333
H -0.653094 2.360799 0.086816
H -0.720762 1.768249 -1.556556
C 1.060905 1.168922 -0.492610
C 1.583268 0.937490 0.947134
C 2.997815 1.424126 1.219088
C 4.065533 0.882021 0.270906
C 5.469678 1.363765 0.632125
C 6.548799 0.822953 -0.303946
C 7.958490 1.291044 0.055324
C 9.028630 0.742900 -0.887006
H 8.840026 1.055308 -1.917563
H 9.042444 -0.350166 -0.870253
H 10.025054 1.093386 -0.609317
H 7.986797 2.385808 0.044747
H 8.185565 0.988852 1.083172
H 6.319963 1.124926 -1.332527
H 6.519443 -0.272765 -0.293461
H 5.494043 2.459414 0.618047
H 5.700503 1.065285 1.661027
H 3.839309 1.178842 -0.757554
H 4.039653 -0.210283 0.288433
H 3.251655 1.153351 2.248481
H 2.983431 2.518527 1.183144
H 0.897348 1.383423 1.671032
O 1.523269 -0.504914 1.091276

H 1.646296 1.902861 -1.038803
O 1.271802 -0.117102 -1.109305
C -3.583985 1.437591 -0.023700
O -3.383594 2.628830 -0.162686
C -5.014339 0.828556 0.105166
C -5.594187 0.726210 -1.285952
C -6.679248 1.365750 -1.700812
H -7.245749 2.010351 -1.040697
H -7.042103 1.248877 -2.713847
H -5.046604 0.076865 -1.962998
C -4.768397 -0.573735 0.704511
H -4.982400 -0.543972 1.774729
H -5.387685 -1.349585 0.256300
O -5.795804 1.621080 0.981027
H -5.656804 2.546987 0.734285
C -0.746157 -3.406758 0.572029
H -1.162457 -3.912211 1.444524
H -1.066833 -3.987081 -0.298118
O 0.972922 -2.814388 -1.677223
H 1.276236 -2.140248 -2.298750
H 1.181941 -4.377158 0.463688
H 1.112099 -3.067791 1.634273

1a-c252, ΔG = 0.5779 kcal/mol, population = 2.34 %
C -0.362333 -4.124529 0.337867
C 0.339405 -3.415585 -0.808653
H 1.367868 -3.772483 -0.914415
C 0.406992 -1.925029 -0.509506
C -0.931676 -1.334411 -0.103275
C -1.952939 -2.134231 0.428793
N -3.134995 -1.630300 0.829033
C -3.310588 -0.324743 0.687822
C -2.360465 0.539226 0.131763
C -1.123720 0.035572 -0.273247
C -0.043449 0.875394 -0.900648
H 0.141847 1.767732 -0.301091
H -0.389189 1.226077 -1.877185
C 1.235236 0.060706 -1.059512
C 1.868044 -0.340524 0.297780
C 3.384849 -0.345114 0.313153
C 3.974467 1.064347 0.349063
C 5.502156 1.072538 0.372412
C 6.086478 2.484372 0.420013
C 7.613341 2.533401 0.533088

C 8.347506 1.959449 -0.679234
H 8.040847 2.469341 -1.596801
H 8.144713 0.894608 -0.808635
H 9.428339 2.078177 -0.577053
H 7.921026 3.573494 0.676537
H 7.922944 1.995639 1.435860
H 5.651001 3.015423 1.272999
H 5.773841 3.034009 -0.475526
H 5.853552 0.507455 1.243311
H 5.873664 0.542420 -0.509351
H 3.595560 1.590226 1.232074
H 3.628673 1.635675 -0.519174
H 3.745354 -0.889520 -0.565087
H 3.714451 -0.903180 1.193622
H 1.485052 0.296186 1.099801
O 1.375636 -1.688520 0.506601
H 1.943429 0.549791 -1.722429
O 0.903335 -1.218861 -1.636339
C -2.894302 1.896934 0.116824
O -2.329493 2.947517 -0.119228
C -4.400931 1.815577 0.512323
C -5.213655 1.935741 -0.755017
C -6.073297 2.912521 -1.010588
H -6.263575 3.702361 -0.294862
H -6.625115 2.940670 -1.941310
H -5.044054 1.147608 -1.483010
C -4.547856 0.408477 1.132804
H -4.560371 0.500234 2.220612
H -5.457671 -0.105590 0.825655
O -4.699681 2.819162 1.466519
H -4.300295 3.643657 1.153134
C -1.797538 -3.626382 0.522611
H -2.201805 -3.960279 1.479248
H -2.442083 -4.058986 -0.248127
O -0.399490 -3.645918 -2.006843
H 0.000629 -3.113269 -2.706068
H -0.362503 -5.199019 0.152441
H 0.219575 -3.950956 1.244791

1a-c19, ΔG = 0.5804 kcal/mol, population = 2.33 %
C -0.118859 3.872946 0.690366
C -0.861628 3.111234 -0.394646
H -1.931944 3.333885 -0.361261
C -0.704212 1.615858 -0.157514

C 0.734666 1.192262 0.070101
C 1.705388 2.101617 0.512462
N 2.979117 1.740995 0.755827
C 3.295099 0.471779 0.544742
C 2.398495 -0.491686 0.068395
C 1.070075 -0.138403 -0.171730
C 0.033512 -1.091836 -0.702906
H 0.024280 -2.010451 -0.114476
H 0.309431 -1.381545 -1.720767
C -1.345939 -0.440241 -0.701155
C -1.887157 -0.158389 0.721868
C -3.392165 -0.293641 0.858744
C -3.855035 -1.751551 0.878743
C -5.366349 -1.914286 1.061680
C -6.202974 -1.389281 -0.105498
C -7.699672 -1.647928 0.065554
C -8.533405 -1.127437 -1.103741
H -8.231493 -1.601678 -2.041351
H -8.408779 -0.048046 -1.224012
H -9.597282 -1.325956 -0.956964
H -7.866771 -2.723588 0.185289
H -8.040291 -1.180094 0.995358
H -5.858590 -1.860335 -1.033740
H -6.042273 -0.313928 -0.230011
H -5.589295 -2.976663 1.203086
H -5.674120 -1.410524 1.984925
H -3.338351 -2.272655 1.690865
H -3.553298 -2.252258 -0.047643
H -3.862101 0.257150 0.040333
H -3.694508 0.196001 1.788649
H -1.378419 -0.784617 1.459223
O -1.507025 1.223087 0.951407
H -2.051189 -0.995803 -1.312640
O -1.236233 0.887655 -1.253506
C 3.090624 -1.769127 -0.063432
O 2.636632 -2.877887 -0.271239
C 4.612569 -1.503886 0.150802
C 5.265138 -1.470688 -1.211179
C 6.190597 -2.324756 -1.626397
H 6.561242 -3.115846 -0.986790
H 6.617129 -2.245880 -2.618061
H 4.914122 -0.678705 -1.866608
C 4.656349 -0.110697 0.816946
H 4.803738 -0.236098 1.891470

H 5.455232 0.524750 0.437102
O 5.154788 -2.491639 1.008622
H 4.818158 -3.349152 0.710374
C 1.379182 3.559212 0.682295
H 1.854447 3.919350 1.595776
H 1.863681 4.089171 -0.143035
O -0.311762 3.471226 -1.660651
H -0.729519 2.915636 -2.331117
H -0.275462 4.943115 0.552276
H -0.558699 3.601335 1.651596

1a-c62, ΔG = 0.7097 kcal/mol, population = 1.87 %
C -0.250417 3.918711 0.502461
C -1.034729 3.075861 -0.489385
H -2.104519 3.293521 -0.422574
C -0.851616 1.602732 -0.152496
C 0.600831 1.206223 0.036818
C 1.581268 2.151365 0.369267
N 2.867983 1.817605 0.580635
C 3.186808 0.538652 0.446619
C 2.279714 -0.462194 0.079705
C 0.938582 -0.135900 -0.125281
C -0.111145 -1.131846 -0.540331
H -0.081364 -2.008754 0.107876
H 0.118604 -1.486970 -1.548909
C -1.495495 -0.492090 -0.514534
C -1.964529 -0.112991 0.912140
C -3.456485 -0.260063 1.143174
C -3.889325 -1.719595 1.285703
C -5.383823 -1.890464 1.579119
C -6.328150 -1.319004 0.515734
C -6.132446 -1.905571 -0.882572
C -7.158207 -1.386201 -1.888360
H -7.101079 -0.298014 -1.977265
H -8.175315 -1.639600 -1.578087
H -6.995995 -1.810688 -2.881456
H -5.126901 -1.672332 -1.244148
H -6.191531 -2.997962 -0.825979
H -6.220236 -0.230671 0.465086
H -7.359319 -1.502764 0.834997
H -5.593158 -2.958171 1.700195
H -5.609867 -1.421323 2.542197
H -3.318181 -2.180815 2.097357
H -3.628452 -2.275830 0.380646

H -3.981322 0.226222 0.316936
H -3.716485 0.288830 2.052658
H -1.404333 -0.676308 1.662789
O -1.597574 1.286633 1.018830
H -2.224567 -1.095083 -1.048139
O -1.427769 0.793038 -1.165654
C 2.977528 -1.741412 0.009099
O 2.525394 -2.864981 -0.099144
C 4.504772 -1.452044 0.137731
C 5.098792 -1.513982 -1.249474
C 6.015363 -2.388946 -1.640111
H 6.421017 -3.127820 -0.960519
H 6.398874 -2.380207 -2.652227
H 4.711585 -0.775285 -1.945420
C 4.563994 -0.014170 0.699786
H 4.758489 -0.061515 1.773081
H 5.339676 0.597730 0.241250
O 5.091887 -2.371608 1.040769
H 4.750419 -3.250502 0.820502
C 1.248721 3.615217 0.446828
H 1.762563 4.045342 1.307603
H 1.688507 4.085425 -0.437641
O -0.547023 3.346597 -1.802248
H -0.989403 2.740971 -2.410733
H -0.424023 4.975136 0.295849
H -0.642433 3.713458 1.500157

1a-c55, ΔG = 0.7229 kcal/mol, population = 1.83 %
C -1.279316 -3.040412 -0.817880
C -1.636342 -2.287603 0.452776
H -2.720837 -2.186381 0.552981
C -1.057547 -0.881518 0.381649
C 0.408328 -0.853663 -0.008465
C 1.004952 -1.917858 -0.698668
N 2.293544 -1.898263 -1.087388
C 2.998273 -0.820335 -0.776359
C 2.491299 0.269151 -0.058400
C 1.152837 0.272268 0.335918
C 0.515160 1.383879 1.125316
H 0.715163 2.347705 0.654849
H 0.978944 1.423752 2.114977
C -0.987447 1.155365 1.264177
C -1.753644 1.271846 -0.074103
C -3.158733 1.824364 0.069522

C -3.941435 1.821540 -1.242172
C -5.363505 2.377681 -1.113598
C -6.266654 1.632806 -0.123675
C -6.431474 0.141552 -0.417755
C -7.410611 -0.541986 0.534913
H -8.404947 -0.092952 0.465588
H -7.076592 -0.446813 1.571647
H -7.508449 -1.606875 0.312982
H -6.772295 0.014291 -1.451096
H -5.460434 -0.357595 -0.353335
H -7.253994 2.106465 -0.131703
H -5.885408 1.755576 0.895367
H -5.309787 3.430958 -0.818862
H -5.833322 2.359140 -2.102434
H -3.973948 0.803206 -1.634548
H -3.395255 2.416747 -1.981258
H -3.083179 2.847539 0.452608
H -3.676389 1.234293 0.830230
H -1.181986 1.853760 -0.801436
O -1.816544 -0.103285 -0.538398
H -1.415905 1.781705 2.041883
O -1.219830 -0.221296 1.627567
C 3.545572 1.262051 0.116860
O 3.471680 2.406890 0.519963
C 4.883949 0.615804 -0.355551
C 5.643031 0.185456 0.877608
C 6.822512 0.667331 1.245118
H 7.340314 1.412634 0.654657
H 7.312821 0.315903 2.143799
H 5.144999 -0.568580 1.480286
C 4.433303 -0.610043 -1.180548
H 4.492510 -0.363552 -2.242507
H 5.040706 -1.496734 -1.003800
O 5.599539 1.533270 -1.162783
H 5.569854 2.392878 -0.718003
C 0.235218 -3.175027 -0.993293
H 0.479092 -3.505410 -2.003898
H 0.618637 -3.946557 -0.319177
O -1.090543 -2.991208 1.567021
H -1.247536 -2.457778 2.356640
H -1.740632 -4.028133 -0.794779
H -1.711986 -2.498696 -1.660847

1a-c39, ΔG = 0.8101 kcal/mol, population = 1.58 %

C 1.121624 3.137920 -0.852772
C 1.656905 2.175337 0.194192
H 2.739843 2.057950 0.096145
C 1.032922 0.804027 -0.020290
C -0.477495 0.845290 -0.161307
C -1.145857 2.012699 -0.555674
N -2.480043 2.061991 -0.726395
C -3.157130 0.947631 -0.491194
C -2.574034 -0.248781 -0.057924
C -1.190289 -0.321166 0.108475
C -0.467570 -1.551744 0.588282
H -0.776133 -2.423965 0.010165
H -0.759284 -1.750347 1.623539
C 1.043327 -1.354834 0.501246
C 1.552663 -1.238103 -0.956571
C 2.915919 -1.854024 -1.226620
C 4.042194 -1.361186 -0.320392
C 5.391220 -1.983312 -0.682463
C 6.533952 -1.589561 0.257146
C 6.879943 -0.099612 0.245672
C 8.083301 0.234238 1.125686
H 8.311508 1.302084 1.103595
H 8.974392 -0.305050 0.793596
H 7.896613 -0.045053 2.166109
H 7.080681 0.211874 -0.785344
H 6.017703 0.484079 0.579246
H 7.428520 -2.159123 -0.016613
H 6.280655 -1.891910 1.280180
H 5.290806 -3.073864 -0.679712
H 5.653986 -1.702940 -1.709000
H 3.813313 -1.593876 0.724278
H 4.099079 -0.273877 -0.387852
H 3.172506 -1.652358 -2.271130
H 2.810394 -2.940504 -1.139755
H 0.817254 -1.659822 -1.645505
O 1.608518 0.195636 -1.171704
H 1.576738 -2.108701 1.073081
O 1.374455 -0.063267 1.049985
C -3.614039 -1.256975 0.113950
O -3.507604 -2.452672 0.308163
C -4.993296 -0.543937 -0.035324
C -5.561491 -0.348546 1.350275
C -6.684885 -0.899012 1.790126
H -7.294163 -1.530401 1.155714

H -7.036485 -0.719510 2.797949
H -4.970011 0.288658 2.001527
C -4.643618 0.812054 -0.687710
H -4.879713 0.764321 -1.752561
H -5.188738 1.651275 -0.257479
O -5.833906 -1.306929 -0.883224
H -5.763803 -2.231208 -0.603478
C -0.396887 3.301303 -0.753233
H -0.790602 3.802298 -1.638661
H -0.644572 3.943945 0.096740
O 1.320183 2.674900 1.487237
H 1.579648 2.008083 2.135910
H 1.608527 4.106591 -0.736197
H 1.397869 2.751107 -1.835269

1a-c1, ΔG = 0.8296 kcal/mol, population = 1.53 %
C 0.813493 3.959925 0.868125
C 0.034575 3.493848 -0.350731
H -0.940416 3.987114 -0.399377
C -0.221195 1.997507 -0.240482
C 1.028291 1.197851 0.078793
C 2.138251 1.788919 0.696535
N 3.243180 1.091551 1.023818
C 3.250980 -0.197134 0.719939
C 2.202436 -0.857283 0.068748
C 1.043852 -0.155153 -0.259067
C -0.132100 -0.766769 -0.972633
H -0.435657 -1.692004 -0.480865
H 0.173090 -1.042122 -1.986076
C -1.294606 0.218822 -1.027174
C -1.893074 0.532739 0.366926
C -3.392800 0.762681 0.375687
C -4.194399 -0.532036 0.250012
C -5.703899 -0.296739 0.254191
C -6.516447 -1.587040 0.167241
C -8.027026 -1.355377 0.169814
C -8.831146 -2.652132 0.096696
H -9.905813 -2.457640 0.101257
H -8.605603 -3.300866 0.947198
H -8.596549 -3.208254 -0.814874
H -8.303549 -0.803923 1.074707
H -8.292985 -0.710922 -0.674687
H -6.250554 -2.238346 1.007710
H -6.236888 -2.131395 -0.742222

H -5.980846 0.244540 1.165774
H -5.969487 0.357020 -0.583910
H -3.929003 -1.200783 1.075882
H -3.917398 -1.056889 -0.670549
H -3.647562 1.451815 -0.435510
H -3.651472 1.263446 1.312613
H -1.624372 -0.247525 1.083884
O -1.218624 1.758970 0.747489
H -2.048814 -0.091341 -1.744971
O -0.793949 1.506059 -1.442844
C 2.566756 -2.258292 -0.124964
O 1.880109 -3.197984 -0.479009
C 4.074562 -2.398187 0.235037
C 4.816594 -2.486596 -1.080531
C 5.488517 -1.514174 -1.683317
H 5.590183 -0.522445 -1.260254
H 5.964002 -1.681340 -2.641403
H 4.722619 -3.456685 -1.560922
C 4.375015 -1.140013 1.062393
H 4.326677 -1.402229 2.121098
H 5.354953 -0.707594 0.871761
O 4.289486 -3.573710 1.010850
H 3.786617 -4.290134 0.596546
C 2.173360 3.265938 0.975058
H 2.611656 3.425145 1.961334
H 2.871152 3.705961 0.256621
O 0.802875 3.779724 -1.518015
H 0.339817 3.399310 -2.275334
H 0.950209 5.040531 0.817758
H 0.209135 3.749721 1.752315

1a-c35, ΔG = 0.8421 kcal/mol, population = 1.50 %
C -0.783647 -3.300100 -1.287015
C -0.980407 -3.068390 0.202239
H -2.012566 -3.280613 0.495273
C -0.712056 -1.604570 0.522505
C 0.599661 -1.097468 -0.047800
C 1.214200 -1.723405 -1.141146
N 2.357960 -1.270018 -1.687267
C 2.903630 -0.196805 -1.134454
C 2.377802 0.465382 -0.018954
C 1.182725 0.019534 0.546932
C 0.542650 0.649883 1.754977
H 0.460207 1.729519 1.621203

H 1.193147 0.496250 2.620543
C -0.828669 0.034684 2.015977
C -1.859169 0.356677 0.905969
C -3.277534 0.543012 1.408974
C -4.294354 0.760337 0.290836
C -5.714707 0.962063 0.820188
C -6.779796 1.097329 -0.270982
C -6.641445 2.347378 -1.141733
C -7.769543 2.482230 -2.163196
H -8.742615 2.538785 -1.668010
H -7.791225 1.621661 -2.837139
H -7.652384 3.380475 -2.773291
H -6.623512 3.231627 -0.495214
H -5.681943 2.331159 -1.665789
H -7.768779 1.107386 0.199409
H -6.755421 0.207926 -0.911609
H -5.972014 0.113805 1.463010
H -5.739343 1.850731 1.461435
H -4.278684 -0.100339 -0.383734
H -3.987415 1.626878 -0.302362
H -3.281787 1.407095 2.081762
H -3.553561 -0.331207 2.007143
H -1.543163 1.229652 0.328682
O -1.790861 -0.809327 0.043295
H -1.196798 0.279804 3.008461
O -0.718936 -1.400881 1.926968
C 3.246124 1.584914 0.329296
O 3.056635 2.497968 1.109674
C 4.541605 1.460777 -0.530557
C 5.621226 0.880275 0.351817
C 6.747807 1.500655 0.674876
H 6.979709 2.489591 0.300044
H 7.481171 1.026797 1.314548
H 5.413873 -0.115766 0.732331
C 4.150898 0.470315 -1.649621
H 3.928743 1.032978 -2.558507
H 4.933047 -0.251664 -1.880665
O 4.879944 2.724657 -1.072627
H 4.802089 3.375146 -0.359656
C 0.648294 -2.986313 -1.727318
H 0.713941 -2.926790 -2.814626
H 1.314529 -3.799995 -1.425989
O -0.071743 -3.904680 0.916059
H -0.143849 -3.684046 1.853496

H -1.028502 -4.335110 -1.527251
H -1.492836 -2.665496 -1.821319

1a-c91, ΔG = 0.8798 kcal/mol, population = 1.40 %
C -0.763714 4.118176 -0.979883
C -0.056239 3.752212 0.314110
H 0.883254 4.303691 0.411754
C 0.290576 2.270483 0.294924
C -0.884586 1.384115 -0.075974
C -1.976155 1.881775 -0.801414
N -3.014436 1.109032 -1.171027
C -2.975914 -0.163983 -0.806052
C -1.944232 -0.732905 -0.050485
C -0.852403 0.050124 0.326269
C 0.295547 -0.459356 1.156002
H 0.687085 -1.386869 0.735645
H -0.072314 -0.705147 2.156116
C 1.394803 0.593646 1.245111
C 2.072559 0.872640 -0.119787
C 3.557166 1.181294 -0.037822
C 4.432710 -0.026139 0.313957
C 4.448402 -1.127703 -0.749225
C 5.376184 -2.299811 -0.414686
C 6.865333 -1.950268 -0.402317
C 7.754632 -3.166610 -0.150298
H 8.812449 -2.894932 -0.147024
H 7.607225 -3.927171 -0.921703
H 7.525563 -3.626143 0.814962
H 7.131294 -1.493902 -1.362058
H 7.065647 -1.194938 0.362366
H 5.211999 -3.097822 -1.146325
H 5.094699 -2.716839 0.559400
H 3.436952 -1.518556 -0.889269
H 4.745243 -0.694159 -1.711095
H 4.112707 -0.455059 1.269883
H 5.448958 0.338996 0.475691
H 3.697632 1.972229 0.704292
H 3.870318 1.590720 -1.002254
H 1.880176 0.053366 -0.814032
O 1.372236 2.048152 -0.603293
H 2.109654 0.361300 2.029178
O 0.795772 1.867568 1.558901
C -2.239561 -2.142530 0.182510
O -1.527034 -3.018810 0.632984

C -3.700617 -2.402509 -0.296622
C -4.582461 -2.436571 0.929031
C -5.272517 -3.493992 1.334142
H -5.260019 -4.425857 0.782958
H -5.885123 -3.451727 2.225501
H -4.616131 -1.508915 1.493175
C -4.026287 -1.176536 -1.178174
H -3.922623 -1.457623 -2.228048
H -5.034217 -0.792032 -1.027945
O -3.749567 -3.597027 -1.055995
H -3.239268 -4.265316 -0.575832
C -2.070260 3.340066 -1.154668
H -2.438200 3.428320 -2.177888
H -2.845850 3.769981 -0.514125
O -0.926405 4.041830 1.406589
H -0.500623 3.724699 2.213323
H -0.964657 5.189796 -0.991248
H -0.082122 3.906272 -1.805516

1a-c14, ΔG = 0.9745 kcal/mol, population = 1.20 %
C -0.741315 -3.255724 -0.926526
C -1.192703 -2.594074 0.364858
H -2.280858 -2.640558 0.465381
C -0.804767 -1.122453 0.337379
C 0.645003 -0.891636 -0.045707
C 1.375877 -1.847445 -0.763214
N 2.653666 -1.650126 -1.141177
C 3.212164 -0.502170 -0.790132
C 2.566288 0.490087 -0.043230
C 1.238699 0.309604 0.340700
C 0.462753 1.303473 1.162920
H 0.536240 2.299567 0.723995
H 0.916115 1.370561 2.155886
C -0.997674 0.877815 1.283778
C -1.765003 0.937429 -0.058782
C -3.224969 1.322969 0.082878
C -3.991365 1.279674 -1.238609
C -5.447269 1.739337 -1.125061
C -6.328695 0.836591 -0.261186
C -7.795799 1.264761 -0.247012
C -8.673474 0.363221 0.619216
H -8.644666 -0.670086 0.263355
H -9.715540 0.690145 0.610274
H -8.332031 0.364813 1.657777

H -7.864600 2.297227 0.112014
H -8.177872 1.271915 -1.273338
H -5.955645 0.821236 0.767610
H -6.256323 -0.193905 -0.628638
H -5.475811 2.760379 -0.727656
H -5.877078 1.790457 -2.130767
H -3.961508 0.262176 -1.638219
H -3.472216 1.913832 -1.964889
H -3.265041 2.337341 0.494345
H -3.682610 0.658460 0.820095
H -1.259762 1.601897 -0.764571
O -1.662169 -0.422260 -0.558444
H -1.507817 1.418089 2.076592
O -1.050278 -0.527802 1.602657
C 3.488912 1.601014 0.173978
O 3.272921 2.717809 0.605341
C 4.899153 1.127825 -0.283232
C 5.671723 0.823144 0.981483
C 5.906908 -0.373945 1.503006
H 5.568084 -1.291889 1.039040
H 6.453280 -0.475529 2.432045
H 6.011586 1.715313 1.500177
C 4.609702 -0.089342 -1.173494
H 4.626478 0.232621 -2.216549
H 5.327952 -0.899248 -1.064215
O 5.545583 2.156233 -1.028065
H 5.389650 2.991039 -0.562462
C 0.777430 -3.185009 -1.100063
H 1.062495 -3.448877 -2.119544
H 1.259096 -3.919904 -0.448462
O -0.557377 -3.252347 1.459291
H -0.774777 -2.762406 2.262675
H -1.068799 -4.295751 -0.933798
H -1.242152 -2.751061 -1.754621

1a-c4, ΔG = 0.9827 kcal/mol, population = 1.18 %
C -0.669492 3.296612 0.821115
C -1.288329 2.383280 -0.223862
H -2.378996 2.385049 -0.143119
C -0.819110 0.955340 0.016141
C 0.684047 0.837441 0.180715
C 1.468082 1.932435 0.565365
N 2.800800 1.844330 0.741074
C 3.357717 0.663858 0.519567

C 2.653450 -0.472204 0.103158
C 1.272052 -0.402514 -0.068127
C 0.427479 -1.555674 -0.541442
H 0.628801 -2.446005 0.055983
H 0.713105 -1.802993 -1.567794
C -1.055095 -1.195610 -0.486462
C -1.578774 -1.007912 0.958870
C -3.009485 -1.462342 1.202135
C -4.052432 -0.851292 0.268298
C -5.474283 -1.302631 0.597669
C -6.525116 -0.693966 -0.329026
C -7.952801 -1.132714 -0.006074
C -8.992950 -0.516064 -0.939679
H -8.974973 0.575397 -0.879624
H -10.003066 -0.847247 -0.689058
H -8.799088 -0.793137 -1.979349
H -8.013882 -2.224867 -0.060006
H -8.185305 -0.863890 1.029830
H -6.291441 -0.962582 -1.365788
H -6.463483 0.399125 -0.275438
H -5.531999 -2.395674 0.541929
H -5.707928 -1.035691 1.634588
H -3.825827 -1.114453 -0.769204
H -3.993050 0.238157 0.328838
H -3.263273 -1.224481 2.239663
H -3.027429 -2.554424 1.123133
H -0.911443 -1.503645 1.667552
O -1.476615 0.424894 1.162393
H -1.655363 -1.893743 -1.062704
O -1.233731 0.118020 -1.052642
C 3.591193 -1.579408 -0.065214
O 3.373710 -2.765372 -0.226397
C 5.026734 -0.984762 0.018889
C 5.529369 -0.902175 -1.406085
C 5.565879 0.177360 -2.176588
H 5.241680 1.152409 -1.834652
H 5.922569 0.117515 -3.196935
H 5.840857 -1.863009 -1.806168
C 4.823601 0.371278 0.710125
H 5.042895 0.254715 1.773235
H 5.460015 1.164798 0.323807
O 5.867234 -1.830279 0.798270
H 5.678108 -2.744024 0.538680
C 0.859210 3.295202 0.745300

H 1.289964 3.755159 1.635918
H 1.188906 3.903951 -0.101691
O -0.878827 2.826968 -1.516260
H -1.199980 2.184809 -2.162200
H -1.047986 4.310147 0.685250
H -1.001334 2.954324 1.802989

1a-c10, ΔG = 1.0034 kcal/mol, population = 1.14 %
C -0.046038 -3.697020 -0.958783
C -0.594015 -3.160431 0.352773
H -1.641323 -3.447861 0.482452
C -0.542563 -1.639230 0.333228
C 0.809270 -1.090664 -0.081635
C 1.712612 -1.856615 -0.829950
N 2.906240 -1.380730 -1.233452
C 3.208713 -0.141362 -0.878849
C 2.379497 0.679728 -0.105798
C 1.133921 0.208834 0.306261
C 0.179786 1.000456 1.159753
H 0.023029 1.992813 0.734633
H 0.632074 1.155018 2.143435
C -1.148255 0.263594 1.306676
C -1.941468 0.160971 -0.018915
C -3.449807 0.210782 0.140186
C -3.968049 1.621611 0.422730
C -5.475360 1.682594 0.685386
C -6.341308 1.270057 -0.505091
C -7.838283 1.432507 -0.242861
C -8.701726 1.016162 -1.432192
H -9.765422 1.140481 -1.217962
H -8.533407 -0.032781 -1.690021
H -8.466820 1.615709 -2.315680
H -8.114677 0.839632 0.635606
H -8.046799 2.476323 0.014690
H -6.140548 0.227674 -0.771351
H -6.060099 1.869636 -1.378984
H -5.716911 1.048201 1.545662
H -5.738718 2.704897 0.974993
H -3.720172 2.271797 -0.423377
H -3.445218 2.036215 1.290205
H -3.741748 -0.470046 0.946378
H -3.892206 -0.176283 -0.779755
H -1.609065 0.924529 -0.726875
O -1.557517 -1.140904 -0.532590

H -1.743452 0.672395 2.118233
O -0.882999 -1.121130 1.610495
C 3.042456 1.963838 0.105872
O 2.598167 3.003594 0.554722
C 4.511745 1.812230 -0.384378
C 5.358057 1.668753 0.861710
C 5.843919 0.543188 1.369126
H 5.689374 -0.422956 0.905048
H 6.418945 0.552091 2.286278
H 5.519111 2.609607 1.380855
C 4.474014 0.568174 -1.284700
H 4.398421 0.894428 -2.323779
H 5.353173 -0.066974 -1.198399
O 4.904979 2.962129 -1.127584
H 4.577884 3.739611 -0.651701
C 1.413251 -3.289209 -1.175021
H 1.715859 -3.469974 -2.207536
H 2.066989 -3.907659 -0.553150
O 0.203488 -3.668096 1.420860
H -0.096867 -3.248137 2.237018
H -0.134490 -4.783732 -0.970259
H -0.671605 -3.311322 -1.765714

1a-c109, ΔG = 1.0329 kcal/mol, population = 1.08 %
C 0.365846 -3.716997 1.405870
C 0.676232 -3.576515 -0.074828
H 1.646238 -4.022644 -0.312108
C 0.759032 -2.099304 -0.430356
C -0.432625 -1.297825 0.061779
C -1.224325 -1.743211 1.129917
N -2.266777 -1.036569 1.604758
C -2.534143 0.115087 1.006725
C -1.818551 0.615764 -0.086708
C -0.724933 -0.096328 -0.580928
C 0.095242 0.345743 -1.762702
H 0.410057 1.383009 -1.639518
H -0.530164 0.318086 -2.659424
C 1.306905 -0.564006 -1.938978
C 2.322975 -0.446167 -0.776193
C 3.772305 -0.576393 -1.205095
C 4.766388 -0.639944 -0.040688
C 4.708410 0.544072 0.930679
C 4.964032 1.906659 0.287426
C 4.986241 3.052450 1.298473

C 5.231646 4.415177 0.653626
H 4.451276 4.650131 -0.075059
H 6.190515 4.433336 0.128802
H 5.243967 5.214146 1.398146
H 4.034411 3.069773 1.840098
H 5.760653 2.855927 2.047478
H 4.197171 2.117287 -0.465072
H 5.918970 1.879190 -0.250618
H 3.738173 0.560702 1.437772
H 5.451986 0.379344 1.716948
H 5.775517 -0.711602 -0.457882
H 4.594841 -1.564358 0.515830
H 4.001705 0.274426 -1.852515
H 3.871357 -1.476517 -1.818320
H 2.162814 0.484464 -0.228251
O 1.964289 -1.552542 0.092018
H 1.771227 -0.431963 -2.912503
O 0.878256 -1.937329 -1.835779
C -2.397135 1.889996 -0.498620
O -1.970210 2.716343 -1.281883
C -3.728495 2.080325 0.291140
C -4.865305 1.738704 -0.642711
C -5.804493 2.589812 -1.032641
H -5.825273 3.613287 -0.680000
H -6.592276 2.280089 -1.707217
H -4.868609 0.713713 -1.002428
C -3.628915 1.053267 1.441090
H -3.338162 1.573071 2.356102
H -4.563375 0.527363 1.632449
O -3.798540 3.400206 0.800508
H -3.540005 3.999925 0.085664
C -0.978999 -3.082666 1.767978
H -1.077644 -2.977611 2.849375
H -1.795191 -3.737729 1.449711
O -0.364048 -4.206596 -0.820185
H -0.204571 -4.028189 -1.755827
H 0.363619 -4.773259 1.676588
H 1.173459 -3.240036 1.963911

1a-c96, ΔG = 1.0366 kcal/mol, population = 1.08 %
C -1.171825 -3.156234 -0.733497
C -1.374179 -2.595854 0.664265
H -2.433835 -2.601261 0.935147
C -0.907461 -1.147192 0.697008

C 0.476889 -0.950670 0.108428
C 1.027396 -1.869610 -0.795645
N 2.239977 -1.699074 -1.354702
C 2.915453 -0.614183 -1.005238
C 2.454401 0.335760 -0.086415
C 1.193335 0.181322 0.490787
C 0.615974 1.131342 1.505401
H 0.681010 2.158836 1.144334
H 1.221603 1.086741 2.414915
C -0.831620 0.765904 1.822206
C -1.790026 0.982928 0.626969
C -3.184834 1.425453 1.024768
C -4.145065 1.551683 -0.155638
C -5.551705 1.960888 0.278801
C -6.517435 2.219047 -0.882463
C -6.741807 1.031370 -1.826320
C -7.298780 -0.213779 -1.136767
H -6.595775 -0.620064 -0.406646
H -7.510847 -1.002436 -1.861828
H -8.228977 0.013963 -0.608811
H -7.432910 1.344438 -2.614617
H -5.805170 0.778660 -2.331709
H -6.146705 3.067479 -1.467627
H -7.484235 2.528904 -0.471329
H -5.960352 1.189529 0.939121
H -5.486369 2.870795 0.884465
H -4.180525 0.599225 -0.688675
H -3.752287 2.290477 -0.863753
H -3.097729 2.392039 1.531781
H -3.579364 0.713323 1.756591
H -1.358461 1.683153 -0.092947
O -1.848350 -0.329818 0.008164
H -1.179412 1.256447 2.727220
O -0.920054 -0.658779 2.029436
C 3.458062 1.383020 0.067948
O 3.373156 2.460125 0.625854
C 4.748346 0.911826 -0.670760
C 5.715926 0.412341 0.375784
C 6.903198 0.948497 0.623539
H 7.272629 1.795637 0.059403
H 7.548941 0.542724 1.391603
H 5.368633 -0.442921 0.948384
C 4.260320 -0.244224 -1.571864
H 4.150140 0.125059 -2.593336

H 4.941496 -1.094003 -1.590537
O 5.266851 1.973829 -1.451849
H 5.252327 2.770719 -0.901998
C 0.302314 -3.138938 -1.145467
H 0.406137 -3.316274 -2.216831
H 0.835270 -3.954700 -0.648370
O -0.615160 -3.379977 1.582852
H -0.678984 -2.959954 2.450163
H -1.557421 -4.175338 -0.773495
H -1.766270 -2.555881 -1.424452

1a-c24, ΔG = 1.0580 kcal/mol, population = 1.04 %
C -0.615783 -3.131625 -1.796635
C -0.703009 -3.283509 -0.287107
H -1.664018 -3.716606 0.004722
C -0.612021 -1.909504 0.361425
C 0.555483 -1.082845 -0.145743
C 1.145873 -1.335030 -1.390822
N 2.157643 -0.591269 -1.878105
C 2.594208 0.399560 -1.116326
C 2.086619 0.697205 0.153981
C 1.028443 -0.051998 0.665926
C 0.426943 0.170439 2.027686
H 0.181734 1.224199 2.168059
H 1.172910 -0.076533 2.788346
C -0.813612 -0.697837 2.212081
C -1.978593 -0.295508 1.275174
C -3.353946 -0.461682 1.895753
C -4.514773 -0.238774 0.923931
C -4.579509 1.169579 0.332021
C -5.817841 1.397632 -0.532809
C -5.884340 2.797983 -1.141162
C -7.131530 3.022165 -1.994071
H -7.152578 4.029444 -2.415698
H -8.040429 2.889473 -1.401199
H -7.171865 2.312088 -2.824392
H -5.853109 3.540176 -0.336332
H -4.989147 2.967936 -1.748693
H -6.716908 1.222905 0.069281
H -5.839341 0.653656 -1.337588
H -4.565542 1.905104 1.144805
H -3.687008 1.362066 -0.271727
H -5.449308 -0.444590 1.454741
H -4.450962 -0.971737 0.115008

H -3.428858 0.247083 2.727088
H -3.416644 -1.466475 2.323489
H -1.836473 0.723105 0.910069
O -1.832100 -1.206163 0.154226
H -1.116474 -0.749309 3.254437
O -0.513776 -2.038275 1.771987
C 2.819048 1.834393 0.705830
O 2.575581 2.515468 1.683891
C 4.042269 2.090734 -0.221632
C 5.245198 1.528309 0.503741
C 5.838326 0.363442 0.276772
H 5.512681 -0.317498 -0.499878
H 6.681399 0.044829 0.876442
H 5.592768 2.164211 1.313268
C 3.676401 1.364834 -1.524303
H 3.272669 2.096595 -2.226756
H 4.516266 0.869099 -2.006674
O 4.203657 3.487430 -0.451016
H 4.081081 3.936132 0.398407
C 0.711283 -2.503994 -2.230088
H 0.666159 -2.187921 -3.273235
H 1.509896 -3.249309 -2.171435
O 0.375150 -4.108649 0.150450
H 0.363928 -4.118613 1.116114
H -0.736557 -4.108526 -2.265785
H -1.452793 -2.509916 -2.119235

1a-c25, ΔG = 1.0831 kcal/mol, population = 1.00 %
C -0.699669 4.160652 -0.605274
C 0.143773 3.596713 0.526089
H 1.126380 4.076237 0.552928
C 0.372190 2.110525 0.289037
C -0.905102 1.348891 -0.014929
C -2.042515 1.996120 -0.518047
N -3.174031 1.336892 -0.829155
C -3.182382 0.027791 -0.625339
C -2.106459 -0.689958 -0.090625
C -0.919899 -0.024705 0.219849
C 0.286260 -0.699906 0.815499
H 0.548340 -1.588461 0.239393
H 0.035528 -1.048377 1.821416
C 1.463613 0.267482 0.876674
C 1.986160 0.679819 -0.522455
C 3.486034 0.894977 -0.599893

C 4.275502 -0.413374 -0.592480
C 5.785928 -0.187004 -0.668626
C 6.610765 -1.474484 -0.742217
C 6.522335 -2.357321 0.503802
C 7.428299 -3.584817 0.421602
H 7.350679 -4.201054 1.319923
H 8.475351 -3.292598 0.305756
H 7.162867 -4.209676 -0.435372
H 6.789929 -1.761161 1.383057
H 5.489849 -2.683059 0.656891
H 7.660376 -1.211201 -0.910259
H 6.297976 -2.055183 -1.617967
H 6.004417 0.426503 -1.548544
H 6.106099 0.398348 0.200809
H 3.960495 -1.031536 -1.440526
H 4.034300 -0.983215 0.308802
H 3.792677 1.530820 0.236587
H 3.701513 1.448818 -1.517636
H 1.666925 -0.042004 -1.278711
O 1.307051 1.937246 -0.770878
H 2.252268 -0.102938 1.525287
O 1.005932 1.524632 1.415746
C -2.471555 -2.098429 0.013200
O -1.772709 -3.063279 0.256780
C -3.996807 -2.213524 -0.291713
C -4.717113 -2.356914 1.028043
C -5.426861 -3.416875 1.390311
H -5.552813 -4.268194 0.733242
H -5.917770 -3.455896 2.354178
H -4.611530 -1.510435 1.700553
C -4.343027 -0.868249 -0.967415
H -4.396759 -1.018137 -2.047535
H -5.292253 -0.449473 -0.635751
O -4.226557 -3.295516 -1.176347
H -3.709840 -4.047752 -0.852664
C -2.073326 3.490378 -0.680100
H -2.569506 3.729407 -1.621754
H -2.719045 3.879946 0.112394
O -0.549663 3.800182 1.755888
H -0.049609 3.355361 2.452158
H -0.817360 5.235782 -0.466866
H -0.152563 4.010904 -1.537714

1a-c12, ΔG = 1.0875 kcal/mol, population = 0.99 %

C -0.553376 -3.932721 0.881891
C 0.176543 -3.428827 -0.352270
H 1.178868 -3.862207 -0.414308
C 0.342368 -1.918852 -0.255739
C -0.947851 -1.194439 0.082027
C -2.009617 -1.848033 0.721226
N -3.148727 -1.216958 1.065174
C -3.239015 0.067628 0.757492
C -2.243676 0.786381 0.085297
C -1.051499 0.153243 -0.261989
C 0.070864 0.829979 -1.002819
H 0.328599 1.774715 -0.522049
H -0.270792 1.080198 -2.011163
C 1.289153 -0.084539 -1.075618
C 1.933077 -0.349758 0.308301
C 3.443901 -0.485078 0.285509
C 4.156060 0.854568 0.099521
C 5.677152 0.717522 0.060948
C 6.390242 2.057101 -0.125785
C 7.913131 1.950776 -0.250732
C 8.608582 1.434897 1.009570
H 9.694251 1.444353 0.891194
H 8.313261 0.410842 1.245789
H 8.361721 2.058952 1.873053
H 8.158578 1.299106 -1.096331
H 8.314029 2.937925 -0.499434
H 6.143250 2.716914 0.714146
H 5.993847 2.542516 -1.023798
H 6.013996 0.238991 0.985101
H 5.958340 0.043225 -0.756047
H 3.872914 1.530046 0.914033
H 3.817241 1.332252 -0.825978
H 3.721505 -1.179525 -0.513571
H 3.758717 -0.940276 1.228307
H 1.631612 0.418840 1.024740
O 1.342292 -1.611947 0.710956
H 2.008649 0.264572 -1.810832
O 0.860120 -1.402938 -1.472418
C -2.693409 2.163053 -0.103223
O -2.069429 3.141028 -0.469335
C -4.199843 2.215098 0.284216
C -4.969198 2.258393 -1.017827
C -5.591587 1.246872 -1.609298
H -5.624965 0.251051 -1.184944

H -6.093594 1.384408 -2.558520
H -4.942622 3.232562 -1.498287
C -4.410915 0.943131 1.117962
H -4.359964 1.210017 2.175397
H -5.366546 0.452457 0.945369
O -4.468451 3.378046 1.062415
H -4.008678 4.119587 0.642149
C -1.951513 -3.323183 1.004517
H -2.366814 -3.505604 1.996623
H -2.629666 -3.807450 0.295819
O -0.591369 -3.768694 -1.505383
H -0.167176 -3.361149 -2.271364
H -0.623570 -5.020103 0.841380
H 0.050004 -3.678004 1.755016

1a-c58, ΔG = 1.0906 kcal/mol, population = 0.98 %
C -0.869598 4.201436 -0.808401
C -0.074468 3.789497 0.418803
H 0.849813 4.369275 0.495273
C 0.321071 2.325406 0.292566
C -0.843423 1.418563 -0.059550
C -1.994371 1.915350 -0.687071
N -3.028960 1.127095 -1.033699
C -2.926263 -0.161342 -0.742832
C -1.829364 -0.733263 -0.087888
C -0.740903 0.066419 0.262351
C 0.474680 -0.445929 0.987137
H 0.873314 -1.329375 0.486080
H 0.178835 -0.768575 1.989314
C 1.538857 0.643103 1.080365
C 2.132741 1.029637 -0.296976
C 3.603031 1.409552 -0.268054
C 4.549577 0.232666 -0.014171
C 4.546532 -0.830521 -1.116385
C 5.628228 -1.899902 -0.944250
C 5.452631 -2.781180 0.293942
C 6.498052 -3.891526 0.381254
H 6.353760 -4.508142 1.271015
H 7.508134 -3.475405 0.424281
H 6.447731 -4.547292 -0.492033
H 5.503655 -2.167338 1.197443
H 4.449322 -3.220894 0.278051
H 6.611492 -1.416623 -0.906789
H 5.635537 -2.541194 -1.831870

H 3.569990 -1.323300 -1.162349
H 4.685222 -0.333964 -2.082371
H 4.312540 -0.228431 0.948485
H 5.564217 0.629713 0.086918
H 3.740216 2.172091 0.503763
H 3.848010 1.877161 -1.225772
H 1.946331 0.241279 -1.027704
O 1.353797 2.194015 -0.678145
H 2.301995 0.393629 1.811674
O 0.913028 1.872592 1.500655
C -2.065685 -2.162111 0.087888
O -1.298033 -3.036396 0.441248
C -3.548647 -2.444310 -0.303343
C -4.342706 -2.558761 0.976566
C -4.975393 -3.652991 1.377555
H -4.976844 -4.558364 0.783765
H -5.526493 -3.668305 2.309021
H -4.362008 -1.658726 1.584455
C -3.966407 -1.189334 -1.101331
H -3.918678 -1.416446 -2.168173
H -4.974497 -0.846661 -0.871892
O -3.618906 -3.604396 -1.112331
H -3.057341 -4.277371 -0.700808
C -2.157417 3.386298 -0.952820
H -2.586494 3.515495 -1.947531
H -2.908969 3.752647 -0.247386
O -0.887961 3.983394 1.574232
H -0.406979 3.632075 2.334433
H -1.107153 5.263728 -0.745347
H -0.231795 4.062568 -1.683059

1a-c115, ΔG = 1.1000 kcal/mol, population = 0.97 %
C -0.805839 -3.273847 -1.671866
C -0.934615 -3.324091 -0.158687
H -1.923749 -3.686967 0.134990
C -0.785070 -1.917812 0.403219
C 0.439620 -1.190217 -0.121260
C 1.046257 -1.555516 -1.331387
N 2.112713 -0.905775 -1.833395
C 2.589535 0.105456 -1.122413
C 2.067643 0.512747 0.110625
C 0.951443 -0.138749 0.636672
C 0.326404 0.208846 1.961268
H 0.137501 1.281615 2.022535

H 1.036400 -0.024740 2.759609
C -0.965272 -0.576639 2.164133
C -2.082193 -0.176007 1.169511
C -3.480291 -0.227203 1.759807
C -4.601502 -0.049169 0.733854
C -4.580398 1.292344 0.000487
C -5.773379 1.465622 -0.940192
C -5.737476 2.747943 -1.777057
C -5.819637 4.032951 -0.952227
H -5.863092 4.912804 -1.597781
H -4.954782 4.149140 -0.296358
H -6.714862 4.035097 -0.324062
H -4.821683 2.757704 -2.378128
H -6.569162 2.726742 -2.487654
H -6.700238 1.442775 -0.355173
H -5.815181 0.603594 -1.614356
H -4.562621 2.102169 0.736363
H -3.658204 1.384792 -0.582513
H -5.559779 -0.157080 1.251206
H -4.550202 -0.862486 0.005022
H -3.546860 0.557264 2.520819
H -3.599973 -1.185179 2.274182
H -1.874925 0.806590 0.742029
O -1.958503 -1.166224 0.115767
H -1.298142 -0.542171 3.197971
O -0.729417 -1.957904 1.821136
C 2.846417 1.638106 0.615248
O 2.616888 2.380508 1.550544
C 4.097029 1.795804 -0.303488
C 5.276680 1.195027 0.423591
C 6.352002 1.869868 0.806795
H 6.463756 2.926779 0.599902
H 7.162677 1.376911 1.327764
H 5.190578 0.132932 0.634879
C 3.742068 0.970044 -1.559908
H 3.423174 1.650412 -2.352017
H 4.575070 0.378044 -1.936762
O 4.278027 3.161453 -0.633227
H 4.172464 3.671831 0.182819
C 0.563297 -2.748445 -2.108958
H 0.558961 -2.495344 -3.170111
H 1.317059 -3.531506 -1.985073
O 0.087698 -4.174498 0.357231
H 0.051806 -4.125860 1.321099

H -0.969724 -4.270693 -2.082165
H -1.599898 -2.630215 -2.054459

1a-c113, ΔG = 1.1057 kcal/mol, population = 0.96 %
C 0.843540 -3.311059 0.818197
C 1.227333 -2.643736 -0.491902
H 2.310291 -2.676085 -0.641721
C 0.822205 -1.177016 -0.445704
C -0.611694 -0.965581 0.001611
C -1.298477 -1.932481 0.748697
N -2.561394 -1.754146 1.178913
C -3.151415 -0.613481 0.853302
C -2.550933 0.389193 0.083075
C -1.236165 0.227798 -0.355305
C -0.508112 1.234830 -1.204469
H -0.570550 2.226732 -0.754448
H -1.008597 1.306261 -2.174172
C 0.949478 0.825446 -1.398063
C 1.783517 0.892009 -0.096804
C 3.234086 1.278022 -0.315847
C 4.067816 1.242330 0.964412
C 5.521712 1.684416 0.771687
C 6.350562 0.740933 -0.102691
C 7.807690 1.176858 -0.289732
C 8.637021 1.163596 0.994996
H 9.680571 1.413241 0.791328
H 8.615433 0.174626 1.461300
H 8.264728 1.882856 1.726827
H 8.278899 0.515659 -1.023135
H 7.827397 2.181039 -0.727143
H 5.886062 0.655154 -1.088648
H 6.328370 -0.263847 0.335884
H 5.540900 2.689705 0.334406
H 5.988003 1.769413 1.756478
H 4.048156 0.230469 1.378782
H 3.593123 1.891219 1.707839
H 3.252293 2.289968 -0.734408
H 3.652025 0.609247 -1.072351
H 1.314783 1.557842 0.632360
O 1.709293 -0.466392 0.412059
H 1.412763 1.372003 -2.214942
O 1.002632 -0.579421 -1.720439
C -3.493572 1.488104 -0.096387
O -3.306103 2.603000 -0.544104

C -4.878995 1.011547 0.438332
C -5.723143 0.633936 -0.755845
C -6.858776 1.227922 -1.096451
H -7.274783 2.037428 -0.509969
H -7.415342 0.908317 -1.968047
H -5.328939 -0.182358 -1.354446
C -4.540490 -0.231642 1.290776
H -4.538889 0.051657 2.345166
H -5.247557 -1.049848 1.160538
O -5.460004 2.026988 1.236675
H -5.355141 2.863876 0.761119
C -0.666784 -3.260645 1.060391
H -0.901665 -3.525718 2.092258
H -1.167505 -4.003825 0.433078
O 0.551384 -3.309758 -1.556962
H 0.729223 -2.819132 -2.369613
H 1.184966 -4.346551 0.810621
H 1.374263 -2.799207 1.622940

1a-c30, ΔG = 1.1157 kcal/mol, population = 0.94 %
C 1.124525 4.253290 0.528551
C 0.167431 3.793334 -0.558267
H -0.735186 4.411035 -0.567581
C -0.267218 2.363160 -0.272171
C 0.895148 1.428519 0.009021
C 2.132225 1.912477 0.458003
N 3.165704 1.101288 0.749996
C 2.975297 -0.199183 0.582287
C 1.788071 -0.762406 0.100949
C 0.701914 0.063068 -0.191765
C -0.608325 -0.440978 -0.734521
H -0.971959 -1.275137 -0.132919
H -0.446202 -0.836014 -1.741303
C -1.635003 0.686435 -0.773468
C -2.028251 1.189530 0.642131
C -3.480224 1.606293 0.817432
C -4.428004 0.452921 1.162204
C -4.554784 -0.637687 0.098641
C -5.615714 -1.683476 0.438457
C -5.732395 -2.786420 -0.612230
C -6.798119 -3.826740 -0.272362
H -6.858093 -4.603510 -1.037715
H -7.784992 -3.363854 -0.188615
H -6.579586 -4.314235 0.681467

H -5.958622 -2.333702 -1.583431
H -4.760927 -3.279558 -0.723337
H -6.586819 -1.189021 0.554370
H -5.382528 -2.133260 1.410354
H -4.798292 -0.179558 -0.866619
H -3.596025 -1.149036 -0.034116
H -5.419314 0.875275 1.351010
H -4.105563 -0.001818 2.105221
H -3.815083 2.123117 -0.087459
H -3.509027 2.337842 1.628114
H -1.756356 0.448031 1.398492
O -1.179542 2.353723 0.819397
H -2.494794 0.429618 -1.382285
O -1.022178 1.852461 -1.359557
C 1.937693 -2.211540 0.027682
O 1.095834 -3.067671 -0.164590
C 3.438930 -2.543832 0.288707
C 4.083752 -2.822578 -1.048542
C 4.623298 -3.981925 -1.399448
H 4.651273 -4.823860 -0.719387
H 5.068712 -4.116599 -2.376700
H 4.074817 -1.987547 -1.743252
C 4.002844 -1.248570 0.914255
H 4.072080 -1.378402 1.996101
H 4.991078 -0.983734 0.540607
O 3.538559 -3.627402 1.195364
H 2.902516 -4.300136 0.911639
C 2.386701 3.389243 0.579964
H 2.942998 3.572787 1.500238
H 3.056602 3.662633 -0.240487
O 0.837976 3.862622 -1.815305
H 0.252618 3.480929 -2.481969
H 1.394272 5.295454 0.355054
H 0.594856 4.209034 1.481832

1a-c75, ΔG = 1.1264 kcal/mol, population = 0.93 %
C -0.196834 -3.801631 -0.827869
C -0.787961 -3.164567 0.418734
H -1.853141 -3.397339 0.505607
C -0.663831 -1.650875 0.315671
C 0.733439 -1.188137 -0.052500
C 1.637451 -2.035271 -0.708444
N 2.870270 -1.637003 -1.073664
C 3.214132 -0.393768 -0.771430

C 2.388026 0.504431 -0.085879
C 1.099807 0.114178 0.281640
C 0.140782 0.997370 1.033629
H 0.057913 1.969768 0.546063
H 0.544546 1.187706 2.032002
C -1.227983 0.332408 1.141617
C -1.946791 0.187643 -0.222582
C -3.456368 0.324964 -0.158225
C -3.911042 1.776755 0.002939
C -5.421022 1.927872 0.205053
C -6.260555 1.458664 -0.985425
C -7.754401 1.771509 -0.854905
C -8.442593 1.042888 0.300027
H -9.515469 1.246631 0.310599
H -8.040121 1.348304 1.267700
H -8.308848 -0.038936 0.211993
H -7.885278 2.852406 -0.734444
H -8.252411 1.505165 -1.791964
H -6.136984 0.380233 -1.127381
H -5.874942 1.934313 -1.893843
H -5.712240 1.381481 1.107163
H -5.645364 2.982215 0.398348
H -3.601425 2.350106 -0.877703
H -3.397864 2.227760 0.857957
H -3.829809 -0.284347 0.671225
H -3.868028 -0.099518 -1.075890
H -1.531462 0.887219 -0.952501
O -1.605465 -1.161381 -0.633594
H -1.848363 0.815326 1.891329
O -1.048228 -1.042412 1.539134
C 3.096085 1.767173 0.094325
O 2.676095 2.843740 0.472734
C 4.578607 1.539179 -0.331869
C 5.391047 1.357213 0.928707
C 6.362031 2.168343 1.325647
H 6.654923 3.032305 0.742523
H 6.904804 1.980387 2.243076
H 5.119714 0.491370 1.526017
C 4.531434 0.229337 -1.149754
H 4.539323 0.475488 -2.213432
H 5.370155 -0.435861 -0.949027
O 5.019573 2.621009 -1.131915
H 4.720079 3.436629 -0.704417
C 1.289315 -3.471155 -0.986157

H 1.637823 -3.727659 -1.987636
H 1.879372 -4.078336 -0.293449
O -0.073174 -3.642736 1.556691
H -0.397011 -3.163118 2.329841
H -0.334726 -4.882242 -0.781765
H -0.760442 -3.438240 -1.688980

1a-c84, ΔG = 1.1678 kcal/mol, population = 0.86 %
C -0.540714 -4.091959 1.239330
C 0.034983 -3.844389 -0.145005
H 0.953175 -4.420228 -0.291766
C 0.396869 -2.372554 -0.281952
C -0.728874 -1.439503 0.126878
C -1.744689 -1.856771 0.998749
N -2.733237 -1.038912 1.405361
C -2.722739 0.198217 0.931472
C -1.770457 0.684687 0.028642
C -0.728806 -0.144787 -0.388984
C 0.332301 0.274938 -1.370579
H 0.764219 1.232780 -1.077494
H -0.131723 0.431978 -2.348385
C 1.413841 -0.795259 -1.469865
C 2.212609 -0.965968 -0.152117
C 3.686304 -1.281825 -0.337274
C 4.517029 -0.104588 -0.857856
C 4.575190 1.099827 0.083527
C 5.520082 2.192047 -0.418868
C 5.531093 3.459752 0.440506
C 6.046895 3.242350 1.863456
H 6.098540 4.185394 2.411776
H 7.050341 2.807594 1.851081
H 5.401043 2.568008 2.429114
H 6.152035 4.213179 -0.053271
H 4.518268 3.875229 0.478649
H 5.234404 2.459686 -1.441692
H 6.537261 1.788106 -0.480032
H 3.577060 1.532023 0.208052
H 4.888211 0.759435 1.075038
H 4.139270 0.216944 -1.834217
H 5.535171 -0.462777 -1.035973
H 3.769693 -2.130841 -1.021584
H 4.082968 -1.609971 0.627574
H 2.078596 -0.092036 0.487018
O 1.562815 -2.096379 0.485186

H 2.054631 -0.639903 -2.332939
O 0.780766 -2.082766 -1.617624
C -2.078168 2.074278 -0.292389
O -1.409490 2.898523 -0.885780
C -3.480482 2.396543 0.309016
C -4.484377 2.335866 -0.817898
C -5.207847 3.363048 -1.242069
H -5.134472 4.339470 -0.780018
H -5.909270 3.250929 -2.058786
H -4.580837 1.362991 -1.291355
C -3.719858 1.256060 1.323244
H -3.502073 1.624395 2.327714
H -4.740936 0.877048 1.314722
O -3.444393 3.653328 0.960697
H -2.976829 4.267768 0.376316
C -1.815165 -3.279247 1.480133
H -2.077533 -3.277140 2.539023
H -2.655959 -3.746222 0.958922
O -0.945611 -4.203759 -1.116618
H -0.603090 -3.957599 -1.985398
H -0.749721 -5.155403 1.358907
H 0.223153 -3.826295 1.972160

1a-c18, ΔG = 1.1772 kcal/mol, population = 0.85 %
C -1.929108 -2.514563 -0.292752
C -2.044721 -1.647996 0.949699
H -3.085219 -1.362086 1.126900
C -1.253557 -0.364844 0.738598
C 0.154932 -0.600537 0.230460
C 0.506927 -1.782237 -0.435466
N 1.737918 -1.991056 -0.938332
C 2.627267 -1.023740 -0.768514
C 2.367984 0.172555 -0.088690
C 1.091871 0.411827 0.422954
C 0.710369 1.656522 1.179383
H 1.009920 2.545468 0.622485
H 1.267594 1.681676 2.120142
C -0.790506 1.682217 1.463279
C -1.654535 1.845566 0.189625
C -2.938016 2.625888 0.419240
C -3.797453 2.862381 -0.828444
C -4.353176 1.615168 -1.526773
C -5.213033 0.719689 -0.636905
C -5.699796 -0.541172 -1.349423

C -6.521694 -1.458365 -0.445891
H -5.935317 -1.781223 0.418658
H -6.853053 -2.353606 -0.976640
H -7.410485 -0.945199 -0.069185
H -6.292722 -0.259927 -2.226369
H -4.831246 -1.089146 -1.730004
H -6.075266 1.287093 -0.266627
H -4.636957 0.420061 0.241720
H -4.949822 1.943160 -2.384909
H -3.529623 1.022630 -1.930112
H -3.208370 3.437303 -1.550875
H -4.633313 3.507051 -0.537685
H -2.651210 3.601934 0.822654
H -3.512211 2.121400 1.200252
H -1.072155 2.296942 -0.617868
O -1.952193 0.471949 -0.174868
H -1.038376 2.411392 2.229439
O -1.197894 0.383915 1.942381
C 3.574619 0.992593 -0.082310
O 3.718173 2.156499 0.239295
C 4.744113 0.112216 -0.620121
C 5.539442 -0.365488 0.572037
C 6.813459 -0.073800 0.795848
H 7.386291 0.532964 0.105993
H 7.325768 -0.449149 1.672312
H 4.985322 -0.983909 1.272329
C 4.029961 -1.068812 -1.313705
H 4.020195 -0.893147 -2.391259
H 4.507873 -2.030312 -1.130959
O 5.515612 0.852693 -1.548345
H 5.661935 1.730523 -1.166635
C -0.477679 -2.909692 -0.576741
H -0.381138 -3.334205 -1.576912
H -0.165153 -3.693472 0.119508
O -1.524997 -2.374139 2.062075
H -1.521659 -1.780897 2.824122
H -2.541693 -3.408003 -0.168978
H -2.341257 -1.951494 -1.131909

1a-c33, ΔG = 1.1898 kcal/mol, population = 0.83 %
C -1.508794 -2.897069 -0.623568
C -1.526090 -2.455555 0.830406
H -2.550100 -2.418403 1.212675
C -0.954739 -1.048430 0.933675

C 0.373445 -0.886464 0.216396
C 0.759040 -1.751168 -0.817445
N 1.914809 -1.605883 -1.491835
C 2.700790 -0.603769 -1.127335
C 2.411202 0.281206 -0.082249
C 1.208581 0.155579 0.614419
C 0.810839 1.042191 1.763778
H 0.922522 2.092430 1.490458
H 1.495093 0.867041 2.598748
C -0.623143 0.747939 2.194260
C -1.671444 1.135214 1.120084
C -2.964903 1.681441 1.699447
C -4.001362 2.152562 0.675620
C -4.694206 1.048561 -0.125075
C -5.817692 1.581304 -1.012214
C -6.512926 0.493275 -1.829989
C -7.644630 1.031229 -2.703663
H -8.415533 1.510458 -2.094405
H -8.121407 0.234036 -3.278196
H -7.272367 1.776386 -3.411833
H -5.771910 -0.010992 -2.459320
H -6.906357 -0.269936 -1.150088
H -5.413833 2.339699 -1.693386
H -6.560600 2.094451 -0.390373
H -3.962857 0.520110 -0.739440
H -5.102315 0.305151 0.569139
H -3.525114 2.858748 -0.014304
H -4.765699 2.723815 1.212311
H -2.689404 2.533294 2.328900
H -3.397576 0.924349 2.361170
H -1.246681 1.850337 0.410285
O -1.901402 -0.117288 0.423459
H -0.845833 1.178568 3.166455
O -0.795216 -0.680349 2.294723
C 3.501209 1.241256 0.051326
O 3.557037 2.261786 0.710206
C 4.666512 0.763332 -0.868677
C 5.702255 0.102027 0.009118
C 6.947307 0.531712 0.163177
H 7.316520 1.403809 -0.361725
H 7.640635 0.010532 0.810642
H 5.356221 -0.780839 0.538974
C 3.999974 -0.265310 -1.808957
H 3.806296 0.209357 -2.772873

H 4.610624 -1.150163 -1.983588
O 5.174760 1.861854 -1.604414
H 5.282490 2.598697 -0.985529
C -0.087290 -2.936874 -1.188753
H -0.105905 -3.026312 -2.275788
H 0.433329 -3.824866 -0.818351
O -0.736318 -3.366826 1.592311
H -0.681516 -3.025106 2.493938
H -1.970396 -3.881346 -0.707742
H -2.125083 -2.199201 -1.193101

1a-c98, ΔG = 1.2067 kcal/mol, population = 0.81 %
C 1.232461 -3.005401 1.490836
C 1.281426 -3.010966 -0.028113
H 2.292589 -3.229686 -0.382952
C 0.910697 -1.628833 -0.547736
C -0.364841 -1.079978 0.064578
C -0.846404 -1.543920 1.296517
N -1.956564 -1.048257 1.874540
C -2.601546 -0.095488 1.217905
C -2.211417 0.398358 -0.032466
C -1.053353 -0.092139 -0.636663
C -0.561485 0.355613 -1.986573
H -0.515577 1.444714 -2.032592
H -1.284589 0.046000 -2.746412
C 0.805316 -0.252111 -2.287620
C 1.923433 0.276487 -1.356722
C 3.276048 0.402717 -2.034660
C 4.425871 0.745157 -1.085259
C 4.273018 2.096887 -0.383978
C 5.501111 2.517079 0.427719
C 5.807905 1.613716 1.623940
C 6.987001 2.116271 2.455125
H 7.896223 2.171821 1.850516
H 7.189824 1.457169 3.302029
H 6.790873 3.116906 2.849430
H 4.915610 1.544268 2.255758
H 6.018020 0.597980 1.278005
H 5.351915 3.539410 0.791127
H 6.376461 2.551156 -0.231440
H 4.065207 2.863899 -1.137760
H 3.402879 2.075436 0.280123
H 5.357272 0.748593 -1.660381
H 4.522157 -0.052355 -0.345887

H 3.188907 1.177389 -2.803806
H 3.487947 -0.539120 -2.549072
H 1.623988 1.222061 -0.901432
O 1.989358 -0.730383 -0.312885
H 1.062039 -0.160643 -3.339521
O 0.773542 -1.656665 -1.960477
C -3.160781 1.418618 -0.464154
O -3.086480 2.203769 -1.389707
C -4.366716 1.386315 0.523863
C -5.487696 0.630437 -0.150142
C -6.669048 1.148571 -0.457312
H -6.917095 2.175377 -0.220121
H -7.430521 0.551804 -0.942695
H -5.262374 -0.405097 -0.388601
C -3.827926 0.603273 1.741553
H -3.551493 1.312550 2.524252
H -4.549614 -0.097864 2.158583
O -4.722550 2.707237 0.890110
H -4.735415 3.237783 0.080191
C -0.167997 -2.677109 2.014070
H -0.138224 -2.447882 3.080191
H -0.817203 -3.551190 1.907993
O 0.356810 -3.984602 -0.509347
H 0.331920 -3.915245 -1.472290
H 1.551316 -3.978317 1.866103
H 1.953380 -2.267240 1.846746

1a-c286, ΔG = 1.2080 kcal/mol, population = 0.81 %
C -0.195159 -4.195575 0.259145
C 0.539345 -3.416120 -0.818896
H 1.577414 -3.750775 -0.900606
C 0.568448 -1.942286 -0.440304
C -0.794051 -1.395070 -0.055425
C -1.821550 -2.237472 0.392029
N -3.025439 -1.772943 0.774474
C -3.216564 -0.464229 0.697657
C -2.260742 0.442305 0.224966
C -1.001219 -0.020667 -0.157727
C 0.089198 0.867183 -0.695340
H 0.239123 1.725897 -0.039413
H -0.226249 1.269263 -1.662280
C 1.386066 0.080797 -0.854602
C 1.983434 -0.385177 0.497350
C 3.499408 -0.389256 0.550117

C 4.091859 1.014018 0.673613
C 5.619781 1.004161 0.691515
C 6.251911 2.384003 0.904648
C 5.894643 3.439246 -0.149153
C 6.280915 3.047582 -1.575159
H 5.726245 2.171336 -1.917240
H 7.346396 2.810451 -1.640957
H 6.075631 3.860272 -2.275121
H 4.823148 3.655778 -0.109042
H 6.398377 4.373553 0.115918
H 7.340689 2.268442 0.931117
H 5.958493 2.757908 1.891300
H 5.960333 0.337309 1.489982
H 5.985418 0.566759 -0.242582
H 3.719918 1.485881 1.589793
H 3.739946 1.635502 -0.154535
H 3.880628 -0.885887 -0.347636
H 3.808104 -0.995513 1.405988
H 1.576914 0.208619 1.320240
O 1.492634 -1.744256 0.624306
H 2.105871 0.614901 -1.468237
O 1.094198 -1.170766 -1.509309
C -2.817098 1.790487 0.260515
O -2.262138 2.860822 0.102523
C -4.336058 1.665111 0.592124
C -5.101934 1.834330 -0.698564
C -5.964782 2.811362 -0.942143
H -6.193570 3.565295 -0.199409
H -6.480663 2.875643 -1.891508
H -4.893743 1.083464 -1.455402
C -4.480183 0.226551 1.136185
H -4.529152 0.263729 2.226248
H -5.370894 -0.285201 0.774101
O -4.689304 2.614856 1.581836
H -4.290180 3.459463 1.327175
C -1.645226 -3.730230 0.409394
H -2.084109 -4.123234 1.327509
H -2.248413 -4.128272 -0.411856
O -0.146362 -3.594786 -2.056896
H 0.274003 -3.021784 -2.710841
H -0.168529 -5.258662 0.018227
H 0.346231 -4.060902 1.197275

1a-c89, ΔG = 1.2080 kcal/mol, population = 0.81 %

C 0.379612 -4.029366 -1.007657
C -0.295242 -3.587694 0.280649
H -1.293803 -4.025714 0.366035
C -0.465539 -2.075123 0.265062
C 0.808432 -1.332138 -0.094210
C 1.842231 -1.952395 -0.809799
N 2.965101 -1.304041 -1.171113
C 3.070084 -0.035001 -0.805648
C 2.105206 0.648292 -0.056459
C 0.926985 -0.003441 0.309298
C -0.162490 0.635307 1.128588
H -0.441705 1.600388 0.703403
H 0.223162 0.840740 2.131171
C -1.375797 -0.284981 1.213357
C -2.082965 -0.486728 -0.150150
C -3.590495 -0.632464 -0.062373
C -4.299775 0.692306 0.217161
C -5.817901 0.536485 0.302610
C -6.560130 1.829198 0.657081
C -6.374238 2.984651 -0.333778
C -6.835774 2.662402 -1.754899
H -6.756386 3.538314 -2.402262
H -7.878962 2.334375 -1.762165
H -6.236901 1.867340 -2.203688
H -6.932605 3.849059 0.037527
H -5.324442 3.291562 -0.352953
H -6.237698 2.160428 1.649987
H -7.629243 1.606507 0.739877
H -6.191725 0.139228 -0.646086
H -6.052962 -0.218954 1.059165
H -4.040227 1.405872 -0.570248
H -3.933224 1.124300 1.154633
H -3.827526 -1.365766 0.714705
H -3.947201 -1.044881 -1.010080
H -1.818431 0.317033 -0.842057
O -1.507081 -1.724589 -0.640660
H -2.060404 0.026743 1.996959
O -0.928368 -1.619704 1.526918
C 2.560090 2.013970 0.181639
O 1.949645 2.967170 0.625793
C 4.046830 2.100505 -0.280051
C 4.912619 2.011209 0.954859
C 5.737630 2.963136 1.368860
H 5.856767 3.888746 0.820018

H 6.331552 2.835441 2.264680
H 4.814894 1.085692 1.514935
C 4.230742 0.851878 -1.170844
H 4.159797 1.151099 -2.218520
H 5.187700 0.352855 -1.024279
O 4.251899 3.287796 -1.023614
H 3.816734 4.007525 -0.543695
C 1.771398 -3.412431 -1.160117
H 2.145065 -3.547120 -2.176101
H 2.478129 -3.928526 -0.503706
O 0.523512 -3.981341 1.380343
H 0.130258 -3.618446 2.184304
H 0.451057 -5.117286 -1.023526
H -0.260866 -3.733047 -1.840227

1a-c69, ΔG = 1.2268 kcal/mol, population = 0.78 %
C -0.158050 3.865089 0.635869
C -0.834351 3.116009 -0.500224
H -1.904480 3.340159 -0.530195
C -0.693530 1.618079 -0.269415
C 0.729271 1.190170 0.037419
C 1.673478 2.093726 0.544391
N 2.932600 1.730480 0.851759
C 3.261162 0.464462 0.641094
C 2.392389 -0.493245 0.105334
C 1.078631 -0.137441 -0.202037
C 0.073883 -1.084767 -0.800831
H 0.029496 -2.008129 -0.221441
H 0.408090 -1.366450 -1.803346
C -1.302688 -0.431123 -0.872452
C -1.924568 -0.164296 0.520884
C -3.434277 -0.305557 0.572289
C -3.891357 -1.765250 0.544827
C -5.409104 -1.935665 0.653738
C -6.186270 -1.375710 -0.539819
C -7.681776 -1.707528 -0.520601
C -8.443904 -1.072875 0.643326
H -8.313947 0.013012 0.646451
H -8.098030 -1.450314 1.607438
H -9.513776 -1.280696 0.573455
H -8.127268 -1.373825 -1.462519
H -7.807415 -2.795237 -0.490184
H -5.745679 -1.773538 -1.460533
H -6.067569 -0.288631 -0.587080

H -5.636117 -3.002802 0.748782
H -5.752886 -1.464449 1.579325
H -3.411444 -2.299295 1.371022
H -3.544128 -2.247212 -0.375480
H -3.860787 0.255378 -0.262851
H -3.789434 0.169175 1.491262
H -1.455904 -0.796833 1.279114
O -1.561779 1.215488 0.784969
H -1.971576 -0.979017 -1.529963
O -1.160105 0.902350 -1.403004
C 3.092675 -1.768177 -0.007069
O 2.652018 -2.874869 -0.251346
C 4.601010 -1.503207 0.286584
C 5.319679 -1.445770 -1.041138
C 6.269137 -2.288881 -1.423268
H 6.611908 -3.088398 -0.778656
H 6.743800 -2.192148 -2.391217
H 4.997087 -0.645399 -1.701049
C 4.607246 -0.120784 0.976196
H 4.697426 -0.263314 2.054907
H 5.424087 0.521323 0.649433
O 5.103691 -2.503640 1.153506
H 4.781613 -3.356498 0.827105
C 1.337527 3.549065 0.715401
H 1.755631 3.894962 1.661828
H 1.872550 4.090675 -0.070117
O -0.207310 3.487469 -1.726426
H -0.579845 2.935430 -2.425861
H -0.304833 4.936895 0.499667
H -0.655802 3.584478 1.565745

1a-c156, ΔG = 1.2550 kcal/mol, population = 0.75 %
C 0.694695 3.579450 -1.530794
C 0.952841 3.514383 -0.034848
H 1.934582 3.930895 0.208208
C 0.957148 2.059138 0.409472
C -0.254936 1.281858 -0.071482
C -0.987349 1.693100 -1.194317
N -2.039315 0.999712 -1.667478
C -2.375140 -0.103190 -1.014383
C -1.724003 -0.563031 0.135691
C -0.622530 0.135943 0.631373
C 0.133849 -0.262222 1.871015
H 0.391265 -1.321737 1.835900

H -0.516020 -0.131234 2.740908
C 1.388574 0.591508 2.019384
C 2.412684 0.344778 0.882711
C 3.862769 0.383940 1.324593
C 4.867089 0.280350 0.170598
C 4.685335 -0.941505 -0.736481
C 4.787820 -2.283750 -0.009980
C 4.675493 -3.501346 -0.932389
C 3.301255 -3.654529 -1.586111
H 3.070729 -2.816130 -2.245997
H 2.514696 -3.705211 -0.827963
H 3.248254 -4.567308 -2.183284
H 5.445674 -3.438905 -1.708890
H 4.896578 -4.403085 -0.353641
H 4.008605 -2.354950 0.756089
H 5.743802 -2.321850 0.523339
H 3.724730 -0.869068 -1.253026
H 5.448704 -0.907278 -1.520666
H 5.875434 0.267960 0.595566
H 4.797610 1.185693 -0.437047
H 4.013495 -0.436772 2.031153
H 4.032960 1.312095 1.877356
H 2.189341 -0.597821 0.379273
O 2.149430 1.429427 -0.044308
H 1.829372 0.495495 3.007872
O 1.033209 1.975831 1.824568
C -2.364646 -1.791198 0.593137
O -1.998750 -2.586924 1.436677
C -3.668660 -1.982011 -0.241362
C -4.831024 -1.552794 0.622105
C -5.808815 -2.352998 1.024729
H -5.845177 -3.395737 0.735560
H -6.613351 -1.980962 1.645913
H -4.819530 -0.507655 0.917837
C -3.486296 -1.025444 -1.440594
H -3.179739 -1.605745 -2.313054
H -4.392362 -0.478768 -1.698666
O -3.762334 -3.325972 -0.679258
H -3.549533 -3.891417 0.077423
C -0.664413 2.980962 -1.899797
H -0.734868 2.816736 -2.975944
H -1.460706 3.686908 -1.646367
O -0.079898 4.233377 0.636823
H 0.046803 4.111878 1.586465

H 0.745997 4.617099 -1.861769
H 1.497766 3.037591 -2.033310

1a-c50, ΔG = 1.3109 kcal/mol, population = 0.68 %
C 0.018607 3.782934 1.534543
C -0.385534 3.666667 0.074288
H -1.329084 4.188212 -0.110238
C -0.605037 2.200753 -0.270840
C 0.540866 1.303742 0.159556
C 1.416953 1.680066 1.186233
N 2.412646 0.882779 1.618427
C 2.547306 -0.288857 1.016139
C 1.742923 -0.725039 -0.042977
C 0.697773 0.080603 -0.492092
C -0.211793 -0.284754 -1.635427
H -0.597375 -1.297058 -1.506352
H 0.369224 -0.292524 -2.561973
C -1.356255 0.716871 -1.743257
C -2.309082 0.673685 -0.522026
C -3.771567 0.923701 -0.842700
C -4.445408 -0.208566 -1.627802
C -4.383741 -1.594482 -0.975145
C -4.979842 -1.659496 0.430505
C -4.982776 -3.071477 1.014543
C -5.569842 -3.134254 2.423365
H -5.558946 -4.153101 2.816526
H -6.605427 -2.784172 2.432273
H -5.001704 -2.504254 3.112852
H -5.548822 -3.733761 0.351105
H -3.956945 -3.455083 1.027628
H -6.005941 -1.274204 0.406587
H -4.422921 -1.000516 1.104302
H -4.917823 -2.299343 -1.619975
H -3.348601 -1.949954 -0.942953
H -4.006233 -0.270548 -2.627819
H -5.493346 0.066837 -1.777735
H -3.842681 1.855899 -1.409752
H -4.295576 1.090235 0.101018
H -2.183975 -0.263538 0.023066
O -1.821128 1.751875 0.317866
H -1.880287 0.626158 -2.690230
O -0.815833 2.051824 -1.666651
C 2.188522 -2.049668 -0.467247
O 1.654499 -2.846176 -1.215800

C 3.540360 -2.335298 0.249622
C 4.620193 -2.131936 -0.790568
C 5.396474 -1.065497 -0.933793
H 5.339417 -0.207665 -0.275299
H 6.123476 -1.012999 -1.734166
H 4.697263 -2.956145 -1.494408
C 3.563557 -1.328920 1.409567
H 3.237039 -1.838929 2.317953
H 4.544566 -0.900211 1.603604
O 3.564380 -3.672093 0.741806
H 3.205618 -4.242694 0.046474
C 1.323421 3.038116 1.824652
H 1.476008 2.930209 2.899381
H 2.171933 3.620044 1.452939
O 0.657629 4.212740 -0.730984
H 0.432175 4.047965 -1.655478
H 0.124997 4.835916 1.797006
H -0.793431 3.377371 2.140600

1a-c22, ΔG = 1.3335 kcal/mol, population = 0.65 %
C 0.602414 3.308250 -1.014495
C 0.932465 2.849645 0.396439
H 1.992677 3.003373 0.617083
C 0.658959 1.356912 0.515142
C -0.711482 0.955197 0.000357
C -1.409559 1.747650 -0.920163
N -2.608939 1.392804 -1.420348
C -3.123730 0.251491 -0.990432
C -2.512325 -0.580122 -0.044711
C -1.262901 -0.236875 0.469263
C -0.530930 -1.049752 1.503687
H -0.485941 -2.097230 1.201868
H -1.095465 -1.022645 2.440013
C 0.872507 -0.493961 1.724167
C 1.788183 -0.651474 0.485808
C 3.241574 -0.928968 0.818157
C 4.147709 -0.978292 -0.409728
C 5.601860 -1.291992 -0.061383
C 6.515394 -1.324671 -1.286791
C 7.963855 -1.721205 -0.984180
C 8.706957 -0.731587 -0.085923
H 8.692170 0.272909 -0.518071
H 9.751351 -1.024191 0.042209
H 8.258523 -0.671092 0.907481

H 7.973863 -2.713592 -0.520215
H 8.505569 -1.820742 -1.929576
H 6.504407 -0.340490 -1.769467
H 6.100519 -2.028470 -2.016134
H 5.963506 -0.546991 0.653570
H 5.652189 -2.259858 0.451052
H 4.096941 -0.019808 -0.933570
H 3.768908 -1.734348 -1.106707
H 3.285554 -1.887786 1.345112
H 3.592498 -0.162086 1.515995
H 1.398474 -1.420997 -0.185873
O 1.670788 0.634288 -0.177989
H 1.325373 -0.892124 2.628081
O 0.788886 0.939113 1.865540
C -3.373906 -1.733288 0.203801
O -3.136864 -2.766852 0.799906
C -4.742874 -1.442386 -0.476583
C -5.688145 -1.041246 0.634927
C -6.051884 0.191561 0.964171
H -5.704804 1.067078 0.429512
H -6.717110 0.368154 1.799738
H -6.043500 -1.884705 1.220434
C -4.424371 -0.327425 -1.483322
H -4.277982 -0.779424 -2.466250
H -5.206805 0.423019 -1.574794
O -5.219772 -2.606386 -1.144771
H -5.063892 -3.360714 -0.557690
C -0.872091 3.082133 -1.355712
H -1.041935 3.194559 -2.427444
H -1.486058 3.844778 -0.867665
O 0.116445 3.576374 1.313200
H 0.266466 3.210271 2.194196
H 0.850898 4.364883 -1.118529
H 1.241301 2.754402 -1.704700

1a-c9, ΔG = 1.3435 kcal/mol, population = 0.64 %
C -1.274778 -3.929351 1.211786
C -0.566053 -3.754670 -0.120952
H 0.270123 -4.453926 -0.210784
C 0.010594 -2.348407 -0.202460
C -0.994447 -1.266724 0.147895
C -2.123012 -1.542399 0.930825
N -3.010275 -0.592527 1.283979
C -2.781127 0.635122 0.844498

C -1.699391 0.989435 0.029587
C -0.763504 0.022111 -0.332387
C 0.418370 0.292814 -1.224810
H 0.969872 1.165628 -0.872229
H 0.055229 0.541889 -2.225934
C 1.329825 -0.928461 -1.288046
C 2.014918 -1.242523 0.064899
C 3.419589 -1.807826 -0.051828
C 4.465970 -0.798174 -0.533288
C 4.695824 0.386099 0.407932
C 5.839507 1.289341 -0.048810
C 6.079283 2.483908 0.874175
C 7.229067 3.375997 0.410319
H 7.377984 4.220507 1.086583
H 7.035305 3.777622 -0.587805
H 8.166255 2.815040 0.365076
H 5.160367 3.075885 0.941597
H 6.283739 2.119738 1.886612
H 5.629919 1.652985 -1.061433
H 6.759610 0.698144 -0.120492
H 3.784266 0.986112 0.490151
H 4.908666 0.010674 1.415228
H 4.194063 -0.422186 -1.525330
H 5.411708 -1.331521 -0.666146
H 3.384514 -2.662313 -0.733414
H 3.708865 -2.194474 0.929296
H 1.999956 -0.364903 0.712844
O 1.148563 -2.251196 0.647956
H 2.036272 -0.857642 -2.109949
O 0.522062 -2.103602 -1.503705
C -1.778228 2.416058 -0.273709
O -0.950419 3.150765 -0.778562
C -3.174148 2.912906 0.201740
C -4.022673 3.038929 -1.045127
C -4.921707 2.168599 -1.486268
H -5.157846 1.252686 -0.958650
H -5.456679 2.345308 -2.410632
H -3.802519 3.930805 -1.625287
C -3.635410 1.828027 1.186168
H -3.412474 2.163275 2.200935
H -4.699617 1.607086 1.134153
O -3.053532 4.172443 0.855399
H -2.434291 4.707849 0.337761
C -2.435860 -2.945358 1.371178

H -2.780362 -2.918452 2.405914
H -3.289085 -3.280651 0.774290
O -1.510908 -3.962322 -1.169244
H -1.071376 -3.759973 -2.005055
H -1.640271 -4.953252 1.295352
H -0.539111 -3.780844 2.004276

1a-c87, ΔG = 1.3611 kcal/mol, population = 0.62 %
C 0.510814 -4.160628 -1.183743
C -0.128759 -3.878649 0.165450
H -1.057020 -4.445548 0.280258
C -0.487502 -2.402285 0.252065
C 0.661097 -1.483780 -0.125493
C 1.714828 -1.926122 -0.938019
N 2.726159 -1.122507 -1.316108
C 2.699551 0.125864 -0.873363
C 1.707667 0.638104 -0.029322
C 0.643817 -0.177119 0.359117
C -0.458566 0.269952 1.281370
H -0.871314 1.222820 0.947264
H -0.038562 0.447394 2.275414
C -1.549182 -0.792931 1.354904
C -2.284920 -0.988820 0.004075
C -3.769247 -1.286472 0.121479
C -4.611770 -0.095960 0.591641
C -4.585931 1.112714 -0.345238
C -5.538852 2.223007 0.098564
C -5.550879 3.446368 -0.822876
C -4.227485 4.212058 -0.858441
H -4.310358 5.111927 -1.471586
H -3.418516 3.606790 -1.272038
H -3.929828 4.519859 0.147825
H -5.817931 3.129710 -1.836903
H -6.345026 4.123910 -0.495417
H -5.272387 2.540698 1.113530
H -6.552158 1.812901 0.161704
H -3.570959 1.511914 -0.406777
H -4.852257 0.791323 -1.358802
H -4.290706 0.216452 1.590847
H -5.644905 -0.437392 0.703780
H -3.895433 -2.127729 0.808734
H -4.122477 -1.619583 -0.858464
H -2.110167 -0.131869 -0.648049
O -1.615690 -2.139225 -0.574093

H -2.228566 -0.614924 2.183312
O -0.930793 -2.079473 1.561512
C 2.005859 2.034460 0.270548
O 1.312323 2.876695 0.807414
C 3.438117 2.335249 -0.267636
C 4.383364 2.296010 0.909945
C 5.087172 3.330450 1.349241
H 5.039988 4.297278 0.864164
H 5.745800 3.234110 2.202763
H 4.452637 1.332937 1.407582
C 3.720768 1.169451 -1.241025
H 3.555392 1.513996 -2.263768
H 4.737991 0.786171 -1.172687
O 3.442758 3.576368 -0.949442
H 2.947319 4.205990 -0.405581
C 1.800214 -3.359971 -1.381829
H 2.113391 -3.384372 -2.426528
H 2.612181 -3.818300 -0.809716
O 0.801149 -4.221809 1.191138
H 0.417523 -3.956161 2.036686
H 0.718806 -5.227536 -1.269169
H -0.215689 -3.907940 -1.957959

1a-c121, ΔG = 1.3956 kcal/mol, population = 0.59 %
C -0.868105 3.247953 1.420436
C -0.996244 3.152606 -0.090860
H -2.006053 3.423065 -0.412473
C -0.754032 1.714016 -0.524819
C 0.514524 1.118860 0.057145
C 1.092353 1.629278 1.227814
N 2.197877 1.097322 1.781735
C 2.741496 0.060334 1.161587
C 2.250592 -0.488046 -0.028952
C 1.095207 0.039952 -0.606217
C 0.496387 -0.463941 -1.892419
H 0.379724 -1.548159 -1.858405
H 1.190635 -0.254839 -2.711137
C -0.844370 0.212590 -2.159072
C -1.933428 -0.170710 -1.127500
C -3.331414 -0.261006 -1.713412
C -4.441544 -0.439153 -0.674752
C -4.358760 -1.748074 0.113587
C -5.460449 -1.911588 1.164914
C -6.874168 -2.041712 0.595276

C -7.924641 -2.279451 1.679028
H -7.939319 -1.454247 2.395960
H -7.713739 -3.196716 2.235227
H -8.926457 -2.369372 1.253501
H -7.134797 -1.140371 0.033911
H -6.893970 -2.868060 -0.123656
H -5.427439 -1.060829 1.855552
H -5.244257 -2.800832 1.766471
H -4.394333 -2.593409 -0.583526
H -3.390616 -1.808250 0.618447
H -5.400529 -0.383649 -1.194803
H -4.417513 0.404091 0.021337
H -3.343885 -1.101090 -2.415742
H -3.514556 0.646826 -2.295496
H -1.661814 -1.095913 -0.616322
O -1.875563 0.915130 -0.165671
H -1.172387 0.063996 -3.184327
O -0.700389 1.631171 -1.941287
C 3.104692 -1.598577 -0.435647
O 2.928271 -2.433862 -1.301521
C 4.360828 -1.590023 0.489168
C 5.498429 -0.974766 -0.291113
C 6.619614 -1.604032 -0.615869
H 6.803674 -2.628299 -0.317028
H 7.396111 -1.103437 -1.179834
H 5.339831 0.056178 -0.594434
C 3.946812 -0.684516 1.670292
H 3.670013 -1.313477 2.518713
H 4.737114 -0.008155 1.993173
O 4.634670 -2.906026 0.935609
H 4.567137 -3.491616 0.167434
C 0.530355 2.850853 1.899576
H 0.537491 2.689913 2.978573
H 1.233262 3.667037 1.708873
O -0.029768 4.017212 -0.685107
H -0.058265 3.877502 -1.640282
H -1.095412 4.265617 1.739159
H -1.621044 2.592772 1.862132

1a-c15, ΔG = 1.3981 kcal/mol, population = 0.59 %
C -0.929124 -3.224621 -0.739594
C -1.100595 -2.737868 0.689392
H -2.141339 -2.837078 1.010955
C -0.745603 -1.260050 0.761871

C 0.587798 -0.931032 0.116848
C 1.158555 -1.765846 -0.852789
N 2.324536 -1.477620 -1.462626
C 2.932782 -0.359965 -1.096303
C 2.450252 0.510861 -0.112429
C 1.237828 0.235950 0.517567
C 0.646038 1.092830 1.604457
H 0.622182 2.138503 1.294341
H 1.295667 1.047075 2.483021
C -0.754652 0.607426 1.964446
C -1.776689 0.800456 0.817122
C -3.178204 1.144035 1.282843
C -4.185158 1.257276 0.140668
C -5.594776 1.589879 0.631057
C -6.610646 1.825428 -0.489352
C -6.890341 0.600822 -1.362565
C -7.966966 0.860885 -2.414863
H -7.671066 1.673327 -3.083979
H -8.912877 1.146143 -1.946842
H -8.150783 -0.024624 -3.027155
H -5.970778 0.281700 -1.860618
H -7.197034 -0.232520 -0.720948
H -6.262073 2.647833 -1.125190
H -7.554064 2.161713 -0.046201
H -5.950749 0.779992 1.278211
H -5.547338 2.485184 1.259870
H -4.193585 0.320422 -0.419811
H -3.852040 2.035042 -0.556305
H -3.127835 2.094698 1.823852
H -3.502468 0.382906 1.999616
H -1.418666 1.547965 0.104165
O -1.781605 -0.493072 0.157465
H -1.098204 1.033298 2.903288
O -0.726902 -0.826722 2.113712
C 3.385153 1.622082 0.042689
O 3.253155 2.670418 0.645628
C 4.670727 1.260728 -0.756695
C 5.699327 0.839003 0.269707
C 6.045751 -0.401836 0.586971
H 5.618232 -1.271057 0.102783
H 6.778811 -0.591587 1.360625
H 6.138474 1.676876 0.804204
C 4.212632 0.146604 -1.709226
H 4.004660 0.589560 -2.685144

H 4.946889 -0.643459 -1.852842
O 5.129704 2.393410 -1.489038
H 5.067517 3.161165 -0.902029
C 0.515514 -3.074130 -1.222593
H 0.575812 -3.199472 -2.304654
H 1.135143 -3.864488 -0.788990
O -0.237375 -3.495506 1.535170
H -0.285693 -3.114027 2.421091
H -1.236378 -4.268752 -0.805673
H -1.603726 -2.646568 -1.373656

1a-c47, ΔG = 1.3993 kcal/mol, population = 0.58 %
C -0.796709 3.374406 0.634755
C -1.353913 2.442216 -0.428166
H -2.447058 2.430877 -0.398772
C -0.879167 1.022872 -0.150664
C 0.616241 0.925347 0.086358
C 1.366965 2.033960 0.500595
N 2.688167 1.963341 0.748542
C 3.270561 0.786893 0.570111
C 2.603619 -0.360167 0.124231
C 1.230774 -0.308194 -0.120500
C 0.424939 -1.476542 -0.622860
H 0.611392 -2.359581 -0.010030
H 0.760398 -1.727512 -1.633171
C -1.062954 -1.136317 -0.637712
C -1.650939 -0.939562 0.781851
C -3.081159 -1.418516 0.972219
C -4.098195 -0.834458 -0.006218
C -5.518359 -1.332052 0.257086
C -6.543999 -0.737681 -0.708330
C -7.964104 -1.285966 -0.540089
C -8.608060 -0.952679 0.806410
H -8.620955 0.127836 0.974754
H -8.069252 -1.410687 1.638131
H -9.639792 -1.308552 0.847574
H -8.591549 -0.887240 -1.342910
H -7.946786 -2.372725 -0.677133
H -6.211198 -0.927252 -1.734319
H -6.561597 0.351916 -0.588290
H -5.541938 -2.425316 0.178200
H -5.794527 -1.092869 1.288415
H -3.816551 -1.084882 -1.033361
H -4.074441 0.256074 0.060952

H -3.380253 -1.179004 1.997143
H -3.075302 -2.511268 0.900715
H -1.004767 -1.413426 1.524300
O -1.583231 0.497381 0.969692
H -1.627833 -1.849111 -1.231587
O -1.233317 0.168256 -1.227289
C 3.556245 -1.460425 0.020874
O 3.353366 -2.646337 -0.155124
C 4.984510 -0.865299 0.215642
C 5.609464 -0.723534 -1.152507
C 6.705388 -1.354646 -1.551378
H 7.248407 -2.021023 -0.893186
H 7.101137 -1.208502 -2.548176
H 5.085962 -0.051960 -1.826944
C 4.725807 0.520067 0.848600
H 4.897725 0.455350 1.924845
H 5.367473 1.304243 0.449014
O 5.733135 -1.688998 1.090975
H 5.593142 -2.606302 0.814299
C 0.733865 3.390786 0.633008
H 1.115872 3.867709 1.536692
H 1.096753 3.992274 -0.205588
O -0.889201 2.877589 -1.704675
H -1.176378 2.227538 -2.358661
H -1.179827 4.381694 0.468862
H -1.171734 3.039224 1.603449

1a-c101, ΔG = 1.4019 kcal/mol, population = 0.58 %
C 1.372572 -2.787914 1.497165
C 1.414593 -2.803329 -0.021870
H 2.437072 -2.953706 -0.379800
C 0.945707 -1.455015 -0.549707
C -0.365494 -0.995631 0.062095
C -0.807430 -1.481196 1.300837
N -1.947974 -1.061757 1.879826
C -2.663339 -0.164903 1.217266
C -2.315478 0.343050 -0.039826
C -1.126646 -0.066599 -0.644571
C -0.670718 0.407002 -1.998237
H -0.710090 1.495832 -2.053113
H -1.366204 0.035266 -2.756052
C 0.739525 -0.096244 -2.292560
C 1.811263 0.522060 -1.357690
C 3.138231 0.796111 -2.043781

C 4.213228 1.439140 -1.161450
C 4.866575 0.501725 -0.142581
C 6.050073 1.128876 0.598849
C 5.675662 2.296325 1.514220
C 6.865255 2.828287 2.311624
H 7.293411 2.047133 2.945471
H 6.575379 3.660494 2.956881
H 7.655990 3.183112 1.645135
H 5.246788 3.110013 0.922893
H 4.888241 1.969748 2.202430
H 6.798990 1.467128 -0.127374
H 6.537906 0.356096 1.202863
H 4.121522 0.163250 0.579758
H 5.210107 -0.395441 -0.668358
H 3.778256 2.305195 -0.653310
H 4.996920 1.834564 -1.816255
H 2.922156 1.470491 -2.878217
H 3.508294 -0.138372 -2.477588
H 1.432637 1.438001 -0.895832
O 1.957958 -0.482780 -0.321089
H 0.992481 0.010760 -3.343729
O 0.810601 -1.498123 -1.961820
C -3.337660 1.287946 -0.476413
O -3.324049 2.067054 -1.409963
C -4.533613 1.179284 0.518149
C -5.599816 0.336430 -0.141198
C -6.816577 0.764345 -0.448916
H -7.136678 1.773808 -0.223605
H -7.535189 0.108136 -0.922762
H -5.301747 -0.683339 -0.367371
C -3.933658 0.450644 1.741150
H -3.703734 1.186797 2.513993
H -4.601183 -0.295130 2.170752
O -4.983101 2.474924 0.871668
H -5.036266 2.994382 0.056216
C -0.044801 -2.554727 2.026112
H -0.024911 -2.311690 3.089434
H -0.630258 -3.474255 1.934478
O 0.558282 -3.843258 -0.490746
H 0.523554 -3.783159 -1.453989
H 1.761082 -3.732608 1.878851
H 2.041846 -1.997988 1.842482

1a-c308, ΔG = 1.4062 kcal/mol, population = 0.58 %

C 1.011176 4.076452 1.180501
C 0.363306 3.864881 -0.177621
H -0.500256 4.524352 -0.302312
C -0.143346 2.433203 -0.273089
C 0.902949 1.402061 0.112249
C 1.987890 1.733473 0.936197
N 2.910446 0.831245 1.318261
C 2.762551 -0.406979 0.868969
C 1.735054 -0.813356 0.012881
C 0.761233 0.105279 -0.379830
C -0.372528 -0.226566 -1.312434
H -0.881841 -1.133866 -0.984522
H 0.035072 -0.443973 -2.303700
C -1.349731 0.940551 -1.390604
C -2.074102 1.206063 -0.045483
C -3.522931 1.640510 -0.178922
C -4.469193 0.511140 -0.601420
C -4.603090 -0.615313 0.424376
C -5.632701 -1.671126 0.025028
C -5.758165 -2.806281 1.040012
C -6.789006 -3.859166 0.636902
H -7.782780 -3.415418 0.533327
H -6.856417 -4.658240 1.378368
H -6.528867 -4.315063 -0.322106
H -4.779928 -3.281606 1.168212
H -6.024929 -2.387039 2.015942
H -5.362027 -2.089192 -0.951422
H -6.610389 -1.193499 -0.106523
H -3.637006 -1.107417 0.572775
H -4.880403 -0.186127 1.393797
H -4.146235 0.092082 -1.560493
H -5.457023 0.944523 -0.781933
H -3.569268 2.461223 -0.900208
H -3.844805 2.044194 0.785050
H -1.989077 0.336092 0.607313
O -1.302237 2.285969 0.539548
H -2.037709 0.834720 -2.224508
O -0.603590 2.159482 -1.587783
C 1.890616 -2.230799 -0.311900
O 1.125214 -2.956968 -0.909886
C 3.276235 -2.685089 0.254256
C 4.240102 -2.732502 -0.907525
C 4.676768 -3.846228 -1.481766
H 4.356972 -4.825218 -1.147116

H 5.374174 -3.809084 -2.308590
H 4.565905 -1.763558 -1.273068
C 3.664319 -1.551271 1.240795
H 3.448057 -1.879047 2.259751
H 4.717454 -1.275432 1.190352
O 3.080328 -3.944709 0.870235
H 3.944689 -4.299734 1.114846
C 2.211052 3.149653 1.390233
H 2.510658 3.137285 2.439177
H 3.072451 3.527249 0.831719
O 1.336740 4.113182 -1.190339
H 0.939241 3.889188 -2.041512
H 1.324220 5.116813 1.273525
H 0.253271 3.895487 1.944674

1a-c95, ΔG = 1.4495 kcal/mol, population = 0.54 %
C -0.269091 3.951378 1.270337
C -0.602784 3.713702 -0.193080
H -1.558537 4.177347 -0.453540
C -0.743773 2.218304 -0.439581
C 0.422045 1.409777 0.100929
C 1.236348 1.898864 1.132227
N 2.248718 1.184510 1.657728
C 2.463975 -0.018699 1.146706
C 1.726271 -0.566585 0.091005
C 0.662467 0.154093 -0.453410
C -0.180707 -0.337167 -1.599629
H -0.526447 -1.354069 -1.408374
H 0.438517 -0.387546 -2.499704
C -1.362497 0.599130 -1.828447
C -2.366352 0.598974 -0.648043
C -3.822489 0.755655 -1.046744
C -4.406531 -0.458947 -1.779426
C -4.314209 -1.792371 -1.028237
C -4.980998 -1.790678 0.346729
C -4.944380 -3.156253 1.031857
C -5.605340 -3.150929 2.408899
H -6.655930 -2.857074 2.337684
H -5.108791 -2.443990 3.078790
H -5.565700 -4.137313 2.875987
H -5.438075 -3.893722 0.390334
H -3.902717 -3.481047 1.126531
H -6.022414 -1.464171 0.242911
H -4.495432 -1.056893 0.998083

H -4.779478 -2.565924 -1.647107
H -3.266761 -2.091975 -0.918653
H -3.917669 -0.568235 -2.751976
H -5.457442 -0.245401 -1.995002
H -3.910561 1.642611 -1.680002
H -4.396499 0.958870 -0.140143
H -2.225773 -0.289705 -0.030318
O -1.963294 1.755953 0.130205
H -1.839258 0.415875 -2.786924
O -0.885586 1.960287 -1.828098
C 2.247003 -1.892950 -0.222387
O 1.782081 -2.756599 -0.941112
C 3.568701 -2.082754 0.583426
C 4.721130 -1.861236 -0.367746
C 5.631027 -2.775099 -0.676222
H 5.611398 -3.764994 -0.238266
H 6.434299 -2.551585 -1.366379
H 4.764581 -0.870433 -0.810851
C 3.514870 -0.970029 1.653549
H 3.199827 -1.406703 2.603331
H 4.471992 -0.474060 1.809361
O 3.580713 -3.363274 1.188970
H 3.292118 -4.001539 0.520440
C 1.053763 3.291630 1.666913
H 1.163557 3.266983 2.751954
H 1.890843 3.885891 1.288820
O 0.450630 4.248438 -0.992472
H 0.273056 4.008992 -1.911094
H -0.221252 5.024061 1.460134
H -1.088342 3.551335 1.870344

1a-c86, ΔG = 1.4596 kcal/mol, population = 0.53 %
C -0.705251 3.460738 0.732699
C -1.157893 2.752762 -0.533384
H -2.242744 2.825366 -0.652187
C -0.813460 1.273670 -0.430711
C 0.624450 1.020057 -0.017602
C 1.373814 1.988515 0.664830
N 2.640671 1.775078 1.066550
C 3.172476 0.596871 0.776305
C 2.507658 -0.411512 0.068955
C 1.188235 -0.213024 -0.338940
C 0.393950 -1.223870 -1.121791
H 0.434283 -2.199067 -0.634300

H 0.856913 -1.352825 -2.104133
C -1.052149 -0.763892 -1.282047
C -1.836588 -0.736636 0.051478
C -3.305183 -1.088581 -0.088570
C -4.083744 -0.957075 1.219974
C -5.545020 -1.401592 1.116400
C -6.394768 -0.540879 0.178235
C -7.891178 -0.866619 0.219127
C -8.233901 -2.272412 -0.275713
H -7.859410 -2.428785 -1.291248
H -9.314100 -2.432629 -0.290090
H -7.795178 -3.043016 0.360945
H -8.257546 -0.740659 1.243736
H -8.427616 -0.133216 -0.390365
H -6.039230 -0.647635 -0.851535
H -6.252088 0.511950 0.445129
H -5.577354 -2.447457 0.796018
H -5.991688 -1.373128 2.116122
H -4.041866 0.081323 1.560288
H -3.582167 -1.556220 1.987264
H -3.369541 -2.121159 -0.448485
H -3.736982 -0.450202 -0.863501
H -1.358885 -1.378894 0.795930
O -1.701566 0.642776 0.485790
H -1.568241 -1.327631 -2.054377
O -1.060510 0.625610 -1.669162
C 3.397262 -1.557124 -0.086357
O 3.149576 -2.682213 -0.475570
C 4.817227 -1.117426 0.385164
C 5.637790 -0.827500 -0.849342
C 6.736471 -1.483172 -1.197767
H 7.137552 -2.284225 -0.589664
H 7.277250 -1.224831 -2.099104
H 5.259058 -0.021158 -1.470983
C 4.556919 0.174414 1.191408
H 4.574511 -0.063422 2.256827
H 5.293464 0.955004 1.005732
O 5.381096 -2.121645 1.209395
H 5.226101 -2.972678 0.774279
C 0.808326 3.355858 0.931755
H 1.087407 3.658524 1.942031
H 1.319364 4.046075 0.254194
O -0.488691 3.340447 -1.647557
H -0.710445 2.820551 -2.430653

H -1.002754 4.508779 0.687285
H -1.231960 3.009504 1.575491

1a-c38, ΔG = 1.4615 kcal/mol, population = 0.53 %
C -1.178194 -2.963610 -0.939433
C -1.555131 -2.261377 0.354302
H -2.641835 -2.184845 0.452690
C -1.003705 -0.842484 0.334114
C 0.461072 -0.771765 -0.051644
C 1.081607 -1.799820 -0.772745
N 2.371619 -1.740202 -1.156009
C 3.050891 -0.658428 -0.807494
C 2.517773 0.397932 -0.059116
C 1.180104 0.359878 0.330558
C 0.516895 1.430457 1.155139
H 0.693074 2.413276 0.715383
H 0.981411 1.449590 2.145081
C -0.980009 1.162959 1.289116
C -1.753714 1.309891 -0.041176
C -3.174846 1.812392 0.128404
C -3.960923 1.838966 -1.180995
C -5.404499 2.328669 -1.024988
C -6.273996 1.497590 -0.074473
C -6.380731 0.018063 -0.445188
C -7.335542 -0.749876 0.467114
H -8.345568 -0.334511 0.417513
H -7.007233 -0.696948 1.508670
H -7.393612 -1.804578 0.189500
H -6.714445 -0.068946 -1.485026
H -5.391824 -0.447033 -0.402165
H -7.279637 1.930759 -0.056913
H -5.895144 1.582649 0.949266
H -5.394089 3.366984 -0.677185
H -5.874936 2.340735 -2.013621
H -3.952803 0.839690 -1.620619
H -3.441940 2.490158 -1.891928
H -3.130477 2.820617 0.553628
H -3.670847 1.174671 0.864667
H -1.202961 1.937714 -0.746060
O -1.776821 -0.046932 -0.559879
H -1.419704 1.750801 2.090252
O -1.180643 -0.230775 1.602620
C 3.554842 1.404566 0.151858
O 3.461013 2.539161 0.580528

C 4.904656 0.781422 -0.308397
C 5.642726 0.393139 0.954124
C 5.725887 -0.819565 1.485950
H 5.267224 -1.689876 1.033023
H 6.261106 -0.982505 2.412699
H 6.097684 1.239296 1.461621
C 4.483016 -0.398361 -1.196400
H 4.530811 -0.081300 -2.239972
H 5.110831 -1.280309 -1.087468
O 5.657056 1.732347 -1.055985
H 5.594292 2.579826 -0.591472
C 0.339515 -3.062642 -1.111333
H 0.594223 -3.355141 -2.130957
H 0.735086 -3.847900 -0.460506
O -1.000529 -2.992212 1.446467
H -1.169233 -2.488075 2.252753
H -1.620469 -3.960196 -0.951954
H -1.617475 -2.401863 -1.765742

1a-c162, ΔG = 1.4803 kcal/mol, population = 0.51 %
C 0.408479 -3.873475 1.011869
C 0.751889 -3.504383 -0.421498
H 1.744505 -3.876513 -0.690614
C 0.786613 -1.989005 -0.553498
C -0.446803 -1.309334 0.012840
C -1.245588 -1.932949 0.981928
N -2.321691 -1.336893 1.527853
C -2.615760 -0.118078 1.099209
C -1.895900 0.560240 0.109248
C -0.767942 -0.036456 -0.455190
C 0.061591 0.599799 -1.538147
H 0.326752 1.621591 -1.263227
H -0.539544 0.672568 -2.448904
C 1.314667 -0.228495 -1.801073
C 2.287999 -0.245148 -0.595223
C 3.760627 -0.263154 -0.963114
C 4.275381 1.053997 -1.554415
C 4.102062 2.290300 -0.660566
C 4.703339 2.170044 0.742995
C 6.209783 1.909849 0.761577
C 6.785530 1.883471 2.176383
H 6.314892 1.099077 2.775161
H 6.616228 2.835368 2.686534
H 7.861332 1.695569 2.167591

H 6.428560 0.959449 0.266755
H 6.713874 2.684103 0.173122
H 4.196237 1.376332 1.301645
H 4.498191 3.096719 1.288883
H 4.560051 3.143920 -1.170223
H 3.039528 2.534708 -0.570204
H 3.779492 1.244727 -2.510794
H 5.333194 0.922109 -1.793358
H 3.926088 -1.075444 -1.676143
H 4.325250 -0.517739 -0.063130
H 2.066802 0.581485 0.082118
O 1.955813 -1.485016 0.082074
H 1.799462 0.060969 -2.728881
O 0.939912 -1.616603 -1.914731
C -2.508176 1.863558 -0.123596
O -2.093039 2.810388 -0.763814
C -3.860129 1.894940 0.653237
C -4.967536 1.660576 -0.347013
C -5.923505 2.533850 -0.633161
H -5.980401 3.495141 -0.138314
H -6.688793 2.303560 -1.363130
H -4.933734 0.698018 -0.849360
C -3.748352 0.712582 1.641501
H -3.491398 1.099904 2.629421
H -4.668502 0.136825 1.732185
O -3.983229 3.123256 1.347959
H -3.717810 3.827338 0.738457
C -0.967759 -3.343928 1.421838
H -1.098488 -3.404692 2.503176
H -1.750707 -3.970214 0.984210
O -0.242929 -4.053310 -1.283682
H -0.061840 -3.739820 -2.178982
H 0.437876 -4.957765 1.123058
H 1.183202 -3.458916 1.659195

1a-c78, ΔG = 1.4828 kcal/mol, population = 0.51 %
C 0.067730 3.663795 -1.430603
C 0.390908 3.574865 0.051506
H 1.317457 4.109673 0.279249
C 0.606240 2.116840 0.431318
C -0.507065 1.202175 -0.046973
C -1.328007 1.552374 -1.126882
N -2.291347 0.738523 -1.599867
C -2.450208 -0.423070 -0.984902

C -1.703069 -0.833332 0.125724
C -0.689910 -0.011155 0.616142
C 0.158610 -0.350599 1.812587
H 0.558581 -1.361442 1.720572
H -0.470901 -0.347967 2.706963
C 1.289198 0.661703 1.963996
C 2.302637 0.606442 0.793137
C 3.742908 0.846080 1.204193
C 4.718360 0.962679 0.027607
C 4.722627 -0.230019 -0.934664
C 5.057742 -1.571449 -0.283560
C 5.134067 -2.721417 -1.287160
C 5.454229 -4.065127 -0.635313
H 4.691336 -4.335565 0.099530
H 6.415774 -4.029865 -0.116344
H 5.503825 -4.867157 -1.374970
H 4.181107 -2.792087 -1.822443
H 5.892368 -2.489605 -2.042541
H 4.309026 -1.817573 0.476445
H 6.013709 -1.487962 0.246717
H 3.752050 -0.305333 -1.435731
H 5.451296 -0.029497 -1.726536
H 5.725469 1.099914 0.433080
H 4.484113 1.870324 -0.533479
H 4.039282 0.022895 1.859988
H 3.784165 1.759488 1.804226
H 2.204934 -0.340084 0.257773
O 1.855951 1.672505 -0.083935
H 1.769538 0.588470 2.935936
O 0.742085 1.990521 1.838858
C -2.167994 -2.150571 0.553013
O -1.674041 -2.930289 1.345392
C -3.479166 -2.453439 -0.228378
C -4.613227 -2.216536 0.745004
C -5.390446 -1.143574 0.815533
H -5.293839 -0.304977 0.137011
H -6.160328 -1.066119 1.572619
H -4.733569 -3.020207 1.466135
C -3.435100 -1.479063 -1.414187
H -3.048650 -2.011144 -2.285684
H -4.403925 -1.062327 -1.681591
O -3.482472 -3.803610 -0.682514
H -3.153696 -4.353543 0.043790
C -1.211240 2.899956 -1.782319

H -1.300216 2.772698 -2.862117
H -2.085454 3.479067 -1.470698
O -0.700462 4.121391 0.789811
H -0.526369 3.968706 1.727423
H -0.034179 4.711276 -1.715897
H 0.916756 3.257296 -1.982972

1a-c17, ΔG = 1.5525 kcal/mol, population = 0.45 %
C 0.630391 -3.254476 1.329521
C 0.840230 -3.051279 -0.161981
H 1.863326 -3.310704 -0.448932
C 0.634127 -1.582155 -0.502664
C -0.656601 -1.013331 0.055540
C -1.299635 -1.598796 1.153844
N -2.426572 -1.091147 1.689175
C -2.925431 -0.005222 1.118805
C -2.367949 0.620171 -0.002592
C -1.191331 0.119373 -0.557231
C -0.520934 0.705523 -1.771586
H -0.393192 1.782427 -1.652316
H -1.174217 0.567046 -2.637589
C 0.824077 0.029565 -2.018787
C 1.864320 0.323372 -0.909934
C 3.290954 0.441409 -1.409829
C 4.309948 0.645547 -0.291151
C 5.739882 0.777970 -0.816285
C 6.802508 0.912278 0.277502
C 6.703551 2.195857 1.103805
C 7.828608 2.325983 2.129191
H 8.806575 2.330824 1.640528
H 7.814798 1.489302 2.832773
H 7.739210 3.248570 2.706821
H 6.721843 3.057216 0.427091
H 5.740548 2.232243 1.620325
H 7.794019 0.871026 -0.185817
H 6.742566 0.046953 0.948065
H 5.970360 -0.100041 -1.428593
H 5.800410 1.643170 -1.486627
H 4.256531 -0.196634 0.404592
H 4.034388 1.538203 0.278262
H 3.332550 1.288565 -2.102474
H 3.534524 -0.456949 -1.985850
H 1.584675 1.217570 -0.346488
O 1.743969 -0.826012 -0.030737

H 1.205094 0.245058 -3.013315
O 0.653679 -1.398549 -1.910130
C -3.193268 1.768078 -0.369180
O -2.966965 2.668588 -1.155143
C -4.501075 1.684777 0.470907
C -5.574349 1.182051 -0.469399
C -6.054396 -0.052519 -0.545384
H -5.720121 -0.852309 0.103639
H -6.809031 -0.308795 -1.278033
H -5.925873 1.941873 -1.161992
C -4.143926 0.727083 1.617042
H -3.884193 1.320780 2.495658
H -4.949602 0.052180 1.898463
O -4.843318 2.971519 0.978443
H -4.721449 3.608288 0.259022
C -0.788259 -2.875447 1.760711
H -0.853753 -2.796619 2.846848
H -1.486993 -3.665292 1.469929
O -0.100838 -3.858577 -0.867267
H -0.019631 -3.651238 -1.806994
H 0.831268 -4.295379 1.584609
H 1.363988 -2.642735 1.857500

1a-c118, ΔG = 1.5631 kcal/mol, population = 0.44 %
C 0.845007 3.501946 -0.526274
C 1.507669 2.476535 0.378668
H 2.592935 2.484339 0.243747
C 1.015801 1.085370 0.006724
C -0.495195 0.989071 -0.094594
C -1.288215 2.121251 -0.326249
N -2.625782 2.054391 -0.461422
C -3.181410 0.856530 -0.352130
C -2.468986 -0.317567 -0.081808
C -1.080168 -0.267998 0.044752
C -0.223928 -1.467555 0.352917
H -0.466793 -2.290985 -0.320104
H -0.456546 -1.817105 1.362795
C 1.255905 -1.108656 0.253425
C 1.694031 -0.762805 -1.191631
C 3.094605 -1.208698 -1.577590
C 4.212218 -0.702322 -0.668315
C 5.586556 -1.206559 -1.107767
C 6.751910 -0.610112 -0.311346
C 6.705968 -0.858726 1.200857

C 6.688800 -2.337675 1.586696
H 6.734862 -2.461279 2.670792
H 7.542881 -2.865639 1.153449
H 5.781505 -2.835542 1.238070
H 7.578025 -0.379247 1.655435
H 5.831778 -0.361059 1.630858
H 6.782959 0.470185 -0.488915
H 7.690371 -1.013786 -0.706468
H 5.608507 -2.298892 -1.039687
H 5.731415 -0.967922 -2.166511
H 4.019642 -1.011742 0.361466
H 4.207701 0.390812 -0.665832
H 3.281274 -0.880735 -2.604552
H 3.095665 -2.303719 -1.599401
H 0.972856 -1.164913 -1.906974
O 1.609047 0.685045 -1.223738
H 1.881998 -1.868340 0.712664
O 1.478518 0.132970 0.951996
C -3.398130 -1.440041 -0.014642
O -3.167946 -2.633420 0.025679
C -4.844824 -0.857382 -0.041169
C -5.373502 -0.881527 1.373248
C -6.429589 -1.576096 1.774070
H -7.008531 -2.177821 1.084863
H -6.755246 -1.549435 2.805964
H -4.812738 -0.276806 2.080242
C -4.652495 0.594376 -0.533776
H -4.916235 0.647272 -1.591923
H -5.264206 1.317427 0.004208
O -5.641274 -1.596306 -0.950283
H -5.467113 -2.535251 -0.790078
C -0.678332 3.494175 -0.377024
H -1.148451 4.055958 -1.185342
H -0.961720 3.999812 0.550744
O 1.166143 2.775151 1.731183
H 1.509417 2.061873 2.284508
H 1.236178 4.493349 -0.295831
H 1.127202 3.272362 -1.555411

1a-c111, ΔG = 1.5776 kcal/mol, population = 0.43 %
C -0.705508 -3.422109 -1.223029
C -0.970751 -3.127533 0.244451
H -2.010144 -3.348991 0.503168
C -0.745851 -1.645243 0.508757

C 0.576659 -1.133615 -0.031560
C 1.248759 -1.793351 -1.069789
N 2.402077 -1.336536 -1.592274
C 2.900069 -0.225640 -1.069655
C 2.315776 0.472534 -0.006407
C 1.109973 0.023050 0.533341
C 0.408430 0.690747 1.686002
H 0.306635 1.760994 1.500057
H 1.027482 0.591851 2.582128
C -0.958064 0.054666 1.921575
C -1.951256 0.302980 0.760115
C -3.392256 0.473750 1.199664
C -4.367644 0.633706 0.035993
C -5.809493 0.829923 0.502529
C -6.832180 0.905923 -0.636426
C -6.610273 2.042608 -1.641505
C -6.631809 3.436651 -1.015065
H -7.563759 3.605311 -0.468307
H -6.547727 4.212424 -1.779104
H -5.807438 3.576752 -0.312739
H -5.661706 1.893631 -2.165440
H -7.389002 1.981071 -2.407508
H -7.832208 1.011436 -0.202254
H -6.830664 -0.047071 -1.176228
H -6.084069 -0.000377 1.161261
H -5.869019 1.733581 1.117339
H -4.310125 -0.247226 -0.609758
H -4.052406 1.486376 -0.573154
H -3.441412 1.359137 1.842349
H -3.673969 -0.386211 1.815475
H -1.634131 1.158722 0.158083
O -1.820729 -0.896420 -0.048256
H -1.370032 0.334462 2.887302
O -0.813675 -1.380281 1.901297
C 3.142773 1.628580 0.322315
O 2.901794 2.569945 1.053252
C 4.472305 1.500735 -0.483511
C 5.532770 0.991540 0.463647
C 6.627124 1.659039 0.803180
H 6.845664 2.638176 0.395796
H 7.347596 1.234993 1.490721
H 5.339233 0.006392 0.878411
C 4.149764 0.449418 -1.568281
H 3.949620 0.962905 -2.510763

H 4.957914 -0.261619 -1.734708
O 4.797264 2.747510 -1.071655
H 4.674963 3.427639 -0.393432
C 0.737002 -3.096266 -1.616613
H 0.849526 -3.088359 -2.701615
H 1.406414 -3.878114 -1.246033
O -0.077146 -3.910685 1.033691
H -0.191922 -3.647505 1.955795
H -0.917859 -4.472475 -1.424423
H -1.404293 -2.829354 -1.816003

1a-c140, ΔG = 1.5863 kcal/mol, population = 0.43 %
C 0.184275 3.855761 -1.001498
C 0.705906 3.368689 0.339703
H 1.744887 3.676754 0.487557
C 0.678193 1.847323 0.366087
C -0.653939 1.264431 -0.066287
C -1.550310 1.993209 -0.860607
N -2.725421 1.487301 -1.278580
C -3.018253 0.253048 -0.895874
C -2.196352 -0.530951 -0.078257
C -0.968216 -0.027046 0.351595
C -0.025305 -0.777780 1.252874
H 0.159352 -1.778890 0.860864
H -0.502521 -0.912984 2.227555
C 1.285812 -0.014303 1.414830
C 2.115517 0.060609 0.110073
C 3.618804 0.041954 0.314839
C 4.144442 -1.345709 0.683250
C 5.648805 -1.376258 0.971324
C 6.544818 -0.948914 -0.198498
C 6.360040 -1.755432 -1.489072
C 6.636321 -3.250418 -1.331867
H 6.560119 -3.766888 -2.291050
H 5.928340 -3.722244 -0.647645
H 7.641731 -3.423015 -0.938032
H 5.345698 -1.612309 -1.873911
H 7.029305 -1.344507 -2.250665
H 7.588936 -1.030504 0.121761
H 6.379507 0.109998 -0.417036
H 5.857909 -0.724608 1.825802
H 5.921522 -2.386222 1.288799
H 3.903958 -2.045963 -0.122324
H 3.617768 -1.714017 1.568831

H 3.878453 0.766731 1.093075
H 4.084061 0.388043 -0.610260
H 1.818478 -0.731695 -0.581832
O 1.723287 1.338165 -0.456608
H 1.863415 -0.388104 2.255397
O 0.990805 1.374529 1.667629
C -2.836871 -1.820911 0.153055
O -2.382460 -2.831684 0.653442
C -4.290970 -1.731197 -0.404015
C -5.215766 -1.525828 0.772696
C -6.162675 -2.375725 1.146083
H -6.349935 -3.292107 0.600770
H -6.790437 -2.169607 2.003402
H -5.050143 -0.607948 1.329601
C -4.264831 -0.481496 -1.312179
H -4.184228 -0.801251 -2.352973
H -5.154186 0.140010 -1.216387
O -4.589508 -2.894062 -1.155356
H -4.286810 -3.656291 -0.640715
C -1.263107 3.419508 -1.240705
H -1.543040 3.565546 -2.284961
H -1.940650 4.045627 -0.652915
O -0.127252 3.895483 1.370638
H 0.159971 3.507420 2.207107
H 0.257267 4.942823 -1.045078
H 0.835453 3.454638 -1.780208

1a-c130, ΔG = 1.6027 kcal/mol, population = 0.41 %
C -1.555825 2.872772 1.329958
C -1.602156 2.807064 -0.187404
H -2.625777 2.938238 -0.549821
C -1.135360 1.432088 -0.642179
C 0.179695 1.008371 -0.014097
C 0.627619 1.563364 1.192963
N 1.775680 1.183855 1.784253
C 2.490785 0.255090 1.166811
C 2.133372 -0.327611 -0.054520
C 0.938361 0.042675 -0.672442
C 0.474354 -0.507546 -1.994152
H 0.513155 -1.597807 -1.986976
H 1.165774 -0.180320 -2.775782
C -0.937179 -0.021811 -2.309767
C -2.005154 -0.583257 -1.339433
C -3.351285 -0.853883 -1.989912

C -4.472945 -1.145286 -0.990837
C -4.248861 -2.399228 -0.145123
C -5.373543 -2.675713 0.858264
C -5.602641 -1.569344 1.896607
C -4.360445 -1.221512 2.717378
H -4.597904 -0.496575 3.498897
H -3.574830 -0.788740 2.094916
H -3.948680 -2.111696 3.201209
H -5.973118 -0.667124 1.401618
H -6.402135 -1.891396 2.570419
H -5.148913 -3.609542 1.384675
H -6.307330 -2.847110 0.312159
H -4.144740 -3.264460 -0.808177
H -3.302052 -2.317811 0.394596
H -5.413380 -1.248623 -1.541071
H -4.588594 -0.275468 -0.342002
H -3.226211 -1.700070 -2.673435
H -3.618717 0.015271 -2.597969
H -1.628495 -1.475079 -0.835918
O -2.143474 0.472046 -0.352568
H -1.194825 -0.187498 -3.352412
O -1.008740 1.396665 -2.056000
C 3.155950 -1.292384 -0.444227
O 3.135802 -2.128053 -1.327323
C 4.363696 -1.113392 0.525357
C 5.409518 -0.299283 -0.200014
C 6.627166 -0.730072 -0.500202
H 6.963997 -1.719963 -0.219344
H 7.329771 -0.095092 -1.024332
H 5.094233 0.700768 -0.483761
C 3.771975 -0.319116 1.711023
H 3.557530 -1.010645 2.528287
H 4.438403 0.455496 2.088114
O 4.833596 -2.380306 0.948324
H 4.876868 -2.947770 0.164895
C -0.136764 2.669413 1.866211
H -0.154111 2.477678 2.940085
H 0.446416 3.584741 1.729601
O -0.747294 3.820028 -0.714238
H -0.715503 3.708268 -1.672987
H -1.944070 3.835958 1.662203
H -2.223255 2.101653 1.718741

1a-c65, ΔG = 1.6146 kcal/mol, population = 0.41 %

C -1.044420 -2.952562 -1.590769
C -1.109229 -3.011880 -0.073691
H -2.112434 -3.290782 0.261142
C -0.811161 -1.632290 0.496973
C 0.441513 -1.001101 -0.080966
C 0.957221 -1.399129 -1.321047
N 2.048218 -0.830044 -1.869153
C 2.637242 0.130212 -1.173525
C 2.209356 0.563385 0.087061
C 1.073662 -0.004386 0.661798
C 0.548796 0.367896 2.022918
H 0.450756 1.451021 2.110271
H 1.278844 0.062541 2.777842
C -0.790111 -0.314407 2.285318
C -1.920368 0.194708 1.358032
C -3.285282 0.241680 2.020954
C -4.435549 0.570579 1.067054
C -4.326773 1.950802 0.414531
C -5.566084 2.358314 -0.386247
C -5.840373 1.489273 -1.615342
C -7.035436 1.981115 -2.429918
H -7.946967 1.980835 -1.826251
H -7.213209 1.348320 -3.302133
H -6.874483 3.002541 -2.784864
H -4.945781 1.473960 -2.247605
H -6.015395 0.454862 -1.307275
H -5.450651 3.397592 -0.711978
H -6.443411 2.339202 0.271018
H -4.147756 2.697167 1.195986
H -3.454207 1.982583 -0.245879
H -5.373809 0.517781 1.628411
H -4.490185 -0.204358 0.299908
H -3.240075 0.992520 2.816811
H -3.465166 -0.725105 2.500018
H -1.656734 1.166828 0.937732
O -1.930476 -0.779213 0.281653
H -1.063168 -0.272636 3.336257
O -0.687188 -1.703261 1.909558
C 3.107473 1.613401 0.561002
O 2.986760 2.372585 1.503775
C 4.330798 1.643636 -0.400399
C 5.458908 0.952313 0.333771
C 5.872518 -0.296509 0.161407
H 5.432246 -0.962717 -0.570210

H 6.676448 -0.701989 0.762377
H 5.914807 1.573167 1.100057
C 3.832630 0.910664 -1.654507
H 3.517681 1.654963 -2.388521
H 4.582176 0.276911 -2.124129
O 4.683708 2.988919 -0.708332
H 4.646845 3.493426 0.117514
C 0.343863 -2.539292 -2.084695
H 0.314831 -2.275710 -3.142882
H 1.033620 -3.384105 -1.999290
O -0.144314 -3.956346 0.386171
H -0.132755 -3.917941 1.351114
H -1.311342 -3.926398 -2.002288
H -1.796511 -2.238351 -1.930755

1a-c11, ΔG = 1.6171 kcal/mol, population = 0.40 %
C -0.934638 -4.047696 0.688982
C -0.066902 -3.550699 -0.455190
H 0.884887 -4.088953 -0.481664
C 0.252933 -2.078152 -0.240510
C -0.974190 -1.236377 0.058947
C -2.144523 -1.806392 0.577022
N -3.234440 -1.075662 0.881060
C -3.165650 0.226993 0.654423
C -2.050939 0.871287 0.105500
C -0.908588 0.132834 -0.198966
C 0.331676 0.725178 -0.812681
H 0.650051 1.605855 -0.253012
H 0.095818 1.070829 -1.823103
C 1.448222 -0.312076 -0.861967
C 1.948455 -0.731203 0.543977
C 3.435805 -1.018436 0.627149
C 4.284144 0.252173 0.593478
C 5.783094 -0.038413 0.679812
C 6.662704 1.213778 0.723227
C 6.623153 2.061014 -0.549679
C 7.573793 3.255775 -0.492172
H 7.530913 3.846454 -1.409801
H 8.607589 2.928500 -0.353010
H 7.320974 3.915976 0.341745
H 6.878733 1.428651 -1.406911
H 5.605706 2.420421 -0.726316
H 7.698532 0.910969 0.909292
H 6.367711 1.833602 1.578049

H 5.972661 -0.638210 1.575760
H 6.079539 -0.658792 -0.173535
H 3.994770 0.902478 1.426431
H 4.069944 0.811476 -0.321053
H 3.712106 -1.684357 -0.196328
H 3.625442 -1.562741 1.556204
H 1.665433 0.017486 1.288631
O 1.205212 -1.947663 0.810050
H 2.255785 -0.000357 -1.518126
O 0.913572 -1.547637 -1.379441
C -2.338497 2.297579 -0.023181
O -1.589065 3.221087 -0.278686
C -3.857291 2.490817 0.257492
C -4.511970 2.698084 -1.090578
C -5.185681 1.798991 -1.796287
H -5.355264 0.788016 -1.447306
H -5.592277 2.049212 -2.767870
H -4.346256 3.691543 -1.498214
C -4.266233 1.201346 0.984311
H -4.276372 1.397605 2.058212
H -5.249525 0.828059 0.705672
O -4.063652 3.625819 1.093905
H -3.503358 4.339989 0.756510
C -2.264215 -3.293079 0.765393
H -2.763269 -3.485575 1.716289
H -2.941628 -3.656194 -0.013050
O -0.780027 -3.728243 -1.677658
H -0.258736 -3.324293 -2.383193
H -1.119271 -5.115161 0.565132
H -0.372256 -3.920464 1.615610

1a-c66, ΔG = 1.6171 kcal/mol, population = 0.40 %
C -1.063952 -3.128809 -0.727361
C -1.273911 -2.576623 0.672532
H -2.331236 -2.614381 0.950108
C -0.851026 -1.114911 0.704624
C 0.522322 -0.875301 0.107318
C 1.093580 -1.774068 -0.802806
N 2.295920 -1.563242 -1.372451
C 2.938785 -0.458978 -1.024926
C 2.455733 0.473322 -0.099183
C 1.206648 0.278527 0.487685
C 0.608050 1.206992 1.510265
H 0.640711 2.237420 1.153303

H 1.220193 1.175930 2.415967
C -0.825978 0.797521 1.834071
C -1.796648 0.988263 0.644049
C -3.202294 1.389798 1.047516
C -4.167083 1.490386 -0.131790
C -5.589980 1.836657 0.304264
C -6.562548 2.069431 -0.856790
C -6.734362 0.885602 -1.816502
C -7.243724 -0.389344 -1.144950
H -7.420319 -1.177131 -1.880420
H -8.184884 -0.205702 -0.619222
H -6.528390 -0.775621 -0.416017
H -7.433992 1.180285 -2.604359
H -5.785898 0.677332 -2.320089
H -6.225646 2.940029 -1.429747
H -7.542616 2.333500 -0.445102
H -5.967926 1.040644 0.953482
H -5.563648 2.740778 0.921485
H -4.163222 0.543015 -0.674785
H -3.803819 2.251847 -0.831598
H -3.142597 2.356943 1.557335
H -3.574037 0.663816 1.777668
H -1.388328 1.700716 -0.077456
O -1.821142 -0.324970 0.024184
H -1.183674 1.275247 2.742130
O -0.869737 -0.629676 2.038389
C 3.432198 1.548971 0.049849
O 3.322450 2.627112 0.602595
C 4.732195 1.098036 -0.676947
C 5.696535 0.676838 0.410113
C 5.983366 -0.563191 0.784841
H 5.547436 -1.433944 0.311129
H 6.673180 -0.750204 1.597946
H 6.141822 1.516992 0.935917
C 4.264294 -0.035027 -1.602207
H 4.116899 0.374705 -2.603318
H 4.967301 -0.861648 -1.681893
O 5.272418 2.177088 -1.434758
H 5.218299 2.972267 -0.884560
C 0.406273 -3.065616 -1.149027
H 0.508119 -3.239017 -2.221236
H 0.967333 -3.864873 -0.656018
O -0.485771 -3.338883 1.584957
H -0.554555 -2.920609 2.452723

H -1.418405 -4.159206 -0.766602
H -1.681141 -2.545905 -1.413360

1a-c26, ΔG = 1.6391 kcal/mol, population = 0.39 %
C 1.011473 -3.050200 1.015837
C 1.572102 -2.136406 -0.061266
H 2.656811 -2.039074 0.038838
C 0.976423 -0.745352 0.101962
C -0.534535 -0.750343 0.237894
C -1.229560 -1.889577 0.662860
N -2.567538 -1.906756 0.819450
C -3.218146 -0.788713 0.537527
C -2.605626 0.382437 0.075338
C -1.220703 0.422613 -0.075826
C -0.468411 1.621317 -0.589824
H -0.756369 2.516935 -0.037364
H -0.755436 1.796615 -1.630560
C 1.037932 1.392908 -0.497119
C 1.550833 1.317393 0.961536
C 2.935747 1.897729 1.199819
C 4.036473 1.340091 0.299683
C 5.413060 1.907596 0.647038
C 6.530779 1.455240 -0.296266
C 6.805245 -0.049480 -0.279020
C 7.983282 -0.444489 -1.167925
H 7.799940 -0.162581 -2.208232
H 8.161572 -1.521688 -1.141429
H 8.901690 0.054528 -0.847385
H 7.000637 -0.364947 0.751841
H 5.913117 -0.593299 -0.601554
H 7.453153 1.982668 -0.030621
H 6.286209 1.764135 -1.319508
H 5.359669 3.001358 0.633945
H 5.670213 1.626035 1.674649
H 3.814139 1.564524 -0.748253
H 4.045886 0.252687 0.385858
H 3.195313 1.719488 2.247880
H 2.865540 2.983965 1.080364
H 0.833851 1.789014 1.637314
O 1.562611 -0.107952 1.232651
H 1.586693 2.112779 -1.097495
O 1.338420 0.074899 -0.998525
C -3.630126 1.397898 -0.154010
O -3.509760 2.588965 -0.370291

C -5.012072 0.690030 -0.054101
C -5.489317 0.497512 -1.477386
C -5.422326 -0.616423 -2.194992
H -5.018283 -1.541432 -1.802826
H -5.770572 -0.637052 -3.219750
H -5.878118 1.407716 -1.925550
C -4.705335 -0.610776 0.702569
H -4.945046 -0.462920 1.757399
H -5.269781 -1.471700 0.350396
O -5.929556 1.496949 0.677446
H -5.820182 2.409022 0.370688
C -0.509704 -3.185489 0.913455
H -0.917909 -3.643131 1.815731
H -0.767082 -3.855775 0.088139
O 1.231383 -2.674105 -1.337837
H 1.507801 -2.035963 -2.008026
H 1.478946 -4.032124 0.936627
H 1.290756 -2.634369 1.985585

1a-c103, ΔG = 1.6516 kcal/mol, population = 0.38 %
C 0.388696 -3.843523 0.429218
C 1.132705 -2.943148 -0.543102
H 2.211327 -3.113170 -0.481274
C 0.882443 -1.488438 -0.172384
C -0.586451 -1.164011 0.027863
C -1.523090 -2.159910 0.337018
N -2.824335 -1.890471 0.552556
C -3.200807 -0.624599 0.447649
C -2.339126 0.425019 0.108329
C -0.984568 0.164927 -0.102753
C 0.018171 1.217540 -0.494270
H -0.049811 2.075715 0.175747
H -0.230903 1.587311 -1.493009
C 1.430820 0.640419 -0.489430
C 1.917799 0.252426 0.928924
C 3.395421 0.485579 1.199262
C 4.346144 -0.186315 0.209646
C 5.824502 0.002850 0.566029
C 6.307832 1.456518 0.613563
C 6.139310 2.222513 -0.699260
C 6.718047 3.634910 -0.636220
H 6.584809 4.164890 -1.581831
H 7.788053 3.611255 -0.413694
H 6.230515 4.222089 0.146668

H 6.623288 1.661602 -1.506237
H 5.079266 2.281173 -0.962260
H 7.367685 1.460356 0.889336
H 5.790277 1.997600 1.412491
H 6.016381 -0.464714 1.537601
H 6.429895 -0.545714 -0.163019
H 4.160722 0.189217 -0.799662
H 4.124271 -1.254881 0.179373
H 3.612731 0.132764 2.212249
H 3.552680 1.567900 1.211028
H 1.322340 0.771056 1.683779
O 1.615100 -1.164315 1.004700
H 2.128120 1.290265 -1.010175
O 1.413543 -0.630270 -1.170233
C -3.093458 1.672930 0.067484
O -2.691571 2.817947 -0.010288
C -4.606339 1.312597 0.183057
C -5.199795 1.386612 -1.203771
C -6.152015 2.232114 -1.573551
H -6.590211 2.934157 -0.875425
H -6.532683 2.234560 -2.586785
H -4.779842 0.684567 -1.918369
C -4.603137 -0.140872 0.706368
H -4.806065 -0.131648 1.779068
H -5.347562 -0.774620 0.226133
O -5.235516 2.180400 1.109077
H -4.932077 3.079126 0.914670
C -1.123120 -3.608239 0.381308
H -1.614760 -4.081084 1.232687
H -1.542271 -4.078564 -0.513075
O 0.657229 -3.205018 -1.862200
H 1.067947 -2.562165 -2.454536
H 0.610296 -4.886003 0.198846
H 0.772288 -3.643058 1.431174

1a-c21, ΔG = 1.6692 kcal/mol, population = 0.37 %
C -0.147671 3.670781 0.713796
C -0.857694 2.898455 -0.385597
H -1.932530 3.099726 -0.367856
C -0.673388 1.405771 -0.151941
C 0.769377 1.010433 0.099450
C 1.713933 1.936439 0.559710
N 2.991921 1.600105 0.820744
C 3.335055 0.338998 0.609248

C 2.464524 -0.641042 0.117876
C 1.134852 -0.313589 -0.142648
C 0.126283 -1.284170 -0.697493
H 0.125553 -2.207041 -0.115614
H 0.425171 -1.560587 -1.712594
C -1.266118 -0.659236 -0.716892
C -1.828765 -0.397769 0.702267
C -3.327012 -0.599449 0.863593
C -4.196624 0.201222 -0.106007
C -5.696470 0.073571 0.174392
C -6.264952 -1.329573 -0.041492
C -7.779046 -1.401529 0.154127
C -8.343991 -2.803995 -0.064349
H -7.898355 -3.518892 0.632415
H -8.135157 -3.156127 -1.078005
H -9.426247 -2.827297 0.080525
H -8.026345 -1.059790 1.164783
H -8.263728 -0.700023 -0.533123
H -5.786871 -2.037891 0.642511
H -6.015611 -1.665697 -1.054930
H -5.900257 0.392146 1.203103
H -6.233780 0.772883 -0.474449
H -3.999127 -0.115533 -1.134801
H -3.908664 1.252685 -0.046451
H -3.591377 -0.333810 1.891992
H -3.521280 -1.670796 0.762901
H -1.298501 -1.011291 1.434275
O -1.487131 0.991690 0.940666
H -1.949028 -1.227736 -1.341385
O -1.167730 0.671906 -1.262245
C 3.189194 -1.903030 -0.007873
O 2.766822 -3.025144 -0.213656
C 4.699960 -1.590840 0.198149
C 5.322044 -1.598526 -1.181313
C 5.617610 -0.541892 -1.927357
H 5.452503 0.475194 -1.594046
H 6.036968 -0.663415 -2.917940
H 5.481622 -2.599285 -1.573044
C 4.702100 -0.223974 0.898284
H 4.815924 -0.384616 1.972132
H 5.502451 0.438057 0.574231
O 5.300923 -2.583233 1.024399
H 4.971583 -3.444255 0.727516
C 1.356317 3.386599 0.730327

H 1.807941 3.751516 1.653934
H 1.844547 3.930573 -0.083559
O -0.296625 3.274890 -1.642026
H -0.687608 2.707905 -2.319121
H -0.323095 4.738174 0.576552
H -0.598258 3.387323 1.666622

1a-c102, ΔG = 1.7093 kcal/mol, population = 0.35 %
C 0.852678 3.085138 -1.840786
C 0.959187 3.246316 -0.333423
H 1.944423 3.629186 -0.052476
C 0.798625 1.885227 0.328674
C -0.415393 1.118521 -0.163431
C -1.002199 1.392799 -1.405638
N -2.058096 0.702551 -1.877757
C -2.540967 -0.257541 -1.104584
C -2.037746 -0.573967 0.162823
C -0.936556 0.121047 0.660282
C -0.337350 -0.122945 2.019287
H -0.146105 -1.187061 2.165447
H -1.063991 0.167439 2.783311
C 0.948041 0.680962 2.189024
C 2.084694 0.210150 1.249777
C 3.470586 0.312586 1.861079
C 4.612945 0.025613 0.884437
C 4.605673 -1.389803 0.306182
C 5.815001 -1.669792 -0.586036
C 5.882902 -3.101413 -1.126688
C 4.727816 -3.473104 -2.057229
H 3.767858 -3.457299 -1.537751
H 4.861809 -4.475625 -2.469235
H 4.662472 -2.773695 -2.895452
H 6.827893 -3.228873 -1.663259
H 5.912853 -3.801482 -0.284600
H 6.727682 -1.461929 -0.017615
H 5.811472 -0.967225 -1.427710
H 4.587019 -2.117202 1.126649
H 3.687680 -1.546913 -0.265343
H 5.560163 0.193048 1.406181
H 4.577030 0.752516 0.068242
H 3.515230 -0.392426 2.697828
H 3.585372 1.316291 2.280586
H 1.888270 -0.803583 0.896531
O 1.977765 1.115428 0.120242

H 1.259426 0.725573 3.229165
O 0.716499 2.031420 1.738500
C -2.822253 -1.668714 0.728179
O -2.604264 -2.356061 1.707887
C -4.065547 -1.868136 -0.186636
C -5.233286 -1.247421 0.548213
C -5.772404 -0.056084 0.323896
H -5.422297 0.606875 -0.457603
H -6.593765 0.303459 0.930507
H -5.603437 -1.864622 1.362191
C -3.677938 -1.165251 -1.495714
H -3.322737 -1.918726 -2.201371
H -4.496421 -0.626639 -1.968855
O -4.295674 -3.256388 -0.409695
H -4.188825 -3.707389 0.440627
C -0.510433 2.528537 -2.258906
H -0.490932 2.200949 -3.299318
H -1.265928 3.317604 -2.201494
O -0.069386 4.132781 0.103408
H -0.049864 4.152351 1.068788
H 1.024371 4.049176 -2.320537
H 1.651381 2.414583 -2.162691

1a-c29, ΔG = 1.7169 kcal/mol, population = 0.34 %
C 0.753615 -3.241063 0.861754
C 1.203351 -2.532781 -0.405227
H 2.291532 -2.574326 -0.506889
C 0.812595 -1.064103 -0.325258
C -0.637264 -0.848435 0.065812
C -1.364412 -1.827681 0.754644
N -2.639797 -1.642904 1.146412
C -3.199745 -0.483545 0.837358
C -2.558483 0.532316 0.118643
C -1.233157 0.364510 -0.279117
C -0.460519 1.384614 -1.072738
H -0.542132 2.368712 -0.609050
H -0.910506 1.473969 -2.065525
C 1.002704 0.969105 -1.199665
C 1.752802 0.985249 0.155393
C 3.209614 1.417639 0.103904
C 4.083752 0.639910 -0.879953
C 5.546195 1.096038 -0.887992
C 6.305309 0.807127 0.407484
C 7.782664 1.192700 0.338472

C 8.538172 0.899310 1.633309
H 8.104373 1.448318 2.473313
H 9.589864 1.183841 1.556670
H 8.497490 -0.165428 1.877799
H 8.255494 0.656755 -0.491391
H 7.864563 2.258654 0.100304
H 6.221409 -0.260798 0.641093
H 5.841039 1.340105 1.242980
H 6.062829 0.597141 -1.714277
H 5.591457 2.169906 -1.103116
H 3.677017 0.743470 -1.889220
H 4.036626 -0.425058 -0.638106
H 3.610319 1.328218 1.116227
H 3.230463 2.484690 -0.142642
H 1.214277 1.608640 0.873013
O 1.667195 -0.394735 0.595483
H 1.512912 1.540228 -1.969926
O 1.057053 -0.423735 -1.568465
C -3.481969 1.650172 -0.055465
O -3.268714 2.779707 -0.453825
C -4.888746 1.164600 0.399986
C -5.676212 0.907139 -0.865775
C -5.930712 -0.270483 -1.421483
H -5.598757 -1.206495 -0.989781
H -6.487253 -0.336582 -2.347699
H -6.010745 1.818444 -1.353973
C -4.593769 -0.083123 1.244957
H -4.602514 0.202365 2.298656
H -5.313438 -0.888461 1.113459
O -5.523903 2.168005 1.187949
H -5.374162 3.018047 0.748762
C -0.764791 -3.176087 1.042309
H -1.046743 -3.476171 2.052518
H -1.248393 -3.887396 0.366352
O 0.569737 -3.152457 -1.523075
H 0.784015 -2.630962 -2.307220
H 1.080416 -4.280887 0.830035
H 1.257223 -2.767771 1.706608

1a-c46, ΔG = 1.7570 kcal/mol, population = 0.32 %
C 0.160793 -3.671858 1.103568
C 0.637934 -3.241378 -0.272913
H 1.670170 -3.560509 -0.443147
C 0.615509 -1.722218 -0.359651

C -0.698472 -1.114780 0.093268
C -1.567239 -1.803852 0.949569
N -2.723916 -1.272688 1.390129
C -3.025497 -0.055887 0.964050
C -2.232220 0.687260 0.081974
C -1.023405 0.160158 -0.369220
C -0.110160 0.866012 -1.335551
H 0.088367 1.883896 -0.997160
H -0.618418 0.955671 -2.299737
C 1.194211 0.092356 -1.502965
C 2.059971 0.069884 -0.218900
C 3.557089 0.087095 -0.463328
C 4.065167 1.462002 -0.898083
C 5.562259 1.487517 -1.225957
C 6.491282 1.082387 -0.076377
C 6.372827 1.958707 1.170832
C 7.381342 1.579951 2.254141
H 8.406491 1.679230 1.887678
H 7.242658 0.542827 2.571183
H 7.278650 2.214667 3.136947
H 6.513259 3.007310 0.886707
H 5.361620 1.886582 1.581456
H 7.524286 1.121338 -0.437640
H 6.311127 0.038453 0.200078
H 5.746768 0.826672 -2.079145
H 5.828113 2.496957 -1.556004
H 3.841800 2.191256 -0.113976
H 3.514118 1.793493 -1.783331
H 3.801287 -0.666678 -1.218695
H 4.048135 -0.219448 0.462420
H 1.776897 0.887169 0.449469
O 1.689610 -1.186346 0.406421
H 1.748575 0.428498 -2.374586
O 0.887809 -1.304464 -1.688813
C -2.886874 1.965017 -0.187422
O -2.455042 2.958445 -0.740947
C -4.326300 1.878634 0.398083
C -5.245031 1.648502 -0.782146
C -5.770149 0.492694 -1.167776
H -5.597412 -0.434808 -0.635911
H -6.396756 0.435275 -2.048663
H -5.425793 2.547179 -1.365350
C -4.249704 0.710610 1.392213
H -4.099245 1.117650 2.394032

H -5.142890 0.089638 1.415856
O -4.662591 3.090485 1.066417
H -4.365267 3.821977 0.505697
C -1.274335 -3.215495 1.377456
H -1.516035 -3.316812 2.436463
H -1.977054 -3.860161 0.841470
O -0.231417 -3.804864 -1.253347
H 0.026432 -3.449194 -2.113477
H 0.228578 -4.756964 1.186781
H 0.841891 -3.245876 1.842447

1a-c167, ΔG = 1.7577 kcal/mol, population = 0.32 %
C 0.751962 -3.412547 1.306021
C 0.973277 -3.206014 -0.183455
H 1.995621 -3.474145 -0.464920
C 0.783286 -1.733845 -0.520158
C -0.506712 -1.155425 0.030607
C -1.162159 -1.738388 1.124009
N -2.287845 -1.223518 1.652777
C -2.775448 -0.131874 1.081826
C -2.206494 0.490881 -0.035138
C -1.028215 -0.018065 -0.582521
C -0.344566 0.566957 -1.789742
H -0.209460 1.642415 -1.665447
H -0.992514 0.437293 -2.661115
C 0.997170 -0.118092 -2.028664
C 2.029815 0.163342 -0.909254
C 3.460936 0.267643 -1.404556
C 4.509864 0.369769 -0.294609
C 4.389098 1.629829 0.563710
C 5.444128 1.731290 1.671376
C 6.901198 1.764322 1.194897
C 7.217411 2.923063 0.249708
H 6.663908 2.841459 -0.688039
H 8.280747 2.950086 0.002366
H 6.955311 3.882001 0.705365
H 7.548104 1.831612 2.074691
H 7.155239 0.818163 0.708692
H 5.316536 0.887286 2.357638
H 5.247022 2.635442 2.257220
H 4.440769 2.511743 -0.083087
H 3.402802 1.659993 1.034857
H 5.496539 0.338895 -0.762902
H 4.444387 -0.515003 0.345304

H 3.524072 1.149093 -2.051388
H 3.668603 -0.605165 -2.030241
H 1.744854 1.054020 -0.346558
O 1.895710 -0.989475 -0.036960
H 1.387916 0.098402 -3.019259
O 0.815283 -1.545311 -1.926664
C -3.017126 1.645340 -0.407352
O -2.776297 2.538194 -1.196839
C -4.326895 1.592449 0.438297
C -5.420929 1.049333 -0.450072
C -6.510143 1.718315 -0.802981
H -6.698758 2.724609 -0.450534
H -7.255978 1.269130 -1.446057
H -5.257174 0.037327 -0.809176
C -3.994711 0.600569 1.575033
H -3.754783 1.165118 2.478214
H -4.812475 -0.080204 1.807879
O -4.613524 2.877796 0.959064
H -4.497651 3.514004 0.238436
C -0.665680 -3.021251 1.729712
H -0.737525 -2.945541 2.815634
H -1.369929 -3.803505 1.431685
O 0.028809 -4.001551 -0.897523
H 0.119796 -3.793814 -1.836270
H 0.940742 -4.456396 1.558235
H 1.488226 -2.810260 1.841081

1a-c7, ΔG = 1.7853 kcal/mol, population = 0.30 %
C 1.841909 2.506646 -0.339045
C 1.966646 1.671168 0.923891
H 3.012000 1.414301 1.116408
C 1.207664 0.365152 0.736704
C -0.201051 0.556166 0.211637
C -0.574363 1.712080 -0.485214
N -1.806257 1.879150 -1.003951
C -2.673070 0.895707 -0.817114
C -2.391539 -0.277603 -0.106626
C -1.116469 -0.473912 0.421332
C -0.713374 -1.689832 1.212756
H -0.987909 -2.599689 0.676969
H -1.277454 -1.702948 2.149681
C 0.785145 -1.673646 1.508482
C 1.662622 -1.847966 0.245268
C 2.960183 -2.594988 0.504583

C 3.831679 -2.852721 -0.730456
C 4.358074 -1.617289 -1.471440
C 5.188715 -0.666631 -0.612010
C 5.648864 0.576746 -1.371764
C 6.437219 1.552619 -0.500699
H 6.749250 2.433670 -1.065859
H 7.336123 1.080291 -0.095577
H 5.834400 1.895208 0.344707
H 6.257385 0.275500 -2.231244
H 4.769576 1.084492 -1.782583
H 6.062381 -1.196629 -0.214045
H 4.598365 -0.347098 0.249886
H 4.967702 -1.960735 -2.314293
H 3.521270 -1.062274 -1.900637
H 3.261536 -3.468393 -1.434199
H 4.682687 -3.464233 -0.413330
H 2.690874 -3.563859 0.936341
H 3.519349 -2.055559 1.273067
H 1.096172 -2.330285 -0.555625
O 1.934302 -0.477616 -0.149352
H 1.043905 -2.377944 2.294100
O 1.158546 -0.354927 1.958311
C -3.582289 -1.123302 -0.090649
O -3.706363 -2.284373 0.250547
C -4.763296 -0.270089 -0.638318
C -5.579433 0.142628 0.567129
C -5.549455 1.317511 1.182800
H -4.922476 2.135562 0.850510
H -6.158238 1.499775 2.059162
H -6.205285 -0.656433 0.954810
C -4.072287 0.886121 -1.375670
H -4.042603 0.648799 -2.440900
H -4.572978 1.845593 -1.262560
O -5.553456 -1.041751 -1.538091
H -5.671660 -1.917163 -1.141108
C 0.383931 2.859614 -0.644874
H 0.286778 3.257829 -1.655800
H 0.046232 3.651902 0.029618
O 1.420291 2.410410 2.014747
H 1.419609 1.833417 2.789204
H 2.431391 3.417397 -0.229761
H 2.275631 1.935444 -1.161713

1a-c41, ΔG = 1.7991 kcal/mol, population = 0.30 %

C -1.624301 -2.637817 -0.783884
C -1.965392 -1.635843 0.306119
H -3.024443 -1.367415 0.268807
C -1.163476 -0.361318 0.084108
C 0.317457 -0.614398 -0.126597
C 0.792852 -1.855098 -0.573135
N 2.098148 -2.091642 -0.801600
C 2.938391 -1.093076 -0.573141
C 2.552412 0.164136 -0.093680
C 1.201539 0.430765 0.133213
C 0.684692 1.739863 0.668188
H 1.093236 2.573007 0.094661
H 1.042445 1.867694 1.693760
C -0.841076 1.763687 0.641670
C -1.418167 1.754463 -0.797168
C -2.668602 2.600297 -0.997034
C -3.863780 2.254193 -0.097907
C -4.756220 1.140584 -0.650259
C -5.846112 0.704049 0.325427
C -6.733582 -0.413112 -0.221869
C -7.810503 -0.861391 0.764281
H -7.362815 -1.232904 1.689950
H -8.428082 -1.660483 0.348384
H -8.471520 -0.031127 1.026987
H -7.204336 -0.075637 -1.151300
H -6.104458 -1.268399 -0.490931
H -6.469984 1.565382 0.592037
H -5.378142 0.365739 1.257474
H -5.217506 1.484032 -1.583350
H -4.138609 0.280354 -0.912675
H -4.470550 3.153519 0.041373
H -3.509168 1.970773 0.896880
H -2.964829 2.534976 -2.048099
H -2.362506 3.637910 -0.833278
H -0.651761 2.074062 -1.507605
O -1.695268 0.349858 -1.028573
H -1.238806 2.572331 1.248268
O -1.331156 0.520224 1.183213
C 3.734704 1.005438 0.056884
O 3.812654 2.199414 0.273647
C 4.987062 0.098854 -0.148900
C 5.546803 -0.221934 1.216851
C 6.755933 0.123338 1.637751
H 7.446679 0.661114 1.000702

H 7.095948 -0.138040 2.631570
H 4.873100 -0.769663 1.869587
C 4.420718 -1.169052 -0.825583
H 4.622957 -1.118948 -1.897303
H 4.852450 -2.092533 -0.441921
O 5.916465 0.749971 -0.996550
H 5.989863 1.666966 -0.694343
C -0.141598 -3.017400 -0.763774
H 0.135427 -3.541844 -1.679415
H 0.050819 -3.714577 0.056887
O -1.638217 -2.209500 1.570648
H -1.769939 -1.529659 2.243871
H -2.238649 -3.530040 -0.659748
H -1.889283 -2.191170 -1.743813

1a-c40, ΔG = 1.8003 kcal/mol, population = 0.30 %
C 1.232259 -4.042964 -0.887571
C 0.412358 -3.736195 0.354349
H -0.448282 -4.407252 0.426758
C -0.128484 -2.316846 0.259685
C 0.937850 -1.292198 -0.082282
C 2.124127 -1.659392 -0.731337
N 3.070179 -0.764415 -1.075261
C 2.842251 0.500893 -0.758728
C 1.703112 0.947663 -0.079133
C 0.706915 0.037571 0.269436
C -0.543579 0.412585 1.018835
H -1.027744 1.268554 0.546444
H -0.269848 0.731616 2.028470
C -1.497532 -0.775146 1.085509
C -2.050198 -1.182619 -0.304206
C -3.486415 -1.676031 -0.296509
C -4.520249 -0.577734 -0.029329
C -4.591809 0.499725 -1.114913
C -5.734362 1.499639 -0.917859
C -5.597571 2.373904 0.330128
C -6.700101 3.425760 0.438176
H -6.582532 4.038202 1.334752
H -7.686391 2.956078 0.482343
H -6.690530 4.093900 -0.427073
H -5.608959 1.747560 1.226332
H -4.619462 2.867132 0.312927
H -6.686849 0.958499 -0.877357
H -5.788885 2.150639 -1.796691

H -3.648146 1.052750 -1.160737
H -4.707795 0.009844 -2.087233
H -4.321042 -0.115633 0.941250
H -5.502936 -1.050406 0.060876
H -3.571505 -2.461414 0.459657
H -3.687726 -2.142026 -1.265167
H -1.922188 -0.366619 -1.017179
O -1.177523 -2.272245 -0.701265
H -2.281733 -0.618580 1.820576
O -0.754679 -1.947211 1.478846
C 1.799719 2.391664 0.117327
O 0.954103 3.184166 0.487036
C 3.247684 2.814219 -0.266245
C 3.983764 3.025175 1.038950
C 4.823454 2.181085 1.624481
H 5.087830 1.224178 1.191864
H 5.277871 2.422734 2.576845
H 3.730145 3.962298 1.526767
C 3.761782 1.640591 -1.113224
H 3.643323 1.896511 -2.167902
H 4.809156 1.398751 -0.944894
O 3.225521 4.017028 -1.028814
H 2.588469 4.613436 -0.608791
C 2.430965 -3.101405 -1.026462
H 2.861380 -3.169286 -2.026619
H 3.221686 -3.403674 -0.333614
O 1.252533 -3.869365 1.499287
H 0.746472 -3.581908 2.269980
H 1.574560 -5.077507 -0.848306
H 0.574486 -3.950932 -1.753627

1a-c108, ΔG = 1.8079 kcal/mol, population = 0.29 %
C 0.407221 3.655118 -0.824834
C 0.844060 3.085951 0.514847
H 1.903771 3.288116 0.695017
C 0.668403 1.573946 0.500829
C -0.703640 1.135337 0.022512
C -1.500940 1.964682 -0.778327
N -2.706952 1.584916 -1.240434
C -3.131119 0.379118 -0.892571
C -2.416285 -0.496685 -0.067272
C -1.156664 -0.125652 0.404845
C -0.317816 -0.984692 1.312540
H -0.228587 -1.993163 0.906067

H -0.827846 -1.085555 2.274712
C 1.060406 -0.362731 1.514237
C 1.913765 -0.345048 0.222457
C 3.397255 -0.553182 0.458301
C 4.230608 -0.448864 -0.818333
C 5.740231 -0.563461 -0.587167
C 6.193567 -1.922375 -0.049979
C 7.707843 -2.042234 0.152815
C 8.520332 -1.986623 -1.141506
H 8.417343 -1.023994 -1.645673
H 8.193537 -2.762936 -1.838964
H 9.583377 -2.141585 -0.944741
H 8.042358 -1.247950 0.829055
H 7.918849 -2.986431 0.663785
H 5.858802 -2.708958 -0.736947
H 5.699807 -2.119512 0.905436
H 6.248062 -0.357889 -1.532881
H 6.058701 0.221479 0.108914
H 4.016281 0.508563 -1.298977
H 3.910544 -1.227823 -1.519812
H 3.519385 -1.540266 0.912159
H 3.740023 0.183462 1.192343
H 1.536754 -1.075106 -0.498608
O 1.678502 0.985068 -0.310866
H 1.585327 -0.813025 2.352217
O 0.900378 1.042463 1.796493
C -3.189926 -1.719042 0.121693
O -2.854986 -2.778413 0.615721
C -4.609780 -1.474197 -0.475297
C -5.545512 -1.190899 0.676131
C -6.589535 -1.939889 1.003857
H -6.856110 -2.823072 0.437040
H -7.219800 -1.679752 1.844503
H -5.302488 -0.304218 1.254813
C -4.429468 -0.221907 -1.361388
H -4.344215 -0.535111 -2.403810
H -5.255792 0.483733 -1.285894
O -5.001161 -2.590482 -1.254075
H -4.792943 -3.386146 -0.743094
C -1.067194 3.363775 -1.115146
H -1.300118 3.558815 -2.162933
H -1.700045 4.039000 -0.531862
O 0.039040 3.665995 1.539702
H 0.259295 3.225781 2.370693

H 0.582223 4.731316 -0.834104
H 1.040600 3.216490 -1.597933

1a-c192, ΔG = 1.8135 kcal/mol, population = 0.29 %
C 1.783989 -2.690248 1.946637
C 1.881833 -2.946058 0.451933
H 2.918539 -3.134170 0.158321
C 1.418454 -1.706754 -0.299506
C 0.078978 -1.178056 0.181586
C -0.398990 -1.462146 1.468628
N -1.561532 -0.970658 1.935709
C -2.262363 -0.202749 1.114412
C -1.878662 0.098487 -0.197308
C -0.668071 -0.389112 -0.691299
C -0.172406 -0.138620 -2.090858
H -0.235426 0.923369 -2.332108
H -0.828246 -0.655358 -2.797225
C 1.259102 -0.641232 -2.241116
C 2.263378 0.147666 -1.362712
C 3.617189 0.368445 -2.008410
C 4.673638 1.011005 -1.102643
C 4.298411 2.395903 -0.546802
C 3.611869 2.371701 0.821435
C 3.195881 3.757429 1.311371
C 2.525850 3.727334 2.683937
H 2.236009 4.728199 3.011199
H 3.197804 3.310747 3.438969
H 1.624952 3.108085 2.665879
H 4.075939 4.408676 1.346476
H 2.513102 4.205177 0.581201
H 4.295039 1.920337 1.550025
H 2.733086 1.722917 0.796938
H 5.204357 3.003254 -0.459584
H 3.658732 2.917795 -1.268004
H 5.592293 1.093200 -1.688651
H 4.899867 0.334477 -0.274107
H 3.459768 1.005629 -2.884866
H 3.983250 -0.595521 -2.374453
H 1.823016 1.098528 -1.057087
O 2.408301 -0.691744 -0.189616
H 1.563750 -0.689907 -3.282994
O 1.342422 -1.972818 -1.692178
C -2.892113 0.949571 -0.811309
O -2.853516 1.567374 -1.857933

C -4.117618 0.992808 0.152488
C -5.163977 0.051723 -0.396453
C -6.368771 0.420839 -0.809583
H -6.694159 1.452343 -0.761291
H -7.072818 -0.305419 -1.194875
H -4.861612 -0.990464 -0.445184
C -3.554236 0.471314 1.493022
H -3.357186 1.321579 2.148943
H -4.230785 -0.206948 2.011317
O -4.576720 2.326333 0.283012
H -4.609889 2.708437 -0.606088
C 0.346968 -2.394435 2.382048
H 0.326661 -1.983985 3.392554
H -0.227555 -3.324730 2.419287
O 1.055383 -4.061498 0.124518
H 1.059631 -4.158299 -0.836360
H 2.165239 -3.557058 2.487176
H 2.434120 -1.846607 2.184733

1a-c43, ΔG = 1.8267 kcal/mol, population = 0.28 %
C -1.867169 2.616316 0.181218
C -1.984295 1.729707 -1.046948
H -3.029708 1.477485 -1.245735
C -1.247360 0.422798 -0.791434
C 0.160237 0.620899 -0.260066
C 0.538344 1.803985 0.390017
N 1.768198 1.986951 0.905506
C 2.633084 0.993594 0.762679
C 2.349885 -0.204655 0.096681
C 1.074348 -0.417156 -0.427865
C 0.672747 -1.660270 -1.176035
H 0.949051 -2.550627 -0.609456
H 1.233773 -1.705548 -2.113820
C -0.825888 -1.650453 -1.463381
C -1.689622 -1.756277 -0.180465
C -2.958907 -2.569489 -0.362163
C -3.822872 -2.727182 0.894205
C -4.525798 -1.462873 1.403720
C -5.483930 -0.837532 0.389227
C -6.315239 0.321009 0.949536
C -5.489989 1.553587 1.320928
H -4.939354 1.926655 0.453999
H -4.762299 1.333209 2.103932
H -6.129077 2.362176 1.682348

H -7.066346 0.608112 0.207411
H -6.869831 -0.026317 1.828163
H -6.161071 -1.614821 0.017661
H -4.918934 -0.479794 -0.476672
H -5.093167 -1.730038 2.302265
H -3.780806 -0.728502 1.709455
H -3.199787 -3.138663 1.695447
H -4.583870 -3.485188 0.681743
H -2.647628 -3.566243 -0.689818
H -3.536153 -2.136134 -1.182771
H -1.098601 -2.159819 0.646403
O -2.001172 -0.370075 0.116383
H -1.096482 -2.392573 -2.209177
O -1.192469 -0.353846 -1.977945
C 3.534945 -1.055560 0.114023
O 3.651913 -2.227078 -0.190076
C 4.721252 -0.197730 0.651534
C 5.539028 0.243169 -0.539516
C 6.806740 -0.084699 -0.748548
H 7.358033 -0.696754 -0.045948
H 7.335729 0.265365 -1.625581
H 5.007189 0.866009 -1.253137
C 4.030449 1.009886 1.322829
H 4.004732 0.847686 2.402250
H 4.534759 1.956572 1.133785
O 5.465228 -0.944651 1.596888
H 5.593104 -1.831008 1.228499
C -0.410326 2.965349 0.493721
H -0.322542 3.400263 1.490277
H -0.053924 3.728092 -0.204790
O -1.410371 2.413153 -2.159586
H -1.407186 1.803714 -2.908739
H -2.444407 3.528267 0.025796
H -2.318883 2.085975 1.020623

1a-c125, ΔG = 1.8430 kcal/mol, population = 0.28 %
C 0.429674 -3.676377 0.618854
C 1.101171 -2.817064 -0.439487
H 2.187685 -2.936484 -0.403897
C 0.799126 -1.351891 -0.161715
C -0.675460 -1.074670 0.068267
C -1.556139 -2.083862 0.481654
N -2.859097 -1.853229 0.729599
C -3.294356 -0.614846 0.550565

C -2.492963 0.441654 0.102075
C -1.136502 0.223670 -0.141372
C -0.195750 1.286201 -0.643904
H -0.283721 2.189386 -0.038460
H -0.490007 1.566715 -1.659255
C 1.240457 0.771507 -0.641132
C 1.780948 0.504562 0.786132
C 3.249374 0.832537 1.003358
C 4.216104 0.139856 0.042833
C 5.689586 0.442874 0.333042
C 6.085626 1.902847 0.105358
C 7.568266 2.197910 0.357431
C 8.519955 1.506225 -0.619480
H 8.278223 1.773238 -1.652060
H 8.467516 0.418943 -0.537768
H 9.555340 1.799662 -0.433261
H 7.724752 3.279216 0.299620
H 7.822069 1.907508 1.382747
H 5.488777 2.548176 0.755478
H 5.832004 2.185099 -0.923734
H 5.921002 0.164700 1.368074
H 6.302362 -0.202309 -0.301439
H 3.991079 0.429151 -0.988322
H 4.052381 -0.937975 0.101606
H 3.505928 0.563834 2.032897
H 3.347208 1.919505 0.935664
H 1.176386 1.041767 1.520631
O 1.555170 -0.917110 0.963573
H 1.895260 1.411857 -1.224991
O 1.259625 -0.543326 -1.233381
C -3.304296 1.649555 -0.003065
O -2.958392 2.801704 -0.180767
C -4.794144 1.233451 0.193433
C -5.431215 1.164828 -1.174651
C -6.433337 1.932089 -1.581318
H -6.884398 2.670554 -0.930418
H -6.843129 1.831310 -2.578054
H -4.999878 0.424581 -1.842464
C -4.705766 -0.170842 0.830565
H -4.865473 -0.082113 1.906981
H -5.439792 -0.871714 0.435234
O -5.436268 2.145481 1.065742
H -5.178890 3.037496 0.790706
C -1.091519 -3.507965 0.609650

H -1.533729 -3.940759 1.508063
H -1.518965 -4.055873 -0.235277
O 0.595679 -3.192668 -1.719529
H 0.959678 -2.578810 -2.370267
H 0.689014 -4.722391 0.452666
H 0.837559 -3.390741 1.590063

1a-c13, ΔG = 1.8706 kcal/mol, population = 0.26 %
C -1.463051 -4.081017 0.618817
C -0.480650 -3.727023 -0.485627
H 0.363294 -4.422773 -0.492305
C 0.082956 -2.336572 -0.230539
C -0.987398 -1.296617 0.047778
C -2.254068 -1.659760 0.524162
N -3.204839 -0.753432 0.822034
C -2.900913 0.520572 0.627954
C -1.678022 0.966834 0.113612
C -0.676744 0.043639 -0.182289
C 0.663639 0.418050 -0.756346
H 1.107157 1.233001 -0.182663
H 0.521525 0.798189 -1.771877
C 1.586175 -0.795613 -0.777836
C 1.945374 -1.301586 0.646229
C 3.361688 -1.826132 0.824656
C 4.393445 -0.741740 1.151921
C 4.582466 0.333336 0.082028
C 5.703038 1.316997 0.415465
C 5.879122 2.410574 -0.636862
C 6.999642 3.392141 -0.298386
H 7.101689 4.163316 -1.064949
H 7.960050 2.876802 -0.213507
H 6.807377 3.892295 0.654542
H 6.079978 1.945212 -1.607633
H 4.935896 2.955782 -0.748147
H 6.644825 0.768332 0.529056
H 5.499080 1.780753 1.387378
H 4.795609 -0.142910 -0.881598
H 3.655097 0.899421 -0.050624
H 5.354857 -1.231606 1.331857
H 4.114487 -0.260953 2.095937
H 3.653548 -2.379042 -0.073755
H 3.337776 -2.546822 1.645157
H 1.737025 -0.525587 1.388195
O 1.008160 -2.390747 0.849295

H 2.460150 -0.629219 -1.397824
O 0.866676 -1.913663 -1.335387
C -1.706194 2.423531 0.010691
O -0.799529 3.204894 -0.206653
C -3.173635 2.876306 0.263476
C -3.753984 3.206159 -1.094389
C -4.556890 2.442422 -1.824035
H -4.903649 1.471871 -1.491468
H -4.894775 2.767962 -2.799614
H -3.413778 4.161175 -1.485292
C -3.816199 1.669369 0.962806
H -3.805421 1.847272 2.039928
H -4.846317 1.482118 0.667076
O -3.197519 4.022026 1.109717
H -2.502520 4.622769 0.803076
C -2.640586 -3.105202 0.668557
H -3.203513 -3.226301 1.595060
H -3.338979 -3.326774 -0.143876
O -1.168751 -3.758572 -1.734783
H -0.561147 -3.436563 -2.412965
H -1.827775 -5.097112 0.465588
H -0.920214 -4.069507 1.565667

1a-c44, ΔG = 1.8913 kcal/mol, population = 0.25 %
C -0.015783 3.782043 0.750487
C -0.710152 3.094850 -0.413358
H -1.772209 3.355132 -0.440551
C -0.620337 1.586420 -0.232102
C 0.785082 1.100674 0.067582
C 1.754419 1.953210 0.612057
N 2.996373 1.534744 0.922523
C 3.281059 0.265566 0.674575
C 2.385788 -0.642338 0.096890
C 1.090343 -0.231048 -0.212221
C 0.057812 -1.122135 -0.849574
H -0.020859 -2.063724 -0.304182
H 0.387759 -1.378261 -1.860331
C -1.295629 -0.420216 -0.904486
C -1.915401 -0.177829 0.494341
C -3.429438 -0.266681 0.537532
C -3.941165 -1.706325 0.457689
C -5.461532 -1.825483 0.595260
C -6.244412 -1.182630 -0.551817
C -7.751717 -1.452322 -0.506396

C -8.455377 -0.839675 0.705117
H -9.534373 -1.001529 0.657581
H -8.280912 0.238983 0.751134
H -8.098679 -1.272716 1.641498
H -8.206501 -1.057951 -1.420032
H -7.923523 -2.534086 -0.520679
H -5.844191 -1.557174 -1.500337
H -6.080559 -0.100476 -0.555190
H -5.726641 -2.886708 0.647890
H -5.765002 -1.384867 1.549589
H -3.464887 -2.293327 1.249388
H -3.631679 -2.159813 -0.490044
H -3.833335 0.339228 -0.277219
H -3.768706 0.187634 1.472759
H -1.472538 -0.850748 1.233113
O -1.506903 1.179155 0.805243
H -1.979373 -0.923222 -1.582365
O -1.105151 0.924365 -1.390371
C 3.046149 -1.936856 -0.049174
O 2.575037 -3.022415 -0.331113
C 4.557895 -1.722116 0.253405
C 5.259215 -1.712605 -1.087296
C 5.672331 -0.647263 -1.761628
H 5.556997 0.362609 -1.387768
H 6.143031 -0.753226 -2.730712
H 5.374004 -2.703697 -1.517517
C 4.596516 -0.386702 1.010913
H 4.640865 -0.596933 2.081307
H 5.449437 0.240475 0.759812
O 5.052075 -2.780012 1.069699
H 4.696483 -3.608434 0.715840
C 1.467856 3.413695 0.826503
H 1.891699 3.715562 1.785333
H 2.025178 3.961428 0.060876
O -0.064471 3.485965 -1.623578
H -0.451143 2.970054 -2.342619
H -0.126358 4.862189 0.650504
H -0.527637 3.486285 1.667965

1a-c90, ΔG = 1.9359 kcal/mol, population = 0.24 %
C 0.564056 -3.274956 1.652088
C 0.701439 -3.334548 0.139714
H 1.663408 -3.769668 -0.145835
C 0.660718 -1.921379 -0.423527

C -0.509639 -1.103835 0.092498
C -1.147489 -1.418487 1.299396
N -2.162088 -0.685388 1.796882
C -2.555796 0.356524 1.081335
C -2.000306 0.718576 -0.151659
C -0.936749 -0.017615 -0.671090
C -0.282842 0.275957 -1.994504
H -0.018435 1.332183 -2.062176
H -1.003218 0.086188 -2.795189
C 0.951383 -0.599908 -2.185000
C 2.084092 -0.276982 -1.179330
C 3.481587 -0.418685 -1.755578
C 4.601862 -0.305885 -0.719147
C 4.655358 1.036715 0.010347
C 5.848798 1.140939 0.960773
C 5.882184 2.425211 1.794606
C 6.041336 3.701768 0.968051
H 6.132232 4.578872 1.612418
H 5.187359 3.866099 0.308195
H 6.937917 3.651883 0.343753
H 4.965556 2.488437 2.391146
H 6.707804 2.358088 2.509435
H 6.777376 1.060959 0.383533
H 5.833346 0.279840 1.637242
H 4.692538 1.844151 -0.727358
H 3.735711 1.186129 0.585500
H 5.556597 -0.473267 -1.227144
H 4.494967 -1.112313 0.011377
H 3.604680 0.356914 -2.518739
H 3.545987 -1.384466 -2.265177
H 1.938478 0.716635 -0.751889
O 1.884878 -1.258679 -0.129035
H 1.295516 -0.591362 -3.215720
O 0.613219 -1.959561 -1.842079
C -2.698296 1.896261 -0.661398
O -2.412052 2.629448 -1.588912
C -3.951044 2.114650 0.235497
C -5.132873 1.602195 -0.558630
C -5.743071 0.433193 -0.412452
H -5.450053 -0.290531 0.338141
H -6.568453 0.154982 -1.055305
H -5.448469 2.282961 -1.344467
C -3.636289 1.315709 1.508369
H -3.244158 2.002954 2.260616

H -4.497884 0.808343 1.937378
O -4.110172 3.498521 0.533043
H -3.948926 3.990069 -0.285690
C -0.763866 -2.644946 2.079688
H -0.747081 -2.393139 3.141037
H -1.575026 -3.367553 1.950621
O -0.376604 -4.107081 -0.385351
H -0.330885 -4.058205 -1.348835
H 0.647506 -4.281781 2.062459
H 1.402309 -2.694745 2.041860

1a-c120, ΔG = 1.9559 kcal/mol, population = 0.23 %
C 1.371652 -4.055048 0.927555
C 1.863223 -3.342260 -0.321577
H 2.953648 -3.390325 -0.391781
C 1.479753 -1.871516 -0.242769
C 0.016852 -1.651621 0.099631
C -0.736947 -2.633043 0.759074
N -2.024656 -2.448704 1.104603
C -2.573069 -1.286180 0.783066
C -1.905152 -0.268394 0.093133
C -0.566290 -0.438146 -0.260664
C 0.232233 0.585008 -1.022889
H 0.130236 1.567272 -0.559584
H -0.179060 0.676838 -2.032047
C 1.697617 0.170211 -1.088898
C 2.386155 0.173055 0.299649
C 3.840438 0.610658 0.281614
C 4.022866 2.109400 0.018889
C 3.515844 3.008630 1.148104
C 3.743586 4.503286 0.896115
C 3.045086 5.065875 -0.348182
C 1.529154 4.870468 -0.350044
H 1.070578 5.360547 -1.211315
H 1.077838 5.289308 0.553560
H 1.258915 3.813592 -0.393365
H 3.270151 6.134334 -0.414906
H 3.469842 4.613994 -1.249046
H 4.819400 4.691058 0.813791
H 3.399567 5.060912 1.773599
H 2.451040 2.832846 1.320327
H 4.021032 2.725439 2.076962
H 3.528443 2.375484 -0.919512
H 5.087155 2.306896 -0.139005

H 4.360299 0.027614 -0.483661
H 4.287499 0.346949 1.243971
H 1.808123 0.769163 1.007414
O 2.309960 -1.216324 0.710162
H 2.244378 0.745993 -1.830037
O 1.772072 -1.219093 -1.468871
C -2.811425 0.857951 -0.100635
O -2.572013 1.985303 -0.488412
C -4.237613 0.394782 0.328679
C -5.018883 0.094706 -0.928371
C -6.115578 0.736600 -1.307430
H -6.543135 1.532080 -0.710168
H -6.628097 0.471846 -2.223281
H -4.612093 -0.706565 -1.538595
C -3.981532 -0.893411 1.142411
H -4.047296 -0.659461 2.206777
H -4.693414 -1.688865 0.925886
O -4.839108 1.389513 1.138246
H -4.690231 2.242803 0.705455
C -0.150935 -3.983996 1.061570
H -0.465427 -4.285502 2.061698
H -0.616769 -4.691196 0.369090
O 1.257460 -3.950628 -1.460694
H 1.505651 -3.432996 -2.237339
H 1.694360 -5.096217 0.900064
H 1.851295 -3.588627 1.789876

1a-c42, ΔG = 1.9767 kcal/mol, population = 0.22 %
C 0.119083 3.811414 -0.650040
C 0.927028 3.017587 0.362884
H 1.991499 3.255948 0.282716
C 0.774928 1.530949 0.073148
C -0.667716 1.096609 -0.101276
C -1.670751 2.008980 -0.452879
N -2.951077 1.639221 -0.648224
C -3.237269 0.357437 -0.480102
C -2.303789 -0.613052 -0.097047
C -0.971733 -0.250222 0.094442
C 0.103682 -1.208427 0.532450
H 0.099458 -2.099552 -0.096816
H -0.120260 -1.547685 1.547759
C 1.472646 -0.534957 0.498975
C 1.948596 -0.184628 -0.931289
C 3.449851 -0.271118 -1.133432

C 3.959424 -1.710787 -1.203966
C 5.458831 -1.814875 -1.504978
C 6.378463 -1.114509 -0.498639
C 6.232402 -1.606827 0.941308
C 7.231691 -0.951115 1.892690
H 7.103117 0.134520 1.906410
H 8.260223 -1.158423 1.585892
H 7.108260 -1.313939 2.915375
H 5.217390 -1.411482 1.298582
H 6.360437 -2.694530 0.964230
H 6.207509 -0.033467 -0.526509
H 7.415379 -1.261222 -0.818436
H 5.729734 -2.874439 -1.554453
H 5.647083 -1.402104 -2.501415
H 3.408536 -2.245670 -1.983903
H 3.737344 -2.230821 -0.267710
H 3.936312 0.276407 -0.322200
H 3.697154 0.250484 -2.062375
H 1.426432 -0.794898 -1.672391
O 1.525683 1.194509 -1.090163
H 2.209986 -1.104919 1.056796
O 1.369271 0.766866 1.111128
C -2.975522 -1.905981 0.006649
O -2.502772 -3.020910 0.121633
C -4.506097 -1.639362 -0.082035
C -5.030743 -1.734514 1.334390
C -5.304557 -0.725249 2.150984
H -5.192613 0.311492 1.858688
H -5.650714 -0.907772 3.160311
H -5.133417 -2.757719 1.685079
C -4.600427 -0.240888 -0.710274
H -4.778404 -0.353549 -1.781510
H -5.400068 0.374272 -0.302869
O -5.130249 -2.611606 -0.914556
H -4.749525 -3.472872 -0.688137
C -1.372594 3.477402 -0.575140
H -1.900313 3.869598 -1.445622
H -1.817904 3.964875 0.297074
O 0.440758 3.319498 1.669493
H 0.900042 2.743712 2.294166
H 0.271098 4.877399 -0.478422
H 0.509588 3.582761 -1.643282

1a-c80, ΔG = 1.9905 kcal/mol, population = 0.22 %

C 0.178818 -4.249091 0.161205
C 1.057808 -3.393274 -0.735425
H 2.098267 -3.727664 -0.692132
C 1.025274 -1.953502 -0.243493
C -0.381735 -1.428156 -0.022551
C -1.459714 -2.295464 0.204419
N -2.709914 -1.853929 0.435841
C -2.895280 -0.542123 0.427214
C -1.884782 0.391265 0.170330
C -0.579955 -0.049044 -0.053930
C 0.571433 0.870908 -0.357237
H 0.613087 1.680376 0.372972
H 0.403811 1.343055 -1.329282
C 1.881204 0.090930 -0.374291
C 2.262864 -0.476375 1.020321
C 3.747048 -0.487461 1.352683
C 4.257157 0.822464 1.966131
C 4.167995 2.069526 1.079693
C 5.068139 2.026252 -0.156512
C 4.979506 3.279190 -1.033929
C 3.627527 3.460161 -1.726416
H 2.815181 3.589702 -1.008729
H 3.388017 2.593045 -2.348232
H 3.633079 4.339036 -2.374317
H 5.197910 4.161381 -0.422745
H 5.764027 3.231637 -1.794599
H 4.834857 1.149072 -0.768974
H 6.102509 1.891234 0.175759
H 3.128726 2.243391 0.788713
H 4.450468 2.939759 1.680487
H 5.299760 0.676114 2.263521
H 3.699131 1.007036 2.889578
H 4.313877 -0.759159 0.458257
H 3.906141 -1.290203 2.076261
H 1.707538 0.043988 1.805832
O 1.785135 -1.845225 0.954199
H 2.681325 0.660955 -0.831186
O 1.699543 -1.104476 -1.159153
C -2.447614 1.736266 0.218986
O -1.881185 2.812254 0.223563
C -3.998412 1.594742 0.306510
C -4.559303 1.856721 -1.071455
C -5.381300 2.851668 -1.376054
H -5.726551 3.552752 -0.626779

H -5.746228 2.983601 -2.386503
H -4.233545 1.159258 -1.837859
C -4.214506 0.121658 0.719242
H -4.427172 0.081043 1.789339
H -5.037532 -0.358204 0.191339
O -4.504898 2.474284 1.294437
H -4.073953 3.331878 1.166479
C -1.280001 -3.786642 0.138872
H -1.842011 -4.242330 0.955372
H -1.760660 -4.121092 -0.785157
O 0.558794 -3.470967 -2.069427
H 1.070940 -2.857451 -2.611619
H 0.243862 -5.290012 -0.156898
H 0.579696 -4.193461 1.174850

1a-c128, ΔG = 2.0212 kcal/mol, population = 0.20 %
C -1.565524 2.817673 0.732887
C -1.831019 2.072511 -0.564614
H -2.903442 1.912343 -0.706734
C -1.174909 0.701029 -0.498672
C 0.276285 0.749746 -0.058112
C 0.783642 1.829144 0.677961
N 2.056208 1.875003 1.114516
C 2.834281 0.848028 0.805797
C 2.418484 -0.251161 0.045372
C 1.097706 -0.322879 -0.398665
C 0.554884 -1.451955 -1.233049
H 0.801285 -2.412643 -0.778637
H 1.047897 -1.438487 -2.209282
C -0.954272 -1.311277 -1.410078
C -1.744104 -1.497336 -0.090135
C -3.087977 -2.181099 -0.274803
C -3.877932 -2.436729 1.012867
C -4.400224 -1.197928 1.753996
C -5.260100 -0.249933 0.913265
C -6.516623 -0.885701 0.318512
C -7.374461 0.117356 -0.451119
H -8.265705 -0.354170 -0.871068
H -6.810363 0.559351 -1.276898
H -7.702350 0.933238 0.198580
H -6.237550 -1.705197 -0.350066
H -7.108769 -1.336027 1.122739
H -4.652612 0.172952 0.108298
H -5.557668 0.596843 1.541320

H -4.987592 -1.542868 2.612063
H -3.557328 -0.635533 2.158565
H -3.249838 -3.015685 1.698151
H -4.720857 -3.086120 0.761896
H -2.887397 -3.147040 -0.748343
H -3.678059 -1.599739 -0.988116
H -1.142047 -2.038778 0.644622
O -1.918435 -0.135056 0.378985
H -1.324176 -1.948159 -2.208699
O -1.256730 0.054936 -1.759787
C 3.535113 -1.176408 -0.112821
O 3.543447 -2.314394 -0.541247
C 4.815671 -0.462791 0.418706
C 5.582969 0.046548 -0.778803
C 6.799983 -0.352641 -1.122186
H 7.345236 -1.081747 -0.536205
H 7.293743 0.052900 -1.995859
H 5.057440 0.785292 -1.377012
C 4.263953 0.711027 1.258175
H 4.299074 0.437881 2.314705
H 4.823777 1.636223 1.128073
O 5.562321 -1.355769 1.224887
H 5.597661 -2.203626 0.758379
C -0.068548 3.032888 0.969730
H 0.118220 3.352540 1.995917
H 0.295211 3.840472 0.327659
O -1.286954 2.830383 -1.643719
H -1.383756 2.306291 -2.449071
H -2.079952 3.778809 0.709403
H -1.999844 2.236453 1.548227

1a-c56, ΔG = 2.0269 kcal/mol, population = 0.20 %
C -0.637646 4.048759 -0.646137
C 0.233660 3.475732 0.459223
H 1.222202 3.943715 0.455191
C 0.437435 1.986843 0.217115
C -0.856556 1.239560 -0.048215
C -1.999711 1.898719 -0.519390
N -3.146165 1.250733 -0.801825
C -3.161333 -0.057682 -0.599205
C -2.079348 -0.786839 -0.092551
C -0.879699 -0.134866 0.187802
C 0.334968 -0.822127 0.752256
H 0.570238 -1.716308 0.173257

H 0.109383 -1.162191 1.766959
C 1.524554 0.131704 0.774301
C 2.005927 0.534687 -0.642568
C 3.505544 0.724304 -0.770674
C 4.271174 -0.598176 -0.787468
C 5.782707 -0.398514 -0.890155
C 6.580169 -1.703162 -0.993769
C 6.404487 -2.674878 0.179302
C 6.797831 -2.088883 1.534940
H 6.726063 -2.840673 2.323747
H 6.152516 -1.254341 1.816950
H 7.826890 -1.719373 1.517700
H 5.368459 -3.023526 0.220189
H 7.011298 -3.563681 -0.017481
H 7.642797 -1.455687 -1.089073
H 6.300077 -2.215635 -1.920297
H 5.999972 0.214186 -1.770802
H 6.125871 0.181404 -0.027818
H 3.926650 -1.207046 -1.630721
H 4.038326 -1.168248 0.116179
H 3.851188 1.354266 0.055021
H 3.700611 1.274196 -1.695275
H 1.649748 -0.183120 -1.386113
O 1.338754 1.802248 -0.869762
H 2.329408 -0.246146 1.398276
O 1.098399 1.394832 1.324956
C -2.459376 -2.192649 0.016986
O -1.767830 -3.169343 0.235500
C -3.993718 -2.279111 -0.230819
C -4.631249 -2.476783 1.126807
C -5.231243 -1.554235 1.867928
H -5.344110 -0.526498 1.545984
H -5.632199 -1.801771 2.842540
H -4.521143 -3.488453 1.507619
C -4.333431 -0.948705 -0.918471
H -4.384322 -1.118982 -1.995626
H -5.281999 -0.517318 -0.605427
O -4.291951 -3.377859 -1.087882
H -3.771353 -4.134267 -0.780205
C -2.020103 3.393495 -0.678865
H -2.542585 3.639131 -1.604445
H -2.636332 3.789088 0.133880
O -0.418810 3.688426 1.709775
H 0.095993 3.236052 2.390254

H -0.739147 5.125155 -0.504583
H -0.120758 3.892963 -1.594761

1a-c233, ΔG = 2.0432 kcal/mol, population = 0.20 %
C -0.817984 3.538248 -0.275094
C -1.286713 2.422529 -1.194184
H -2.378807 2.397247 -1.247142
C -0.830562 1.084340 -0.631490
C 0.642484 1.055688 -0.268443
C 1.354307 2.232957 -0.000019
N 2.651374 2.230142 0.359710
C 3.250075 1.050918 0.438775
C 2.624765 -0.168030 0.151074
C 1.275817 -0.184197 -0.205152
C 0.515674 -1.437008 -0.551609
H 0.660226 -2.195021 0.219470
H 0.926734 -1.855428 -1.474679
C -0.968286 -1.130088 -0.734304
C -1.659383 -0.692281 0.580756
C -3.096606 -1.154324 0.761426
C -4.044854 -0.786809 -0.380101
C -5.481864 -1.267317 -0.154562
C -6.190081 -0.585493 1.018613
C -7.675179 -0.940396 1.142833
C -7.937435 -2.414214 1.454449
H -9.003109 -2.601616 1.602650
H -7.415051 -2.717653 2.366061
H -7.598418 -3.065297 0.646481
H -8.118592 -0.324922 1.931345
H -8.188799 -0.666368 0.214815
H -6.091786 0.499297 0.902003
H -5.688303 -0.839602 1.957700
H -6.060586 -1.081371 -1.065605
H -5.478277 -2.352297 -0.010460
H -3.672781 -1.213464 -1.315091
H -4.041037 0.298012 -0.514174
H -3.457692 -0.731920 1.701869
H -3.080914 -2.241069 0.897537
H -1.064161 -1.011089 1.439608
O -1.616461 0.756391 0.508843
H -1.485338 -1.946926 -1.229574
O -1.098171 0.044758 -1.560217
C 3.585146 -1.252906 0.321204
O 3.400151 -2.454535 0.343937

C 4.991380 -0.609177 0.521363
C 5.727115 -0.702551 -0.794447
C 6.849277 -1.382736 -0.985592
H 7.333505 -1.918489 -0.178836
H 7.325786 -1.410053 -1.957076
H 5.263571 -0.165091 -1.616818
C 4.678451 0.861624 0.875727
H 4.761227 0.990560 1.956741
H 5.349027 1.573837 0.396828
O 5.666151 -1.253141 1.587038
H 5.546652 -2.206882 1.469808
C 0.707685 3.580547 -0.162703
H 1.016529 4.218844 0.666299
H 1.132950 4.026961 -1.066339
O -0.724787 2.632087 -2.488520
H -0.947907 1.865780 -3.032332
H -1.190005 4.493301 -0.647198
H -1.267161 3.374679 0.706141

1a-c148, ΔG = 2.0570 kcal/mol, population = 0.19 %
C -0.370105 -4.090472 0.991861
C 0.268640 -3.683080 -0.325807
H 1.249249 -4.153019 -0.443609
C 0.486813 -2.176698 -0.333032
C -0.749163 -1.389502 0.063861
C -1.772857 -1.969126 0.826608
N -2.859677 -1.281596 1.223878
C -2.939115 -0.013573 0.848348
C -1.984059 0.630637 0.053473
C -0.842713 -0.062339 -0.351327
C 0.232850 0.532412 -1.220776
H 0.554884 1.495634 -0.822295
H -0.183421 0.732621 -2.212071
C 1.415070 -0.424016 -1.332209
C 2.155511 -0.627989 0.013507
C 3.657535 -0.812166 -0.115996
C 4.416104 0.466764 -0.486308
C 4.405734 1.542076 0.602215
C 5.163624 2.818261 0.219218
C 6.654587 2.633459 -0.087590
C 7.458293 2.048355 1.073395
H 7.138961 1.032451 1.314792
H 7.340139 2.654893 1.975602
H 8.523060 2.009526 0.833960

H 6.777909 2.000625 -0.971204
H 7.071851 3.608104 -0.357437
H 5.061402 3.541095 1.035604
H 4.676843 3.269229 -0.652157
H 3.375308 1.820373 0.838630
H 4.822347 1.121487 1.522674
H 4.012174 0.887771 -1.413549
H 5.447621 0.187877 -0.711821
H 3.838853 -1.586024 -0.867178
H 4.034566 -1.198111 0.835257
H 1.916661 0.181229 0.705164
O 1.570788 -1.851731 0.530565
H 2.083702 -0.141925 -2.140392
O 0.918720 -1.748281 -1.615205
C -2.402631 2.008661 -0.179407
O -1.779850 2.936339 -0.659059
C -3.863849 2.151638 0.346395
C -4.786444 2.094451 -0.848168
C -5.589326 3.078016 -1.230684
H -5.646999 4.009850 -0.682408
H -6.226275 2.971601 -2.099320
H -4.751292 1.164174 -1.407777
C -4.055443 0.913415 1.250074
H -3.936881 1.214524 2.292846
H -5.033028 0.445667 1.141155
O -3.989275 3.347250 1.094899
H -3.552418 4.049912 0.591807
C -1.734019 -3.426827 1.191999
H -2.070063 -3.537608 2.223917
H -2.483552 -3.927182 0.571851
O -0.602755 -4.061556 -1.389772
H -0.227936 -3.720970 -2.212081
H -0.476157 -5.175277 1.021690
H 0.311598 -3.807967 1.795979

1a-c93, ΔG = 2.0739 kcal/mol, population = 0.19 %
C 0.902175 4.194726 0.906004
C 0.089533 3.820752 -0.321955
H -0.828437 4.413050 -0.374938
C -0.321949 2.358933 -0.224558
C 0.836732 1.431034 0.091363
C 1.999532 1.899716 0.719157
N 3.028377 1.092047 1.036374
C 2.908454 -0.187998 0.716158

C 1.799592 -0.731492 0.057473
C 0.717423 0.088647 -0.263676
C -0.509951 -0.392739 -0.989625
H -0.905128 -1.290993 -0.512949
H -0.229603 -0.682393 -2.006192
C -1.566900 0.706394 -1.031732
C -2.126708 1.056528 0.374157
C -3.603306 1.416208 0.431658
C -4.537053 0.210863 0.577969
C -4.538156 -0.764227 -0.601238
C -5.598112 -1.863749 -0.491023
C -5.376367 -2.839182 0.666284
C -6.403104 -3.970139 0.689048
H -6.226413 -4.654571 1.521538
H -6.366677 -4.551886 -0.235826
H -7.417503 -3.575468 0.791419
H -4.367717 -3.259586 0.588560
H -5.412113 -2.302032 1.617932
H -5.617225 -2.430666 -1.427651
H -6.587606 -1.402834 -0.390976
H -4.703612 -0.202100 -1.526192
H -3.556126 -1.238607 -0.698844
H -5.555494 0.585014 0.720635
H -4.275950 -0.320804 1.497561
H -3.863810 2.004044 -0.454246
H -3.738747 2.070819 1.295675
H -1.909071 0.250512 1.080559
O -1.344949 2.219138 0.753551
H -2.347096 0.488744 -1.752831
O -0.937100 1.939725 -1.432721
C 2.016006 -2.159056 -0.150963
O 1.234081 -3.015745 -0.515923
C 3.497970 -2.469011 0.222685
C 4.280361 -2.568498 -1.065571
C 4.892446 -3.663808 -1.494616
H 4.884067 -4.581381 -0.919899
H 5.435968 -3.668193 -2.430636
H 4.308934 -1.656151 -1.654401
C 3.939003 -1.236188 1.042553
H 3.898681 -1.484960 2.104854
H 4.949097 -0.901259 0.810449
O 3.558276 -3.646247 1.007421
H 2.982182 -4.302007 0.588233
C 2.181788 3.361896 1.018789

H 2.621132 3.462068 2.012352
H 2.931455 3.736569 0.315736
O 0.892950 4.031593 -1.481421
H 0.398373 3.707193 -2.244846
H 1.151522 5.255409 0.865172
H 0.271632 4.042835 1.783721

1a-c117, ΔG = 2.0890 kcal/mol, population = 0.18 %
C 0.832841 4.192882 0.705175
C -0.009290 3.706238 -0.462262
H -0.958314 4.247988 -0.507802
C -0.338938 2.233548 -0.263837
C 0.879368 1.386130 0.054077
C 2.041105 1.949325 0.600605
N 3.124223 1.214864 0.915683
C 3.060050 -0.085742 0.671758
C 1.954982 -0.722517 0.095419
C 0.816682 0.020934 -0.218448
C -0.414175 -0.564619 -0.856222
H -0.741003 -1.448266 -0.306172
H -0.162498 -0.906603 -1.863961
C -1.527927 0.475928 -0.920343
C -2.055384 0.885550 0.481428
C -3.543567 1.189439 0.567087
C -4.425981 -0.031230 0.843853
C -4.403432 -1.117888 -0.233219
C -5.353200 -2.286740 0.047036
C -6.839217 -1.927967 -0.006917
C -7.745525 -3.141783 0.190953
H -7.565632 -3.895720 -0.580032
H -8.800878 -2.864253 0.147083
H -7.564550 -3.611787 1.161359
H -7.071225 -1.179284 0.755226
H -7.057526 -1.460621 -0.973315
H -5.119497 -2.713443 1.029394
H -5.159769 -3.079157 -0.683402
H -4.653433 -0.675382 -1.204471
H -3.390247 -1.518348 -0.328248
H -5.449537 0.324902 0.978398
H -4.128270 -0.474862 1.800616
H -3.859242 1.693778 -0.351669
H -3.675868 1.906518 1.380471
H -1.780547 0.133903 1.226577
O -1.313000 2.100513 0.763989

H -2.321207 0.174995 -1.596147
O -0.978664 1.712455 -1.418621
C 2.242241 -2.145248 -0.051834
O 1.495089 -3.060272 -0.339941
C 3.753984 -2.354911 0.268807
C 4.485942 -2.480881 -1.046460
C 5.145805 -3.560407 -1.443502
H 5.217663 -4.443478 -0.821129
H 5.649061 -3.584954 -2.401478
H 4.434584 -1.602833 -1.683944
C 4.160226 -1.058482 1.003999
H 4.179733 -1.252260 2.078306
H 5.139066 -0.682606 0.709204
O 3.913320 -3.483271 1.109512
H 3.362284 -4.192682 0.747939
C 2.158200 3.433737 0.807567
H 2.633488 3.615154 1.772725
H 2.856304 3.802810 0.050576
O 0.733266 3.888609 -1.666293
H 0.225656 3.494923 -2.387400
H 1.023673 5.260504 0.592920
H 0.248454 4.061392 1.617446

1a-c68, ΔG = 2.0902 kcal/mol, population = 0.18 %
C -1.702167 2.866260 0.022859
C -1.696073 2.147513 -1.315977
H -2.715934 2.007088 -1.685244
C -1.085996 0.764627 -1.138212
C 0.243216 0.785767 -0.408330
C 0.599182 1.847942 0.434401
N 1.759065 1.874389 1.116673
C 2.576756 0.844915 0.951552
C 2.313281 -0.239609 0.106809
C 1.107809 -0.290962 -0.594201
C 0.734756 -1.401249 -1.540124
H 0.874573 -2.371565 -1.061475
H 1.415450 -1.380154 -2.395840
C -0.706342 -1.238374 -2.015886
C -1.744879 -1.430683 -0.880675
C -3.027546 -2.113048 -1.326007
C -4.027904 -2.420305 -0.207668
C -4.696311 -1.201496 0.429567
C -5.730401 -1.582594 1.488162
C -6.456515 -0.386772 2.112756

C -5.540029 0.563954 2.884255
H -6.113947 1.354817 3.371998
H -4.810541 1.042921 2.228560
H -4.985708 0.026732 3.659050
H -6.976491 0.168363 1.324396
H -7.233352 -0.759618 2.787190
H -5.237878 -2.157003 2.282002
H -6.471303 -2.253741 1.040050
H -3.932383 -0.559676 0.866359
H -5.181723 -0.606843 -0.353286
H -3.521496 -3.003203 0.570348
H -4.804417 -3.074011 -0.617836
H -2.735910 -3.055633 -1.799260
H -3.495979 -1.498832 -2.101480
H -1.298938 -1.977245 -0.045124
O -2.007020 -0.070097 -0.447210
H -0.915045 -1.860801 -2.881510
O -0.915812 0.135967 -2.398953
C 3.433152 -1.173122 0.158493
O 3.519496 -2.303368 -0.281607
C 4.586170 -0.481771 0.949133
C 5.590455 0.024951 -0.058854
C 6.848849 -0.384633 -0.143552
H 7.254240 -1.120990 0.538855
H 7.516994 0.019552 -0.893096
H 5.208334 0.771260 -0.749525
C 3.889111 0.691503 1.673138
H 3.716371 0.412971 2.714560
H 4.470091 1.612765 1.660819
O 5.139419 -1.392315 1.882401
H 5.269585 -2.234077 1.421935
C -0.286125 3.055300 0.572772
H -0.315892 3.347616 1.623430
H 0.210845 3.873082 0.042831
O -0.926274 2.913650 -2.240685
H -0.856159 2.403989 -3.057975
H -2.190231 3.834899 -0.088419
H -2.303888 2.276309 0.716494

1a-c106, ΔG = 2.1003 kcal/mol, population = 0.18 %
C -0.448287 -3.648755 -0.678443
C -1.061052 -2.849972 0.459982
H -2.146866 -2.978683 0.483622
C -0.787059 -1.369860 0.238226

C 0.669536 -1.066578 -0.062668
C 1.531489 -2.045656 -0.575595
N 2.817386 -1.791198 -0.882574
C 3.254742 -0.560092 -0.663441
C 2.473181 0.465415 -0.118371
C 1.133469 0.223577 0.187200
C 0.215421 1.250751 0.794432
H 0.261481 2.183189 0.229944
H 0.566064 1.484487 1.803717
C -1.214820 0.722402 0.848821
C -1.834971 0.520977 -0.557259
C -3.315250 0.844892 -0.675056
C -4.221410 0.105488 0.309586
C -5.711500 0.375986 0.081598
C -6.129541 1.827856 0.322674
C -7.642093 2.060973 0.252680
C -8.246115 1.803272 -1.128526
H -7.754317 2.417302 -1.888225
H -9.311358 2.043597 -1.143711
H -8.139902 0.758851 -1.427862
H -8.138050 1.423640 0.992875
H -7.853051 3.093369 0.547332
H -5.638536 2.483345 -0.403697
H -5.766915 2.136688 1.309382
H -5.973095 0.076478 -0.937904
H -6.291753 -0.269897 0.749216
H -3.960406 0.380027 1.336330
H -4.033688 -0.966398 0.219915
H -3.628235 0.616357 -1.698740
H -3.416935 1.927039 -0.556478
H -1.276816 1.099067 -1.297479
O -1.611259 -0.888075 -0.817465
H -1.838889 1.326327 1.501243
O -1.190043 -0.620358 1.374390
C 3.283398 1.672227 0.007215
O 2.942265 2.811164 0.262347
C 4.762669 1.277254 -0.287243
C 5.470028 1.136941 1.040334
C 6.496464 1.879651 1.431698
H 6.915985 2.649471 0.796208
H 6.958275 1.726524 2.398597
H 5.071126 0.364355 1.691533
C 4.646523 -0.090602 -0.995414
H 4.745887 0.058691 -2.072446

H 5.404766 -0.806274 -0.680584
O 5.356253 2.240065 -1.138965
H 5.109948 3.113719 -0.801719
C 1.069450 -3.465346 -0.751790
H 1.459107 -3.844936 -1.697436
H 1.551591 -4.053414 0.034618
O -0.476362 -3.282938 1.687093
H -0.799504 -2.701307 2.387160
H -0.689432 -4.704361 -0.549861
H -0.916110 -3.319472 -1.607909

1a-c133, ΔG = 2.1009 kcal/mol, population = 0.18 %
C 0.722331 3.827628 -1.474441
C 1.175761 3.573994 -0.046452
H 2.213200 3.890880 0.093266
C 1.117523 2.080590 0.239261
C -0.210173 1.449509 -0.140773
C -1.053877 2.037619 -1.093780
N -2.220034 1.481871 -1.471947
C -2.558976 0.341219 -0.889525
C -1.795118 -0.293213 0.096656
C -0.575496 0.260252 0.487244
C 0.308162 -0.333169 1.551168
H 0.470768 -1.395085 1.360799
H -0.204405 -0.268522 2.515069
C 1.636477 0.413050 1.612370
C 2.484969 0.232794 0.327711
C 3.984083 0.157600 0.551932
C 4.449948 -1.128913 1.245937
C 4.019908 -2.435212 0.567591
C 4.464745 -2.561442 -0.889837
C 4.130394 -3.914387 -1.525767
C 2.630108 -4.181265 -1.657908
H 2.442147 -5.121869 -2.179875
H 2.141780 -3.382372 -2.222956
H 2.142045 -4.243520 -0.683635
H 4.588983 -3.958780 -2.517976
H 4.593841 -4.713513 -0.937211
H 5.546245 -2.396336 -0.942455
H 4.005900 -1.769779 -1.491252
H 4.436983 -3.272000 1.137126
H 2.934453 -2.544721 0.634843
H 4.092225 -1.135216 2.279716
H 5.541747 -1.099883 1.306129

H 4.287651 1.025246 1.143939
H 4.470261 0.255638 -0.421109
H 2.135168 -0.632234 -0.238171
O 2.182158 1.426170 -0.440643
H 2.187807 0.177559 2.518178
O 1.374066 1.831206 1.613236
C -2.477886 -1.508291 0.527086
O -2.071748 -2.419135 1.222788
C -3.902924 -1.496333 -0.106082
C -4.872975 -1.054351 0.964054
C -5.863540 -1.796599 1.439819
H -6.056101 -2.794662 1.066860
H -6.523563 -1.417614 2.209470
H -4.704432 -0.052108 1.347692
C -3.801164 -0.439613 -1.228483
H -3.675765 -0.949959 -2.185490
H -4.678743 0.201704 -1.298580
O -4.201212 -2.773477 -0.640221
H -3.932955 -3.431503 0.017542
C -0.720351 3.370679 -1.704167
H -0.948422 3.333535 -2.770374
H -1.412778 4.097676 -1.269661
O 0.309451 4.281084 0.838917
H 0.552666 4.041320 1.742263
H 0.816096 4.890302 -1.700047
H 1.400923 3.292734 -2.141193

1a-c134, ΔG = 2.1091 kcal/mol, population = 0.18 %
C -0.596003 -3.442272 -1.635247
C -0.858256 -3.426643 -0.138678
H -1.836309 -3.860283 0.088518
C -0.878539 -1.985606 0.350230
C 0.326693 -1.182574 -0.106210
C 1.069757 -1.554328 -1.234565
N 2.123099 -0.841574 -1.677851
C 2.448840 0.239609 -0.986738
C 1.784451 0.661932 0.170602
C 0.682475 -0.055095 0.633813
C -0.084830 0.302758 1.877875
H -0.359688 1.358497 1.864756
H 0.563587 0.163144 2.747405
C -1.326533 -0.573620 2.004978
C -2.364231 -0.313343 0.884632
C -3.806810 -0.412066 1.343362

C -4.831790 -0.355958 0.205839
C -4.710047 0.861122 -0.717661
C -4.868689 2.207743 -0.009957
C -4.815622 3.414329 -0.952744
C -3.452226 3.620379 -1.614594
H -3.187178 2.786529 -2.267305
H -2.665541 3.713590 -0.860555
H -3.441784 4.528485 -2.221116
H -5.585301 3.303286 -1.724116
H -5.075665 4.314262 -0.387497
H -4.089846 2.326712 0.750277
H -5.822976 2.211798 0.527669
H -3.749646 0.824370 -1.237988
H -5.475176 0.782346 -1.496930
H -5.833062 -0.376042 0.647033
H -4.738575 -1.264387 -0.393990
H -3.980969 0.402343 2.052171
H -3.930567 -1.345973 1.899086
H -2.173716 0.652458 0.412673
O -2.078797 -1.356618 -0.082650
H -1.762477 -0.513861 2.998542
O -0.952821 -1.946669 1.767534
C 2.423522 1.875536 0.672216
O 2.049219 2.653904 1.528981
C 3.746674 2.065458 -0.124503
C 4.862174 1.630158 0.800514
C 5.500457 0.467039 0.786346
H 5.286116 -0.309753 0.062946
H 6.269498 0.248403 1.516184
H 5.096487 2.366554 1.564276
C 3.558369 1.184730 -1.368552
H 3.234274 1.819169 -2.195794
H 4.460062 0.663237 -1.682968
O 3.907135 3.432183 -0.493519
H 3.666142 3.968974 0.275753
C 0.758915 -2.821058 -1.982696
H 0.828620 -2.621016 -3.052904
H 1.560786 -3.528453 -1.752052
O 0.179686 -4.155807 0.513704
H 0.055280 -4.055349 1.466139
H -0.638674 -4.469313 -1.999037
H -1.402547 -2.891200 -2.121968

1a-c110, ΔG = 2.1285 kcal/mol, population = 0.17 %

C -0.256905 3.708783 1.277707
C -0.648610 3.435031 -0.164757
H -1.646992 3.828061 -0.376963
C -0.697149 1.931549 -0.394176
C 0.550190 1.212671 0.086787
C 1.383518 1.768399 1.066577
N 2.475305 1.134160 1.535608
C 2.748224 -0.052954 1.016826
C 1.990528 -0.664043 0.010965
C 0.848335 -0.029072 -0.473873
C -0.019540 -0.589376 -1.568453
H -0.280903 -1.626365 -1.353381
H 0.551505 -0.603367 -2.501193
C -1.275496 0.259313 -1.737771
C -2.215048 0.203832 -0.506563
C -3.695448 0.266526 -0.836232
C -4.250411 -1.013464 -1.470998
C -4.126551 -2.280540 -0.612305
C -4.725308 -2.177707 0.793548
C -6.221975 -1.866468 0.822011
C -6.792840 -1.849136 2.238623
H -7.861795 -1.625443 2.236784
H -6.294895 -1.092626 2.851021
H -6.654033 -2.815945 2.729711
H -6.409481 -0.898970 0.347613
H -6.753533 -2.610627 0.219067
H -4.190690 -1.418409 1.374018
H -4.552111 -3.125826 1.313097
H -4.614868 -3.101725 -1.146616
H -3.074326 -2.566687 -0.526746
H -3.753917 -1.192884 -2.429259
H -5.301347 -0.838904 -1.712472
H -3.860333 1.112614 -1.509038
H -4.234191 0.493106 0.086608
H -1.986612 -0.669562 0.106447
O -1.848771 1.391191 0.243765
H -1.789232 0.033496 -2.667801
O -0.895189 1.650388 -1.771637
C 2.597866 -1.948054 -0.330341
O 2.159312 -2.860994 -1.004136
C 3.988879 -2.000447 0.365035
C 5.010059 -1.719170 -0.715745
C 5.619756 -0.564880 -0.952970
H 5.449300 0.320786 -0.353586

H 6.317314 -0.465356 -1.774772
H 5.194101 -2.571873 -1.363419
C 3.895361 -0.924857 1.457446
H 3.647977 -1.411629 2.402841
H 4.814603 -0.361323 1.603408
O 4.206669 -3.286325 0.937514
H 3.901834 -3.943560 0.294733
C 1.128729 3.147876 1.607735
H 1.293367 3.136100 2.686113
H 1.901089 3.799179 1.188264
O 0.321431 4.034698 -1.021451
H 0.112406 3.776540 -1.928263
H -0.276178 4.783668 1.459889
H -1.013026 3.255927 1.921440

1a-c153, ΔG = 2.1511 kcal/mol, population = 0.16 %
C 0.031221 3.910201 -1.431208
C 0.506958 3.709292 -0.001896
H 1.486642 4.171377 0.149744
C 0.662917 2.219971 0.270753
C -0.555074 1.406997 -0.129164
C -1.465366 1.875698 -1.087025
N -2.529846 1.156551 -1.488684
C -2.701259 -0.029959 -0.924674
C -1.864862 -0.555825 0.066722
C -0.746679 0.168737 0.480993
C 0.204728 -0.298785 1.549988
H 0.524779 -1.322985 1.353356
H -0.321988 -0.320406 2.508290
C 1.407940 0.633811 1.635343
C 2.294742 0.598075 0.365322
C 3.779781 0.769667 0.628069
C 4.443402 -0.447758 1.283057
C 4.370460 -1.756363 0.485239
C 4.981551 -1.672448 -0.913545
C 4.956836 -2.995564 -1.686534
C 5.831701 -4.092840 -1.079326
H 5.487257 -4.385077 -0.085563
H 6.867905 -3.756072 -0.985718
H 5.827773 -4.988591 -1.704113
H 3.922447 -3.349079 -1.757587
H 5.285408 -2.806794 -2.712813
H 6.016882 -1.320040 -0.832920
H 4.447798 -0.919657 -1.499988

H 4.885412 -2.526821 1.064074
H 3.331802 -2.093314 0.401478
H 4.005523 -0.613377 2.272093
H 5.494498 -0.201017 1.459024
H 3.914482 1.647965 1.265503
H 4.265059 0.997838 -0.322973
H 2.100356 -0.308216 -0.210580
O 1.817442 1.732431 -0.404197
H 1.975678 0.472093 2.547147
O 0.940039 1.998276 1.645060
C -2.361666 -1.867070 0.469323
O -1.832913 -2.716642 1.160188
C -3.760963 -2.063091 -0.190720
C -4.807026 -1.794210 0.865466
C -5.684797 -2.686831 1.302537
H -5.716537 -3.693146 0.904443
H -6.410420 -2.429114 2.063175
H -4.798746 -0.786638 1.271281
C -3.804579 -0.984703 -1.295931
H -3.587890 -1.454015 -2.257610
H -4.768527 -0.483505 -1.372751
O -3.849378 -3.361885 -0.748627
H -3.496384 -3.981614 -0.093664
C -1.328224 3.251827 -1.676216
H -1.544979 3.197980 -2.743971
H -2.120293 3.863674 -1.234530
O -0.459095 4.274321 0.882506
H -0.192160 4.059547 1.785466
H -0.028514 4.977680 -1.645239
H 0.784408 3.486483 -2.097757

1a-c83, ΔG = 2.1549 kcal/mol, population = 0.16 %
C -0.632572 -4.334758 0.511218
C 0.269360 -3.790943 -0.584256
H 1.219047 -4.332928 -0.608685
C 0.588959 -2.331667 -0.293304
C -0.644379 -1.497134 0.004152
C -1.830873 -2.084173 0.466318
N -2.925862 -1.363391 0.771518
C -2.848982 -0.051767 0.601449
C -1.719791 0.610248 0.106228
C -0.570147 -0.119863 -0.197145
C 0.687392 0.493739 -0.751285
H 0.982846 1.357669 -0.154313

H 0.485263 0.870785 -1.757714
C 1.804903 -0.542380 -0.795026
C 2.241578 -1.013652 0.619953
C 3.727609 -1.284680 0.798240
C 4.541845 -0.045513 1.182936
C 4.515771 1.096106 0.166941
C 5.408704 2.269309 0.569952
C 5.424293 3.419621 -0.441463
C 4.072488 4.112670 -0.618674
H 4.156738 4.971787 -1.287493
H 3.690741 4.472565 0.340833
H 3.323138 3.440993 -1.041712
H 6.164138 4.158550 -0.119923
H 5.770337 3.041444 -1.409458
H 6.430790 1.902895 0.710869
H 5.081434 2.650985 1.544074
H 4.832915 0.723329 -0.813875
H 3.491168 1.455284 0.045492
H 5.578698 -0.354305 1.344800
H 4.179893 0.328402 2.146979
H 4.121300 -1.738434 -0.116607
H 3.827484 -2.030989 1.589578
H 1.896496 -0.305063 1.378293
O 1.508486 -2.254236 0.788905
H 2.639039 -0.212229 -1.404369
O 1.289739 -1.753633 -1.383529
C -1.996851 2.040438 0.026330
O -1.235916 2.966279 -0.179548
C -3.519303 2.240192 0.298286
C -4.198605 2.444157 -1.035408
C -4.847331 3.544049 -1.392849
H -4.947712 4.388048 -0.722144
H -5.312181 3.625007 -2.366987
H -4.118412 1.605985 -1.721735
C -3.959217 0.906463 0.942000
H -4.022365 1.039516 2.023853
H -4.926197 0.552995 0.586589
O -3.707736 3.320196 1.194616
H -3.137825 4.044443 0.897357
C -1.960455 -3.577133 0.585265
H -2.486640 -3.809432 1.512281
H -2.617546 -3.901153 -0.227170
O -0.409354 -3.907449 -1.833327
H 0.133409 -3.474013 -2.504353

H -0.818416 -5.394354 0.333501
H -0.095765 -4.253138 1.457884

1a-c99, ΔG = 2.1624 kcal/mol, population = 0.16 %
C 1.617198 2.845352 -0.370928
C 1.655413 2.225951 1.016128
H 2.686901 2.112672 1.361691
C 1.042797 0.834095 0.959994
C -0.309304 0.802185 0.272224
C -0.694263 1.800716 -0.633293
N -1.876643 1.779810 -1.276221
C -2.688608 0.767711 -1.008141
C -2.397078 -0.251480 -0.094217
C -1.168441 -0.254722 0.566983
C -0.766216 -1.291876 1.581304
H -0.921301 -2.295028 1.181524
H -1.418991 -1.204415 2.454387
C 0.689146 -1.097009 1.995634
C 1.689437 -1.375503 0.843708
C 2.980465 -2.035008 1.297256
C 3.955529 -2.411122 0.177942
C 4.641129 -1.238123 -0.524094
C 5.694312 -1.697040 -1.532690
C 6.330059 -0.560239 -2.338991
C 7.113432 0.442108 -1.490622
H 7.602779 1.190926 -2.117212
H 7.888140 -0.063434 -0.907166
H 6.464753 0.971546 -0.790431
H 6.998338 -0.991840 -3.090376
H 5.545892 -0.033426 -2.893603
H 5.231699 -2.406407 -2.227751
H 6.481688 -2.251985 -1.008654
H 3.895878 -0.622118 -1.031384
H 5.101561 -0.596280 0.232881
H 3.428197 -3.022121 -0.563527
H 4.727316 -3.057622 0.608164
H 2.696242 -2.947272 1.830711
H 3.468593 -1.379501 2.025488
H 1.214347 -1.976687 0.063471
O 1.944332 -0.051058 0.306921
H 0.927205 -1.653816 2.897665
O 0.910628 0.301255 2.268761
C -3.520021 -1.180801 -0.034823
O -3.593000 -2.273950 0.492670

C -4.699308 -0.545584 -0.833779
C -5.664487 0.042725 0.168105
C -6.918918 -0.354780 0.332037
H -7.349802 -1.141208 -0.274723
H -7.558343 0.109308 1.071864
H -5.256066 0.839754 0.782812
C -4.025456 0.564636 -1.670533
H -3.889031 0.204487 -2.692151
H -4.603981 1.486648 -1.710634
O -5.289112 -1.521759 -1.673483
H -5.400703 -2.326387 -1.146562
C 0.184359 2.994134 -0.888187
H 0.180579 3.208046 -1.958010
H -0.297355 3.849153 -0.404995
O 0.914013 3.057783 1.906693
H 0.871514 2.609353 2.760949
H 2.106377 3.819519 -0.345926
H 2.198081 2.206716 -1.038630

1a-c122, ΔG = 2.1950 kcal/mol, population = 0.15 %
C 0.015290 3.763245 1.094961
C -0.529185 3.336991 -0.258147
H -1.551362 3.700306 -0.397824
C -0.578372 1.817334 -0.323434
C 0.720995 1.155890 0.096773
C 1.648404 1.815008 0.914015
N 2.791376 1.235088 1.328681
C 3.020769 -0.001038 0.913913
C 2.166574 -0.717423 0.067014
C 0.970706 -0.139510 -0.355320
C -0.003683 -0.814608 -1.283089
H -0.237181 -1.818342 -0.925177
H 0.469326 -0.939163 -2.261288
C -1.275797 0.016011 -1.421268
C -2.098902 0.095979 -0.112229
C -3.601886 0.148528 -0.311740
C -4.191951 -1.208389 -0.696333
C -5.694817 -1.163481 -0.989802
C -6.572786 -0.696149 0.178588
C -6.430190 -1.513029 1.467841
C -6.777375 -2.992763 1.306559
H -6.091269 -3.496253 0.622594
H -7.788958 -3.115865 0.910095
H -6.728139 -3.514440 2.264665

H -5.411182 -1.419228 1.855500
H -7.081046 -1.072117 2.228612
H -7.618652 -0.727353 -0.144733
H -6.358010 0.353303 0.399734
H -5.868261 -0.499273 -1.842540
H -6.016013 -2.157682 -1.311795
H -3.988719 -1.927402 0.102979
H -3.679978 -1.593028 -1.583705
H -3.830714 0.895111 -1.078961
H -4.047122 0.503405 0.619903
H -1.834878 -0.725091 0.559225
O -1.650575 1.340840 0.483830
H -1.872560 -0.305535 -2.270227
O -0.911789 1.394718 -1.637095
C 2.753125 -2.027374 -0.204621
O 2.258716 -3.007248 -0.729204
C 4.212617 -1.999627 0.333561
C 5.102530 -1.820226 -0.877347
C 5.665501 -0.692137 -1.290683
H 5.550989 0.246810 -0.763139
H 6.266361 -0.670396 -2.190899
H 5.225729 -2.731098 -1.456567
C 4.219744 -0.820040 1.316618
H 4.078782 -1.209558 2.326752
H 5.141522 -0.242032 1.308144
O 4.516565 -3.219690 1.002239
H 4.157854 -3.940621 0.464104
C 1.437239 3.245632 1.324800
H 1.722951 3.354752 2.371953
H 2.147676 3.847422 0.750483
O 0.329846 3.847442 -1.276086
H 0.025833 3.493061 -2.121543
H -0.000779 4.851288 1.164885
H -0.657511 3.377720 1.863126

1a-c81, ΔG = 2.2095 kcal/mol, population = 0.15 %
C -0.543751 3.358205 1.287757
C -0.822601 3.105710 -0.184856
H -1.851133 3.378931 -0.437505
C -0.664365 1.620897 -0.479097
C 0.634181 1.039917 0.047309
C 1.336187 1.647903 1.096136
N 2.469919 1.129490 1.606076
C 2.916670 0.009540 1.058627

C 2.298927 -0.640729 -0.016035
C 1.114480 -0.128638 -0.543036
C 0.381388 -0.740179 -1.707517
H 0.230881 -1.808105 -1.543004
H 1.002793 -0.651229 -2.603025
C -0.955217 -0.038105 -1.926933
C -1.957278 -0.265330 -0.769008
C -3.405963 -0.359561 -1.206574
C -4.381826 -0.501963 -0.040869
C -5.833692 -0.624648 -0.501310
C -6.850598 -0.688062 0.643845
C -6.666822 -1.857859 1.618145
C -6.740850 -3.233430 0.956038
H -7.680718 -3.355236 0.410376
H -6.680477 -4.031256 1.699310
H -5.925120 -3.383809 0.245782
H -5.711492 -1.756958 2.141172
H -7.439803 -1.788012 2.389247
H -7.857108 -0.742327 0.215281
H -6.806806 0.250050 1.207378
H -6.077558 0.233463 -1.136023
H -5.935670 -1.508728 -1.138598
H -4.281723 0.361037 0.623719
H -4.100678 -1.380983 0.547118
H -3.498404 -1.226612 -1.869075
H -3.650354 0.526563 -1.800761
H -1.679542 -1.147705 -0.186268
O -1.770757 0.908971 0.064675
H -1.380525 -0.278806 -2.897423
O -0.745951 1.387948 -1.877022
C 3.078450 -1.823797 -0.370686
O 2.797828 -2.744919 -1.114062
C 4.419524 -1.747516 0.416138
C 5.469683 -1.311559 -0.581705
C 5.988109 -0.097535 -0.715090
H 5.707610 0.732571 -0.078611
H 6.720658 0.110037 -1.484659
H 5.766786 -2.104041 -1.263183
C 4.133096 -0.738785 1.537748
H 3.891176 -1.292330 2.447130
H 4.966996 -0.077546 1.763907
O 4.745384 -3.023969 0.959369
H 4.589035 -3.682044 0.266346
C 0.883142 2.960605 1.672079

H 0.995240 2.923760 2.756582
H 1.586349 3.720158 1.318098
O 0.104852 3.863977 -0.959281
H -0.020955 3.623745 -1.886195
H -0.709153 4.412516 1.511353
H -1.267744 2.784876 1.869507

1a-c143, ΔG = 2.2327 kcal/mol, population = 0.14 %
C -0.966009 3.422723 0.749126
C -1.674000 2.331142 -0.036304
H -2.737110 2.298514 0.218291
C -1.079162 0.979628 0.333681
C 0.436463 0.954128 0.277306
C 1.195997 2.128145 0.368757
N 2.542180 2.124333 0.350546
C 3.137453 0.947538 0.225136
C 2.454939 -0.267609 0.093183
C 1.060412 -0.282887 0.129616
C 0.232913 -1.530321 -0.025966
H 0.577956 -2.304698 0.660642
H 0.381126 -1.926713 -1.034417
C -1.247838 -1.231410 0.199565
C -1.571431 -0.842138 1.662390
C -2.910795 -1.299488 2.229593
C -4.180349 -0.766068 1.554966
C -4.548215 -1.397550 0.211522
C -5.917360 -0.947798 -0.297481
C -6.284889 -1.551619 -1.652124
C -7.650431 -1.094935 -2.162423
H -7.886249 -1.540913 -3.131158
H -7.681449 -0.008322 -2.279081
H -8.443604 -1.375173 -1.464170
H -5.513437 -1.286584 -2.382922
H -6.269306 -2.643910 -1.573837
H -5.931184 0.145667 -0.375570
H -6.686362 -1.211927 0.437676
H -3.801095 -1.140554 -0.540994
H -4.539699 -2.489369 0.309106
H -4.092314 0.317147 1.437768
H -5.011210 -0.931006 2.248131
H -2.922752 -0.995279 3.279763
H -2.913487 -2.394676 2.231044
H -0.774551 -1.199811 2.318829
O -1.520987 0.606639 1.634656

H -1.871433 -2.038828 -0.168676
O -1.589895 -0.031166 -0.522340
C 3.423443 -1.349144 -0.051413
O 3.253713 -2.552385 -0.007918
C 4.825542 -0.697961 -0.254046
C 5.132465 -0.730844 -1.733151
C 6.149429 -1.383886 -2.278768
H 6.855441 -1.940859 -1.675787
H 6.313644 -1.366159 -3.348493
H 4.441118 -0.170241 -2.355840
C 4.630810 0.755537 0.232827
H 5.016118 0.841934 1.250769
H 5.140656 1.491437 -0.387399
O 5.788817 -1.373306 0.533235
H 5.637303 -2.323177 0.421136
C 0.530997 3.474578 0.433097
H 1.058204 4.090016 1.163461
H 0.688561 3.952227 -0.538283
O -1.497847 2.584605 -1.428901
H -1.873739 1.838020 -1.912708
H -1.426923 4.385329 0.525725
H -1.122810 3.226056 1.811240

1a-c136, ΔG = 2.2427 kcal/mol, population = 0.14 %
C 1.158963 3.149511 0.262943
C 1.506123 2.025799 1.225220
H 2.590371 1.909009 1.308666
C 0.950818 0.714659 0.688352
C -0.508281 0.800442 0.281273
C -1.107096 2.024006 -0.045178
N -2.387458 2.119016 -0.452557
C -3.080019 0.992680 -0.519557
C -2.571893 -0.264975 -0.172993
C -1.243378 -0.383575 0.232445
C -0.604756 -1.684181 0.642296
H -0.793086 -2.453587 -0.107788
H -1.075373 -2.032996 1.565846
C 0.894674 -1.499567 0.859482
C 1.654537 -1.163682 -0.447790
C 3.051938 -1.748806 -0.576416
C 3.992411 -1.439476 0.587210
C 5.410922 -1.985172 0.384976
C 6.153111 -1.443165 -0.841836
C 6.295186 0.078997 -0.873229

C 7.134893 0.567922 -2.051952
H 8.146627 0.155873 -2.011591
H 7.217599 1.656923 -2.060479
H 6.690329 0.260021 -3.002163
H 5.305723 0.542081 -0.921204
H 6.745981 0.417379 0.066165
H 5.655108 -1.773937 -1.759019
H 7.151808 -1.891877 -0.867272
H 5.998603 -1.756655 1.280073
H 5.367770 -3.077467 0.317643
H 3.587357 -1.862573 1.510099
H 4.028040 -0.359701 0.740569
H 3.473100 -1.370033 -1.510629
H 2.951696 -2.832239 -0.701255
H 1.056385 -1.456231 -1.313924
O 1.735528 0.284785 -0.418734
H 1.326765 -2.341444 1.392574
O 1.103527 -0.314624 1.654205
C -3.619513 -1.267431 -0.348421
O -3.543803 -2.481537 -0.335816
C -4.952977 -0.504805 -0.599364
C -5.726594 -0.569008 0.699241
C -5.842983 0.392308 1.606376
H -5.388222 1.368072 1.488571
H -6.404343 0.228389 2.517402
H -6.180982 -1.539441 0.878946
C -4.500704 0.903022 -1.012519
H -4.516622 0.965910 -2.102326
H -5.128706 1.702246 -0.624523
O -5.686533 -1.127995 -1.649880
H -5.640676 -2.084671 -1.506346
C -0.353845 3.316750 0.101584
H -0.582548 3.953442 -0.754237
H -0.765717 3.825222 0.978283
O 0.932316 2.321368 2.497368
H 1.078213 1.556914 3.069323
H 1.600001 4.080273 0.620959
H 1.620282 2.920616 -0.699363

1a-c149, ΔG = 2.2672 kcal/mol, population = 0.13 %
C 1.318285 -3.053682 0.608690
C 1.697625 -2.161383 -0.561319
H 2.783146 -2.042805 -0.622323
C 1.102732 -0.776891 -0.348911

C -0.372990 -0.803617 0.003599
C -0.974431 -1.941685 0.558670
N -2.272413 -1.975885 0.913627
C -2.981127 -0.876105 0.704927
C -2.468565 0.290010 0.124351
C -1.121634 0.346949 -0.235820
C -0.479049 1.542230 -0.887919
H -0.708136 2.449957 -0.328006
H -0.913314 1.676820 -1.882610
C 1.030396 1.345259 -1.000894
C 1.739263 1.318075 0.375260
C 3.127736 1.935715 0.419701
C 4.109226 1.390660 -0.617324
C 5.516798 1.983638 -0.495922
C 6.228416 1.708673 0.835679
C 6.332402 0.229499 1.224093
C 7.077330 -0.630265 0.203088
H 7.186625 -1.656926 0.559025
H 8.078625 -0.233426 0.014087
H 6.551677 -0.668340 -0.753143
H 6.841751 0.161938 2.189982
H 5.331452 -0.181889 1.384222
H 5.721786 2.248423 1.640912
H 7.237254 2.131185 0.779558
H 6.125803 1.596974 -1.317584
H 5.463808 3.067218 -0.645084
H 3.733514 1.596055 -1.623078
H 4.153940 0.303785 -0.528529
H 3.518434 1.776776 1.427480
H 3.017497 3.018621 0.299278
H 1.105802 1.786297 1.132085
O 1.829702 -0.099820 0.670777
H 1.474199 2.059684 -1.688033
O 1.287094 0.020331 -1.508600
C -3.528531 1.288300 0.034388
O -3.456423 2.471977 -0.234535
C -4.871537 0.582474 0.395675
C -5.593172 0.289526 -0.898562
C -6.769295 0.799012 -1.238706
H -7.311852 1.464795 -0.579477
H -7.231950 0.550138 -2.185040
H -5.070228 -0.383491 -1.572012
C -4.428528 -0.723707 1.091228
H -4.517991 -0.597025 2.171942

H -5.021265 -1.590249 0.801250
O -5.618135 1.397510 1.281018
H -5.589756 2.301122 0.934180
C -0.198270 -3.217927 0.731129
H -0.462526 -3.660182 1.692721
H -0.558110 -3.912622 -0.033546
O 1.185880 -2.740540 -1.760066
H 1.355626 -2.120925 -2.481147
H 1.789403 -4.029456 0.486889
H 1.726115 -2.606415 1.516858

1a-c70, ΔG = 2.4247 kcal/mol, population = 0.10 %
C 0.198810 -3.779714 0.526147
C 0.977350 -2.932042 -0.466131
H 2.047440 -3.149987 -0.406095
C 0.795792 -1.460763 -0.120856
C -0.654828 -1.065552 0.082366
C -1.633201 -2.011715 0.412914
N -2.920465 -1.678775 0.629621
C -3.238608 -0.400093 0.501170
C -2.331600 0.603443 0.139771
C -0.992681 0.279014 -0.070939
C 0.055683 1.276278 -0.487876
H 0.033684 2.148750 0.166662
H -0.182375 1.638658 -1.491967
C 1.440138 0.634216 -0.480750
C 1.918151 0.249659 0.941666
C 3.406546 0.419167 1.199752
C 4.319799 -0.311509 0.216414
C 5.807629 -0.185426 0.562214
C 6.354847 1.245740 0.593586
C 6.207752 2.007530 -0.724116
C 6.839210 3.397607 -0.675610
H 6.378056 4.009069 0.104673
H 6.721191 3.924482 -1.624959
H 7.908736 3.335723 -0.457978
H 6.665555 1.422664 -1.529364
H 5.148918 2.103062 -0.981267
H 7.416146 1.205474 0.860588
H 5.867967 1.814926 1.392243
H 5.984903 -0.652539 1.536766
H 6.383107 -0.766931 -0.165561
H 4.146444 0.056701 -0.797763
H 4.048473 -1.368970 0.203627

H 3.613597 0.073595 2.217391
H 3.612670 1.493199 1.192300
H 1.352310 0.808928 1.690315
O 1.551097 -1.149709 1.045400
H 2.162945 1.241980 -1.017311
O 1.359480 -0.646367 -1.137801
C -3.037038 1.880720 0.070297
O -2.594588 3.010507 -0.016918
C -4.559536 1.570722 0.152184
C -5.084446 1.673027 -1.263895
C -5.326744 0.668068 -2.095518
H -5.182989 -0.368535 -1.816863
H -5.677233 0.853984 -3.102704
H -5.217620 2.698026 -1.598635
C -4.614341 0.159090 0.755266
H -4.784536 0.248006 1.830021
H -5.402408 -0.467836 0.343302
O -5.212241 2.511252 0.998466
H -4.848991 3.385112 0.792320
C -1.300678 -3.476196 0.481623
H -1.808340 -3.910056 1.344294
H -1.746586 -3.943003 -0.401549
O 0.483174 -3.196073 -1.777924
H 0.917179 -2.581473 -2.383499
H 0.371325 -4.835226 0.313771
H 0.597064 -3.578950 1.522312

1a-c76, ΔG = 2.4649 kcal/mol, population = 0.10 %
C -1.125831 -3.037249 -0.789539
C -1.631031 -2.074250 0.271853
H -2.717606 -1.965553 0.211290
C -1.025067 -0.698853 0.030118
C 0.478451 -0.730320 -0.170164
C 1.141729 -1.895665 -0.574604
N 2.472094 -1.938159 -0.782807
C 3.147260 -0.818771 -0.573257
C 2.568003 0.379391 -0.138206
C 1.190882 0.445049 0.066092
C 0.475292 1.673754 0.560978
H 0.746009 2.540947 -0.043153
H 0.813126 1.890706 1.578315
C -1.036355 1.461024 0.549988
C -1.622774 1.338593 -0.877133
C -3.017636 1.914507 -1.063981

C -4.068380 1.393283 -0.085905
C -5.468451 1.918793 -0.398907
C -6.529082 1.501345 0.625705
C -6.696627 -0.011708 0.811309
C -7.070209 -0.755811 -0.470418
H -6.276105 -0.695368 -1.217517
H -7.976525 -0.336785 -0.916339
H -7.253990 -1.813741 -0.271333
H -5.776880 -0.437505 1.222897
H -7.470716 -0.182825 1.565314
H -7.492589 1.929124 0.328448
H -6.279687 1.948675 1.593882
H -5.440047 3.012441 -0.445440
H -5.762857 1.582454 -1.398205
H -3.801634 1.674019 0.937800
H -4.064894 0.302657 -0.115049
H -3.332050 1.694919 -2.088925
H -2.941121 3.004410 -0.991717
H -0.940482 1.783246 -1.604885
O -1.652116 -0.095488 -1.097567
H -1.545969 2.209807 1.149590
O -1.327312 0.166590 1.113961
C 3.613972 1.388252 0.010139
O 3.518147 2.589306 0.177741
C 4.981197 0.654940 -0.106333
C 5.500074 0.509583 1.308222
C 5.427727 -0.571643 2.073701
H 4.987965 -1.500572 1.732469
H 5.807523 -0.559821 3.087315
H 5.925677 1.427089 1.705032
C 4.629556 -0.669916 -0.799576
H 4.832663 -0.567117 -1.867338
H 5.194874 -1.524387 -0.433196
O 5.886914 1.417184 -0.897461
H 5.797333 2.342953 -0.627362
C 0.396150 -3.190622 -0.741005
H 0.762491 -3.692363 -1.637780
H 0.676872 -3.828713 0.102032
O -1.245762 -2.566239 1.554137
H -1.489591 -1.899648 2.209095
H -1.602531 -4.008442 -0.653383
H -1.437712 -2.655366 -1.763321

1a-c48, ΔG = 2.4831 kcal/mol, population = 0.09 %

C 1.771182 2.579506 -0.570741
C 2.063746 1.527852 0.485454
H 3.116410 1.237809 0.462791
C 1.240777 0.281646 0.193230
C -0.228297 0.574065 -0.042969
C -0.666348 1.841049 -0.452107
N -1.959603 2.112536 -0.708155
C -2.824589 1.122108 -0.545511
C -2.476855 -0.160941 -0.106742
C -1.138382 -0.463307 0.148044
C -0.660157 -1.803332 0.641536
H -1.068571 -2.604011 0.023472
H -1.048079 -1.965778 1.651074
C 0.865607 -1.857436 0.655141
C 1.482973 -1.803645 -0.765282
C 2.729794 -2.656717 -0.961379
C 3.911001 -2.340045 -0.033170
C 4.811473 -1.212909 -0.544150
C 5.878930 -0.793903 0.465357
C 6.804342 0.321317 -0.031325
C 6.096287 1.657119 -0.264040
H 5.339669 1.584384 -1.047696
H 6.805439 2.431965 -0.562960
H 5.597580 1.996991 0.648006
H 7.606443 0.466772 0.698485
H 7.289326 -0.001091 -0.959094
H 6.482508 -1.667845 0.733619
H 5.388073 -0.465849 1.389609
H 5.298004 -1.536976 -1.471725
H 4.189355 -0.358809 -0.807121
H 4.513185 -3.244458 0.092505
H 3.540534 -2.081276 0.962604
H 3.046835 -2.569010 -2.004728
H 2.412226 -3.695281 -0.828269
H 0.733287 -2.087355 -1.508047
O 1.782742 -0.394967 -0.936724
H 1.230483 -2.697370 1.239480
O 1.364495 -0.646674 1.258689
C -3.678002 -0.984851 -0.027935
O -3.783662 -2.184585 0.140242
C -4.907336 -0.048797 -0.241750
C -5.517877 0.222093 1.112775
C -6.749186 -0.118499 1.468004
H -7.421436 -0.614813 0.779626

H -7.126093 0.105611 2.457581
H -4.862902 0.728595 1.815971
C -4.296443 1.237539 -0.840286
H -4.464966 1.241289 -1.918940
H -4.723629 2.150645 -0.427742
O -5.810775 -0.647635 -1.154099
H -5.912309 -1.574720 -0.893804
C 0.296338 2.990019 -0.571159
H 0.053273 3.557221 -1.470759
H 0.097906 3.657119 0.272779
O 1.718337 2.058017 1.764360
H 1.822523 1.349980 2.412833
H 2.401109 3.452170 -0.395862
H 2.050988 2.166133 -1.541308

1a-c112, ΔG = 2.4981 kcal/mol, population = 0.09 %
C 1.362406 2.879773 -1.396376
C 1.427645 2.853126 0.121681
H 2.446527 3.046054 0.469439
C 1.037320 1.467414 0.614366
C -0.259393 0.960432 0.009983
C -0.745240 1.458144 -1.206222
N -1.873481 0.997641 -1.780212
C -2.530074 0.047052 -1.133648
C -2.134744 -0.480742 0.101160
C -0.960317 -0.028226 0.700025
C -0.458553 -0.516638 2.032017
H -0.441356 -1.607266 2.055371
H -1.158859 -0.202476 2.811071
C 0.929265 0.047983 2.319330
C 2.012921 -0.484447 1.350049
C 3.376279 -0.677915 1.991477
C 4.498545 -0.958348 0.989719
C 4.316972 -2.249210 0.190530
C 5.462634 -2.535881 -0.786290
C 5.684819 -1.462380 -1.859450
C 4.452826 -1.180249 -2.719708
H 3.639726 -0.751869 -2.130451
H 4.079570 -2.098179 -3.182460
H 4.686682 -0.474484 -3.519625
H 6.019164 -0.532762 -1.390144
H 6.507884 -1.786002 -2.503524
H 5.267101 -3.492733 -1.282072
H 6.390861 -2.666635 -0.219782

H 4.226161 -3.090587 0.885528
H 3.375554 -2.212848 -0.363471
H 5.447337 -1.005668 1.533330
H 4.574094 -0.107572 0.310278
H 3.293655 -1.505265 2.703927
H 3.614300 0.221233 2.567291
H 1.673394 -1.404268 0.870926
O 2.091596 0.553493 0.338712
H 1.206210 -0.078195 3.362553
O 0.925825 1.461170 2.030072
C -3.102733 -1.490793 0.520520
O -3.035484 -2.305451 1.421505
C -4.323566 -1.387758 -0.438815
C -5.404641 -0.663612 0.333463
C -5.736565 0.616602 0.227540
H -5.254356 1.290409 -0.469793
H -6.512745 1.041196 0.851374
H -5.900093 -1.292505 1.067968
C -3.775235 -0.623707 -1.652810
H -3.509581 -1.347313 -2.425964
H -4.479123 0.083235 -2.087224
O -4.764623 -2.688225 -0.817738
H -4.762221 -3.237835 -0.020323
C -0.049196 2.588410 -1.912272
H -0.032104 2.374100 -2.981854
H -0.679289 3.474070 -1.788933
O 0.526371 3.832640 0.634484
H 0.506562 3.738889 1.595492
H 1.696032 3.853829 -1.755465
H 2.065737 2.135504 -1.774042

1a-c59, ΔG = 2.6261 kcal/mol, population = 0.07 %
C 0.112985 -4.233152 -0.126681
C -0.833619 -3.409416 0.730430
H -1.854808 -3.794756 0.660559
C -0.855072 -1.976579 0.218108
C 0.531464 -1.387029 0.033118
C 1.656753 -2.204124 -0.146199
N 2.891126 -1.705554 -0.344645
C 3.012656 -0.386259 -0.350449
C 1.950316 0.500301 -0.141433
C 0.661522 0.000166 0.047547
C -0.542813 0.867159 0.297521
H -0.600502 1.660306 -0.449258

H -0.427967 1.364219 1.264773
C -1.814221 0.024957 0.290314
C -2.128100 -0.581817 -1.104568
C -3.600159 -0.680657 -1.474002
C -4.168769 0.587908 -2.121433
C -4.177104 1.850976 -1.254317
C -5.087623 1.771244 -0.029178
C -5.050204 3.034885 0.829143
C -5.964902 2.953947 2.049635
H -5.918738 3.867681 2.645898
H -5.681740 2.118662 2.695545
H -7.005202 2.802611 1.749907
H -4.020191 3.216192 1.154473
H -5.332133 3.895794 0.214071
H -4.816020 0.912873 0.593688
H -6.115492 1.585446 -0.360481
H -3.158121 2.099285 -0.939535
H -4.501409 2.691706 -1.875186
H -5.193126 0.376292 -2.441959
H -3.599428 0.791052 -3.034039
H -4.173327 -0.971527 -0.589647
H -3.694665 -1.502031 -2.187903
H -1.580862 -0.043873 -1.883819
O -1.580003 -1.922101 -1.004629
H -2.653775 0.562749 0.715761
O -1.598302 -1.148707 1.099252
C 2.448902 1.870324 -0.192530
O 1.831300 2.917037 -0.232204
C 4.006706 1.803370 -0.227032
C 4.508469 2.111558 1.163987
C 5.270812 3.149614 1.479608
H 5.605720 3.856782 0.731336
H 5.595875 3.312828 2.499048
H 4.191995 1.409477 1.930089
C 4.307319 0.336891 -0.609549
H 4.557554 0.291919 -1.671298
H 5.134386 -0.095023 -0.047628
O 4.502913 2.692230 -1.211878
H 4.030413 3.531092 -1.109049
C 1.546728 -3.701111 -0.064684
H 2.155530 -4.139714 -0.856610
H 2.012792 -4.000628 0.878543
O -0.373066 -3.444999 2.079966
H -0.931842 -2.851201 2.597467

H 0.087114 -5.272268 0.202601
H -0.257323 -4.207812 -1.153066

1a-c168, ΔG = 2.7636 kcal/mol, population = 0.06 %
C 1.426257 -2.956912 0.709090
C 1.679062 -2.276997 -0.626114
H 2.752007 -2.178135 -0.813428
C 1.095974 -0.871707 -0.590670
C -0.338573 -0.828159 -0.097183
C -0.870389 -1.850907 0.700470
N -2.125166 -1.813861 1.186147
C -2.862112 -0.760356 0.866084
C -2.422793 0.284971 0.045500
C -1.118672 0.270576 -0.451198
C -0.553038 1.336686 -1.350907
H -0.737235 2.326100 -0.929837
H -1.079218 1.307238 -2.309279
C 0.940735 1.114736 -1.569150
C 1.778429 1.308810 -0.279702
C 3.141060 1.933262 -0.523277
C 3.976890 2.190603 0.734934
C 4.505752 0.952534 1.470823
C 5.354132 0.004470 0.614787
C 6.566240 0.646809 -0.068620
C 7.567883 1.272072 0.902583
H 7.918178 0.535300 1.630765
H 7.125853 2.101229 1.458704
H 8.441035 1.659218 0.372945
H 7.073743 -0.118319 -0.663821
H 6.230768 1.406146 -0.781398
H 4.717306 -0.454994 -0.145525
H 5.703039 -0.815136 1.252436
H 5.095015 1.296452 2.326035
H 3.668555 0.386756 1.881788
H 3.379841 2.786284 1.433501
H 4.821140 2.824522 0.450393
H 2.962620 2.893414 -1.017047
H 3.685916 1.310267 -1.237210
H 1.220333 1.896000 0.454742
O 1.913846 -0.044309 0.226949
H 1.316207 1.703893 -2.401092
O 1.164057 -0.276409 -1.877187
C -3.495739 1.261504 -0.106083
O -3.463519 2.377867 -0.587107

C -4.786607 0.644760 0.515687
C -5.649189 0.147595 -0.619692
C -6.860242 0.606229 -0.905155
H -7.328591 1.381203 -0.311521
H -7.426508 0.205846 -1.736168
H -5.201620 -0.636530 -1.223589
C -4.262987 -0.532231 1.368516
H -4.241517 -0.228122 2.416857
H -4.875355 -1.429435 1.288493
O -5.431633 1.607972 1.329721
H -5.452120 2.438716 0.832722
C -0.069167 -3.085016 1.009214
H -0.231767 -3.354549 2.053705
H -0.497824 -3.896971 0.414381
O 1.056314 -3.045736 -1.653629
H 1.152911 -2.560906 -2.483251
H 1.890599 -3.943503 0.705699
H 1.920073 -2.367817 1.483861

1a-c139, ΔG = 2.9744 kcal/mol, population = 0.04 %
C -1.197504 -3.016558 -0.725801
C -1.602321 -2.184982 0.479957
H -2.690943 -2.102438 0.545756
C -1.050893 -0.775048 0.325805
C 0.424044 -0.740320 -0.028722
C 1.057149 -1.831548 -0.637557
N 2.352517 -1.805418 -1.005831
C 3.025287 -0.693624 -0.752144
C 2.481620 0.425792 -0.110845
C 1.137894 0.422176 0.260523
C 0.460572 1.565005 0.969426
H 0.663264 2.505483 0.455141
H 0.889073 1.662818 1.970881
C -1.042438 1.318331 1.068734
C -1.745933 1.328993 -0.310845
C -3.148857 1.914127 -0.338039
C -4.122810 1.300326 0.667056
C -5.543850 1.863859 0.561841
C -6.235807 1.641208 -0.789583
C -6.298391 0.182266 -1.255270
C -7.032923 -0.747598 -0.289652
H -7.114642 -1.755956 -0.701007
H -8.045093 -0.384516 -0.090862
H -6.515970 -0.825132 0.668955

H -6.795033 0.152692 -2.229648
H -5.285669 -0.195694 -1.423742
H -5.735970 2.234913 -1.560346
H -7.255650 2.034712 -0.722184
H -6.150509 1.419855 1.355799
H -5.519625 2.939246 0.767174
H -3.758166 1.469596 1.683578
H -4.140295 0.217695 0.529808
H -3.529301 1.789903 -1.354595
H -3.065735 2.993142 -0.170051
H -1.121144 1.843022 -1.044951
O -1.800025 -0.077482 -0.663684
H -1.509503 1.988993 1.784047
O -1.259539 -0.034175 1.518599
C 3.514691 1.450274 0.017909
O 3.414664 2.619053 0.340325
C 4.870521 0.792636 -0.372354
C 5.600239 0.525848 0.926065
C 5.698532 -0.635638 1.559845
H 5.259526 -1.549421 1.178954
H 6.226962 -0.709627 2.501768
H 6.035953 1.419624 1.364102
C 4.459700 -0.465225 -1.151418
H 4.514031 -0.244398 -2.219209
H 5.089424 -1.331328 -0.958852
O 5.625387 1.674337 -1.198546
H 5.553199 2.561174 -0.816117
C 0.323334 -3.124667 -0.860489
H 0.597018 -3.517527 -1.840654
H 0.709843 -3.838465 -0.126975
O -1.071691 -2.796447 1.654264
H -1.254972 -2.209211 2.398805
H -1.635253 -4.011704 -0.641488
H -1.624297 -2.547812 -1.614248

1a-c186, ΔG = 3.3070 kcal/mol, population = 0.02 %
C 1.870617 -3.011201 1.624729
C 2.013214 -3.056726 0.112016
H 3.053287 -3.240505 -0.172625
C 1.616160 -1.708281 -0.465527
C 0.263401 -1.219529 0.026885
C -0.252114 -1.641501 1.261003
N -1.399293 -1.158450 1.771071
C -2.048732 -0.259998 1.045369

C -1.634609 0.175930 -0.218179
C -0.439278 -0.303854 -0.755633
C 0.102915 0.107782 -2.100205
H 0.020483 1.187882 -2.226774
H -0.500933 -0.347968 -2.889871
C 1.553600 -0.341939 -2.212938
C 2.422848 0.322265 -1.106961
C 3.767280 0.871806 -1.539680
C 4.610763 1.443401 -0.389187
C 4.264420 2.870316 0.065834
C 2.842874 3.123585 0.585820
C 2.415258 2.240449 1.760560
C 0.942330 2.418754 2.127780
H 0.664265 1.793064 2.978476
H 0.294890 2.141976 1.291138
H 0.719681 3.457157 2.386977
H 2.594476 1.189652 1.526013
H 3.044690 2.470505 2.626593
H 2.124963 3.015759 -0.232913
H 2.772800 4.172874 0.891289
H 4.976144 3.148693 0.850427
H 4.451215 3.554551 -0.768565
H 5.655216 1.452200 -0.711535
H 4.567258 0.754602 0.457295
H 3.590630 1.645947 -2.293566
H 4.316385 0.065719 -2.034345
H 1.832949 1.100092 -0.627671
O 2.612932 -0.747442 -0.150196
H 1.955680 -0.211386 -3.213933
O 1.618494 -1.746340 -1.884525
C -2.584608 1.162936 -0.721253
O -2.495286 1.903716 -1.681278
C -3.811384 1.171343 0.242341
C -4.921479 0.393428 -0.423204
C -6.097952 0.899834 -0.766565
H -6.350709 1.933913 -0.568711
H -6.851254 0.286092 -1.243420
H -4.692494 -0.649509 -0.622983
C -3.299538 0.440391 1.502861
H -3.054676 1.181279 2.266537
H -4.025188 -0.253942 1.924596
O -4.168613 2.507190 0.549885
H -4.177005 3.003716 -0.281300
C 0.432183 -2.717547 2.057222

H 0.394101 -2.448657 3.113620
H -0.177369 -3.620012 1.950593
O 1.162623 -4.081850 -0.397858
H 1.202440 -4.046031 -1.362080
H 2.198572 -3.961788 2.046371
H 2.543831 -2.238723 1.999830

Table S3. Geometry data of conformers of structure **1b**.

1b-c207, ΔG = 0.0000 kcal/mol, population = 8.10 %
C 0.672187 4.115793 0.803894
C -0.155346 3.628341 -0.373735
H -1.172527 4.027274 -0.324148
C -0.261215 2.111114 -0.318855
C 1.078725 1.423057 -0.135240
C 2.174559 2.091267 0.428489
N 3.359953 1.490033 0.641114
C 3.457610 0.215544 0.292663
C 2.417773 -0.522380 -0.284694
C 1.186977 0.089430 -0.524384
C 0.020837 -0.600411 -1.179756
H -0.162276 -1.570015 -0.713315
H 0.274217 -0.805053 -2.223371
C -1.227927 0.273220 -1.107699
C -1.749175 0.474342 0.336193
C -3.258184 0.581678 0.452896
C -3.969052 -0.759325 0.272000
C -5.488294 -0.641088 0.380684
C -6.213234 -1.971416 0.185711
C -7.733285 -1.855607 0.292199
C -8.450134 -3.189074 0.087871
H -8.230052 -3.604391 -0.899114
H -9.533584 -3.077975 0.169015
H -8.133095 -3.922785 0.833746
H -7.994245 -1.445637 1.273613
H -8.090397 -1.130858 -0.447136
H -5.951030 -2.385080 -0.794861
H -5.853605 -2.692739 0.928531
H -5.748926 -0.225693 1.360427
H -5.847160 0.078554 -0.363536
H -3.604436 -1.467298 1.024046
H -3.714447 -1.188914 -0.702690
H -3.615235 1.305114 -0.286643
H -3.493352 0.991061 1.438994

H -1.371122 -0.312278 0.994281
O -1.147106 1.734098 0.731193
H -2.000405 -0.078071 -1.785688
O -0.880581 1.618084 -1.496584
C 2.878797 -1.881650 -0.543821
O 2.354603 -2.765395 -1.193935
C 4.262334 -2.049856 0.156330
C 4.031215 -2.799908 1.446904
C 4.539951 -3.991428 1.729521
H 5.188731 -4.511003 1.035658
H 4.333111 -4.475788 2.675140
H 3.389960 -2.292426 2.161958
C 4.715901 -0.599331 0.433605
H 5.442606 -0.303718 -0.325584
H 5.176828 -0.470955 1.412149
O 5.166250 -2.702602 -0.716802
H 4.695936 -3.450564 -1.112908
C 2.094828 3.552858 0.771510
H 2.597377 3.715967 1.725951
H 2.685806 4.085066 0.020387
O 0.486647 4.035055 -1.580782
H -0.000108 3.645291 -2.318270
H 0.703195 5.205720 0.797043
H 0.163650 3.810550 1.720077

1b-c170, ΔG = 0.0747 kcal/mol, population = 7.14 %
C 0.861396 -3.491097 1.721368
C 1.196571 -3.500669 0.239379
H 2.231691 -3.815081 0.078250
C 1.061894 -2.088944 -0.312077
C -0.257893 -1.430686 0.045218
C -1.002122 -1.843094 1.159187
N -2.152780 -1.246954 1.523740
C -2.566347 -0.232989 0.777859
C -1.890743 0.234317 -0.355340
C -0.704389 -0.380988 -0.755210
C 0.076359 0.017720 -1.979139
H 0.210698 1.100391 -2.010915
H -0.501236 -0.246713 -2.868961
C 1.425684 -0.693075 -2.000883
C 2.351206 -0.257893 -0.837435
C 3.821261 -0.181915 -1.202650
C 4.747170 0.070835 -0.007446
C 4.399186 1.306163 0.830497

C 4.439018 2.624687 0.056157
C 4.106471 3.854185 0.907530
C 2.653743 3.897302 1.384463
H 2.416438 3.057554 2.040289
H 1.966033 3.858685 0.535041
H 2.447659 4.815430 1.938716
H 4.777787 3.886882 1.772448
H 4.315202 4.755196 0.323145
H 5.436540 2.744903 -0.379697
H 3.740998 2.587493 -0.786623
H 3.414255 1.168646 1.284138
H 5.104941 1.369161 1.665269
H 5.771311 0.163904 -0.381612
H 4.731727 -0.809596 0.639212
H 3.934933 0.612107 -1.945675
H 4.104956 -1.117266 -1.693428
H 2.007127 0.690814 -0.421160
O 2.143955 -1.293307 0.157638
H 1.908385 -0.614052 -2.971186
O 1.214972 -2.093075 -1.723071
C -2.631355 1.351287 -0.930554
O -2.488248 1.903664 -2.004278
C -3.741824 1.751623 0.088000
C -3.252905 2.969544 0.836543
C -3.837344 4.159403 0.804691
H -4.741560 4.332001 0.234756
H -3.433967 4.993952 1.363601
H -2.350687 2.815147 1.421543
C -3.841031 0.529093 1.027327
H -4.703509 -0.073362 0.735696
H -3.955389 0.799380 2.076276
O -4.964062 1.966725 -0.594131
H -4.764929 2.490760 -1.383722
C -0.574964 -3.028844 1.978967
H -0.721932 -2.794697 3.034371
H -1.272167 -3.839261 1.747089
O 0.293108 -4.380222 -0.427287
H 0.457882 -4.307109 -1.376068
H 1.009565 -4.490320 2.131966
H 1.569100 -2.826521 2.219958

1b-c5, ΔG = 0.0772 kcal/mol, population = 7.11 %
C -0.688612 3.221830 1.451508
C -1.009757 3.024109 -0.020932

H -2.079674 3.156042 -0.205755
C -0.654618 1.600300 -0.424276
C 0.743379 1.189284 -0.002507
C 1.408500 1.827193 1.053549
N 2.633672 1.455148 1.469502
C 3.201923 0.441652 0.832708
C 2.612037 -0.244104 -0.235248
C 1.348053 0.136855 -0.686540
C 0.646004 -0.505456 -1.852725
H 0.660172 -1.592598 -1.756683
H 1.197250 -0.271790 -2.767526
C -0.787843 0.004503 -1.963239
C -1.684355 -0.432508 -0.779749
C -3.128337 -0.692543 -1.164063
C -4.031621 -1.007309 0.026036
C -5.482523 -1.254046 -0.383330
C -6.405001 -1.547761 0.798125
C -7.857892 -1.787089 0.389144
C -8.776825 -2.066530 1.576949
H -8.777851 -1.226943 2.277199
H -8.450242 -2.954032 2.125567
H -9.807158 -2.233765 1.255566
H -8.224356 -0.912511 -0.158908
H -7.899746 -2.627107 -0.312254
H -6.033239 -2.425313 1.339635
H -6.363202 -0.711458 1.505466
H -5.857224 -0.377046 -0.923317
H -5.522712 -2.089671 -1.091304
H -3.990283 -0.178735 0.738041
H -3.645078 -1.888570 0.550221
H -3.140835 -1.531535 -1.867721
H -3.506336 0.183149 -1.701223
H -1.259340 -1.303236 -0.273812
O -1.612060 0.700436 0.125187
H -1.226115 -0.237849 -2.927461
O -0.782629 1.440910 -1.828548
C 3.519841 -1.287051 -0.698960
O 3.462837 -1.971189 -1.702552
C 4.685783 -1.389578 0.331995
C 4.422349 -2.594222 1.204013
C 5.211670 -3.656963 1.281953
H 6.133925 -3.721882 0.718477
H 4.963581 -4.489561 1.927566
H 3.506097 -2.546682 1.785442

C 4.578766 -0.081227 1.146611
H 5.337273 0.620252 0.793866
H 4.724458 -0.226687 2.216293
O 5.924204 -1.454536 -0.352818
H 5.823619 -2.095519 -1.071537
C 0.800058 3.016497 1.742860
H 0.974851 2.925644 2.815923
H 1.365483 3.893729 1.415252
O -0.247185 3.958533 -0.782409
H -0.388960 3.761420 -1.717149
H -0.992607 4.223630 1.756424
H -1.288694 2.511741 2.023186

1b-c120, ΔG = 0.3533 kcal/mol, population = 4.46 %
C 0.879027 -3.416669 0.834617
C 1.353312 -2.751848 -0.446894
H 2.445884 -2.720643 -0.486699
C 0.859118 -1.312614 -0.475254
C -0.626283 -1.184706 -0.190750
C -1.324751 -2.175237 0.513108
N -2.634034 -2.069335 0.807699
C -3.253585 -0.970897 0.401926
C -2.634131 0.062063 -0.311118
C -1.280926 -0.038118 -0.636142
C -0.531735 0.993368 -1.436003
H -0.715930 1.993465 -1.039364
H -0.917449 0.993783 -2.458913
C 0.962248 0.683958 -1.442469
C 1.619631 0.820923 -0.047983
C 3.045122 1.337225 -0.083408
C 3.707880 1.369976 1.293363
C 5.112361 1.979150 1.288634
C 6.143410 1.182860 0.487965
C 7.557282 1.753424 0.594039
C 8.583734 0.962545 -0.214870
H 8.629522 -0.077091 0.120158
H 9.584705 1.388566 -0.117911
H 8.325179 0.956484 -1.277076
H 7.549656 2.795451 0.256840
H 7.857659 1.774814 1.646996
H 6.145635 0.143831 0.838004
H 5.854276 1.149595 -0.567055
H 5.062275 3.001881 0.897358
H 5.460567 2.064043 2.323169

H 3.752764 0.353550 1.694096
H 3.071683 1.944871 1.974518
H 3.023205 2.349148 -0.501983
H 3.617527 0.714942 -0.775936
H 1.006115 1.442218 0.609815
O 1.601318 -0.538881 0.461341
H 1.486457 1.252374 -2.205869
O 1.145197 -0.717822 -1.731603
C -3.614433 1.102835 -0.599506
O -3.528792 2.061039 -1.343144
C -4.902599 0.774472 0.216234
C -4.911085 1.667030 1.434460
C -5.837316 2.579212 1.696081
H -6.683404 2.734376 1.038477
H -5.783202 3.188423 2.589105
H -4.075344 1.521934 2.113025
C -4.720073 -0.706265 0.617211
H -5.316551 -1.330938 -0.050565
H -5.025976 -0.917146 1.641166
O -6.044665 0.936936 -0.605382
H -5.940489 1.772815 -1.082840
C -0.648368 -3.452218 0.927196
H -0.968326 -3.704157 1.939362
H -1.040408 -4.241192 0.278861
O 0.829102 -3.477179 -1.557583
H 1.061028 -2.993492 -2.360649
H 1.278207 -4.430032 0.885689
H 1.297513 -2.859696 1.674683

1b-c2, ΔG = 0.4198 kcal/mol, population = 3.99 %
C -0.542042 3.261526 1.369580
C -0.856250 3.077930 -0.105800
H -1.918495 3.251480 -0.300425
C -0.552826 1.641678 -0.506817
C 0.825346 1.177101 -0.075132
C 1.501351 1.785034 0.990558
N 2.703848 1.359461 1.423727
C 3.234888 0.322467 0.794401
C 2.633669 -0.333367 -0.286107
C 1.395326 0.101341 -0.755127
C 0.680649 -0.509297 -1.931127
H 0.658744 -1.596982 -1.843445
H 1.243462 -0.285823 -2.841467
C -0.735005 0.048284 -2.043584

C -1.642967 -0.358525 -0.857117
C -3.093417 -0.593331 -1.232836
C -3.986643 -0.900181 -0.032844
C -5.448388 -1.119910 -0.418081
C -6.344492 -1.437947 0.777610
C -7.811463 -1.644823 0.402680
C -8.695952 -1.966903 1.606058
H -8.362107 -2.881357 2.103447
H -9.737915 -2.108833 1.311107
H -8.664543 -1.159385 2.342351
H -8.184899 -0.743996 -0.095744
H -7.883887 -2.453680 -0.332064
H -5.970478 -2.337419 1.280060
H -6.271443 -0.625663 1.509958
H -5.825967 -0.224684 -0.924907
H -5.513617 -1.935805 -1.146861
H -3.919918 -0.078141 0.684836
H -3.606903 -1.791856 0.478587
H -3.125497 -1.429741 -1.938809
H -3.462834 0.290068 -1.763119
H -1.236004 -1.234252 -0.344491
O -1.546390 0.779655 0.038575
H -1.181921 -0.180728 -3.007139
O -0.680127 1.483910 -1.911410
C 3.504008 -1.418931 -0.729763
O 3.452576 -2.091994 -1.741699
C 4.610342 -1.594041 0.350555
C 4.218126 -2.811791 1.158492
C 3.615952 -2.821974 2.340763
H 3.356068 -1.915267 2.873123
H 3.353749 -3.756145 2.820822
H 4.433826 -3.748909 0.652893
C 4.580507 -0.268626 1.126429
H 5.373155 0.376667 0.742941
H 4.732351 -0.380820 2.198107
O 5.877966 -1.790428 -0.268547
H 5.745378 -2.398424 -1.010756
C 0.934345 2.997665 1.675207
H 1.094822 2.899117 2.749783
H 1.536840 3.852322 1.353873
O -0.051292 3.982862 -0.859119
H -0.198011 3.797372 -1.795451
H -0.809150 4.274325 1.672677
H -1.175193 2.575247 1.934588

1b-c39, ΔG = 0.6074 kcal/mol, population = 2.90 %
C 0.596401 -3.397467 1.261464
C 0.978151 -3.085323 -0.176029
H 2.051342 -3.227929 -0.331768
C 0.669737 -1.624841 -0.471132
C -0.735511 -1.217522 -0.071819
C -1.453446 -1.921866 0.904464
N -2.685271 -1.554613 1.304563
C -3.207613 -0.480384 0.730990
C -2.563296 0.273925 -0.256201
C -1.290550 -0.098995 -0.689562
C -0.528980 0.619768 -1.770662
H -0.528437 1.695710 -1.587488
H -1.045155 0.471766 -2.723028
C 0.899026 0.089732 -1.864107
C 1.750931 0.407188 -0.611532
C 3.215814 0.669005 -0.904618
C 4.055516 0.882366 0.352536
C 5.533587 1.121169 0.048393
C 6.371193 1.348523 1.306988
C 7.874524 1.489701 1.049023
C 8.252951 2.720578 0.224477
H 9.336782 2.807452 0.121735
H 7.888458 3.634963 0.701008
H 7.829500 2.679861 -0.780928
H 8.393727 1.531713 2.011216
H 8.237157 0.587145 0.545048
H 6.008498 2.244554 1.824104
H 6.206667 0.511180 1.993370
H 5.933329 0.259070 -0.498167
H 5.625816 1.980219 -0.623105
H 3.954695 0.011052 1.005255
H 3.656176 1.736959 0.910419
H 3.277151 1.556414 -1.543076
H 3.609467 -0.172226 -1.483879
H 1.318538 1.241020 -0.052364
O 1.620046 -0.796482 0.190564
H 1.382821 0.401874 -2.785609
O 0.860626 -1.352434 -1.850794
C -3.432026 1.369573 -0.669989
O -3.322120 2.128487 -1.613704
C -4.637056 1.416169 0.319335
C -4.391950 2.547844 1.288836

C -5.168934 3.615062 1.414840
H -6.066556 3.736498 0.821726
H -4.935541 4.393599 2.129660
H -3.500609 2.441640 1.900494
C -4.584120 0.048383 1.036056
H -5.343615 -0.607677 0.606546
H -4.764615 0.117084 2.108101
O -5.845198 1.554226 -0.407926
H -5.704125 2.241460 -1.075273
C -0.897081 -3.177079 1.517065
H -1.108041 -3.166436 2.587445
H -1.471027 -4.010864 1.102340
O 0.225856 -3.933643 -1.041460
H 0.407212 -3.661619 -1.950226
H 0.864189 -4.429456 1.490115
H 1.191513 -2.755208 1.913040

1b-c41, ΔG = 0.6219 kcal/mol, population = 2.83 %
C -0.854853 -3.097722 -1.974255
C -0.974714 -3.356241 -0.481349
H -1.981652 -3.698974 -0.226653
C -0.735812 -2.055682 0.271535
C 0.530615 -1.339385 -0.161094
C 1.101431 -1.558289 -1.422487
N 2.200413 -0.905444 -1.844843
C 2.736634 -0.028901 -1.008562
C 2.237832 0.241947 0.270734
C 1.106576 -0.434412 0.728248
C 0.515083 -0.246292 2.099196
H 0.401504 0.815655 2.324339
H 1.211509 -0.644705 2.841855
C -0.826293 -0.965258 2.204240
C -1.915088 -0.357694 1.286072
C -3.313076 -0.396633 1.877212
C -4.418838 0.015430 0.902825
C -4.306158 1.449884 0.386143
C -5.470937 1.847778 -0.519059
C -5.364091 3.273525 -1.058431
C -6.529494 3.656934 -1.968758
H -7.481732 3.588320 -1.436112
H -6.585667 2.990612 -2.833613
H -6.428229 4.678755 -2.340805
H -5.310191 3.972923 -0.217399
H -4.421555 3.379889 -1.605936

H -5.527625 1.147772 -1.360617
H -6.411879 1.739641 0.032639
H -4.253911 2.140212 1.235949
H -3.371102 1.574455 -0.169042
H -5.382418 -0.106264 1.406959
H -4.424370 -0.675787 0.055620
H -3.322473 0.266402 2.748693
H -3.501931 -1.410349 2.242266
H -1.642835 0.658991 0.996519
O -1.859336 -1.198558 0.104321
H -1.154665 -1.054637 3.236196
O -0.680673 -2.297263 1.668909
C 3.078921 1.245775 0.912993
O 3.097381 1.611486 2.072490
C 4.051522 1.806587 -0.169387
C 3.507729 3.140839 -0.623265
C 4.136118 4.300260 -0.484485
H 5.119507 4.363425 -0.035949
H 3.687670 5.222114 -0.831853
H 2.524783 3.100839 -1.084072
C 3.980749 0.764835 -1.307981
H 4.860314 0.120309 -1.255221
H 3.948488 1.212643 -2.300413
O 5.363018 1.886412 0.359417
H 5.295176 2.277757 1.242473
C 0.539087 -2.593675 -2.356087
H 0.539301 -2.187697 -3.368729
H 1.245602 -3.428774 -2.362767
O -0.001453 -4.329692 -0.107543
H -0.025461 -4.415511 0.854100
H -1.080550 -4.014947 -2.519014
H -1.612992 -2.362036 -2.248255

1b-c27, ΔG = 0.6507 kcal/mol, population = 2.70 %
C -1.270578 3.973381 -1.346242
C -0.491451 3.892984 -0.044012
H 0.382690 4.549768 -0.073028
C 0.017710 2.471940 0.149031
C -1.060770 1.419732 -0.029286
C -2.219797 1.677177 -0.774233
N -3.173795 0.748969 -0.976106
C -2.973753 -0.444375 -0.436698
C -1.849767 -0.782814 0.325638
C -0.859337 0.172047 0.557828

C 0.360270 -0.073845 1.404770
H 0.847178 -1.006772 1.114771
H 0.046292 -0.202666 2.444059
C 1.332978 1.096198 1.294755
C 1.951105 1.243067 -0.116598
C 3.379863 1.758951 -0.127666
C 4.414682 0.752906 0.384877
C 4.574159 -0.497633 -0.481789
C 5.689619 -1.418315 0.009111
C 5.868143 -2.670497 -0.848679
C 6.987930 -3.581689 -0.349647
H 6.792341 -3.919602 0.671466
H 7.947235 -3.057439 -0.346632
H 7.093765 -4.467498 -0.979620
H 4.925481 -3.227587 -0.872111
H 6.072531 -2.371128 -1.882116
H 6.632858 -0.860556 0.034440
H 5.482766 -1.716488 1.043426
H 3.636832 -1.062227 -0.508438
H 4.780002 -0.195349 -1.514784
H 4.170154 0.453249 1.409638
H 5.380034 1.263596 0.446911
H 3.414057 2.670837 0.475091
H 3.626028 2.046555 -1.153575
H 1.868007 0.306626 -0.670108
O 1.091425 2.224353 -0.753538
H 2.082642 1.066553 2.079919
O 0.598079 2.329440 1.436247
C -1.965371 -2.169033 0.763864
O -1.305503 -2.778962 1.583100
C -3.168622 -2.804219 0.001872
C -2.606649 -3.627499 -1.133014
C -2.748555 -4.940065 -1.257140
H -3.309202 -5.521423 -0.536106
H -2.318342 -5.470120 -2.097108
H -2.047187 -3.062865 -1.873302
C -3.951660 -1.585365 -0.535329
H -4.809898 -1.401505 0.113929
H -4.318401 -1.721379 -1.551908
O -3.961185 -3.557641 0.901716
H -3.357266 -4.082214 1.447406
C -2.486945 3.044359 -1.339432
H -2.894323 2.933203 -2.345376
H -3.284650 3.484755 -0.734168

O -1.359346 4.256017 1.028287
H -0.884397 4.103586 1.855290
H -1.589556 5.002528 -1.513638
H -0.593070 3.706350 -2.159162

1b-c1, ΔG = 0.6896 kcal/mol, population = 2.53 %
C -0.950412 3.922502 -1.022905
C -0.072766 3.579066 0.169477
H 0.907658 4.055468 0.079637
C 0.156841 2.074943 0.205624
C -1.123631 1.269678 0.084133
C -2.276164 1.809625 -0.501142
N -3.410877 1.100720 -0.658182
C -3.396883 -0.153707 -0.235031
C -2.292932 -0.767328 0.368424
C -1.116729 -0.043299 0.554057
C 0.108848 -0.593254 1.233535
H 0.367477 -1.571395 0.824074
H -0.116810 -0.752771 2.291291
C 1.279673 0.374048 1.090050
C 1.766937 0.527069 -0.371485
C 3.261669 0.743777 -0.517226
C 4.073895 -0.525617 -0.262330
C 5.579546 -0.303282 -0.394141
C 6.400070 -1.562952 -0.123470
C 7.907423 -1.346034 -0.250204
C 8.718514 -2.609887 0.030037
H 9.790841 -2.427792 -0.068606
H 8.450330 -3.409677 -0.665350
H 8.534036 -2.976799 1.043186
H 8.134379 -0.980720 -1.257362
H 8.215049 -0.553069 0.439717
H 6.172781 -1.932079 0.883292
H 6.089744 -2.353328 -0.816581
H 5.803579 0.067170 -1.400603
H 5.889192 0.485880 0.300007
H 3.758188 -1.304517 -0.964735
H 3.858724 -0.911166 0.739912
H 3.568831 1.537477 0.170821
H 3.455169 1.106585 -1.530278
H 1.445602 -0.325982 -0.974671
O 1.061505 1.708063 -0.832230
H 2.085718 0.130556 1.776181
O 0.825933 1.707263 1.401890

C -2.637774 -2.145643 0.705215
O -2.043376 -2.947260 1.400727
C -3.992280 -2.465384 0.008162
C -3.653928 -3.299031 -1.208479
C -3.580310 -2.880794 -2.465513
H -3.784034 -1.857517 -2.755754
H -3.302833 -3.561676 -3.260098
H -3.413985 -4.330909 -0.967163
C -4.581991 -1.081837 -0.301960
H -5.296527 -0.825311 0.482490
H -5.101994 -1.026049 -1.256125
O -4.842954 -3.190847 0.890924
H -4.299616 -3.862481 1.328591
C -2.321540 3.248734 -0.933403
H -2.845512 3.310934 -1.888260
H -2.946333 3.776988 -0.207266
O -0.733199 4.006777 1.359188
H -0.208961 3.703630 2.111509
H -1.070589 5.004582 -1.083308
H -0.428233 3.602059 -1.926151

1b-c29, ΔG = 0.6934 kcal/mol, population = 2.51 %
C 0.490396 3.988501 1.109209
C -0.263704 3.602149 -0.152190
H -1.282270 4.000130 -0.132058
C -0.370883 2.086159 -0.226619
C 0.960932 1.384245 -0.033423
C 2.020881 2.000893 0.646033
N 3.199153 1.387006 0.862249
C 3.326359 0.151475 0.401003
C 2.323516 -0.533361 -0.294555
C 1.100587 0.092653 -0.537829
C -0.025979 -0.540116 -1.309414
H -0.220422 -1.550078 -0.943987
H 0.276108 -0.643958 -2.354949
C -1.283582 0.316750 -1.210879
C -1.856725 0.387016 0.227630
C -3.371001 0.431502 0.308017
C -4.017719 -0.922871 0.020883
C -5.542158 -0.886510 0.117526
C -6.187657 -2.242909 -0.165864
C -7.719261 -2.231262 -0.140642
C -8.317492 -1.915525 1.230681
H -7.952964 -2.618540 1.984894

H -9.407237 -1.984383 1.209776
H -8.058423 -0.908826 1.564082
H -8.084331 -1.504836 -0.874838
H -8.082780 -3.208794 -0.471402
H -5.851680 -2.593340 -1.147534
H -5.821626 -2.975355 0.563183
H -5.825339 -0.537112 1.114664
H -5.932661 -0.146945 -0.590840
H -3.628307 -1.664716 0.726602
H -3.731154 -1.270170 -0.977328
H -3.739961 1.188019 -0.391473
H -3.646860 0.763416 1.312608
H -1.471248 -0.438557 0.831850
O -1.309204 1.627808 0.741201
H -2.027928 0.022517 -1.945179
O -0.930224 1.691373 -1.469688
C 2.809989 -1.859155 -0.659304
O 2.326824 -2.679140 -1.415994
C 4.160709 -2.086059 0.086911
C 3.878539 -2.963062 1.283811
C 4.382950 -4.175694 1.466855
H 5.065546 -4.620443 0.753758
H 4.137816 -4.754302 2.348166
H 3.202022 -2.533040 2.016920
C 4.583973 -0.666957 0.527154
H 5.345586 -0.294499 -0.160641
H 4.992908 -0.632173 1.536145
O 5.111716 -2.642542 -0.803092
H 4.668126 -3.349158 -1.294563
C 1.913378 3.424206 1.118501
H 2.351296 3.495183 2.115252
H 2.552482 4.021785 0.462003
O 0.450562 4.102264 -1.281064
H 0.012850 3.772303 -2.076174
H 0.521082 5.075180 1.193356
H -0.073197 3.611975 1.964542

1b-c23, ΔG = 0.7800 kcal/mol, population = 2.17 %
C -0.115149 3.738602 1.234458
C -0.722138 3.335755 -0.099487
H -1.788129 3.578752 -0.129827
C -0.596768 1.829074 -0.271077
C 0.807898 1.312763 -0.016205
C 1.718496 2.027986 0.774054

N 2.955577 1.573158 1.047736
C 3.291596 0.399540 0.532851
C 2.449154 -0.372852 -0.274890
C 1.168180 0.089163 -0.577820
C 0.201877 -0.643648 -1.469311
H 0.131356 -1.692877 -1.176958
H 0.588611 -0.634560 -2.491826
C -1.170617 0.019415 -1.424051
C -1.853490 -0.087894 -0.037708
C -3.361673 -0.244333 -0.088786
C -3.789938 -1.652660 -0.506168
C -5.299217 -1.802344 -0.714447
C -6.137419 -1.576463 0.543828
C -7.628327 -1.832060 0.325529
C -8.465807 -1.592736 1.580471
H -8.144254 -2.245519 2.396346
H -9.525268 -1.784747 1.397535
H -8.368577 -0.559919 1.925452
H -7.988012 -1.186718 -0.482992
H -7.770041 -2.861948 -0.018764
H -5.771295 -2.231764 1.343046
H -6.004280 -0.551232 0.902820
H -5.624894 -1.106088 -1.495479
H -5.500955 -2.808056 -1.096811
H -3.454014 -2.369650 0.250845
H -3.281022 -1.928178 -1.434994
H -3.768333 0.497891 -0.783416
H -3.757366 -0.006765 0.900384
H -1.406520 -0.895597 0.547746
O -1.520662 1.175285 0.593130
H -1.809344 -0.326032 -2.232075
O -1.003740 1.444400 -1.574889
C 3.146149 -1.591040 -0.671335
O 2.841140 -2.419213 -1.507727
C 4.457037 -1.676238 0.168848
C 4.211632 -2.654559 1.293647
C 4.866932 -3.795522 1.458325
H 5.657741 -4.098231 0.783659
H 4.639806 -4.454402 2.286497
H 3.427267 -2.365798 1.987504
C 4.641694 -0.242523 0.714403
H 5.389109 0.274316 0.109340
H 4.969140 -0.215489 1.752933
O 5.540495 -2.042481 -0.666103

H 5.238554 -2.769834 -1.229452
C 1.372584 3.388839 1.311589
H 1.734124 3.461232 2.338381
H 1.953016 4.112840 0.732364
O -0.021266 4.012024 -1.141590
H -0.355409 3.679890 -1.984628
H -0.253552 4.809481 1.385925
H -0.667984 3.224409 2.022533

1b-c6, ΔG = 0.8283 kcal/mol, population = 2.00 %
C -0.784972 -3.313343 -0.924078
C -1.435228 -2.466787 0.157743
H -2.515467 -2.400742 0.000560
C -0.880010 -1.051747 0.087390
C 0.635442 -1.002786 0.043322
C 1.392267 -2.096671 -0.397679
N 2.735317 -2.061494 -0.484975
C 3.329999 -0.930839 -0.133996
C 2.650989 0.204130 0.324943
C 1.260415 0.179343 0.435399
C 0.439469 1.327645 0.959030
H 0.730596 2.260056 0.472162
H 0.655453 1.457148 2.022943
C -1.049908 1.058262 0.760839
C -1.465444 1.024504 -0.729561
C -2.847485 1.578092 -1.038618
C -3.994364 0.944456 -0.253289
C -5.357151 1.513724 -0.644329
C -6.515553 0.876742 0.120958
C -7.884213 1.432456 -0.270515
C -9.034684 0.783935 0.497288
H -9.062436 -0.294955 0.322847
H -10.000281 1.195658 0.195764
H -8.926006 0.942057 1.573566
H -7.894520 2.514420 -0.101148
H -8.035044 1.289634 -1.345813
H -6.505243 -0.206593 -0.045848
H -6.362032 1.021667 1.196569
H -5.362763 2.596435 -0.474239
H -5.512993 1.371760 -1.719767
H -3.840592 1.090490 0.820031
H -3.989461 -0.135482 -0.419996
H -3.025468 1.451147 -2.110744
H -2.818886 2.657609 -0.856923

H -0.720215 1.541539 -1.338359
O -1.416757 -0.388693 -1.053144
H -1.658877 1.736481 1.351503
O -1.336457 -0.287857 1.192730
C 3.617842 1.254303 0.624665
O 3.459667 2.306628 1.212965
C 5.000308 0.791144 0.071551
C 5.225249 1.500134 -1.243295
C 6.212486 2.352632 -1.482135
H 6.953517 2.588833 -0.728895
H 6.316496 2.828445 -2.448754
H 4.497980 1.270629 -2.016779
C 4.822217 -0.728235 -0.144295
H 5.280226 -1.261396 0.691093
H 5.276230 -1.088725 -1.066431
O 6.011785 1.050624 1.027846
H 5.865458 1.945723 1.367007
C 0.732336 -3.402364 -0.744275
H 1.204711 -3.804865 -1.641592
H 0.969011 -4.100699 0.063784
O -1.145898 -3.048712 1.427553
H -1.483941 -2.451949 2.107515
H -1.220393 -4.312931 -0.911372
H -1.027147 -2.865584 -1.889520

1b-c13, ΔG = 0.8409 kcal/mol, population = 1.96 %
C 0.703746 -3.292728 1.072327
C 1.208345 -2.763857 -0.259845
H 2.301050 -2.791049 -0.299736
C 0.787186 -1.309161 -0.411364
C -0.689228 -1.082962 -0.142192
C -1.434353 -1.972184 0.642665
N -2.736250 -1.774206 0.926403
C -3.298163 -0.682892 0.430379
C -2.629284 0.252482 -0.368030
C -1.286265 0.055028 -0.683932
C -0.487385 0.974606 -1.568172
H -0.620346 2.013304 -1.260164
H -0.873241 0.904953 -2.588663
C 0.989193 0.591483 -1.542372
C 1.650530 0.812707 -0.160341
C 3.097716 1.260582 -0.229892
C 3.759194 1.373855 1.143129
C 5.186130 1.926702 1.097796

C 6.185189 1.036788 0.357439
C 7.620646 1.556512 0.429290
C 8.614946 0.673568 -0.322515
H 8.620087 -0.342306 0.081426
H 9.631906 1.065589 -0.252052
H 8.355896 0.606560 -1.382507
H 7.654578 2.573307 0.023556
H 7.921513 1.636005 1.479312
H 6.145392 0.024874 0.777676
H 5.895941 0.942951 -0.693904
H 5.174935 2.920138 0.634661
H 5.538079 2.071576 2.124392
H 3.764064 0.390260 1.620832
H 3.144853 2.023471 1.775449
H 3.121421 2.235764 -0.728071
H 3.642994 0.560347 -0.867720
H 1.064350 1.513008 0.440683
O 1.569612 -0.498072 0.458689
H 1.541839 1.067108 -2.348036
O 1.101528 -0.837046 -1.712324
C -3.558452 1.317791 -0.734899
O -3.439457 2.201818 -1.561954
C -4.835427 1.136221 0.135904
C -4.747760 2.173830 1.234086
C -4.349517 1.976311 2.484197
H -4.055841 1.003005 2.857475
H -4.298261 2.798760 3.186212
H -5.006818 3.174649 0.899842
C -4.748383 -0.319385 0.617856
H -5.370728 -0.938010 -0.031672
H -5.086295 -0.467427 1.641547
O -6.004071 1.338874 -0.652543
H -5.845048 2.114614 -1.210090
C -0.823314 -3.242591 1.164561
H -1.152944 -3.391641 2.193849
H -1.256288 -4.062613 0.584386
O 0.646115 -3.553788 -1.306057
H 0.900969 -3.152911 -2.146874
H 1.051339 -4.317060 1.209621
H 1.151760 -2.688466 1.863013

1b-c132, ΔG = 0.8754 kcal/mol, population = 1.85 %
C -1.070273 3.021412 2.002416
C -1.234518 3.279887 0.513858

H -2.263839 3.568290 0.282240
C -0.941949 1.997498 -0.251016
C 0.369260 1.347026 0.152058
C 0.952722 1.589325 1.403555
N 2.092140 0.992912 1.801090
C 2.657088 0.149599 0.949636
C 2.148015 -0.140947 -0.321051
C 0.973975 0.477046 -0.752969
C 0.365585 0.263286 -2.112610
H 0.303686 -0.802240 -2.340903
H 1.024643 0.700952 -2.867190
C -1.014079 0.910014 -2.186183
C -2.048185 0.241145 -1.247607
C -3.458677 0.201302 -1.807770
C -4.517133 -0.271051 -0.808407
C -4.303834 -1.692568 -0.287589
C -5.426572 -2.154046 0.641979
C -5.265831 -3.585866 1.162344
C -4.057121 -3.782926 2.078443
H -4.099046 -3.097053 2.929134
H -3.116649 -3.602608 1.554283
H -4.023628 -4.801031 2.472265
H -6.173171 -3.866382 1.705586
H -5.194842 -4.271219 0.310733
H -5.491332 -1.467406 1.494225
H -6.380125 -2.072889 0.109655
H -4.230019 -2.384330 -1.135062
H -3.348893 -1.748998 0.240390
H -5.496932 -0.210350 -1.291903
H -4.545786 0.423154 0.035854
H -3.450399 -0.462478 -2.678669
H -3.711910 1.202500 -2.168396
H -1.712675 -0.758922 -0.967292
O -2.014894 1.081391 -0.064921
H -1.368983 0.985344 -3.210490
O -0.928720 2.246308 -1.648166
C 3.027240 -1.095890 -0.985698
O 3.039373 -1.456765 -2.146808
C 4.053070 -1.606945 0.072378
C 3.597507 -2.973841 0.525551
C 4.284209 -4.095018 0.352834
H 5.256137 -4.098085 -0.124367
H 3.898005 -5.044410 0.700802
H 2.627872 -2.993076 1.014964

C 3.946906 -0.578614 1.220473
H 4.790007 0.112044 1.157854
H 3.956210 -1.034888 2.209554
O 5.355357 -1.606459 -0.485036
H 5.291075 -1.995861 -1.369159
C 0.355692 2.589914 2.353510
H 0.397411 2.180287 3.363823
H 1.017607 3.460870 2.350589
O -0.324043 4.306893 0.125768
H -0.379612 4.402139 -0.833649
H -1.332987 3.922836 2.556801
H -1.782519 2.245156 2.287342

1b-c20, ΔG = 0.9124 kcal/mol, population = 1.73 %
C 0.407950 -3.378319 1.295576
C 0.798949 -3.126613 -0.151067
H 1.864168 -3.322214 -0.304118
C 0.555405 -1.663058 -0.487915
C -0.830693 -1.182769 -0.101072
C -1.575372 -1.825563 0.896010
N -2.789359 -1.392112 1.287543
C -3.262523 -0.311607 0.686057
C -2.588958 0.383802 -0.325075
C -1.338503 -0.058869 -0.752090
C -0.549230 0.591766 -1.856645
H -0.505987 1.672550 -1.710817
H -1.069482 0.430466 -2.804672
C 0.856007 0.001909 -1.927166
C 1.711882 0.321399 -0.676928
C 3.185072 0.536888 -0.964619
C 4.017948 0.768387 0.294045
C 5.501905 0.978096 -0.002543
C 6.330562 1.230324 1.257394
C 7.836918 1.355016 1.008834
C 8.230676 2.565553 0.161610
H 9.315862 2.642468 0.065411
H 7.869343 3.492311 0.616168
H 7.814549 2.506971 -0.845983
H 8.348782 1.413773 1.974085
H 8.196986 0.439441 0.527015
H 5.969876 2.141491 1.748808
H 6.153985 0.411445 1.962777
H 5.895286 0.095830 -0.520687
H 5.611835 1.816707 -0.696804

H 3.897987 -0.084356 0.967793
H 3.626970 1.642885 0.826391
H 3.273979 1.403268 -1.628185
H 3.562962 -0.330454 -1.515056
H 1.300130 1.179744 -0.139469
O 1.542105 -0.859538 0.150684
H 1.357434 0.267834 -2.853780
O 0.757885 -1.436495 -1.874319
C -3.407555 1.514877 -0.753073
O -3.285753 2.239696 -1.722366
C -4.563598 1.662978 0.277857
C -4.179133 2.819460 1.175247
C -3.651664 2.742416 2.390206
H -3.455458 1.798653 2.883686
H -3.388130 3.638361 2.937662
H -4.331547 3.790153 0.711572
C -4.611158 0.295163 0.975388
H -5.394629 -0.305364 0.509140
H -4.823823 0.349604 2.041134
O -5.791270 1.934409 -0.390981
H -5.603753 2.580497 -1.087738
C -1.073725 -3.085512 1.545807
H -1.281594 -3.033385 2.615584
H -1.684238 -3.905612 1.156793
O 0.007128 -3.965757 -0.989776
H 0.202004 -3.733290 -1.906637
H 0.631181 -4.413461 1.555657
H 1.031966 -2.742751 1.926313

1b-c37, ΔG = 0.9457 kcal/mol, population = 1.64 %
C 0.755591 3.193405 -1.664526
C 1.014535 3.172602 -0.167231
H 2.068030 3.373588 0.047540
C 0.700637 1.785707 0.374581
C -0.659239 1.267945 -0.055686
C -1.298823 1.756186 -1.203503
N -2.485496 1.284078 -1.629041
C -3.039817 0.318534 -0.910785
C -2.474124 -0.219168 0.250863
C -1.251420 0.268636 0.713737
C -0.579835 -0.209729 1.972973
H -0.549790 -1.300403 1.999841
H -1.178888 0.101936 2.832772
C 0.828066 0.368555 2.080299

C 1.783945 -0.156091 0.981517
C 3.219640 -0.335422 1.435726
C 4.158916 -0.765162 0.311003
C 5.600908 -0.948673 0.785108
C 6.586984 -1.308871 -0.329702
C 6.338899 -2.671303 -0.979073
C 7.397225 -3.030601 -2.020687
H 7.201223 -4.006058 -2.471124
H 8.393697 -3.063582 -1.572128
H 7.421641 -2.290741 -2.825313
H 6.317069 -3.439339 -0.198048
H 5.352063 -2.684628 -1.449619
H 6.558797 -0.531030 -1.101736
H 7.602888 -1.296520 0.079248
H 5.932079 -0.022543 1.266329
H 5.631836 -1.724311 1.559007
H 4.132836 -0.017422 -0.486793
H 3.784651 -1.697018 -0.122899
H 3.227805 -1.090093 2.229085
H 3.565269 0.602839 1.880970
H 1.402604 -1.086376 0.551788
O 1.716794 0.877001 -0.036253
H 1.237167 0.248232 3.079653
O 0.773506 1.781399 1.791674
C -3.356399 -1.251748 0.782338
O -3.322626 -1.819831 1.856896
C -4.457536 -1.526077 -0.287428
C -4.079836 -2.793639 -1.016984
C -4.798097 -3.908339 -1.017613
H -5.741513 -3.976746 -0.490828
H -4.467540 -4.783802 -1.561571
H -3.140534 -2.745424 -1.560472
C -4.374588 -0.302399 -1.227312
H -5.180846 0.390730 -0.979755
H -4.462719 -0.562040 -2.281489
O -5.725841 -1.596471 0.338961
H -5.626051 -2.141894 1.132785
C -0.708210 2.890935 -1.993344
H -0.829586 2.676860 -3.056189
H -1.323080 3.771936 -1.787587
O 0.178300 4.147437 0.453043
H 0.288093 4.064025 1.408897
H 1.033088 4.168343 -2.066200
H 1.409653 2.452446 -2.127442

1b-c32, ΔG = 0.9500 kcal/mol, population = 1.63 %

C -1.058718 3.122129 1.289686
C -1.283636 2.914551 -0.199332
H -2.347637 2.981907 -0.443746
C -0.818702 1.518182 -0.586327
C 0.577238 1.192034 -0.087486
C 1.138979 1.861500 1.008311
N 2.357374 1.561332 1.495748
C 3.022346 0.587809 0.891599
C 2.540564 -0.123953 -0.213263
C 1.285701 0.184395 -0.739399
C 0.695364 -0.488303 -1.949371
H 0.778978 -1.573158 -1.863824
H 1.278012 -0.204194 -2.829802
C -0.761390 -0.074355 -2.132945
C -1.684285 -0.578299 -0.997282
C -3.081894 -0.951979 -1.451319
C -4.001034 -1.364985 -0.304180
C -5.404428 -1.739669 -0.781778
C -6.328145 -2.254664 0.324842
C -6.670813 -1.220713 1.399062
C -7.652059 -1.755414 2.440667
H -7.240515 -2.629154 2.953002
H -8.593149 -2.058679 1.974169
H -7.881883 -1.002657 3.197836
H -5.757326 -0.890359 1.900998
H -7.094120 -0.332723 0.916930
H -7.259728 -2.609560 -0.128754
H -5.867925 -3.128564 0.800792
H -5.865684 -0.869407 -1.262712
H -5.318813 -2.507954 -1.557559
H -4.054195 -0.548291 0.418282
H -3.558758 -2.219147 0.221611
H -2.992386 -1.776649 -2.166068
H -3.510503 -0.102377 -1.992284
H -1.220526 -1.413732 -0.466068
O -1.747057 0.557003 -0.094054
H -1.133256 -0.340241 -3.118673
O -0.858100 1.358369 -1.995857
C 3.534470 -1.110986 -0.620131
O 3.584063 -1.788341 -1.628654
C 4.629389 -1.159553 0.489193
C 4.356044 -2.372150 1.347588

C 5.181107 -3.400861 1.487177
H 6.142454 -3.429972 0.989980
H 4.922931 -4.240374 2.119768
H 3.400492 -2.360472 1.864139
C 4.405171 0.143259 1.288573
H 5.143183 0.883932 0.974501
H 4.496436 0.009492 2.365685
O 5.914908 -1.171596 -0.104550
H 5.893136 -1.814956 -0.827814
C 0.420712 3.007574 1.664118
H 0.540366 2.922222 2.745143
H 0.948144 3.919782 1.370258
O -0.538849 3.898909 -0.913999
H -0.616549 3.700044 -1.855877
H -1.441321 4.101120 1.579807
H -1.644749 2.372395 1.824131

1b-c19, ΔG = 0.9952 kcal/mol, population = 1.51 %
C -0.096591 -3.843519 -0.953767
C -0.896992 -3.202836 0.168127
H -1.965701 -3.399482 0.043884
C -0.706317 -1.693837 0.120120
C 0.751029 -1.277764 0.043857
C 1.737207 -2.147360 -0.442334
N 3.030730 -1.790420 -0.549854
C 3.347612 -0.560402 -0.172949
C 2.428397 0.367329 0.329901
C 1.086240 0.009241 0.459507
C 0.029100 0.916092 1.029478
H 0.073696 1.899460 0.557893
H 0.235931 1.075185 2.091088
C -1.355283 0.300337 0.852993
C -1.792925 0.196594 -0.629282
C -3.279909 0.391923 -0.859760
C -3.706580 1.855760 -0.739018
C -5.197191 2.087848 -1.001129
C -6.126038 1.442249 0.027708
C -7.598665 1.785620 -0.194425
C -8.525507 1.134936 0.830704
H -8.271300 1.452170 1.845551
H -8.445768 0.045233 0.795078
H -9.569884 1.398838 0.650615
H -7.722104 2.873279 -0.162399
H -7.891828 1.474047 -1.202677

H -6.009940 0.354215 0.008239
H -5.828038 1.764582 1.032510
H -5.385002 3.166239 -1.016105
H -5.450253 1.718199 -2.001100
H -3.121711 2.451536 -1.446814
H -3.458965 2.234761 0.258191
H -3.822796 -0.235841 -0.148985
H -3.521174 0.024056 -1.860978
H -1.215838 0.887582 -1.249274
O -1.424682 -1.160001 -0.987862
H -2.095653 0.796844 1.473606
O -1.304468 -1.084653 1.253617
C 3.123948 1.607505 0.653937
O 2.729547 2.575360 1.275361
C 4.569613 1.499908 0.078604
C 4.608322 2.290703 -1.207727
C 5.364299 3.360486 -1.413788
H 6.036582 3.735541 -0.652502
H 5.343099 3.884249 -2.360770
H 3.947394 1.925623 -1.988641
C 4.749014 -0.010081 -0.194564
H 5.338123 -0.449209 0.612891
H 5.254996 -0.220501 -1.135974
O 5.503681 1.951922 1.042525
H 5.158992 2.777012 1.414006
C 1.401850 -3.564838 -0.814870
H 1.929184 -3.818123 -1.735837
H 1.826357 -4.203819 -0.035118
O -0.430614 -3.720532 1.412615
H -0.885852 -3.244300 2.118773
H -0.275947 -4.919019 -0.957219
H -0.470584 -3.448360 -1.899876

1b-c106, ΔG = 1.0065 kcal/mol, population = 1.48 %
C -1.306673 -2.821876 -1.855323
C -1.412179 -3.044687 -0.355633
H -2.449848 -3.229275 -0.063200
C -0.956471 -1.787134 0.370444
C 0.381809 -1.263985 -0.116098
C 0.868727 -1.574127 -1.393087
N 2.032049 -1.086892 -1.864128
C 2.715786 -0.284725 -1.061381
C 2.307188 0.067038 0.230355
C 1.110730 -0.439437 0.738510

C 0.602553 -0.157054 2.127141
H 0.646425 0.912399 2.340793
H 1.264811 -0.641709 2.849491
C -0.820654 -0.680561 2.292154
C -1.849365 0.066182 1.409451
C -3.218771 0.215067 2.049150
C -4.290578 0.766014 1.106043
C -4.017458 2.188774 0.613135
C -5.150971 2.779563 -0.230265
C -5.383475 2.065552 -1.562915
C -6.460352 2.737735 -2.412950
H -6.609620 2.212850 -3.359034
H -6.190025 3.771748 -2.643097
H -7.418809 2.756035 -1.887193
H -4.440672 2.035814 -2.120119
H -5.665184 1.024667 -1.382845
H -6.079764 2.767924 0.352139
H -4.929866 3.833000 -0.432226
H -3.842625 2.834891 1.480077
H -3.094630 2.208998 0.024242
H -5.251884 0.752063 1.629325
H -4.390455 0.087366 0.257085
H -3.111243 0.875905 2.915650
H -3.526547 -0.764361 2.426707
H -1.454010 1.036308 1.103225
O -1.949726 -0.776803 0.232328
H -1.116922 -0.716015 3.337001
O -0.884460 -2.023529 1.768324
C 3.301817 0.952071 0.825501
O 3.416079 1.323361 1.977733
C 4.297720 1.364204 -0.301057
C 3.916429 2.752425 -0.758678
C 4.693852 3.821808 -0.657074
H 5.691694 3.760938 -0.241209
H 4.357682 4.790301 -1.004341
H 2.921672 2.834600 -1.187360
C 4.045489 0.326024 -1.417299
H 4.830398 -0.431879 -1.381010
H 4.039741 0.760234 -2.416273
O 5.627462 1.277661 0.178518
H 5.642699 1.677630 1.060185
C 0.131903 -2.530128 -2.289305
H 0.156640 -2.142633 -3.308835
H 0.709937 -3.458756 -2.302265

O -0.583748 -4.149869 0.000404
H -0.590025 -4.223581 0.963326
H -1.681387 -3.702116 -2.378473
H -1.958929 -1.986602 -2.116321

1b-c8, ΔG = 1.0586 kcal/mol, population = 1.35 %
C -0.076075 -3.809179 0.915195
C 0.748494 -3.205763 -0.209845
H 1.808928 -3.442584 -0.084109
C 0.616226 -1.690608 -0.169749
C -0.824218 -1.218128 -0.098689
C -1.842095 -2.044857 0.394473
N -3.121504 -1.636539 0.500626
C -3.389147 -0.397989 0.116776
C -2.435365 0.489192 -0.395431
C -1.109821 0.078750 -0.524349
C -0.017698 0.940047 -1.099647
H -0.027052 1.928971 -0.637696
H -0.213858 1.095468 -2.163827
C 1.341354 0.274059 -0.910560
C 1.763288 0.163700 0.576257
C 3.252533 0.317884 0.821048
C 3.720898 1.769283 0.700101
C 5.213219 1.961836 0.982753
C 6.139210 1.293215 -0.033767
C 7.616912 1.598657 0.209330
C 8.540851 0.927033 -0.804854
H 8.307935 1.252351 -1.822264
H 8.433548 -0.160424 -0.772627
H 9.588996 1.164565 -0.610496
H 7.768253 2.682907 0.181870
H 7.888164 1.277436 1.220666
H 5.995175 0.208509 -0.017095
H 5.863515 1.623902 -1.042173
H 5.429167 3.034914 1.001504
H 5.442361 1.584903 1.985794
H 3.142748 2.383809 1.397323
H 3.497412 2.150361 -0.302037
H 3.785547 -0.327178 0.118277
H 3.472879 -0.052445 1.826152
H 1.200596 0.873995 1.187732
O 1.352611 -1.179829 0.937164
H 2.104326 0.738653 -1.528620
O 1.240659 -1.110130 -1.304276

C -3.085258 1.757546 -0.714404
O -2.666073 2.707352 -1.348245
C -4.512374 1.712118 -0.095667
C -4.450533 2.549013 1.163469
C -4.337152 2.106150 2.409007
H -4.297079 1.052036 2.654017
H -4.274097 2.797938 3.239270
H -4.459638 3.617714 0.967835
C -4.765407 0.215233 0.136922
H -5.354385 -0.170971 -0.697208
H -5.306090 -0.003996 1.055408
O -5.464823 2.249697 -1.008178
H -5.079081 3.052118 -1.389334
C -1.562621 -3.471647 0.777486
H -2.096647 -3.696226 1.702095
H -2.014943 -4.099879 0.004652
O 0.263044 -3.711536 -1.451997
H 0.734663 -3.254865 -2.160320
H 0.060306 -4.890974 0.922813
H 0.314809 -3.425720 1.859306

1b-c64, ΔG = 1.1113 kcal/mol, population = 1.24 %
C -0.236437 3.862126 0.866846
C -1.086307 3.146668 -0.170328
H -2.150197 3.324653 0.010489
C -0.853647 1.646519 -0.059612
C 0.615606 1.267749 -0.048617
C 1.606114 2.182129 0.335440
N 2.911675 1.857991 0.388886
C 3.236109 0.616321 0.059583
C 2.312780 -0.355215 -0.343138
C 0.957680 -0.032060 -0.416093
C -0.107486 -0.989011 -0.879079
H -0.011890 -1.946437 -0.363917
H 0.043009 -1.196127 -1.941920
C -1.494499 -0.395883 -0.653921
C -1.850365 -0.227021 0.844106
C -3.316927 -0.438566 1.169881
C -3.719470 -1.913085 1.135594
C -5.178712 -2.160725 1.533546
C -6.223728 -1.467009 0.652875
C -6.163051 -1.854347 -0.824947
C -7.284056 -1.219366 -1.645855
H -7.220394 -1.503287 -2.698494

H -7.237736 -0.128397 -1.591150
H -8.265019 -1.527502 -1.274581
H -5.198459 -1.558200 -1.246882
H -6.213559 -2.945084 -0.912805
H -7.218425 -1.710993 1.040263
H -6.126553 -0.380192 0.742195
H -5.364165 -3.239554 1.518590
H -5.319928 -1.838592 2.570362
H -3.071296 -2.472928 1.816970
H -3.535930 -2.324874 0.139298
H -3.912812 0.148273 0.466215
H -3.507430 -0.030660 2.166440
H -1.224299 -0.875178 1.462829
O -1.491853 1.152618 1.114407
H -2.255187 -0.939635 -1.206750
O -1.500964 0.967680 -1.124451
C 3.018306 -1.596859 -0.639066
O 2.614280 -2.603753 -1.188041
C 4.488760 -1.430530 -0.147028
C 4.610508 -2.147858 1.176782
C 5.397850 -3.191085 1.400757
H 6.036995 -3.596749 0.626754
H 5.436535 -3.661373 2.374883
H 3.983750 -1.751905 1.970658
C 4.648835 0.095502 0.034384
H 5.184486 0.501037 -0.826116
H 5.199012 0.367238 0.934402
O 5.381741 -1.917914 -1.132377
H 5.034285 -2.767014 -1.442124
C 1.257810 3.609947 0.651913
H 1.832280 3.924590 1.524436
H 1.618799 4.214960 -0.184842
O -0.710665 3.610833 -1.465539
H -1.194219 3.087318 -2.117279
H -0.442478 4.932117 0.827206
H -0.543092 3.508020 1.852582

1b-c109, ΔG = 1.1383 kcal/mol, population = 1.18 %
C 1.705169 -2.824408 0.736804
C 1.779296 -2.449441 -0.733853
H 2.819023 -2.334917 -1.053134
C 1.092939 -1.107236 -0.942911
C -0.291090 -1.039247 -0.326292
C -0.665949 -1.885070 0.726486

N -1.875329 -1.818934 1.314298
C -2.717848 -0.906110 0.853308
C -2.427100 -0.029420 -0.198240
C -1.182708 -0.092319 -0.825950
C -0.786270 0.774041 -1.991663
H -1.014617 1.821232 -1.785112
H -1.389167 0.493838 -2.859542
C 0.696578 0.598436 -2.307118
C 1.620119 1.124982 -1.178509
C 2.900365 1.772620 -1.678058
C 3.763333 2.433017 -0.599362
C 4.421593 1.481544 0.401171
C 5.360180 2.210246 1.363219
C 5.951349 1.321145 2.461672
C 6.853872 0.201997 1.940828
H 7.304106 -0.355932 2.764796
H 7.665108 0.608095 1.330138
H 6.300500 -0.509833 1.325565
H 6.523526 1.948239 3.152144
H 5.134764 0.886557 3.048441
H 6.176482 2.671282 0.794429
H 4.812090 3.033956 1.833472
H 3.654684 0.954114 0.972673
H 4.971721 0.713155 -0.150059
H 3.153502 3.163934 -0.055856
H 4.547556 3.008467 -1.101998
H 2.606518 2.539404 -2.401444
H 3.477983 1.021895 -2.226192
H 1.074538 1.820385 -0.534484
O 1.913109 -0.072513 -0.412749
H 0.951295 1.007732 -3.280637
O 1.000994 -0.811071 -2.327336
C -3.583372 0.823112 -0.448545
O -3.802580 1.574633 -1.378994
C -4.597538 0.594642 0.714091
C -4.466539 1.757215 1.669353
C -5.431656 2.625309 1.940215
H -6.413193 2.541668 1.491000
H -5.270446 3.438920 2.635632
H -3.491603 1.855155 2.138382
C -4.119089 -0.718667 1.372243
H -4.757542 -1.537995 1.036267
H -4.149374 -0.690839 2.460763
O -5.903900 0.447136 0.186419

H -6.025871 1.138700 -0.480242
C 0.258202 -2.965211 1.216276
H 0.213160 -2.995825 2.305908
H -0.155805 -3.915666 0.867363
O 1.126830 -3.463530 -1.495755
H 1.107832 -3.173073 -2.416567
H 2.242855 -3.759052 0.898972
H 2.220967 -2.049495 1.306440

1b-c26, ΔG = 1.1716 kcal/mol, population = 1.12 %
C -0.820902 4.105767 -0.915721
C 0.102096 3.702665 0.222458
H 1.081930 4.176117 0.113270
C 0.319806 2.196980 0.180837
C -0.972661 1.407392 0.080847
C -2.144349 1.981435 -0.431649
N -3.291162 1.289998 -0.569597
C -3.273782 0.018390 -0.198375
C -2.151565 -0.629448 0.330559
C -0.959649 0.076729 0.494260
C 0.288264 -0.513058 1.094297
H 0.515180 -1.476814 0.635279
H 0.109046 -0.712533 2.154175
C 1.462326 0.447533 0.935840
C 1.881109 0.661150 -0.539631
C 3.370387 0.863922 -0.747032
C 4.167298 -0.434819 -0.632121
C 5.672030 -0.214407 -0.792057
C 6.498051 -1.502838 -0.805403
C 6.460261 -2.296381 0.502164
C 7.365455 -3.526716 0.470548
H 7.323492 -4.079018 1.411796
H 8.406620 -3.243457 0.294951
H 7.068894 -4.209200 -0.330337
H 6.760029 -1.640131 1.326554
H 5.435169 -2.610366 0.717407
H 6.154165 -2.144239 -1.625276
H 7.539567 -1.251000 -1.031567
H 5.849497 0.330484 -1.724843
H 6.027676 0.435287 0.015516
H 3.821660 -1.139460 -1.396587
H 3.965984 -0.907329 0.333301
H 3.728620 1.598252 -0.018715
H 3.519182 1.299679 -1.738650

H 1.518111 -0.158550 -1.165041
O 1.173375 1.871342 -0.911989
H 2.297774 0.166220 1.570773
O 1.038753 1.769946 1.327477
C -2.493169 -2.015135 0.631323
O -1.869286 -2.846038 1.262833
C -3.894295 -2.298484 0.008033
C -3.672739 -3.043155 -1.287585
C -4.102314 -4.272958 -1.535305
H -4.672913 -4.833070 -0.805281
H -3.907386 -4.750224 -2.487019
H -3.109659 -2.495948 -2.038281
C -4.472673 -0.890825 -0.257767
H -5.175346 -0.639260 0.538978
H -4.996976 -0.812475 -1.209279
O -4.697546 -3.008903 0.932693
H -4.149711 -3.712381 1.310269
C -2.193106 3.437806 -0.801055
H -2.753259 3.543965 -1.731164
H -2.785250 3.938743 -0.029673
O -0.504886 4.080312 1.456619
H 0.048486 3.742110 2.172219
H -0.934400 5.190190 -0.921906
H -0.338685 3.823909 -1.853323

1b-c82, ΔG = 1.1766 kcal/mol, population = 1.11 %
C 0.209381 -3.766489 1.195001
C 0.784786 -3.410661 -0.166122
H 1.843023 -3.681062 -0.222760
C 0.690826 -1.905661 -0.371183
C -0.691569 -1.347397 -0.084744
C -1.593743 -2.019682 0.751276
N -2.809089 -1.526032 1.053909
C -3.130490 -0.355322 0.523726
C -2.295257 0.376033 -0.328733
C -1.038373 -0.128183 -0.664081
C -0.083988 0.556051 -1.605956
H 0.021548 1.610843 -1.345891
H -0.504056 0.528906 -2.614982
C 1.272701 -0.140858 -1.587291
C 1.999728 -0.015524 -0.226141
C 3.506214 0.114103 -0.336414
C 4.206991 0.151416 1.020028
C 5.735329 0.210006 0.922224

C 6.298970 1.410376 0.153818
C 5.901184 2.770729 0.726900
C 6.561627 3.935807 -0.008032
H 7.651229 3.870464 0.051723
H 6.260875 4.897565 0.413119
H 6.288126 3.935111 -1.066628
H 4.814604 2.888299 0.682972
H 6.169123 2.805181 1.788540
H 7.391534 1.335745 0.148611
H 5.992203 1.360307 -0.896132
H 6.148569 0.214080 1.936205
H 6.092909 -0.710660 0.449193
H 3.925694 -0.738644 1.587486
H 3.835005 1.008294 1.590226
H 3.713948 1.031675 -0.894183
H 3.886476 -0.719171 -0.936282
H 1.588645 0.815524 0.353293
O 1.656399 -1.254832 0.447913
H 1.896116 0.169378 -2.421476
O 1.064287 -1.564209 -1.696971
C -2.970515 1.607121 -0.723726
O -2.672212 2.410647 -1.586170
C -4.246001 1.747200 0.161973
C -3.928944 2.732823 1.262278
C -4.546268 3.893201 1.437630
H -5.354572 4.209716 0.790624
H -4.268717 4.555334 2.247657
H -3.125978 2.431189 1.928832
C -4.455194 0.328627 0.736786
H -5.235865 -0.175365 0.163613
H -4.750364 0.327271 1.785276
O -5.347459 2.136086 -0.638222
H -5.043163 2.844434 -1.224086
C -1.265915 -3.376757 1.308891
H -1.597768 -3.417361 2.347388
H -1.881704 -4.098053 0.763763
O 0.036065 -4.093760 -1.169874
H 0.350035 -3.787335 -2.030247
H 0.325516 -4.836816 1.367699
H 0.799129 -3.248833 1.953477

1b-c85, ΔG = 1.1885 kcal/mol, population = 1.09 %

C -0.681870 -3.440904 -1.058132
C -1.199323 -2.904898 0.266291

H -2.290727 -2.962361 0.309083
C -0.819720 -1.436494 0.393958
C 0.648438 -1.171504 0.115344
C 1.418252 -2.052356 -0.656698
N 2.711796 -1.820746 -0.948785
C 3.243935 -0.704595 -0.473210
C 2.549865 0.224077 0.310897
C 1.213019 -0.008654 0.635614
C 0.391623 0.900372 1.509738
H 0.492406 1.937600 1.185440
H 0.785203 0.858079 2.528848
C -1.073135 0.473386 1.498578
C -1.748954 0.654686 0.117964
C -3.209515 1.055641 0.191905
C -3.883798 1.136343 -1.177164
C -5.325560 1.649067 -1.121891
C -6.290236 0.720907 -0.379972
C -7.757257 1.154892 -0.458218
C -8.046780 2.488973 0.231143
H -7.733463 2.460535 1.278560
H -9.113738 2.720939 0.208245
H -7.520022 3.315755 -0.249239
H -8.059117 1.213522 -1.509635
H -8.378629 0.377104 -0.004251
H -6.193457 -0.287626 -0.796587
H -6.001109 0.646444 0.673218
H -5.331003 2.640655 -0.658942
H -5.690363 1.785984 -2.145469
H -3.863421 0.148958 -1.646722
H -3.293856 1.798793 -1.819319
H -3.262386 2.033894 0.681790
H -3.727952 0.343987 0.839363
H -1.189326 1.365719 -0.495606
O -1.628394 -0.660929 -0.484524
H -1.634683 0.944140 2.300923
O -1.142296 -0.955174 1.689425
C 3.444067 1.321647 0.661953
O 3.286984 2.220053 1.466113
C 4.747511 1.153857 -0.178199
C 4.676216 2.125049 -1.332666
C 5.525420 3.123793 -1.532250
H 6.360559 3.300019 -0.866111
H 5.417553 3.786395 -2.381296
H 3.850302 1.959923 -2.018517

C 4.681398 -0.306024 -0.678823
H 5.335183 -0.923572 -0.059864
H 4.990808 -0.420883 -1.716927
O 5.879426 1.350708 0.650259
H 5.713775 2.142472 1.182395
C 0.843088 -3.349334 -1.153261
H 1.176638 -3.509072 -2.179639
H 1.299409 -4.145058 -0.557346
O -0.612680 -3.661129 1.323872
H -0.875958 -3.252729 2.158476
H -1.000499 -4.476763 -1.178180
H -1.147512 -2.862686 -1.858100

1b-c44, ΔG = 1.2205 kcal/mol, population = 1.03 %
C 0.761023 -3.340197 1.075363
C 1.279131 -2.737248 -0.219900
H 2.372512 -2.748670 -0.242845
C 0.840989 -1.282734 -0.305399
C -0.641357 -1.086816 -0.044892
C -1.388008 -2.024741 0.680949
N -2.696425 -1.858734 0.950899
C -3.267047 -0.752311 0.497679
C -2.597785 0.230241 -0.241067
C -1.245020 0.066967 -0.541378
C -0.446637 1.038589 -1.368564
H -0.596182 2.059145 -1.011674
H -0.820407 1.015643 -2.395685
C 1.034053 0.670460 -1.344978
C 1.674356 0.834995 0.055594
C 3.112484 1.328306 0.067378
C 4.092999 0.492172 -0.755603
C 5.499898 1.093260 -0.821315
C 6.226007 1.167910 0.522543
C 7.657511 1.689001 0.400739
C 8.381113 1.772211 1.743632
H 8.439493 0.789421 2.218797
H 7.856161 2.441084 2.430822
H 9.400300 2.147107 1.627027
H 8.219522 1.039668 -0.278901
H 7.639377 2.678971 -0.067476
H 5.673231 1.813163 1.212327
H 6.241321 0.171652 0.980015
H 6.102685 0.495078 -1.512421
H 5.443510 2.097848 -1.256181

H 3.713283 0.380594 -1.774469
H 4.144654 -0.516350 -0.337114
H 3.437223 1.373467 1.109365
H 3.106136 2.361030 -0.298161
H 1.059875 1.495461 0.672019
O 1.603152 -0.503462 0.610213
H 1.586138 1.190227 -2.122758
O 1.159850 -0.746864 -1.580558
C -3.533340 1.295104 -0.584392
O -3.399498 2.221199 -1.360901
C -4.846830 1.044627 0.218534
C -4.845538 1.984556 1.400502
C -5.743232 2.938377 1.607538
H -6.570796 3.096245 0.927432
H -5.683894 3.580913 2.476537
H -4.027864 1.837592 2.100282
C -4.725567 -0.425022 0.679225
H -5.335623 -1.052943 0.026949
H -5.053899 -0.583945 1.705650
O -5.966915 1.215098 -0.631411
H -5.823342 2.027365 -1.138540
C -0.767769 -3.312310 1.147345
H -1.110397 -3.516823 2.162812
H -1.182562 -4.107121 0.520634
O 0.744451 -3.479506 -1.314383
H 1.000141 -3.026806 -2.128298
H 1.118436 -4.366449 1.164761
H 1.190540 -2.773565 1.903392

1b-c46, ΔG = 1.2393 kcal/mol, population = 1.00 %
C 0.036004 3.821565 -0.790518
C 0.916860 3.136167 0.241017
H 1.972063 3.360382 0.060272
C 0.748300 1.628602 0.120102
C -0.703383 1.187535 0.110461
C -1.731911 2.061084 -0.265745
N -3.023699 1.681881 -0.317030
C -3.292549 0.425520 0.002661
C -2.327464 -0.509102 0.395809
C -0.988728 -0.129345 0.469594
C 0.118154 -1.043138 0.922473
H 0.062236 -1.999976 0.400241
H -0.019414 -1.263770 1.984342
C 1.477603 -0.389163 0.696947

C 1.818363 -0.194816 -0.801460
C 3.289966 -0.343956 -1.137232
C 3.755144 -1.800475 -1.112758
C 5.218784 -1.983767 -1.528125
C 6.242865 -1.249165 -0.656217
C 6.219697 -1.649399 0.819179
C 7.319252 -0.966925 1.630971
H 7.283137 -1.261376 2.682028
H 7.219446 0.120897 1.585570
H 8.309081 -1.224309 1.244882
H 5.247472 -1.403390 1.255796
H 6.323905 -2.737035 0.897810
H 7.242286 -1.444978 -1.058573
H 6.095194 -0.167290 -0.736100
H 5.450780 -3.053665 -1.519697
H 5.334248 -1.651902 -2.565115
H 3.123756 -2.385371 -1.788886
H 3.600904 -2.222949 -0.115826
H 3.865422 0.264121 -0.434627
H 3.456401 0.076232 -2.133063
H 1.215278 -0.863016 -1.421676
O 1.403521 1.171267 -1.059374
H 2.263768 -0.903651 1.242188
O 1.427546 0.969883 1.177865
C -2.982226 -1.785678 0.668201
O -2.545559 -2.782895 1.210973
C -4.441592 -1.676310 0.140316
C -4.468290 -2.421231 -1.176792
C -4.422503 -1.890578 -2.392044
H -4.380539 -0.822009 -2.562488
H -4.420593 -2.520863 -3.272163
H -4.483006 -3.501013 -1.057706
C -4.679557 -0.163048 0.028755
H -5.206574 0.172489 0.923999
H -5.272798 0.128608 -0.835491
O -5.348387 -2.265016 1.067396
H -4.949201 -3.091438 1.376836
C -1.445975 3.504012 -0.576770
H -2.033085 3.796373 -1.448732
H -1.833222 4.090478 0.261434
O 0.523761 3.575000 1.539872
H 1.030684 3.068470 2.187237
H 0.195944 4.899131 -0.743615
H 0.357077 3.487881 -1.778828

1b-c53, ΔG = 1.2475 kcal/mol, population = 0.98 %
C -1.326694 -3.010958 -0.943525
C -1.730222 -2.332599 0.355377
H -2.812455 -2.177999 0.391952
C -1.074768 -0.961190 0.428406
C 0.415355 -0.994142 0.147180
C 1.001444 -2.035884 -0.584526
N 2.314900 -2.065762 -0.879144
C 3.050787 -1.053506 -0.444235
C 2.547278 0.020896 0.298582
C 1.190737 0.061676 0.621471
C 0.557871 1.148934 1.447806
H 0.845644 2.132157 1.071060
H 0.945902 1.086112 2.467887
C -0.961511 1.002801 1.460026
C -1.616039 1.256299 0.082375
C -2.997663 1.876935 0.162795
C -3.674848 2.008279 -1.200151
C -5.089149 2.594023 -1.133240
C -6.088171 1.786671 -0.295776
C -6.256023 0.333185 -0.740294
C -7.329961 -0.406062 0.055734
H -8.305241 0.074309 -0.059045
H -7.088959 -0.414193 1.122164
H -7.427839 -1.443534 -0.271253
H -6.505809 0.309522 -1.806722
H -5.304902 -0.196700 -0.637611
H -5.793864 1.805164 0.758589
H -7.061861 2.285619 -0.342255
H -5.036146 3.612844 -0.735101
H -5.475680 2.682844 -2.153794
H -3.702193 1.027831 -1.679556
H -3.057616 2.645633 -1.841681
H -2.901701 2.865128 0.624874
H -3.601799 1.264642 0.837208
H -0.960293 1.851708 -0.557970
O -1.717872 -0.077698 -0.484527
H -1.413584 1.598223 2.248595
O -1.294828 -0.379487 1.703962
C 3.635673 0.938158 0.616321
O 3.656517 1.874861 1.391385
C 4.878624 0.497378 -0.215053
C 4.975681 1.418585 -1.408324

C 5.987669 2.241415 -1.647184
H 6.845141 2.293498 -0.988231
H 5.993642 2.878085 -2.522508
H 4.129294 1.376583 -2.087964
C 4.537803 -0.943512 -0.655962
H 5.063208 -1.646935 -0.007113
H 4.820046 -1.156912 -1.686155
O 6.034642 0.515987 0.602735
H 6.014521 1.339262 1.112184
C 0.187370 -3.218212 -1.031366
H 0.483097 -3.481391 -2.048022
H 0.482641 -4.061441 -0.400210
O -1.299574 -3.144672 1.445995
H -1.474067 -2.657755 2.261575
H -1.838683 -3.970229 -1.024741
H -1.673062 -2.386264 -1.768742

1b-c18, ΔG = 1.2538 kcal/mol, population = 0.97 %
C 1.980155 -2.433127 0.653735
C 2.161906 -1.722499 -0.677327
H 3.198289 -1.398206 -0.805545
C 1.292800 -0.473892 -0.697612
C -0.143662 -0.735011 -0.291573
C -0.495623 -1.843488 0.489857
N -1.758563 -2.071698 0.896608
C -2.676771 -1.191554 0.524853
C -2.410214 -0.060311 -0.255553
C -1.108131 0.186277 -0.692063
C -0.720191 1.353141 -1.560245
H -1.118362 2.282908 -1.149909
H -1.183799 1.229962 -2.542695
C 0.797562 1.439357 -1.710184
C 1.531391 1.806503 -0.397332
C 2.791796 2.626167 -0.617671
C 3.547718 3.032804 0.653047
C 4.121913 1.893312 1.505382
C 5.079119 0.965399 0.760717
C 5.586831 -0.187882 1.625046
C 6.508077 -1.142098 0.867587
H 5.993320 -1.581254 0.008766
H 6.850476 -1.960382 1.504901
H 7.391596 -0.619567 0.491409
H 6.110075 0.213929 2.499297
H 4.726344 -0.743891 2.012406

H 4.573817 0.547322 -0.112915
H 5.931697 1.541804 0.381887
H 4.647026 2.337841 2.357751
H 3.306102 1.297574 1.918945
H 2.880959 3.638349 1.276114
H 4.366056 3.695343 0.352623
H 2.490642 3.539025 -1.140914
H 3.444619 2.076346 -1.300215
H 0.858001 2.318392 0.295184
O 1.858506 0.507151 0.162321
H 1.079399 2.085635 -2.536761
O 1.313562 0.120533 -1.985620
C -3.650456 0.671772 -0.482859
O -3.876823 1.589070 -1.248446
C -4.744246 0.043706 0.434294
C -4.921971 0.955732 1.624889
C -6.044941 1.593030 1.927002
H -6.939724 1.481016 1.327801
H -6.104808 2.235955 2.795703
H -4.038146 1.075711 2.244921
C -4.140076 -1.313227 0.859365
H -4.597103 -2.107267 0.265726
H -4.298860 -1.544289 1.911997
O -5.936393 -0.147244 -0.307434
H -6.091701 0.661151 -0.817291
C 0.530036 -2.875871 0.868564
H 0.364789 -3.167251 1.906906
H 0.322250 -3.764466 0.265471
O 1.777796 -2.611139 -1.724730
H 1.815327 -2.119706 -2.555203
H 2.643627 -3.297322 0.696572
H 2.289437 -1.746529 1.443643

1b-c12, ΔG = 1.2927 kcal/mol, population = 0.91 %
C 0.669336 -3.913677 -1.009789
C -0.184351 -3.500597 0.177607
H -1.195211 -3.908636 0.088270
C -0.309068 -1.984071 0.202794
C 1.025333 -1.271668 0.083941
C 2.138097 -1.893908 -0.496671
N 3.320756 -1.268219 -0.651722
C 3.396074 -0.015734 -0.228904
C 2.337515 0.676086 0.371326
C 1.110842 0.039434 0.551537

C -0.075980 0.678123 1.221681
H -0.259839 1.671693 0.808969
H 0.152399 0.822459 2.280992
C -1.312587 -0.202097 1.070122
C -1.795453 -0.326376 -0.396111
C -3.300695 -0.426320 -0.559026
C -4.013270 0.905697 -0.326972
C -5.528685 0.803822 -0.493610
C -6.245064 2.131502 -0.246706
C -7.757866 2.085333 -0.482962
C -8.510817 1.162771 0.476411
H -8.212548 0.119807 0.353838
H -9.588794 1.217843 0.310022
H -8.318937 1.442448 1.516094
H -8.158669 3.099185 -0.390703
H -7.949501 1.773431 -1.515488
H -6.051007 2.459180 0.781266
H -5.807234 2.893983 -0.899550
H -5.758541 0.452238 -1.505879
H -5.909449 0.040468 0.191107
H -3.620074 1.652999 -1.024678
H -3.790316 1.278132 0.678565
H -3.678464 -1.186790 0.131405
H -3.509414 -0.782192 -1.571492
H -1.402215 0.495495 -1.000095
O -1.179890 -1.561691 -0.842618
H -2.105605 0.102579 1.746975
O -0.958472 -1.562909 1.392270
C 2.782268 2.023754 0.715334
O 2.246598 2.864018 1.412982
C 4.160160 2.245368 0.026507
C 3.892069 3.114411 -1.182652
C 3.797019 2.716845 -2.444862
H 3.927129 1.684605 -2.745494
H 3.575673 3.425028 -3.233176
H 3.727062 4.158395 -0.930611
C 4.645581 0.823849 -0.293182
H 5.341637 0.511015 0.487449
H 5.157697 0.735581 -1.249193
O 5.059061 2.895972 0.920037
H 4.567029 3.605170 1.359168
C 2.083258 -3.334031 -0.925051
H 2.599857 -3.434464 -1.880703
H 2.672724 -3.901233 -0.198771

O 0.441803 -3.964462 1.372406
H -0.061859 -3.620012 2.121013
H 0.715837 -5.001934 -1.059773
H 0.170947 -3.567494 -1.916921

1b-c4, ΔG = 1.3002 kcal/mol, population = 0.90 %
C -0.666916 3.304408 0.849298
C -1.337037 2.462626 -0.224044
H -2.418806 2.425803 -0.067670
C -0.817999 1.034949 -0.137106
C 0.695567 0.948234 -0.092355
C 1.478872 2.026243 0.338807
N 2.821417 1.958090 0.426580
C 3.386464 0.810217 0.085557
C 2.679485 -0.310997 -0.364867
C 1.291137 -0.253553 -0.473668
C 0.440991 -1.386420 -0.984374
H 0.709361 -2.320616 -0.487764
H 0.651149 -1.532445 -2.047270
C -1.040787 -1.077917 -0.786261
C -1.451710 -1.016402 0.704967
C -2.844508 -1.535741 1.024592
C -3.979734 -0.886933 0.234765
C -5.353525 -1.419444 0.638947
C -6.500352 -0.767587 -0.131207
C -7.879661 -1.285866 0.273964
C -9.018242 -0.623340 -0.499578
H -8.917231 -0.800138 -1.573697
H -9.021219 0.458426 -0.341462
H -9.991657 -1.008695 -0.188463
H -7.915189 -2.369816 0.121455
H -8.022799 -1.123006 1.347464
H -6.464695 0.317600 0.019350
H -6.354925 -0.932014 -1.205128
H -5.384244 -2.504122 0.484747
H -5.501698 -1.258283 1.712764
H -3.833724 -1.051477 -0.837000
H -3.949868 0.194850 0.386545
H -3.016281 -1.391546 2.095560
H -2.840087 -2.617797 0.856361
H -0.716228 -1.542186 1.318174
O -1.371299 0.399016 1.010956
H -1.667795 -1.747643 -1.367681
O -1.294643 0.269817 -1.233344

C 3.624477 -1.385167 -0.657410
O 3.448671 -2.435622 -1.245086
C 5.005569 -0.953733 -0.084532
C 5.186020 -1.728676 1.202651
C 4.988549 -1.280562 2.435736
H 4.684660 -0.262698 2.647018
H 5.121820 -1.932012 3.289981
H 5.464633 -2.766496 1.041953
C 4.873318 0.566335 0.090792
H 5.331380 1.056533 -0.770390
H 5.355011 0.951237 0.987329
O 6.043665 -1.267388 -1.007881
H 5.865497 -2.154736 -1.352526
C 0.852692 3.351808 0.672605
H 1.333082 3.749409 1.567883
H 1.109660 4.037050 -0.140477
O -1.031860 3.022791 -1.499956
H -1.382754 2.426267 -2.173605
H -1.076162 4.314769 0.824129
H -0.922664 2.874055 1.819124

1b-c89, ΔG = 1.3033 kcal/mol, population = 0.90 %
C -0.499530 -4.098084 0.891327
C 0.412545 -3.606973 -0.220617
H 1.423873 -4.004513 -0.096216
C 0.511529 -2.089852 -0.153340
C -0.840148 -1.405070 -0.075153
C -1.974300 -2.075847 0.402880
N -3.174867 -1.478500 0.520147
C -3.249636 -0.205406 0.160959
C -2.170120 0.535015 -0.334499
C -0.921795 -0.072345 -0.473605
C 0.290980 0.621577 -1.033006
H 0.429949 1.593697 -0.556710
H 0.123988 0.821328 -2.094793
C 1.532872 -0.246144 -0.855411
C 1.930594 -0.449195 0.627566
C 3.424794 -0.541811 0.872862
C 4.125359 0.814248 0.802081
C 5.635210 0.701379 1.009125
C 6.361365 2.049047 1.074988
C 6.222689 2.930603 -0.172173
C 6.745222 2.280292 -1.452930
H 6.167198 1.393536 -1.721660

H 7.788080 1.972154 -1.337685
H 6.692855 2.973564 -2.295082
H 5.175549 3.215740 -0.309588
H 6.766197 3.863529 0.004371
H 5.992753 2.606138 1.942876
H 7.425191 1.863934 1.257959
H 5.824589 0.158621 1.940728
H 6.061200 0.086618 0.210366
H 3.702241 1.480399 1.562140
H 3.922005 1.284825 -0.164231
H 3.858094 -1.233712 0.143810
H 3.578941 -0.983002 1.861280
H 1.490037 0.330868 1.254014
O 1.309639 -1.716005 0.965473
H 2.359265 0.108972 -1.464801
O 1.223312 -1.591421 -1.275230
C -2.614763 1.890880 -0.635985
O -2.044362 2.773840 -1.247164
C -4.049972 2.056412 -0.049493
C -3.925617 2.815610 1.250393
C -4.459051 4.007008 1.483758
H -5.051693 4.520257 0.736997
H -4.329791 4.497975 2.439707
H -3.341246 2.315498 2.017521
C -4.518070 0.605219 0.200671
H -5.184269 0.302390 -0.609469
H -5.051445 0.480120 1.142157
O -4.883767 2.699417 -0.996658
H -4.384076 3.443613 -1.362822
C -1.916887 -3.535896 0.757045
H -2.484003 -3.694787 1.675420
H -2.455237 -4.072282 -0.029720
O -0.137184 -4.011712 -1.472999
H 0.403870 -3.621916 -2.171552
H -0.529108 -5.187976 0.879642
H -0.059807 -3.794809 1.843006

1b-c24, ΔG = 1.3058 kcal/mol, population = 0.89 %
C 0.644299 -3.079066 2.003641
C 0.747990 -3.381072 0.517695
H 1.733613 -3.786657 0.271960
C 0.582096 -2.088439 -0.267735
C -0.642554 -1.292942 0.143329
C -1.222358 -1.445953 1.409159

N -2.284713 -0.722558 1.812738
C -2.769780 0.160472 0.953626
C -2.257236 0.369698 -0.332087
C -1.168191 -0.380703 -0.771145
C -0.568468 -0.263953 -2.146880
H -0.399555 0.783463 -2.403119
H -1.284782 -0.647778 -2.878240
C 0.733382 -1.054547 -2.228174
C 1.848071 -0.479034 -1.319672
C 3.244127 -0.591295 -1.905441
C 4.364635 -0.210318 -0.935186
C 4.308061 1.234085 -0.437198
C 5.491297 1.600083 0.457407
C 5.435900 3.033130 0.985162
C 6.622437 3.386594 1.880160
H 6.667535 2.724823 2.749130
H 6.556854 4.413627 2.245812
H 7.566175 3.284927 1.337746
H 5.395681 3.726748 0.138573
H 4.502964 3.174383 1.541130
H 5.530059 0.905623 1.304561
H 6.424502 1.455889 -0.099132
H 4.277222 1.915029 -1.295599
H 3.380915 1.400085 0.120397
H 5.322995 -0.376998 -1.436408
H 4.341887 -0.889661 -0.078744
H 3.284304 0.055188 -2.788337
H 3.389534 -1.618523 -2.252188
H 1.621494 0.554800 -1.052490
O 1.751642 -1.291315 -0.120751
H 1.060431 -1.187849 -3.255767
O 0.515897 -2.362655 -1.659100
C -3.039847 1.409220 -0.994977
O -3.052209 1.745762 -2.163839
C -3.944991 2.065472 0.087185
C -3.288617 3.382198 0.442144
C -2.518483 3.628078 1.494290
H -2.295277 2.878746 2.243831
H -2.073936 4.604361 1.639633
H -3.461251 4.159886 -0.296530
C -3.968851 1.032285 1.223554
H -4.882676 0.441308 1.136849
H -3.949713 1.470856 2.219117
O -5.254821 2.281134 -0.428526

H -5.158136 2.624173 -1.329107
C -0.717394 -2.484521 2.371844
H -0.690927 -2.049851 3.372217
H -1.470770 -3.276937 2.404509
O -0.279625 -4.306284 0.167443
H -0.267404 -4.409521 -0.792723
H 0.816433 -3.993901 2.571468
H 1.444429 -2.382381 2.259501

1b-c90, ΔG = 1.3102 kcal/mol, population = 0.89 %
C -0.199862 3.847834 1.641510
C -0.665085 3.770004 0.196770
H -1.665694 4.198060 0.087900
C -0.749114 2.310558 -0.224853
C 0.508425 1.522817 0.091732
C 1.393566 1.931630 1.098922
N 2.492185 1.227239 1.428941
C 2.712690 0.107599 0.755433
C 1.886822 -0.366562 -0.270029
C 0.752289 0.358761 -0.634679
C -0.174057 -0.038797 -1.752530
H -0.439991 -1.094213 -1.672499
H 0.353186 0.072840 -2.703771
C -1.424040 0.835011 -1.742284
C -2.296146 0.623832 -0.479228
C -3.792934 0.734883 -0.706472
C -4.391292 -0.409235 -1.534142
C -4.141559 -1.821262 -0.992245
C -4.640340 -2.054216 0.433650
C -4.460692 -3.498421 0.899021
C -4.946819 -3.731173 2.328372
H -4.404004 -3.097300 3.034592
H -4.805348 -4.769686 2.635345
H -6.010377 -3.496995 2.423502
H -4.996591 -4.165318 0.215534
H -3.402100 -3.769260 0.823487
H -4.114435 -1.391884 1.128853
H -5.700291 -1.781343 0.495543
H -4.634711 -2.534168 -1.660505
H -3.074175 -2.060381 -1.041744
H -4.012320 -0.354379 -2.558949
H -5.469881 -0.241546 -1.604756
H -3.994360 1.688757 -1.201485
H -4.275356 0.781972 0.272330

H -2.044974 -0.324085 -0.000256
O -1.873591 1.699445 0.398998
H -1.988149 0.738311 -2.665386
O -1.023759 2.214876 -1.614008
C 2.433612 -1.614260 -0.790856
O 2.125476 -2.236469 -1.789007
C 3.580777 -2.060380 0.167126
C 3.029137 -3.143510 1.064013
C 3.468898 -4.394118 1.096061
H 4.290439 -4.723426 0.472455
H 3.026658 -5.123649 1.762239
H 2.208962 -2.832627 1.704828
C 3.905503 -0.784566 0.976056
H 4.803569 -0.321633 0.562540
H 4.079824 -0.977613 2.033779
O 4.702740 -2.477165 -0.589987
H 4.375341 -3.048140 -1.300165
C 1.188157 3.230117 1.828256
H 1.402014 3.073749 2.886584
H 1.953350 3.920920 1.462259
O 0.272960 4.466197 -0.621765
H 0.015645 4.332840 -1.543025
H -0.189108 4.889970 1.961971
H -0.933715 3.325530 2.257737

1b-c290, ΔG = 1.3611 kcal/mol, population = 0.81 %
C -0.615614 -3.308068 -0.829888
C -1.246730 -2.508898 0.298206
H -2.334827 -2.481962 0.191542
C -0.749815 -1.072192 0.232338
C 0.759105 -0.963428 0.121953
C 1.535281 -2.017122 -0.376971
N 2.871621 -1.928568 -0.522194
C 3.437689 -0.784186 -0.171262
C 2.738194 0.313400 0.344314
C 1.356893 0.234180 0.512953
C 0.516954 1.339854 1.095113
H 0.750403 2.291826 0.614898
H 0.773811 1.457806 2.151241
C -0.968270 1.016850 0.955477
C -1.447928 0.994940 -0.515998
C -2.861119 1.503833 -0.752277
C -3.947436 0.818405 0.074438
C -5.343460 1.361945 -0.225664

C -6.436480 0.668669 0.588256
C -7.839537 1.249512 0.389510
C -8.387482 1.085783 -1.028777
H -9.411411 1.458942 -1.100680
H -8.394694 0.032796 -1.323980
H -7.787081 1.630351 -1.759964
H -8.522985 0.765200 1.093461
H -7.828652 2.311983 0.656210
H -6.449401 -0.398550 0.337350
H -6.175161 0.730330 1.650038
H -5.368123 2.438001 -0.016808
H -5.546757 1.252180 -1.295152
H -3.739358 0.939687 1.141596
H -3.923861 -0.256432 -0.122217
H -3.084522 1.388417 -1.817195
H -2.861054 2.580595 -0.552621
H -0.749124 1.549668 -1.146388
O -1.363313 -0.409377 -0.869255
H -1.575658 1.660167 1.585511
O -1.184992 -0.347029 1.372034
C 3.682240 1.391283 0.626334
O 3.520264 2.421528 1.252437
C 5.041295 0.995450 -0.020076
C 5.155235 1.809384 -1.290606
C 4.907694 1.394142 -2.526160
H 4.606213 0.378853 -2.752665
H 4.995160 2.071092 -3.366374
H 5.429497 2.845465 -1.112139
C 4.919797 -0.520596 -0.234601
H 5.421619 -1.029682 0.590502
H 5.365913 -0.872689 -1.162521
O 6.115672 1.296173 0.865336
H 5.943265 2.171734 1.241634
C 0.910832 -3.340390 -0.722705
H 1.355317 -3.704836 -1.650073
H 1.212267 -4.045772 0.057179
O -0.877958 -3.103772 1.541236
H -1.208697 -2.534683 2.248002
H -1.011090 -4.324140 -0.818514
H -0.919683 -2.851064 -1.773213

1b-c22, ΔG = 1.3931 kcal/mol, population = 0.77 %
C -0.634222 -3.271616 -1.228001
C -0.999761 -2.961582 0.214158

H -2.075581 -3.076701 0.374583
C -0.650468 -1.512510 0.519394
C 0.763338 -1.140211 0.116828
C 1.456092 -1.854277 -0.869305
N 2.696009 -1.516313 -1.273267
C 3.248178 -0.461078 -0.694342
C 2.629810 0.301245 0.303616
C 1.351648 -0.041142 0.741281
C 0.614217 0.688912 1.831951
H 0.641264 1.765982 1.657130
H 1.129643 0.519014 2.781024
C -0.827301 0.197069 1.926361
C -1.672956 0.548036 0.678307
C -3.128658 0.853189 0.975602
C -3.959623 1.104769 -0.280347
C -5.429834 1.389536 0.021759
C -6.255131 1.648943 -1.238738
C -7.723229 1.997448 -0.974739
C -8.527837 0.865135 -0.335313
H -8.479988 -0.039728 -0.947653
H -9.579347 1.140702 -0.229577
H -8.153040 0.612365 0.658348
H -7.772067 2.886293 -0.336293
H -8.193189 2.276471 -1.922597
H -5.791456 2.467627 -1.799133
H -6.206308 0.766858 -1.888060
H -5.848161 0.543082 0.574410
H -5.504752 2.258532 0.686017
H -3.887071 0.235025 -0.939042
H -3.530403 1.949080 -0.831509
H -3.161974 1.736325 1.622145
H -3.548851 0.019408 1.546868
H -1.216860 1.371877 0.122969
O -1.581184 -0.653557 -0.131585
H -1.299937 0.515018 2.851651
O -0.828286 -1.245406 1.901905
C 3.531710 1.373575 0.713937
O 3.461813 2.124280 1.668629
C 4.703688 1.409703 -0.309162
C 4.436218 2.590682 -1.216174
C 3.918841 2.556427 -2.437471
H 3.644191 1.632732 -2.931652
H 3.744850 3.470408 -2.990939
H 4.670641 3.546053 -0.754796

C 4.637559 0.037728 -0.996480
H 5.375248 -0.619554 -0.532242
H 4.845807 0.067749 -2.064029
O 5.945007 1.573382 0.371217
H 5.812034 2.243973 1.057259
C 0.863082 -3.088437 -1.490561
H 1.067605 -3.072779 -2.562155
H 1.416976 -3.941408 -1.087726
O -0.265959 -3.837084 1.068369
H -0.435480 -3.568325 1.980366
H -0.930676 -4.294130 -1.463849
H -1.215800 -2.608310 -1.870606

1b-c14, ΔG = 1.4313 kcal/mol, population = 0.72 %
C 0.924102 -3.151443 1.203855
C 1.148295 -2.937340 -0.284252
H 2.208754 -3.033503 -0.534445
C 0.722982 -1.524272 -0.656265
C -0.660342 -1.161421 -0.149847
C -1.234345 -1.820083 0.944950
N -2.438491 -1.481652 1.445392
C -3.073105 -0.480930 0.854712
C -2.578353 0.220054 -0.251518
C -1.340361 -0.125655 -0.790018
C -0.734789 0.537731 -1.998399
H -0.788704 1.623910 -1.906275
H -1.326050 0.273809 -2.879351
C 0.709847 0.085498 -2.186310
C 1.644119 0.552145 -1.043333
C 3.050576 0.900998 -1.489661
C 3.975880 1.274205 -0.333773
C 5.389440 1.623002 -0.800693
C 6.321475 2.095556 0.317939
C 6.635232 1.034245 1.374246
C 7.623783 1.526390 2.429812
H 7.834726 0.753425 3.171974
H 7.229018 2.397872 2.958967
H 8.572806 1.819151 1.972776
H 5.712080 0.716026 1.866186
H 7.040036 0.145797 0.877256
H 7.263019 2.435817 -0.126165
H 5.880939 2.971636 0.808333
H 5.831185 0.750386 -1.295523
H 5.325220 2.406769 -1.562893

H 4.007336 0.445228 0.375810
H 3.552241 2.130154 0.204389
H 2.981421 1.738799 -2.191235
H 3.461128 0.050698 -2.043424
H 1.198632 1.390388 -0.500790
O 1.680669 -0.596470 -0.156196
H 1.090144 0.351253 -3.168839
O 0.765238 -1.350823 -2.064108
C -3.542990 1.246788 -0.636003
O -3.593685 1.927878 -1.642579
C -4.595782 1.340863 0.505898
C -4.240147 2.576576 1.303665
C -3.576503 2.618699 2.451897
H -3.229184 1.728100 2.960879
H -3.350630 3.565094 2.926492
H -4.544008 3.500828 0.820052
C -4.435603 0.015409 1.264746
H -5.202723 -0.678916 0.916746
H -4.536769 0.109517 2.344079
O -5.906370 1.456952 -0.039717
H -5.855756 2.076646 -0.782330
C -0.549183 -2.994058 1.586954
H -0.660341 -2.914409 2.669309
H -1.106235 -3.886908 1.288278
O 0.371471 -3.892787 -1.004072
H 0.451765 -3.688347 -1.944539
H 1.277339 -4.144553 1.483177
H 1.535742 -2.425259 1.742050

1b-c70, ΔG = 1.4389 kcal/mol, population = 0.71 %
C 0.124060 -3.829931 0.979226
C 0.866492 -3.232079 -0.204449
H 1.938673 -3.436140 -0.133022
C 0.692514 -1.720489 -0.192130
C -0.754782 -1.286728 -0.049150
C -1.720248 -2.130886 0.516817
N -3.003042 -1.757940 0.682672
C -3.329659 -0.536986 0.285220
C -2.431214 0.365939 -0.294544
C -1.101172 -0.009418 -0.485548
C -0.068956 0.868357 -1.140372
H -0.079410 1.866598 -0.699169
H -0.331799 0.995628 -2.193899
C 1.317371 0.244021 -1.019328

C 1.832272 0.180484 0.440363
C 3.330270 0.372461 0.586923
C 3.756947 1.830155 0.408179
C 5.260002 2.058744 0.590972
C 6.127165 1.378025 -0.470944
C 7.608890 1.760724 -0.402713
C 8.305328 1.319044 0.884943
H 8.208359 0.239288 1.028948
H 7.880264 1.809400 1.762725
H 9.370737 1.557553 0.858102
H 8.126209 1.317218 -1.258722
H 7.704077 2.846187 -0.515495
H 6.039675 0.290394 -0.384310
H 5.737370 1.640126 -1.460654
H 5.455902 3.135892 0.563841
H 5.552728 1.718408 1.588777
H 3.210734 2.446807 1.128922
H 3.462756 2.185891 -0.585155
H 3.832550 -0.275290 -0.135687
H 3.622356 0.027616 1.582918
H 1.292418 0.893472 1.068969
O 1.475900 -1.162101 0.857957
H 2.027919 0.714169 -1.693057
O 1.232999 -1.151617 -1.374513
C -3.133110 1.602147 -0.619466
O -2.764663 2.547080 -1.290215
C -4.547380 1.525833 0.033518
C -4.512429 2.356259 1.294795
C -5.245733 3.440923 1.504883
H -5.951420 3.800099 0.766555
H -5.171181 3.993445 2.432620
H -3.816294 2.007683 2.052371
C -4.723795 0.026656 0.362713
H -5.357278 -0.431829 -0.399107
H -5.181616 -0.150168 1.335145
O -5.526613 1.957320 -0.894521
H -5.191287 2.762935 -1.314288
C -1.377202 -3.539322 0.915733
H -1.853613 -3.756819 1.872889
H -1.851381 -4.198573 0.182880
O 0.327435 -3.782078 -1.404909
H 0.748761 -3.333621 -2.149252
H 0.293456 -4.906729 1.005731
H 0.554106 -3.410495 1.890424

1b-c9, ΔG = 1.4389 kcal/mol, population = 0.71 %
C 0.030825 -3.730063 -1.111569
C -0.588673 -3.319277 0.214274
H -1.646558 -3.594768 0.250099
C -0.510282 -1.806001 0.354474
C 0.877601 -1.251768 0.088904
C 1.809762 -1.953953 -0.686029
N 3.034312 -1.467457 -0.966445
C 3.332208 -0.274675 -0.475212
C 2.465180 0.488063 0.316000
C 1.199660 -0.005829 0.626789
C 0.209321 0.715144 1.501764
H 0.106053 1.755061 1.186530
H 0.594130 0.739991 2.524723
C -1.141681 0.008473 1.469517
C -1.825856 0.065586 0.080799
C -3.337996 0.178885 0.127753
C -3.806203 1.584600 0.509592
C -5.319005 1.697661 0.714884
C -6.150960 1.413971 -0.535882
C -7.648210 1.634083 -0.322316
C -8.479777 1.337579 -1.568951
H -8.354439 0.298736 -1.885472
H -8.176848 1.976290 -2.402897
H -9.543967 1.505498 -1.389755
H -7.989273 1.001448 0.504124
H -7.817873 2.668707 -0.005916
H -5.803957 2.057254 -1.353222
H -5.989026 0.383390 -0.866741
H -5.625235 1.014406 1.515040
H -5.548866 2.707473 1.069527
H -3.490059 2.291130 -0.265564
H -3.305200 1.897678 1.430859
H -3.723720 -0.557207 0.840633
H -3.725590 -0.094564 -0.855374
H -1.402737 0.873844 -0.521449
O -1.454388 -1.200045 -0.522724
H -1.792026 0.350435 2.269745
O -0.929914 -1.407046 1.650162
C 3.127302 1.737157 0.681753
O 2.809130 2.572711 1.506510
C 4.413130 1.848600 -0.187358
C 4.089902 2.832902 -1.290541

C 3.746892 2.543479 -2.539208
H 3.683992 1.527296 -2.908352
H 3.507967 3.329295 -3.244387
H 4.112503 3.868136 -0.961634
C 4.662082 0.409278 -0.661982
H 5.407827 -0.047854 -0.008808
H 5.027211 0.338253 -1.684556
O 5.502824 2.318087 0.600172
H 5.167212 3.033288 1.160406
C 1.506639 -3.334581 -1.198096
H 1.868172 -3.413896 -2.224443
H 2.110908 -4.029441 -0.607690
O 0.132624 -3.951751 1.269895
H -0.210541 -3.611072 2.105882
H -0.073592 -4.807725 -1.240791
H -0.538639 -3.250161 -1.909397

1b-c71, ΔG = 1.4539 kcal/mol, population = 0.69 %
C -1.117395 -2.783784 -1.931747
C -1.215183 -3.073801 -0.443153
H -2.243366 -3.321261 -0.163887
C -0.823235 -1.827023 0.336482
C 0.491460 -1.222891 -0.121765
C 0.995899 -1.457008 -1.407436
N 2.138739 -0.898988 -1.851656
C 2.781077 -0.100802 -1.013088
C 2.351977 0.179642 0.289432
C 1.179337 -0.400370 0.769980
C 0.652992 -0.197990 2.165612
H 0.645377 0.862993 2.421852
H 1.333871 -0.679967 2.872275
C -0.745014 -0.793075 2.301034
C -1.804244 -0.059817 1.443164
C -3.180696 0.001381 2.081697
C -4.273572 0.541434 1.156669
C -4.059975 1.991349 0.716468
C -5.212673 2.562146 -0.114556
C -5.398005 1.893570 -1.477998
C -6.496205 2.549756 -2.313090
H -6.612642 2.056511 -3.280568
H -6.270713 3.603120 -2.499661
H -7.459406 2.504383 -1.797764
H -4.449067 1.930798 -2.024116
H -5.632440 0.834148 -1.343978

H -6.145440 2.482966 0.456026
H -5.039868 3.632241 -0.271074
H -3.918601 2.613236 1.606926
H -3.135596 2.072797 0.135453
H -5.233930 0.468661 1.676815
H -4.344562 -0.109619 0.283415
H -3.105337 0.630341 2.974947
H -3.445169 -1.005530 2.417473
H -1.452632 0.938668 1.177295
O -1.862474 -0.859604 0.233713
H -1.043595 -0.883918 3.341904
O -0.745038 -2.115740 1.724054
C 3.304930 1.088207 0.920763
O 3.409151 1.416298 2.087479
C 4.260372 1.603028 -0.194868
C 3.800118 3.005384 -0.529253
C 3.044120 3.370403 -1.556706
H 2.686265 2.667368 -2.298592
H 2.747825 4.403359 -1.687389
H 4.110932 3.744554 0.203833
C 4.087218 0.581137 -1.328389
H 4.904157 -0.140862 -1.273176
H 4.097871 1.020038 -2.324053
O 5.605267 1.615521 0.273950
H 5.593456 1.971108 1.174702
C 0.306189 -2.405655 -2.347935
H 0.313131 -1.971842 -3.348973
H 0.927472 -3.304491 -2.401263
O -0.334924 -4.151440 -0.129584
H -0.343409 -4.268518 0.828982
H -1.448777 -3.658300 -2.492431
H -1.808402 -1.970393 -2.159755

1b-c128, ΔG = 1.4891 kcal/mol, population = 0.65 %
C 0.699724 3.354141 -1.802692
C 0.915000 3.479852 -0.303482
H 1.929116 3.826234 -0.084716
C 0.757126 2.110173 0.340090
C -0.516886 1.396985 -0.075396
C -1.175959 1.711257 -1.271979
N -2.281469 1.062887 -1.684051
C -2.735103 0.093856 -0.902923
C -2.145408 -0.276392 0.311295
C -1.007581 0.394987 0.759726

C -0.323783 0.103091 2.067840
H -0.166476 -0.970501 2.186361
H -0.981110 0.409169 2.886107
C 1.002151 0.852685 2.154242
C 2.041186 0.362891 1.116129
C 3.474538 0.385993 1.614902
C 4.516321 0.051409 0.544793
C 4.384248 -1.353585 -0.043909
C 5.510464 -1.691830 -1.021318
C 5.361418 -3.052175 -1.709063
C 5.427916 -4.244757 -0.754336
H 5.385185 -5.189199 -1.301114
H 4.600770 -4.239970 -0.041872
H 6.359146 -4.232729 -0.181027
H 4.412387 -3.074900 -2.255780
H 6.149386 -3.153329 -2.461485
H 5.556396 -0.910738 -1.787668
H 6.469638 -1.658287 -0.491606
H 4.366568 -2.081741 0.772943
H 3.426940 -1.452872 -0.565990
H 5.510431 0.158059 0.989691
H 4.456864 0.790932 -0.258421
H 3.550257 -0.331610 2.438558
H 3.675196 1.377070 2.031952
H 1.771970 -0.630447 0.752506
O 1.888053 1.305535 0.023223
H 1.395832 0.858477 3.167078
O 0.788144 2.222209 1.754530
C -2.908878 -1.369434 0.903007
O -2.842990 -1.842697 2.021096
C -3.924395 -1.866892 -0.170382
C -3.352362 -3.119475 -0.791803
C -3.915018 -4.318594 -0.729346
H -4.859840 -4.476585 -0.224958
H -3.451013 -5.177247 -1.197323
H -2.408082 -2.981388 -1.310693
C -3.971006 -0.714397 -1.198015
H -4.865020 -0.114320 -1.017680
H -3.995957 -1.058289 -2.231211
O -5.194053 -2.065485 0.424341
H -5.051765 -2.533257 1.260149
C -0.705369 2.847140 -2.136917
H -0.766744 2.539343 -3.181772
H -1.429917 3.657570 -2.014896

O -0.051878 4.387662 0.221307
H 0.037877 4.389733 1.182879
H 0.867646 4.322701 -2.274544
H 1.453232 2.667329 -2.192063

1b-c119, ΔG = 1.4916 kcal/mol, population = 0.65 %
C 1.240402 -3.075416 0.815843
C 1.771053 -2.167371 -0.281242
H 2.844997 -1.999360 -0.160157
C 1.087351 -0.811493 -0.184086
C -0.424006 -0.905922 -0.090831
C -1.058928 -2.066715 0.370683
N -2.395682 -2.159563 0.501984
C -3.106233 -1.089983 0.176133
C -2.553178 0.104834 -0.299653
C -1.171250 0.211784 -0.457608
C -0.481052 1.432141 -1.005973
H -0.844744 2.333861 -0.510159
H -0.740942 1.537351 -2.062784
C 1.032266 1.305945 -0.852954
C 1.491240 1.309964 0.625680
C 2.818176 1.999118 0.900664
C 3.997325 1.487368 0.076106
C 5.313255 2.158629 0.467036
C 6.513995 1.712552 -0.374498
C 6.810501 0.208039 -0.342638
C 7.076120 -0.342174 1.058255
H 6.196129 -0.253613 1.698517
H 7.893491 0.198639 1.543391
H 7.350506 -1.398511 1.020277
H 5.980937 -0.341431 -0.796965
H 7.681120 0.014830 -0.976387
H 6.350876 2.017142 -1.413818
H 7.402142 2.254061 -0.031403
H 5.204529 3.244099 0.372249
H 5.511627 1.968399 1.526468
H 3.813390 1.653630 -0.989953
H 4.075134 0.407291 0.209345
H 3.040277 1.880882 1.965618
H 2.675349 3.071725 0.732715
H 0.714445 1.746285 1.257919
O 1.595338 -0.102283 0.941629
H 1.556090 2.040325 -1.457881
O 1.433348 -0.005564 -1.299538

C -3.624046 1.060598 -0.558443
O -3.586822 2.124580 -1.145871
C -4.936000 0.468340 0.041035
C -5.174922 1.145249 1.370392
C -6.217557 1.915729 1.649018
H -6.998061 2.100576 0.921622
H -6.327972 2.372966 2.623874
H -4.407744 0.966755 2.118434
C -4.609857 -1.029047 0.236332
H -5.042503 -1.596867 -0.589691
H -4.997603 -1.437412 1.168688
O -6.002085 0.640389 -0.874629
H -5.943210 1.543638 -1.218696
C -0.267741 -3.304894 0.687786
H -0.669298 -3.752794 1.597918
H -0.465582 -4.019304 -0.116643
O 1.495435 -2.770135 -1.544389
H 1.750523 -2.141163 -2.231477
H 1.765647 -4.030305 0.779466
H 1.474574 -2.612572 1.776153

1b-c100, ΔG = 1.5035 kcal/mol, population = 0.64 %
C -1.024166 -4.032858 1.224512
C -0.223620 -3.842318 -0.053570
H 0.704392 -4.420220 -0.019886
C 0.161504 -2.376184 -0.188368
C -1.012932 -1.430283 -0.011660
C -2.161299 -1.813337 0.695319
N -3.199706 -0.981172 0.899268
C -3.097137 0.241174 0.398748
C -1.992934 0.701123 -0.327913
C -0.914583 -0.152813 -0.560412
C 0.298476 0.229689 -1.365112
H 0.685231 1.196536 -1.038062
H 0.004620 0.353842 -2.410806
C 1.373256 -0.845081 -1.246826
C 1.946182 -0.970637 0.187029
C 3.425228 -1.310216 0.246489
C 4.347807 -0.159283 -0.169802
C 4.326926 1.040551 0.780542
C 5.258722 2.181987 0.361951
C 6.748454 1.837162 0.400232
C 7.638013 3.031861 0.060192
H 7.420592 3.408347 -0.942955

H 8.696546 2.765090 0.093595
H 7.477693 3.853322 0.763531
H 7.001931 1.462587 1.398005
H 6.960940 1.022016 -0.296603
H 4.991376 2.514187 -0.648129
H 5.082177 3.038718 1.020697
H 3.310409 1.436645 0.851469
H 4.595472 0.703955 1.788365
H 4.091570 0.178738 -1.180012
H 5.363333 -0.554756 -0.235944
H 3.597945 -2.179029 -0.394740
H 3.661397 -1.617262 1.269140
H 1.727328 -0.070883 0.764115
O 1.183782 -2.067339 0.753775
H 2.147712 -0.721800 -1.998527
O 0.764589 -2.137082 -1.450434
C -2.223022 2.084404 -0.728972
O -1.605336 2.774722 -1.516599
C -3.490979 2.587245 0.026556
C -3.023304 3.417969 1.198467
C -3.286726 4.707094 1.363488
H -3.887641 5.259244 0.651952
H -2.919714 5.244809 2.228219
H -2.425696 2.880848 1.929360
C -4.173740 1.288192 0.509844
H -5.000786 1.050630 -0.162100
H -4.569481 1.360065 1.522118
O -4.330234 3.298733 -0.864909
H -3.764668 3.888186 -1.384846
C -2.317613 -3.215322 1.214505
H -2.754601 -3.171406 2.213217
H -3.060499 -3.705264 0.578342
O -1.028722 -4.246090 -1.159536
H -0.543647 -4.038193 -1.968321
H -1.254031 -5.091000 1.352559
H -0.392274 -3.733522 2.062521

1b-c35, ΔG = 1.5324 kcal/mol, population = 0.61 %
C 1.366044 -4.161224 -0.769387
C 0.325142 -3.872905 0.299489
H -0.552087 -4.512976 0.168981
C -0.143500 -2.431478 0.166420
C 0.999064 -1.435495 0.094011
C 2.278619 -1.819777 -0.330495

N 3.298507 -0.948151 -0.441362
C 3.045966 0.314659 -0.129819
C 1.802415 0.779936 0.312417
C 0.738507 -0.112542 0.446043
C -0.623238 0.277159 0.954530
H -0.977827 1.175929 0.446958
H -0.543928 0.534514 2.014083
C -1.607322 -0.873262 0.768872
C -1.875663 -1.199535 -0.725658
C -3.294112 -1.628668 -1.067008
C -4.251654 -0.466205 -1.344905
C -4.494839 0.483882 -0.172132
C -5.549511 1.547235 -0.473585
C -5.778709 2.514814 0.686573
C -6.831866 3.578130 0.380721
H -6.974084 4.255079 1.225836
H -7.798100 3.119580 0.154359
H -6.540785 4.180368 -0.483992
H -6.078337 1.946701 1.573586
H -4.830595 3.000697 0.940190
H -5.247787 2.113740 -1.362073
H -6.495658 1.056820 -0.729039
H -4.805116 -0.092403 0.706862
H -3.563622 0.989636 0.102217
H -5.211029 -0.885757 -1.661527
H -3.873567 0.107964 -2.197917
H -3.678935 -2.262882 -0.262179
H -3.234015 -2.254303 -1.960417
H -1.575259 -0.358566 -1.357129
O -0.974153 -2.307526 -0.982146
H -2.518425 -0.721095 1.337091
O -0.994704 -2.087469 1.248209
C 1.888469 2.212713 0.570874
O 1.098762 2.945564 1.134649
C 3.255909 2.709849 0.010578
C 2.989838 3.372630 -1.320632
C 3.224757 4.650146 -1.587367
H 3.651644 5.314811 -0.846969
H 3.010173 5.064610 -2.563956
H 2.569231 2.720338 -2.080600
C 4.075573 1.412841 -0.171878
H 4.769565 1.311086 0.664696
H 4.652915 1.394228 -1.095335
O 3.874396 3.570086 0.950410

H 3.195246 4.180352 1.272842
C 2.594856 -3.259241 -0.627810
H 3.211630 -3.302769 -1.526714
H 3.225124 -3.618212 0.191063
O 0.916838 -4.083384 1.580136
H 0.274922 -3.812890 2.249068
H 1.664738 -5.208170 -0.709575
H 0.895713 -4.012494 -1.742949

1b-c25, ΔG = 1.5349 kcal/mol, population = 0.61 %
C -1.007137 -3.098188 -0.969721
C -1.621622 -2.223248 0.110487
H -2.694130 -2.093100 -0.060029
C -0.983961 -0.842654 0.059220
C 0.532518 -0.883105 0.040780
C 1.229940 -2.014611 -0.400711
N 2.574933 -2.059495 -0.463992
C 3.228937 -0.971626 -0.087172
C 2.610932 0.195928 0.375998
C 1.221056 0.255273 0.459070
C 0.461021 1.446094 0.979738
H 0.817968 2.363655 0.508503
H 0.662138 1.551868 2.049142
C -1.036892 1.268745 0.747403
C -1.418842 1.270312 -0.753573
C -2.750692 1.919796 -1.093432
C -3.951719 1.381418 -0.318534
C -5.267007 2.017504 -0.769978
C -6.481102 1.596805 0.062261
C -6.818533 0.106886 -0.015580
C -8.083479 -0.252628 0.761987
H -7.977230 0.001670 1.820027
H -8.304215 -1.320258 0.696502
H -8.949478 0.292140 0.376654
H -6.939613 -0.177453 -1.066681
H -5.981376 -0.483343 0.366811
H -6.312570 1.872368 1.110033
H -7.353038 2.171576 -0.267804
H -5.168184 3.107292 -0.725408
H -5.445381 1.768811 -1.822476
H -3.819696 1.563852 0.752654
H -3.995575 0.298649 -0.443696
H -2.919532 1.789872 -2.166792
H -2.646340 2.996930 -0.926114

H -0.624246 1.735342 -1.341593
O -1.462905 -0.141404 -1.083627
H -1.616818 1.978733 1.329800
O -1.413444 -0.060214 1.162729
C 3.637110 1.179012 0.710935
O 3.538484 2.228087 1.318862
C 4.988806 0.643489 0.156512
C 5.267209 1.431437 -1.104853
C 5.072739 1.030530 -2.354552
H 4.696731 0.045554 -2.602172
H 5.282889 1.690347 -3.186676
H 5.621694 2.439775 -0.909419
C 4.731230 -0.855009 -0.060146
H 5.128714 -1.402721 0.796429
H 5.196265 -1.258596 -0.957313
O 6.027386 0.844000 1.110938
H 5.914214 1.732808 1.478597
C 0.500421 -3.275622 -0.772366
H 0.960365 -3.694520 -1.668661
H 0.686161 -3.995423 0.030107
O -1.383897 -2.830666 1.379089
H -1.693601 -2.219494 2.059730
H -1.499151 -4.071315 -0.969381
H -1.211554 -2.631155 -1.934748

1b-c40, ΔG = 1.5437 kcal/mol, population = 0.60 %
C 0.230147 3.762309 -0.853004
C 1.005352 3.054112 0.245832
H 2.081455 3.203372 0.120056
C 0.743502 1.557675 0.158954
C -0.731214 1.210197 0.075287
C -1.676742 2.134020 -0.389944
N -2.983255 1.834654 -0.515806
C -3.353221 0.608085 -0.177779
C -2.477310 -0.370462 0.305956
C -1.122591 -0.072176 0.455241
C -0.107610 -1.036970 1.007348
H -0.199531 -2.009612 0.520837
H -0.318805 -1.202650 2.067192
C 1.303416 -0.481747 0.837301
C 1.726641 -0.363659 -0.648592
C 3.187293 -0.669115 -0.937716
C 4.193403 0.170165 -0.149821
C 5.651900 -0.165592 -0.476116

C 6.089587 -1.561542 -0.032135
C 7.571171 -1.839449 -0.284362
C 8.002877 -3.232901 0.168732
H 7.828785 -3.367439 1.239596
H 9.064671 -3.404712 -0.020727
H 7.440143 -4.008277 -0.357836
H 7.782388 -1.719549 -1.352358
H 8.171509 -1.083558 0.233000
H 5.877780 -1.678868 1.037315
H 5.495498 -2.323181 -0.546945
H 5.817183 -0.058307 -1.554322
H 6.297810 0.574252 0.007574
H 4.030795 0.038997 0.924320
H 4.008746 1.225326 -0.361174
H 3.357755 -0.528184 -2.009541
H 3.334063 -1.734529 -0.740443
H 1.092205 -1.001229 -1.268869
O 1.430856 1.020150 -0.966450
H 2.023741 -1.030049 1.437562
O 1.314370 0.892477 1.274595
C -3.225848 -1.589877 0.587799
O -2.877583 -2.590449 1.184574
C -4.659965 -1.407365 0.002551
C -4.718087 -2.159477 -1.306184
C -5.517637 -3.188706 -1.550735
H -6.214689 -3.555033 -0.807713
H -5.508100 -3.685934 -2.512115
H -4.032981 -1.802115 -2.069644
C -4.775340 0.115829 -0.229246
H -5.356119 0.555721 0.583762
H -5.260965 0.372908 -1.169789
O -5.621221 -1.847905 0.944928
H -5.313812 -2.696657 1.295216
C -1.279161 3.546528 -0.716757
H -1.797372 3.854328 -1.626074
H -1.671792 4.177302 0.086151
O 0.571151 3.558884 1.507320
H 1.003709 3.038573 2.196459
H 0.457215 4.828461 -0.825779
H 0.583307 3.378920 -1.811884

1b-c309, ΔG = 1.5612 kcal/mol, population = 0.58 %
C 0.684029 -3.442443 0.786192
C 1.317886 -2.584352 -0.296136

H 2.403253 -2.540759 -0.169418
C 0.790969 -1.161699 -0.179333
C -0.721754 -1.087251 -0.091228
C -1.485220 -2.177337 0.348314
N -2.824491 -2.121890 0.472882
C -3.409155 -0.974587 0.161085
C -2.723578 0.158321 -0.292645
C -1.336962 0.112814 -0.441563
C -0.511029 1.257777 -0.964212
H -0.772876 2.184873 -0.451206
H -0.753001 1.412586 -2.019118
C 0.978130 0.959532 -0.811624
C 1.432190 0.885959 0.666320
C 2.829563 1.413574 0.950325
C 3.946060 0.787800 0.116427
C 5.325405 1.332880 0.483882
C 6.450009 0.711857 -0.344913
C 7.856875 1.169656 0.052100
C 8.117241 2.658653 -0.178550
H 7.478372 3.283949 0.447917
H 7.926613 2.929627 -1.220855
H 9.153841 2.916551 0.048805
H 8.028016 0.927728 1.106681
H 8.588295 0.588125 -0.517117
H 6.392996 -0.377847 -0.252142
H 6.283971 0.938306 -1.404637
H 5.325315 2.419847 0.358137
H 5.517231 1.146287 1.546944
H 3.764238 0.965480 -0.947786
H 3.933088 -0.296297 0.253524
H 3.035836 1.256339 2.013344
H 2.809756 2.497752 0.797628
H 0.710713 1.399305 1.306104
O 1.371343 -0.533368 0.959514
H 1.582699 1.640591 -1.403466
O 1.230466 -0.381264 -1.280074
C -3.679986 1.229953 -0.546215
O -3.517910 2.292607 -1.114548
C -5.057495 0.776227 0.027480
C -5.249110 1.465954 1.357514
C -6.227140 2.318322 1.632221
H -6.984062 2.569187 0.899869
H -6.307176 2.779131 2.608344
H -4.505963 1.220852 2.110816

C -4.896894 -0.748976 0.214863
H -5.386208 -1.262417 -0.615111
H -5.331049 -1.116920 1.143616
O -6.083279 1.064207 -0.905474
H -5.937164 1.966302 -1.225567
C -0.839187 -3.501109 0.649039
H -1.292796 -3.914110 1.551241
H -1.111497 -4.178049 -0.165971
O 0.981691 -3.133302 -1.569096
H 1.311289 -2.528010 -2.245683
H 1.100720 -4.448973 0.740138
H 0.961660 -3.019273 1.753136

1b-c34, ΔG = 1.5732 kcal/mol, population = 0.57 %
C 1.584943 -2.636168 1.381594
C 1.638064 -2.641221 -0.137127
H 2.673695 -2.652075 -0.488629
C 0.995400 -1.366550 -0.663668
C -0.374563 -1.099514 -0.068188
C -0.755201 -1.642955 1.166676
N -1.951784 -1.394467 1.731009
C -2.775951 -0.598959 1.065250
C -2.477821 -0.019473 -0.173249
C -1.246638 -0.276352 -0.777555
C -0.844795 0.263524 -2.123490
H -1.038605 1.336221 -2.178554
H -1.469135 -0.201046 -2.891346
C 0.626606 -0.032611 -2.399027
C 1.586288 0.720930 -1.441829
C 2.872777 1.188217 -2.100242
C 3.814698 1.995589 -1.201403
C 4.552549 1.191863 -0.129682
C 5.545947 2.035996 0.666829
C 6.288178 1.240913 1.740266
C 7.276460 2.088065 2.539738
H 6.765668 2.907916 3.051808
H 8.034169 2.527967 1.885776
H 7.793705 1.493692 3.296149
H 5.558194 0.789693 2.420728
H 6.819181 0.408402 1.266399
H 6.274746 2.486989 -0.016947
H 5.014884 2.870146 1.140477
H 3.832750 0.735992 0.552247
H 5.084772 0.362432 -0.609484

H 3.246948 2.803428 -0.725512
H 4.556130 2.483815 -1.841920
H 2.579438 1.814716 -2.948228
H 3.391273 0.316871 -2.512572
H 1.073933 1.568924 -0.978871
O 1.858386 -0.264430 -0.411941
H 0.877292 0.118707 -3.445275
O 0.888263 -1.414400 -2.077856
C -3.612691 0.782776 -0.615580
O -3.824444 1.289610 -1.700433
C -4.614726 0.879441 0.575713
C -4.440756 2.238115 1.211937
C -5.384998 3.166661 1.279769
H -6.378294 2.995875 0.884258
H -5.193750 4.123113 1.749014
H -3.453290 2.427118 1.623296
C -4.162038 -0.244506 1.534492
H -4.829392 -1.100482 1.417490
H -4.173071 0.052478 2.582457
O -5.932127 0.642848 0.111436
H -6.048698 1.157056 -0.700704
C 0.146160 -2.594376 1.903452
H 0.127746 -2.341242 2.964613
H -0.307594 -3.586158 1.819714
O 0.931621 -3.784744 -0.614495
H 0.895364 -3.727009 -1.577845
H 2.092616 -3.522296 1.763613
H 2.142176 -1.765897 1.732515

1b-c10, ΔG = 1.5738 kcal/mol, population = 0.57 %
C 1.049522 4.017119 0.844473
C 0.108142 3.653875 -0.292303
H -0.844286 4.181906 -0.190892
C -0.192705 2.163099 -0.235226
C 1.054337 1.304767 -0.126376
C 2.254251 1.818184 0.382487
N 3.363105 1.066513 0.524936
C 3.276473 -0.203893 0.162427
C 2.121124 -0.794104 -0.362852
C 0.969575 -0.027332 -0.530631
C -0.308887 -0.553670 -1.125759
H -0.587626 -1.499683 -0.658157
H -0.142368 -0.770749 -2.184236
C -1.428242 0.470729 -0.973101

C -1.828709 0.718033 0.502755
C -3.306158 0.987813 0.717923
C -4.159102 -0.275497 0.610468
C -5.650846 0.006708 0.794898
C -6.531050 -1.245460 0.801366
C -6.548215 -2.015914 -0.520199
C -7.506332 -3.205659 -0.496960
H -7.501525 -3.742114 -1.448270
H -8.531659 -2.880572 -0.301670
H -7.229320 -3.915046 0.287393
H -6.830610 -1.331734 -1.327855
H -5.541371 -2.370715 -0.756887
H -6.201876 -1.915258 1.604381
H -7.557044 -0.953350 1.049084
H -5.791861 0.545304 1.737526
H -5.989409 0.682442 0.001477
H -3.831825 -0.997698 1.366624
H -3.992346 -0.751075 -0.359917
H -3.636257 1.735087 -0.010480
H -3.430152 1.431722 1.709324
H -1.498775 -0.111563 1.133509
O -1.064643 1.897886 0.859471
H -2.279957 0.230487 -1.603262
O -0.933914 1.764300 -1.377877
C 2.392171 -2.199159 -0.653501
O 1.733479 -3.002559 -1.286256
C 3.763850 -2.548201 -0.006166
C 3.448685 -3.319403 1.257140
C 3.447660 -2.848915 2.497728
H 3.705122 -1.824148 2.735385
H 3.178917 -3.485287 3.331239
H 3.157098 -4.348911 1.068772
C 4.422974 -1.179815 0.221099
H 5.114598 -0.986469 -0.601081
H 4.983896 -1.108359 1.150694
O 4.541805 -3.342530 -0.896676
H 3.950033 -4.001939 -1.287847
C 2.382558 3.272112 0.742147
H 2.942619 3.353333 1.674914
H 3.006415 3.733805 -0.028758
O 0.738060 3.984071 -1.528559
H 0.168420 3.669653 -2.242229
H 1.223559 5.093513 0.839344
H 0.549337 3.772940 1.783253

1b-c38, ΔG = 1.5845 kcal/mol, population = 0.56 %
C 1.598433 -2.834639 0.922690
C 1.642620 -2.595450 -0.577374
H 2.676143 -2.550409 -0.932335
C 0.999063 -1.252207 -0.888174
C -0.366892 -1.083353 -0.249162
C -0.741232 -1.818065 0.884488
N -1.934012 -1.662591 1.488739
C -2.760670 -0.769692 0.964644
C -2.468812 0.001218 -0.166323
C -1.241657 -0.156098 -0.811555
C -0.846773 0.592928 -2.055552
H -1.038932 1.660675 -1.936137
H -1.476499 0.258491 -2.884299
C 0.622426 0.343720 -2.384179
C 1.589174 0.932736 -1.324255
C 2.872661 1.498462 -1.906951
C 3.821505 2.149960 -0.895901
C 4.564342 1.183699 0.027968
C 5.563988 1.887910 0.943721
C 6.311567 0.929860 1.870329
C 7.306038 1.636592 2.789606
H 7.827067 0.927808 3.437010
H 6.799659 2.363417 3.430373
H 8.060341 2.175793 2.210304
H 5.585089 0.375274 2.473796
H 6.838378 0.184299 1.265188
H 6.289265 2.442731 0.336894
H 5.037202 2.635186 1.548907
H 3.847837 0.624614 0.632117
H 5.092212 0.441857 -0.582322
H 3.258159 2.871244 -0.292739
H 4.559948 2.734197 -1.454214
H 2.575527 2.253383 -2.641409
H 3.386971 0.704185 -2.457138
H 1.081230 1.695829 -0.727930
O 1.865430 -0.205565 -0.467742
H 0.867133 0.661074 -3.394020
O 0.883499 -1.071950 -2.290962
C -3.604857 0.865045 -0.467002
O -3.821971 1.540025 -1.454864
C -4.599916 0.769396 0.730237
C -4.420439 2.008041 1.575559

C -5.363012 2.913980 1.797412
H -6.358842 2.809427 1.385550
H -5.167751 3.782484 2.413157
H -3.430346 2.128065 2.006066
C -4.143378 -0.494295 1.493181
H -4.812818 -1.319906 1.244273
H -4.147701 -0.369536 2.575293
O -5.920269 0.611071 0.241697
H -6.040971 1.249499 -0.476217
C 0.162801 -2.876285 1.453174
H 0.151018 -2.797159 2.541325
H -0.293119 -3.841336 1.213694
O 0.931409 -3.646803 -1.228090
H 0.889711 -3.434972 -2.169407
H 2.106842 -3.771031 1.154157
H 2.159173 -2.032519 1.405593

1b-c93, ΔG = 1.5857 kcal/mol, population = 0.56 %
C 0.800918 4.017013 1.419725
C 0.116355 3.902566 0.067571
H -0.804442 4.492489 0.047539
C -0.271305 2.450996 -0.175752
C 0.871750 1.479732 0.055172
C 1.956961 1.809960 0.878677
N 2.965177 0.953705 1.128294
C 2.895765 -0.239168 0.555983
C 1.855334 -0.645832 -0.287210
C 0.808586 0.233613 -0.565672
C -0.334399 -0.088968 -1.490635
H -0.760026 -1.064162 -1.247486
H 0.049066 -0.167777 -2.511422
C -1.402773 0.996830 -1.416091
C -2.105042 1.063095 -0.037211
C -3.575149 1.440630 -0.091068
C -4.481878 0.356105 -0.680646
C -4.508114 -0.953921 0.109113
C -5.531098 -1.948247 -0.441181
C -5.510119 -3.319052 0.241109
C -5.890044 -3.285776 1.721878
H -5.922030 -4.293358 2.142006
H -6.876521 -2.834767 1.861260
H -5.175378 -2.707254 2.310293
H -6.197659 -3.985076 -0.288734
H -4.512752 -3.758535 0.130442

H -6.535164 -1.516687 -0.354913
H -5.346884 -2.083901 -1.512239
H -3.519974 -1.424747 0.093115
H -4.725883 -0.731593 1.158069
H -4.191451 0.147712 -1.715618
H -5.498112 0.757699 -0.731973
H -3.666433 2.361698 -0.673504
H -3.898617 1.673989 0.927176
H -1.960618 0.129849 0.509185
O -1.375946 2.110293 0.655412
H -2.103978 0.924129 -2.242298
O -0.761482 2.286534 -1.497186
C 2.110639 -2.006892 -0.744771
O 1.556909 -2.645496 -1.618858
C 3.310188 -2.564221 0.081967
C 2.746944 -3.467560 1.153290
C 2.996136 -4.766076 1.254436
H 3.654265 -5.273585 0.560499
H 2.558403 -5.357567 2.048217
H 2.090093 -2.976517 1.865538
C 3.950767 -1.303275 0.703448
H 4.838880 -1.035045 0.127921
H 4.249026 -1.438735 1.742322
O 4.222276 -3.219448 -0.781145
H 3.703986 -3.778730 -1.377930
C 2.081098 3.181245 1.482077
H 2.427053 3.079786 2.511795
H 2.882696 3.691719 0.940326
O 1.022100 4.348177 -0.940257
H 0.609875 4.186767 -1.798561
H 1.030008 5.063840 1.621212
H 0.093503 3.686199 2.182096

1b-c83, ΔG = 1.5970 kcal/mol, population = 0.55 %
C -0.052459 -3.674642 -1.591780
C -0.470261 -3.666419 -0.130411
H -1.454305 -4.127482 -0.005914
C -0.581297 -2.227306 0.350792
C 0.641658 -1.391002 0.025334
C 1.501503 -1.733036 -1.026059
N 2.567007 -0.981765 -1.365072
C 2.775253 0.117315 -0.656485
C 1.972217 0.525809 0.414850
C 0.874247 -0.246972 0.788218

C -0.028783 0.080013 1.948007
H -0.331420 1.128157 1.914059
H 0.530937 -0.048672 2.878324
C -1.251183 -0.832318 1.944437
C -2.170686 -0.602753 0.720045
C -3.650441 -0.763861 1.012218
C -4.541586 -0.683989 -0.231905
C -4.416809 0.612562 -1.039406
C -4.753421 1.881737 -0.257307
C -4.699325 3.146738 -1.112918
C -5.033410 4.414118 -0.328504
H -4.985633 5.302062 -0.962576
H -4.334726 4.557759 0.500060
H -6.040289 4.359336 0.093861
H -3.699659 3.239613 -1.550663
H -5.392987 3.042255 -1.953892
H -5.753918 1.781734 0.179824
H -4.063940 1.997953 0.585186
H -3.405700 0.699355 -1.450528
H -5.083985 0.542943 -1.904583
H -5.581645 -0.809231 0.084423
H -4.311399 -1.531256 -0.882186
H -3.933167 0.010167 1.730879
H -3.800254 -1.726620 1.509006
H -1.967442 0.373538 0.275505
O -1.741967 -1.627123 -0.214329
H -1.790802 -0.786189 2.886513
O -0.812813 -2.193083 1.750962
C 2.499828 1.774558 0.957745
O 2.222863 2.350143 1.992965
C 3.568146 2.298328 -0.045105
C 2.903772 3.413987 -0.822280
C 2.386342 3.337197 -2.041580
H 2.409460 2.429470 -2.631478
H 1.908617 4.196127 -2.495392
H 2.831494 4.341850 -0.261584
C 3.930477 1.059102 -0.877373
H 4.845640 0.623959 -0.471347
H 4.101376 1.268583 -1.931384
O 4.706840 2.787046 0.657344
H 4.381951 3.298751 1.412751
C 1.309203 -3.007247 -1.800107
H 1.480628 -2.802327 -2.857909
H 2.106824 -3.688234 -1.489153

O 0.513881 -4.366248 0.628821
H 0.284832 -4.274517 1.562528
H -0.021669 -4.703010 -1.952911
H -0.822387 -3.152385 -2.162370

1b-c117, ΔG = 1.6278 kcal/mol, population = 0.52 %
C 0.408613 -3.545120 1.214669
C 0.910484 -3.152702 -0.165139
H 1.988018 -3.320939 -0.248310
C 0.667904 -1.665943 -0.381830
C -0.752020 -1.239162 -0.061578
C -1.565681 -1.985499 0.801861
N -2.815599 -1.607054 1.128699
C -3.260966 -0.479589 0.593763
C -2.518387 0.320050 -0.282463
C -1.225039 -0.062800 -0.638981
C -0.360181 0.703591 -1.602952
H -0.340454 1.762898 -1.340433
H -0.805941 0.643502 -2.599372
C 1.053656 0.130118 -1.630125
C 1.823485 0.329092 -0.302559
C 3.313579 0.547002 -0.484631
C 4.082851 0.602122 0.834918
C 5.600327 0.718040 0.663402
C 6.065343 2.034373 0.037966
C 7.582834 2.137664 -0.148643
C 8.377436 2.142654 1.157989
H 8.042526 2.951160 1.813864
H 9.443323 2.286938 0.968693
H 8.265392 1.204754 1.704908
H 7.922314 1.308554 -0.779108
H 7.805689 3.053848 -0.703681
H 5.588218 2.164308 -0.937302
H 5.720797 2.867806 0.661887
H 6.066802 0.597392 1.644380
H 5.957369 -0.117532 0.049701
H 3.857513 -0.301244 1.406128
H 3.718215 1.446719 1.430828
H 3.442050 1.479360 -1.040794
H 3.707802 -0.259193 -1.112125
H 1.384998 1.142108 0.281820
O 1.588920 -0.917125 0.404785
H 1.613006 0.486312 -2.490929
O 0.970579 -1.307552 -1.721218

C -3.322383 1.467467 -0.686591
O -3.118432 2.283017 -1.565167
C -4.599342 1.486695 0.208862
C -4.391271 2.534006 1.277177
C -5.135922 3.621517 1.422691
H -5.978142 3.822826 0.772873
H -4.931938 4.336250 2.209507
H -3.555980 2.345279 1.945430
C -4.644106 0.069587 0.823354
H -5.381012 -0.528506 0.283927
H -4.913106 0.066531 1.878903
O -5.742374 1.724064 -0.593506
H -5.525197 2.448420 -1.198269
C -1.094250 -3.296922 1.366130
H -1.391559 -3.353104 2.414353
H -1.652934 -4.082098 0.848514
O 0.204135 -3.916691 -1.140772
H 0.468323 -3.596235 -2.012653
H 0.631776 -4.597081 1.394885
H 0.965549 -2.963327 1.951179

1b-c95, ΔG = 1.6284 kcal/mol, population = 0.52 %
C -0.482569 -3.993373 1.259206
C 0.244778 -3.703753 -0.042989
H 1.244669 -4.146951 -0.035465
C 0.417575 -2.199807 -0.194761
C -0.873100 -1.427039 0.006847
C -1.936800 -1.960651 0.747850
N -3.078113 -1.282501 0.970489
C -3.163811 -0.065016 0.454719
C -2.153699 0.538988 -0.302437
C -0.969532 -0.154582 -0.553337
C 0.158212 0.387474 -1.389824
H 0.412573 1.402199 -1.078262
H -0.175426 0.459385 -2.428443
C 1.376819 -0.524340 -1.293329
C 1.998943 -0.556618 0.125652
C 3.510655 -0.678637 0.155043
C 4.217704 0.625267 -0.212425
C 5.740056 0.504884 -0.146543
C 6.486937 1.771734 -0.577158
C 6.179249 3.026165 0.249721
C 6.495831 2.879887 1.737797
H 6.331217 3.819631 2.269185

H 7.538935 2.588413 1.889107
H 5.869409 2.120904 2.210963
H 6.757644 3.859147 -0.160662
H 5.127824 3.301856 0.126814
H 7.563361 1.575222 -0.530266
H 6.257600 1.977025 -1.628225
H 6.032741 0.226814 0.870519
H 6.057695 -0.322950 -0.788444
H 3.876440 1.414424 0.463767
H 3.927555 0.939354 -1.220906
H 3.813477 -1.483966 -0.521266
H 3.804960 -0.981254 1.163759
H 1.678827 0.315375 0.702034
O 1.408801 -1.741192 0.718525
H 2.106958 -0.300920 -2.066021
O 0.950094 -1.890749 -1.473526
C -2.591248 1.866599 -0.717712
O -2.094969 2.626089 -1.527285
C -3.907908 2.190735 0.052505
C -3.553914 3.113736 1.194526
C -4.001746 4.354501 1.329951
H -4.686074 4.792789 0.614556
H -3.706455 4.964586 2.173865
H -2.874689 2.691470 1.929667
C -4.378901 0.816080 0.577902
H -5.179127 0.445175 -0.065389
H -4.754872 0.850883 1.599665
O -4.859548 2.742798 -0.839788
H -4.399010 3.399125 -1.382722
C -1.877462 -3.363384 1.286391
H -2.282525 -3.365506 2.299440
H -2.566084 -3.961346 0.682447
O -0.528767 -4.223640 -1.122794
H -0.105358 -3.950019 -1.946522
H -0.559335 -5.072112 1.398225
H 0.126521 -3.602984 2.076290

1b-c61, ΔG = 1.6290 kcal/mol, population = 0.52 %
C 0.701336 -3.913296 -1.235132
C -0.051750 -3.667543 0.061926
H -1.031465 -4.153296 0.039541
C -0.291602 -2.173761 0.225005
C 0.964722 -1.342403 0.040039
C 2.055966 -1.820393 -0.697063

N 3.165810 -1.086916 -0.909381
C 3.189350 0.129415 -0.386898
C 2.148796 0.679360 0.370585
C 0.998495 -0.069964 0.610243
C -0.158791 0.413069 1.442893
H -0.456675 1.417918 1.137979
H 0.162930 0.489997 2.484911
C -1.334022 -0.551757 1.328315
C -1.942231 -0.600101 -0.096254
C -3.446864 -0.787978 -0.136577
C -4.212405 0.482834 0.230665
C -5.727492 0.288599 0.176077
C -6.532726 1.521579 0.599892
C -6.296658 2.780527 -0.243550
C -6.615515 2.601150 -1.727413
H -7.642473 2.252572 -1.867908
H -5.952444 1.871974 -2.197407
H -6.505282 3.542639 -2.269671
H -6.914728 3.586954 0.162066
H -5.260230 3.112144 -0.131735
H -7.598310 1.270663 0.565886
H -6.304133 1.750043 1.646315
H -6.012840 -0.014004 -0.836078
H -6.000480 -0.547080 0.828313
H -3.913491 1.284266 -0.451152
H -3.931133 0.815080 1.235889
H -3.718266 -1.607607 0.535902
H -3.722236 -1.099910 -1.147705
H -1.656183 0.289333 -0.663904
O -1.295302 -1.752767 -0.692905
H -2.080629 -0.368114 2.095882
O -0.847781 -1.898994 1.501536
C 2.524241 2.025989 0.791678
O 2.001206 2.757271 1.611079
C 3.804155 2.417925 -0.001689
C 3.349167 3.356666 -1.097903
C 3.150186 3.052028 -2.373911
H 3.323031 2.059768 -2.771904
H 2.796731 3.798727 -3.073460
H 3.137349 4.360890 -0.741420
C 4.359235 1.073111 -0.494183
H 5.153429 0.755203 0.183947
H 4.775146 1.108497 -1.499002
O 4.737865 3.066154 0.856940

H 4.239764 3.684620 1.411384
C 2.066573 -3.221057 -1.243396
H 2.481768 -3.199366 -2.252109
H 2.774825 -3.791266 -0.635254
O 0.731937 -4.163957 1.145420
H 0.286588 -3.919381 1.966705
H 0.827692 -4.986397 -1.381645
H 0.083443 -3.543921 -2.055395

1b-c121, ΔG = 1.6635 kcal/mol, population = 0.49 %
C -0.676309 -3.361473 -1.542231
C -1.003166 -3.243879 -0.063054
H -2.061560 -3.452016 0.118019
C -0.739031 -1.817202 0.395680
C 0.630342 -1.302220 -0.007911
C 1.329131 -1.853112 -1.091086
N 2.525011 -1.386031 -1.495921
C 3.030161 -0.363971 -0.820937
C 2.404715 0.237138 0.277231
C 1.171131 -0.243040 0.718235
C 0.436161 0.303796 1.912560
H 0.389905 1.393323 1.868089
H 0.998975 0.057480 2.817006
C -0.966677 -0.290476 1.992490
C -1.874978 0.142813 0.815587
C -3.331782 0.338285 1.187844
C -4.216839 0.693049 -0.004929
C -5.680211 0.892953 0.387164
C -6.607524 1.203268 -0.793361
C -6.265843 2.477310 -1.575361
C -6.271268 3.747104 -0.724459
H -5.492927 3.722615 0.040994
H -7.231108 3.873584 -0.215941
H -6.099776 4.633032 -1.339525
H -5.289576 2.366430 -2.056010
H -6.989681 2.586594 -2.388422
H -6.598831 0.352507 -1.483028
H -7.633736 1.285272 -0.419416
H -6.040069 -0.013545 0.884566
H -5.748075 1.692000 1.132057
H -4.146475 -0.097635 -0.757214
H -3.826666 1.602172 -0.472468
H -3.381821 1.140600 1.931498
H -3.694036 -0.573403 1.673127

H -1.481060 1.044555 0.339008
O -1.750618 -0.959272 -0.121481
H -1.425428 -0.110093 2.960785
O -0.877479 -1.718111 1.804455
C 3.245602 1.318634 0.777572
O 3.156110 1.952994 1.810999
C 4.386600 1.545857 -0.261243
C 4.016025 2.755531 -1.086608
C 4.708975 3.885557 -1.124178
H 5.624720 4.009519 -0.560043
H 4.385562 4.717459 -1.736491
H 3.104254 2.650998 -1.667693
C 4.366353 0.262154 -1.120899
H 5.173019 -0.397019 -0.794356
H 4.495955 0.453968 -2.185328
O 5.625628 1.684396 0.410430
H 5.482387 2.279893 1.160389
C 0.794956 -3.050429 -1.827154
H 0.959800 -2.905503 -2.895780
H 1.416075 -3.902374 -1.535535
O -0.179149 -4.156645 0.659543
H -0.336491 -4.012654 1.601439
H -0.916609 -4.367768 -1.886487
H -1.322913 -2.669245 -2.084238

1b-c97, ΔG = 1.6893 kcal/mol, population = 0.47 %
C 0.842737 4.052694 1.394294
C 0.090953 3.934258 0.078545
H -0.826782 4.528833 0.101105
C -0.315455 2.483483 -0.136994
C 0.833130 1.507289 0.043235
C 1.959665 1.836633 0.809859
N 2.975409 0.977076 1.013756
C 2.871899 -0.218998 0.453472
C 1.788686 -0.625440 -0.334067
C 0.734352 0.258025 -0.566994
C -0.452522 -0.064110 -1.434825
H -0.869803 -1.036472 -1.167110
H -0.119086 -0.148201 -2.472612
C -1.511082 1.026176 -1.311531
C -2.139905 1.099510 0.102445
C -3.613791 1.465207 0.122224
C -4.537815 0.363545 -0.406623
C -4.532044 -0.924537 0.418664

C -5.535167 -1.956577 -0.096778
C -5.598473 -3.243473 0.731791
C -4.307238 -4.062617 0.710193
H -4.020847 -4.310286 -0.315755
H -4.426673 -4.999502 1.258464
H -3.475477 -3.520595 1.164099
H -5.854647 -2.991864 1.766674
H -6.418792 -3.862258 0.356017
H -6.529942 -1.499472 -0.120771
H -5.288379 -2.209700 -1.134557
H -3.530714 -1.361360 0.417691
H -4.762758 -0.685685 1.463329
H -4.280125 0.127341 -1.444520
H -5.556415 0.760977 -0.436107
H -3.744034 2.377562 -0.466433
H -3.886549 1.708553 1.152867
H -1.960940 0.170941 0.646625
O -1.381049 2.154573 0.748261
H -2.254913 0.952563 -2.099452
O -0.870399 2.313131 -1.431892
C 2.012265 -1.992113 -0.791656
O 1.412436 -2.634538 -1.631857
C 3.246590 -2.550640 -0.018909
C 2.729479 -3.435403 1.090702
C 2.972640 -4.734720 1.196256
H 3.591137 -5.256735 0.477066
H 2.570264 -5.312162 2.018582
H 2.113534 -2.929305 1.828482
C 3.927554 -1.287983 0.554454
H 4.784314 -1.030916 -0.071515
H 4.281260 -1.415509 1.576763
O 4.108345 -3.225265 -0.917822
H 3.554930 -3.781406 -1.485351
C 2.120359 3.210835 1.397711
H 2.517093 3.113976 2.409413
H 2.896126 3.713771 0.813010
O 0.947573 4.369661 -0.975549
H 0.493023 4.204398 -1.811437
H 1.086684 5.099441 1.577905
H 0.172690 3.729930 2.193025

1b-c102, ΔG = 1.7162 kcal/mol, population = 0.45 %
C -1.200353 3.036161 1.209400
C -1.464234 2.730497 -0.255705

H -2.537989 2.723809 -0.463815
C -0.932721 1.341422 -0.579915
C 0.496242 1.124148 -0.117615
C 1.056638 1.889193 0.914196
N 2.307416 1.687956 1.369304
C 3.005627 0.718707 0.796587
C 2.525982 -0.085481 -0.243926
C 1.236503 0.118767 -0.736313
C 0.640413 -0.660677 -1.877851
H 0.784899 -1.731968 -1.726695
H 1.175981 -0.405727 -2.796269
C -0.842437 -0.335852 -2.033072
C -1.701628 -0.817421 -0.839670
C -3.097282 -1.271524 -1.221965
C -3.959873 -1.650004 -0.020305
C -5.371130 -2.077310 -0.421541
C -6.236363 -2.554033 0.750312
C -6.452528 -1.524469 1.865705
C -7.115843 -0.230261 1.395400
H -7.317454 0.436399 2.236539
H -8.067068 -0.436648 0.896815
H -6.484288 0.313209 0.689684
H -7.071813 -1.983456 2.642120
H -5.497145 -1.290111 2.343878
H -7.213038 -2.861714 0.361578
H -5.781353 -3.451430 1.183039
H -5.864821 -1.245302 -0.933372
H -5.303516 -2.885806 -1.156847
H -4.006248 -0.799053 0.662146
H -3.475049 -2.464567 0.530124
H -2.998966 -2.132213 -1.891490
H -3.578164 -0.471640 -1.794029
H -1.185115 -1.603831 -0.282975
O -1.783870 0.361217 0.004916
H -1.230109 -0.678976 -2.988439
O -1.013485 1.095720 -1.975020
C 3.561021 -1.037554 -0.631573
O 3.614434 -1.770048 -1.600586
C 4.695006 -0.958212 0.436022
C 4.516396 -2.126974 1.376175
C 5.399827 -3.100864 1.547967
H 6.344376 -3.111179 1.018886
H 5.208053 -3.911658 2.238928
H 3.578728 -2.132597 1.924516

C 4.426047 0.378611 1.162333
H 5.107058 1.137636 0.772428
H 4.566162 0.319409 2.240895
O 5.958056 -0.940999 -0.203592
H 5.942534 -1.622039 -0.891713
C 0.295511 3.028493 1.532684
H 0.457489 3.011587 2.611433
H 0.757751 3.951066 1.169660
O -0.802639 3.711610 -1.052050
H -0.900507 3.453979 -1.977644
H -1.629853 4.006704 1.459219
H -1.721719 2.286181 1.806787

1b-c54, ΔG = 1.7376 kcal/mol, population = 0.43 %
C -0.550536 -3.423283 -1.000607
C -1.082161 -2.901113 0.323844
H -2.172026 -2.984009 0.364581
C -0.736989 -1.424506 0.454151
C 0.724174 -1.124339 0.177603
C 1.513472 -1.981696 -0.599530
N 2.798958 -1.712433 -0.899194
C 3.300358 -0.582253 -0.425524
C 2.586046 0.322862 0.368196
C 1.259436 0.053185 0.698854
C 0.415530 0.939390 1.575509
H 0.492316 1.979860 1.254992
H 0.807431 0.902042 2.595435
C -1.038842 0.478390 1.559113
C -1.712692 0.645065 0.175478
C -3.182168 1.014098 0.240346
C -3.847067 1.080216 -1.134037
C -5.305419 1.545013 -1.090731
C -6.244855 0.582736 -0.359717
C -7.726538 0.957712 -0.462546
C -8.079049 2.284185 0.211742
H -7.780948 2.276154 1.263984
H -9.153784 2.473855 0.170782
H -7.577526 3.127015 -0.267629
H -8.014697 0.996861 -1.518694
H -8.323240 0.158964 -0.011882
H -6.102559 -0.422774 -0.770632
H -5.969356 0.523400 0.698018
H -5.348687 2.535031 -0.626426
H -5.664809 1.671474 -2.117601

H -3.790621 0.096279 -1.607823
H -3.272974 1.764257 -1.767870
H -3.260805 1.990434 0.730533
H -3.689127 0.290231 0.883297
H -1.165070 1.367646 -0.435537
O -1.561494 -0.667598 -0.426141
H -1.614496 0.935089 2.359558
O -1.074576 -0.951638 1.748856
C 3.454038 1.446599 0.709742
O 3.293542 2.331412 1.528876
C 4.726563 1.332160 -0.178427
C 4.555671 2.345580 -1.289523
C 4.138909 2.105698 -2.526126
H 3.891122 1.111452 -2.876796
H 4.024421 2.912313 -3.238935
H 4.765853 3.364521 -0.976685
C 4.722861 -0.133136 -0.639445
H 5.392213 -0.702938 0.007908
H 5.053565 -0.273466 -1.666598
O 5.892203 1.619380 0.587519
H 5.690935 2.387000 1.142746
C 0.971727 -3.293276 -1.095808
H 1.308650 -3.444683 -2.122322
H 1.447949 -4.077407 -0.499989
O -0.479997 -3.644960 1.381530
H -0.755001 -3.244513 2.216202
H -0.843279 -4.466719 -1.121147
H -1.030314 -2.856321 -1.800267

1b-c11, ΔG = 1.7708 kcal/mol, population = 0.41 %
C 1.658607 3.784709 1.354272
C 0.907477 3.815911 0.033489
H 0.114417 4.568851 0.056671
C 0.243035 2.466536 -0.195889
C 1.193082 1.295316 -0.023522
C 2.349776 1.402387 0.759742
N 3.183045 0.364913 0.969595
C 2.862848 -0.786153 0.398898
C 1.736055 -0.975369 -0.409610
C 0.870741 0.090465 -0.648095
C -0.346321 0.001648 -1.529399
H -0.930751 -0.887594 -1.287071
H -0.024273 -0.114116 -2.567753
C -1.195174 1.259401 -1.382748

C -1.791339 1.419281 0.039516
C -3.185461 2.019116 0.079903
C -4.284430 1.074946 -0.418175
C -4.493128 -0.168218 0.448369
C -5.677341 -1.019717 -0.006030
C -5.889256 -2.268034 0.849376
C -7.078546 -3.111148 0.393010
H -8.007075 -2.535959 0.438855
H -7.204255 -3.997696 1.018293
H -6.947837 -3.446503 -0.639277
H -4.979014 -2.876659 0.827745
H -6.031827 -1.967759 1.892845
H -6.588327 -0.410529 0.010955
H -5.528621 -1.318987 -1.049959
H -3.591861 -0.789004 0.442574
H -4.646165 0.140715 1.488541
H -4.075257 0.767252 -1.448298
H -5.221235 1.638104 -0.459280
H -3.179279 2.935648 -0.516662
H -3.396724 2.312236 1.112051
H -1.760529 0.466929 0.571272
O -0.865124 2.328692 0.687214
H -1.946154 1.331917 -2.164191
O -0.335041 2.412832 -1.490921
C 1.704480 -2.361508 -0.868246
O 1.019068 -2.879682 -1.729226
C 2.770639 -3.142477 -0.046704
C 2.004150 -3.917425 1.002831
C 1.851600 -3.592609 2.280150
H 2.300758 -2.709589 2.717580
H 1.256139 -4.209508 2.941009
H 1.510598 -4.800542 0.606494
C 3.688741 -2.041900 0.505284
H 4.565076 -1.964104 -0.141055
H 4.038791 -2.225461 1.519051
O 3.496460 -4.028674 -0.893030
H 2.856045 -4.459598 -1.477927
C 2.756437 2.717943 1.363754
H 3.113585 2.540643 2.379264
H 3.621237 3.071358 0.794600
O 1.835885 4.094184 -1.012969
H 1.362790 4.025536 -1.852072
H 2.092906 4.766451 1.545509
H 0.933510 3.590393 2.146409

1b-c101, ΔG = 1.7771 kcal/mol, population = 0.40 %
C -0.832124 3.004826 2.050428
C -0.983019 3.320617 0.571397
H -1.992709 3.679868 0.352821
C -0.771450 2.047655 -0.235117
C 0.498155 1.306169 0.140114
C 1.095983 1.469609 1.396295
N 2.196629 0.788606 1.770094
C 2.704072 -0.060312 0.889718
C 2.177540 -0.274035 -0.389467
C 1.046603 0.430822 -0.796874
C 0.424287 0.302383 -2.161437
H 0.300449 -0.748625 -2.428477
H 1.105206 0.729836 -2.902354
C -0.915996 1.029667 -2.204148
C -1.979997 0.389432 -1.278028
C -3.393297 0.435604 -1.830136
C -4.469723 -0.004644 -0.835212
C -4.319846 -1.441980 -0.336215
C -5.458833 -1.863854 0.592377
C -5.371929 -3.313377 1.079944
C -4.155501 -3.604529 1.959743
H -3.219271 -3.477799 1.412816
H -4.179268 -4.629262 2.336740
H -4.128846 -2.932254 2.821985
H -6.281374 -3.550206 1.640260
H -5.364011 -3.983084 0.212961
H -5.480707 -1.193162 1.459319
H -6.410785 -1.720911 0.070199
H -4.280641 -2.124435 -1.193531
H -3.366795 -1.549655 0.187104
H -5.446793 0.107309 -1.315081
H -4.465876 0.676972 0.019800
H -3.423241 -0.207509 -2.715953
H -3.596957 1.456380 -2.166418
H -1.694227 -0.633747 -1.027009
O -1.898245 1.193756 -0.073171
H -1.272567 1.158850 -3.222465
O -0.748509 2.340123 -1.623771
C 2.995498 -1.264609 -1.083504
O 3.002235 -1.580659 -2.258111
C 3.950184 -1.895256 -0.028631
C 3.362917 -3.247658 0.313167

C 2.617828 -3.546086 1.369742
H 2.368560 -2.819627 2.133301
H 2.221034 -4.544238 1.504288
H 3.562991 -4.004642 -0.440017
C 3.945294 -0.881718 1.125604
H 4.830228 -0.248684 1.036843
H 3.960530 -1.338203 2.113310
O 5.259504 -2.038899 -0.570214
H 5.163100 -2.372519 -1.474325
C 0.564713 2.473521 2.381358
H 0.582416 2.028707 3.377418
H 1.279507 3.301289 2.404845
O -0.010821 4.300738 0.212639
H -0.058991 4.426458 -0.743668
H -1.036500 3.902844 2.634221
H -1.591151 2.266077 2.313339

1b-c43, ΔG = 1.7796 kcal/mol, population = 0.40 %
C 0.908688 -3.295517 0.962066
C 1.596636 -2.387440 -0.043787
H 2.663344 -2.299206 0.180610
C 0.997492 -0.991930 0.049638
C -0.518939 -0.985506 0.002726
C -1.268464 -2.116260 0.354281
N -2.614823 -2.121081 0.360785
C -3.220523 -0.994033 0.017181
C -2.548848 0.175807 -0.356887
C -1.154183 0.193544 -0.382368
C -0.338197 1.383855 -0.810760
H -0.686505 2.287951 -0.308146
H -0.492377 1.548950 -1.880438
C 1.143148 1.147618 -0.529067
C 1.462836 1.063860 0.984222
C 2.800002 1.650866 1.406444
C 4.017128 1.076712 0.684210
C 5.327006 1.698258 1.169987
C 6.582338 1.056036 0.573982
C 6.716788 1.208434 -0.942055
C 8.027780 0.635312 -1.477657
H 8.888417 1.135519 -1.025637
H 8.103770 0.753024 -2.560805
H 8.109356 -0.431266 -1.251740
H 5.878325 0.714519 -1.440596
H 6.646256 2.270403 -1.202268

H 7.465051 1.496555 1.049742
H 6.597252 -0.009926 0.830154
H 5.326030 2.770676 0.943208
H 5.372863 1.616504 2.261030
H 3.913401 1.229550 -0.392244
H 4.048768 -0.004821 0.839776
H 2.907911 1.497311 2.484432
H 2.747399 2.733960 1.253450
H 0.660741 1.528335 1.562475
O 1.446060 -0.362799 1.245711
H 1.767764 1.866088 -1.051993
O 1.496498 -0.170939 -0.994805
C -3.526373 1.210204 -0.676196
O -3.364446 2.287531 -1.216234
C -4.924241 0.689946 -0.222777
C -5.241718 1.339541 1.103860
C -6.260098 2.161339 1.318008
H -6.961747 2.413012 0.532735
H -6.430460 2.594971 2.294994
H -4.554597 1.094007 1.908622
C -4.716118 -0.831661 -0.055334
H -5.107903 -1.342604 -0.937008
H -5.213735 -1.240797 0.822992
O -5.887162 0.961749 -1.224609
H -5.743454 1.871479 -1.523557
C -0.592375 -3.416795 0.688768
H -1.104825 -3.868125 1.539638
H -0.761033 -4.087360 -0.158848
O 1.399685 -2.923217 -1.351062
H 1.758142 -2.288970 -1.985118
H 1.372515 -4.281961 0.934350
H 1.081517 -2.883005 1.957681

1b-c49, ΔG = 1.8486 kcal/mol, population = 0.36 %
C 0.110853 -3.639594 1.363232
C 0.666739 -3.365471 -0.024769
H 1.719411 -3.656149 -0.085278
C 0.592917 -1.871804 -0.307739
C -0.774346 -1.275657 -0.026016
C -1.670797 -1.887066 0.859869
N -2.873770 -1.357921 1.156005
C -3.185435 -0.212797 0.569377
C -2.355006 0.457967 -0.336126
C -1.113313 -0.082910 -0.664699

C -0.165649 0.534266 -1.658076
H -0.038249 1.599231 -1.456105
H -0.604533 0.460576 -2.656688
C 1.179530 -0.183318 -1.626679
C 1.937015 -0.000258 -0.287851
C 3.445271 0.086564 -0.425312
C 3.905625 1.445290 -0.954853
C 5.409275 1.514551 -1.243778
C 6.320198 1.208293 -0.050825
C 6.132163 2.142061 1.145109
C 7.119121 1.856957 2.275704
H 6.966357 2.531991 3.120546
H 8.151146 1.975495 1.935085
H 7.007633 0.833111 2.642659
H 6.243892 3.179517 0.811510
H 5.111648 2.053153 1.528485
H 6.172827 0.173498 0.275157
H 7.360874 1.270272 -0.385856
H 5.642924 0.816527 -2.054258
H 5.644704 2.514543 -1.622166
H 3.625025 2.221939 -0.237307
H 3.368881 1.681824 -1.878652
H 3.785573 -0.717433 -1.085902
H 3.882939 -0.101897 0.557171
H 1.554295 0.869231 0.252986
O 1.586805 -1.195362 0.455817
H 1.789123 0.070004 -2.489525
O 0.947073 -1.606802 -1.656343
C -3.022334 1.678530 -0.781308
O -2.740366 2.428908 -1.696198
C -4.257138 1.891071 0.141285
C -3.863682 2.968060 1.128971
C -3.459799 2.790019 2.380193
H -3.390722 1.811231 2.838732
H -3.174351 3.633434 2.995842
H -3.890250 3.968271 0.705610
C -4.495403 0.506643 0.761809
H -5.281049 0.002319 0.195889
H -4.804215 0.537327 1.804714
O -5.383519 2.302559 -0.626967
H -5.071978 2.958122 -1.268217
C -1.354921 -3.217172 1.484864
H -1.664805 -3.194468 2.530719
H -1.994879 -3.956024 0.993772

O -0.110569 -4.088013 -0.977867
H 0.192317 -3.832412 -1.858570
H 0.211933 -4.701559 1.589123
H 0.724205 -3.094551 2.082862

1b-c21, ΔG = 1.8681 kcal/mol, population = 0.34 %
C -0.125742 3.663962 0.867513
C -0.888737 3.003570 -0.269026
H -1.964392 3.169628 -0.161864
C -0.655491 1.500718 -0.221365
C 0.810380 1.124411 -0.117851
C 1.761339 2.016446 0.393443
N 3.060436 1.689650 0.535952
C 3.413865 0.466912 0.171065
C 2.530681 -0.481813 -0.357717
C 1.186130 -0.155387 -0.525239
C 0.164104 -1.086113 -1.121970
H 0.226695 -2.072030 -0.657856
H 0.394347 -1.229745 -2.181038
C -1.240114 -0.508151 -0.968307
C -1.696262 -0.422187 0.509778
C -3.171256 -0.693220 0.760266
C -4.133602 0.177689 -0.047660
C -5.602058 -0.014473 0.339821
C -6.164328 -1.405852 0.046226
C -7.656272 -1.525395 0.355563
C -8.217847 -2.917130 0.070446
H -8.088347 -3.183748 -0.981860
H -9.284175 -2.973827 0.299779
H -7.706879 -3.675942 0.669045
H -7.825029 -1.270458 1.407222
H -8.205104 -0.781198 -0.231208
H -5.993580 -1.648032 -1.009464
H -5.622083 -2.160095 0.625246
H -5.724658 0.206295 1.406417
H -6.203688 0.726668 -0.196504
H -4.015829 -0.027020 -1.116237
H -3.861304 1.224984 0.097565
H -3.360975 -0.549180 1.828644
H -3.344380 -1.753509 0.556806
H -1.092667 -1.092269 1.126354
O -1.375265 0.944249 0.874337
H -1.956144 -1.025544 -1.600265
O -1.214831 0.877805 -1.367184

C 3.266629 -1.708683 -0.649584
O 2.924786 -2.687104 -1.286436
C 4.672276 -1.570122 0.004011
C 4.632121 -2.402827 1.266815
C 4.456125 -1.961299 2.505557
H 4.340090 -0.910633 2.740655
H 4.415983 -2.650675 3.339228
H 4.716458 -3.469588 1.078942
C 4.824587 -0.058901 0.232797
H 5.411084 0.358893 -0.587714
H 5.324993 0.198875 1.164010
O 5.678533 -2.051549 -0.881707
H 5.350121 -2.875304 -1.271030
C 1.382110 3.425337 0.756619
H 1.885140 3.694177 1.686662
H 1.803257 4.074472 -0.016642
O -0.417762 3.538085 -1.504781
H -0.846961 3.049562 -2.218814
H -0.333915 4.734318 0.868556
H -0.506336 3.257587 1.806226

1b-c147, ΔG = 1.9208 kcal/mol, population = 0.32 %
C -0.835130 3.277945 1.039965
C -1.378233 2.606678 -0.210827
H -2.471177 2.573669 -0.188703
C -0.883779 1.168732 -0.262984
C 0.613362 1.040837 -0.052972
C 1.348137 2.031875 0.611803
N 2.669937 1.922960 0.843009
C 3.266015 0.821326 0.411145
C 2.609799 -0.211005 -0.269148
C 1.242650 -0.108134 -0.528027
C 0.453927 -1.139254 -1.289896
H 0.656497 -2.139570 -0.903174
H 0.788971 -1.139680 -2.330600
C -1.038549 -0.828144 -1.224025
C -1.620940 -0.965194 0.204573
C -3.040669 -1.501957 0.294472
C -4.074599 -0.744405 -0.538494
C -5.491543 -1.317666 -0.429235
C -6.124110 -1.172234 0.956354
C -7.552720 -1.718527 1.053430
C -8.575971 -0.954459 0.212307
H -8.584940 0.105858 0.479945

H -9.583506 -1.345918 0.368720
H -8.359181 -1.024744 -0.855200
H -7.553037 -2.773984 0.759813
H -7.866042 -1.693901 2.101425
H -5.506556 -1.688283 1.696227
H -6.123044 -0.113029 1.240357
H -6.120068 -0.814136 -1.168026
H -5.478704 -2.377744 -0.709177
H -3.773535 -0.755397 -1.589236
H -4.082944 0.305208 -0.233551
H -3.324044 -1.492740 1.349348
H -3.016339 -2.554062 -0.009433
H -0.960618 -1.581202 0.819715
O -1.576754 0.394066 0.709845
H -1.596114 -1.396861 -1.962537
O -1.229484 0.573869 -1.504488
C 3.572251 -1.253603 -0.606083
O 3.448003 -2.210415 -1.346061
C 4.900118 -0.928434 0.144095
C 4.969200 -1.824310 1.358038
C 5.901310 -2.744267 1.566319
H 6.708811 -2.904327 0.862972
H 5.891116 -3.356050 2.459174
H 4.172468 -1.675483 2.081252
C 4.740381 0.551844 0.557213
H 5.307934 1.177224 -0.134591
H 5.093769 0.757367 1.566891
O 5.998425 -1.090833 -0.735342
H 5.864817 -1.922150 -1.213521
C 0.695069 3.313018 1.050914
H 1.068571 3.573308 2.042414
H 1.051740 4.096327 0.375734
O -0.919314 3.329076 -1.352059
H -1.189681 2.837698 -2.138345
H -1.230451 4.291978 1.106376
H -1.208653 2.726458 1.904568

1b-c213, ΔG = 1.9509 kcal/mol, population = 0.30 %
C 2.084630 -2.455846 2.071607
C 2.309379 -2.745481 0.596482
H 3.377586 -2.753495 0.361469
C 1.673439 -1.638875 -0.231344
C 0.234329 -1.350270 0.156146
C -0.253079 -1.661666 1.432791

N -1.507250 -1.365994 1.822654
C -2.281936 -0.754400 0.939048
C -1.879809 -0.420144 -0.359331
C -0.588914 -0.734257 -0.785029
C -0.070367 -0.456043 -2.170902
H -0.318656 0.562178 -2.475525
H -0.573307 -1.121396 -2.877807
C 1.436712 -0.681193 -2.220747
C 2.210948 0.312519 -1.316246
C 3.526374 0.797945 -1.890563
C 4.357404 1.677947 -0.949213
C 3.650002 2.956012 -0.465418
C 2.891502 2.807793 0.856716
C 2.123890 4.066913 1.254873
C 1.370692 3.911587 2.574986
H 2.058119 3.684853 3.394222
H 0.645693 3.095238 2.516814
H 0.827644 4.822134 2.837317
H 2.819963 4.909679 1.325006
H 1.415865 4.320594 0.458511
H 2.195818 1.967108 0.806031
H 3.607106 2.554438 1.647218
H 4.391170 3.751493 -0.343337
H 2.962136 3.307552 -1.243232
H 5.270494 1.949510 -1.484491
H 4.674691 1.086545 -0.086005
H 3.295617 1.363491 -2.799344
H 4.112542 -0.074024 -2.195728
H 1.568381 1.158803 -1.065780
O 2.452772 -0.455570 -0.110831
H 1.811931 -0.701382 -3.240284
O 1.731618 -1.956478 -1.613595
C -2.983010 0.240029 -1.048620
O -3.108854 0.509816 -2.227517
C -4.079559 0.550831 0.014684
C -3.938139 2.004978 0.400890
C -4.866630 2.931249 0.205747
H -5.821929 2.691071 -0.243546
H -4.701903 3.957795 0.506808
H -2.989845 2.265447 0.862253
C -3.714005 -0.368937 1.201010
H -4.352869 -1.253894 1.174389
H -3.836615 0.109321 2.171848
O -5.357575 0.230332 -0.502710

H -5.393640 0.566080 -1.410319
C 0.596075 -2.407931 2.423636
H 0.446332 -1.969635 3.411378
H 0.195611 -3.424533 2.477959
O 1.714608 -4.003151 0.282406
H 1.790241 -4.134783 -0.671315
H 2.583914 -3.218640 2.669729
H 2.559307 -1.500203 2.300426

1b-c148, ΔG = 1.9585 kcal/mol, population = 0.30 %
C 0.452950 3.779853 -1.452759
C 0.912395 3.593636 -0.015981
H 1.953535 3.907172 0.102324
C 0.841290 2.116632 0.344658
C -0.493502 1.479575 0.002677
C -1.330759 2.020182 -0.983101
N -2.493776 1.445621 -1.342908
C -2.826927 0.324358 -0.721144
C -2.057598 -0.274807 0.283055
C -0.857664 0.316616 0.679043
C 0.020688 -0.221389 1.776635
H 0.169532 -1.295799 1.656396
H -0.489253 -0.087696 2.734550
C 1.357962 0.511334 1.790566
C 2.192591 0.260612 0.509211
C 3.693017 0.185313 0.724448
C 4.159562 -1.074017 1.463846
C 3.763259 -2.405651 0.809918
C 4.170124 -2.558015 -0.658532
C 5.675820 -2.468544 -0.909387
C 6.042060 -2.690032 -2.375749
H 5.717513 -3.676444 -2.717288
H 7.120329 -2.619594 -2.534110
H 5.561619 -1.944167 -3.014618
H 6.048247 -1.490966 -0.590503
H 6.187632 -3.209055 -0.285298
H 3.808860 -3.526333 -1.020159
H 3.661468 -1.803697 -1.268163
H 4.214265 -3.217068 1.389740
H 2.681705 -2.548338 0.891958
H 3.774772 -1.061908 2.487920
H 5.246845 -1.022275 1.557200
H 4.005210 1.074274 1.279254
H 4.172465 0.244001 -0.255477

H 1.829368 -0.628381 -0.009013
O 1.892920 1.417709 -0.313342
H 1.914819 0.313815 2.702095
O 1.108818 1.930921 1.725808
C -2.725546 -1.488731 0.740043
O -2.489351 -2.183981 1.709161
C -3.895317 -1.779668 -0.248925
C -3.426577 -2.851244 -1.205493
C -3.982704 -4.048614 -1.329172
H -4.846174 -4.336467 -0.742961
H -3.597254 -4.771833 -2.036275
H -2.566652 -2.579167 -1.810908
C -4.098751 -0.437161 -0.985664
H -4.949350 0.084909 -0.543103
H -4.289414 -0.554524 -2.051585
O -5.058374 -2.142313 0.472365
H -4.790241 -2.779359 1.150481
C -0.993685 3.322498 -1.653459
H -1.229267 3.240454 -2.715470
H -1.678501 4.072392 -1.246565
O 0.057465 4.352121 0.837458
H 0.306230 4.159388 1.750504
H 0.553331 4.829513 -1.730346
H 1.124414 3.207708 -2.095302

1b-c67, ΔG = 1.9685 kcal/mol, population = 0.29 %
C 1.765377 2.672430 -1.014375
C 1.834620 2.459410 0.488729
H 2.872939 2.369686 0.820277
C 1.134556 1.154857 0.840460
C -0.251594 1.037245 0.235877
C -0.617308 1.767796 -0.902999
N -1.828534 1.653940 -1.479458
C -2.682434 0.808770 -0.920688
C -2.401061 0.047813 0.219780
C -1.154097 0.161001 0.835217
C -0.764880 -0.581980 2.085382
H -1.008069 -1.641938 1.991572
H -1.359953 -0.203180 2.920624
C 0.721481 -0.394351 2.375451
C 1.632912 -1.047092 1.303896
C 2.901456 -1.666349 1.864996
C 3.784759 -2.386197 0.841206
C 4.532251 -1.477103 -0.135670

C 5.427321 -2.257830 -1.096965
C 6.251379 -1.376600 -2.041096
C 5.408636 -0.537038 -3.002335
H 4.745102 -1.172656 -3.595447
H 6.041395 0.022098 -3.695027
H 4.786305 0.184168 -2.469473
H 6.892353 -0.715972 -1.446991
H 6.924834 -2.014833 -2.621156
H 6.107578 -2.891748 -0.517717
H 4.807353 -2.938579 -1.692907
H 3.813547 -0.878413 -0.693535
H 5.145041 -0.767408 0.433105
H 3.167615 -3.095831 0.277639
H 4.516050 -2.990163 1.387927
H 2.586781 -2.389794 2.623477
H 3.471118 -0.888752 2.383612
H 1.074197 -1.794192 0.733216
O 1.942538 0.061959 0.421041
H 0.976172 -0.704135 3.385133
O 1.043445 1.005799 2.248360
C -3.568885 -0.754752 0.563913
O -3.795136 -1.397970 1.570723
C -4.585392 -0.637090 -0.613284
C -4.481916 -1.900693 -1.433966
C -5.466127 -2.772353 -1.607025
H -6.444516 -2.618449 -1.169662
H -5.324080 -3.661228 -2.208086
H -3.510504 -2.071032 -1.889429
C -4.087513 0.586367 -1.414439
H -4.712277 1.448260 -1.172378
H -4.120645 0.437182 -2.492979
O -5.885566 -0.406763 -0.099889
H -6.018084 -1.020654 0.637088
C 0.320200 2.775188 -1.508824
H 0.275965 2.684108 -2.595140
H -0.082640 3.763562 -1.269487
O 1.191937 3.556821 1.134545
H 1.172094 3.370353 2.081867
H 2.312812 3.578304 -1.276478
H 2.274008 1.834912 -1.494996

1b-c186, ΔG = 2.0112 kcal/mol, population = 0.27 %

C -0.737956 3.371400 1.580484
C -1.015727 3.330457 0.087084

H -2.059461 3.582966 -0.120746
C -0.782655 1.917007 -0.425079
C 0.556593 1.340934 -0.002285
C 1.234364 1.819671 1.127706
N 2.401533 1.298150 1.549194
C 2.898696 0.292315 0.844659
C 2.293060 -0.239207 -0.299493
C 1.090195 0.299032 -0.758419
C 0.379926 -0.170988 -1.999267
H 0.302564 -1.259672 -2.006889
H 0.977458 0.100641 -2.873673
C -1.003036 0.466066 -2.092221
C -1.953396 0.008334 -0.957857
C -3.403093 -0.138596 -1.383050
C -4.369826 -0.436319 -0.233449
C -4.122635 -1.778990 0.455105
C -5.079762 -2.067522 1.617814
C -6.567425 -2.122819 1.250558
C -6.908165 -3.179825 0.200437
H -6.566184 -4.169428 0.516098
H -6.439431 -2.959762 -0.760813
H -7.986120 -3.236012 0.034670
H -7.138226 -2.325181 2.161764
H -6.900786 -1.141618 0.900843
H -4.795933 -3.022146 2.073511
H -4.935188 -1.303945 2.389555
H -4.184313 -2.581122 -0.287365
H -3.101773 -1.808820 0.845640
H -5.385923 -0.412059 -0.634295
H -4.310146 0.368830 0.504483
H -3.452399 -0.945037 -2.122221
H -3.703784 0.781893 -1.891570
H -1.583249 -0.914640 -0.507995
O -1.836647 1.071998 0.022646
H -1.439758 0.339761 -3.079151
O -0.879582 1.881515 -1.840444
C 3.115934 -1.325648 -0.819008
O 3.040690 -1.912832 -1.881074
C 4.214183 -1.633846 0.244557
C 3.781706 -2.865606 1.004569
C 4.445283 -4.013587 1.023414
H 5.379160 -4.138676 0.490206
H 4.078009 -4.860283 1.588932
H 2.850868 -2.760451 1.554475

C 4.204301 -0.388603 1.159382
H 5.041279 0.257854 0.888508
H 4.291953 -0.630449 2.217848
O 5.470081 -1.781357 -0.393260
H 5.334906 -2.343436 -1.169999
C 0.711398 2.998378 1.900977
H 0.832518 2.798324 2.966591
H 1.369464 3.841759 1.672457
O -0.136397 4.244669 -0.565189
H -0.263213 4.146626 -1.517539
H -0.957829 4.368931 1.961875
H -1.425420 2.677944 2.067918

1b-c133, ΔG = 2.0168 kcal/mol, population = 0.27 %
C -1.358943 -2.996434 -0.905274
C -1.712165 -2.374557 0.435696
H -2.793901 -2.241622 0.528405
C -1.077087 -0.994732 0.530430
C 0.398150 -0.989084 0.178564
C 0.966685 -1.991842 -0.618494
N 2.265078 -1.986392 -0.974622
C 3.003303 -0.977148 -0.536693
C 2.516989 0.060080 0.267786
C 1.176760 0.063301 0.655034
C 0.565368 1.106658 1.551021
H 0.817241 2.108668 1.198912
H 1.003606 1.013787 2.548265
C -0.948836 0.930905 1.630376
C -1.673704 1.223545 0.296433
C -3.060859 1.813612 0.464289
C -3.798194 1.988126 -0.862161
C -5.214476 2.551927 -0.714952
C -6.171080 1.689201 0.119996
C -6.303593 0.232816 -0.339524
C -6.809363 0.076753 -1.773423
H -6.110066 0.501110 -2.496629
H -7.770109 0.581632 -1.907144
H -6.947298 -0.976074 -2.028803
H -5.340551 -0.276307 -0.236954
H -6.988499 -0.282092 0.340704
H -5.853371 1.699741 1.166533
H -7.161142 2.157149 0.102170
H -5.159772 3.547907 -0.263070
H -5.635159 2.696961 -1.713767

H -3.834440 1.026060 -1.377442
H -3.215879 2.656935 -1.504352
H -2.960984 2.784063 0.961724
H -3.624393 1.163425 1.138223
H -1.060623 1.856273 -0.350371
O -1.777480 -0.089689 -0.316995
H -1.372119 1.487483 2.462094
O -1.246110 -0.465705 1.836336
C 3.602627 0.986274 0.567945
O 3.641186 1.897658 1.371998
C 4.815981 0.595681 -0.330159
C 4.855069 1.563070 -1.489252
C 5.848473 2.405987 -1.736124
H 6.730999 2.442492 -1.109876
H 5.813237 3.075767 -2.585651
H 3.982697 1.536828 -2.135961
C 4.476751 -0.832241 -0.812519
H 5.043257 -1.551823 -0.218310
H 4.713082 -0.999591 -1.862574
O 6.003633 0.598164 0.441712
H 5.998088 1.404505 0.977825
C 0.152572 -3.171538 -1.071981
H 0.403937 -3.390279 -2.110857
H 0.491880 -4.032566 -0.488842
O -1.217185 -3.218485 1.473643
H -1.362359 -2.765798 2.314304
H -1.857581 -3.961629 -0.998488
H -1.755377 -2.348648 -1.689138

1b-c149, ΔG = 2.0369 kcal/mol, population = 0.26 %
C -0.044286 -3.721113 -1.363523
C -0.562015 -3.556572 0.056170
H -1.577398 -3.952888 0.147002
C -0.623261 -2.074587 0.398002
C 0.665888 -1.336125 0.088994
C 1.578165 -1.820710 -0.857014
N 2.710372 -1.164211 -1.176079
C 2.935421 -0.018808 -0.551295
C 2.082864 0.531154 0.413122
C 0.913117 -0.140762 0.763304
C -0.049779 0.343221 1.814018
H -0.289951 1.396467 1.657230
H 0.434517 0.280486 2.792128
C -1.318313 -0.503683 1.806002

C -2.144624 -0.349063 0.506049
C -3.645009 -0.417926 0.720138
C -4.463453 -0.459925 -0.573172
C -4.229836 0.718482 -1.528455
C -4.443346 2.108654 -0.920455
C -5.853616 2.354554 -0.383815
C -6.042244 3.775141 0.145430
H -7.055144 3.931540 0.522664
H -5.859775 4.513641 -0.639729
H -5.346221 3.982816 0.962610
H -6.579219 2.158440 -1.180559
H -6.076198 1.640795 0.414289
H -3.720796 2.281125 -0.115618
H -4.220956 2.860108 -1.685247
H -3.214349 0.661252 -1.931594
H -4.899012 0.600768 -2.386761
H -5.521418 -0.512400 -0.304717
H -4.237438 -1.388358 -1.102654
H -3.931525 0.449744 1.321496
H -3.866388 -1.304989 1.320398
H -1.867812 0.571208 -0.011525
O -1.707848 -1.473811 -0.300829
H -1.914904 -0.341820 2.699662
O -0.945433 -1.897217 1.769071
C 2.650145 1.789882 0.889474
O 2.335603 2.470930 1.847054
C 3.822657 2.157279 -0.065457
C 3.291293 3.233863 -0.986779
C 2.853719 3.072209 -2.228854
H 2.855863 2.111680 -2.729377
H 2.466748 3.910719 -2.793634
H 3.243887 4.213109 -0.518575
C 4.163345 0.829455 -0.757910
H 5.014510 0.378135 -0.244383
H 4.425831 0.934205 -1.808643
O 4.935906 2.639691 0.679975
H 4.591448 3.237721 1.359420
C 1.365971 -3.149316 -1.526935
H 1.623909 -3.053666 -2.582703
H 2.097377 -3.839490 -1.096358
O 0.324480 -4.232925 0.945680
H 0.038135 -4.041802 1.848000
H -0.050118 -4.778541 -1.629546
H -0.739816 -3.213473 -2.034086

1b-c92, ∆G = 2.0808 kcal/mol, population = 0.24 %

C 0.834538 4.281146 0.741094
C -0.167381 3.884109 -0.330580
H -1.102529 4.438577 -0.211284
C -0.496367 2.405599 -0.183353
C 0.737562 1.525305 -0.097063
C 1.972386 2.036269 0.326583
N 3.073334 1.270520 0.443517
C 2.947467 -0.012920 0.139771
C 1.756259 -0.602143 -0.298599
C 0.610043 0.180391 -0.439834
C -0.705267 -0.345967 -0.946924
H -0.969320 -1.271228 -0.431794
H -0.600241 -0.602790 -2.004320
C -1.796888 0.703990 -0.770949
C -2.094885 1.016397 0.721721
C -3.551817 1.289030 1.064129
C -4.356542 0.028298 1.393783
C -4.452004 -0.998565 0.265562
C -5.310240 -2.208097 0.634980
C -5.459721 -3.235825 -0.490876
C -4.145866 -3.898318 -0.908326
H -3.447720 -3.179236 -1.341032
H -4.318312 -4.676819 -1.654478
H -3.653422 -4.362502 -0.049245
H -6.162783 -4.009354 -0.168236
H -5.916078 -2.750923 -1.360593
H -4.878204 -2.697786 1.515357
H -6.303680 -1.858134 0.934071
H -4.866411 -0.522760 -0.630722
H -3.449978 -1.342313 -0.001430
H -5.366102 0.332049 1.685472
H -3.914852 -0.450942 2.274416
H -4.016397 1.841619 0.241623
H -3.560996 1.950061 1.933687
H -1.702843 0.219922 1.360563
O -1.317604 2.217273 0.962982
H -2.689214 0.457882 -1.336495
O -1.305697 1.967471 -1.263229
C 1.983359 -2.021116 -0.547321
O 1.267851 -2.833072 -1.101604
C 3.396282 -2.375694 0.009642
C 3.203415 -3.057763 1.343532

C 3.568072 -4.303624 1.614159
H 4.058701 -4.924624 0.875304
H 3.399041 -4.733850 2.592869
H 2.720567 -2.449101 2.102762
C 4.081623 -1.002330 0.185289
H 4.760958 -0.834707 -0.652718
H 4.655469 -0.921901 1.107598
O 4.095643 -3.171315 -0.930455
H 3.481969 -3.849480 -1.248446
C 2.145498 3.501445 0.614990
H 2.747969 3.610646 1.518007
H 2.744921 3.913677 -0.201857
O 0.409251 4.137671 -1.610146
H -0.201134 3.803486 -2.279770
H 1.030826 5.351541 0.673385
H 0.374539 4.096008 1.713306

1b-c65, ΔG = 2.0978 kcal/mol, population = 0.23 %
C 1.204935 4.096609 1.054236
C 0.312633 3.890842 -0.158741
H -0.579983 4.519654 -0.094500
C -0.156273 2.443479 -0.195399
C 0.976185 1.443514 -0.048560
C 2.187432 1.794991 0.563350
N 3.193226 0.917636 0.738791
C 2.994859 -0.318155 0.304541
C 1.821806 -0.749810 -0.324965
C 0.774876 0.149964 -0.526629
C -0.508015 -0.201025 -1.231416
H -0.922097 -1.129673 -0.834506
H -0.291544 -0.388034 -2.286588
C -1.515171 0.935787 -1.097290
C -1.983401 1.158217 0.362865
C -3.434536 1.582972 0.503859
C -4.440657 0.467796 0.201874
C -4.389461 -0.707940 1.181648
C -5.510543 -1.730601 0.978825
C -5.444380 -2.483467 -0.351305
C -6.526789 -3.554028 -0.477916
H -6.438757 -4.296871 0.319286
H -6.460386 -4.079362 -1.433080
H -7.524306 -3.111885 -0.409140
H -5.536255 -1.778750 -1.182270
H -4.456280 -2.946480 -0.449105

H -5.475383 -2.459414 1.795314
H -6.480249 -1.225768 1.059884
H -3.427089 -1.223491 1.102579
H -4.443883 -0.314978 2.202172
H -4.294378 0.108212 -0.820296
H -5.445224 0.900682 0.225722
H -3.605143 2.432892 -0.162797
H -3.582587 1.943520 1.525612
H -1.775025 0.274270 0.967479
O -1.126741 2.234623 0.824926
H -2.342614 0.821451 -1.791552
O -0.854557 2.181418 -1.402731
C 1.955315 -2.158522 -0.678121
O 1.250252 -2.847467 -1.389977
C 3.248068 -2.695129 0.009420
C 2.826113 -3.460851 1.241410
C 3.041978 -4.754618 1.436436
H 3.566714 -5.358776 0.707192
H 2.711966 -5.244378 2.343528
H 2.305556 -2.871406 1.990836
C 4.022952 -1.414842 0.393449
H 4.816718 -1.246921 -0.337032
H 4.478085 -1.466925 1.381703
O 3.989199 -3.477618 -0.909400
H 3.362898 -4.062346 -1.360440
C 2.450047 3.208114 1.004021
H 2.947098 3.184419 1.974959
H 3.175795 3.626944 0.300930
O 1.061744 4.197815 -1.333123
H 0.514282 3.976851 -2.097438
H 1.498477 5.145086 1.113008
H 0.615076 3.873616 1.944953

1b-c112, ΔG = 2.1034 kcal/mol, population = 0.23 %
C 1.405300 -2.562787 1.949924
C 1.532740 -2.831008 0.459447
H 2.582351 -2.946326 0.174309
C 0.984984 -1.638761 -0.311811
C -0.391338 -1.203231 0.154225
C -0.859040 -1.506462 1.439934
N -2.060377 -1.097809 1.889987
C -2.801321 -0.381371 1.057426
C -2.414075 -0.042041 -0.244239
C -1.178112 -0.468616 -0.730585

C -0.685766 -0.194984 -2.126742
H -0.809652 0.860284 -2.376411
H -1.305635 -0.752615 -2.833931
C 0.774201 -0.614499 -2.270271
C 1.738977 0.242764 -1.411822
C 3.080913 0.503887 -2.073809
C 4.038981 1.402937 -1.286521
C 4.659171 0.766727 -0.039800
C 5.756453 1.619238 0.603018
C 5.270131 2.953140 1.173251
C 6.373179 3.722553 1.898159
H 6.007120 4.673109 2.292348
H 7.205986 3.939578 1.223808
H 6.769165 3.142977 2.736290
H 4.865974 3.575356 0.370021
H 4.439568 2.764412 1.862356
H 6.222139 1.044027 1.410622
H 6.547753 1.809980 -0.131830
H 3.880714 0.559006 0.696942
H 5.080346 -0.205073 -0.317849
H 3.513010 2.323962 -1.017036
H 4.849147 1.703113 -1.959615
H 2.866717 0.982475 -3.034467
H 3.553656 -0.458181 -2.295672
H 1.266137 1.189328 -1.136028
O 1.899191 -0.553245 -0.209292
H 1.076350 -0.664689 -3.312603
O 0.936241 -1.929324 -1.699912
C -3.472736 0.738776 -0.873536
O -3.608009 1.060818 -2.038239
C -4.507631 1.105431 0.233511
C -4.247589 2.535733 0.643684
C -5.111702 3.531370 0.500615
H -6.097566 3.371400 0.082492
H -4.861342 4.536416 0.814905
H -3.266483 2.716270 1.073673
C -4.178617 0.131095 1.386646
H -4.898828 -0.689299 1.374536
H -4.214825 0.599093 2.369605
O -5.821915 0.891003 -0.248799
H -5.865450 1.261995 -1.142124
C -0.053300 -2.368323 2.371706
H -0.111864 -1.946465 3.376127
H -0.557189 -3.338077 2.418722

O 0.791813 -4.008182 0.143313
H 0.804814 -4.114630 -0.816477
H 1.845288 -3.391720 2.505120
H 1.989837 -1.670442 2.180639

1b-c45, ΔG = 2.1116 kcal/mol, population = 0.23 %
C -1.941282 -2.589329 -0.433565
C -2.109968 -1.845946 0.881181
H -3.154218 -1.559253 1.034558
C -1.294585 -0.562496 0.836521
C 0.139132 -0.777780 0.389717
C 0.509962 -1.894747 -0.371774
N 1.767087 -2.086822 -0.813282
C 2.662643 -1.163505 -0.495429
C 2.379341 -0.025150 0.267958
C 1.083689 0.183945 0.741623
C 0.683899 1.352399 1.602098
H 1.045441 2.286980 1.169482
H 1.171621 1.256768 2.575940
C -0.831481 1.393257 1.779273
C -1.590224 1.694305 0.461522
C -2.835057 2.543166 0.651995
C -3.563789 2.935182 -0.638271
C -4.238765 1.799167 -1.416013
C -5.338050 1.079805 -0.632544
C -6.117151 0.049973 -1.456976
C -5.289473 -1.167542 -1.869150
H -5.891807 -1.881682 -2.434915
H -4.898547 -1.684853 -0.989660
H -4.438253 -0.886761 -2.492152
H -6.979932 -0.289124 -0.875574
H -6.522578 0.537279 -2.350472
H -4.902712 0.579289 0.237576
H -6.038247 1.826959 -0.242390
H -4.680058 2.226058 -2.323544
H -3.485160 1.081851 -1.741234
H -2.851248 3.443890 -1.296308
H -4.323883 3.679677 -0.380007
H -2.515862 3.461730 1.154116
H -3.506452 2.026213 1.342389
H -0.922066 2.162877 -0.266435
O -1.936582 0.370478 -0.023613
H -1.122728 2.057605 2.587993
O -1.295892 0.067804 2.107783

C 3.597746 0.758644 0.434792
O 3.815330 1.701701 1.170964
C 4.681175 0.152012 -0.508603
C 4.775418 1.039946 -1.726650
C 5.859630 1.711361 -2.090221
H 6.781872 1.648692 -1.526494
H 5.859081 2.333433 -2.976010
H 3.863296 1.110609 -2.312411
C 4.117191 -1.238049 -0.877986
H 4.624960 -1.997820 -0.280729
H 4.249019 -1.490744 -1.929358
O 5.907447 0.027254 0.189980
H 6.049994 0.854089 0.673310
C -0.482960 -2.979620 -0.683924
H -0.339991 -3.296980 -1.717920
H -0.217103 -3.838987 -0.061432
O -1.656277 -2.685414 1.941306
H -1.682902 -2.168835 2.756850
H -2.570319 -3.480029 -0.429408
H -2.302575 -1.941119 -1.232789

1b-c17, ΔG = 2.1204 kcal/mol, population = 0.23 %
C 1.736210 -3.967890 -0.712261
C 0.668430 -3.768177 0.350498
H -0.150116 -4.481091 0.216201
C 0.079452 -2.372128 0.211907
C 1.133382 -1.282211 0.141746
C 2.441850 -1.555938 -0.277372
N 3.383387 -0.599134 -0.389894
C 3.020244 0.637624 -0.086882
C 1.739879 0.995432 0.350754
C 0.758072 0.015928 0.487275
C -0.633737 0.288521 0.990828
H -1.061748 1.153104 0.480460
H -0.580877 0.551913 2.050524
C -1.514540 -0.942061 0.802281
C -1.744212 -1.291674 -0.693258
C -3.120375 -1.835304 -1.044400
C -4.170097 -0.755144 -1.323471
C -4.511631 0.155470 -0.144206
C -5.648174 1.128589 -0.452865
C -5.993395 2.046424 0.718843
C -7.125929 3.021284 0.401726
H -6.862877 3.665820 -0.441176

H -7.351774 3.663968 1.255333
H -8.041102 2.485702 0.135720
H -6.267999 1.434767 1.584711
H -5.098981 2.606714 1.011192
H -5.375001 1.738657 -1.321488
H -6.540154 0.562371 -0.744110
H -4.786516 -0.456773 0.722162
H -3.631663 0.734767 0.152486
H -5.085327 -1.251718 -1.658831
H -3.831160 -0.140510 -2.164649
H -3.455980 -2.502227 -0.243956
H -3.003548 -2.450130 -1.939707
H -1.508830 -0.429765 -1.324002
O -0.753521 -2.322407 -0.940901
H -2.439227 -0.868130 1.363909
O -0.803747 -2.098713 1.288426
C 1.702099 2.435434 0.591058
O 0.857464 3.107406 1.152043
C 3.008538 3.036119 -0.003511
C 2.615620 3.676843 -1.316911
C 2.789394 3.173847 -2.532265
H 3.270882 2.219887 -2.707756
H 2.444068 3.708960 -3.407652
H 2.102842 4.626704 -1.192806
C 3.945856 1.826051 -0.129858
H 4.609470 1.809324 0.736825
H 4.566926 1.838152 -1.023190
O 3.555123 4.007386 0.883549
H 2.821605 4.553195 1.202729
C 2.882637 -2.964100 -0.566028
H 3.507506 -2.958684 -1.460324
H 3.534664 -3.265125 0.259156
O 1.268325 -3.926197 1.634971
H 0.601803 -3.710258 2.299657
H 2.122770 -4.985487 -0.649084
H 1.260216 -3.860857 -1.688510

1b-c96, ΔG = 2.1511 kcal/mol, population = 0.21 %
C 0.472538 3.318654 -1.840607
C 0.675835 3.494378 -0.344777
H 1.667098 3.905694 -0.133730
C 0.596713 2.134685 0.333888
C -0.633814 1.339965 -0.060796
C -1.304360 1.582284 -1.266341

N -2.370304 0.859561 -1.661512
C -2.768455 -0.111556 -0.854373
C -2.163686 -0.412055 0.371921
C -1.069061 0.335095 0.802913
C -0.371578 0.120396 2.119799
H -0.157244 -0.939078 2.271070
H -1.044561 0.416777 2.928846
C 0.912388 0.941481 2.180717
C 1.971406 0.478332 1.149709
C 3.403670 0.576786 1.641706
C 4.454926 0.268458 0.572922
C 4.380124 -1.150704 0.008545
C 5.520634 -1.460766 -0.961551
C 5.425911 -2.837091 -1.626872
C 5.532978 -4.010019 -0.651735
H 6.460957 -3.953859 -0.075775
H 5.527177 -4.964557 -1.182384
H 4.703498 -4.023477 0.057851
H 4.480665 -2.904712 -2.176392
H 6.220290 -2.921073 -2.374656
H 5.537974 -0.691345 -1.740807
H 6.476599 -1.381090 -0.430821
H 4.389990 -1.864983 0.837716
H 3.428369 -1.296929 -0.512668
H 5.445326 0.424907 1.011315
H 4.360592 0.990812 -0.242448
H 3.514706 -0.120501 2.478686
H 3.560987 1.583895 2.038480
H 1.746684 -0.534113 0.809004
O 1.772133 1.388370 0.037096
H 1.308957 0.996032 3.190951
O 0.622721 2.286027 1.744914
C -2.865725 -1.535135 0.987568
O -2.789319 -1.972770 2.119919
C -3.817338 -2.129850 -0.090418
C -3.139118 -3.381365 -0.603860
C -2.440301 -3.504701 -1.724976
H -2.301380 -2.686712 -2.420848
H -1.971212 -4.445094 -1.984793
H -3.226793 -4.225299 0.074779
C -3.954198 -1.001418 -1.123420
H -4.878702 -0.456790 -0.922377
H -3.989344 -1.347838 -2.154374
O -5.081435 -2.445102 0.485030

H -4.912420 -2.867904 1.339792
C -0.897677 2.717781 -2.163756
H -0.934399 2.375195 -3.198943
H -1.670338 3.486418 -2.069913
O -0.344630 4.356023 0.156237
H -0.261128 4.382514 1.118033
H 0.581936 4.283781 -2.336116
H 1.268046 2.670199 -2.211713

1b-c80, ΔG = 2.1536 kcal/mol, population = 0.21 %
C -1.126531 -3.083465 -0.694690
C -1.677494 -2.164561 0.383231
H -2.754833 -2.022966 0.258268
C -1.023828 -0.796416 0.257653
C 0.489089 -0.858848 0.166750
C 1.149088 -2.012563 -0.274079
N 2.488039 -2.075784 -0.409694
C 3.172510 -0.983051 -0.109060
C 2.593282 0.206616 0.348549
C 1.211215 0.284240 0.509287
C 0.492539 1.499673 1.032309
H 0.836953 2.399008 0.518686
H 0.747016 1.631704 2.087402
C -1.017179 1.336273 0.878839
C -1.471393 1.296605 -0.600654
C -2.811336 1.951452 -0.895656
C -3.982354 1.433526 -0.063314
C -5.312375 2.060212 -0.479723
C -6.508357 1.598222 0.360031
C -6.763589 0.085810 0.354984
C -6.998619 -0.499016 -1.037410
H -7.824453 0.009965 -1.542254
H -7.245334 -1.561339 -0.980994
H -6.113949 -0.399875 -1.669618
H -5.924259 -0.431745 0.828584
H -7.635519 -0.118800 0.983324
H -6.363300 1.927037 1.394637
H -7.407555 2.108754 -0.001267
H -5.234269 3.149988 -0.406779
H -5.495446 1.842705 -1.536628
H -3.809296 1.633727 0.998773
H -4.032233 0.348684 -0.167090
H -3.026942 1.803445 -1.958236
H -2.692038 3.030510 -0.752645

H -0.701326 1.734019 -1.240353
O -1.546126 -0.124049 -0.884188
H -1.559331 2.072161 1.465496
O -1.389698 0.026420 1.354317
C 3.645819 1.193493 0.574933
O 3.595307 2.266532 1.145598
C 4.951506 0.625773 -0.052962
C 5.132365 1.350160 -1.369205
C 4.831083 0.891855 -2.577310
H 4.427315 -0.099249 -2.742917
H 4.976493 1.507460 -3.455798
H 5.510138 2.362124 -1.252506
C 4.674370 -0.879373 -0.179035
H 5.120316 -1.387248 0.678281
H 5.081504 -1.329924 -1.081912
O 6.060374 0.865797 0.807762
H 5.981180 1.773981 1.134681
C 0.386690 -3.275371 -0.564305
H 0.796971 -3.732085 -1.466136
H 0.602081 -3.968883 0.253815
O -1.389818 -2.734683 1.658823
H -1.659041 -2.097257 2.332597
H -1.629881 -4.049098 -0.637348
H -1.371993 -2.646626 -1.664308

1b-c144, ΔG = 2.1850 kcal/mol, population = 0.20 %
C 0.085853 3.955548 -0.773766
C 0.954971 3.260480 0.261038
H 2.010875 3.499130 0.104637
C 0.805038 1.753741 0.109328
C -0.641596 1.298038 0.067029
C -1.673470 2.169308 -0.308834
N -2.960170 1.780657 -0.382923
C -3.224358 0.517606 -0.082339
C -2.256556 -0.415104 0.307529
C -0.920119 -0.025692 0.400858
C 0.188535 -0.937395 0.853030
H 0.150534 -1.884200 0.311409
H 0.035652 -1.181217 1.907701
C 1.545179 -0.265808 0.663973
C 1.915043 -0.040336 -0.822857
C 3.396331 -0.159945 -1.126596
C 3.887087 -1.608140 -1.111141
C 5.368060 -1.759522 -1.471661

C 6.342281 -1.020905 -0.544229
C 6.215202 -1.366876 0.943386
C 6.438751 -2.845443 1.260109
H 7.414496 -3.180358 0.897519
H 5.679466 -3.477916 0.795721
H 6.402909 -3.025974 2.336529
H 6.940849 -0.766559 1.499915
H 5.231135 -1.061552 1.311461
H 7.363147 -1.242950 -0.872485
H 6.215593 0.058598 -0.666521
H 5.614573 -2.824607 -1.484498
H 5.522047 -1.400860 -2.494460
H 3.292078 -2.191943 -1.820279
H 3.703507 -2.052037 -0.128154
H 3.944137 0.448134 -0.402434
H 3.578460 0.277641 -2.112134
H 1.336750 -0.706104 -1.468662
O 1.485382 1.324197 -1.066124
H 2.325498 -0.782581 1.215249
O 1.471999 1.083730 1.167675
C -2.900989 -1.696155 0.572802
O -2.449063 -2.693745 1.101101
C -4.376942 -1.591763 0.079701
C -4.464738 -2.294401 -1.254561
C -5.210571 -3.363672 -1.496993
H -5.837043 -3.804007 -0.731606
H -5.227214 -3.821165 -2.477797
H -3.850597 -1.863183 -2.039941
C -4.609594 -0.072445 -0.078153
H -5.171145 0.292310 0.784074
H -5.165502 0.186921 -0.978298
O -5.246916 -2.134811 1.056873
H -4.862903 -2.973524 1.351261
C -1.396137 3.619616 -0.591747
H -1.971142 3.920322 -1.468901
H -1.804229 4.188040 0.248959
O 0.532769 3.670857 1.560147
H 1.030955 3.155760 2.207561
H 0.233738 5.033606 -0.704604
H 0.428127 3.643353 -1.762005

1b-c174, ΔG = 2.2684 kcal/mol, population = 0.18 %
C -0.086829 3.913239 -1.595819
C 0.476801 3.817318 -0.187940

H 1.467163 4.278427 -0.134617
C 0.640512 2.351725 0.185857
C -0.608561 1.526961 -0.066024
C -1.575820 1.930233 -0.997398
N -2.673085 1.198717 -1.267358
C -2.811777 0.057986 -0.607705
C -1.901372 -0.412351 0.345354
C -0.764780 0.338746 0.645480
C 0.251785 -0.058210 1.681937
H 0.548617 -1.100116 1.547810
H -0.209038 0.004721 2.671232
C 1.466798 0.860834 1.610489
C 2.255638 0.715145 0.284735
C 3.759765 0.867475 0.417830
C 4.446908 -0.301606 1.134498
C 4.232371 -1.685756 0.508642
C 4.668774 -1.788285 -0.952954
C 4.476147 -3.179785 -1.565283
C 5.341559 -4.269543 -0.931749
H 5.088069 -4.431059 0.117611
H 6.400795 -4.001958 -0.979921
H 5.214620 -5.222337 -1.450172
H 3.420039 -3.460546 -1.488665
H 4.699871 -3.124737 -2.634759
H 4.107863 -1.066686 -1.553329
H 5.723302 -1.498801 -1.034255
H 4.786882 -2.411435 1.108449
H 3.179895 -1.978440 0.588418
H 4.118090 -0.333496 2.177601
H 5.519699 -0.089980 1.165464
H 3.963571 1.797358 0.955881
H 4.173166 0.990212 -0.585400
H 2.000289 -0.225504 -0.205949
O 1.741865 1.801587 -0.528653
H 2.098824 0.760381 2.488147
O 1.011548 2.228400 1.550382
C -2.375762 -1.684149 0.878183
O -1.982719 -2.313818 1.841275
C -3.574512 -2.146162 -0.005707
C -3.057270 -3.197966 -0.958384
C -3.460158 -4.461163 -0.979322
H -4.224623 -4.824474 -0.304120
H -3.045408 -5.167022 -1.687358
H -2.294822 -2.852657 -1.650778

C -3.990259 -0.866475 -0.765264
H -4.871722 -0.438163 -0.284143
H -4.229967 -1.045424 -1.812706
O -4.628458 -2.608177 0.819824
H -4.235593 -3.174851 1.499655
C -1.462743 3.251653 -1.706366
H -1.740972 3.111710 -2.752008
H -2.224707 3.906671 -1.273969
O -0.426708 4.459125 0.709807
H -0.100770 4.315810 1.607520
H -0.154199 4.961680 -1.887649
H 0.621096 3.432160 -2.272894

1b-c164, ΔG = 2.2703 kcal/mol, population = 0.17 %
C -0.783332 3.310002 -0.677250
C -1.280815 2.136220 -1.504744
H -2.373023 2.082778 -1.483582
C -0.752950 0.841534 -0.904696
C 0.741852 0.866145 -0.646587
C 1.437563 2.071064 -0.487083
N 2.757923 2.117235 -0.223919
C 3.387808 0.957754 -0.115251
C 2.770856 -0.289711 -0.267164
C 1.408389 -0.355396 -0.553841
C 0.658602 -1.641181 -0.780209
H 0.875221 -2.356309 0.015423
H 1.015121 -2.097228 -1.707656
C -0.841874 -1.377992 -0.874392
C -1.453734 -0.880910 0.458180
C -2.865005 -1.360496 0.759188
C -3.891560 -1.088923 -0.340610
C -5.305678 -1.559359 0.014560
C -5.954022 -0.794712 1.168951
C -7.400711 -1.212071 1.432073
C -8.041055 -0.448009 2.589398
H -9.073267 -0.763837 2.755637
H -8.048422 0.627268 2.392142
H -7.488606 -0.609902 3.518777
H -7.990103 -1.060302 0.521594
H -7.432051 -2.286763 1.641266
H -5.375074 -0.936369 2.086722
H -5.922142 0.279028 0.949256
H -5.939673 -1.456959 -0.872163
H -5.285405 -2.628926 0.253762

H -3.577103 -1.583906 -1.263229
H -3.909570 -0.017909 -0.558233
H -3.176393 -0.884231 1.691604
H -2.813707 -2.435300 0.963768
H -0.796844 -1.140288 1.291759
O -1.448746 0.561700 0.305468
H -1.370472 -2.232826 -1.286216
O -1.058374 -0.254916 -1.753157
C 3.769389 -1.340605 -0.091338
O 3.695110 -2.530648 -0.332224
C 5.054878 -0.667111 0.470668
C 5.087351 -0.988998 1.948832
C 4.714668 -0.194882 2.944347
H 4.354769 0.814165 2.785136
H 4.753193 -0.537754 3.970442
H 5.415995 -2.002921 2.159459
C 4.866482 0.820776 0.139327
H 5.420682 1.046275 -0.773802
H 5.219571 1.494647 0.917285
O 6.209538 -1.187954 -0.180975
H 6.092650 -2.146712 -0.252619
C 0.745090 3.393708 -0.665016
H 1.087881 4.074559 0.115650
H 1.100745 3.811966 -1.611122
O -0.810074 2.289145 -2.842562
H -1.048773 1.490484 -3.330320
H -1.203039 4.235211 -1.073246
H -1.164992 3.188792 0.338064

1b-c52, ΔG = 2.2879 kcal/mol, population = 0.17 %
C -0.289650 -3.563328 -1.072177
C -0.858718 -3.113295 0.263395
H -1.937381 -3.289778 0.304688
C -0.639618 -1.615852 0.422009
C 0.790503 -1.188344 0.149824
C 1.646821 -1.960602 -0.645491
N 2.904492 -1.578459 -0.940476
C 3.311888 -0.421387 -0.441881
C 2.526741 0.403286 0.372020
C 1.227068 0.018483 0.695446
C 0.313951 0.814502 1.589119
H 0.301947 1.863329 1.287058
H 0.709813 0.792433 2.607941
C -1.096210 0.233076 1.564916

C -1.786658 0.369333 0.186040
C -3.281610 0.612747 0.263633
C -3.959572 0.644749 -1.105571
C -5.483990 0.767440 -1.033536
C -5.979244 2.072426 -0.405989
C -7.496991 2.265171 -0.484756
C -8.295876 1.235669 0.315490
H -8.153316 0.224478 -0.070279
H -9.365481 1.453517 0.280484
H -7.989229 1.236235 1.365237
H -7.743626 3.267686 -0.122507
H -7.808312 2.232971 -1.534515
H -5.671929 2.122543 0.643675
H -5.486887 2.911176 -0.910336
H -5.891511 0.689490 -2.047226
H -5.879652 -0.088175 -0.477625
H -3.700728 -0.268728 -1.646031
H -3.552888 1.479190 -1.688274
H -3.432415 1.562538 0.783682
H -3.730079 -0.168933 0.885590
H -1.303408 1.147208 -0.411224
O -1.527352 -0.913683 -0.442004
H -1.706072 0.623565 2.374993
O -1.010988 -1.198204 1.726355
C 3.300287 1.584785 0.743003
O 3.067299 2.433350 1.582886
C 4.579559 1.595525 -0.142997
C 4.331081 2.624855 -1.224194
C 3.943491 2.390696 -2.471315
H 3.783017 1.391121 -2.855918
H 3.765896 3.207545 -3.159112
H 4.452710 3.647523 -0.878207
C 4.692238 0.147790 -0.645021
H 5.408316 -0.383616 -0.015324
H 5.029386 0.063495 -1.676176
O 5.717457 1.951199 0.636134
H 5.455059 2.683091 1.213662
C 1.215664 -3.303282 -1.167151
H 1.560901 -3.405535 -2.196999
H 1.758080 -4.056001 -0.587606
O -0.191471 -3.823616 1.304974
H -0.497977 -3.465865 2.148018
H -0.493031 -4.625416 -1.212003
H -0.818615 -3.024206 -1.859958

1b-c158, ΔG = 2.3061 kcal/mol, population = 0.16 %

C -0.098905 -3.812835 -1.356506
C -0.692604 -3.518045 0.011340
H -1.739788 -3.830645 0.055131
C -0.657784 -2.016800 0.259700
C 0.703734 -1.398580 -0.002058
C 1.633835 -2.010839 -0.853474
N 2.831953 -1.464183 -1.132867
C 3.108145 -0.300186 -0.563993
C 2.242858 0.373267 0.305930
C 1.003612 -0.186776 0.617732
C 0.021234 0.431981 1.575629
H -0.119158 1.490690 1.350699
H 0.436131 0.384120 2.585986
C -1.309740 -0.310183 1.522500
C -2.027340 -0.168835 0.156259
C -3.539517 -0.085694 0.239429
C -4.018529 1.276544 0.743127
C -5.537841 1.373768 0.912139
C -6.351230 1.140990 -0.367941
C -5.993854 2.059137 -1.542096
C -6.184053 3.546902 -1.247532
H -5.518267 3.890164 -0.453082
H -7.210107 3.754523 -0.931490
H -5.978631 4.152184 -2.132985
H -4.959823 1.879233 -1.851605
H -6.614717 1.780606 -2.398710
H -6.239689 0.100718 -0.686880
H -7.412081 1.269833 -0.128693
H -5.858223 0.646922 1.665374
H -5.778255 2.358529 1.321516
H -3.669644 2.053705 0.056475
H -3.550822 1.496034 1.707543
H -3.903045 -0.884829 0.893325
H -3.940942 -0.281270 -0.756939
H -1.632509 0.689184 -0.394311
O -1.645181 -1.380049 -0.544423
H -1.949238 -0.048790 2.360716
O -1.052560 -1.728024 1.591814
C 2.872255 1.614112 0.744576
O 2.542098 2.378198 1.630898
C 4.146493 1.827405 -0.128022
C 3.801345 2.835730 -1.199079

C 4.378180 4.022154 -1.333025
H 5.170023 4.346320 -0.669701
H 4.083000 4.699328 -2.124176
H 3.014252 2.527045 -1.881153
C 4.408898 0.436277 -0.746184
H 5.203274 -0.058231 -0.183662
H 4.710089 0.478409 -1.792130
O 5.228984 2.229294 0.691481
H 4.894622 2.902523 1.301919
C 1.359851 -3.360557 -1.455429
H 1.692861 -3.353922 -2.494321
H 2.004431 -4.073965 -0.933781
O 0.078467 -4.199684 0.999036
H -0.248252 -3.928225 1.866373
H -0.171172 -4.881965 -1.558208
H -0.707351 -3.299478 -2.103028

1b-c107, ΔG = 2.3067 kcal/mol, population = 0.16 %
C 1.171755 4.110623 1.119298
C 0.265758 3.915111 -0.084983
H -0.629750 4.537482 -0.001109
C -0.194884 2.465658 -0.135493
C 0.945620 1.471126 -0.018666
C 2.161196 1.820507 0.586044
N 3.173758 0.946566 0.737809
C 2.977947 -0.283786 0.287021
C 1.801558 -0.712429 -0.338112
C 0.747648 0.184342 -0.515802
C -0.539883 -0.162996 -1.213411
H -0.936766 -1.106634 -0.835261
H -0.335297 -0.320245 -2.275755
C -1.555882 0.960675 -1.038433
C -1.994833 1.147578 0.439797
C -3.452263 1.523091 0.659607
C -4.396582 0.319942 0.748533
C -4.508936 -0.518838 -0.526209
C -5.564605 -1.625431 -0.444712
C -5.252821 -2.717325 0.579776
C -6.277481 -3.850433 0.565322
H -6.317747 -4.330767 -0.415927
H -7.279150 -3.474772 0.790848
H -6.035758 -4.618199 1.303461
H -4.254948 -3.121208 0.376377
H -5.209530 -2.284926 1.582945

H -6.538714 -1.179141 -0.213297
H -5.666409 -2.089770 -1.431089
H -4.748686 0.141673 -1.365899
H -3.543543 -0.979300 -0.760583
H -5.393096 0.686194 1.013564
H -4.072280 -0.311819 1.580343
H -3.770613 2.209728 -0.131016
H -3.502038 2.077506 1.599525
H -1.747032 0.256207 1.023309
O -1.150842 2.237604 0.892935
H -2.395201 0.856634 -1.717281
O -0.910978 2.216203 -1.334633
C 1.938880 -2.115466 -0.712083
O 1.230474 -2.798259 -1.426588
C 3.240224 -2.654992 -0.043359
C 2.832250 -3.439012 1.181828
C 3.053687 -4.734648 1.357284
H 3.573738 -5.327367 0.615425
H 2.732971 -5.237557 2.260500
H 2.316347 -2.861687 1.943790
C 4.012412 -1.376188 0.350733
H 4.798164 -1.194221 -0.385019
H 4.477378 -1.439449 1.333743
O 3.976834 -3.421647 -0.979004
H 3.349407 -4.003666 -1.432018
C 2.420630 3.228623 1.044276
H 2.927581 3.194022 2.009741
H 3.137331 3.660671 0.339855
O 0.998083 4.242196 -1.264452
H 0.442484 4.028191 -2.024860
H 1.461327 5.159697 1.186570
H 0.593772 3.875084 2.014552

1b-c188, ΔG = 2.3362 kcal/mol, population = 0.16 %
C -0.523498 -3.586156 -1.687328
C -0.835143 -3.611501 -0.199956
H -1.842705 -3.997594 -0.021081
C -0.793161 -2.191162 0.343451
C 0.469780 -1.440700 -0.039381
C 1.223713 -1.803420 -1.164119
N 2.320694 -1.126559 -1.552187
C 2.670662 -0.080026 -0.819248
C 1.981687 0.343989 0.323005
C 0.851659 -0.355635 0.747604

C 0.065759 -0.008659 1.983246
H -0.148193 1.061173 2.013004
H 0.676707 -0.224612 2.863938
C -1.227090 -0.816182 2.032512
C -2.203235 -0.455697 0.885378
C -3.667405 -0.488527 1.279989
C -4.634570 -0.281388 0.109178
C -4.426208 1.009224 -0.692022
C -4.539048 2.290514 0.135606
C -4.332548 3.576244 -0.671714
C -5.410607 3.836520 -1.724289
H -5.419868 3.064533 -2.496022
H -5.250808 4.795655 -2.221522
H -6.403408 3.859399 -1.266277
H -4.299649 4.422912 0.020479
H -3.350376 3.539320 -1.155700
H -5.521861 2.320899 0.620930
H -3.801381 2.269451 0.942478
H -3.448261 0.987054 -1.184775
H -5.164846 1.027241 -1.497157
H -5.655153 -0.296771 0.503807
H -4.551850 -1.134570 -0.568198
H -3.821416 0.282767 2.039431
H -3.874703 -1.449469 1.759173
H -1.939097 0.514470 0.459701
O -1.937496 -1.477285 -0.110763
H -1.696334 -0.766927 3.011333
O -0.921061 -2.198870 1.756772
C 2.645968 1.518497 0.877057
O 2.477242 2.069311 1.947844
C 3.709633 1.990148 -0.160894
C 3.128173 3.166502 -0.909535
C 3.637452 4.390855 -0.905572
H 4.541508 4.627893 -0.359138
H 3.169723 5.190838 -1.464904
H 2.224973 2.949119 -1.472513
C 3.880764 0.772272 -1.095940
H 4.790464 0.236240 -0.818544
H 3.955042 1.043444 -2.148272
O 4.924381 2.292570 0.501310
H 4.703297 2.816597 1.284963
C 0.872267 -3.023894 -1.968465
H 0.988593 -2.789832 -3.027653
H 1.628362 -3.780341 -1.738648

O 0.138741 -4.421294 0.455880
H -0.018333 -4.358809 1.406718
H -0.605632 -4.595798 -2.090842
H -1.284357 -2.976783 -2.178104

1b-c145, ΔG = 2.3569 kcal/mol, population = 0.15 %
C 1.697164 -2.762139 1.536370
C 1.818697 -2.814952 0.022429
H 2.868468 -2.860674 -0.281255
C 1.232309 -1.541959 -0.569074
C -0.157425 -1.226860 -0.047227
C -0.606556 -1.724524 1.183681
N -1.821158 -1.431383 1.684707
C -2.594564 -0.637282 0.958923
C -2.226659 -0.102532 -0.281166
C -0.976108 -0.405916 -0.820174
C -0.499477 0.084088 -2.161183
H -0.666158 1.158518 -2.257084
H -1.097800 -0.390434 -2.943559
C 0.975736 -0.250506 -2.357616
C 1.905886 0.505545 -1.377349
C 3.247765 0.897534 -1.971304
C 4.257899 1.400948 -0.937746
C 3.812499 2.656413 -0.187214
C 4.846960 3.176026 0.817058
C 5.229618 2.186646 1.925595
C 4.041289 1.684259 2.745829
H 3.357246 1.088061 2.139077
H 3.472626 2.519728 3.163839
H 4.374779 1.058596 3.576501
H 5.757911 1.332092 1.493241
H 5.946271 2.676962 2.591119
H 5.753622 3.470114 0.277613
H 4.454865 4.087677 1.280513
H 3.594534 3.448519 -0.911333
H 2.873512 2.460178 0.336513
H 5.205161 1.605147 -1.446612
H 4.455270 0.594658 -0.228997
H 3.063956 1.670199 -2.725054
H 3.659196 0.028898 -2.493628
H 1.393053 1.376574 -0.965919
O 2.107213 -0.452101 -0.306212
H 1.280109 -0.133315 -3.394223
O 1.191107 -1.627997 -1.985399

C -3.319822 0.712965 -0.797675
O -3.469980 1.191966 -1.905166
C -4.371159 0.869617 0.343799
C -4.189893 2.242020 0.947937
C -5.110524 3.196415 0.942067
H -6.088017 3.038914 0.503980
H -4.916101 4.161628 1.391633
H -3.218192 2.418232 1.400433
C -3.991321 -0.236483 1.353368
H -4.673870 -1.079411 1.229169
H -4.043127 0.090823 2.391035
O -5.672423 0.652732 -0.172482
H -5.741691 1.151891 -0.999258
C 0.237316 -2.673301 1.988959
H 0.176011 -2.388098 3.040307
H -0.235095 -3.656917 1.912821
O 1.106138 -3.955352 -0.453204
H 1.111819 -3.924306 -1.418439
H 2.166701 -3.646642 1.967792
H 2.257015 -1.893173 1.886562

1b-c204, ΔG = 2.3745 kcal/mol, population = 0.15 %
C -1.726345 -2.569361 -0.419911
C -2.083808 -1.494687 0.592562
H -3.128101 -1.193944 0.484110
C -1.223678 -0.264897 0.341171
C 0.255559 -0.582903 0.236576
C 0.706053 -1.862542 -0.115648
N 2.012069 -2.159569 -0.251437
C 2.876281 -1.178676 -0.035786
C 2.511942 0.123278 0.326899
C 1.163224 0.445214 0.482303
C 0.667604 1.801527 0.907939
H 1.143376 2.585758 0.316493
H 0.966230 1.976104 1.945082
C -0.851808 1.878985 0.783810
C -1.343170 1.797432 -0.684273
C -2.545941 2.671397 -1.013420
C -3.801115 2.429954 -0.163575
C -4.695438 1.299727 -0.679297
C -5.842046 0.969332 0.274676
C -6.771424 -0.142962 -0.221314
C -6.093392 -1.508416 -0.345847
H -5.295565 -1.498654 -1.090807

H -6.809249 -2.278434 -0.641271
H -5.653049 -1.812290 0.608003
H -7.617935 -0.228055 0.466604
H -7.192638 0.145358 -1.190563
H -5.424751 0.678958 1.246430
H -6.431639 1.875033 0.453834
H -5.105600 1.584268 -1.655393
H -4.082879 0.415864 -0.850224
H -4.385646 3.353878 -0.128582
H -3.513127 2.214641 0.869192
H -2.785667 2.543414 -2.073067
H -2.209376 3.706540 -0.901922
H -0.523362 2.041810 -1.364204
O -1.661030 0.391442 -0.844354
H -1.252392 2.738788 1.313284
O -1.421493 0.691277 1.370465
C 3.714675 0.930302 0.496033
O 3.840424 2.038259 0.981152
C 4.919603 0.097456 -0.038739
C 5.260259 0.616705 -1.415755
C 6.418644 1.169509 -1.749026
H 7.225596 1.271537 -1.034410
H 6.598306 1.522666 -2.756291
H 4.467656 0.516332 -2.151767
C 4.371086 -1.344966 -0.112122
H 4.732357 -1.907299 0.751093
H 4.675913 -1.875841 -1.013087
O 5.999944 0.180485 0.873455
H 6.090677 1.110285 1.128152
C -0.261491 -2.998459 -0.302618
H 0.043167 -3.575863 -1.176792
H -0.138060 -3.662180 0.558066
O -1.843117 -2.003933 1.903360
H -1.988940 -1.283180 2.529456
H -2.379362 -3.430911 -0.277882
H -1.926765 -2.170058 -1.415565

1b-c345, ΔG = 2.3758 kcal/mol, population = 0.15 %
C -0.171246 3.680751 1.147197
C -0.750153 3.271757 -0.197250
H -1.819488 3.496980 -0.243833
C -0.596963 1.767852 -0.371165
C 0.811328 1.273731 -0.095417
C 1.696327 2.000388 0.712600

N 2.934421 1.562570 1.007641
C 3.296527 0.395232 0.494606
C 2.481472 -0.387691 -0.328361
C 1.199110 0.056008 -0.652290
C 0.256921 -0.689290 -1.559553
H 0.197812 -1.739872 -1.269724
H 0.659656 -0.674488 -2.575793
C -1.126143 -0.046413 -1.537116
C -1.831049 -0.167254 -0.164176
C -3.334353 -0.346398 -0.253863
C -4.022415 -0.361558 1.110310
C -5.549247 -0.440028 1.030870
C -6.087749 -1.729154 0.409787
C -7.613785 -1.813744 0.432530
C -8.151130 -3.101806 -0.188307
H -7.842616 -3.191331 -1.233253
H -9.242373 -3.136024 -0.158777
H -7.775090 -3.979626 0.343868
H -7.962237 -1.731657 1.467536
H -8.028350 -0.950447 -0.098917
H -5.744629 -1.819474 -0.625700
H -5.670029 -2.588784 0.947279
H -5.958331 -0.339375 2.041455
H -5.924142 0.419068 0.462610
H -3.739696 0.542382 1.654769
H -3.644204 -1.208562 1.694083
H -3.520905 -1.284582 -0.783502
H -3.744785 0.459108 -0.871699
H -1.385189 -0.971232 0.427458
O -1.521565 1.096999 0.479103
H -1.747504 -0.400918 -2.355046
O -0.977551 1.381403 -1.682340
C 3.190184 -1.608057 -0.711938
O 2.866761 -2.443511 -1.529414
C 4.491354 -1.674676 0.153028
C 4.223545 -2.640984 1.282848
C 4.715684 -3.871083 1.357914
H 5.359850 -4.276951 0.587846
H 4.488529 -4.510403 2.201165
H 3.568643 -2.264211 2.062483
C 4.649442 -0.229484 0.696577
H 5.396027 0.295748 0.097244
H 4.965368 -0.192134 1.739004
O 5.541601 -2.089371 -0.700216

H 6.324766 -2.250883 -0.158366
C 1.320597 3.354920 1.246855
H 1.664769 3.431371 2.279301
H 1.897715 4.089527 0.677766
O -0.044633 3.963159 -1.226356
H -0.359161 3.626937 -2.075305
H -0.329633 4.748811 1.299279
H -0.728000 3.155260 1.925006

1b-c130, ΔG = 2.3776 kcal/mol, population = 0.15 %
C 1.059122 -4.113645 -0.987276
C 0.113983 -3.806328 0.162717
H -0.821933 -4.361852 0.054604
C -0.234222 -2.325055 0.142113
C 0.984918 -1.425640 0.047810
C 2.197631 -1.889048 -0.481103
N 3.280272 -1.100024 -0.614172
C 3.157914 0.158984 -0.220257
C 1.989549 0.698838 0.329469
C 0.863132 -0.108778 0.487347
C -0.425132 0.362162 1.106537
H -0.731171 1.315529 0.672145
H -0.258106 0.549748 2.170525
C -1.515368 -0.689291 0.926900
C -1.906718 -0.901715 -0.560392
C -3.375665 -1.193751 -0.825455
C -4.250355 0.053846 -0.985492
C -4.335126 0.961367 0.243201
C -5.283836 2.151685 0.069113
C -6.763144 1.777508 -0.037095
C -7.673285 3.000247 -0.140998
H -8.724089 2.711738 -0.213619
H -7.428898 3.595328 -1.024965
H -7.563736 3.647073 0.733518
H -6.925534 1.135542 -0.907019
H -7.043754 1.183823 0.839637
H -5.156672 2.829769 0.919387
H -4.989756 2.720308 -0.820879
H -4.651750 0.372739 1.111997
H -3.341058 1.350595 0.479973
H -5.253302 -0.279218 -1.260611
H -3.879821 0.640558 -1.833671
H -3.761540 -1.836460 -0.027827
H -3.424520 -1.775031 -1.749083

H -1.577198 -0.052230 -1.165028
O -1.121334 -2.062249 -0.939011
H -2.371419 -0.496707 1.564229
O -0.981754 -1.977061 1.296397
C 2.214411 2.102861 0.654044
O 1.529496 2.864907 1.308727
C 3.577874 2.517300 0.021333
C 3.276449 3.261719 -1.258371
C 3.592068 4.529258 -1.486706
H 4.118730 5.124523 -0.751516
H 3.344923 5.004433 -2.427289
H 2.755332 2.680321 -2.013680
C 4.272948 1.169724 -0.275708
H 5.005005 0.966922 0.508426
H 4.790059 1.153493 -1.234238
O 4.327127 3.279863 0.949667
H 3.723615 3.924578 1.346847
C 2.368689 -3.329693 -0.874960
H 2.925874 -3.371914 -1.811982
H 3.011182 -3.788891 -0.117918
O 0.761906 -4.144027 1.387569
H 0.187201 -3.864217 2.111532
H 1.266950 -5.183816 -1.007855
H 0.545671 -3.863966 -1.917435

1b-c56, ΔG = 2.3883 kcal/mol, population = 0.14 %
C 0.154862 -3.741156 -1.753196
C -0.350070 -3.770214 -0.320182
H -1.319892 -4.272446 -0.261066
C -0.549827 -2.343199 0.168134
C 0.657363 -1.454291 -0.071751
C 1.591807 -1.746321 -1.073991
N 2.643574 -0.948159 -1.341382
C 2.764063 0.148962 -0.610379
C 1.885196 0.508483 0.418042
C 0.801362 -0.314087 0.718922
C -0.176780 -0.040039 1.829688
H -0.509673 0.998992 1.800007
H 0.330201 -0.173652 2.789067
C -1.365353 -0.989970 1.729549
C -2.200728 -0.766295 0.442614
C -3.698362 -0.947664 0.605766
C -4.373355 0.141612 1.448586
C -4.155676 1.581238 0.968486

C -4.594857 1.846317 -0.471308
C -4.438549 3.309771 -0.882265
C -4.865438 3.574388 -2.324929
H -4.739928 4.625582 -2.593232
H -5.916345 3.314419 -2.476588
H -4.272856 2.978277 -3.023969
H -5.025299 3.938487 -0.204359
H -3.393233 3.608424 -0.748980
H -4.016382 1.223544 -1.161341
H -5.641665 1.544297 -0.592354
H -4.707838 2.250134 1.636046
H -3.102376 1.859660 1.077766
H -4.034242 0.062500 2.485704
H -5.446657 -0.068159 1.467970
H -3.879095 -1.926293 1.058704
H -4.140972 -0.977500 -0.392308
H -1.972869 0.210492 0.012258
O -1.698117 -1.784337 -0.460544
H -1.968973 -0.976919 2.632431
O -0.870039 -2.333003 1.550845
C 2.325084 1.772978 1.001530
O 1.965624 2.323026 2.025190
C 3.419328 2.356810 0.062384
C 2.741967 3.441550 -0.747702
C 2.291887 3.339190 -1.991686
H 2.391778 2.432971 -2.576382
H 1.795228 4.172456 -2.472165
H 2.594660 4.363536 -0.192128
C 3.886763 1.143705 -0.755531
H 4.795395 0.745578 -0.299759
H 4.108943 1.372084 -1.795897
O 4.492245 2.897023 0.826736
H 4.102892 3.386691 1.566068
C 1.498419 -3.017245 -1.872067
H 1.724399 -2.792090 -2.915382
H 2.302934 -3.669807 -1.520484
O 0.614071 -4.439635 0.490188
H 0.321630 -4.376391 1.408427
H 0.248791 -4.762441 -2.123566
H -0.600170 -3.242229 -2.363124

1b-c115, ΔG = 2.4354 kcal/mol, population = 0.13 %
C 0.409355 3.618380 -0.999860
C 1.113554 2.947519 0.168041

H 2.198824 3.045077 0.075034
C 0.791306 1.460325 0.158829
C -0.692742 1.168336 0.037237
C -1.581033 2.101669 -0.513913
N -2.894511 1.849823 -0.668727
C -3.328120 0.661389 -0.275113
C -2.511595 -0.323689 0.292498
C -1.150978 -0.073427 0.473144
C -0.197066 -1.046043 1.113714
H -0.309770 -2.038649 0.673953
H -0.456006 -1.148076 2.171012
C 1.241496 -0.555841 0.974442
C 1.732916 -0.540444 -0.493995
C 3.194732 -0.904982 -0.696563
C 4.189273 -0.055668 0.095298
C 5.650495 -0.434728 -0.161910
C 6.028787 -1.828853 0.344596
C 7.525617 -2.141383 0.257659
C 8.067002 -2.200783 -1.171119
H 7.987191 -1.235685 -1.675154
H 7.513561 -2.932410 -1.766490
H 9.119881 -2.490844 -1.181233
H 8.082439 -1.390166 0.828353
H 7.713428 -3.100476 0.749766
H 5.707496 -1.918493 1.388155
H 5.476485 -2.592129 -0.213015
H 5.857530 -0.356191 -1.233758
H 6.295522 0.301168 0.329930
H 3.984072 -0.141337 1.166852
H 4.036004 0.993767 -0.164084
H 3.419007 -0.825038 -1.764865
H 3.298639 -1.962489 -0.438727
H 1.106478 -1.194585 -1.104878
O 1.496992 0.830081 -0.905798
H 1.912438 -1.095831 1.636388
O 1.290538 0.840211 1.333349
C -3.321283 -1.493399 0.613631
O -3.037620 -2.472244 1.276959
C -4.724923 -1.285408 -0.033176
C -4.767504 -2.101838 -1.303425
C -5.597830 -3.111986 -1.523858
H -6.332934 -3.412939 -0.788184
H -5.575523 -3.658513 -2.457876
H -4.043323 -1.810200 -2.058665

C -4.768163 0.226558 -0.347022
H -5.352723 0.732289 0.423984
H -5.214420 0.453915 -1.314289
O -5.736381 -1.638110 0.893316
H -5.473000 -2.474917 1.303247
C -1.111807 3.474529 -0.907318
H -1.584412 3.748759 -1.851550
H -1.503531 4.167796 -0.157239
O 0.656214 3.545575 1.379578
H 1.043988 3.053763 2.114737
H 0.682187 4.673731 -1.028389
H 0.778329 3.161671 -1.919866

1b-c7, ΔG = 2.4498 kcal/mol, population = 0.13 %
C 1.880397 2.480526 -0.486623
C 2.069841 1.715716 0.812933
H 3.111908 1.406775 0.932576
C 1.225820 0.450385 0.773273
C -0.213291 0.700736 0.370705
C -0.583663 1.835345 -0.361469
N -1.850619 2.057578 -0.762256
C -2.750850 1.142942 -0.435859
C -2.464515 -0.017953 0.292816
C -1.161235 -0.257805 0.724495
C -0.753200 -1.454475 1.541637
H -1.128834 -2.372971 1.086963
H -1.224759 -1.385683 2.525589
C 0.765135 -1.515422 1.698109
C 1.514406 -1.809689 0.375914
C 2.790835 -2.611105 0.568743
C 3.561594 -2.947758 -0.713198
C 4.119485 -1.763153 -1.512822
C 5.054625 -0.849300 -0.723902
C 5.553303 0.342755 -1.539599
C 6.452365 1.281705 -0.737697
H 6.789588 2.127427 -1.341042
H 7.339318 0.758362 -0.370843
H 5.922005 1.681922 0.130585
H 6.091353 -0.018471 -2.422530
H 4.688412 0.900058 -1.915166
H 4.534300 -0.471300 0.159211
H 5.912133 -1.426519 -0.357655
H 4.658650 -2.162153 -2.378861
H 3.295669 -1.165753 -1.908011

H 2.910098 -3.538544 -1.365885
H 4.390572 -3.607192 -0.435976
H 2.506105 -3.551608 1.050574
H 3.428302 -2.077991 1.278369
H 0.856267 -2.305311 -0.342623
O 1.816466 -0.480706 -0.124894
H 1.055310 -2.191028 2.497913
O 1.251661 -0.199469 2.034290
C -3.693773 -0.784451 0.473096
O -3.916913 -1.734024 1.200113
C -4.774615 -0.149789 -0.448932
C -4.902456 -1.068314 -1.644548
C -4.363663 -0.893215 -2.844314
H -3.769603 -0.023986 -3.098089
H -4.497798 -1.631441 -3.624674
H -5.468818 -1.970922 -1.432267
C -4.218157 1.246793 -0.763461
H -4.694811 1.968474 -0.097342
H -4.394636 1.572523 -1.786629
O -6.016464 -0.054087 0.243075
H -6.141709 -0.883680 0.726879
C 0.422791 2.903178 -0.690805
H 0.257150 3.234033 -1.717234
H 0.194277 3.761794 -0.052801
O 1.663348 2.549800 1.896218
H 1.706998 2.023303 2.704594
H 2.526480 3.358879 -0.487250
H 2.207783 1.835800 -1.304065

1b-c84, ΔG = 2.4661 kcal/mol, population = 0.13 %
C 0.504403 -3.366608 1.512494
C 0.833339 -3.276403 0.031575
H 1.881475 -3.531382 -0.149207
C 0.629720 -1.843933 -0.439371
C -0.716920 -1.268501 -0.040058
C -1.434264 -1.775722 1.051024
N -2.607708 -1.252208 1.455528
C -3.068118 -0.215894 0.772487
C -2.422532 0.344421 -0.336187
C -1.215004 -0.194094 -0.776932
C -0.459831 0.309063 -1.977651
H -0.371368 1.396491 -1.946078
H -1.030508 0.072681 -2.879823
C 0.918821 -0.340072 -2.047593

C 1.837785 0.068751 -0.869770
C 3.300303 0.221072 -1.238651
C 4.191173 0.559831 -0.045679
C 5.656381 0.742696 -0.439174
C 6.590931 1.028608 0.741693
C 6.263262 2.294599 1.542696
C 6.270714 3.575635 0.708776
H 6.109936 4.454304 1.337021
H 5.486101 3.566499 -0.050578
H 7.227278 3.702782 0.194289
H 5.289614 2.183623 2.028641
H 6.993404 2.388364 2.352034
H 6.577618 0.168614 1.419706
H 7.616301 1.106809 0.364638
H 6.002661 -0.163474 -0.946722
H 5.733191 1.548434 -1.175987
H 4.110742 -0.232023 0.704432
H 3.813642 1.472663 0.425013
H 3.373524 1.017591 -1.986680
H 3.639465 -0.702561 -1.717975
H 1.470284 0.984027 -0.397482
O 1.677138 -1.025659 0.070734
H 1.388174 -0.185626 -3.015349
O 0.772832 -1.761604 -1.848682
C -3.214091 1.467728 -0.831199
O -3.116201 2.088620 -1.872600
C -4.300848 1.771887 0.239568
C -3.812194 2.985822 0.999829
C -3.215031 2.995612 2.184651
H -3.031252 2.093531 2.755244
H -2.879832 3.924387 2.628398
H -3.950360 3.915891 0.455640
C -4.371069 0.480888 1.068480
H -5.204530 -0.122181 0.703033
H -4.522171 0.646860 2.133151
O -5.551023 2.042282 -0.386171
H -5.374491 2.609513 -1.151172
C -0.950776 -2.987407 1.798330
H -1.104909 -2.824176 2.865945
H -1.609947 -3.814209 1.518037
O -0.030189 -4.159741 -0.681479
H 0.131110 -4.031243 -1.624937
H 0.700563 -4.379764 1.864618
H 1.182711 -2.699505 2.047213

1b-c113, ΔG = 2.4805 kcal/mol, population = 0.12 %

C 0.882595 -4.252665 -0.683953
C -0.153992 -3.821579 0.340542
H -1.093820 -4.360764 0.191553
C -0.449509 -2.339622 0.159218
C 0.802109 -1.482730 0.106937
C 2.043072 -2.021316 -0.260393
N 3.160328 -1.275606 -0.349146
C 3.044539 0.014954 -0.072659
C 1.847703 0.631146 0.309899
C 0.683670 -0.129964 0.420007
C -0.641606 0.426321 0.865523
H -0.871590 1.344465 0.322196
H -0.571857 0.703775 1.920651
C -1.744390 -0.609093 0.671046
C -2.000716 -0.945597 -0.823156
C -3.447681 -1.225074 -1.199577
C -4.254766 0.026574 -1.556282
C -4.409323 1.052266 -0.433226
C -5.322497 2.215979 -0.821082
C -5.409797 3.322828 0.233684
C -6.022779 2.873188 1.560439
H -6.126295 3.713456 2.250207
H -5.409329 2.116850 2.053818
H -7.016592 2.444278 1.404568
H -4.407020 3.725593 0.413055
H -6.002480 4.148091 -0.171930
H -4.960692 2.647969 -1.760008
H -6.327632 1.830533 -1.027029
H -4.800847 0.553688 0.458350
H -3.430840 1.459482 -0.157806
H -5.249658 -0.292169 -1.881021
H -3.791934 0.511764 -2.422598
H -3.928781 -1.776508 -0.385727
H -3.431640 -1.890938 -2.065371
H -1.589600 -0.161886 -1.465593
O -1.221109 -2.153427 -1.021640
H -2.650112 -0.337292 1.202464
O -1.291426 -1.871636 1.201013
C 2.089763 2.049865 0.544979
O 1.367922 2.882365 1.059197
C 3.528889 2.371994 0.037594
C 3.399189 3.037213 -1.312231

C 3.793552 4.273271 -1.586163
H 4.264725 4.897903 -0.837754
H 3.669649 4.691229 -2.576872
H 2.937074 2.424614 -2.081084
C 4.196397 0.984615 -0.091471
H 4.841416 0.819203 0.773626
H 4.802293 0.879830 -0.990549
O 4.205816 3.169454 0.992644
H 3.592587 3.862768 1.276945
C 2.201435 -3.494107 -0.516541
H 2.838743 -3.629719 -1.391622
H 2.758697 -3.902567 0.331499
O 0.366101 -4.066274 1.645802
H -0.264517 -3.711352 2.285312
H 1.056598 -5.325157 -0.592523
H 0.465476 -4.074575 -1.676629

1b-c123, ΔG = 2.5088 kcal/mol, population = 0.12 %
C -3.061416 2.844465 1.308030
C -3.390635 2.121110 0.012434
H -4.395833 1.691724 0.053855
C -2.413132 0.972359 -0.178698
C -0.959450 1.396557 -0.041782
C -0.598487 2.516910 0.720010
N 0.683548 2.872058 0.927476
C 1.615579 2.109948 0.373830
C 1.342121 0.993642 -0.424139
C 0.020081 0.620444 -0.661096
C -0.378151 -0.548685 -1.521004
H 0.249901 -1.413538 -1.304737
H -0.208722 -0.296979 -2.571371
C -1.847486 -0.866199 -1.286716
C -2.125982 -1.261023 0.192662
C -3.058241 -2.442663 0.376923
C -2.349905 -3.765134 0.057939
C -1.357503 -4.205929 1.144367
C -0.287610 -5.184502 0.647123
C 0.758019 -4.567423 -0.291764
C 1.642043 -3.507480 0.367738
H 1.063189 -2.652036 0.721094
H 2.387636 -3.124332 -0.331378
H 2.171000 -3.922057 1.230026
H 1.394752 -5.369620 -0.675643
H 0.264379 -4.135921 -1.167863

H 0.231072 -5.613427 1.510744
H -0.777815 -6.020609 0.137128
H -0.864748 -3.334015 1.581384
H -1.918317 -4.665535 1.962910
H -1.835864 -3.669267 -0.902770
H -3.095570 -4.550662 -0.084310
H -3.931895 -2.304617 -0.265294
H -3.414525 -2.449565 1.410568
H -1.178743 -1.446916 0.703242
O -2.707679 -0.061714 0.753569
H -2.232439 -1.590291 -1.999210
O -2.604799 0.353071 -1.440904
C 2.597932 0.397009 -0.865577
O 2.797048 -0.449691 -1.714816
C 3.743527 1.069039 -0.050489
C 4.107953 0.125225 1.072020
C 5.292034 -0.453935 1.216383
H 6.104396 -0.264828 0.526074
H 5.487095 -1.124977 2.042938
H 3.308874 -0.073738 1.780339
C 3.095564 2.359396 0.501138
H 3.392484 3.201906 -0.126546
H 3.384262 2.582353 1.527448
O 4.831467 1.370856 -0.903935
H 4.973864 0.599601 -1.471941
C -1.645892 3.427443 1.298596
H -1.341085 3.716671 2.305291
H -1.627554 4.343414 0.700565
O -3.281668 3.045793 -1.068010
H -3.408008 2.555579 -1.890426
H -3.788941 3.640234 1.470378
H -3.172478 2.130833 2.125965

1b-c57, ΔG = 2.5533 kcal/mol, population = 0.11 %
C 0.742066 -4.019545 -0.824962
C -0.196880 -3.574369 0.283914
H -1.184131 -4.028839 0.161247
C -0.380230 -2.065674 0.207861
C 0.931641 -1.306889 0.126854
C 2.100909 -1.915596 -0.347491
N 3.267198 -1.252457 -0.467615
C 3.269442 0.024327 -0.117050
C 2.149981 0.706107 0.374243
C 0.938964 0.031674 0.517839

C -0.310903 0.659722 1.073974
H -0.503368 1.620525 0.593376
H -0.156487 0.872360 2.135153
C -1.502010 -0.276605 0.898386
C -1.882949 -0.508061 -0.585033
C -3.369520 -0.676479 -0.836057
C -4.136027 0.644005 -0.775203
C -5.637690 0.457918 -0.988999
C -6.425679 1.769894 -1.065518
C -6.332122 2.664883 0.176130
C -6.827797 1.999299 1.459622
H -6.209374 1.142813 1.736568
H -7.854405 1.641204 1.342973
H -6.811566 2.700050 2.297042
H -5.300110 3.000131 0.314748
H -6.918299 3.570052 -0.008185
H -6.080942 2.338183 -1.935962
H -7.479155 1.534180 -1.250082
H -5.796325 -0.098170 -1.918397
H -6.038200 -0.171729 -0.188658
H -3.742051 1.326450 -1.536455
H -3.960464 1.128431 0.189639
H -3.771095 -1.385365 -0.105136
H -3.497372 -1.129566 -1.822818
H -1.479652 0.290009 -1.213895
O -1.197247 -1.743331 -0.913379
H -2.348734 0.035056 1.503631
O -1.119778 -1.600426 1.326248
C 2.521133 2.088803 0.660900
O 1.911347 2.946159 1.271565
C 3.930038 2.325847 0.044259
C 3.704890 3.103692 -1.234132
C 3.702786 2.621013 -2.470023
H 3.891181 1.577726 -2.691393
H 3.503555 3.266065 -3.316254
H 3.484241 4.153662 -1.063059
C 4.488731 0.909031 -0.153572
H 5.141825 0.672291 0.688556
H 5.066567 0.784849 -1.067078
O 4.744427 3.069447 0.945924
H 4.195341 3.776854 1.314794
C 2.125706 -3.378609 -0.693300
H 2.701067 -3.511260 -1.610693
H 2.692502 -3.879667 0.096738

O 0.373466 -3.940892 1.538836
H -0.188859 -3.577676 2.234902
H 0.832104 -5.106064 -0.807622
H 0.286696 -3.746096 -1.778289

1b-c352, ΔG = 2.5690 kcal/mol, population = 0.11 %
C -0.730028 -3.276341 -1.325369
C -1.094332 -2.985538 0.121105
H -2.173188 -3.078479 0.275424
C -0.712555 -1.551243 0.456015
C 0.711323 -1.203596 0.065088
C 1.390350 -1.913642 -0.934278
N 2.636168 -1.594025 -1.331424
C 3.211177 -0.561693 -0.730905
C 2.609554 0.193880 0.279977
C 1.323260 -0.131012 0.711361
C 0.600217 0.592755 1.815645
H 0.655237 1.672237 1.664892
H 1.108171 0.390265 2.762327
C -0.852889 0.134516 1.894758
C -1.682518 0.531068 0.649470
C -3.129615 0.874861 0.945868
C -3.946423 1.177733 -0.307947
C -5.404723 1.516682 -0.003900
C -6.217009 1.827444 -1.261173
C -7.668764 2.236215 -0.992941
C -8.522138 1.132558 -0.366835
H -8.514024 0.233988 -0.990129
H -9.560537 1.452661 -0.257055
H -8.158574 0.851704 0.623429
H -7.679064 3.118656 -0.343927
H -8.125705 2.546756 -1.937357
H -5.717988 2.632446 -1.810964
H -6.205922 0.952486 -1.921737
H -5.857566 0.680056 0.536355
H -5.445537 2.378379 0.672712
H -3.906577 0.315868 -0.979694
H -3.482705 2.012665 -0.845305
H -3.138165 1.745869 1.609498
H -3.578656 0.043951 1.499068
H -1.199903 1.350984 0.110874
O -1.621126 -0.658286 -0.181015
H -1.322284 0.446171 2.823821
O -0.889618 -1.306939 1.842497

C 3.516470 1.257365 0.710797
O 3.405019 2.005956 1.658398
C 4.705244 1.285994 -0.304775
C 4.437569 2.417003 -1.270010
C 5.074974 3.580884 -1.265443
H 5.850399 3.804756 -0.543519
H 4.838844 4.347216 -1.992298
H 3.653843 2.223831 -1.995870
C 4.605405 -0.083040 -1.029263
H 5.336112 -0.768693 -0.595143
H 4.795609 -0.019907 -2.100530
O 5.894159 1.459571 0.443150
H 6.621561 1.604430 -0.175658
C 0.772194 -3.123612 -1.578277
H 0.982322 -3.095569 -2.648481
H 1.303897 -3.995664 -1.186505
O -0.384801 -3.894222 0.961077
H -0.550824 -3.638118 1.877358
H -1.049613 -4.286850 -1.581834
H -1.293086 -2.587269 -1.957273

1b-c161, ΔG = 2.5753 kcal/mol, population = 0.10 %
C 1.600303 3.112476 -1.839075
C 1.941651 3.152130 -0.358643
H 3.020813 3.256665 -0.213866
C 1.522917 1.839517 0.284150
C 0.086401 1.453812 -0.024242
C -0.555104 1.912749 -1.182425
N -1.796578 1.520943 -1.528442
C -2.403175 0.666806 -0.718523
C -1.846408 0.181196 0.470033
C -0.568677 0.585633 0.850307
C 0.114421 0.133593 2.114074
H -0.015494 -0.940872 2.254823
H -0.357245 0.619416 2.972461
C 1.592718 0.494794 2.049651
C 2.306052 -0.225416 0.873220
C 3.679005 -0.781310 1.188047
C 4.402527 -1.362927 -0.034372
C 3.562466 -2.327025 -0.879761
C 3.043120 -3.553665 -0.125868
C 2.074037 -4.413403 -0.945289
C 0.717571 -3.747301 -1.186111
H 0.229099 -3.504173 -0.238576

H 0.047705 -4.405087 -1.743525
H 0.812001 -2.819946 -1.754676
H 2.536303 -4.665688 -1.905762
H 1.914307 -5.361716 -0.423875
H 3.897523 -4.161679 0.188650
H 2.535042 -3.246747 0.793928
H 2.724991 -1.777650 -1.316652
H 4.167243 -2.667105 -1.726227
H 5.308179 -1.870375 0.311400
H 4.732495 -0.538999 -0.671449
H 3.547721 -1.552007 1.952442
H 4.288283 0.007765 1.637211
H 1.659560 -1.018182 0.492207
O 2.401207 0.804726 -0.140648
H 2.094924 0.352029 3.002596
O 1.706350 1.888350 1.690668
C -2.790500 -0.739876 1.097802
O -2.786315 -1.215172 2.217381
C -3.900337 -1.041558 0.050485
C -3.567088 -2.402383 -0.522513
C -2.939704 -2.651973 -1.664612
H -2.614447 -1.868252 -2.337666
H -2.719300 -3.668017 -1.965244
H -3.846572 -3.222940 0.132361
C -3.788962 0.121027 -0.947224
H -4.534127 0.875170 -0.686869
H -3.955373 -0.168794 -1.982865
O -5.180845 -1.056655 0.672070
H -5.088559 -1.528995 1.512541
C 0.099447 2.927214 -2.078881
H -0.094222 2.653295 -3.116944
H -0.421418 3.874886 -1.912500
O 1.244862 4.241730 0.242723
H 1.408231 4.207699 1.193903
H 1.940971 4.033857 -2.312393
H 2.159578 2.290086 -2.288077

1b-c98, ΔG = 2.6443 kcal/mol, population = 0.09 %
C -0.705595 3.429752 0.811611
C -1.462827 2.521701 -0.143130
H -2.531072 2.517865 0.091199
C -0.959491 1.094698 0.018256
C 0.552192 0.982357 -0.037391
C 1.379486 2.076703 0.243919

N 2.723929 1.993125 0.226312
C 3.246928 0.813258 -0.069331
C 2.493686 -0.328416 -0.367839
C 1.101755 -0.255927 -0.368021
C 0.200959 -1.409426 -0.721460
H 0.488715 -2.304203 -0.166442
H 0.336019 -1.648559 -1.779651
C -1.259221 -1.055584 -0.452070
C -1.573451 -0.874516 1.052958
C -2.956382 -1.329631 1.490896
C -4.116678 -0.709613 0.715167
C -5.475708 -1.216303 1.195807
C -6.667902 -0.552966 0.497249
C -6.707918 -0.718575 -1.027067
C -6.735665 -2.174227 -1.491807
H -6.835026 -2.238623 -2.577421
H -7.577204 -2.711924 -1.046236
H -5.821766 -2.702785 -1.213149
H -7.595078 -0.202861 -1.406438
H -5.850272 -0.209877 -1.476525
H -7.592670 -0.960075 0.920081
H -6.665900 0.516155 0.734969
H -5.524062 -2.301844 1.063584
H -5.561276 -1.040924 2.273212
H -4.000757 -0.923853 -0.349630
H -4.077415 0.378376 0.814258
H -3.063053 -1.104286 2.556225
H -2.987197 -2.420823 1.401898
H -0.813412 -1.373487 1.658459
O -1.441772 0.557485 1.246121
H -1.933030 -1.753902 -0.940310
O -1.520943 0.259482 -0.982760
C 3.399846 -1.436111 -0.657826
O 3.163208 -2.525778 -1.144033
C 4.831455 -0.982239 -0.249016
C 5.130995 -1.663239 1.068537
C 5.048684 -1.125781 2.278829
H 4.765573 -0.093287 2.442653
H 5.260773 -1.714713 3.162034
H 5.394263 -2.711604 0.958734
C 4.726360 0.548434 -0.174966
H 5.110775 0.968319 -1.106468
H 5.288137 0.990499 0.645440
O 5.774871 -1.375280 -1.241557

H 5.557629 -2.281989 -1.503624
C 0.797656 3.434648 0.523378
H 1.347511 3.886273 1.350542
H 1.004983 4.054357 -0.353797
O -1.242008 2.976241 -1.477113
H -1.647426 2.335897 -2.075710
H -1.101708 4.442958 0.737946
H -0.895824 3.079292 1.827579

1b-c124, ΔG = 2.6575 kcal/mol, population = 0.09 %
C 1.474028 -2.638766 1.812728
C 1.595080 -2.874145 0.316065
H 2.640907 -3.015971 0.028932
C 1.086784 -1.645350 -0.423421
C -0.280583 -1.186167 0.046836
C -0.758325 -1.505683 1.323999
N -1.949978 -1.075053 1.781639
C -2.667983 -0.319431 0.965078
C -2.269376 0.038666 -0.328164
C -1.045707 -0.408966 -0.822296
C -0.539914 -0.111977 -2.209096
H -0.641348 0.951385 -2.433470
H -1.164564 -0.640939 -2.933825
C 0.912633 -0.554588 -2.350905
C 1.881145 0.253709 -1.452403
C 3.242913 0.507592 -2.075803
C 4.282942 1.048743 -1.092167
C 3.907740 2.386807 -0.454782
C 4.973907 2.935520 0.499862
C 5.308721 2.026482 1.689449
C 4.099533 1.660095 2.550524
H 3.579454 2.557107 2.898407
H 4.403746 1.088711 3.430132
H 3.380616 1.053496 1.996696
H 5.788506 1.110829 1.332253
H 6.053828 2.533103 2.310007
H 5.892167 3.132129 -0.063799
H 4.633892 3.903810 0.882526
H 3.727686 3.123201 -1.245055
H 2.962961 2.286042 0.085238
H 5.236640 1.157144 -1.618180
H 4.442123 0.299608 -0.314662
H 3.102465 1.212853 -2.901461
H 3.602960 -0.429660 -2.510193

H 1.414575 1.190470 -1.142904
O 2.028511 -0.589093 -0.281189
H 1.227951 -0.575550 -3.390628
O 1.042498 -1.890806 -1.821212
C -3.305987 0.868746 -0.934798
O -3.443088 1.218091 -2.091888
C -4.304290 1.258054 0.193526
C -3.980237 2.688305 0.566784
C -3.274060 3.098645 1.612398
H -2.859836 2.416009 2.343922
H -3.079811 4.151840 1.770467
H -4.352231 3.412672 -0.152616
C -4.033217 0.225140 1.297598
H -4.777613 -0.569548 1.219379
H -4.085473 0.631319 2.305696
O -5.645010 1.155829 -0.276674
H -5.668073 1.536119 -1.167065
C 0.021818 -2.408424 2.238841
H -0.023113 -2.003149 3.250815
H -0.511216 -3.363110 2.269300
O 0.817540 -4.018587 -0.030498
H 0.829122 -4.101847 -0.992569
H 1.887177 -3.494274 2.347836
H 2.085706 -1.770814 2.065089

1b-c201, ΔG = 2.6613 kcal/mol, population = 0.09 %
C 2.531334 -2.558769 1.801876
C 2.865096 -2.459749 0.322223
H 3.927734 -2.243605 0.179083
C 2.081852 -1.309084 -0.288809
C 0.595847 -1.361045 0.025056
C 0.119247 -2.013951 1.170481
N -1.179181 -2.003184 1.525523
C -2.011272 -1.337387 0.738244
C -1.624182 -0.683261 -0.436831
C -0.286290 -0.699690 -0.829363
C 0.228321 -0.047174 -2.085783
H -0.211692 0.943928 -2.208529
H -0.082867 -0.636963 -2.952103
C 1.747244 0.040977 -2.019813
C 2.201520 0.907077 -0.812589
C 3.311657 1.898448 -1.084830
C 3.771116 2.659995 0.168251
C 2.632965 3.201728 1.041592

C 1.679948 4.172455 0.335831
C 0.383231 4.430024 1.113140
C -0.580180 3.240385 1.106189
H -0.861817 2.973235 0.083959
H -1.496986 3.473961 1.651472
H -0.141639 2.354319 1.569964
H 0.626637 4.700577 2.146147
H -0.125067 5.296604 0.680751
H 2.202218 5.118891 0.164754
H 1.414691 3.793097 -0.655644
H 2.069409 2.357252 1.445247
H 3.067411 3.706422 1.909729
H 4.421008 3.481915 -0.146626
H 4.387199 1.994748 0.777903
H 2.939051 2.599220 -1.836916
H 4.159814 1.375359 -1.534465
H 1.332581 1.429036 -0.409727
O 2.625862 -0.075400 0.162467
H 2.188422 0.352773 -2.962506
O 2.264750 -1.267318 -1.695708
C -2.791854 -0.053001 -1.042404
O -2.923566 0.435877 -2.147850
C -3.947380 -0.117665 0.000296
C -4.002711 1.226550 0.688979
C -5.032238 2.060302 0.636131
H -5.935310 1.813116 0.092390
H -5.005545 3.011626 1.151783
H -3.108972 1.491560 1.245788
C -3.492288 -1.218146 0.985291
H -3.994980 -2.153162 0.730218
H -3.714502 -0.985128 2.025757
O -5.159152 -0.471059 -0.639094
H -5.207754 0.035162 -1.463251
C 1.042272 -2.824434 2.037510
H 0.780118 -2.654652 3.082672
H 0.818399 -3.875873 1.834037
O 2.511251 -3.688579 -0.309496
H 2.650649 -3.582922 -1.259242
H 3.126302 -3.353221 2.253298
H 2.828059 -1.621094 2.274513

1b-c69, ΔG = 2.6795 kcal/mol, population = 0.09 %
C 1.182191 -3.064480 0.737182
C 1.594822 -2.342865 -0.535111

H 2.682208 -2.236820 -0.585037
C 1.003874 -0.940784 -0.527906
C -0.480992 -0.916380 -0.217788
C -1.101479 -1.961823 0.477654
N -2.408936 -1.942457 0.802015
C -3.100987 -0.874564 0.436240
C -2.561427 0.208709 -0.267494
C -1.214271 0.197998 -0.624278
C -0.546323 1.290383 -1.416330
H -0.788027 2.269320 -0.998529
H -0.947707 1.282724 -2.433217
C 0.964834 1.078613 -1.452863
C 1.627918 1.229473 -0.061887
C 3.014674 1.852515 -0.048460
C 4.029206 1.190597 -0.980024
C 5.435561 1.792320 -0.876852
C 6.094375 1.686942 0.503324
C 6.216244 0.259023 1.036345
C 6.972195 0.187532 2.361861
H 7.988940 0.574958 2.255638
H 7.042220 -0.838905 2.728434
H 6.470514 0.782297 3.130014
H 5.220222 -0.173055 1.166764
H 6.723031 -0.361291 0.288941
H 7.094594 2.128809 0.443662
H 5.544344 2.293655 1.229860
H 6.077469 1.293839 -1.610604
H 5.396207 2.846619 -1.170641
H 3.690466 1.280111 -2.015570
H 4.065832 0.121154 -0.766631
H 3.371522 1.816148 0.983505
H 2.909007 2.912999 -0.300660
H 0.970085 1.788711 0.607573
O 1.706301 -0.138147 0.415472
H 1.435168 1.695883 -2.212710
O 1.228400 -0.302718 -1.775474
C -3.611252 1.196193 -0.501739
O -3.613331 2.170318 -1.230369
C -4.835396 0.782889 0.365729
C -4.831900 1.704600 1.565780
C -4.390440 1.422979 2.784926
H -3.990658 0.452910 3.053152
H -4.410336 2.170531 3.567670
H -5.199297 2.701935 1.340417

C -4.575972 -0.695962 0.688199
H -5.147567 -1.309617 -0.010680
H -4.863924 -0.985547 1.696774
O -6.040801 0.934633 -0.378092
H -5.980927 1.774891 -0.855983
C -0.338216 -3.202480 0.849433
H -0.625000 -3.499814 1.859229
H -0.687700 -3.999383 0.186533
O 1.109033 -3.079635 -1.655826
H 1.299244 -2.564865 -2.450507
H 1.648091 -4.050092 0.760709
H 1.575057 -2.498869 1.583894

1b-c126, ΔG = 2.8953 kcal/mol, population = 0.06 %
C 0.126851 3.919598 0.719597
C -0.780614 3.266491 -0.309768
H -1.823009 3.555510 -0.147539
C -0.702501 1.754470 -0.157316
C 0.720720 1.229559 -0.124829
C 1.794541 2.049222 0.245657
N 3.062301 1.598347 0.312698
C 3.261854 0.324438 0.012567
C 2.248290 -0.560025 -0.374775
C 0.933305 -0.106873 -0.462435
C -0.221127 -0.963713 -0.907747
H -0.223745 -1.912673 -0.368493
H -0.089834 -1.210853 -1.964538
C -1.541518 -0.228043 -0.703914
C -1.881169 0.013862 0.788050
C -3.361523 -0.050740 1.112420
C -3.902665 -1.480698 1.107986
C -5.387834 -1.578583 1.470289
C -6.336220 -0.812497 0.538257
C -6.219265 -1.169879 -0.947545
C -6.496913 -2.640831 -1.257337
H -7.485056 -2.937105 -0.894861
H -5.762519 -3.298533 -0.787948
H -6.465830 -2.828261 -2.332725
H -6.921150 -0.545852 -1.508423
H -5.223860 -0.903157 -1.315056
H -7.364237 -0.998795 0.866177
H -6.173557 0.262695 0.655590
H -5.670493 -2.634547 1.489358
H -5.529518 -1.209015 2.491001

H -3.327277 -2.080045 1.820274
H -3.736646 -1.937952 0.127873
H -3.896935 0.573631 0.392925
H -3.515350 0.396270 2.098608
H -1.319560 -0.673322 1.426179
O -1.396067 1.360621 1.022824
H -2.352812 -0.706227 -1.245243
O -1.409016 1.116099 -1.209769
C 2.832887 -1.874135 -0.628276
O 2.345035 -2.851497 -1.163414
C 4.292848 -1.839796 -0.091689
C 4.269651 -2.571072 1.233143
C 4.246312 -2.026292 2.442769
H 4.263379 -0.955367 2.602795
H 4.203603 -2.646581 3.328939
H 4.224475 -3.651231 1.125132
C 4.613851 -0.341139 0.005297
H 5.166481 -0.046040 -0.888764
H 5.215094 -0.072954 0.871628
O 5.171686 -2.487534 -1.006224
H 4.731147 -3.295882 -1.307066
C 1.589997 3.510912 0.532003
H 2.181391 3.781021 1.408247
H 2.023272 4.060741 -0.308485
O -0.346670 3.655114 -1.611746
H -0.870159 3.161108 -2.255619
H 0.031702 5.003524 0.649091
H -0.225958 3.626320 1.709935

1b-c127, ΔG = 2.9819 kcal/mol, population = 0.05 %
C -0.179274 3.755620 1.435052
C -0.644245 3.604553 -0.004066
H -1.661571 3.987763 -0.124783
C -0.670536 2.127870 -0.369901
C 0.619204 1.404109 -0.031099
C 1.485290 1.881996 0.960790
N 2.608634 1.230423 1.318411
C 2.870542 0.096265 0.687149
C 2.067727 -0.443447 -0.324516
C 0.908569 0.223886 -0.715763
C -0.004252 -0.250759 -1.815056
H -0.223433 -1.313579 -1.698603
H 0.511944 -0.146595 -2.773173
C -1.291408 0.567109 -1.823838

C -2.139529 0.364549 -0.542388
C -3.642380 0.387020 -0.752953
C -4.193810 -0.831854 -1.501601
C -3.872899 -2.194170 -0.870104
C -4.272576 -2.343958 0.600635
C -5.767611 -2.171116 0.869758
C -6.132483 -2.406213 2.334438
H -5.866782 -3.419255 2.647659
H -7.202617 -2.272878 2.506903
H -5.600061 -1.708100 2.985945
H -6.083988 -1.165575 0.578509
H -6.328758 -2.864560 0.234118
H -3.964406 -3.337027 0.943902
H -3.714446 -1.629876 1.215347
H -4.379074 -2.968014 -1.455792
H -2.803192 -2.401855 -0.966998
H -3.820542 -0.829634 -2.529885
H -5.276912 -0.712074 -1.580723
H -3.898928 1.299474 -1.297994
H -4.113023 0.467556 0.229683
H -1.832812 -0.548109 -0.028718
O -1.765619 1.496503 0.284947
H -1.860402 0.410769 -2.735847
O -0.950101 1.966968 -1.752140
C 2.662839 -1.693379 -0.789975
O 2.398830 -2.361741 -1.771375
C 3.786554 -2.071137 0.217823
C 3.209393 -3.157464 1.099870
C 2.710721 -3.008657 2.320339
H 2.688682 -2.053591 2.830353
H 2.295921 -3.852715 2.856416
H 3.184694 -4.131680 0.619486
C 4.090859 -0.750415 0.940816
H 4.963890 -0.291295 0.473046
H 4.303924 -0.866605 2.001527
O 4.935525 -2.546134 -0.476264
H 4.624662 -3.129983 -1.183720
C 1.230838 3.197475 1.642405
H 1.449767 3.088020 2.705586
H 1.969728 3.902906 1.251105
O 0.262620 4.306604 -0.852262
H 0.007890 4.128831 -1.766704
H -0.206977 4.809218 1.714473
H -0.892428 3.231078 2.073228

1b-c171, ΔG = 2.9895 kcal/mol, population = 0.05 %

C -1.329316 3.081122 0.535400
C -1.766830 2.177893 -0.605717
H -2.846770 2.008026 -0.573182
C -1.091356 0.822275 -0.457601
C 0.406960 0.918317 -0.239200
C 1.000330 2.078962 0.275322
N 2.321984 2.173958 0.513548
C 3.059021 1.107305 0.241057
C 2.548736 -0.087086 -0.280600
C 1.183500 -0.197130 -0.546346
C 0.542031 -1.418370 -1.148763
H 0.863560 -2.319090 -0.622815
H 0.889819 -1.525061 -2.179716
C -0.979159 -1.293897 -1.124105
C -1.562646 -1.301132 0.310186
C -2.912039 -1.981914 0.475506
C -4.004102 -1.482622 -0.469320
C -5.371144 -2.128463 -0.220917
C -5.975000 -1.865724 1.165653
C -6.091894 -0.387223 1.553435
C -6.952220 0.438785 0.597502
H -6.516354 0.480869 -0.402671
H -7.059562 1.465816 0.952917
H -7.954138 0.010803 0.503765
H -6.514704 -0.327149 2.560654
H -5.094399 0.056977 1.619638
H -6.970885 -2.320185 1.198029
H -5.384960 -2.383463 1.927158
H -6.063849 -1.774341 -0.989223
H -5.289057 -3.210770 -0.365801
H -3.711777 -1.679057 -1.504041
H -4.083396 -0.397835 -0.380106
H -3.218697 -1.839605 1.514424
H -2.763716 -3.058773 0.342725
H -0.843646 -1.740495 1.005390
O -1.690904 0.110602 0.620064
H -1.448250 -2.027323 -1.773510
O -1.342319 0.018614 -1.599826
C 3.639839 -1.037419 -0.463446
O 3.648707 -2.100277 -1.054003
C 4.904049 -0.440946 0.228324
C 5.063835 -1.132248 1.561618

C 6.103606 -1.880046 1.905145
H 6.939824 -2.032934 1.234541
H 6.154651 -2.350103 2.878805
H 4.239944 -0.985129 2.254111
C 4.553444 1.051696 0.417947
H 5.046568 1.634228 -0.362688
H 4.863984 1.448741 1.383569
O 6.029005 -0.591027 -0.618854
H 6.011016 -1.495204 -0.964892
C 0.183576 3.314350 0.532287
H 0.507985 3.757876 1.474806
H 0.445658 4.033794 -0.248959
O -1.390321 2.786819 -1.839484
H -1.591454 2.162614 -2.548450
H -1.852626 4.034917 0.461975
H -1.639174 2.611552 1.470659

1b-c135, ΔG = 3.0321 kcal/mol, population = 0.05 %
C 1.612628 -2.743119 1.015058
C 1.934227 -2.158476 -0.350134
H 3.004913 -1.955472 -0.442040
C 1.206420 -0.831651 -0.509613
C -0.270149 -0.914195 -0.172043
C -0.774644 -1.915308 0.668776
N -2.072853 -1.982433 1.018927
C -2.874615 -1.046787 0.532174
C -2.454586 -0.016774 -0.317692
C -1.114945 0.059852 -0.699962
C -0.567787 1.101349 -1.638838
H -0.899757 2.097588 -1.341014
H -0.978655 0.926521 -2.636803
C 0.956472 1.034673 -1.685403
C 1.626034 1.431550 -0.345570
C 2.944150 2.166808 -0.514158
C 3.597748 2.648546 0.785336
C 4.100510 1.562344 1.746289
C 5.081531 0.555374 1.138980
C 6.348517 1.174638 0.548726
C 7.326712 0.123388 0.026584
H 7.642212 -0.550584 0.827478
H 8.222891 0.582407 -0.396676
H 6.863199 -0.485890 -0.754052
H 6.083523 1.854236 -0.266184
H 6.840165 1.788944 1.311048

H 5.369335 -0.160478 1.916573
H 4.571808 -0.028668 0.367615
H 4.583988 2.062443 2.592697
H 3.248575 1.014967 2.152989
H 2.882169 3.282776 1.319268
H 4.430832 3.303018 0.515499
H 2.739527 3.043435 -1.136420
H 3.625635 1.530463 -1.084807
H 0.938814 2.022114 0.266501
O 1.831283 0.151445 0.306492
H 1.355538 1.588974 -2.530234
O 1.356757 -0.343448 -1.833147
C -3.598839 0.819378 -0.661865
O -3.695529 1.687557 -1.507750
C -4.785898 0.391279 0.254045
C -4.885843 1.403440 1.371113
C -5.931750 2.189637 1.586126
H -6.815623 2.141609 0.962716
H -5.938990 2.895965 2.406265
H -4.012714 1.461873 2.014793
C -4.355821 -0.989630 0.797194
H -4.869942 -1.769433 0.231959
H -4.586460 -1.128482 1.852661
O -5.971246 0.285467 -0.513666
H -6.014768 1.065239 -1.086126
C 0.114987 -3.013564 1.181295
H -0.129931 -3.201017 2.227800
H -0.159738 -3.921910 0.637153
O 1.507675 -3.079330 -1.352594
H 1.631950 -2.656647 -2.211987
H 2.174783 -3.667006 1.154086
H 1.957662 -2.035508 1.770907

1b-c197, ΔG = 3.0710 kcal/mol, population = 0.05 %
C 2.120956 2.713443 -1.848598
C 2.428485 2.761929 -0.360935
H 3.508409 2.736444 -0.188897
C 1.833459 1.534436 0.309361
C 0.366573 1.321518 -0.023851
C -0.183193 1.813016 -1.216249
N -1.453834 1.567052 -1.585587
C -2.184321 0.825322 -0.766326
C -1.721940 0.314899 0.452121
C -0.412907 0.574363 0.858757

C 0.177880 0.086921 2.156401
H -0.098251 -0.953798 2.333840
H -0.240792 0.666114 2.983739
C 1.692920 0.245353 2.111437
C 2.306157 -0.600260 0.960622
C 3.574791 -1.362091 1.291013
C 4.182677 -2.118296 0.099407
C 3.569009 -3.492183 -0.212388
C 2.069782 -3.534266 -0.534303
C 1.635571 -2.667247 -1.719098
C 0.116185 -2.589020 -1.862948
H -0.332206 -2.141517 -0.971927
H -0.324722 -3.581092 -1.990737
H -0.175199 -1.978204 -2.719921
H 2.030373 -1.655011 -1.611479
H 2.076975 -3.069448 -2.636734
H 1.791319 -4.573295 -0.738862
H 1.494860 -3.253702 0.353530
H 4.120585 -3.920113 -1.056343
H 3.755324 -4.155216 0.639041
H 5.244146 -2.276786 0.306392
H 4.144753 -1.476163 -0.783179
H 3.349861 -2.061717 2.102634
H 4.301057 -0.644377 1.682588
H 1.542442 -1.275385 0.581864
O 2.579948 0.384937 -0.064899
H 2.160048 0.063318 3.075452
O 1.993717 1.601664 1.717986
C -2.785113 -0.461654 1.080912
O -2.857843 -0.902641 2.211576
C -3.916947 -0.642538 0.024670
C -3.753775 -2.017028 -0.582012
C -4.648320 -2.991861 -0.494759
H -5.590329 -2.853771 0.020819
H -4.468424 -3.955494 -0.953765
H -2.819559 -2.174788 -1.112481
C -3.621259 0.454979 -1.022125
H -4.271697 1.311118 -0.832482
H -3.781027 0.127347 -2.048464
O -5.180388 -0.443593 0.630391
H -5.169414 -0.909516 1.479254
C 0.615233 2.705416 -2.125517
H 0.414749 2.420103 -3.159184
H 0.210971 3.715055 -2.005250

O 1.858917 3.949363 0.186581
H 1.993167 3.927723 1.142690
H 2.584398 3.569084 -2.340537
H 2.584819 1.813420 -2.255326

1b-c86, ΔG = 3.0792 kcal/mol, population = 0.04 %
C -0.132349 -4.228067 -0.461923
C -1.108857 -3.472142 0.423896
H -2.137481 -3.783738 0.221053
C -1.017646 -1.985195 0.114177
C 0.405645 -1.458047 0.125522
C 1.501770 -2.306795 -0.082290
N 2.770863 -1.858796 -0.115650
C 2.953253 -0.557919 0.056116
C 1.917764 0.356496 0.280751
C 0.598441 -0.093323 0.333978
C -0.580370 0.799184 0.614152
H -0.537025 1.695857 -0.006737
H -0.527949 1.141804 1.650990
C -1.882904 0.041897 0.380656
C -2.090647 -0.351589 -1.107146
C -3.521521 -0.298456 -1.618965
C -3.955726 1.086698 -2.114847
C -4.001989 2.202810 -1.065515
C -5.054667 1.994141 0.024145
C -5.114125 3.124222 1.056985
C -3.867728 3.227839 1.937950
H -3.979745 4.015602 2.685767
H -2.973786 3.456876 1.354741
H -3.686942 2.290044 2.470670
H -5.276043 4.075781 0.539487
H -5.988380 2.970404 1.696042
H -6.032927 1.894209 -0.457182
H -4.881577 1.047898 0.547360
H -3.012842 2.335092 -0.619584
H -4.218030 3.146206 -1.576898
H -4.946997 0.989833 -2.567504
H -3.280014 1.388653 -2.921310
H -4.194391 -0.676069 -0.844571
H -3.587273 -0.998264 -2.455212
H -1.440626 0.248041 -1.750587
O -1.627072 -1.726345 -1.145489
H -2.732539 0.565086 0.802765
O -1.797248 -1.239631 1.035486

C 2.480743 1.693960 0.428460
O 1.951539 2.717933 0.815498
C 3.982534 1.617686 0.017608
C 4.095005 2.141275 -1.395140
C 4.783722 3.218006 -1.748367
H 5.340091 3.798746 -1.023535
H 4.823418 3.540537 -2.780839
H 3.551107 1.567290 -2.139866
C 4.304276 0.107404 0.070806
H 4.822348 -0.112488 1.006379
H 4.933237 -0.228958 -0.752404
O 4.772718 2.337384 0.946172
H 4.321562 3.176456 1.119558
C 1.314723 -3.792291 -0.219143
H 1.965690 -4.153824 -1.016563
H 1.687423 -4.242935 0.705354
O -0.768721 -3.714442 1.787727
H -1.337157 -3.158804 2.336248
H -0.233737 -5.298326 -0.279766
H -0.414311 -4.047028 -1.500616

1b-c191, ΔG = 3.1294 kcal/mol, population = 0.04 %
C -2.534411 2.311244 1.857227
C -2.807701 2.388284 0.363822
H -3.869134 2.224330 0.156580
C -2.032671 1.285911 -0.339296
C -0.558744 1.262059 0.030279
C -0.111407 1.773311 1.257006
N 1.174067 1.700345 1.648962
C 2.023731 1.116687 0.816537
C 1.666367 0.600884 -0.434064
C 0.340646 0.678185 -0.861348
C -0.143838 0.166812 -2.192720
H 0.278047 -0.818424 -2.398674
H 0.211156 0.830133 -2.985885
C -1.666238 0.113464 -2.187948
C -2.188748 -0.860110 -1.093784
C -3.316000 -1.781912 -1.503483
C -3.809188 -2.706565 -0.380864
C -2.692648 -3.530481 0.288269
C -2.125550 -2.888325 1.563636
C -0.777126 -3.463089 2.009372
C 0.396740 -2.998697 1.144391
H 0.278689 -3.296366 0.100283

H 1.339910 -3.420511 1.498172
H 0.486988 -1.909418 1.169417
H -0.588071 -3.165430 3.044930
H -0.825900 -4.557431 2.009748
H -2.022230 -1.809519 1.427289
H -2.860513 -3.017636 2.364322
H -3.072267 -4.523043 0.545164
H -1.891502 -3.701551 -0.437771
H -4.558471 -3.374947 -0.811252
H -4.327557 -2.112841 0.377068
H -2.944829 -2.395471 -2.330893
H -4.143669 -1.182357 -1.892880
H -1.350408 -1.448929 -0.718750
O -2.626042 0.030373 -0.040353
H -2.078716 -0.086952 -3.172924
O -2.162384 1.396163 -1.748529
C 2.847141 0.034266 -1.076333
O 3.000080 -0.337856 -2.223611
C 3.986566 -0.004267 -0.012497
C 4.074560 -1.420404 0.505900
C 5.129482 -2.211529 0.366330
H 6.031532 -1.873948 -0.128179
H 5.125345 -3.219944 0.759646
H 3.183596 -1.777868 1.012777
C 3.495814 0.959100 1.091090
H 4.005947 1.917724 0.978438
H 3.683968 0.591424 2.099016
O 5.195469 0.459217 -0.586053
H 5.284275 0.037741 -1.453244
C -1.050039 2.498368 2.181077
H -0.837374 2.192049 3.206277
H -0.788783 3.559072 2.120658
O -2.392378 3.666583 -0.113065
H -2.495070 3.671035 -1.073308
H -3.124901 3.070211 2.371228
H -2.878897 1.337444 2.208865

1b-c365, ΔG = 3.1426 kcal/mol, population = 0.04 %
C 0.303628 3.765345 -1.221658
C 0.831450 3.413333 0.159540
H 1.892806 3.663569 0.244540
C 0.701855 1.912917 0.378256
C -0.681062 1.376607 0.055125
C -1.544034 2.056350 -0.815085

N -2.758563 1.581994 -1.149139
C -3.117983 0.423465 -0.615325
C -2.324283 -0.314551 0.267886
C -1.069054 0.170252 0.636526
C -0.157165 -0.519194 1.616075
H -0.063890 -1.578760 1.372506
H -0.608653 -0.472583 2.610722
C 1.211800 0.152319 1.632792
C 1.980105 -0.002622 0.296622
C 3.483013 -0.148719 0.441805
C 3.891735 -1.531630 0.949157
C 5.400160 -1.682219 1.174677
C 6.272418 -1.473315 -0.067947
C 5.970888 -2.433251 -1.218942
C 6.931375 -2.262753 -2.394668
H 7.964232 -2.445937 -2.086859
H 6.881308 -1.246835 -2.795633
H 6.696990 -2.953614 -3.207396
H 6.020165 -3.463157 -0.848991
H 4.945519 -2.283355 -1.568798
H 6.176268 -0.442952 -0.425542
H 7.321340 -1.591064 0.223697
H 5.712564 -0.975153 1.949981
H 5.592183 -2.682637 1.575869
H 3.541804 -2.290968 0.243865
H 3.381638 -1.741786 1.894072
H 3.849749 0.629834 1.118414
H 3.931343 0.041933 -0.535578
H 1.572981 -0.835103 -0.283265
O 1.681672 1.233593 -0.401142
H 1.799582 -0.157228 2.492418
O 1.028396 1.580626 1.718684
C -3.020860 -1.541947 0.652595
O -2.715114 -2.347853 1.505693
C -4.278227 -1.664246 -0.268821
C -3.935067 -2.648892 -1.361968
C -4.398793 -3.889976 -1.435841
H -5.073061 -4.293829 -0.690966
H -4.117245 -4.540882 -2.253453
H -3.249734 -2.274114 -2.115955
C -4.446645 -0.238135 -0.856868
H -5.229169 0.284795 -0.302974
H -4.719299 -0.235122 -1.912089
O -5.355793 -2.085047 0.546436

H -6.112843 -2.268589 -0.024609
C -1.174420 3.401104 -1.376951
H -1.472579 3.435573 -2.425866
H -1.793692 4.139809 -0.859834
O 0.067832 4.122331 1.133696
H 0.349985 3.818632 2.005991
H 0.444877 4.831553 -1.401305
H 0.906391 3.229244 -1.956849

1b-c73, ΔG = 3.2047 kcal/mol, population = 0.04 %
C -0.192292 3.793639 0.619725
C -1.038327 3.022056 -0.379591
H -2.102966 3.204575 -0.208334
C -0.798943 1.531141 -0.192350
C 0.671337 1.158324 -0.161669
C 1.656251 2.092321 0.183565
N 2.962658 1.773354 0.264155
C 3.289963 0.517596 0.000917
C 2.371584 -0.473947 -0.363793
C 1.017865 -0.158409 -0.463380
C -0.044586 -1.138907 -0.884128
H 0.056801 -2.074355 -0.331110
H 0.103039 -1.387399 -1.938533
C -1.433501 -0.540903 -0.680215
C -1.770580 -0.300838 0.813111
C -3.218274 -0.547221 1.205481
C -4.247006 0.255468 0.410543
C -5.687168 0.035126 0.885308
C -6.201098 -1.405509 0.783987
C -6.179868 -1.985424 -0.630379
C -6.797184 -3.381060 -0.704292
H -6.261878 -4.079464 -0.055418
H -6.766168 -3.779905 -1.720537
H -7.841826 -3.365759 -0.382660
H -6.717158 -1.308793 -1.303821
H -5.151192 -2.029182 -0.999105
H -5.623237 -2.057557 1.447142
H -7.229094 -1.433349 1.160541
H -5.771065 0.367359 1.925495
H -6.347390 0.685298 0.301991
H -4.168281 0.014828 -0.652395
H -4.006397 1.316833 0.497187
H -3.323421 -0.321303 2.271190
H -3.400070 -1.620707 1.100125

H -1.114180 -0.903646 1.445214
O -1.431806 1.095933 1.006997
H -2.196060 -1.116909 -1.196627
O -1.443096 0.795890 -1.221011
C 3.086113 -1.727594 -0.586743
O 2.699977 -2.761366 -1.098626
C 4.535103 -1.533740 -0.053732
C 4.585956 -2.236422 1.285743
C 4.503467 -1.672669 2.483978
H 4.408792 -0.602753 2.622456
H 4.523448 -2.276028 3.382558
H 4.653146 -3.317429 1.199789
C 4.702063 -0.008198 0.011884
H 5.226486 0.322819 -0.886568
H 5.268892 0.337729 0.873909
O 5.475229 -2.107585 -0.956649
H 5.117428 -2.960928 -1.242632
C 1.303363 3.533852 0.423520
H 1.873757 3.895761 1.280173
H 1.665622 4.095190 -0.442713
O -0.665129 3.421305 -1.697203
H -1.144834 2.861110 -2.320702
H -0.400461 4.859527 0.522018
H -0.501715 3.492514 1.622148

1b-c240, ΔG = 3.3271 kcal/mol, population = 0.03 %
C 0.558301 -3.930286 1.265563
C 1.320213 -3.639832 -0.017296
H 2.363123 -3.957214 0.072326
C 1.322767 -2.140280 -0.269112
C -0.061157 -1.517950 -0.192910
C -1.085916 -2.111285 0.557668
N -2.297351 -1.543641 0.708751
C -2.492966 -0.376677 0.112275
C -1.534258 0.271528 -0.674398
C -0.281044 -0.311964 -0.856888
C 0.804421 0.283484 -1.711786
H 0.885596 1.356139 -1.534682
H 0.534979 0.164849 -2.764826
C 2.123243 -0.423144 -1.428685
C 2.576527 -0.250774 0.048975
C 4.065285 -0.031724 0.247787
C 4.549207 1.371710 -0.157064
C 4.494665 2.430084 0.955241

C 3.113301 2.734465 1.545798
C 2.118884 3.337916 0.551912
C 0.699711 3.431900 1.108240
H 0.317230 2.441435 1.369916
H 0.011770 3.869651 0.382740
H 0.669724 4.044785 2.012688
H 2.470018 4.331207 0.255158
H 2.103099 2.746028 -0.366402
H 2.690048 1.825583 1.984555
H 3.237177 3.429383 2.382393
H 5.155708 2.106415 1.765587
H 4.922918 3.359167 0.564566
H 3.994601 1.717715 -1.034313
H 5.588100 1.293166 -0.484776
H 4.593146 -0.794863 -0.330034
H 4.301521 -0.215023 1.299559
H 2.000194 0.546327 0.516580
O 2.188766 -1.504014 0.662880
H 2.894965 -0.152545 -2.143964
O 1.904343 -1.845948 -1.530132
C -2.085208 1.528562 -1.167654
O -1.652780 2.283752 -2.016629
C -3.425513 1.779362 -0.411702
C -3.141698 2.771873 0.691069
C -3.667299 3.986728 0.767973
H -4.372356 4.348607 0.030253
H -3.416298 4.651531 1.584407
H -2.440838 2.427953 1.445721
C -3.785417 0.394410 0.171743
H -4.535620 -0.076080 -0.466816
H -4.184030 0.443438 1.184227
O -4.415655 2.220496 -1.322348
H -4.004490 2.883632 -1.895901
C -0.897566 -3.463285 1.187817
H -1.353435 -3.454233 2.178806
H -1.480945 -4.170604 0.590770
O 0.677410 -4.319432 -1.094057
H 1.120900 -4.061318 -1.912207
H 0.594036 -5.000594 1.470915
H 1.075377 -3.425394 2.083229

1b-c129, ΔG = 3.3434 kcal/mol, population = 0.03 %
C 0.055173 -3.868068 1.601182
C 0.611467 -3.827076 0.187309

H 1.581047 -4.331120 0.138900
C 0.832387 -2.379150 -0.221386
C -0.383508 -1.498217 0.010965
C -1.338155 -1.820805 0.984242
N -2.375595 -1.013979 1.279817
C -2.462748 0.121986 0.604991
C -1.572265 0.505656 -0.404142
C -0.503820 -0.324483 -0.734662
C 0.496097 -0.015575 -1.816907
H 0.796265 1.032231 -1.772660
H 0.026096 -0.161364 -2.793202
C 1.699956 -0.936547 -1.670512
C 2.414306 -0.731717 -0.304665
C 3.929488 -0.728717 -0.336757
C 4.529009 0.471356 -1.086215
C 3.956691 1.840116 -0.697615
C 4.085723 2.190673 0.785677
C 3.396362 3.504974 1.169217
C 1.868935 3.442542 1.099438
H 1.481078 2.643351 1.736592
H 1.511077 3.257118 0.084822
H 1.419461 4.379121 1.435216
H 3.692759 3.774018 2.187186
H 3.761651 4.308521 0.520817
H 3.667006 1.388872 1.402102
H 5.148617 2.245967 1.042041
H 4.466449 2.609194 -1.285910
H 2.907321 1.891702 -0.997911
H 4.391553 0.331289 -2.162296
H 5.609611 0.468375 -0.918128
H 4.274002 -1.658643 -0.796288
H 4.280938 -0.739501 0.697422
H 2.045731 0.182512 0.163317
O 1.949378 -1.852898 0.485366
H 2.372940 -0.873807 -2.520681
O 1.222350 -2.295989 -1.583456
C -1.967108 1.814915 -0.917138
O -1.590178 2.407397 -1.910320
C -3.031310 2.387254 0.061961
C -2.295968 3.385375 0.931105
C -1.834646 3.182735 2.158215
H -1.969632 2.247423 2.687151
H -1.288665 3.958095 2.679724
H -2.108990 4.332047 0.432054

C -3.549009 1.147020 0.806467
H -4.470841 0.812464 0.326756
H -3.764798 1.322257 1.858449
O -4.080581 3.023756 -0.658896
H -3.671289 3.537304 -1.370739
C -1.283073 -3.133439 1.715540
H -1.540752 -2.962516 2.761608
H -2.083024 -3.755627 1.303012
O -0.324383 -4.449987 -0.690673
H 0.003868 -4.346875 -1.593038
H -0.063715 -4.906851 1.910569
H 0.794009 -3.414398 2.263764

1b-c151, ΔG = 3.3773 kcal/mol, population = 0.03 %
C -2.857362 -2.709657 -1.619113
C -3.275762 -2.192594 -0.251894
H -4.298544 -1.806106 -0.281779
C -2.366547 -1.041032 0.144999
C -0.888452 -1.370330 0.013397
C -0.439302 -2.346117 -0.887881
N 0.867107 -2.591608 -1.101495
C 1.735708 -1.863233 -0.414940
C 1.373041 -0.895026 0.527781
C 0.024784 -0.639805 0.773303
C -0.467868 0.366124 1.778832
H 0.120998 1.282393 1.721177
H -0.324858 -0.030855 2.787370
C -1.942267 0.646254 1.525381
C -2.169524 1.230984 0.101524
C -3.138773 2.390651 -0.002581
C -2.629489 3.676340 0.669031
C -1.186239 4.067436 0.325155
C -0.906173 4.243928 -1.168162
C 0.573831 4.475800 -1.493208
C 1.458763 3.251292 -1.246941
H 1.485087 2.967835 -0.193273
H 2.488767 3.440066 -1.555875
H 1.094983 2.388681 -1.812249
H 0.661367 4.768885 -2.543368
H 0.946747 5.322128 -0.906498
H -1.247397 3.364804 -1.723603
H -1.499558 5.085161 -1.540376
H -0.947269 5.002125 0.841351
H -0.503215 3.323068 0.741418

H -2.709933 3.573544 1.755003
H -3.303896 4.491625 0.392718
H -4.093909 2.096147 0.439551
H -3.324870 2.570147 -1.063849
H -1.207124 1.508786 -0.330733
O -2.677264 0.100439 -0.645465
H -2.392951 1.243401 2.312760
O -2.639165 -0.616899 1.471656
C 2.577583 -0.288037 1.082900
O 2.704752 0.440123 2.048174
C 3.778473 -0.756756 0.208309
C 4.085879 0.361837 -0.760631
C 5.225653 1.038307 -0.795492
H 6.042504 0.809827 -0.122564
H 5.378521 1.835014 -1.512036
H 3.281589 0.605348 -1.448554
C 3.231107 -1.996149 -0.535511
H 3.574080 -2.897374 -0.023517
H 3.552236 -2.052263 -1.574734
O 4.874835 -1.101700 1.033852
H 4.956625 -0.411555 1.708255
C -1.414675 -3.222034 -1.623882
H -1.058142 -3.363326 -2.645003
H -1.371768 -4.205922 -1.147134
O -3.166406 -3.256568 0.691728
H -3.368252 -2.898589 1.565611
H -3.535302 -3.506953 -1.925146
H -2.971576 -1.893289 -2.334101

1b-c165, ΔG = 3.5216 kcal/mol, population = 0.02 %
C -1.500135 2.870811 1.028024
C -2.047353 1.907725 -0.011889
H -3.099100 1.689172 0.184945
C -1.282375 0.593741 0.069352
C 0.224929 0.781423 0.047292
C 0.818222 1.989769 0.439408
N 2.151992 2.169481 0.465040
C 2.903819 1.141821 0.099384
C 2.394860 -0.093534 -0.316678
C 1.014463 -0.291533 -0.362954
C 0.366117 -1.563623 -0.838896
H 0.831730 -2.430928 -0.367595
H 0.538934 -1.667818 -1.913479
C -1.130952 -1.532760 -0.552188

C -1.447093 -1.518457 0.966180
C -2.631695 -2.369519 1.400882
C -3.955916 -2.164187 0.655431
C -4.758214 -0.939194 1.095859
C -6.134036 -0.828829 0.430672
C -6.117055 -0.749201 -1.101202
C -5.273529 0.399114 -1.655512
H -5.573630 1.355182 -1.217270
H -5.384482 0.479720 -2.739066
H -4.213819 0.251321 -1.443465
H -5.755831 -1.694593 -1.516590
H -7.148294 -0.642856 -1.450923
H -6.746792 -1.686004 0.730796
H -6.638063 0.061397 0.822183
H -4.897182 -0.982794 2.181217
H -4.179037 -0.036620 0.906918
H -4.572059 -3.055284 0.813205
H -3.769942 -2.118579 -0.420228
H -2.782576 -2.217565 2.473824
H -2.310059 -3.409238 1.284760
H -0.564965 -1.835031 1.528864
O -1.673295 -0.112458 1.241424
H -1.655973 -2.319021 -1.086330
O -1.658124 -0.265594 -0.996583
C 3.501670 -0.981427 -0.654333
O 3.484231 -2.058378 -1.218702
C 4.819322 -0.290737 -0.185745
C 5.241743 -0.941040 1.110408
C 6.373218 -1.610172 1.285188
H 7.095849 -1.720361 0.486618
H 6.616307 -2.054364 2.241809
H 4.534283 -0.834074 1.927770
C 4.407693 1.180909 0.041376
H 4.736412 1.777814 -0.811533
H 4.837985 1.614429 0.943320
O 5.798023 -0.386831 -1.205519
H 5.786423 -1.298162 -1.532310
C -0.021394 3.185217 0.793423
H 0.414282 3.672255 1.667072
H 0.079568 3.895196 -0.032696
O -1.892098 2.490608 -1.304109
H -2.162721 1.833781 -1.958374
H -2.084110 3.791447 1.009744
H -1.640728 2.416129 2.010149

1b-c87, ΔG = 3.7337 kcal/mol, population = 0.01 %
C 1.775707 3.501974 -1.444852
C 2.325870 3.066065 -0.096816
H 3.418753 3.026906 -0.119649
C 1.828438 1.662987 0.215171
C 0.324818 1.511804 0.058569
C -0.406237 2.352506 -0.791016
N -1.725806 2.193202 -1.010905
C -2.320814 1.188070 -0.387093
C -1.670899 0.315557 0.493220
C -0.311278 0.479399 0.748818
C 0.473461 -0.377652 1.705520
H 0.219139 -1.429501 1.574110
H 0.192539 -0.118353 2.729930
C 1.964393 -0.144424 1.498925
C 2.427627 -0.543357 0.069378
C 3.780585 -1.228659 -0.007928
C 3.783669 -2.684367 0.488693
C 3.437355 -3.741258 -0.571685
C 2.040927 -3.656226 -1.197381
C 0.892616 -3.863982 -0.208413
C -0.478662 -3.578804 -0.815931
H -0.666904 -4.216078 -1.683824
H -0.553449 -2.540979 -1.149794
H -1.281303 -3.747421 -0.095719
H 0.928975 -4.891287 0.167928
H 1.034695 -3.225375 0.666929
H 1.919849 -2.695097 -1.706267
H 1.965421 -4.412719 -1.984973
H 4.182548 -3.678253 -1.371105
H 3.558836 -4.730913 -0.118702
H 3.121709 -2.785799 1.353962
H 4.784237 -2.912150 0.862876
H 4.487283 -0.630945 0.573861
H 4.120997 -1.194226 -1.046592
H 1.656966 -1.145962 -0.409242
O 2.496816 0.727243 -0.622583
H 2.562763 -0.601099 2.282413
O 2.208207 1.277843 1.527385
C -2.629056 -0.671863 0.982787
O -2.522297 -1.474829 1.890020
C -3.915030 -0.532382 0.117651
C -3.877929 -1.675386 -0.872807

C -3.504557 -1.614803 -2.144058
H -3.197574 -0.692444 -2.621192
H -3.482748 -2.506318 -2.757494
H -4.145997 -2.632007 -0.433197
C -3.787803 0.865044 -0.507323
H -4.372105 1.567506 0.090151
H -4.144170 0.924310 -1.533646
O -5.075860 -0.615762 0.938588
H -4.936102 -1.341233 1.564734
C 0.245245 3.530528 -1.460405
H -0.128387 3.602817 -2.482760
H -0.113775 4.424428 -0.941651
O 1.875040 3.982107 0.899124
H 2.165721 3.650232 1.758224
H 2.168169 4.488928 -1.691498
H 2.148903 2.806145 -2.198068

Table S4. Geometry data of conformers of structure **2a**.

2a-c29, ΔG = 0.0000 kcal/mol, population = 18.68 %
C 1.053668 -5.634028 -0.022020
C 2.021122 -4.806340 -0.864622
C 1.980790 -3.352309 -0.499618
C 0.904237 -2.744792 0.081193
C -0.245051 -3.541294 0.424791
C -0.318875 -4.987217 -0.048207
C 0.856092 -1.259696 0.408884
N 3.120836 -2.635234 -0.780273
C 3.235828 -1.330092 -0.406516
C 2.211760 -0.661820 0.177935
C 4.498742 -0.539142 -0.547360
C 4.074913 0.878429 -0.103606
C 2.661176 0.662048 0.519156
O 2.106133 1.518238 1.194398
O 4.960458 1.368917 0.891259
C 3.939358 1.804038 -1.286342
C 4.513629 2.995058 -1.386862
C -0.235791 -0.518754 -0.421226
C -1.137685 0.366065 0.429851
C -2.101219 1.206049 -0.418255
C -2.790536 2.293297 0.403411
C -3.686980 3.209602 -0.425401
C -4.377098 4.287654 0.408595
C -5.274790 5.209041 -0.415290

C -5.968021 6.288882 0.414725
C -6.862314 7.203864 -0.420078
O -1.201196 -3.096170 1.076696
O -1.244064 -5.716387 0.735640
O -1.936527 -0.411862 1.338172
O -3.052404 0.344527 -1.059029
H 0.994712 -6.652148 -0.406641
H 1.402909 -5.683182 1.011955
H 1.765849 -4.897085 -1.926530
H 3.040537 -5.180444 -0.755550
H -0.669313 -4.941200 -1.092080
H 0.598026 -1.153341 1.467053
H 3.919060 -3.118629 -1.165823
H 5.272025 -0.917536 0.123637
H 4.889447 -0.572380 -1.565440
H 4.483195 2.055604 1.379763
H 3.303513 1.430875 -2.084316
H 4.367012 3.613636 -2.262947
H 5.155562 3.381748 -0.605474
H 0.252848 0.098781 -1.177397
H -0.851648 -1.239829 -0.961933
H -0.508749 1.045581 1.013598
H -1.533819 1.675574 -1.226205
H -3.374917 1.816762 1.196930
H -2.016246 2.889858 0.897897
H -3.087334 3.687430 -1.209041
H -4.442158 2.607504 -0.937987
H -4.974241 3.809134 1.193356
H -3.619418 4.888574 0.924680
H -4.678249 5.686925 -1.201101
H -6.033373 4.608217 -0.930281
H -6.564044 5.811085 1.199588
H -5.209441 6.888503 0.929061
H -7.649400 6.632784 -0.919668
H -7.343655 7.965747 0.196991
H -6.284298 7.716849 -1.193361
H -1.828410 -5.052646 1.138079
H -1.655332 -1.346017 1.298915
H -3.363033 -0.249098 -0.358961

2a-c80, ΔG = 0.0452 kcal/mol, population = 17.31 %
C 0.233346 -5.336489 -0.228502
C 1.617819 -4.762562 -0.518841
C 1.667442 -3.276045 -0.322295

C 0.580258 -2.459477 -0.395842
C -0.727996 -3.054930 -0.579514
C -0.815199 -4.526557 -0.971250
C 0.661493 -0.945054 -0.255006
N 2.920385 -2.760207 -0.041844
C 3.081592 -1.447267 0.252310
C 2.045272 -0.569708 0.188010
C 4.373389 -0.849368 0.712078
C 4.051925 0.659232 0.806493
C 2.514015 0.724179 0.574571
O 1.877101 1.767319 0.730394
O 4.352733 1.131358 2.112614
C 4.740793 1.440926 -0.282991
C 5.512102 2.501151 -0.085824
C 0.303935 -0.204057 -1.577744
C -1.086622 0.432752 -1.664829
C -1.372792 1.444360 -0.545499
C -2.790407 1.995901 -0.613916
C -3.162744 2.863272 0.585869
C -4.574973 3.438291 0.493296
C -4.960255 4.296298 1.696997
C -6.367424 4.884822 1.602177
C -6.742515 5.738479 2.812280
O -1.775524 -2.422617 -0.437338
O -2.119247 -5.017875 -0.728064
O -1.213369 1.077220 -2.942445
O -0.447906 2.538333 -0.640738
H 0.186186 -6.381591 -0.534990
H 0.023866 -5.289848 0.842887
H 1.902580 -4.977006 -1.555184
H 2.367777 -5.238605 0.115318
H -0.597634 -4.568661 -2.050213
H -0.050397 -0.642617 0.515464
H 3.697391 -3.396841 0.059105
H 4.650427 -1.229822 1.696727
H 5.190082 -1.069917 0.023551
H 3.896558 1.977589 2.224856
H 4.549749 1.079633 -1.289589
H 5.969397 3.020933 -0.917863
H 5.721250 2.874710 0.908689
H 1.037902 0.588724 -1.741446
H 0.414682 -0.893929 -2.416304
H -1.849731 -0.343025 -1.636563
H -1.246371 0.946937 0.421257

H -2.899909 2.568898 -1.538660
H -3.482370 1.150649 -0.684136
H -3.073903 2.268304 1.502349
H -2.441994 3.680448 0.675792
H -4.660170 4.038288 -0.420002
H -5.295410 2.618694 0.388791
H -4.882598 3.694350 2.609857
H -4.235624 5.111574 1.806212
H -6.443313 5.487906 0.691091
H -7.090764 4.069987 1.490707
H -7.750955 6.147140 2.717438
H -6.706024 5.150346 3.733150
H -6.051858 6.577953 2.928654
H -2.676609 -4.226797 -0.628567
H -0.713347 1.903290 -2.870972
H 0.365841 2.288880 -0.163533

2a-c1, ΔG = 0.3947 kcal/mol, population = 9.59 %
C -1.886281 -3.321892 -0.535268
C -0.732370 -2.863421 -1.422579
C 0.238247 -1.988815 -0.684442
C -0.075724 -1.269125 0.432011
C -1.408310 -1.393387 0.984494
C -2.476387 -2.111542 0.163650
C 0.924763 -0.371368 1.156029
N 1.513983 -1.961473 -1.204975
C 2.513448 -1.294816 -0.565431
C 2.283220 -0.557060 0.546657
C 3.944619 -1.346402 -0.998116
C 4.625442 -0.320617 -0.066615
C 3.533006 -0.016780 1.004623
O 3.785812 0.578610 2.044789
O 5.759962 -0.897797 0.562388
C 4.939007 0.956861 -0.803344
C 6.117316 1.565519 -0.799814
C 0.472599 1.109922 1.175186
C 0.288396 1.757577 -0.190264
C -0.528942 3.055682 -0.138334
C -1.958910 2.907711 0.369727
C -2.813034 1.867472 -0.353035
C -4.234882 1.794013 0.200621
C -5.112336 0.767060 -0.512257
C -6.532009 0.684754 0.046350
C -7.388937 -0.363977 -0.660337

O -1.729395 -0.930523 2.081279
O -3.572122 -2.457575 0.986938
O 1.550097 2.043804 -0.834628
O -0.578185 3.626464 -1.453091
H -2.649383 -3.818562 -1.134779
H -1.528966 -4.032503 0.213818
H -1.120093 -2.295065 -2.275747
H -0.202111 -3.722993 -1.835833
H -2.804222 -1.395165 -0.604895
H 0.960669 -0.678187 2.206289
H 1.730033 -2.544637 -2.000148
H 4.369724 -2.337289 -0.828339
H 4.057300 -1.107480 -2.056661
H 5.928937 -0.383339 1.365255
H 4.099977 1.377592 -1.349634
H 6.270607 2.488062 -1.345021
H 6.965548 1.156668 -0.265223
H -0.465458 1.150397 1.724447
H 1.198387 1.689523 1.752440
H -0.204024 1.065639 -0.874865
H 0.000321 3.751021 0.530042
H -1.928886 2.680166 1.437843
H -2.433919 3.889478 0.284771
H -2.843477 2.096574 -1.421369
H -2.350752 0.882022 -0.262446
H -4.191976 1.551516 1.268503
H -4.702581 2.782522 0.128973
H -5.156693 1.007358 -1.580717
H -4.647876 -0.221332 -0.437868
H -6.482337 0.455737 1.116180
H -7.010078 1.666689 -0.035439
H -8.398403 -0.403720 -0.245352
H -7.475091 -0.144712 -1.727936
H -6.947311 -1.359106 -0.561434
H -3.472710 -1.913669 1.787593
H 2.094412 2.548443 -0.215585
H 0.306110 3.503121 -1.826970

2a-c63, ΔG = 0.4267 kcal/mol, population = 9.08 %
C 0.367830 -5.568262 0.196750
C 1.102630 -4.702155 -0.823768
C 1.050104 -3.247679 -0.462646
C 0.071809 -2.695394 0.313563
C -0.953838 -3.545872 0.861978

C -1.022054 -5.004419 0.427100
C 0.002456 -1.211465 0.638316
N 2.070091 -2.469197 -0.960880
C 2.183015 -1.156599 -0.612538
C 1.255911 -0.541209 0.161154
C 3.350046 -0.299186 -0.991442
C 2.960526 1.090278 -0.439604
C 1.696907 0.802350 0.428691
O 1.237182 1.627336 1.206445
O 3.996870 1.592187 0.391631
C 2.577750 2.039031 -1.546391
C 3.085683 3.252202 -1.714139
C -1.263642 -0.554613 0.013435
C -2.021972 0.324348 0.996005
C -3.262966 0.969520 0.375714
C -2.982488 1.915381 -0.786250
C -1.897214 2.963108 -0.540347
C -1.806824 3.983223 -1.677256
C -0.562912 4.872376 -1.615981
C -0.449729 5.727013 -0.353396
C 0.789037 6.621226 -0.362105
O -1.811236 -3.139114 1.659672
O -1.734340 -5.769949 1.380804
O -2.447860 -0.416812 2.156038
O -3.963282 1.699557 1.395239
H 0.300373 -6.596007 -0.159844
H 0.910722 -5.574231 1.144808
H 0.649582 -4.824040 -1.814207
H 2.143210 -5.017864 -0.915030
H -1.566626 -5.003074 -0.531133
H -0.070923 -1.102793 1.723647
H 2.806091 -2.910805 -1.492657
H 4.263884 -0.644963 -0.505136
H 3.520595 -0.300057 -2.069036
H 3.597037 2.260672 0.967089
H 1.811433 1.665972 -2.220124
H 2.755178 3.888918 -2.524228
H 3.850404 3.643149 -1.055072
H -0.962603 0.037586 -0.851034
H -1.936267 -1.328992 -0.364120
H -1.359878 1.111236 1.362887
H -3.911506 0.157965 0.017258
H -2.720800 1.318590 -1.664420
H -3.925452 2.414203 -1.030513

H -2.093811 3.472742 0.405067
H -0.924716 2.475196 -0.429145
H -1.806875 3.448288 -2.633382
H -2.707958 4.607188 -1.676252
H 0.328117 4.241654 -1.695271
H -0.551426 5.531794 -2.490792
H -1.350447 6.342455 -0.251226
H -0.418358 5.080012 0.527428
H 0.856631 7.223459 0.546583
H 1.699942 6.020980 -0.433919
H 0.775188 7.304226 -1.215840
H -2.283571 -5.135066 1.869767
H -2.321020 -1.369892 1.992337
H -3.971727 1.117121 2.168167

2a-c43, ΔG = 0.4869 kcal/mol, population = 8.21 %
C 0.903959 -5.628029 -0.637439
C 2.020965 -4.704292 -1.118170
C 1.803511 -3.287899 -0.676388
C 0.576350 -2.767085 -0.380075
C -0.580534 -3.622194 -0.436086
C -0.440614 -5.027684 -1.005614
C 0.359059 -1.318656 0.026978
N 2.935821 -2.513035 -0.569042
C 2.871482 -1.246840 -0.069928
C 1.689956 -0.665475 0.250569
C 4.078682 -0.412329 0.225762
C 3.471791 0.942430 0.653340
C 1.952284 0.629627 0.819331
O 1.182658 1.400793 1.376441
O 4.009575 1.349772 1.902261
C 3.629873 1.980595 -0.428547
C 4.133844 3.193846 -0.248259
C -0.479444 -0.538897 -1.030666
C -1.621327 0.254702 -0.409590
C -2.328601 1.162099 -1.424522
C -3.250342 2.189673 -0.766896
C -2.511943 3.272642 0.020624
C -3.442430 4.353516 0.568649
C -2.707206 5.439166 1.352835
C -3.629144 6.527986 1.900162
C -2.881543 7.606494 2.682474
O -1.704096 -3.257582 -0.059165
O -1.506176 -5.847707 -0.564176

O -2.623167 -0.612456 0.151490
O -3.050735 0.351232 -2.361927
H 1.009445 -6.614292 -1.089240
H 0.954725 -5.745856 0.447515
H 2.071856 -4.715756 -2.212808
H 2.988989 -5.054061 -0.755589
H -0.488752 -4.911236 -2.100485
H -0.201667 -1.310336 0.966751
H 3.834932 -2.935104 -0.750130
H 4.652016 -0.835342 1.052645
H 4.740123 -0.333766 -0.638072
H 3.378425 1.972372 2.291942
H 3.276418 1.672054 -1.408462
H 4.209457 3.894659 -1.069729
H 4.497107 3.518514 0.718652
H 0.178508 0.147329 -1.567336
H -0.882835 -1.227694 -1.775459
H -1.209763 0.873705 0.391466
H -1.570037 1.691011 -2.007212
H -3.835437 2.658737 -1.563110
H -3.959374 1.668316 -0.116297
H -1.962268 2.824915 0.853722
H -1.759899 3.739614 -0.625714
H -3.990347 4.814231 -0.260987
H -4.196344 3.888252 1.213647
H -2.161458 4.977851 2.184005
H -1.949524 5.899764 0.708341
H -4.173117 6.989137 1.068989
H -4.386358 6.067167 2.543340
H -3.562745 8.370633 3.063183
H -2.352891 7.174927 3.536553
H -2.140020 8.104597 2.052154
H -2.217470 -5.238685 -0.305408
H -2.298775 -1.533044 0.134852
H -3.498371 -0.316766 -1.820700

2a-c81, ΔG = 0.5748 kcal/mol, population = 7.07 %
C -0.071514 -5.577266 -0.556689
C 0.986217 -4.727200 -1.257805
C 1.057676 -3.341967 -0.687929
C 0.017699 -2.734644 -0.044632
C -1.208575 -3.462404 0.154153
C -1.385701 -4.818385 -0.516674
C 0.083789 -1.317348 0.497706

N 2.261211 -2.691473 -0.840675
C 2.472586 -1.469240 -0.276005
C 1.491435 -0.813043 0.390008
C 3.794605 -0.765059 -0.289962
C 3.460602 0.611289 0.327725
C 2.032206 0.410549 0.919110
O 1.534530 1.202675 1.707924
O 4.369606 0.924746 1.370522
C 3.376439 1.683382 -0.730321
C 3.972763 2.865847 -0.661343
C -0.897749 -0.360311 -0.241944
C -1.728392 0.483041 0.716033
C -2.443621 1.637622 0.009004
C -2.985393 2.671998 0.992862
C -3.549884 3.931579 0.326275
C -2.577332 4.649447 -0.618679
C -1.232970 5.020156 0.007845
C -0.303192 5.757043 -0.955775
C 1.065573 6.061079 -0.348937
O -2.147045 -3.023303 0.834909
O -2.390727 -5.566574 0.141265
O -2.731985 -0.308363 1.375350
O -3.473955 1.126049 -0.847358
H -0.202515 -6.523924 -1.080777
H 0.242047 -5.797899 0.466355
H 0.754418 -4.644594 -2.325733
H 1.966670 -5.201027 -1.185675
H -1.700101 -4.600191 -1.550211
H -0.208892 -1.343810 1.552106
H 3.026027 -3.177521 -1.285683
H 4.519284 -1.283479 0.340420
H 4.209326 -0.696971 -1.296603
H 3.923037 1.557809 1.951877
H 2.748545 1.433152 -1.580871
H 3.854194 3.597808 -1.449828
H 4.604512 3.134464 0.175911
H -0.319984 0.301108 -0.890146
H -1.566116 -0.925497 -0.894260
H -1.058899 0.909950 1.469577
H -1.720655 2.117010 -0.652510
H -3.758663 2.202254 1.607295
H -2.174693 2.946747 1.673232
H -4.452130 3.667816 -0.231510
H -3.863677 4.625502 1.112665

H -2.397035 4.029010 -1.502613
H -3.060517 5.559399 -0.989236
H -1.402284 5.638570 0.897146
H -0.722828 4.118055 0.359194
H -0.174458 5.150753 -1.858918
H -0.779665 6.688879 -1.278203
H 0.968464 6.680254 0.546814
H 1.578562 5.139532 -0.061134
H 1.707816 6.593091 -1.054295
H -2.933062 -4.916104 0.617319
H -2.518740 -1.253089 1.258677
H -3.933516 0.459450 -0.314953

2a-c13, ΔG = 0.8214 kcal/mol, population = 4.66 %
C -1.700728 -3.472009 -0.887883
C -0.523219 -2.930498 -1.694405
C 0.343519 -2.018894 -0.877591
C -0.099665 -1.315424 0.203029
C -1.461112 -1.505246 0.653982
C -2.418932 -2.317667 -0.214626
C 0.779273 -0.334746 0.968862
N 1.659900 -1.935119 -1.281356
C 2.571017 -1.247747 -0.543001
C 2.203549 -0.503893 0.528905
C 4.044935 -1.296514 -0.796204
C 4.616266 -0.316516 0.251491
C 3.385392 0.020992 1.152659
O 3.501050 0.612007 2.219541
O 5.601726 -0.967041 1.043010
C 5.119515 0.948639 -0.392965
C 6.306952 1.495726 -0.170660
C 0.276059 1.127736 0.839229
C -0.064224 1.584413 -0.570641
C -0.642728 3.004250 -0.617252
C -1.944438 3.201994 0.152071
C -3.073490 2.237315 -0.209433
C -4.374775 2.574346 0.519381
C -5.563795 1.694576 0.124964
C -5.383532 0.210487 0.445026
C -6.628763 -0.620151 0.144108
O -1.892829 -1.022221 1.702968
O -3.509555 -2.775973 0.560921
O 1.062769 1.521005 -1.469974
O -0.873345 3.368290 -1.985358

H -2.388090 -4.012924 -1.538558
H -1.344789 -4.164650 -0.121561
H -0.890281 -2.363764 -2.557690
H 0.078189 -3.749975 -2.091303
H -2.779243 -1.625563 -0.993257
H 0.712578 -0.571674 2.034706
H 1.971610 -2.518060 -2.044183
H 4.439782 -2.298300 -0.618335
H 4.287795 -1.016708 -1.822547
H 5.664982 -0.474052 1.874088
H 4.420939 1.420663 -1.077653
H 6.600136 2.413850 -0.663355
H 7.025737 1.037539 0.496897
H -0.619547 1.222604 1.451111
H 1.028108 1.792627 1.273981
H -0.787170 0.902767 -1.018647
H 0.113172 3.681935 -0.193780
H -1.732178 3.138054 1.222398
H -2.271632 4.229859 -0.030874
H -3.242489 2.262198 -1.289716
H -2.777326 1.216230 0.037966
H -4.211151 2.495141 1.600516
H -4.627600 3.621775 0.324147
H -6.459465 2.060090 0.638640
H -5.758342 1.809135 -0.947941
H -4.542839 -0.189559 -0.125320
H -5.115161 0.101925 1.501580
H -7.483104 -0.275942 0.733062
H -6.903195 -0.544816 -0.911676
H -6.465473 -1.675312 0.371865
H -3.553812 -2.177130 1.325310
H 1.796178 2.007297 -1.071470
H -0.117074 3.023135 -2.481162

2a-c5, ΔG = 1.0254 kcal/mol, population = 3.30 %
C -2.286134 -2.983291 0.034605
C -1.333635 -2.428311 -1.021606
C -0.175199 -1.696605 -0.410264
C -0.205527 -1.122736 0.827073
C -1.387146 -1.287228 1.647407
C -2.642667 -1.885025 1.018831
C 0.963451 -0.335334 1.408526
N 0.964729 -1.638764 -1.183320
C 2.120970 -1.122547 -0.679080

C 2.169960 -0.540578 0.542559
C 3.434761 -1.189595 -1.392197
C 4.377570 -0.383417 -0.471172
C 3.524864 -0.145274 0.813390
O 4.008002 0.292159 1.849579
O 5.518705 -1.164702 -0.143007
C 4.723019 0.954163 -1.073295
C 5.956302 1.406214 -1.257490
C 0.610321 1.164007 1.564569
C 0.461234 1.921153 0.247720
C -0.534769 3.087032 0.337593
C -1.991751 2.660869 0.497205
C -2.544392 1.784194 -0.626645
C -4.023145 1.452450 -0.435207
C -4.589788 0.585742 -1.559892
C -6.059286 0.194574 -1.379043
C -6.310680 -0.741064 -0.195977
O -1.432791 -0.949202 2.831876
O -3.514019 -2.355810 2.028525
O 1.728113 2.423901 -0.229999
O -0.432981 3.890439 -0.843322
H -3.189152 -3.369231 -0.438856
H -1.810570 -3.805103 0.574908
H -1.865874 -1.731490 -1.679083
H -0.961143 -3.232092 -1.658740
H -3.125392 -1.063783 0.469733
H 1.165013 -0.710790 2.415506
H 0.973641 -2.109531 -2.076274
H 3.787534 -2.219437 -1.471466
H 3.367959 -0.778067 -2.400404
H 5.905987 -0.778022 0.655602
H 3.861642 1.561068 -1.335252
H 6.133676 2.383822 -1.687457
H 6.823814 0.813015 -0.996410
H -0.308810 1.229456 2.144651
H 1.384125 1.650004 2.165076
H 0.134541 1.249535 -0.546356
H -0.254576 3.696630 1.209411
H -2.109586 2.149316 1.455752
H -2.590701 3.573509 0.567655
H -2.400690 2.288176 -1.585310
H -1.980217 0.848957 -0.684671
H -4.153175 0.951981 0.529452
H -4.600871 2.381337 -0.373347

H -4.473816 1.122926 -2.506622
H -3.989096 -0.326668 -1.655907
H -6.661212 1.102297 -1.263355
H -6.409194 -0.289379 -2.295670
H -7.360748 -1.036543 -0.144835
H -5.711951 -1.651420 -0.284593
H -6.055602 -0.271342 0.755624
H -3.227707 -1.907428 2.842793
H 2.187071 2.848579 0.506563
H 0.507495 3.895401 -1.074851

2a-c78, ΔG = 1.0429 kcal/mol, population = 3.21 %
C 1.134759 -5.749010 -0.610650
C 1.968867 -4.667771 -1.294333
C 1.637690 -3.296660 -0.784695
C 0.452463 -2.962211 -0.206429
C -0.541800 -3.995471 0.009296
C -0.331683 -5.356605 -0.648099
C 0.114001 -1.539077 0.210382
N 2.645187 -2.353308 -0.915438
C 2.542476 -1.145982 -0.306302
C 1.383559 -0.738679 0.279963
C 3.692018 -0.203819 -0.134121
C 3.059495 0.995507 0.606216
C 1.630489 0.493708 0.962008
O 0.901033 1.114530 1.738351
O 3.779332 1.254605 1.802882
C 2.939541 2.203053 -0.289215
C 3.381499 3.416499 0.010969
C -0.910653 -0.937971 -0.779690
C -1.284883 0.514693 -0.495153
C -2.450426 0.988160 -1.384317
C -2.429156 2.488816 -1.682326
C -2.443639 3.419680 -0.471071
C -2.525060 4.892518 -0.875486
C -2.402134 5.872208 0.294395
C -3.534028 5.783027 1.319738
C -3.413956 6.832257 2.423651
O -1.555151 -3.820560 0.686263
O -1.144885 -6.330047 -0.021399
O -1.690680 0.688886 0.868122
O -3.695290 0.574860 -0.805927
H 1.278638 -6.707694 -1.109302
H 1.445515 -5.860582 0.430876

H 1.786874 -4.681873 -2.375037
H 3.033454 -4.863514 -1.154733
H -0.639264 -5.235961 -1.699055
H -0.357458 -1.564236 1.194860
H 3.538431 -2.650185 -1.281328
H 4.471762 -0.649458 0.485829
H 4.138227 0.069027 -1.091542
H 3.199023 1.772152 2.379673
H 2.435761 2.019450 -1.233906
H 3.254428 4.242561 -0.676782
H 3.892890 3.619448 0.943431
H -0.499758 -0.996563 -1.791571
H -1.814377 -1.549928 -0.752450
H -0.423697 1.157196 -0.699655
H -2.381901 0.461959 -2.339366
H -1.540141 2.695271 -2.289231
H -3.293772 2.708054 -2.317321
H -3.289322 3.165090 0.171872
H -1.543654 3.262913 0.129893
H -1.729034 5.104335 -1.597573
H -3.470009 5.072031 -1.401510
H -1.444579 5.707036 0.802246
H -2.366410 6.893560 -0.099967
H -4.493270 5.900457 0.803605
H -3.548336 4.786732 1.769960
H -3.434460 7.843240 2.007986
H -4.230270 6.750688 3.144676
H -2.473808 6.718539 2.970041
H -1.820109 -5.825942 0.464387
H -0.883970 0.852922 1.392332
H -3.592183 0.692448 0.149755

2a-c224, ΔG = 1.2086 kcal/mol, population = 2.42 %
C -1.168497 -4.567596 -0.439176
C 0.336524 -4.354122 -0.584220
C 0.727497 -2.922851 -0.361708
C -0.114611 -1.866365 -0.532241
C -1.503568 -2.124298 -0.854456
C -1.909111 -3.534405 -1.270580
C 0.320285 -0.418111 -0.359413
N 2.031374 -2.725521 0.057782
C 2.469660 -1.491036 0.406532
C 1.688969 -0.389237 0.253051
C 3.804006 -1.223161 1.027304

C 3.840891 0.318004 1.139392
C 2.402328 0.752460 0.732947
O 2.016554 1.917035 0.847685
O 4.081983 0.692277 2.489259
C 4.824757 0.920927 0.169669
C 5.796405 1.761201 0.497786
C 0.319969 0.372165 -1.702320
C -0.856375 1.323725 -1.943636
C -1.037116 2.362438 -0.829487
C -2.276657 3.221951 -1.034455
C -2.593318 4.150831 0.142251
C -2.756174 3.450296 1.496361
C -3.808041 2.341684 1.515283
C -3.987054 1.704183 2.892421
C -5.011913 0.571336 2.895585
O -2.372345 -1.251695 -0.810832
O -3.311088 -3.689413 -1.161715
O -0.661644 1.986291 -3.202858
O 0.114263 3.217560 -0.769623
H -1.440108 -5.572765 -0.762006
H -1.464404 -4.458039 0.606924
H 0.660507 -4.642643 -1.590712
H 0.880928 -4.992956 0.113757
H -1.603802 -3.640798 -2.323490
H -0.381895 0.059690 0.326373
H 2.616200 -3.531647 0.223117
H 3.867643 -1.666865 2.022383
H 4.619183 -1.627213 0.425752
H 3.828049 1.621897 2.579631
H 4.678579 0.626090 -0.865642
H 6.463604 2.163549 -0.253535
H 5.963630 2.063396 1.523957
H 1.236687 0.964495 -1.756022
H 0.367295 -0.333319 -2.533785
H -1.778122 0.751924 -2.037125
H -1.137733 1.830151 0.118256
H -2.145494 3.815398 -1.942341
H -3.121844 2.554857 -1.221929
H -1.802993 4.900639 0.226237
H -3.514365 4.695262 -0.090099
H -1.794212 3.036784 1.816477
H -3.019283 4.202993 2.246789
H -4.768345 2.746161 1.173937
H -3.538106 1.557479 0.801340

H -3.019835 1.322521 3.237065
H -4.286171 2.474058 3.611534
H -5.994970 0.931148 2.580501
H -4.715212 -0.225016 2.207719
H -5.120640 0.130986 3.889170
H -3.665950 -2.785239 -1.108760
H -0.004192 2.677626 -3.037384
H 0.783380 2.784972 -0.206656

2a-c92, ΔG = 1.2845 kcal/mol, population = 2.13 %
C 1.459587 -5.571671 -0.932689
C 2.226889 -4.336883 -1.400625
C 1.670269 -3.074564 -0.812301
C 0.393527 -2.940461 -0.361345
C -0.484248 -4.094807 -0.377080
C -0.029335 -5.344314 -1.125738
C -0.164831 -1.618620 0.141157
N 2.560550 -2.014883 -0.724580
C 2.239632 -0.901662 -0.020066
C 0.980244 -0.697149 0.454305
C 3.232937 0.144197 0.380380
C 2.366079 1.186338 1.121367
C 0.993669 0.472637 1.276990
O 0.118365 0.912086 2.026097
O 2.911883 1.452388 2.403974
C 2.155576 2.430206 0.293160
C 2.316162 3.670625 0.732690
C -1.125475 -1.019976 -0.915422
C -1.646533 0.367357 -0.558548
C -2.694818 0.887899 -1.565472
C -2.617106 2.402648 -1.771933
C -2.691702 3.256350 -0.507410
C -2.599346 4.752888 -0.807789
C -2.597818 5.640532 0.440134
C -1.350712 5.492185 1.314745
C -1.350523 6.440873 2.511944
O -1.585794 -4.107590 0.172391
O -0.784298 -6.464681 -0.705637
O -2.259012 0.375481 0.737313
O -4.004147 0.463526 -1.168920
H 1.780091 -6.449865 -1.493424
H 1.656056 -5.757051 0.126057
H 2.175399 -4.255200 -2.492349
H 3.283972 -4.425242 -1.143966

H -0.221184 -5.145018 -2.192136
H -0.745480 -1.801679 1.047300
H 3.519259 -2.166469 -1.003596
H 3.977676 -0.267111 1.063828
H 3.757544 0.555966 -0.482811
H 2.197737 1.815353 2.947825
H 1.823891 2.247547 -0.724990
H 2.128064 4.519277 0.087998
H 2.648534 3.878126 1.741996
H -0.598558 -0.953018 -1.871572
H -1.967974 -1.701893 -1.046489
H -0.809687 1.070203 -0.562642
H -2.510378 0.411460 -2.531060
H -1.674994 2.614660 -2.290909
H -3.420037 2.687742 -2.459181
H -3.625614 3.053202 0.025897
H -1.884957 2.971018 0.169524
H -1.687564 4.948001 -1.384622
H -3.436458 5.038710 -1.453253
H -2.684793 6.688076 0.132282
H -3.488587 5.423962 1.041576
H -1.265377 4.462773 1.672534
H -0.462379 5.675034 0.700636
H -1.397073 7.483813 2.187218
H -2.213896 6.256600 3.156862
H -0.450074 6.319529 3.118120
H -1.570240 -6.094782 -0.268063
H -1.556962 0.550288 1.392493
H -4.003479 0.490421 -0.200322

2a-c22, ΔG = 1.4458 kcal/mol, population = 1.62 %
C -0.016717 -5.501009 -0.475254
C 1.054677 -4.652790 -1.159247
C 1.021919 -3.229385 -0.689027
C -0.092779 -2.633565 -0.174038
C -1.301703 -3.399404 -0.020325
C -1.363912 -4.809984 -0.590218
C -0.137937 -1.177957 0.250083
N 2.202697 -2.527910 -0.793323
C 2.316672 -1.272092 -0.272967
C 1.253519 -0.626313 0.264790
C 3.611873 -0.524585 -0.176393
C 3.179815 0.849569 0.383984
C 1.708943 0.610632 0.840301

O 1.124149 1.378858 1.590995
O 3.971878 1.206069 1.505356
C 3.162361 1.908053 -0.690912
C 3.674328 3.124601 -0.564270
C -1.064714 -0.345218 -0.683575
C -1.999468 0.575666 0.084134
C -2.816353 1.484608 -0.847538
C -3.415630 2.697171 -0.131202
C -2.388664 3.673106 0.456862
C -1.385892 4.215926 -0.565195
C -0.461229 5.308860 -0.022842
C 0.467002 4.839322 1.098969
C 1.525914 5.876986 1.465271
O -2.313201 -2.947951 0.536921
O -2.379842 -5.557683 0.052186
O -2.930914 -0.170655 0.890808
O -3.842284 0.713917 -1.486766
H -0.063473 -6.488856 -0.933535
H 0.226152 -5.630310 0.582056
H 0.900568 -4.657386 -2.244223
H 2.045785 -5.073981 -0.982544
H -1.609518 -4.688815 -1.657782
H -0.551714 -1.130247 1.261268
H 3.025512 -3.006462 -1.130114
H 4.290520 -1.009771 0.527343
H 4.115984 -0.457749 -1.141594
H 3.438828 1.811302 2.042163
H 2.653916 1.618679 -1.606245
H 3.605397 3.846104 -1.368116
H 4.182831 3.435206 0.339592
H -0.446167 0.256099 -1.352467
H -1.656949 -1.007813 -1.318341
H -1.397618 1.196766 0.751232
H -2.165976 1.827590 -1.652621
H -4.043196 3.223785 -0.856250
H -4.079188 2.338971 0.661767
H -2.929984 4.514122 0.902251
H -1.852598 3.194258 1.279622
H -0.762496 3.399455 -0.943044
H -1.937352 4.604843 -1.428140
H 0.151314 5.687417 -0.848521
H -1.060011 6.157550 0.328253
H -0.121399 4.594325 1.987717
H 0.956459 3.910159 0.795234

H 2.169735 5.522160 2.273324
H 2.164064 6.102346 0.606471
H 1.066711 6.814285 1.791670
H -2.994645 -4.902866 0.422059
H -2.728021 -1.122054 0.817425
H -4.210260 0.158203 -0.782424

2a-c49, ΔG = 1.4508 kcal/mol, population = 1.61 %
C -1.487249 -3.953149 0.067996
C -0.207803 -3.875316 -0.764355
C 0.540262 -2.595546 -0.535633
C -0.030565 -1.447774 -0.083138
C -1.423801 -1.468900 0.319010
C -2.277362 -2.664113 -0.087256
C 0.724704 -0.131232 0.027852
N 1.901928 -2.643144 -0.789853
C 2.704419 -1.619520 -0.411453
C 2.196371 -0.434000 0.026950
C 4.196093 -1.716653 -0.350930
C 4.625633 -0.329428 0.176413
C 3.276288 0.381145 0.482322
O 3.224029 1.474184 1.050235
O 5.351452 -0.493581 1.388580
C 5.375624 0.461290 -0.863952
C 6.576615 0.996197 -0.692310
C 0.303491 0.805364 -1.147002
C 0.281548 2.314916 -0.897120
C -0.694481 2.784311 0.194933
C -2.151147 2.530477 -0.155408
C -3.124909 2.973708 0.936558
C -4.592014 2.682719 0.604619
C -4.947336 1.195643 0.571524
C -6.425365 0.930046 0.291204
C -6.767978 -0.558962 0.281138
O -1.947502 -0.559817 0.960539
O -3.471853 -2.689280 0.669819
O 1.577624 2.878324 -0.648631
O -0.508152 4.195349 0.388023
H -2.091496 -4.803293 -0.248990
H -1.239882 -4.090573 1.123336
H -0.450814 -3.938871 -1.831114
H 0.441373 -4.723679 -0.541167
H -2.515807 -2.519964 -1.152822
H 0.446376 0.340312 0.971705

H 2.313948 -3.526699 -1.053686
H 4.511353 -2.493272 0.347647
H 4.621967 -1.950459 -1.327687
H 5.371145 0.366353 1.832189
H 4.847587 0.587409 -1.804886
H 7.054235 1.560147 -1.483114
H 7.123834 0.879064 0.234625
H 0.960831 0.608767 -1.996972
H -0.698964 0.518949 -1.468392
H -0.038288 2.785731 -1.830952
H -0.457110 2.268370 1.134668
H -2.380475 3.052901 -1.090956
H -2.277741 1.464326 -0.332179
H -2.862294 2.468889 1.872976
H -2.999808 4.044110 1.113958
H -5.227309 3.177251 1.347101
H -4.841460 3.137348 -0.361434
H -4.344752 0.681241 -0.182280
H -4.674566 0.743743 1.531766
H -7.033737 1.440849 1.045367
H -6.695447 1.375122 -0.672445
H -7.828819 -0.726679 0.082447
H -6.195935 -1.087565 -0.485790
H -6.531085 -1.020090 1.243751
H -3.561752 -1.793908 1.040042
H 2.019062 2.416581 0.091480
H 0.443411 4.340768 0.274720

2a-c3, ΔG = 1.4696 kcal/mol, population = 1.56 %
C -1.896179 -3.333220 -0.703871
C -0.783733 -2.752924 -1.574463
C 0.160617 -1.897039 -0.782164
C -0.181918 -1.252229 0.370862
C -1.500910 -1.469167 0.924608
C -2.540984 -2.209856 0.086393
C 0.779923 -0.351136 1.139106
N 1.440248 -1.805100 -1.284497
C 2.421166 -1.175620 -0.578098
C 2.158853 -0.513621 0.573188
C 3.863149 -1.191156 -0.976769
C 4.523776 -0.252853 0.057222
C 3.391613 0.003499 1.099803
O 3.603295 0.547825 2.175838
O 5.608536 -0.916212 0.692479

C 4.912215 1.063149 -0.565397
C 6.118151 1.610407 -0.491684
C 0.309136 1.124652 1.136340
C 0.351294 1.812894 -0.223413
C -0.593153 3.018887 -0.325769
C -2.076691 2.689174 -0.197761
C -2.580462 1.605996 -1.151330
C -4.093400 1.382243 -1.084135
C -4.608994 0.978535 0.299057
C -6.052958 0.480209 0.283363
C -6.556435 0.075197 1.667368
O -1.832019 -1.073773 2.044568
O -3.584044 -2.682955 0.917070
O 1.684513 2.251827 -0.567120
O -0.386875 3.665215 -1.588856
H -2.641088 -3.830934 -1.324991
H -1.486182 -4.070971 -0.010161
H -1.216704 -2.135342 -2.369719
H -0.226485 -3.551393 -2.066960
H -2.944454 -1.472260 -0.623795
H 0.778100 -0.664434 2.187103
H 1.679673 -2.337451 -2.107976
H 4.285314 -2.193804 -0.888589
H 4.000612 -0.857149 -2.006331
H 5.778427 -0.448990 1.523254
H 4.102191 1.568516 -1.082330
H 6.326051 2.566629 -0.954722
H 6.935258 1.118443 0.021015
H -0.705221 1.143068 1.530646
H 0.924002 1.692922 1.840066
H 0.097092 1.107361 -1.015269
H -0.324770 3.718754 0.479137
H -2.280928 2.405866 0.835864
H -2.631328 3.616074 -0.372739
H -2.296845 1.868554 -2.173022
H -2.078380 0.660759 -0.929129
H -4.613034 2.288824 -1.413272
H -4.359642 0.599898 -1.802874
H -3.968477 0.194825 0.714357
H -4.531799 1.825094 0.988083
H -6.701063 1.259574 -0.131475
H -6.127102 -0.374717 -0.396982
H -5.942361 -0.726824 2.084996
H -6.516988 0.918623 2.361730

H -7.588757 -0.279495 1.631046
H -3.500249 -2.180269 1.745819
H 2.039761 2.753232 0.178958
H 0.568275 3.638458 -1.744585

2a-c4, ΔG = 1.4847 kcal/mol, population = 1.52 %
C -1.701025 -3.329336 -0.974675
C -0.485319 -2.795139 -1.728748
C 0.404074 -1.962357 -0.853078
C -0.018872 -1.328164 0.277721
C -1.388066 -1.504696 0.711189
C -2.372403 -2.185901 -0.235824
C 0.885303 -0.423609 1.104756
N 1.719709 -1.873246 -1.259747
C 2.648674 -1.259612 -0.478127
C 2.303197 -0.605193 0.656244
C 4.121432 -1.293782 -0.750217
C 4.687656 -0.335284 0.308429
C 3.500218 -0.134949 1.295682
O 3.651930 0.347992 2.411884
O 5.788831 -0.940306 0.987448
C 5.076627 1.033928 -0.197855
C 4.777432 1.575815 -1.372173
C 0.429855 1.057571 1.036391
C 0.213780 1.612307 -0.363937
C -0.473726 2.984372 -0.362486
C -1.878579 2.989017 0.234337
C -2.830307 1.935681 -0.331525
C -4.223958 1.994032 0.289674
C -5.147514 0.890166 -0.222145
C -6.538176 0.910851 0.410190
C -7.439145 -0.213319 -0.098037
O -1.805375 -1.106167 1.800692
O -3.512433 -2.621879 0.479450
O 1.429968 1.700576 -1.133608
O -0.557571 3.460125 -1.712850
H -2.403014 -3.790131 -1.669845
H -1.393206 -4.089314 -0.252544
H -0.811764 -2.170484 -2.568221
H 0.090743 -3.617016 -2.157236
H -2.668763 -1.419114 -0.969077
H 0.800787 -0.712631 2.155909
H 2.014594 -2.399735 -2.069004
H 4.521463 -2.294646 -0.579150

H 4.359296 -1.013920 -1.775780
H 5.847670 -0.518110 1.857472
H 5.626033 1.616095 0.536665
H 5.090275 2.584588 -1.610210
H 4.212641 1.054331 -2.134537
H -0.506039 1.137059 1.586942
H 1.160116 1.674479 1.568757
H -0.405376 0.927042 -0.942968
H 0.154062 3.675364 0.218271
H -1.798541 2.866547 1.317399
H -2.297312 3.986721 0.072969
H -2.903091 2.051478 -1.416174
H -2.423084 0.938227 -0.153729
H -4.134510 1.911964 1.378937
H -4.675104 2.972665 0.090841
H -5.243967 0.973650 -1.310838
H -4.684809 -0.084035 -0.030048
H -6.437418 0.835235 1.498131
H -7.011066 1.878795 0.212822
H -8.425297 -0.179949 0.370223
H -7.580744 -0.143245 -1.179846
H -7.000583 -1.191734 0.115191
H -3.495171 -2.126177 1.316298
H 2.128260 2.086264 -0.588071
H 0.285460 3.222153 -2.125376

2a-c86, ΔG = 1.5681 kcal/mol, population = 1.32 %
C 0.127753 -5.596264 -0.239323
C 1.145806 -4.737429 -0.987512
C 1.056999 -3.294716 -0.588762
C -0.071664 -2.721371 -0.078889
C -1.243450 -3.531713 0.134157
C -1.250189 -4.971535 -0.363825
C -0.169523 -1.248972 0.279222
N 2.203065 -2.549925 -0.757069
C 2.271241 -1.262179 -0.314810
C 1.193783 -0.631438 0.213174
C 3.533107 -0.455594 -0.303450
C 3.056116 0.931033 0.183318
C 1.606531 0.661983 0.689914
O 1.003514 1.458063 1.396251
O 3.858714 1.383134 1.262254
C 2.977251 1.920540 -0.952432
C 3.482252 3.146118 -0.924731

C -1.171221 -0.511013 -0.655373
C -2.104710 0.417927 0.102956
C -3.057849 1.180443 -0.818148
C -2.381595 2.123886 -1.813491
C -1.210488 2.976451 -1.312337
C -1.530278 3.982054 -0.206847
C -0.297687 4.778553 0.216144
C -0.562461 5.767047 1.350388
C 0.687429 6.537584 1.772333
O -2.267466 -3.100442 0.683617
O -2.221906 -5.729031 0.332884
O -2.904966 -0.310644 1.056846
O -3.985986 1.926410 -0.015386
H 0.117782 -6.607788 -0.645231
H 0.394976 -5.658867 0.818147
H 0.971640 -4.804356 -2.067452
H 2.158683 -5.103518 -0.810948
H -1.517606 -4.915455 -1.431602
H -0.551639 -1.171165 1.300289
H 3.038309 -3.008974 -1.090113
H 4.251742 -0.865897 0.408188
H 4.007220 -0.428414 -1.285664
H 3.321605 2.012830 1.765632
H 2.434707 1.569473 -1.825638
H 3.372472 3.814468 -1.769003
H 4.026488 3.515312 -0.064758
H -0.610395 0.052387 -1.400900
H -1.770510 -1.239616 -1.207147
H -1.516156 1.134303 0.679788
H -3.622701 0.431609 -1.391346
H -2.030235 1.517646 -2.652697
H -3.163489 2.772910 -2.218858
H -0.398002 2.329918 -0.970761
H -0.807774 3.520616 -2.173263
H -2.315407 4.665736 -0.549267
H -1.941666 3.460096 0.659993
H 0.485138 4.078401 0.525475
H 0.100396 5.320984 -0.649870
H -1.342136 6.472590 1.043211
H -0.964423 5.223372 2.212104
H 0.474236 7.236130 2.584524
H 1.469511 5.854895 2.115306
H 1.094380 7.112124 0.935854
H -2.858623 -5.082896 0.680530

H -2.788051 -1.266790 0.909606
H -4.266966 1.317474 0.682593

2a-c8, ΔG = 1.8267 kcal/mol, population = 0.85 %
C -2.290680 -3.179831 0.115390
C -1.352241 -2.622919 -0.950931
C -0.201097 -1.864819 -0.358204
C -0.213163 -1.304109 0.885353
C -1.378282 -1.491984 1.723815
C -2.640065 -2.074790 1.095211
C 0.938377 -0.463808 1.428580
N 0.910680 -1.758493 -1.166437
C 2.057974 -1.189821 -0.701511
C 2.120770 -0.606294 0.518354
C 3.345532 -1.185656 -1.463979
C 4.253389 -0.281883 -0.600889
C 3.459497 -0.131936 0.732323
O 3.971581 0.315870 1.750889
O 5.497224 -0.918444 -0.349633
C 4.396782 1.086507 -1.218157
C 5.551994 1.697395 -1.445099
C 0.516707 1.018219 1.602209
C 0.025249 1.682827 0.315609
C -0.850261 2.912746 0.588417
C -1.554255 3.445107 -0.654900
C -2.421298 2.416843 -1.388784
C -3.512184 1.772475 -0.532307
C -4.305885 0.705021 -1.282057
C -5.442716 0.099927 -0.458766
C -6.133128 -1.065963 -1.162938
O -1.410250 -1.177721 2.915344
O -3.525126 -2.525760 2.101569
O 1.104727 2.041538 -0.557205
O -0.025664 3.986958 1.088625
H -3.197259 -3.570464 -0.347203
H -1.807642 -3.997869 0.654973
H -1.899995 -1.938530 -1.608859
H -0.972405 -3.426420 -1.584403
H -3.105009 -1.248773 0.537701
H 1.199341 -0.828622 2.425991
H 0.910640 -2.227362 -2.060244
H 3.771866 -2.188892 -1.521816
H 3.213862 -0.814946 -2.481759
H 5.864592 -0.508196 0.446976

H 3.453166 1.568680 -1.454759
H 5.583484 2.686982 -1.883078
H 6.498691 1.226029 -1.212229
H -0.280550 1.047082 2.346668
H 1.362790 1.577167 2.008603
H -0.572081 0.958257 -0.237457
H -1.590975 2.646735 1.346523
H -2.169297 4.295078 -0.347058
H -0.794243 3.831966 -1.337817
H -2.889553 2.912359 -2.244200
H -1.785280 1.633816 -1.812391
H -3.069416 1.313386 0.356874
H -4.191771 2.549597 -0.165437
H -4.715035 1.129892 -2.205302
H -3.620324 -0.091697 -1.594398
H -5.053510 -0.238534 0.507137
H -6.174786 0.881409 -0.231039
H -6.550342 -0.752888 -2.123621
H -5.426206 -1.876880 -1.357863
H -6.948402 -1.471773 -0.560463
H -3.215022 -2.103770 2.921596
H 1.462884 2.870301 -0.207204
H 0.182665 3.812906 2.014155

2a-c20, ΔG = 1.8474 kcal/mol, population = 0.82 %
C -1.868205 -3.239726 -0.740935
C -0.736554 -2.668117 -1.591040
C 0.251220 -1.892579 -0.770703
C -0.043161 -1.311462 0.427844
C -1.362706 -1.499484 0.990929
C -2.447137 -2.138176 0.126993
C 0.955081 -0.462266 1.207271
N 1.517015 -1.792444 -1.309101
C 2.526506 -1.201884 -0.610517
C 2.312426 -0.607103 0.587615
C 3.951586 -1.188633 -1.067315
C 4.641008 -0.275703 -0.028534
C 3.567785 -0.119285 1.091814
O 3.831675 0.341540 2.194575
O 5.796625 -0.913394 0.493643
C 4.919094 1.091318 -0.601947
C 6.108494 1.678105 -0.614191
C 0.522157 1.023179 1.244173
C 0.495012 1.691979 -0.133179

C -0.745206 2.567330 -0.358134
C -2.010944 1.733243 -0.475127
C -3.287797 2.550344 -0.664633
C -4.546729 1.681629 -0.763244
C -4.913828 0.962942 0.537041
C -6.120373 0.026925 0.417567
C -7.434868 0.736642 0.092100
O -1.662742 -1.142413 2.132334
O -3.496989 -2.621658 0.942458
O 1.686119 2.465473 -0.387330
O -0.580464 3.318261 -1.565340
H -2.644687 -3.660404 -1.380068
H -1.490444 -4.038409 -0.098376
H -1.144847 -1.992518 -2.351547
H -0.219331 -3.465536 -2.127187
H -2.824572 -1.335872 -0.526545
H 0.978297 -0.810075 2.243406
H 1.719117 -2.272110 -2.174005
H 4.386493 -2.188819 -1.023234
H 4.045979 -0.822931 -2.090729
H 6.002604 -0.478846 1.334042
H 4.042589 1.598248 -0.994856
H 6.237738 2.668319 -1.032373
H 6.988410 1.186669 -0.218139
H -0.455099 1.082247 1.720294
H 1.212004 1.568447 1.892499
H 0.502393 0.938997 -0.923633
H -0.840356 3.261721 0.490599
H -1.889642 1.042237 -1.316503
H -2.104321 1.125960 0.424891
H -3.395788 3.251473 0.170804
H -3.192964 3.155175 -1.568766
H -5.382416 2.312480 -1.076204
H -4.409881 0.939055 -1.558802
H -4.060992 0.377934 0.889495
H -5.110856 1.711263 1.313840
H -5.910416 -0.726900 -0.348465
H -6.232734 -0.520091 1.358178
H -7.406138 1.214378 -0.888937
H -8.270948 0.033878 0.090518
H -7.651998 1.511990 0.832213
H -3.411273 -2.143914 1.784973
H 1.871849 3.008494 0.389662
H 0.355486 3.566603 -1.590188

2a-c6, ΔG = 1.9120 kcal/mol, population = 0.74 %

C -1.823359 -3.432168 -0.669952
C -0.738946 -2.821632 -1.555066
C 0.202840 -1.953891 -0.772975
C -0.129283 -1.328640 0.393779
C -1.431939 -1.578898 0.971577
C -2.474179 -2.330605 0.145657
C 0.829021 -0.415905 1.152737
N 1.468823 -1.827990 -1.301717
C 2.449377 -1.179432 -0.612296
C 2.197467 -0.535609 0.551556
C 3.880860 -1.150480 -1.046469
C 4.539050 -0.199278 -0.022646
C 3.427950 0.012426 1.051955
O 3.650939 0.552827 2.127694
O 5.662078 -0.831356 0.576552
C 4.866729 1.133497 -0.644911
C 6.058128 1.715145 -0.607222
C 0.323730 1.047668 1.185885
C 0.307505 1.757010 -0.163876
C -0.665629 2.943191 -0.215834
C -2.136351 2.578119 -0.044050
C -2.644074 1.487705 -0.987600
C -4.150208 1.235125 -0.879896
C -4.615936 0.816394 0.518017
C -6.040214 0.256611 0.556272
C -7.114394 1.263725 0.144669
O -1.747894 -1.202623 2.102489
O -3.489827 -2.835369 0.991532
O 1.618931 2.229351 -0.544827
O -0.515233 3.612712 -1.474755
H -2.570490 -3.937243 -1.282371
H -1.386389 -4.169585 0.007435
H -1.198120 -2.206279 -2.337073
H -0.175144 -3.604601 -2.064663
H -2.907374 -1.593584 -0.547650
H 0.860140 -0.745342 2.195381
H 1.702381 -2.344966 -2.136658
H 4.334377 -2.140772 -0.975321
H 3.982847 -0.806385 -2.076792
H 5.846363 -0.358031 1.400762
H 4.024583 1.618655 -1.128915
H 6.222381 2.681135 -1.067539

H 6.906224 1.242859 -0.127282
H -0.678404 1.034939 1.610317
H 0.945840 1.620191 1.879630
H 0.041633 1.058568 -0.958089
H -0.385622 3.636672 0.590631
H -2.301935 2.286040 0.993880
H -2.717461 3.492611 -0.197164
H -2.395425 1.760363 -2.015878
H -2.115097 0.553311 -0.783715
H -4.687494 2.134189 -1.196113
H -4.423464 0.449439 -1.592968
H -3.930966 0.061593 0.913621
H -4.551369 1.669475 1.200726
H -6.095010 -0.623204 -0.093989
H -6.251280 -0.098646 1.569400
H -8.114712 0.837876 0.248522
H -7.066338 2.160335 0.769012
H -6.996456 1.577718 -0.894283
H -3.403560 -2.336674 1.822378
H 1.984793 2.735297 0.192995
H 0.434367 3.606363 -1.663490

2a-c28, ΔG = 2.0218 kcal/mol, population = 0.61 %
C -1.768631 -3.478616 -0.805934
C -0.646200 -2.879279 -1.649715
C 0.276462 -2.020776 -0.834977
C -0.079117 -1.412914 0.333988
C -1.406496 -1.647083 0.862375
C -2.429971 -2.367823 -0.012169
C 0.871363 -0.529386 1.138562
N 1.550485 -1.883687 -1.341399
C 2.512499 -1.223410 -0.639041
C 2.242297 -0.603624 0.534084
C 3.944357 -1.154935 -1.068175
C 4.570153 -0.187509 -0.039480
C 3.460798 -0.039244 1.046700
O 3.676537 0.473991 2.137245
O 5.729997 -0.768495 0.538611
C 4.822821 1.167815 -0.649789
C 5.986462 1.803851 -0.626784
C 0.357698 0.926629 1.244074
C 0.272901 1.678074 -0.079529
C -0.759369 2.814948 -0.058995
C -2.208151 2.368413 0.113474

C -2.730161 1.406368 -0.955884
C -4.118218 0.844999 -0.636172
C -5.230361 1.894051 -0.591412
C -6.632667 1.300211 -0.425985
C -6.843536 0.566727 0.899304
O -1.758781 -1.277880 1.984821
O -3.494054 -2.850649 0.785547
O 1.549452 2.225270 -0.478166
O -0.663399 3.560794 -1.278949
H -2.501064 -3.970300 -1.446392
H -1.366444 -4.224838 -0.116630
H -1.069461 -2.260117 -2.448853
H -0.070504 -3.667513 -2.137879
H -2.813349 -1.617471 -0.718558
H 0.905601 -0.909356 2.164368
H 1.797604 -2.381148 -2.184065
H 4.423709 -2.132891 -0.994092
H 4.041898 -0.808179 -2.097960
H 5.890605 -0.304738 1.373353
H 3.949849 1.620110 -1.110569
H 6.096635 2.781924 -1.077651
H 6.864280 1.365606 -0.168614
H -0.622255 0.890885 1.714152
H 1.007719 1.479619 1.927404
H 0.021079 0.993879 -0.890176
H -0.502510 3.474510 0.783388
H -2.319021 1.911002 1.099879
H -2.822036 3.271456 0.131231
H -2.749547 1.912712 -1.924153
H -2.037779 0.568750 -1.066553
H -4.378762 0.095209 -1.391320
H -4.072948 0.313972 0.319493
H -5.046499 2.597338 0.226833
H -5.197007 2.481003 -1.515720
H -7.367809 2.106395 -0.508605
H -6.833197 0.615936 -1.257642
H -6.620916 1.222217 1.745976
H -7.877055 0.228938 1.001742
H -6.201109 -0.311935 0.983544
H -3.431692 -2.355666 1.620859
H 1.920282 2.709395 0.271634
H 0.282123 3.613573 -1.480658

2a-c21, ΔG = 2.1680 kcal/mol, population = 0.48 %

C -2.298452 -3.179054 -0.127673
C -1.326881 -2.596087 -1.149525
C -0.192044 -1.859136 -0.501334
C -0.241487 -1.328560 0.754105
C -1.431460 -1.537003 1.552518
C -2.674779 -2.100899 0.871931
C 0.895630 -0.508215 1.355520
N 0.947255 -1.741756 -1.269741
C 2.081455 -1.193281 -0.753861
C 2.106002 -0.633369 0.479147
C 3.396648 -1.192686 -1.467925
C 4.282985 -0.317033 -0.555545
C 3.441984 -0.182015 0.750861
O 3.923533 0.237788 1.796031
O 5.508661 -0.972460 -0.267717
C 4.467981 1.059305 -1.143527
C 5.635341 1.672331 -1.286300
C 0.471407 0.967867 1.567636
C -0.000468 1.678502 0.301771
C -0.895091 2.887454 0.604662
C -1.584431 3.457642 -0.634119
C -2.431272 2.454459 -1.425771
C -3.533583 1.771887 -0.614980
C -4.310107 0.733475 -1.421575
C -5.459316 0.092968 -0.643315
C -6.132428 -1.046684 -1.404848
O -1.497295 -1.257045 2.750991
O -3.588526 -2.578750 1.839488
O 1.090948 2.057578 -0.551888
O -0.142444 3.910116 1.282087
H -3.191743 -3.553574 -0.627928
H -1.833916 -4.013449 0.403024
H -1.852564 -1.892064 -1.804750
H -0.930527 -3.383459 -1.793075
H -3.123513 -1.259474 0.324567
H 1.125270 -0.904807 2.348684
H 0.974509 -2.191257 -2.173040
H 3.811034 -2.200831 -1.526605
H 3.308461 -0.803688 -2.483534
H 5.840701 -0.592827 0.559119
H 3.544567 1.546971 -1.441336
H 5.695454 2.667510 -1.708123
H 6.564607 1.196435 -0.999110
H -0.337103 0.976237 2.298962

H 1.303357 1.518784 2.012051
H -0.586349 0.975815 -0.290698
H -1.646362 2.568492 1.328358
H -2.211629 4.292626 -0.309029
H -0.815387 3.872053 -1.293895
H -2.886781 2.980693 -2.269629
H -1.782718 1.692018 -1.867213
H -3.103896 1.278278 0.261952
H -4.222497 2.530877 -0.228254
H -4.704315 1.194254 -2.333981
H -3.616459 -0.048177 -1.753581
H -5.086258 -0.282019 0.315358
H -6.198247 0.862757 -0.398661
H -6.532919 -0.698086 -2.360403
H -5.419270 -1.847612 -1.617503
H -6.957291 -1.477660 -0.833710
H -3.302545 -2.179734 2.679524
H 1.690171 2.604457 -0.028197
H 0.289246 4.455014 0.612724

2a-c14, ΔG = 2.2239 kcal/mol, population = 0.44 %
C -2.002669 -3.327431 -0.279213
C -0.947679 -2.776543 -1.235265
C 0.101610 -1.973975 -0.523917
C -0.086720 -1.375317 0.686419
C -1.350266 -1.558446 1.369131
C -2.505325 -2.206139 0.610587
C 0.964543 -0.491824 1.347595
N 1.310884 -1.869751 -1.181359
C 2.370922 -1.256172 -0.592802
C 2.259745 -0.635342 0.606827
C 3.753973 -1.245611 -1.169352
C 4.497300 -0.267774 -0.246687
C 3.555950 -0.145914 0.987789
O 3.939738 0.306099 2.060154
O 5.759241 -0.803238 0.146710
C 4.670640 1.129216 -0.798760
C 4.063464 1.658543 -1.854296
C 0.505055 0.988352 1.430274
C -0.089136 1.550268 0.146128
C -0.746202 2.918511 0.361295
C -1.556703 3.395535 -0.845246
C -2.605809 2.403297 -1.360775
C -3.592560 1.904983 -0.304557

C -4.598881 0.898048 -0.858002
C -5.510741 0.301269 0.212704
C -6.472823 -0.748459 -0.339193
O -1.547459 -1.188485 2.528178
O -3.489353 -2.663112 1.518162
O 0.852281 1.606855 -0.937807
O 0.230005 3.904633 0.732812
H -2.830878 -3.760235 -0.840687
H -1.571633 -4.113000 0.345777
H -1.421284 -2.125014 -1.978771
H -0.472789 -3.587888 -1.789634
H -2.928900 -1.414793 -0.026564
H 1.101683 -0.823621 2.380594
H 1.436567 -2.362603 -2.053215
H 4.212026 -2.232888 -1.089164
H 3.763123 -0.959472 -2.220452
H 5.974002 -0.409726 1.005886
H 5.343839 1.739967 -0.203951
H 4.245341 2.687126 -2.138699
H 3.367908 1.103416 -2.470945
H -0.255502 1.062993 2.208178
H 1.348222 1.603833 1.752886
H -0.863388 0.865528 -0.198252
H -1.400236 2.836655 1.230692
H -2.040695 4.335268 -0.564888
H -0.859464 3.625931 -1.656670
H -3.162896 2.882371 -2.171504
H -2.104545 1.542387 -1.813410
H -3.051293 1.432940 0.520855
H -4.124605 2.757329 0.132422
H -5.207938 1.375135 -1.633955
H -4.054879 0.087382 -1.357698
H -4.894978 -0.149947 0.997293
H -6.077254 1.106283 0.692352
H -7.116325 -0.323710 -1.114232
H -5.925116 -1.583557 -0.783839
H -7.116229 -1.153106 0.444957
H -3.315496 -2.184850 2.347022
H 1.713144 1.879858 -0.596927
H 0.750490 4.107763 -0.054947

2a-c71, ΔG = 2.2515 kcal/mol, population = 0.42 %
C -0.033483 -5.535340 -0.089238
C 0.915672 -4.712869 -0.958840

C 0.918980 -3.265599 -0.566561
C -0.122603 -2.655114 0.071095
C -1.286123 -3.426731 0.425173
C -1.396185 -4.867714 -0.056480
C -0.132316 -1.177510 0.422514
N 2.057288 -2.557763 -0.882048
C 2.217253 -1.269769 -0.462662
C 1.231839 -0.607282 0.189904
C 3.492438 -0.497113 -0.612274
C 3.108261 0.907657 -0.094184
C 1.738247 0.672307 0.608880
O 1.258166 1.476866 1.394352
O 4.056593 1.373946 0.850974
C 2.879959 1.872256 -1.232027
C 3.376318 3.099890 -1.295183
C -1.209083 -0.414506 -0.402788
C -2.057559 0.527323 0.437820
C -3.145712 1.219521 -0.387215
C -2.632225 2.115455 -1.513015
C -1.497502 3.095206 -1.188368
C -1.872488 4.253043 -0.261025
C -0.756715 5.292224 -0.110040
C 0.471345 4.783278 0.645242
C 1.589225 5.819494 0.735083
O -2.226627 -2.960625 1.084851
O -2.302262 -5.590805 0.755859
O -2.702383 -0.166730 1.523638
O -3.982361 1.986685 0.490756
H -0.122445 -6.547905 -0.482709
H 0.353951 -5.601825 0.930104
H 0.614572 -4.780879 -2.010373
H 1.930423 -5.109768 -0.898321
H -1.785678 -4.807754 -1.085824
H -0.390969 -1.078026 1.479440
H 2.831406 -3.045072 -1.309564
H 4.278908 -0.917015 0.017386
H 3.848804 -0.494477 -1.643290
H 3.593435 2.005081 1.421890
H 2.229053 1.501618 -2.018822
H 3.152198 3.750666 -2.130449
H 4.022509 3.491246 -0.519917
H -0.714318 0.148381 -1.194275
H -1.867137 -1.131391 -0.900759
H -1.420854 1.286735 0.897177

H -3.756658 0.426144 -0.841289
H -2.303585 1.459871 -2.323455
H -3.491806 2.667963 -1.904359
H -0.644563 2.550718 -0.777444
H -1.147719 3.517244 -2.136907
H -2.767316 4.741913 -0.660334
H -2.153639 3.866041 0.721362
H -0.450287 5.637092 -1.105513
H -1.151431 6.172370 0.409179
H 0.167161 4.480708 1.652896
H 0.858994 3.882671 0.167537
H 2.446873 5.433253 1.290557
H 1.940864 6.101171 -0.261274
H 1.248309 6.730063 1.235745
H -2.875384 -4.923479 1.168106
H -2.646897 -1.127482 1.366149
H -4.148730 1.410641 1.250731

2a-c11, ΔG = 2.2634 kcal/mol, population = 0.41 %
C -1.793232 -3.490031 -1.138618
C -0.589982 -2.888136 -1.860196
C 0.253082 -2.043569 -0.950827
C -0.209426 -1.457136 0.190003
C -1.574595 -1.706302 0.601502
C -2.519785 -2.394604 -0.379075
C 0.644368 -0.544519 1.062295
N 1.568247 -1.887965 -1.337686
C 2.455228 -1.229413 -0.541096
C 2.070501 -0.621466 0.607009
C 3.922062 -1.148753 -0.827343
C 4.437368 -0.181925 0.262318
C 3.229007 -0.051094 1.239036
O 3.333421 0.455933 2.348205
O 5.544695 -0.755382 0.941081
C 4.727786 1.182065 -0.311026
C 5.881523 1.824642 -0.187682
C 0.120626 0.910538 1.017819
C 0.279926 1.589968 -0.344393
C -0.951172 2.420260 -0.734621
C -2.113819 1.526805 -1.143505
C -3.433046 2.261484 -1.390079
C -4.049056 2.907725 -0.142512
C -4.280965 1.955344 1.036683
C -5.149187 0.739916 0.710504

C -5.437113 -0.121635 1.938579
O -2.017546 -1.365226 1.699923
O -3.652727 -2.893882 0.305112
O 1.464129 2.411443 -0.405178
O -0.632314 3.270355 -1.840742
H -2.466811 -3.959085 -1.855946
H -1.465266 -4.256618 -0.432543
H -0.929743 -2.254750 -2.687782
H 0.025244 -3.674403 -2.301004
H -2.833104 -1.618603 -1.094992
H 0.565520 -0.876825 2.100774
H 1.896544 -2.374532 -2.158835
H 4.398402 -2.123915 -0.710242
H 4.118560 -0.796292 -1.840767
H 5.629605 -0.289030 1.785355
H 3.888323 1.637782 -0.827756
H 6.019771 2.809673 -0.615152
H 6.723859 1.386125 0.332788
H -0.925064 0.902865 1.321985
H 0.650591 1.497815 1.771713
H 0.437054 0.848288 -1.130194
H -1.231138 3.042184 0.127208
H -1.813928 0.994114 -2.050644
H -2.263429 0.767959 -0.375422
H -3.279269 3.030569 -2.150841
H -4.144651 1.549443 -1.815704
H -3.416954 3.735309 0.192461
H -5.005810 3.357039 -0.427971
H -3.321713 1.608562 1.434341
H -4.752035 2.518005 1.849299
H -6.091633 1.078745 0.266627
H -4.658901 0.126339 -0.050220
H -6.044843 -0.992470 1.682871
H -4.506352 -0.479404 2.385116
H -5.974422 0.449032 2.700655
H -3.668260 -2.423079 1.156056
H 1.536817 2.910089 0.418657
H 0.282843 3.552707 -1.694451

2a-c56, ΔG = 2.2753 kcal/mol, population = 0.40 %
C -1.444386 -4.131917 -0.158949
C -0.137604 -3.989989 -0.936239
C 0.539129 -2.680284 -0.659751
C -0.106297 -1.560803 -0.234804

C -1.522047 -1.639019 0.066709
C -2.290430 -2.886670 -0.356785
C 0.588243 -0.216702 -0.059563
N 1.912899 -2.669843 -0.833496
C 2.649723 -1.621251 -0.394711
C 2.069794 -0.462112 0.025699
C 4.136629 -1.667332 -0.240363
C 4.480558 -0.275195 0.332193
C 3.089235 0.377995 0.568968
O 2.963676 1.455541 1.155142
O 5.143258 -0.431880 1.580505
C 5.254908 0.563205 -0.651815
C 6.421316 1.142861 -0.403839
C 0.192139 0.719271 -1.240997
C 0.200029 2.232236 -1.009718
C -0.767772 2.740758 0.073028
C -2.231088 2.542136 -0.290333
C -3.192023 3.068780 0.776861
C -4.656863 3.092104 0.326626
C -5.265657 1.731690 -0.027498
C -5.325788 0.746720 1.139763
C -6.067357 -0.540175 0.781943
O -2.134493 -0.737337 0.638524
O -3.509296 -2.972297 0.356000
O 1.507686 2.771568 -0.766588
O -0.533910 4.146267 0.256797
H -1.990307 -5.013036 -0.496317
H -1.235377 -4.252059 0.906689
H -0.332018 -4.052154 -2.012959
H 0.540704 -4.809784 -0.693604
H -2.497089 -2.764519 -1.431871
H 0.235885 0.227320 0.873108
H 2.374020 -3.533720 -1.080761
H 4.433689 -2.443245 0.466922
H 4.631394 -1.871172 -1.191077
H 5.104871 0.420852 2.036735
H 4.776046 0.684620 -1.619320
H 6.918924 1.739310 -1.157617
H 6.920285 1.031734 0.550672
H 0.847600 0.500773 -2.087166
H -0.816135 0.451053 -1.559700
H -0.106503 2.696054 -1.951310
H -0.554483 2.224568 1.018820
H -2.419046 3.054585 -1.240689

H -2.409182 1.478585 -0.447339
H -3.085806 2.464675 1.683282
H -2.897979 4.084721 1.047733
H -5.260180 3.549280 1.118430
H -4.741919 3.753866 -0.542159
H -6.281296 1.894272 -0.404494
H -4.708281 1.275399 -0.852323
H -4.312534 0.500507 1.463579
H -5.819477 1.228471 1.990991
H -6.073006 -1.247094 1.615038
H -7.106980 -0.333822 0.514173
H -5.602835 -1.035940 -0.074607
H -3.666478 -2.080288 0.709777
H 1.897955 2.362231 0.030509
H 0.417890 4.264254 0.116706

2a-c2, ΔG = 2.2785 kcal/mol, population = 0.40 %
C -2.067137 -3.244904 -0.377010
C -1.016632 -2.708456 -1.345200
C 0.023919 -1.883713 -0.646245
C -0.187663 -1.225472 0.530925
C -1.457943 -1.396471 1.202932
C -2.594940 -2.096753 0.461708
C 0.873158 -0.357116 1.201794
N 1.252914 -1.837529 -1.267582
C 2.314565 -1.233805 -0.665565
C 2.183013 -0.551425 0.496571
C 3.711730 -1.302855 -1.196751
C 4.484536 -0.345718 -0.263620
C 3.476710 -0.070590 0.894863
O 3.816761 0.461809 1.944171
O 5.642319 -0.982932 0.255327
C 4.783414 0.959259 -0.956830
C 5.976121 1.537061 -1.005865
C 0.446092 1.130976 1.277682
C 0.198076 1.809651 -0.062832
C -0.579230 3.127388 0.052806
C -1.979758 3.007349 0.643529
C -2.853277 1.915778 0.028597
C -4.308467 1.998468 0.493574
C -5.136171 0.750279 0.177096
C -5.198135 0.382813 -1.306132
C -6.047996 -0.859366 -1.567748
O -1.673271 -0.991051 2.347562

O -3.579590 -2.527175 1.380643
O 1.424594 2.072742 -0.780317
O -0.699090 3.707327 -1.254070
H -2.883684 -3.713423 -0.926719
H -1.625533 -3.997504 0.280357
H -1.494349 -2.078813 -2.104435
H -0.533652 -3.529093 -1.877914
H -3.026567 -1.343961 -0.214583
H 0.975731 -0.684494 2.240864
H 1.395835 -2.383573 -2.104502
H 4.115532 -2.312846 -1.105264
H 3.762406 -1.012504 -2.247265
H 5.877697 -0.516858 1.070916
H 3.921310 1.426681 -1.423274
H 6.118469 2.481380 -1.515601
H 6.846614 1.080436 -0.551763
H -0.460931 1.175392 1.877964
H 1.211385 1.686622 1.826968
H -0.356281 1.143035 -0.724983
H 0.008457 3.805820 0.688604
H -1.898066 2.848078 1.721497
H -2.463165 3.980611 0.516754
H -2.803451 1.981487 -1.059877
H -2.452862 0.935857 0.298323
H -4.327127 2.162627 1.576205
H -4.779033 2.879033 0.042283
H -4.732414 -0.098526 0.739243
H -6.156885 0.896447 0.546235
H -5.597263 1.231913 -1.871498
H -4.187916 0.212272 -1.689257
H -7.076759 -0.709956 -1.229697
H -6.077959 -1.107832 -2.630816
H -5.647316 -1.724688 -1.033276
H -3.408449 -2.022144 2.194446
H 2.011048 2.572854 -0.197051
H 0.153356 3.558649 -1.688006

2a-c30, ΔG = 2.3682 kcal/mol, population = 0.34 %
C -1.596618 -4.099935 0.123987
C -0.489891 -3.844515 -0.897069
C 0.253794 -2.574250 -0.610376
C -0.268191 -1.519371 0.072768
C -1.582802 -1.654146 0.668638
C -2.444570 -2.849694 0.276726

C 0.471170 -0.197461 0.242795
N 1.561930 -2.533183 -1.062375
C 2.394771 -1.543865 -0.657763
C 1.935444 -0.445811 0.004948
C 3.881157 -1.604464 -0.811854
C 4.359981 -0.281518 -0.176137
C 3.059811 0.333641 0.415925
O 3.077733 1.341384 1.125929
O 5.266394 -0.567110 0.881520
C 4.925028 0.664136 -1.204890
C 6.125889 1.222735 -1.142343
C -0.133096 0.846155 -0.741477
C 0.021183 2.336719 -0.428102
C -0.604394 2.793359 0.897052
C -2.095129 2.511465 1.032217
C -2.968999 3.070577 -0.091673
C -4.467529 2.871104 0.156749
C -4.928571 1.411239 0.236473
C -4.642603 0.590929 -1.022874
C -5.331104 -0.772371 -1.008637
O -2.040368 -0.842015 1.473113
O -3.474000 -3.036983 1.229015
O 1.373877 2.813610 -0.490886
O -0.392189 4.207625 1.038607
H -2.218193 -4.937017 -0.194380
H -1.160896 -4.353033 1.093369
H -0.918286 -3.767576 -1.902827
H 0.209126 -4.682271 -0.918265
H -2.885361 -2.597065 -0.700367
H 0.316432 0.150550 1.265424
H 1.946362 -3.357032 -1.502290
H 4.297271 -2.451142 -0.263611
H 4.172688 -1.705261 -1.858263
H 5.336382 0.231702 1.423600
H 4.258109 0.885265 -2.033394
H 6.466980 1.901030 -1.913735
H 6.808270 1.011409 -0.328734
H 0.283291 0.662285 -1.734614
H -1.203899 0.649755 -0.817167
H -0.482534 2.876359 -1.231898
H -0.081564 2.283193 1.717073
H -2.228831 1.433886 1.124622
H -2.420723 2.941342 1.985284
H -2.766288 4.137915 -0.205951

H -2.701594 2.606065 -1.044403
H -4.744135 3.382001 1.085275
H -5.024013 3.368256 -0.644953
H -4.468572 0.914824 1.096312
H -6.007864 1.401896 0.422847
H -4.968322 1.156214 -1.903012
H -3.564305 0.447900 -1.135101
H -5.087603 -1.353388 -1.901132
H -5.029463 -1.358169 -0.137768
H -6.417750 -0.659294 -0.970240
H -3.520876 -2.201701 1.725475
H 1.901863 2.385099 0.209071
H 0.493458 4.375618 0.685358

2a-c77, ΔG = 2.3701 kcal/mol, population = 0.34 %
C -0.010112 -5.595047 -0.028907
C 0.899141 -4.753858 -0.922754
C 0.882839 -3.307051 -0.529440
C -0.155875 -2.717609 0.131548
C -1.294220 -3.513243 0.514833
C -1.385506 -4.956634 0.036337
C -0.186621 -1.241335 0.485482
N 1.999704 -2.576296 -0.868843
C 2.142136 -1.284573 -0.456688
C 1.158681 -0.639577 0.217026
C 3.395998 -0.486697 -0.643689
C 2.997865 0.912157 -0.120445
C 1.643653 0.657789 0.607278
O 1.155937 1.468353 1.382874
O 3.957195 1.389547 0.808595
C 2.740580 1.872700 -1.255308
C 3.250153 3.093211 -1.345249
C -1.302415 -0.497258 -0.304075
C -2.113307 0.441247 0.574326
C -3.176568 1.211714 -0.209061
C -2.635907 2.134607 -1.301466
C -1.392170 2.974718 -0.987708
C -1.562587 4.025951 0.109981
C -0.261881 4.755949 0.459486
C 0.345452 5.567974 -0.685354
C 1.593266 6.339829 -0.259605
O -2.226291 -3.066046 1.198809
O -2.256550 -5.697889 0.870099
O -2.781197 -0.276502 1.631907

O -3.970442 1.978364 0.710955
H -0.087020 -6.609951 -0.418927
H 0.403737 -5.651418 0.980581
H 0.571375 -4.828004 -1.965857
H 1.922766 -5.130380 -0.889092
H -1.800016 -4.905444 -0.983679
H -0.416906 -1.149363 1.549979
H 2.773539 -3.046555 -1.315448
H 4.208446 -0.888211 -0.035403
H 3.723236 -0.480495 -1.684365
H 3.503382 2.031183 1.375016
H 2.060947 1.503486 -2.018109
H 3.007873 3.739896 -2.178312
H 3.928521 3.480621 -0.595907
H -0.845415 0.061297 -1.120609
H -1.978123 -1.221132 -0.766557
H -1.443020 1.153333 1.060582
H -3.828538 0.467680 -0.688095
H -2.419552 1.514272 -2.175172
H -3.457996 2.792560 -1.598890
H -0.555079 2.323879 -0.722093
H -1.094774 3.467997 -1.916745
H -2.322474 4.752516 -0.201339
H -1.954861 3.546998 1.008527
H -0.449645 5.429139 1.303331
H 0.472910 4.021746 0.806854
H 0.602630 4.904015 -1.514542
H -0.406130 6.266295 -1.070558
H 2.017605 6.908633 -1.090112
H 1.364158 7.042921 0.545702
H 2.365473 5.658086 0.105549
H -2.831969 -5.042298 1.297611
H -2.683262 -1.234442 1.481372
H -4.164653 1.376996 1.443996

2a-c65, ΔG = 2.5897 kcal/mol, population = 0.23 %
C -1.765170 -3.647490 -0.151438
C -0.894235 -2.935440 -1.183912
C 0.124504 -2.042443 -0.539418
C -0.023882 -1.489447 0.699363
C -1.188412 -1.839419 1.485406
C -2.318585 -2.622943 0.821315
C 1.002266 -0.545320 1.318378
N 1.262862 -1.810569 -1.280633

C 2.314419 -1.129477 -0.745354
C 2.244350 -0.554153 0.478201
C 3.639630 -0.993483 -1.426288
C 4.421852 -0.049367 -0.486640
C 3.515936 0.043361 0.780516
O 3.904568 0.542775 1.828537
O 5.668108 -0.635312 -0.135479
C 4.558027 1.328393 -1.082424
C 5.704322 1.978191 -1.234003
C 0.425179 0.880659 1.497506
C 0.137071 1.620632 0.195550
C -0.955569 2.689438 0.334824
C -2.345641 2.154886 0.664939
C -2.888009 1.106860 -0.306771
C -4.287797 0.604986 0.061471
C -5.387180 1.676022 0.064991
C -5.537216 2.453222 -1.247197
C -5.871542 1.574395 -2.452338
O -1.318213 -1.521818 2.669502
O -3.134374 -3.225435 1.808238
O 1.322624 2.245968 -0.343321
O -1.046121 3.429166 -0.889895
H -2.581130 -4.174775 -0.645964
H -1.172574 -4.381660 0.399412
H -1.520377 -2.319545 -1.839557
H -0.389825 -3.660204 -1.824954
H -2.909246 -1.887226 0.255842
H 1.239077 -0.905687 2.323497
H 1.367916 -2.273153 -2.171595
H 4.149471 -1.956496 -1.490301
H 3.534894 -0.599588 -2.438343
H 5.968301 -0.196290 0.673572
H 3.616654 1.784732 -1.372864
H 5.729810 2.972160 -1.662272
H 6.650226 1.535740 -0.947638
H -0.484323 0.792765 2.088710
H 1.123697 1.473328 2.094715
H -0.163825 0.920215 -0.584111
H -0.648994 3.372425 1.141014
H -2.339340 1.746339 1.678547
H -3.015508 3.017067 0.692322
H -2.887618 1.516806 -1.318521
H -2.213906 0.248162 -0.332539
H -4.565023 -0.193478 -0.632583

H -4.250813 0.142471 1.053557
H -6.339939 1.189588 0.299113
H -5.204234 2.385268 0.877038
H -6.326129 3.199996 -1.117423
H -4.621259 3.016046 -1.449907
H -6.040118 2.179250 -3.345850
H -5.064884 0.873717 -2.677097
H -6.776614 0.988590 -2.269120
H -2.935760 -2.745462 2.630733
H 1.748348 2.748189 0.364104
H -0.134761 3.541212 -1.197035

2a-c109, ΔG = 2.7736 kcal/mol, population = 0.17 %
C -1.692434 -4.312960 -1.004342
C -0.187371 -4.175910 -1.219080
C 0.316470 -2.816616 -0.834188
C -0.451948 -1.694062 -0.795029
C -1.870722 -1.816717 -1.068674
C -2.398564 -3.129574 -1.640173
C 0.108337 -0.307293 -0.483520
N 1.653031 -2.763143 -0.480550
C 2.173176 -1.642823 0.073123
C 1.473852 -0.474248 0.130099
C 3.510501 -1.607314 0.741632
C 3.646277 -0.135761 1.182724
C 2.247450 0.469365 0.874120
O 1.936282 1.604754 1.241261
O 3.889310 -0.074876 2.581290
C 4.678686 0.598294 0.365513
C 5.694738 1.286724 0.866939
C 0.113744 0.532872 -1.793269
C 0.216708 2.060273 -1.714139
C -0.802286 2.758453 -0.805735
C -2.250326 2.483204 -1.171234
C -3.261909 3.224530 -0.285592
C -3.142107 2.929715 1.213906
C -3.364470 1.463606 1.581550
C -3.177504 1.170759 3.068871
C -3.369270 -0.306968 3.406853
O -2.672146 -0.902668 -0.875320
O -3.800034 -3.204953 -1.464305
O 1.531171 2.532785 -1.368947
O -0.579126 4.177297 -0.878784
H -2.053168 -5.244616 -1.440333

H -1.919781 -4.329965 0.064213
H 0.057907 -4.342294 -2.274101
H 0.349171 -4.936717 -0.649721
H -2.159181 -3.109202 -2.715272
H -0.557231 0.174485 0.235561
H 2.182867 -3.622673 -0.458263
H 3.525588 -2.260466 1.615804
H 4.306149 -1.928268 0.067991
H 3.692635 0.828823 2.867192
H 4.528074 0.542885 -0.708850
H 6.395460 1.798381 0.219864
H 5.865583 1.351406 1.934171
H 0.930409 0.177599 -2.426350
H -0.810862 0.309234 -2.326860
H 0.057574 2.432534 -2.729776
H -0.612041 2.431133 0.223502
H -2.404577 2.775914 -2.215016
H -2.425664 1.409405 -1.111502
H -3.156071 4.299634 -0.445876
H -4.269360 2.957448 -0.621170
H -2.157734 3.246452 1.572968
H -3.868907 3.547870 1.751360
H -4.374811 1.165396 1.276759
H -2.685830 0.827045 1.009896
H -2.172926 1.486621 3.370810
H -3.877822 1.777070 3.653462
H -2.658070 -0.927450 2.854965
H -3.224713 -0.499014 4.472343
H -4.375090 -0.642286 3.140313
H -4.084353 -2.292751 -1.281195
H 1.684808 2.334017 -0.427725
H 0.381673 4.290061 -0.905284

2a-c87, ΔG = 3.6640 kcal/mol, population = 0.04 %
C -1.584517 -4.130787 0.175982
C -0.437239 -3.891552 -0.804256
C 0.292557 -2.611311 -0.524606
C -0.241271 -1.553811 0.143486
C -1.557481 -1.696883 0.735198
C -2.424639 -2.870232 0.295548
C 0.476958 -0.217382 0.289436
N 1.601691 -2.561055 -0.975270
C 2.417743 -1.547821 -0.598539
C 1.942909 -0.442040 0.040850

C 3.903863 -1.583277 -0.763260
C 4.355145 -0.216316 -0.205389
C 3.054248 0.377317 0.406232
O 3.061518 1.406579 1.085143
O 5.315888 -0.413151 0.822718
C 4.834739 0.700949 -1.301853
C 6.010409 1.313514 -1.318678
C -0.157082 0.793431 -0.710800
C -0.031114 2.295087 -0.440475
C -0.628995 2.775221 0.888574
C -2.095404 2.423740 1.099233
C -3.047485 2.867555 -0.012713
C -4.519790 2.595659 0.314580
C -4.875319 1.119400 0.540614
C -4.546082 0.181173 -0.625327
C -5.246177 0.540614 -1.935058
O -2.010122 -0.908468 1.564420
O -3.501613 -3.043657 1.195059
O 1.306538 2.803790 -0.555326
O -0.482788 4.203017 0.964513
H -2.203084 -4.961618 -0.163723
H -1.188977 -4.386794 1.161704
H -0.824624 -3.839613 -1.828221
H 0.264982 -4.726729 -0.778720
H -2.812623 -2.604842 -0.700239
H 0.323887 0.146186 1.306938
H 1.998553 -3.386950 -1.399875
H 4.341410 -2.387812 -0.169820
H 4.191656 -1.737604 -1.804015
H 5.365410 0.409749 1.330026
H 4.124803 0.850655 -2.110470
H 6.287768 1.966191 -2.136364
H 6.734910 1.174263 -0.526158
H 0.249882 0.592112 -1.704493
H -1.224649 0.575270 -0.768575
H -0.571905 2.799019 -1.243767
H -0.047728 2.325815 1.705085
H -2.163306 1.347260 1.251041
H -2.407149 2.885171 2.042060
H -2.916674 3.936795 -0.195044
H -2.790350 2.366941 -0.950179
H -4.791611 3.158246 1.214041
H -5.138806 2.999125 -0.491637
H -4.364186 0.753128 1.434334

H -5.947856 1.052483 0.752905
H -3.465336 0.161443 -0.787525
H -4.820675 -0.836962 -0.335346
H -5.029950 -0.196593 -2.711331
H -6.330889 0.577867 -1.801793
H -4.925842 1.515356 -2.308422
H -3.542772 -2.217622 1.707673
H 1.860611 2.411958 0.145696
H 0.386874 4.398049 0.586734

Table S5. Geometry data of conformers of structure **2b**.

2b-c387, ΔG = 0.0000 kcal/mol, population = 11.32 %
C 2.652344 -0.581118 2.933736
C 2.618246 0.921077 2.667306
C 1.744074 1.262478 1.497349
C 1.389010 0.374546 0.527691
C 1.941614 -0.963568 0.564972
C 2.970995 -1.305383 1.639636
C 0.419076 0.709642 -0.596469
N 1.319251 2.576317 1.436890
C 0.598595 3.026285 0.380379
C 0.196865 2.194185 -0.617662
C 0.152619 4.445428 0.221968
C -0.501220 4.455775 -1.178323
C -0.582028 2.948478 -1.550791
O -1.268639 2.547036 -2.491727
O -1.813250 4.991940 -1.095197
C 0.367678 5.163744 -2.187801
C -0.040767 6.142280 -2.983763
C -0.929156 -0.058651 -0.448482
C -1.265466 -0.944874 -1.637998
C -2.606864 -1.665347 -1.467297
C -2.676019 -2.603607 -0.266346
C -1.532051 -3.611732 -0.149332
C -1.620072 -4.462095 1.116796
C -0.430371 -5.409506 1.272212
C -0.471252 -6.283071 2.529500
C -0.368073 -5.494188 3.835710
O 1.634906 -1.836740 -0.248118
O 3.024016 -2.708414 1.827267
O -1.297010 -0.224739 -2.883151
O -2.881798 -2.420096 -2.657437
H 3.401733 -0.814564 3.690028

H 1.681683 -0.920322 3.303542
H 3.629359 1.290775 2.461555
H 2.264007 1.459985 3.548335
H 3.941050 -0.944800 1.262334
H 0.871601 0.397925 -1.541310
H 1.578092 3.210895 2.178489
H -0.595741 4.702496 0.973693
H 0.982492 5.146737 0.316423
H -2.295732 4.688384 -1.877774
H 1.391077 4.803107 -2.238868
H 0.634756 6.603643 -3.692532
H -1.054582 6.520099 -2.943682
H -1.739466 0.657485 -0.284023
H -0.884478 -0.679407 0.445687
H -0.478418 -1.690891 -1.750752
H -3.383553 -0.895728 -1.352823
H -2.714886 -1.992667 0.639824
H -3.632844 -3.132393 -0.314027
H -1.522270 -4.260459 -1.029994
H -0.574340 -3.086753 -0.142225
H -1.677321 -3.795226 1.982757
H -2.550175 -5.042480 1.113606
H -0.376419 -6.055535 0.389575
H 0.493881 -4.819905 1.277450
H -1.394469 -6.872802 2.529310
H 0.351081 -7.003437 2.483290
H -1.220044 -4.825680 3.973427
H -0.331728 -6.162936 4.698431
H 0.538362 -4.882433 3.848419
H 2.655910 -3.091247 1.012729
H -1.418275 0.731692 -2.721493
H -2.642792 -1.833659 -3.389863

2b-c422, ΔG = 0.1493 kcal/mol, population = 8.79 %
C 2.643929 -0.353203 2.997164
C 2.532997 1.139740 2.701275
C 1.664625 1.411127 1.508868
C 1.378025 0.487513 0.549939
C 2.002402 -0.817129 0.623181
C 3.027344 -1.082217 1.723460
C 0.413416 0.748066 -0.598320
N 1.169618 2.698173 1.415298
C 0.446244 3.088397 0.337096
C 0.110491 2.217413 -0.652088

C -0.073672 4.477645 0.143161
C -0.699913 4.426370 -1.268803
C -0.690214 2.910418 -1.613844
O -1.335037 2.454568 -2.559537
O -2.040979 4.890106 -1.219801
C 0.147752 5.163293 -2.275572
C -0.298774 6.103078 -3.097363
C -0.893220 -0.090803 -0.460690
C -1.156812 -1.015880 -1.638898
C -2.459038 -1.807190 -1.478834
C -2.499144 -2.725522 -0.261270
C -1.303107 -3.665348 -0.103447
C -1.367018 -4.494276 1.178245
C -0.129023 -5.369552 1.374200
C -0.144112 -6.218877 2.648558
C -0.109920 -5.399612 3.939607
O 1.760327 -1.720432 -0.178725
O 3.153197 -2.476575 1.938968
O -1.204573 -0.321329 -2.898000
O -2.668271 -2.598026 -2.658880
H 3.389825 -0.531226 3.771768
H 1.686134 -0.738563 3.355117
H 3.526238 1.560707 2.507313
H 2.132569 1.674275 3.565018
H 3.983559 -0.675724 1.357662
H 0.900843 0.444772 -1.528436
H 1.378509 3.359424 2.149309
H -0.849329 4.706869 0.875983
H 0.714627 5.225190 0.239158
H -2.490775 4.546214 -2.005157
H 1.190329 4.859317 -2.301105
H 0.363727 6.588475 -3.802365
H -1.332510 6.424577 -3.082991
H -1.744771 0.582264 -0.325231
H -0.832056 -0.691678 0.445995
H -0.327421 -1.718555 -1.722994
H -3.279496 -1.080367 -1.393736
H -2.589703 -2.101040 0.631884
H -3.423851 -3.308018 -0.316182
H -1.240666 -4.329640 -0.970241
H -0.376507 -3.087363 -0.090339
H -1.477871 -3.814781 2.029057
H -2.262800 -5.126312 1.170727
H -0.022355 -6.028894 0.506325

H 0.760327 -4.728553 1.383294
H -1.032492 -6.859877 2.644518
H 0.718204 -6.892247 2.631818
H 0.760218 -4.737303 3.955378
H -1.000622 -4.777885 4.048383
H -0.052060 -6.047944 4.816620
H 2.822675 -2.893820 1.125208
H -1.380703 0.629799 -2.756876
H -2.448621 -2.012600 -3.398135

2b-c220, ΔG = 0.2880 kcal/mol, population = 6.96 %
C 5.331400 0.171001 -0.239727
C 4.785188 1.594333 -0.169603
C 3.307643 1.617626 0.080663
C 2.481688 0.555289 -0.143673
C 3.021707 -0.651786 -0.716496
C 4.473153 -0.655329 -1.182390
C 1.007548 0.571843 0.223298
N 2.789267 2.814499 0.525003
C 1.442363 3.012393 0.575491
C 0.564989 1.997999 0.367887
C 0.808634 4.341537 0.839267
C -0.684753 4.079753 0.541297
C -0.771294 2.527053 0.403188
O -1.841537 1.934915 0.354711
O -1.482134 4.498646 1.639762
C -1.098593 4.706000 -0.765681
C -2.146851 5.501839 -0.927450
C 0.768709 -0.254801 1.513021
C -0.615458 -0.892009 1.660886
C -0.983089 -1.807315 0.483900
C -2.341195 -2.469195 0.662272
C -2.820141 -3.218924 -0.578608
C -4.191094 -3.866310 -0.391601
C -4.684856 -4.612235 -1.629734
C -6.060743 -5.252099 -1.447794
C -6.547155 -5.992770 -2.692156
O 2.356052 -1.684595 -0.873314
O 4.954038 -1.982380 -1.284581
O -0.654797 -1.621544 2.897671
O 0.008944 -2.833802 0.328942
H 6.364169 0.179958 -0.587650
H 5.313295 -0.288933 0.751182
H 4.979016 2.120050 -1.111652

H 5.291973 2.163433 0.612395
H 4.477798 -0.180175 -2.176313
H 0.459946 0.107833 -0.598067
H 3.415727 3.589175 0.689646
H 0.927413 4.628365 1.885737
H 1.238201 5.130800 0.221078
H -2.332240 4.041351 1.565180
H -0.470822 4.444642 -1.612999
H -2.393449 5.909970 -1.899171
H -2.781492 5.779685 -0.095455
H 0.953054 0.381027 2.380836
H 1.508077 -1.057001 1.554657
H -1.379727 -0.119460 1.743309
H -1.022736 -1.198020 -0.425066
H -2.295170 -3.150277 1.516078
H -3.065160 -1.690327 0.921660
H -2.861689 -2.524565 -1.425882
H -2.086694 -3.985532 -0.842373
H -4.150518 -4.560399 0.455786
H -4.921247 -3.095164 -0.119959
H -4.719621 -3.919698 -2.478840
H -3.958969 -5.388464 -1.898209
H -6.025473 -5.944755 -0.600068
H -6.784256 -4.475462 -1.177876
H -6.621435 -5.315559 -3.547184
H -5.857122 -6.795587 -2.965137
H -7.531447 -6.438864 -2.533689
H 4.164094 -2.542544 -1.362790
H -0.120991 -2.416740 2.761600
H 0.796004 -2.439600 -0.087645

2b-c189, ΔG = 0.3753 kcal/mol, population = 6.00 %
C 5.207269 1.095828 -0.017967
C 4.237280 2.242872 0.248308
C 2.823338 1.764798 0.385492
C 2.376933 0.559284 -0.073059
C 3.284027 -0.294574 -0.797666
C 4.669455 0.236665 -1.148750
C 0.962334 0.057478 0.173173
N 1.950083 2.645431 0.983254
C 0.609840 2.403401 0.986274
C 0.095896 1.224602 0.552188
C -0.407757 3.397048 1.450990
C -1.743252 2.749126 1.024772

C -1.339477 1.295680 0.626028
O -2.172317 0.418323 0.442993
O -2.641194 2.702408 2.123679
C -2.323328 3.434000 -0.186536
C -3.573027 3.866081 -0.287459
C 0.961786 -1.052996 1.256549
C -0.129286 -2.118161 1.119117
C -0.050839 -2.891132 -0.206247
C -1.106744 -3.983690 -0.336519
C -2.555178 -3.500107 -0.227228
C -2.955743 -2.460072 -1.273865
C -4.417429 -2.032515 -1.162503
C -4.812339 -0.959074 -2.175612
C -6.269294 -0.518648 -2.046180
O 2.987300 -1.435529 -1.178123
O 5.546375 -0.834231 -1.442776
O -0.035365 -3.026260 2.227775
O 1.235279 -3.520760 -0.327271
H 6.190547 1.485601 -0.280621
H 5.316813 0.477920 0.876412
H 4.272844 2.965582 -0.575012
H 4.520924 2.786931 1.151548
H 4.539090 0.867334 -2.042400
H 0.598484 -0.368762 -0.763132
H 2.301064 3.529962 1.320969
H -0.381715 3.498697 2.537534
H -0.248200 4.383591 1.014163
H -3.290022 2.010352 1.929176
H -1.635338 3.541760 -1.020362
H -3.928985 4.338600 -1.193907
H -4.273098 3.772835 0.533146
H 0.865668 -0.587151 2.239017
H 1.929977 -1.556923 1.247349
H -1.108164 -1.648154 1.194685
H -0.178932 -2.182675 -1.028053
H -0.955524 -4.466399 -1.306462
H -0.914877 -4.740293 0.427825
H -3.212809 -4.370503 -0.319285
H -2.735340 -3.093290 0.772286
H -2.332720 -1.569239 -1.167894
H -2.766846 -2.863991 -2.275579
H -5.067937 -2.906496 -1.286642
H -4.601033 -1.653560 -0.150709
H -4.155733 -0.092314 -2.048360

H -4.631771 -1.335580 -3.188277
H -6.523394 0.248923 -2.780635
H -6.949226 -1.361717 -2.195685
H -6.466095 -0.107405 -1.052453
H 4.974295 -1.586162 -1.669562
H 0.735261 -3.585223 2.055198
H 1.870527 -2.837862 -0.606709

2b-c306, ΔG = 0.5051 kcal/mol, population = 4.82 %
C 4.078006 -0.636476 0.133554
C 3.834219 0.832047 0.474437
C 2.376091 1.178499 0.439282
C 1.435531 0.438174 -0.206840
C 1.847603 -0.703189 -0.995844
C 3.339768 -0.984582 -1.147075
C -0.051611 0.727476 -0.100363
N 2.014803 2.351990 1.078342
C 0.776413 2.875116 0.899930
C -0.211610 2.170590 0.279815
C 0.381306 4.256966 1.314466
C -0.989460 4.448573 0.623564
C -1.347968 3.021333 0.123069
O -2.466607 2.743119 -0.313260
O -1.955202 4.872600 1.574130
C -0.877839 5.370420 -0.564685
C -1.636571 6.438057 -0.770402
C -0.713380 -0.263487 0.904267
C -2.096506 -0.753995 0.495206
C -2.602310 -1.907246 1.365097
C -1.734214 -3.158976 1.385130
C -1.354017 -3.741152 0.023354
C -0.645712 -5.090377 0.164208
C -0.212043 -5.721421 -1.161406
C 0.930156 -4.985101 -1.863681
C 1.368674 -5.665844 -3.158888
O 1.045914 -1.442420 -1.567477
O 3.542173 -2.337768 -1.513119
O -3.114427 0.249149 0.618832
O -3.916315 -2.280402 0.919585
H 5.145014 -0.823741 0.013960
H 3.711768 -1.275863 0.940461
H 4.360155 1.478319 -0.237716
H 4.235128 1.068952 1.462334
H 3.697758 -0.324169 -1.952622

H -0.500418 0.552906 -1.081863
H 2.728293 2.893990 1.544575
H 0.252729 4.315916 2.396668
H 1.120301 5.002166 1.018775
H -2.828410 4.703584 1.192201
H -0.114582 5.090981 -1.285436
H -1.507060 7.052125 -1.652289
H -2.399753 6.738896 -0.063601
H -0.758463 0.190612 1.897478
H -0.059851 -1.131856 0.980450
H -2.052505 -1.096568 -0.545501
H -2.676187 -1.523874 2.392089
H -0.827476 -2.945458 1.957534
H -2.281799 -3.910787 1.962031
H -2.254510 -3.869605 -0.584453
H -0.710211 -3.040505 -0.512294
H 0.234301 -4.971151 0.807887
H -1.313927 -5.781952 0.688133
H 0.099418 -6.755545 -0.978538
H -1.074982 -5.777041 -1.835506
H 0.632523 -3.955951 -2.075709
H 1.781452 -4.921562 -1.176447
H 2.189864 -5.126655 -3.636606
H 1.705474 -6.689022 -2.971667
H 0.542917 -5.715289 -3.873794
H 2.693509 -2.639057 -1.879309
H -2.886932 1.047862 0.103988
H -4.372867 -1.446415 0.739046

2b-c337, ΔG = 0.5271 kcal/mol, population = 4.64 %
C 5.269718 0.084950 0.510245
C 4.765220 1.523829 0.445388
C 3.268611 1.595907 0.401787
C 2.466123 0.550073 0.049258
C 3.068532 -0.686019 -0.381415
C 4.580792 -0.740241 -0.562701
C 0.949224 0.620166 0.116222
N 2.716146 2.821345 0.703028
C 1.391587 3.058020 0.490425
C 0.536067 2.062955 0.143662
C 0.763401 4.411440 0.600174
C -0.656333 4.182916 0.035673
C -0.766045 2.630024 -0.080986
O -1.828364 2.067126 -0.311532

O -1.629583 4.653297 0.957334
C -0.801446 4.782933 -1.339328
C -1.779274 5.596784 -1.713455
C 0.425843 -0.166593 1.346564
C -0.971349 -0.777781 1.209556
C -1.088712 -1.739775 0.018554
C -2.466479 -2.377119 -0.087509
C -2.660039 -3.172134 -1.378200
C -4.062913 -3.763957 -1.540237
C -4.419456 -4.834698 -0.508148
C -5.786294 -5.474263 -0.751063
C -6.138555 -6.542777 0.282352
O 2.414999 -1.705759 -0.641343
O 5.030579 -2.081836 -0.547798
O -1.301134 -1.450995 2.434914
O -0.111453 -2.785455 0.132985
H 6.349583 0.057151 0.366093
H 5.048440 -0.349046 1.488190
H 5.155131 2.020795 -0.450447
H 5.127682 2.097619 1.300689
H 4.788191 -0.288999 -1.545889
H 0.559917 0.153173 -0.789711
H 3.325330 3.582488 0.966891
H 0.696211 4.724880 1.643619
H 1.325316 5.170241 0.054429
H -2.465118 4.217502 0.735182
H -0.034094 4.483674 -2.047797
H -1.828922 5.982376 -2.723655
H -2.551093 5.911896 -1.022531
H 0.432802 0.491441 2.217332
H 1.122897 -0.977948 1.565886
H -1.714388 0.010016 1.088136
H -0.908282 -1.172053 -0.900228
H -2.624009 -3.013290 0.785259
H -3.215914 -1.580597 -0.035192
H -2.451901 -2.514313 -2.229066
H -1.920076 -3.975996 -1.421040
H -4.804829 -2.957999 -1.495038
H -4.147359 -4.201018 -2.540892
H -3.648736 -5.614590 -0.519867
H -4.404328 -4.404929 0.498160
H -6.553745 -4.692825 -0.746046
H -5.804024 -5.914084 -1.753890
H -6.159883 -6.120570 1.290557

H -7.117714 -6.984688 0.085065
H -5.400942 -7.349756 0.277698
H 4.251295 -2.621950 -0.759930
H -0.772342 -2.260863 2.451671
H 0.753126 -2.423148 -0.131736

2b-c89, ΔG = 0.5447 kcal/mol, population = 4.51 %
C 5.073297 -0.047560 1.087300
C 4.196553 1.095097 1.591644
C 2.896631 1.176526 0.848771
C 2.680083 0.620801 -0.375477
C 3.776194 -0.038097 -1.050540
C 5.167838 0.030327 -0.425546
C 1.316719 0.621479 -1.050834
N 1.897953 1.891417 1.483438
C 0.725174 2.156421 0.851361
C 0.440516 1.633066 -0.368703
C -0.354622 3.016865 1.427658
C -1.367557 3.134733 0.267585
C -0.855694 2.096150 -0.779117
O -1.528625 1.759737 -1.745566
O -2.661939 2.748536 0.708915
C -1.337293 4.508110 -0.351172
C -2.402357 5.267496 -0.569259
C 0.640495 -0.770412 -1.040732
C 0.513057 -1.393403 0.347899
C -0.786839 -2.188201 0.532544
C -2.002524 -1.276290 0.641160
C -3.333852 -2.011707 0.813724
C -3.740712 -2.862732 -0.389161
C -5.118655 -3.502919 -0.231113
C -5.529265 -4.366386 -1.423236
C -6.910338 -4.997992 -1.257995
O 3.644674 -0.634349 -2.121142
O 5.984558 -0.999788 -0.951219
O 1.642436 -2.236024 0.660420
O -0.700228 -2.962916 1.733866
H 6.067545 0.014435 1.529880
H 4.636168 -1.008599 1.367432
H 4.719197 2.051613 1.476672
H 3.991067 0.979970 2.658055
H 5.583272 1.011570 -0.704785
H 1.447991 0.897416 -2.100609
H 2.077821 2.277786 2.398439

H -0.828856 2.522832 2.277765
H 0.026345 3.981173 1.766167
H -3.166145 2.496892 -0.078573
H -0.348055 4.852819 -0.639331
H -2.307830 6.242657 -1.029482
H -3.396403 4.945796 -0.284988
H 1.204327 -1.447171 -1.685202
H -0.343504 -0.657550 -1.495947
H 0.535824 -0.624541 1.122967
H -0.898020 -2.866288 -0.325051
H -1.841168 -0.606126 1.491195
H -2.057619 -0.646017 -0.248967
H -3.288650 -2.640747 1.706647
H -4.113737 -1.266153 0.998686
H -3.731944 -2.240217 -1.291534
H -3.000746 -3.652095 -0.554376
H -5.130601 -4.115126 0.678157
H -5.867876 -2.717069 -0.080326
H -5.512476 -3.754851 -2.331705
H -4.781900 -5.153295 -1.570502
H -6.944637 -5.638713 -0.372804
H -7.680369 -4.230821 -1.140788
H -7.177659 -5.609269 -2.122745
H 5.549950 -1.273085 -1.777140
H 1.855012 -2.763911 -0.119827
H 0.220054 -3.262451 1.779700

2b-c73, ΔG = 0.7267 kcal/mol, population = 3.32 %
C 4.878199 -0.427906 1.289663
C 4.639445 1.079143 1.277520
C 3.263530 1.428510 0.794449
C 2.475303 0.591097 0.065617
C 3.003424 -0.693872 -0.344258
C 4.468269 -1.008749 -0.051600
C 1.043695 0.926236 -0.325842
N 2.830506 2.704034 1.108599
C 1.671599 3.185417 0.597194
C 0.835750 2.399233 -0.133770
C 1.193079 4.591275 0.780174
C -0.049185 4.663741 -0.138546
C -0.281792 3.183241 -0.549018
O -1.310861 2.811341 -1.116503
O -1.168834 5.128773 0.601464
C 0.227491 5.479793 -1.376059

C -0.522723 6.485497 -1.804601
C 0.011665 0.079754 0.484135
C -0.952140 -0.724265 -0.380245
C -1.819183 -1.675002 0.457495
C -2.553544 -2.696265 -0.408577
C -3.379180 -3.697318 0.395446
C -4.102574 -4.718090 -0.481418
C -4.945541 -5.712590 0.314906
C -5.665825 -6.739193 -0.558651
C -6.512031 -7.722928 0.247640
O 2.329174 -1.533232 -0.942271
O 4.682589 -2.407108 -0.100341
O -1.861165 0.099026 -1.127377
O -2.730657 -0.908207 1.257317
H 5.928602 -0.642363 1.486295
H 4.284786 -0.897703 2.077823
H 5.367294 1.571443 0.622412
H 4.786330 1.499387 2.274741
H 5.052690 -0.515305 -0.844326
H 0.918747 0.676525 -1.383676
H 3.428137 3.302757 1.660085
H 0.890277 4.766121 1.814062
H 1.960853 5.322013 0.524093
H -1.963545 4.879592 0.108190
H 1.109288 5.175264 -1.932673
H -0.269417 7.024127 -2.708620
H -1.402359 6.809697 -1.262845
H -0.553197 0.729525 1.156415
H 0.556789 -0.622389 1.116806
H -0.359911 -1.323180 -1.079464
H -1.175289 -2.204772 1.164278
H -3.198207 -2.161566 -1.113086
H -1.809337 -3.232831 -1.007146
H -2.723631 -4.224573 1.098521
H -4.109872 -3.155254 1.001809
H -4.746166 -4.189560 -1.194092
H -3.367756 -5.266610 -1.081931
H -4.304493 -6.237227 1.032928
H -5.685002 -5.163845 0.909583
H -6.301913 -6.213896 -1.279078
H -4.925920 -7.290322 -1.148745
H -7.015844 -8.443484 -0.400359
H -5.894109 -8.283979 0.953776
H -7.279118 -7.198534 0.823672

H 3.936451 -2.764248 -0.611187
H -1.581497 1.036176 -1.130131
H -3.090901 -0.242926 0.651300

2b-c598, ΔG = 0.7963 kcal/mol, population = 2.95 %
C 4.964090 0.357425 -0.790719
C 4.277841 1.714409 -0.659127
C 2.865444 1.584992 -0.175164
C 2.140713 0.431442 -0.246423
C 2.715736 -0.726948 -0.881957
C 4.067759 -0.588325 -1.572166
C 0.751941 0.297916 0.353775
N 2.290666 2.732701 0.326295
C 0.955529 2.782129 0.592775
C 0.178417 1.670364 0.547053
C 0.223796 4.040749 0.936774
C -1.257964 3.604022 0.899158
C -1.186660 2.047980 0.791890
O -2.175664 1.338551 0.922710
O -1.900880 3.960026 2.115119
C -1.957728 4.142496 -0.322098
C -3.109191 4.800197 -0.309636
C 0.818318 -0.507236 1.677511
C -0.442937 -1.287307 2.058739
C -0.895045 -2.259820 0.960395
C -2.131413 -3.054373 1.358531
C -2.676986 -3.961848 0.253318
C -3.161977 -3.219382 -0.992092
C -3.773693 -4.150987 -2.038335
C -4.325304 -3.434935 -3.275317
C -3.261132 -2.717970 -4.107453
O 2.153009 -1.829973 -0.918073
O 4.671749 -1.858399 -1.729468
O -0.194819 -1.988932 3.287422
O 0.160808 -3.182212 0.651774
H 5.922527 0.466859 -1.297806
H 5.151736 -0.067777 0.198066
H 4.259930 2.223886 -1.629493
H 4.833805 2.362323 0.021518
H 3.863799 -0.148193 -2.561149
H 0.137296 -0.249968 -0.361581
H 2.842149 3.577316 0.373891
H 0.480574 4.377030 1.942992
H 0.450452 4.849752 0.241425

H -2.694440 3.410484 2.189304
H -1.453552 3.936362 -1.262126
H -3.563009 5.148481 -1.228548
H -3.626922 5.021628 0.615149
H 1.067928 0.173175 2.493686
H 1.640511 -1.222457 1.608907
H -1.263393 -0.600333 2.265185
H -1.135779 -1.671805 0.071651
H -1.893021 -3.652139 2.241016
H -2.904539 -2.340818 1.661400
H -1.905529 -4.681828 -0.032900
H -3.507717 -4.544916 0.664054
H -3.905269 -2.467295 -0.700985
H -2.329071 -2.670513 -1.438108
H -3.019756 -4.882453 -2.352586
H -4.581152 -4.724792 -1.571127
H -4.837033 -4.167421 -3.906978
H -5.089220 -2.715105 -2.961674
H -2.787699 -1.907871 -3.549508
H -2.474327 -3.412751 -4.414506
H -3.694636 -2.284504 -5.011380
H 3.946851 -2.502481 -1.668955
H 0.387488 -2.728832 3.065164
H 0.825048 -2.719421 0.110405

2b-c202, ΔG = 0.8365 kcal/mol, population = 2.75 %
C 2.815550 -0.623992 2.791305
C 2.838181 0.876070 2.510663
C 1.898865 1.253735 1.404313
C 1.424267 0.378214 0.475639
C 1.903327 -0.988151 0.489351
C 2.993682 -1.375393 1.485321
C 0.394973 0.757484 -0.579033
N 1.543400 2.588660 1.357955
C 0.776004 3.069730 0.349200
C 0.259148 2.252486 -0.607338
C 0.398707 4.510146 0.204048
C -0.341972 4.544955 -1.152054
C -0.536505 3.041466 -1.496420
O -1.307521 2.671497 -2.383396
O -1.610969 5.161818 -0.996241
C 0.503358 5.186049 -2.224631
C 0.106010 6.179393 -3.007701
C -0.982822 0.075261 -0.321241

C -1.456730 -0.803169 -1.468848
C -2.829726 -1.426177 -1.198783
C -2.876690 -2.357452 0.008973
C -1.786224 -3.428500 0.053244
C -1.905320 -4.348450 1.266987
C -0.751289 -5.343991 1.371354
C -0.854690 -6.283266 2.572049
C 0.315840 -7.260367 2.667246
O 1.486349 -1.851031 -0.284685
O 2.986826 -2.777515 1.686067
O -1.518909 -0.100535 -2.722941
O -3.239743 -2.163065 -2.360725
H 3.608793 -0.890319 3.489783
H 1.860626 -0.908827 3.239628
H 3.848728 1.191900 2.227148
H 2.577574 1.439438 3.409003
H 3.948905 -1.070434 1.029333
H 0.756970 0.407973 -1.549226
H 1.892183 3.213426 2.070304
H -0.285258 4.812523 0.999010
H 1.269034 5.166517 0.237833
H -2.159068 4.878314 -1.742328
H 1.497254 4.761491 -2.334193
H 0.761807 6.589980 -3.764599
H -0.878117 6.619769 -2.908959
H -1.733845 0.842835 -0.112907
H -0.912326 -0.534509 0.578926
H -0.732537 -1.604392 -1.617457
H -3.541638 -0.604565 -1.035218
H -2.824629 -1.747193 0.914917
H -3.862722 -2.831921 0.020103
H -1.822845 -4.025500 -0.862628
H -0.801725 -2.954997 0.072501
H -1.938015 -3.740819 2.178972
H -2.856336 -4.891701 1.226300
H -0.701315 -5.938505 0.451743
H 0.193146 -4.790449 1.428878
H -0.910269 -5.688093 3.489806
H -1.794885 -6.841743 2.511937
H 0.376154 -7.886986 1.773415
H 1.264781 -6.725614 2.760854
H 0.218251 -7.920761 3.531702
H 2.535719 -3.146940 0.907826
H -1.576705 0.863586 -2.571827

H -3.002203 -1.601118 -3.112724

2b-c327, ΔG = 0.8980 kcal/mol, population = 2.48 %
C 5.425355 0.473020 0.211727
C 4.735688 1.832000 0.299286
C 3.243895 1.701151 0.363291
C 2.561866 0.586556 -0.027836
C 3.283546 -0.520371 -0.602416
C 4.772809 -0.355561 -0.881869
C 1.061259 0.439299 0.153804
N 2.557355 2.810061 0.808836
C 1.201443 2.879276 0.694760
C 0.462602 1.804785 0.318103
C 0.408646 4.122716 0.946727
C -0.998926 3.746688 0.431843
C -0.913797 2.204491 0.206430
O -1.905355 1.520154 -0.010277
O -1.973802 4.024248 1.426486
C -1.291978 4.411941 -0.888831
C -2.383249 5.115486 -1.158280
C 0.760605 -0.489286 1.358249
C -0.563011 -1.255439 1.307015
C -0.714958 -2.108084 0.039463
C -2.012936 -2.902320 0.026173
C -2.295940 -3.570482 -1.318819
C -3.552311 -4.445668 -1.325041
C -4.854657 -3.683916 -1.077241
C -6.099811 -4.562896 -1.192520
C -7.396778 -3.799958 -0.928992
O 2.747370 -1.595171 -0.905195
O 5.392729 -1.621441 -1.007608
O -0.664733 -2.075250 2.482433
O 0.384266 -3.024830 -0.075043
H 6.486381 0.601510 -0.001418
H 5.333742 -0.058513 1.161902
H 4.986088 2.439341 -0.578090
H 5.086617 2.387984 1.171089
H 4.847813 0.192474 -1.834576
H 0.664958 -0.019554 -0.752885
H 3.077774 3.625042 1.099639
H 0.359247 4.342268 2.014877
H 0.833985 4.990578 0.441577
H -2.758190 3.499833 1.209389
H -0.532575 4.261716 -1.651113

H -2.535725 5.557566 -2.134469
H -3.149811 5.281814 -0.411928
H 0.785969 0.101148 2.275985
H 1.564886 -1.223179 1.440268
H -1.402110 -0.561493 1.352339
H -0.723147 -1.436833 -0.825390
H -1.975936 -3.657190 0.816455
H -2.824219 -2.217730 0.285244
H -2.391519 -2.797695 -2.090702
H -1.432873 -4.180317 -1.596005
H -3.621781 -4.955723 -2.291764
H -3.446756 -5.233758 -0.570359
H -4.836968 -3.226707 -0.083149
H -4.928613 -2.856625 -1.793253
H -6.133502 -5.009632 -2.191905
H -6.016363 -5.396938 -0.487440
H -7.518046 -2.977132 -1.638555
H -8.269909 -4.449889 -1.019402
H -7.401736 -3.372279 0.077113
H 4.676940 -2.244047 -1.217362
H -0.044377 -2.807173 2.359284
H 1.169546 -2.531641 -0.372514

2b-c178, ΔG = 1.0341 kcal/mol, population = 1.97 %
C 2.602536 -0.427986 2.989495
C 2.298506 1.053510 2.784921
C 1.463054 1.290865 1.561955
C 1.350241 0.406375 0.533144
C 2.136481 -0.809022 0.561875
C 3.141693 -1.005212 1.693466
C 0.417259 0.616450 -0.650746
N 0.806395 2.506510 1.517610
C 0.091370 2.872354 0.425399
C -0.077085 2.033776 -0.632267
C -0.599434 4.192598 0.287021
C -1.136721 4.157261 -1.162335
C -0.915970 2.680146 -1.593537
O -1.452134 2.209030 -2.597821
O -2.525676 4.449442 -1.173746
C -0.332737 5.054380 -2.070238
C -0.847738 5.972791 -2.876004
C -0.767303 -0.395379 -0.634818
C -0.858043 -1.261188 -1.881973
C -2.056808 -2.214678 -1.838082

C -2.046296 -3.196115 -0.671990
C -0.796674 -4.075630 -0.551907
C -0.709196 -4.811873 0.789812
C -0.323182 -3.901211 1.957194
C -0.223966 -4.631203 3.295761
C 0.189232 -3.712430 4.444342
O 2.048269 -1.683343 -0.301196
O 3.459901 -2.378513 1.825293
O -0.938389 -0.494633 -3.096520
O -2.109457 -2.954094 -3.067256
H 3.329597 -0.558319 3.790965
H 1.692687 -0.964052 3.268452
H 3.231226 1.619495 2.680409
H 1.782733 1.463025 3.655887
H 4.043316 -0.442329 1.404370
H 0.986167 0.442688 -1.567658
H 0.887716 3.137488 2.301377
H -1.437014 4.266791 0.982898
H 0.074682 5.028659 0.477471
H -2.883486 4.099082 -2.002461
H 0.740409 4.889140 -2.037798
H -0.212745 6.578497 -3.509618
H -1.913941 6.156352 -2.918686
H -1.707318 0.146476 -0.494211
H -0.657752 -1.046492 0.231083
H 0.057172 -1.847338 -1.966980
H -2.962850 -1.596434 -1.760770
H -2.189557 -2.620210 0.244287
H -2.929496 -3.835301 -0.763625
H -0.791053 -4.795720 -1.373098
H 0.105538 -3.470674 -0.664938
H -1.664751 -5.303709 1.006161
H 0.035682 -5.610350 0.711680
H 0.638482 -3.427787 1.733367
H -1.048227 -3.086491 2.055324
H -1.188850 -5.095672 3.525379
H 0.496835 -5.450907 3.205847
H 0.241720 -4.252758 5.392079
H 1.170907 -3.270626 4.256965
H -0.524292 -2.892961 4.566269
H 3.203305 -2.785042 0.980003
H -1.245659 0.413956 -2.907268
H -1.936014 -2.303147 -3.762594

2b-c430, ΔG = 1.0461 kcal/mol, population = 1.93 %

C 4.004943 -0.833858 0.026214
C 3.715149 0.557795 0.584648
C 2.272330 0.933534 0.429731
C 1.408476 0.310776 -0.416885
C 1.908622 -0.702187 -1.321911
C 3.414390 -0.944723 -1.369416
C -0.081638 0.608218 -0.419904
N 1.844585 2.018418 1.176791
C 0.653608 2.606503 0.901764
C -0.261847 2.011033 0.085922
C 0.248238 3.955163 1.404898
C -1.001306 4.279806 0.553037
C -1.337607 2.925929 -0.132201
O -2.397795 2.745180 -0.734245
O -2.076452 4.664202 1.396446
C -0.684687 5.300142 -0.512111
C -1.369433 6.416479 -0.718686
C -0.826996 -0.466612 0.421352
C -2.246360 -0.777571 -0.037476
C -2.860146 -1.972655 0.697182
C -2.099504 -3.286919 0.577812
C -1.775313 -3.738107 -0.846716
C -1.218966 -5.165738 -0.912618
C 0.052362 -5.408600 -0.090725
C 1.235972 -4.526627 -0.486233
C 2.496058 -4.819332 0.324525
O 1.178636 -1.355014 -2.067527
O 3.688236 -2.201223 -1.959004
O -3.170215 0.292692 0.204134
O -4.195410 -2.178299 0.210990
H 5.079719 -1.011164 -0.006855
H 3.556359 -1.598554 0.664541
H 4.324331 1.307395 0.066476
H 3.986830 0.614154 1.641146
H 3.836965 -0.143719 -1.995950
H -0.441703 0.535129 -1.449213
H 2.501565 2.478399 1.790927
H -0.033666 3.909432 2.458301
H 1.045665 4.690818 1.297319
H -2.892205 4.573868 0.883254
H 0.165933 5.052018 -1.140650
H -1.092464 7.102276 -1.508940
H -2.216492 6.686311 -0.100680

H -0.830675 -0.170703 1.473700
H -0.246309 -1.385910 0.348905
H -2.229046 -1.000082 -1.110927
H -2.911767 -1.700222 1.760359
H -1.184415 -3.206695 1.167698
H -2.706916 -4.052099 1.071425
H -2.683131 -3.685740 -1.452447
H -1.061743 -3.047537 -1.302520
H -1.994161 -5.861860 -0.574736
H -1.016589 -5.415231 -1.959593
H -0.160740 -5.265107 0.973705
H 0.341711 -6.459481 -0.198119
H 1.444060 -4.662986 -1.553083
H 0.971397 -3.473967 -0.368240
H 3.320847 -4.177571 0.010672
H 2.322059 -4.648776 1.390653
H 2.810479 -5.859580 0.203539
H 2.864333 -2.465043 -2.402945
H -2.934374 1.077707 -0.326181
H -4.573725 -1.291939 0.119965

2b-c352, ΔG = 1.0586 kcal/mol, population = 1.89 %
C 5.186034 1.042946 -0.096798
C 4.247021 2.208909 0.197348
C 2.828312 1.758396 0.373709
C 2.346027 0.563823 -0.077013
C 3.215057 -0.304001 -0.830760
C 4.601985 0.198808 -1.215917
C 0.929907 0.088247 0.209218
N 1.989029 2.654078 0.997278
C 0.644189 2.440038 1.031782
C 0.096888 1.271864 0.609846
C -0.343324 3.454559 1.517033
C -1.700817 2.830831 1.125683
C -1.334049 1.369253 0.721417
O -2.187221 0.506303 0.565837
O -2.573069 2.802350 2.245568
C -2.297491 3.522738 -0.073467
C -3.546679 3.961187 -0.151673
C 0.939737 -1.021081 1.294308
C -0.174077 -2.067877 1.194678
C -0.162872 -2.832763 -0.136290
C -1.234314 -3.914869 -0.224048
C -2.673153 -3.415951 -0.067495

C -3.102663 -2.389113 -1.115913
C -4.573673 -1.993159 -0.986683
C -5.007441 -0.874068 -1.937927
C -4.919880 -1.246851 -3.418335
O 2.884025 -1.436379 -1.208975
O 5.448031 -0.889901 -1.534544
O -0.048441 -2.982702 2.294607
O 1.109158 -3.475548 -0.320541
H 6.170376 1.412974 -0.382901
H 5.304497 0.420005 0.792924
H 4.273602 2.931978 -0.626068
H 4.566541 2.746707 1.092215
H 4.462709 0.834338 -2.104838
H 0.532003 -0.331454 -0.716089
H 2.368178 3.527852 1.332748
H -0.289296 3.561600 2.602000
H -0.175993 4.435804 1.071077
H -3.234825 2.117344 2.070145
H -1.623841 3.628018 -0.919227
H -3.916214 4.437299 -1.050785
H -4.232845 3.869209 0.680726
H 0.884974 -0.552549 2.278643
H 1.896896 -1.544544 1.254615
H -1.142134 -1.584392 1.311797
H -0.323039 -2.118271 -0.947265
H -1.122568 -4.402516 -1.196758
H -1.023858 -4.670355 0.536440
H -3.340869 -4.281805 -0.123523
H -2.813892 -2.993278 0.931975
H -2.496094 -1.485069 -1.020746
H -2.909848 -2.799218 -2.112351
H -5.205003 -2.874600 -1.151682
H -4.758859 -1.671401 0.043611
H -6.037490 -0.589183 -1.702222
H -4.392671 0.011624 -1.746724
H -3.891454 -1.449949 -3.723300
H -5.511081 -2.142361 -3.629997
H -5.297797 -0.440601 -4.050946
H 4.854196 -1.628631 -1.748479
H 0.711376 -3.546372 2.092511
H 1.741977 -2.797156 -0.616742

2b-c77, ΔG = 1.0630 kcal/mol, population = 1.88 %
C 2.989094 -0.372449 2.769932

C 2.676741 1.105903 2.561121
C 1.692728 1.319331 1.449477
C 1.438807 0.407672 0.470274
C 2.207158 -0.820081 0.438268
C 3.351372 -0.994009 1.433517
C 0.377341 0.605949 -0.602820
N 1.051450 2.543832 1.453148
C 0.213684 2.893055 0.446591
C -0.094581 2.031725 -0.560372
C -0.466590 4.222424 0.357282
C -1.178859 4.162566 -1.012393
C -1.030771 2.673249 -1.431823
O -1.687996 2.193420 -2.357124
O -2.555190 4.474117 -0.856380
C -0.485460 5.028691 -2.034312
C -1.085108 5.944910 -2.781890
C -0.812766 -0.387735 -0.444438
C -1.053307 -1.257252 -1.670414
C -2.284133 -2.156743 -1.509354
C -2.171897 -3.194295 -0.397735
C -0.941138 -4.100054 -0.478613
C -0.935263 -5.207660 0.580058
C -0.875567 -4.719332 2.032192
C 0.372146 -3.900974 2.366589
C 0.448795 -3.516026 3.842135
O 1.993222 -1.721820 -0.373109
O 3.678820 -2.364755 1.563387
O -1.209541 -0.493091 -2.878651
O -2.526699 -2.838281 -2.748674
H 3.811543 -0.490931 3.475274
H 2.116414 -0.886100 3.178746
H 3.593699 1.654842 2.317397
H 2.284020 1.549759 3.478149
H 4.210330 -0.447981 1.012319
H 0.838438 0.408589 -1.573873
H 1.238438 3.194423 2.202522
H -1.210389 4.331237 1.148535
H 0.240548 5.048511 0.440848
H -3.018092 4.115588 -1.627489
H 0.579679 4.843331 -2.139616
H -0.527651 6.528011 -3.503558
H -2.144143 6.149400 -2.688238
H -1.722288 0.171799 -0.207620
H -0.622927 -1.037723 0.409012

H -0.175455 -1.882776 -1.833416
H -3.143497 -1.505426 -1.293755
H -2.195930 -2.670917 0.560407
H -3.076995 -3.809286 -0.428076
H -0.905259 -4.556792 -1.470500
H -0.031938 -3.502933 -0.383460
H -1.828322 -5.828598 0.450435
H -0.076609 -5.862465 0.397183
H -1.766736 -4.128470 2.267604
H -0.911880 -5.591247 2.693988
H 1.262739 -4.474838 2.089392
H 0.398812 -2.994923 1.756623
H 1.359079 -2.954911 4.061792
H -0.404022 -2.895321 4.130164
H 0.442562 -4.403499 4.480544
H 3.282393 -2.799992 0.789454
H -1.506569 0.415913 -2.671673
H -2.365968 -2.173244 -3.434516

2b-c1, ΔG = 1.0931 kcal/mol, population = 1.78 %
C 4.527549 0.172334 1.887915
C 3.301499 0.996130 2.270900
C 2.245293 0.956689 1.206605
C 2.484212 0.627462 -0.092541
C 3.841946 0.352826 -0.507579
C 4.973758 0.570715 0.493412
C 1.376280 0.480716 -1.125758
N 0.973406 1.303563 1.623431
C -0.036344 1.438834 0.725414
C 0.125929 1.111691 -0.582598
C -1.400983 1.946101 1.073697
C -2.052603 2.155816 -0.311995
C -1.100608 1.388798 -1.279819
O -1.429805 1.096882 -2.422540
O -3.347145 1.582678 -0.354049
C -2.042818 3.614926 -0.697638
C -3.095403 4.283628 -1.148483
C 1.120180 -0.986817 -1.548381
C 0.934921 -1.963775 -0.393634
C 0.080582 -3.180931 -0.774881
C -1.388197 -2.865615 -1.044395
C -2.130716 -2.175642 0.099794
C -3.618469 -1.993152 -0.191109
C -4.383757 -1.367933 0.972637

C -5.850494 -1.085323 0.653721
C -6.600366 -0.440437 1.817977
O 4.131260 -0.034530 -1.641494
O 6.123189 -0.149472 0.087236
O 2.201055 -2.428880 0.124236
O 0.143595 -4.148877 0.280006
H 5.333630 0.337656 2.602846
H 4.280443 -0.891460 1.891242
H 3.585332 2.041718 2.437285
H 2.875017 0.637023 3.209877
H 5.191029 1.650557 0.485470
H 1.679061 1.009579 -2.034123
H 0.820640 1.532350 2.594630
H -1.962832 1.193285 1.630016
H -1.360770 2.853785 1.676640
H -3.525323 1.350641 -1.277119
H -1.079261 4.106588 -0.598394
H -3.019106 5.329170 -1.418304
H -4.064112 3.811131 -1.250420
H 1.947780 -1.329215 -2.172533
H 0.232431 -0.986505 -2.180424
H 0.468987 -1.470924 0.460319
H 0.513790 -3.619513 -1.685882
H -1.461665 -2.259734 -1.951125
H -1.883328 -3.813815 -1.274032
H -2.003248 -2.757984 1.015968
H -1.693530 -1.192506 0.293147
H -3.734435 -1.361964 -1.079085
H -4.063003 -2.963076 -0.442008
H -4.321976 -2.029559 1.844546
H -3.897289 -0.429322 1.251797
H -5.902825 -0.429360 -0.221674
H -6.347341 -2.018894 0.368317
H -7.645368 -0.247150 1.565554
H -6.584115 -1.085357 2.700715
H -6.142124 0.512416 2.095958
H 5.988050 -0.333901 -0.858041
H 2.773704 -2.647314 -0.622398
H 1.054366 -4.114614 0.608020

2b-c371, ΔG = 1.1019 kcal/mol, population = 1.76 %
C 4.253830 0.388274 2.655085
C 2.926973 1.128820 2.796153
C 2.028336 0.896619 1.619545

C 2.453023 0.439426 0.409794
C 3.872007 0.251887 0.185333
C 4.842064 0.677984 1.285764
C 1.479018 0.077785 -0.702484
N 0.697355 1.231938 1.809233
C -0.136325 1.339062 0.745181
C 0.221355 0.868477 -0.481684
C -1.466116 2.020030 0.783258
C -1.872428 2.087137 -0.707659
C -0.775310 1.254025 -1.428040
O -0.843228 0.978042 -2.627697
O -3.128570 1.444630 -0.885552
C -1.847005 3.502326 -1.223828
C -2.855030 4.103498 -1.840399
C 1.288181 -1.460280 -0.708221
C 0.381881 -2.063609 -1.795686
C -1.103278 -2.180853 -1.403849
C -1.337172 -3.212989 -0.310180
C -2.809028 -3.393425 0.074522
C -3.499343 -2.143579 0.629477
C -2.834046 -1.562928 1.879597
C -3.673630 -0.492287 2.585696
C -4.903173 -1.044592 3.307702
O 4.333299 -0.208975 -0.858886
O 6.089437 0.032861 1.109304
O 0.510499 -1.398754 -3.060848
O -1.854199 -2.577775 -2.559740
H 4.946948 0.702675 3.435187
H 4.097384 -0.688477 2.755559
H 3.103362 2.206902 2.887013
H 2.408232 0.822228 3.707133
H 4.967911 1.767097 1.178601
H 1.924178 0.360433 -1.658172
H 0.411328 1.591575 2.708745
H -2.196211 1.429288 1.335679
H -1.403991 3.002219 1.252844
H -3.232423 1.263804 -1.830556
H -0.907402 4.024456 -1.067155
H -2.764501 5.122315 -2.194350
H -3.802395 3.604490 -2.001145
H 0.926391 -1.766499 0.274378
H 2.284098 -1.889052 -0.821023
H 0.731745 -3.080243 -1.991045
H -1.466529 -1.205122 -1.065189

H -0.936544 -4.171012 -0.655587
H -0.762101 -2.932904 0.573723
H -3.361443 -3.748819 -0.798069
H -2.867921 -4.188767 0.824722
H -3.548708 -1.364485 -0.137405
H -4.535079 -2.408774 0.857880
H -2.619648 -2.371921 2.588182
H -1.864436 -1.135993 1.607661
H -3.044829 0.032369 3.311579
H -3.991429 0.256741 1.853010
H -5.595799 -1.528276 2.616418
H -4.612605 -1.784218 4.058583
H -5.450601 -0.248602 3.817039
H 6.105402 -0.248038 0.178807
H 0.141451 -0.493810 -2.983305
H -1.401512 -2.170755 -3.313421

2b-c31, ΔG = 1.2262 kcal/mol, population = 1.43 %
C 4.941220 0.117400 1.443644
C 3.879085 1.107585 1.911755
C 2.667385 1.090702 1.028364
C 2.654868 0.601568 -0.241801
C 3.891393 0.128864 -0.822575
C 5.188263 0.320682 -0.039867
C 1.382169 0.485410 -1.068506
N 1.524136 1.634987 1.583653
C 0.404911 1.809073 0.834766
C 0.316998 1.333587 -0.434339
C -0.819664 2.519060 1.320098
C -1.696494 2.613028 0.051647
C -0.970873 1.680145 -0.968627
O -1.497617 1.325788 -2.015896
O -2.996141 2.105030 0.313276
C -1.711186 4.014703 -0.502408
C -2.805151 4.684441 -0.838601
C 0.891375 -0.971743 -1.250965
C 0.654582 -1.740805 0.042440
C -0.314196 -2.918521 -0.133563
C -1.748893 -2.530018 -0.479657
C -2.415218 -1.551714 0.488761
C -3.873918 -1.245450 0.138056
C -4.828612 -2.429636 0.289832
C -6.287844 -2.061461 0.022825
C -7.241148 -3.245739 0.172050

O 3.951488 -0.406607 -1.931492
O 6.182684 -0.560876 -0.528453
O 1.886416 -2.239610 0.609295
O -0.340383 -3.686907 1.076620
H 5.865993 0.261178 2.002435
H 4.598399 -0.906280 1.609486
H 4.287214 2.124897 1.919811
H 3.574702 0.888037 2.937382
H 5.501741 1.363079 -0.208638
H 1.591475 0.860742 -2.074359
H 1.554989 1.976867 2.532924
H -1.336365 1.927952 2.078387
H -0.582082 3.491055 1.754148
H -3.356097 1.790215 -0.528651
H -0.729423 4.460818 -0.634851
H -2.744756 5.686824 -1.242692
H -3.791452 4.256988 -0.709050
H 1.610045 -1.518038 -1.865468
H -0.035935 -0.922225 -1.821423
H 0.258762 -1.081766 0.815740
H 0.077262 -3.548649 -0.945834
H -1.769996 -2.114088 -1.490150
H -2.321505 -3.459050 -0.524736
H -2.360165 -1.951324 1.505008
H -1.863563 -0.609614 0.497456
H -4.220480 -0.429269 0.778632
H -3.925056 -0.868435 -0.890353
H -4.539104 -3.237778 -0.388800
H -4.738575 -2.834664 1.304774
H -6.587251 -1.261021 0.707706
H -6.373942 -1.647503 -0.987572
H -7.197423 -3.658803 1.183359
H -8.275414 -2.955168 -0.024969
H -6.981207 -4.047597 -0.524107
H 5.878899 -0.822354 -1.414558
H 2.400613 -2.651986 -0.097018
H 0.572921 -3.693647 1.398780

2b-c106, ΔG = 1.3040 kcal/mol, population = 1.25 %
C 2.918126 -0.268102 2.839089
C 2.386731 1.158291 2.732052
C 1.443541 1.319367 1.578008
C 1.370339 0.461240 0.524990
C 2.307831 -0.641131 0.446895

C 3.429581 -0.711259 1.478940
C 0.310761 0.573574 -0.560709
N 0.641968 2.446461 1.612269
C -0.097971 2.799256 0.533909
C -0.235121 1.975107 -0.543565
C -0.806795 4.109771 0.408346
C -1.250688 4.121965 -1.071484
C -1.005719 2.658977 -1.534566
O -1.468361 2.233234 -2.595608
O -2.634745 4.420842 -1.164055
C -0.385689 5.044226 -1.894360
C -0.843625 5.988660 -2.704366
C -0.771815 -0.521502 -0.357939
C -1.412445 -1.054867 -1.639845
C -1.979788 -2.461430 -1.417511
C -0.888571 -3.527744 -1.356234
C -1.271528 -4.750056 -0.515023
C -1.209769 -4.497816 0.996902
C 0.213108 -4.396858 1.549531
C 0.260639 -4.021099 3.030302
C 1.673537 -4.061691 3.610264
O 2.252426 -1.508570 -0.425663
O 3.971442 -2.017484 1.520943
O -2.507342 -0.245493 -2.083432
O -2.880867 -2.805023 -2.478414
H 3.719652 -0.319666 3.575776
H 2.122046 -0.943747 3.159376
H 3.216336 1.862185 2.598817
H 1.877414 1.449862 3.653037
H 4.202174 -0.001689 1.143428
H 0.792489 0.380606 -1.523223
H 0.711641 3.073527 2.400867
H -1.688314 4.136818 1.051347
H -0.165177 4.950200 0.675159
H -2.937223 4.107946 -2.028949
H 0.682420 4.874249 -1.794040
H -0.166197 6.611270 -3.274424
H -1.904161 6.176998 -2.814327
H -1.559930 -0.146108 0.298932
H -0.299261 -1.353064 0.162724
H -0.658307 -1.107137 -2.435622
H -2.543025 -2.440802 -0.474580
H -0.680365 -3.824811 -2.386976
H 0.035685 -3.092633 -0.972637

H -2.279040 -5.071421 -0.794590
H -0.604459 -5.582235 -0.760350
H -1.758021 -3.581315 1.240919
H -1.731265 -5.306034 1.519139
H 0.719305 -5.357871 1.401841
H 0.790314 -3.660100 0.982450
H -0.160386 -3.017531 3.155505
H -0.388684 -4.697500 3.596545
H 2.082642 -5.074711 3.564788
H 2.347100 -3.408341 3.051938
H 1.686244 -3.744707 4.655743
H 3.679563 -2.446390 0.698453
H -2.152836 0.606886 -2.403957
H -3.339452 -1.979518 -2.696384

2b-c290, ΔG = 1.3485 kcal/mol, population = 1.16 %
C 2.964357 -0.931972 2.733776
C 1.786999 0.030746 2.842992
C 1.306955 0.487654 1.497764
C 2.059424 0.434360 0.360888
C 3.433036 -0.011198 0.453083
C 4.017071 -0.317862 1.830972
C 1.525315 0.844281 -1.007693
N 0.029567 1.006581 1.473571
C -0.480643 1.555707 0.334999
C 0.202743 1.532382 -0.831780
C -1.826112 2.202100 0.254106
C -2.016496 2.442630 -1.268696
C -0.585461 2.171842 -1.858106
O -0.265747 2.411481 -3.011943
O -2.906641 1.489809 -1.841339
C -2.485803 3.829822 -1.572140
C -3.491454 4.135264 -2.381439
C 1.436667 -0.322460 -2.026093
C 0.795911 -1.628312 -1.558289
C -0.659367 -1.528333 -1.104476
C -1.237138 -2.876923 -0.683386
C -2.679055 -2.802323 -0.171969
C -2.903509 -1.897522 1.043861
C -2.090334 -2.281647 2.279005
C -2.431464 -1.439912 3.507685
C -1.623848 -1.827275 4.744460
O 4.168886 -0.128784 -0.528925
O 5.146941 -1.160741 1.700332

O 0.807118 -2.572229 -2.646688
O -1.472598 -0.902574 -2.114261
H 3.383047 -1.131510 3.720143
H 2.635778 -1.882602 2.306931
H 2.074974 0.914985 3.422916
H 0.958711 -0.434275 3.379832
H 4.324004 0.651402 2.255398
H 2.236645 1.549638 -1.449065
H -0.502212 1.041964 2.330395
H -2.616299 1.557093 0.640707
H -1.843552 3.130293 0.829322
H -2.444397 0.631675 -1.926301
H -1.918525 4.615459 -1.082208
H -3.770308 5.165939 -2.560903
H -4.063866 3.363610 -2.878658
H 2.454524 -0.559342 -2.339930
H 0.899066 0.038577 -2.903033
H 1.367895 -2.036240 -0.717017
H -0.695414 -0.849631 -0.257878
H -1.190537 -3.560692 -1.533184
H -0.582385 -3.296086 0.085599
H -3.324259 -2.465066 -0.986695
H -3.003371 -3.817032 0.079158
H -2.692094 -0.856990 0.779782
H -3.967449 -1.924047 1.299567
H -2.262381 -3.339573 2.508683
H -1.020115 -2.188112 2.069019
H -2.269646 -0.380944 3.277599
H -3.500195 -1.537955 3.724075
H -0.550541 -1.723363 4.565436
H -1.879655 -1.202306 5.602509
H -1.809673 -2.868216 5.020880
H 5.446834 -1.046396 0.782520
H 1.663516 -2.518549 -3.086399
H -1.348765 -1.386525 -2.941532

2b-c28, ΔG = 1.3642 kcal/mol, population = 1.13 %
C 4.887838 0.324550 1.589326
C 3.703963 1.162404 2.063528
C 2.539588 1.073698 1.123075
C 2.635816 0.678460 -0.176359
C 3.941603 0.384843 -0.725237
C 5.174680 0.651645 0.135341
C 1.420375 0.487681 -1.072055

N 1.320681 1.451212 1.654433
C 0.221475 1.555298 0.863212
C 0.242737 1.174364 -0.440267
C -1.099336 2.071367 1.339848
C -1.924222 2.174750 0.037848
C -1.054478 1.412270 -1.010420
O -1.494249 1.081979 -2.104553
O -3.169190 1.510365 0.191158
C -2.068850 3.608021 -0.406135
C -3.213866 4.184383 -0.745653
C 1.105504 -1.003846 -1.340145
C 0.879613 -1.831560 -0.076063
C -0.233630 -2.877607 -0.228088
C -1.611280 -2.238172 -0.302991
C -2.747290 -3.240426 -0.508909
C -4.101729 -2.572368 -0.760964
C -4.625359 -1.745505 0.413696
C -5.979730 -1.095170 0.133869
C -6.485118 -0.245900 1.298801
O 4.108276 -0.050145 -1.866284
O 6.277296 -0.087419 -0.357163
O 2.091877 -2.476923 0.367880
O -0.204161 -3.761305 0.898605
H 5.766086 0.529733 2.201438
H 4.654811 -0.739198 1.675951
H 3.997010 2.215015 2.150822
H 3.382913 0.846376 3.058292
H 5.382735 1.729961 0.049215
H 1.631137 0.940649 -2.044554
H 1.271884 1.723527 2.625211
H -1.571148 1.353913 2.013881
H -1.003858 3.020744 1.868186
H -3.472866 1.262979 -0.694391
H -1.137047 4.164647 -0.453309
H -3.245258 5.217512 -1.067150
H -4.153178 3.647718 -0.701301
H 1.923767 -1.440737 -1.914930
H 0.223463 -1.039712 -1.979281
H 0.602176 -1.189471 0.762124
H -0.045968 -3.456479 -1.145451
H -1.768108 -1.664385 0.614496
H -1.626677 -1.520948 -1.126190
H -2.501064 -3.878691 -1.364089
H -2.817588 -3.900150 0.360121

H -4.022492 -1.927947 -1.644544
H -4.838732 -3.343339 -1.009355
H -4.707012 -2.388493 1.298266
H -3.908513 -0.960178 0.665885
H -5.897002 -0.470933 -0.762645
H -6.714510 -1.872285 -0.102529
H -6.598601 -0.849794 2.203158
H -5.783055 0.560780 1.523666
H -7.454431 0.205702 1.075891
H 6.045604 -0.311399 -1.274623
H 2.535823 -2.854553 -0.402137
H 0.734693 -3.884391 1.103779

2b-c114, ΔG = 1.3711 kcal/mol, population = 1.12 %
C 4.373103 0.518864 2.575757
C 2.996053 1.155924 2.740868
C 2.107339 0.882315 1.565327
C 2.556311 0.478871 0.344910
C 3.982856 0.394668 0.108322
C 4.927448 0.875594 1.207812
C 1.599653 0.063731 -0.763383
N 0.756341 1.118535 1.767655
C -0.084302 1.201383 0.706268
C 0.304225 0.785108 -0.530514
C -1.456735 1.792637 0.754086
C -1.832030 1.924706 -0.742296
C -0.706304 1.140508 -1.473513
O -0.763226 0.873117 -2.675496
O -3.078816 1.288569 -0.983458
C -1.801405 3.364377 -1.188520
C -2.791930 3.984904 -1.814158
C 1.498839 -1.483529 -0.767808
C 0.556836 -2.143035 -1.787508
C -0.910673 -2.257154 -1.339356
C -1.116790 -3.188619 -0.152895
C -2.541251 -3.165635 0.409577
C -2.923266 -1.827262 1.043121
C -4.294957 -1.837969 1.713668
C -4.673619 -0.487985 2.322928
C -6.043520 -0.496239 2.998320
O 4.467321 -0.021091 -0.944227
O 6.218217 0.329415 1.011689
O 0.623003 -1.523188 -3.081584
O -1.691304 -2.761247 -2.433580

H 5.047657 0.869934 3.356491
H 4.298323 -0.568197 2.657487
H 3.093119 2.241868 2.853319
H 2.508793 0.793532 3.648648
H 4.969700 1.972761 1.118113
H 2.020413 0.372935 -1.721857
H 0.448480 1.430034 2.678031
H -2.155931 1.115479 1.246167
H -1.474881 2.744035 1.285943
H -3.146719 1.134366 -1.936545
H -0.874357 3.887210 -0.971321
H -2.699281 5.020535 -2.114728
H -3.726760 3.485361 -2.035251
H 1.227359 -1.813306 0.235572
H 2.508406 -1.852081 -0.952495
H 0.914983 -3.161901 -1.956344
H -1.276925 -1.261295 -1.081258
H -0.852631 -4.201590 -0.470735
H -0.425810 -2.914930 0.647269
H -3.252731 -3.412881 -0.383181
H -2.629339 -3.954649 1.163087
H -2.161746 -1.552387 1.783313
H -2.913265 -1.040348 0.285182
H -5.055073 -2.126658 0.978525
H -4.316014 -2.607037 2.494477
H -3.908583 -0.197088 3.051204
H -4.655068 0.274755 1.537896
H -6.077081 -1.229885 3.808152
H -6.287128 0.480228 3.422745
H -6.829990 -0.755403 2.284553
H 6.246475 0.063564 0.077023
H 0.246297 -0.621028 -3.020758
H -1.299782 -2.375615 -3.231081

2b-c240, ΔG = 1.3824 kcal/mol, population = 1.09 %
C 3.130998 -0.317079 2.622307
C 2.966896 1.177326 2.359184
C 1.883648 1.454286 1.359534
C 1.418044 0.536052 0.468264
C 2.042538 -0.768739 0.412629
C 3.256790 -1.043590 1.296044
C 0.255125 0.804788 -0.475664
N 1.379438 2.741052 1.370766
C 0.463917 3.138329 0.452720

C -0.050854 2.273915 -0.462117
C -0.088047 4.526762 0.372645
C -0.964682 4.484274 -0.900256
C -1.017622 2.970891 -1.253107
O -1.825026 2.518976 -2.066555
O -2.273994 4.945268 -0.602540
C -0.317207 5.228950 -2.041175
C -0.906719 6.175942 -2.757825
C -1.006577 -0.031304 -0.103117
C -1.472338 -0.958708 -1.214810
C -2.734390 -1.739492 -0.840204
C -2.582801 -2.645556 0.377155
C -1.347907 -3.547843 0.377392
C -1.295402 -4.468905 1.597558
C 0.064963 -5.141623 1.801803
C 0.507495 -6.045347 0.650684
C 1.843970 -6.733171 0.924724
O 1.650900 -1.665613 -0.335656
O 3.411610 -2.439639 1.481145
O -1.719850 -0.264922 -2.451294
O -3.128878 -2.543942 -1.962744
H 4.013226 -0.498589 3.236095
H 2.260652 -0.702163 3.159205
H 3.903783 1.598717 1.977182
H 2.743042 1.708832 3.286408
H 4.127914 -0.642513 0.754104
H 0.558832 0.507373 -1.482397
H 1.726362 3.396961 2.055262
H -0.716063 4.744351 1.238362
H 0.700332 5.279010 0.328568
H -2.858748 4.610376 -1.297772
H 0.701533 4.923804 -2.262766
H -0.386429 6.666071 -3.570607
H -1.918875 6.498194 -2.548029
H -1.820380 0.644578 0.175178
H -0.788272 -0.626286 0.782954
H -0.675365 -1.669672 -1.433577
H -3.529975 -1.008907 -0.635847
H -2.572432 -2.018570 1.273129
H -3.488569 -3.256038 0.445891
H -1.331212 -4.137027 -0.542036
H -0.441663 -2.937149 0.366557
H -1.531888 -3.883066 2.492531
H -2.076968 -5.233017 1.516846

H 0.824684 -4.366208 1.955903
H 0.038360 -5.731872 2.724194
H -0.265688 -6.799866 0.468305
H 0.586779 -5.458631 -0.268372
H 2.635152 -5.995914 1.086432
H 1.787594 -7.358637 1.819638
H 2.146836 -7.369583 0.090367
H 2.928484 -2.853449 0.745678
H -1.890108 0.682030 -2.279786
H -3.025436 -1.969852 -2.735451

2b-c477, ΔG = 1.3912 kcal/mol, population = 1.08 %
C 2.536274 -0.541651 3.072199
C 2.373990 0.957795 2.839804
C 1.578602 1.248133 1.601372
C 1.410224 0.361761 0.581424
C 2.096306 -0.912243 0.637048
C 3.050144 -1.184692 1.797702
C 0.511111 0.628119 -0.617682
N 1.021451 2.511229 1.533231
C 0.345466 2.914916 0.429316
C 0.121838 2.077712 -0.619035
C -0.241971 4.281429 0.268088
C -0.783074 4.264942 -1.179878
C -0.662548 2.771439 -1.593678
O -1.223749 2.327824 -2.596745
O -2.150451 4.647304 -1.189135
C 0.073229 5.097925 -2.100414
C -0.382366 6.049902 -2.902989
C -0.744648 -0.294321 -0.607739
C -0.881193 -1.166792 -1.845769
C -2.136774 -2.044160 -1.798491
C -2.187750 -3.017102 -0.626250
C -0.986684 -3.959401 -0.491171
C -0.982793 -4.735525 0.830724
C -0.596224 -3.870268 2.034060
C -0.518739 -4.630622 3.361165
C -1.865043 -5.164868 3.850778
O 1.961148 -1.782194 -0.224518
O 3.232851 -2.580234 1.954827
O -0.907297 -0.410322 -3.069017
O -2.231187 -2.787917 -3.022849
H 3.229851 -0.724815 3.892821
H 1.574489 -0.988319 3.335760

H 3.356720 1.432489 2.738724
H 1.888532 1.429123 3.696670
H 4.008320 -0.714146 1.525858
H 1.077252 0.403571 -1.525265
H 1.143326 3.145166 2.309741
H -1.069706 4.432906 0.963120
H 0.495688 5.065277 0.443696
H -2.533135 4.318638 -2.015554
H 1.131853 4.855336 -2.079532
H 0.288109 6.606018 -3.545447
H -1.432501 6.311495 -2.933710
H -1.644512 0.315901 -0.487424
H -0.695350 -0.941656 0.266565
H -0.004174 -1.810147 -1.917809
H -3.003624 -1.371431 -1.728250
H -2.309544 -2.430932 0.286681
H -3.102051 -3.610504 -0.723523
H -0.989240 -4.656329 -1.332221
H -0.052996 -3.396304 -0.556917
H -1.965666 -5.190590 0.988598
H -0.269086 -5.563020 0.758337
H 0.376631 -3.413021 1.829831
H -1.305960 -3.043398 2.145315
H 0.189855 -5.459967 3.259565
H -0.101459 -3.964630 4.122614
H -2.592288 -4.353673 3.946261
H -2.281863 -5.903102 3.163146
H -1.768282 -5.643039 4.827942
H 2.974709 -2.972102 1.103233
H -1.146768 0.520306 -2.889531
H -2.013770 -2.154106 -3.721700

2b-c76, ΔG = 1.4182 kcal/mol, population = 1.03 %
C 4.997086 0.193888 1.523732
C 3.893472 1.151838 1.963570
C 2.715523 1.115607 1.037050
C 2.764570 0.651495 -0.242428
C 4.034349 0.220083 -0.784938
C 5.294813 0.422375 0.053112
C 1.528885 0.529683 -1.121716
N 1.539419 1.625121 1.554959
C 0.450489 1.798254 0.761420
C 0.428795 1.359397 -0.523507
C -0.811340 2.462398 1.214268

C -1.636549 2.573013 -0.086731
C -0.839011 1.700412 -1.106056
O -1.306513 1.375337 -2.190299
O -2.926669 2.008698 0.101239
C -1.680119 3.992563 -0.590801
C -2.782885 4.638318 -0.944978
C 1.068495 -0.936673 -1.305072
C 0.799435 -1.676800 0.005507
C -0.452807 -2.563315 -0.045937
C -1.732299 -1.741298 -0.073997
C -3.005257 -2.577889 -0.180098
C -4.263579 -1.709221 -0.217776
C -5.567124 -2.490860 -0.398479
C -5.917483 -3.418070 0.766691
C -7.272425 -4.101623 0.590347
O 4.150310 -0.282238 -1.904219
O 6.325164 -0.431609 -0.408799
O 1.934431 -2.465757 0.420630
O -0.480835 -3.405251 1.112176
H 5.896257 0.353728 2.118753
H 4.676075 -0.840685 1.665514
H 4.274795 2.179003 1.993810
H 3.559974 0.915473 2.975956
H 5.592662 1.473467 -0.088568
H 1.773078 0.906572 -2.118440
H 1.522331 1.945515 2.512113
H -1.342268 1.832853 1.930826
H -0.620236 3.426300 1.687699
H -3.252389 1.746254 -0.772086
H -0.710363 4.475932 -0.670387
H -2.740783 5.656041 -1.311302
H -3.759131 4.176397 -0.868620
H 1.828463 -1.476538 -1.872215
H 0.171100 -0.922597 -1.923638
H 0.658363 -0.972188 0.827012
H -0.398470 -3.191654 -0.948127
H -1.765236 -1.129170 0.833502
H -1.694111 -1.048683 -0.916928
H -2.962013 -3.188557 -1.089449
H -3.050538 -3.272838 0.660068
H -4.321109 -1.118798 0.703769
H -4.166237 -0.987300 -1.035577
H -6.389135 -1.780560 -0.538361
H -5.511213 -3.078906 -1.322219

H -5.142104 -4.180339 0.881377
H -5.917229 -2.837755 1.695975
H -8.076138 -3.364548 0.512571
H -7.289909 -4.706764 -0.320103
H -7.502279 -4.759094 1.431672
H 6.064298 -0.682074 -1.311449
H 2.276295 -2.937151 -0.349730
H 0.443258 -3.644525 1.277999

2b-c9, ΔG = 1.4226 kcal/mol, population = 1.02 %
C 4.623495 0.157529 1.772427
C 3.425498 1.007851 2.185454
C 2.333563 0.975721 1.158098
C 2.519632 0.620054 -0.142728
C 3.856363 0.312579 -0.600177
C 5.026364 0.527439 0.356872
C 1.371765 0.477498 -1.132005
N 1.085292 1.359553 1.611873
C 0.050028 1.508921 0.745919
C 0.159084 1.155539 -0.560821
C -1.288515 2.057183 1.131614
C -1.985662 2.253040 -0.233683
C -1.083360 1.451236 -1.220783
O -1.458043 1.149018 -2.346709
O -3.289197 1.698696 -0.217703
C -1.968102 3.704339 -0.647044
C -3.026946 4.381067 -1.070444
C 1.061061 -0.992935 -1.503905
C 0.864036 -1.925697 -0.314579
C -0.037400 -3.124394 -0.643539
C -1.502918 -2.767987 -0.879282
C -2.190538 -2.037774 0.274387
C -3.687994 -1.846883 0.042852
C -4.386745 -1.178491 1.226620
C -5.851652 -0.815017 0.970955
C -6.763819 -2.019386 0.735141
O 4.098647 -0.098986 -1.736564
O 6.149229 -0.217461 -0.077742
O 2.123069 -2.415039 0.197746
O 0.023435 -4.069756 0.431625
H 5.456999 0.319795 2.455870
H 4.359319 -0.901892 1.801152
H 3.733343 2.049822 2.330214
H 3.023709 0.667057 3.142064

H 5.259898 1.603477 0.324088
H 1.655470 0.973652 -2.064527
H 0.971938 1.610778 2.582832
H -1.846732 1.330255 1.724549
H -1.205915 2.976687 1.712133
H -3.506755 1.459020 -1.130349
H -0.994009 4.182188 -0.593878
H -2.945594 5.419970 -1.363324
H -4.005706 3.921615 -1.125622
H 1.866098 -1.382367 -2.130371
H 0.163029 -0.983727 -2.121394
H 0.428951 -1.390177 0.529616
H 0.357038 -3.597075 -1.555159
H -1.584291 -2.174967 -1.793510
H -2.032182 -3.704615 -1.078442
H -2.031388 -2.597231 1.199955
H -1.735099 -1.055004 0.422695
H -3.839142 -1.236546 -0.854795
H -4.145329 -2.819153 -0.164551
H -4.323313 -1.837214 2.100777
H -3.840726 -0.265882 1.477036
H -6.227649 -0.245385 1.826474
H -5.904113 -0.141173 0.109056
H -6.480753 -2.571452 -0.163299
H -6.716867 -2.713720 1.578860
H -7.804417 -1.709258 0.616439
H 5.977932 -0.414499 -1.014574
H 2.675277 -2.676903 -0.550184
H 0.941050 -4.050292 0.741501

2b-c280, ΔG = 1.5161 kcal/mol, population = 0.87 %
C 3.182454 -0.248237 2.623974
C 2.778564 1.219392 2.505517
C 1.710682 1.426185 1.473785
C 1.417828 0.534642 0.489082
C 2.229572 -0.657360 0.351589
C 3.464044 -0.800256 1.237329
C 0.243129 0.705452 -0.461365
N 1.030429 2.628495 1.547751
C 0.177122 3.000757 0.563333
C -0.177039 2.147994 -0.439257
C -0.439527 4.359996 0.468020
C -1.097242 4.344720 -0.930618
C -1.032573 2.848505 -1.344739

O -1.673348 2.415160 -2.305013
O -2.453674 4.751485 -0.836249
C -0.299105 5.158671 -1.918476
C -0.799628 6.109370 -2.695294
C -0.903237 -0.277675 -0.086307
C -1.638405 -0.892954 -1.278041
C -2.273415 -2.241518 -0.916823
C -1.238466 -3.346950 -0.775515
C -1.778357 -4.614080 -0.116851
C -0.714479 -5.699859 0.082602
C 0.510085 -5.269789 0.900815
C 0.183319 -4.721269 2.290196
C 1.433923 -4.397650 3.105916
O 1.980658 -1.547342 -0.462337
O 3.873296 -2.153941 1.284612
O -2.707652 -0.063489 -1.747307
O -3.213277 -2.624156 -1.928547
H 4.066422 -0.347255 3.253710
H 2.375754 -0.826232 3.081115
H 3.646767 1.828522 2.228938
H 2.425689 1.600712 3.466170
H 4.253077 -0.191838 0.767997
H 0.584104 0.439413 -1.465749
H 1.263390 3.278418 2.284639
H -1.210307 4.490903 1.229576
H 0.297718 5.154157 0.590185
H -2.904169 4.431988 -1.631448
H 0.755030 4.901895 -1.971091
H -0.170395 6.650802 -3.389900
H -1.845983 6.384511 -2.653649
H -1.629810 0.227477 0.554350
H -0.467359 -1.078827 0.508277
H -0.927122 -1.060279 -2.097294
H -2.807947 -2.116023 0.036629
H -0.847571 -3.576406 -1.772197
H -0.396581 -2.967313 -0.196740
H -2.218307 -4.349702 0.849792
H -2.593230 -5.020995 -0.720909
H -1.182595 -6.560677 0.571525
H -0.375383 -6.051562 -0.897593
H 1.175378 -6.133119 1.008418
H 1.083386 -4.518140 0.349087
H -0.425083 -3.817219 2.195685
H -0.433339 -5.448825 2.829428

H 2.041910 -5.292746 3.263357
H 2.056707 -3.660655 2.593573
H 1.176417 -3.993296 4.087650
H 3.440200 -2.581773 0.526200
H -2.335306 0.780678 -2.071649
H -3.625672 -1.796437 -2.217648

2b-c111, ΔG = 1.5707 kcal/mol, population = 0.80 %
C 2.850512 -0.236691 2.927385
C 2.656944 1.245854 2.622927
C 1.683145 1.467741 1.504156
C 1.340353 0.518707 0.590023
C 1.998532 -0.770916 0.636968
C 3.137633 -0.975230 1.632708
C 0.287553 0.737963 -0.487340
N 1.150047 2.740760 1.428910
C 0.337812 3.095757 0.403765
C -0.050000 2.200887 -0.544726
C -0.229505 4.469151 0.231260
C -0.944127 4.384328 -1.136092
C -0.930030 2.863477 -1.457803
O -1.630500 2.383391 -2.350652
O -2.286788 4.827801 -1.011500
C -0.172823 5.116045 -2.206290
C -0.685332 6.032656 -3.015608
C -0.990363 -0.120736 -0.245776
C -1.332485 -1.053837 -1.398637
C -2.653330 -1.796698 -1.172092
C -2.675727 -2.703521 0.052854
C -1.514216 -3.691654 0.159781
C -1.638221 -4.606880 1.377874
C -0.492013 -5.612309 1.520541
C 0.878770 -4.970297 1.742424
C 1.984815 -5.994587 1.987009
O 1.697941 -1.703347 -0.109545
O 3.345744 -2.357490 1.856334
O -1.407990 -0.378603 -2.666256
O -2.943384 -2.589433 -2.333184
H 3.673177 -0.374827 3.628916
H 1.946449 -0.649800 3.381302
H 3.613270 1.700546 2.339901
H 2.310560 1.779749 3.510359
H 4.034432 -0.536656 1.167396
H 0.713597 0.426803 -1.444310

H 1.402568 3.419787 2.131977
H -0.962057 4.685424 1.010871
H 0.542257 5.238790 0.268288
H -2.778181 4.461672 -1.761140
H 0.871322 4.828361 -2.290246
H -0.076211 6.515122 -3.769114
H -1.721762 6.337314 -2.942858
H -1.837392 0.542278 -0.046564
H -0.852168 -0.718927 0.654287
H -0.530280 -1.784041 -1.508973
H -3.441730 -1.038647 -1.059311
H -2.702524 -2.073835 0.946583
H -3.624229 -3.249336 0.039148
H -1.466153 -4.299354 -0.748768
H -0.572309 -3.143491 0.215824
H -1.689551 -3.992551 2.284725
H -2.587551 -5.149969 1.322182
H -0.707927 -6.281195 2.360627
H -0.452295 -6.246167 0.626876
H 1.140145 -4.356646 0.876850
H 0.818625 -4.284009 2.593918
H 1.769719 -6.602706 2.869697
H 2.086009 -6.672192 1.135014
H 2.950701 -5.509252 2.143550
H 2.914868 -2.809238 1.110947
H -1.622835 0.566544 -2.534967
H -2.720402 -2.023119 -3.086486

2b-c12, ΔG = 1.5970 kcal/mol, population = 0.76 %
C 5.012285 0.233474 1.063884
C 3.933968 1.051126 1.768693
C 2.621090 0.986482 1.048040
C 2.483136 0.623054 -0.256364
C 3.667583 0.348995 -1.037613
C 5.034449 0.605934 -0.407410
C 1.125737 0.439303 -0.917757
N 1.515758 1.347980 1.796155
C 0.301692 1.499587 1.206210
C 0.091986 1.144598 -0.088031
C -0.899968 2.060733 1.899302
C -1.919431 2.228214 0.750443
C -1.264372 1.460169 -0.440660
O -1.881882 1.191263 -1.463403
O -3.150797 1.610766 1.094925

C -2.072591 3.676727 0.362196
C -3.230818 4.312030 0.246497
C 0.741512 -1.045452 -1.141344
C 0.742805 -1.910497 0.112833
C -0.106481 -3.181525 -0.027795
C -1.602103 -2.939767 -0.207173
C -2.241634 -2.036813 0.848679
C -3.769282 -1.986565 0.772139
C -4.311538 -1.476220 -0.563811
C -5.812509 -1.192375 -0.537516
C -6.346112 -0.683527 -1.875341
O 3.626781 -0.067645 -2.197334
O 6.033297 -0.109113 -1.111487
O 2.078181 -2.296240 0.507298
O 0.077755 -3.993261 1.139773
H 5.986592 0.422408 1.514586
H 4.794388 -0.832774 1.157055
H 4.240291 2.101357 1.837721
H 3.794932 0.701078 2.793834
H 5.219928 1.687900 -0.497421
H 1.163806 0.893197 -1.911672
H 1.641393 1.600303 2.765629
H -1.289068 1.352638 2.633120
H -0.676745 2.994881 2.416153
H -3.617225 1.422081 0.267883
H -1.139012 4.192223 0.154881
H -3.269910 5.352969 -0.047723
H -4.171275 3.817519 0.454476
H 1.416697 -1.482718 -1.880252
H -0.254478 -1.050117 -1.584901
H 0.371487 -1.344752 0.967801
H 0.262870 -3.733039 -0.904859
H -1.771692 -2.533069 -1.205678
H -2.089515 -3.919619 -0.193245
H -1.940636 -2.380896 1.841040
H -1.860997 -1.017960 0.744227
H -4.179925 -2.982648 0.973255
H -4.133322 -1.334804 1.572631
H -3.774186 -0.565255 -0.848695
H -4.101477 -2.204519 -1.353351
H -6.347702 -2.103838 -0.250420
H -6.023945 -0.455426 0.244834
H -7.418556 -0.481819 -1.829504
H -5.844045 0.242317 -2.168667

H -6.177670 -1.416255 -2.668985
H 5.636047 -0.320004 -1.973799
H 2.540029 -2.634031 -0.271170
H 1.015430 -3.911575 1.367564

2b-c454, ΔG = 1.6240 kcal/mol, population = 0.73 %
C 5.117213 0.043089 0.751629
C 4.633660 1.485414 0.628917
C 3.143007 1.567974 0.497157
C 2.354767 0.525058 0.107218
C 2.969010 -0.723490 -0.267809
C 4.489787 -0.795641 -0.348729
C 0.837871 0.605756 0.091370
N 2.581869 2.798997 0.757733
C 1.273893 3.042905 0.465407
C 0.433482 2.050465 0.076799
C 0.649808 4.400935 0.525883
C -0.734461 4.176960 -0.122952
C -0.848112 2.623806 -0.232804
O -1.897803 2.065512 -0.524473
O -1.758608 4.663064 0.732808
C -0.788905 4.766586 -1.508941
C -1.726731 5.596681 -1.944559
C 0.251343 -0.158328 1.306456
C -1.150568 -0.744987 1.123001
C -1.250333 -1.700653 -0.074878
C -2.637158 -2.308415 -0.220954
C -2.805271 -3.121334 -1.503203
C -4.222049 -3.669623 -1.706922
C -4.735701 -4.585754 -0.590387
C -3.874321 -5.824409 -0.343006
C -4.467944 -6.751871 0.715912
O 2.320530 -1.739808 -0.552355
O 4.922406 -2.141334 -0.279503
O -1.528087 -1.417825 2.335103
O -0.298535 -2.766654 0.067112
H 6.203748 0.001359 0.677988
H 4.828380 -0.370283 1.720915
H 5.080349 1.959812 -0.252535
H 4.951428 2.074036 1.491957
H 4.766262 -0.365393 -1.324384
H 0.493246 0.126514 -0.826011
H 3.179669 3.557868 1.051523
H 0.521123 4.723777 1.560727

H 1.249785 5.151360 0.009869
H -2.582863 4.232833 0.462792
H 0.012572 4.447181 -2.169204
H -1.709801 5.975559 -2.958361
H -2.530064 5.932688 -1.301121
H 0.238664 0.507864 2.171066
H 0.923860 -0.980174 1.559834
H -1.878573 0.053940 0.983947
H -1.031343 -1.135369 -0.986454
H -2.839280 -2.927189 0.655165
H -3.368988 -1.494133 -0.204241
H -2.550167 -2.488900 -2.360338
H -2.082588 -3.939662 -1.504063
H -4.915480 -2.828938 -1.817751
H -4.252033 -4.218284 -2.654464
H -4.824851 -4.020993 0.343260
H -5.751446 -4.907500 -0.844024
H -3.751865 -6.370858 -1.284662
H -2.870698 -5.520206 -0.033457
H -5.459808 -7.102803 0.418775
H -3.838137 -7.629047 0.879764
H -4.573838 -6.233909 1.672958
H 4.153262 -2.677440 -0.534401
H -1.016490 -2.238518 2.361138
H 0.584191 -2.420276 -0.153992

2b-c287, ΔG = 1.6673 kcal/mol, population = 0.68 %
C 4.016085 -0.792569 -0.676129
C 3.909343 0.697441 -0.360010
C 2.493526 1.118492 -0.098310
C 1.403906 0.406003 -0.496996
C 1.592978 -0.790377 -1.290605
C 3.003439 -1.147845 -1.749745
C -0.020414 0.793870 -0.133462
N 2.340492 2.324835 0.562200
C 1.110659 2.872352 0.726238
C -0.016765 2.206792 0.360143
C 0.860264 4.223748 1.318703
C -0.662081 4.421875 1.119159
C -1.150413 3.023967 0.647316
O -2.344445 2.724843 0.583578
O -1.270568 4.742342 2.361886
C -0.948697 5.432180 0.037200
C -1.740722 6.486563 0.174738

C -0.640812 -0.186563 0.909978
C -1.902225 -0.875454 0.409904
C -2.343072 -2.028205 1.324066
C -3.289093 -3.013765 0.631825
C -2.717489 -3.675500 -0.629790
C -1.346656 -4.327743 -0.442317
C -0.816341 -4.989753 -1.712017
C 0.579536 -5.587356 -1.538823
C 1.134422 -6.200242 -2.823126
O 0.661070 -1.518481 -1.634179
O 3.065647 -2.520199 -2.094211
O -3.024914 0.017108 0.297463
O -2.943232 -1.495807 2.512353
H 5.023505 -1.035154 -1.014025
H 3.808836 -1.383460 0.219346
H 4.293821 1.289202 -1.198645
H 4.524557 0.951024 0.505854
H 3.202444 -0.532280 -2.641219
H -0.626692 0.730594 -1.042089
H 3.162767 2.828889 0.861389
H 1.089497 4.229775 2.385750
H 1.460454 4.997922 0.839427
H -2.218589 4.568884 2.272289
H -0.458679 5.232063 -0.911392
H -1.909388 7.168418 -0.648768
H -2.233861 6.708641 1.112682
H -0.846870 0.341127 1.844480
H 0.094376 -0.957570 1.144379
H -1.677876 -1.279073 -0.578424
H -1.453807 -2.563040 1.658186
H -3.550280 -3.782799 1.364779
H -4.215106 -2.488115 0.380936
H -3.429682 -4.430666 -0.976812
H -2.649740 -2.937904 -1.434993
H -0.618371 -3.576413 -0.126587
H -1.401038 -5.067509 0.364845
H -1.510060 -5.772529 -2.039874
H -0.788317 -4.244946 -2.515223
H 1.259685 -4.806163 -1.182973
H 0.551377 -6.347106 -0.750631
H 0.487729 -7.003632 -3.185757
H 1.205004 -5.449904 -3.615178
H 2.131856 -6.617836 -2.669747
H 2.145434 -2.784522 -2.265107

H -2.739085 0.949869 0.361255
H -3.486121 -0.755180 2.199219

2b-c2, ΔG = 1.7031 kcal/mol, population = 0.64 %
C 4.479083 0.297339 1.848171
C 3.232800 1.099878 2.208960
C 2.177596 1.003560 1.147307
C 2.425256 0.644937 -0.142672
C 3.789856 0.396519 -0.551346
C 4.915680 0.669042 0.443183
C 1.321819 0.442931 -1.171681
N 0.897843 1.330523 1.554783
C -0.113398 1.417733 0.652206
C 0.056561 1.058192 -0.645447
C -1.494113 1.895395 0.984101
C -2.131020 2.080912 -0.402409
C -1.177826 1.291471 -1.347080
O -1.510503 0.956332 -2.477379
O -3.437740 1.511578 -0.437383
C -2.176547 3.507321 -0.900438
C -1.536139 4.555637 -0.399943
C 1.100306 -1.041598 -1.551707
C 0.920213 -1.985320 -0.368507
C 0.085147 -3.225494 -0.717481
C -1.387058 -2.938629 -1.000163
C -2.143389 -2.233450 0.125656
C -3.632470 -2.075888 -0.173359
C -4.405589 -1.425794 0.971649
C -5.875439 -1.168299 0.645929
C -6.629393 -0.492865 1.790039
O 4.089918 -0.010379 -1.675655
O 6.082689 -0.033046 0.055970
O 2.189650 -2.416043 0.170619
O 0.157745 -4.161239 0.365335
H 5.280487 0.502071 2.558146
H 4.258958 -0.771926 1.880319
H 3.490023 2.156657 2.345143
H 2.816806 0.756634 3.158509
H 5.106076 1.753450 0.405601
H 1.613132 0.952994 -2.094495
H 0.739225 1.585314 2.518506
H -2.041913 1.122518 1.526400
H -1.491674 2.794135 1.598771
H -3.599486 1.229864 -1.350004

H -2.786662 3.623154 -1.791705
H -1.625652 5.530842 -0.861427
H -0.897537 4.492806 0.472577
H 1.942265 -1.387520 -2.154417
H 0.221053 -1.078396 -2.194684
H 0.441826 -1.473830 0.467242
H 0.528659 -3.683336 -1.613886
H -1.466038 -2.354121 -1.920300
H -1.867680 -3.898822 -1.210047
H -2.012453 -2.793749 1.054970
H -1.718739 -1.241009 0.298895
H -3.754094 -1.471788 -1.079239
H -4.065878 -3.057375 -0.397124
H -4.335836 -2.060465 1.862739
H -3.930136 -0.473774 1.223000
H -5.935274 -0.541723 -0.250243
H -6.363615 -2.115401 0.391882
H -7.676554 -0.317443 1.533580
H -6.605654 -1.108429 2.693313
H -6.179363 0.472663 2.035802
H 5.953518 -0.243487 -0.884699
H 2.768589 -2.650683 -0.566245
H 1.065999 -4.102721 0.696920

2b-c587, ΔG = 1.7369 kcal/mol, population = 0.60 %
C 5.005677 0.445734 -0.798988
C 4.306108 1.791095 -0.625012
C 2.895812 1.632773 -0.142520
C 2.181273 0.475330 -0.248573
C 2.767327 -0.657170 -0.919296
C 4.116969 -0.484247 -1.606715
C 0.792741 0.312658 0.346583
N 2.313244 2.759000 0.395623
C 0.978392 2.789990 0.665981
C 0.209477 1.674559 0.585209
C 0.238422 4.030868 1.054348
C -1.240381 3.587065 0.992569
C -1.157720 2.034981 0.846917
O -2.141062 1.315407 0.964173
O -1.899155 3.911075 2.208276
C -1.928050 4.151347 -0.224399
C -3.071700 4.822334 -0.208653
C 0.862789 -0.535375 1.643219
C -0.390560 -1.344112 1.989486

C -0.808356 -2.302412 0.865232
C -2.033827 -3.129619 1.228372
C -2.547810 -4.017989 0.093347
C -3.045283 -3.252614 -1.133628
C -3.647300 -4.169572 -2.198552
C -4.076083 -3.452298 -3.482694
C -5.204351 -2.439849 -3.282819
O 2.216103 -1.764420 -0.988925
O 4.733981 -1.742804 -1.802296
O -0.149733 -2.065311 3.208203
O 0.269553 -3.197151 0.552718
H 5.961782 0.579907 -1.304530
H 5.200153 -0.007342 0.176030
H 4.280261 2.329379 -1.579519
H 4.855647 2.425084 0.073825
H 3.907483 -0.017256 -2.582192
H 0.181542 -0.215787 -0.386327
H 2.860803 3.604312 0.468298
H 0.488639 4.328205 2.074450
H 0.463925 4.868124 0.392876
H -2.689789 3.354606 2.260194
H -1.419037 3.956436 -1.164231
H -3.514470 5.191334 -1.124869
H -3.593456 5.034175 0.716111
H 1.097495 0.120745 2.483337
H 1.695199 -1.236709 1.556732
H -1.225832 -0.675069 2.195644
H -1.048063 -1.701094 -0.015289
H -1.793652 -3.745648 2.097846
H -2.823969 -2.439382 1.540987
H -1.757057 -4.710531 -0.207589
H -3.366981 -4.632707 0.480807
H -3.785700 -2.512373 -0.814536
H -2.221076 -2.688574 -1.582724
H -2.913326 -4.941947 -2.452062
H -4.511214 -4.695291 -1.774977
H -3.206731 -2.948365 -3.919151
H -4.394062 -4.201203 -4.214373
H -5.520640 -2.009418 -4.235481
H -6.076425 -2.915220 -2.825034
H -4.898473 -1.616157 -2.635100
H 4.015720 -2.395893 -1.764094
H 0.453572 -2.787281 2.983167
H 0.927153 -2.709470 0.025338

2b-c261, ΔG = 1.7646 kcal/mol, population = 0.57 %

C 2.983578 -0.773957 2.746894
C 1.785125 0.165509 2.826421
C 1.296540 0.569782 1.467501
C 2.050437 0.497387 0.333013
C 3.433696 0.085948 0.438748
C 4.023638 -0.165427 1.825513
C 1.505487 0.845306 -1.048246
N 0.007062 1.056850 1.426612
C -0.515410 1.557053 0.271156
C 0.168521 1.510477 -0.894772
C -1.875081 2.170138 0.169437
C -2.071165 2.353714 -1.360578
C -0.634337 2.095752 -1.941845
O -0.320550 2.302038 -3.103751
O -2.939534 1.361882 -1.899468
C -2.571883 3.718711 -1.711078
C -3.582098 3.973629 -2.532150
C 1.440484 -0.361667 -2.020486
C 0.826545 -1.661138 -1.501131
C -0.633483 -1.575085 -1.059496
C -1.184563 -2.917876 -0.586992
C -2.637262 -2.857224 -0.104960
C -2.909127 -1.889980 1.051196
C -2.112586 -2.178980 2.322649
C -2.504648 -1.277805 3.492860
C -1.715905 -1.570684 4.767191
O 4.172015 -0.046546 -0.539539
O 5.170951 -0.988168 1.720511
O 0.865598 -2.648706 -2.549379
O -1.453468 -1.005997 -2.096943
H 3.405755 -0.934160 3.738939
H 2.676110 -1.744153 2.349202
H 2.052191 1.072971 3.379756
H 0.966203 -0.301603 3.375897
H 4.310384 0.822451 2.220024
H 2.200427 1.548684 -1.517847
H -0.524323 1.108359 2.282796
H -2.650683 1.522235 0.579838
H -1.912505 3.117348 0.711729
H -2.456829 0.513168 -1.962000
H -2.024392 4.533339 -1.246628
H -3.883974 4.991094 -2.746435

H -4.135500 3.172991 -3.004509
H 2.463036 -0.589771 -2.325384
H 0.895439 -0.047008 -2.910559
H 1.402531 -2.020636 -0.640760
H -0.690050 -0.865425 -0.239904
H -1.104521 -3.639924 -1.401817
H -0.533936 -3.281573 0.213280
H -3.277155 -2.586887 -0.948271
H -2.936820 -3.864915 0.199571
H -2.717833 -0.861873 0.729857
H -3.977107 -1.929364 1.287800
H -2.260165 -3.226409 2.610108
H -1.040899 -2.066449 2.130025
H -2.366250 -0.229681 3.205210
H -3.575113 -1.393429 3.691127
H -1.879616 -2.599066 5.099023
H -0.642065 -1.446305 4.605250
H -2.008413 -0.905306 5.582040
H 5.467565 -0.897595 0.799010
H 1.725557 -2.597687 -2.982419
H -1.312512 -1.516027 -2.905555

2b-c490, ΔG = 1.8217 kcal/mol, population = 0.52 %
C 5.341265 0.642361 0.112501
C 4.571043 1.954017 0.239939
C 3.092698 1.730074 0.341937
C 2.469833 0.580453 -0.046738
C 3.242127 -0.472036 -0.656972
C 4.710322 -0.212622 -0.973450
C 0.986659 0.339327 0.172733
N 2.353311 2.788955 0.822780
C 0.992774 2.775453 0.751276
C 0.310417 1.662363 0.380030
C 0.132370 3.962855 1.049098
C -1.264329 3.507546 0.570277
C -1.091080 1.976807 0.319493
O -2.045602 1.235429 0.124494
O -2.225077 3.711752 1.595810
C -1.636009 4.170553 -0.731682
C -2.771378 4.817680 -0.956722
C 0.775654 -0.627567 1.365593
C -0.504957 -1.465099 1.335067
C -0.646875 -2.295559 0.051802
C -1.900518 -3.157443 0.053351

C -2.191479 -3.797893 -1.302337
C -3.408516 -4.729404 -1.297716
C -4.728846 -4.079815 -0.868132
C -5.157991 -2.890438 -1.727880
C -6.517547 -2.327020 -1.317997
O 2.764440 -1.573526 -0.961863
O 5.403590 -1.436316 -1.131203
O -0.527442 -2.315292 2.492816
O 0.496308 -3.148346 -0.115686
H 6.385560 0.839838 -0.128651
H 5.311278 0.094193 1.057156
H 4.759105 2.589404 -0.633185
H 4.910143 2.517129 1.111891
H 4.725397 0.348357 -1.921277
H 0.592581 -0.126086 -0.731538
H 2.833366 3.630302 1.108388
H 0.103280 4.161577 2.122062
H 0.486092 4.864069 0.547141
H -2.977349 3.134920 1.398741
H -0.894306 4.071932 -1.519478
H -2.978302 5.262732 -1.921468
H -3.521000 4.933585 -0.184103
H 0.796372 -0.054406 2.294275
H 1.620721 -1.317446 1.408736
H -1.379363 -0.820259 1.419512
H -0.715690 -1.605388 -0.795219
H -1.801341 -3.930584 0.820166
H -2.737696 -2.523260 0.355877
H -2.331954 -3.008322 -2.047481
H -1.314569 -4.366024 -1.621313
H -3.531717 -5.149249 -2.301842
H -3.202121 -5.576625 -0.634975
H -5.516987 -4.839700 -0.899261
H -4.663972 -3.759473 0.176864
H -4.407116 -2.097841 -1.664060
H -5.188556 -3.198898 -2.778691
H -6.501498 -1.986418 -0.279182
H -6.804508 -1.477994 -1.942287
H -7.299658 -3.085939 -1.405050
H 4.722505 -2.100322 -1.329012
H 0.124078 -3.012127 2.332664
H 1.247015 -2.606218 -0.417208

2b-c505, ΔG = 1.8242 kcal/mol, population = 0.52 %

C 5.377306 0.330732 0.267388
C 4.752274 1.722423 0.309904
C 3.255176 1.664908 0.355586
C 2.524168 0.573607 -0.013432
C 3.200393 -0.580845 -0.548549
C 4.699167 -0.494539 -0.812713
C 1.014918 0.505943 0.147294
N 2.620206 2.817889 0.762374
C 1.270141 2.949527 0.636340
C 0.483306 1.903013 0.278557
C 0.536720 4.234854 0.856031
C -0.885707 3.914329 0.344604
C -0.871796 2.365822 0.149874
O -1.894180 1.726031 -0.059959
O -1.848918 4.255051 1.331091
C -1.145825 4.567043 -0.989061
C -2.202182 5.316315 -1.273844
C 0.643786 -0.383369 1.362444
C -0.710789 -1.092493 1.287920
C -0.849122 -1.990644 0.050174
C -2.175737 -2.735214 0.014186
C -2.423163 -3.453187 -1.312364
C -3.690016 -4.312448 -1.324529
C -4.986295 -3.515417 -1.161086
C -6.260472 -4.346601 -1.340828
C -6.452520 -5.428658 -0.277441
O 2.618687 -1.637395 -0.831153
O 5.259448 -1.791279 -0.896645
O -0.897290 -1.856961 2.489815
O 0.215399 -2.953547 0.018034
H 6.445876 0.402801 0.065624
H 5.248582 -0.170251 1.229834
H 5.043933 2.294208 -0.578673
H 5.116757 2.283247 1.172940
H 4.811603 0.023008 -1.778726
H 0.608511 0.052170 -0.757661
H 3.179432 3.613895 1.033014
H 0.496023 4.481587 1.918598
H 1.003433 5.069759 0.332048
H -2.654826 3.760114 1.123766
H -0.393692 4.366623 -1.746996
H -2.332553 5.746632 -2.258451
H -2.960516 5.532602 -0.531908
H 0.667421 0.226131 2.267656

H 1.413029 -1.148638 1.484512
H -1.517523 -0.360084 1.269212
H -0.794676 -1.356078 -0.840676
H -2.199571 -3.455576 0.836535
H -2.971631 -2.011983 0.208307
H -2.481970 -2.711378 -2.117586
H -1.559194 -4.084112 -1.533936
H -3.735634 -4.863237 -2.270560
H -3.614116 -5.066214 -0.534508
H -5.010910 -3.042563 -0.174028
H -4.988150 -2.699128 -1.891927
H -7.123920 -3.674583 -1.324271
H -6.251289 -4.808729 -2.334063
H -6.444436 -4.993340 0.725825
H -7.405633 -5.945523 -0.409002
H -5.662374 -6.180645 -0.318167
H 4.517561 -2.384911 -1.099433
H -0.306766 -2.620058 2.422723
H 1.026894 -2.502990 -0.276799

2b-c48, ΔG = 1.8380 kcal/mol, population = 0.51 %
C 5.006410 0.423392 1.334567
C 3.854166 1.277466 1.855104
C 2.614202 1.113291 1.029577
C 2.597879 0.603126 -0.232842
C 3.851022 0.287588 -0.878427
C 5.151800 0.642486 -0.160421
C 1.306012 0.336309 -0.990935
N 1.445146 1.543832 1.624035
C 0.292880 1.623636 0.909230
C 0.204357 1.132201 -0.353742
C -0.966465 2.243590 1.426498
C -1.900782 2.226862 0.196612
C -1.115081 1.384855 -0.859300
O -1.629662 1.005706 -1.904172
O -3.107145 1.546136 0.519476
C -2.130793 3.611814 -0.346975
C -3.320042 4.135789 -0.611412
C 0.937367 -1.168306 -1.085162
C 0.500948 -1.826315 0.215615
C -0.082851 -3.230927 0.014272
C -1.351911 -3.285710 -0.829393
C -2.451926 -2.305790 -0.420687
C -3.757716 -2.552790 -1.177690

C -4.810399 -1.459039 -0.982024
C -5.262033 -1.261166 0.465700
C -6.380112 -0.228901 0.601536
O 3.922396 -0.235399 -1.993294
O 6.221306 -0.115155 -0.696235
O 1.567245 -1.909499 1.185455
O -0.378945 -3.794011 1.300352
H 5.932990 0.685847 1.845129
H 4.805887 -0.634154 1.522180
H 4.136195 2.336694 1.848570
H 3.625144 1.024098 2.892609
H 5.326667 1.713925 -0.346315
H 1.447377 0.669252 -2.022455
H 1.479421 1.908798 2.564674
H -1.398683 1.636227 2.224020
H -0.795443 3.245657 1.822116
H -3.501230 1.254131 -0.315007
H -1.224768 4.179676 -0.539469
H -3.412392 5.135977 -1.014841
H -4.235448 3.588932 -0.423334
H 1.784845 -1.712816 -1.508222
H 0.120899 -1.250168 -1.803567
H -0.253784 -1.216150 0.711430
H 0.686637 -3.847487 -0.472717
H -1.086466 -3.123731 -1.877206
H -1.731896 -4.310037 -0.768558
H -2.622222 -2.381718 0.655073
H -2.126527 -1.281363 -0.615455
H -3.536629 -2.638391 -2.246969
H -4.174849 -3.519245 -0.872837
H -4.415360 -0.513873 -1.374579
H -5.686218 -1.693811 -1.596256
H -5.599592 -2.222813 0.867197
H -4.412060 -0.952118 1.078552
H -6.685034 -0.105210 1.642909
H -6.060829 0.749558 0.232749
H -7.261511 -0.525974 0.027199
H 5.909149 -0.416798 -1.566537
H 2.349230 -2.272962 0.750000
H 0.354254 -3.530568 1.875239

2b-c29, ΔG = 1.8819 kcal/mol, population = 0.47 %
C 4.342067 -0.002112 2.015027
C 3.149664 0.890361 2.348368

C 2.145894 0.925865 1.234742
C 2.424962 0.589343 -0.054327
C 3.779665 0.228119 -0.407231
C 4.875086 0.376021 0.645309
C 1.358586 0.517839 -1.137191
N 0.880663 1.355102 1.592397
C -0.064790 1.582566 0.644189
C 0.142346 1.256412 -0.658077
C -1.400557 2.199096 0.923034
C -1.942946 2.491076 -0.494702
C -1.011364 1.650598 -1.419035
O -1.299606 1.401831 -2.582908
O -3.281258 2.047142 -0.628747
C -1.768757 3.948331 -0.847818
C -2.721228 4.722245 -1.349989
C 0.998883 -0.930113 -1.553465
C 0.675261 -1.867310 -0.396436
C -0.236715 -3.030863 -0.809578
C -1.665351 -2.623345 -1.159791
C -2.405254 -1.843289 -0.072195
C -3.898466 -1.712211 -0.367765
C -4.675990 -0.905246 0.677783
C -4.612047 -1.454600 2.107971
C -5.131030 -2.885203 2.249014
O 4.095068 -0.179143 -1.527140
O 5.998337 -0.408407 0.288141
O 1.871717 -2.406600 0.208320
O -0.297687 -3.982832 0.260188
H 5.122696 0.111555 2.767277
H 4.033114 -1.049555 1.998467
H 3.486926 1.915265 2.541803
H 2.657377 0.548601 3.261245
H 5.154880 1.441337 0.654903
H 1.745401 1.006174 -2.035932
H 0.695820 1.587213 2.557338
H -2.057587 1.485154 1.423646
H -1.323942 3.088619 1.549210
H -3.419025 1.845195 -1.566047
H -0.771101 4.342978 -0.677325
H -2.528471 5.759633 -1.591656
H -3.721807 4.346550 -1.522694
H 1.821968 -1.353299 -2.132945
H 0.144449 -0.865635 -2.227443
H 0.191484 -1.325426 0.416961

H 0.213594 -3.513783 -1.689158
H -1.658595 -2.051708 -2.091170
H -2.213591 -3.545380 -1.375111
H -2.256915 -2.346673 0.884910
H -1.977679 -0.842292 0.031987
H -4.027049 -1.232113 -1.343628
H -4.330924 -2.713162 -0.465533
H -4.303784 0.122161 0.670256
H -5.726189 -0.855949 0.368701
H -3.584104 -1.402831 2.478851
H -5.197096 -0.794906 2.756100
H -5.135942 -3.202018 3.294236
H -6.153127 -2.969009 1.869076
H -4.513643 -3.594100 1.693529
H 5.896165 -0.582578 -0.663169
H 2.476236 -2.673887 -0.496065
H 0.591713 -3.998042 0.643675

2b-c321, ΔG = 1.8844 kcal/mol, population = 0.47 %
C 4.888232 0.892451 -0.411567
C 4.001395 2.096575 -0.112540
C 2.590569 1.695729 0.199270
C 2.046013 0.489111 -0.135624
C 2.835371 -0.442722 -0.900535
C 4.195568 0.002012 -1.427420
C 0.642552 0.072058 0.285682
N 1.824985 2.652372 0.825605
C 0.483363 2.478765 0.980180
C -0.124241 1.303183 0.677913
C -0.434821 3.547919 1.483401
C -1.833625 2.944824 1.232447
C -1.536332 1.448133 0.912208
O -2.418521 0.600635 0.905153
O -2.629401 3.027190 2.404296
C -2.488975 3.566817 0.024758
C -3.726921 4.041292 -0.005718
C 0.700464 -0.975955 1.431250
C -0.406204 -2.037488 1.432365
C -0.414601 -2.891994 0.157821
C -1.497356 -3.965068 0.134248
C -2.934538 -3.451057 0.280025
C -3.317451 -2.308001 -0.667100
C -3.142451 -2.622981 -2.152446
C -3.509913 -1.452649 -3.070551

C -2.588947 -0.239414 -2.918293
O 2.455224 -1.587727 -1.182045
O 4.989025 -1.124255 -1.750043
O -0.243251 -2.876922 2.585933
O 0.849409 -3.560992 0.017749
H 5.853200 1.219878 -0.797934
H 5.066593 0.318265 0.500662
H 3.978645 2.772318 -0.975345
H 4.402410 2.674718 0.722449
H 3.995450 0.589135 -2.337657
H 0.161974 -0.383705 -0.581656
H 2.249623 3.533622 1.076067
H -0.293178 3.708285 2.553787
H -0.277527 4.499311 0.973820
H -3.311408 2.343587 2.328798
H -1.870815 3.585994 -0.868397
H -4.140878 4.462499 -0.912865
H -4.358492 4.035098 0.873735
H 0.674078 -0.453063 2.388966
H 1.661293 -1.492470 1.389126
H -1.375139 -1.553814 1.544113
H -0.562129 -2.231012 -0.699005
H -1.388808 -4.504211 -0.809514
H -1.289621 -4.684680 0.929630
H -3.611983 -4.296386 0.122355
H -3.098492 -3.120769 1.309730
H -4.365393 -2.043916 -0.486843
H -2.743153 -1.419079 -0.405545
H -2.105365 -2.909766 -2.354757
H -3.754996 -3.493682 -2.410553
H -3.481031 -1.794359 -4.109639
H -4.545063 -1.151694 -2.875216
H -2.651659 0.194817 -1.919256
H -1.546044 -0.520831 -3.091099
H -2.845843 0.542498 -3.636348
H 4.364077 -1.858832 -1.868823
H 0.509427 -3.453626 2.392761
H 1.468562 -2.920754 -0.375458

2b-c304, ΔG = 1.9020 kcal/mol, population = 0.45 %
C 5.003141 1.018174 -0.379557
C 4.091124 2.201458 -0.064782
C 2.692007 1.762960 0.245017
C 2.182325 0.549413 -0.111303

C 2.981585 -0.355115 -0.898416
C 4.335776 0.122366 -1.409400
C 0.801173 0.084697 0.311467
N 1.896237 2.684523 0.891177
C 0.557033 2.471118 1.029689
C -0.010900 1.281649 0.706166
C -0.405724 3.506220 1.522154
C -1.782095 2.859396 1.246736
C -1.433717 1.382130 0.885126
O -2.290182 0.511623 0.802602
O -2.579012 2.879440 2.422062
C -2.460094 3.493730 0.059180
C -3.713348 3.927052 0.043332
C 0.918589 -0.964478 1.446879
C -0.243151 -1.954353 1.569142
C -0.545653 -2.671414 0.249061
C -1.710141 -3.642260 0.359746
C -2.186295 -4.201247 -0.986950
C -2.500276 -3.143456 -2.052170
C -3.512705 -2.081736 -1.620690
C -3.797455 -1.047736 -2.709263
C -4.763770 0.045387 -2.255103
O 2.609481 -1.496773 -1.202811
O 5.155876 -0.984082 -1.736491
O 0.057266 -2.900220 2.607335
O 0.613717 -3.386714 -0.208853
H 5.962105 1.371202 -0.758364
H 5.191340 0.436954 0.526299
H 4.052185 2.887446 -0.918781
H 4.482098 2.776840 0.776817
H 4.130775 0.714116 -2.315700
H 0.338910 -0.386571 -0.556271
H 2.293075 3.575994 1.151105
H -0.286714 3.668630 2.594955
H -0.272910 4.463877 1.017536
H -3.257980 2.197472 2.314625
H -1.845628 3.557972 -0.834366
H -4.143695 4.357807 -0.851570
H -4.342745 3.876341 0.922847
H 1.041329 -0.445481 2.399342
H 1.830458 -1.543570 1.289016
H -1.147323 -1.436247 1.886707
H -0.813905 -1.909124 -0.483577
H -1.428411 -4.465148 1.020545

H -2.531124 -3.116770 0.854147
H -1.426571 -4.880742 -1.381409
H -3.080461 -4.807192 -0.808409
H -1.573663 -2.646667 -2.358629
H -2.873272 -3.652071 -2.947299
H -4.449655 -2.569728 -1.326539
H -3.148227 -1.555258 -0.734730
H -2.851254 -0.590350 -3.018891
H -4.198301 -1.551078 -3.595642
H -4.361592 0.586723 -1.395265
H -4.951397 0.770603 -3.050245
H -5.726299 -0.380731 -1.959189
H 4.547723 -1.728410 -1.877630
H 0.721795 -3.501537 2.242899
H 1.258267 -2.744661 -0.555614

2b-c7, ΔG = 1.9283 kcal/mol, population = 0.44 %
C 5.052141 0.403487 1.177641
C 3.915435 1.212305 1.795672
C 2.640773 1.077148 1.017673
C 2.577748 0.660774 -0.277603
C 3.808241 0.396408 -0.991180
C 5.134210 0.716381 -0.305279
C 1.261511 0.423891 -1.002731
N 1.491768 1.439594 1.695736
C 0.299012 1.512056 1.049200
C 0.160930 1.103230 -0.238425
C -0.956959 2.029371 1.677091
C -1.954588 2.059767 0.498694
C -1.199604 1.314477 -0.646573
O -1.761783 0.967020 -1.677945
O -3.125920 1.328903 0.836203
C -2.246757 3.468744 0.053235
C -3.460859 3.968834 -0.132591
C 0.955711 -1.080837 -1.195641
C 0.934170 -1.890120 0.103195
C -0.225410 -2.894149 0.166651
C -1.564378 -2.194030 0.332070
C -2.770319 -3.132803 0.330092
C -4.103198 -2.385924 0.449321
C -4.474425 -1.565446 -0.786672
C -5.756885 -0.752161 -0.609001
C -6.106910 0.090111 -1.834746
O 3.836186 -0.052061 -2.138548

O 6.186683 0.005399 -0.930481
O 2.183108 -2.575372 0.333646
O -0.029230 -3.775753 1.277507
H 5.996504 0.644407 1.665664
H 4.867128 -0.665467 1.306482
H 4.183980 2.274374 1.833226
H 3.738664 0.901035 2.827346
H 5.292344 1.799304 -0.430407
H 1.333481 0.857315 -2.003838
H 1.561948 1.727469 2.660905
H -1.317637 1.341433 2.443852
H -0.809519 3.005346 2.141390
H -3.544859 1.062590 0.005524
H -1.366438 4.075893 -0.138419
H -3.599262 4.988341 -0.468846
H -4.350962 3.381985 0.056089
H 1.701915 -1.504751 -1.869211
H -0.003463 -1.158708 -1.708094
H 0.835532 -1.232367 0.968673
H -0.228687 -3.481403 -0.764423
H -1.541871 -1.628625 1.269622
H -1.673747 -1.464418 -0.469544
H -2.766891 -3.726309 -0.591320
H -2.674317 -3.840139 1.156611
H -4.903826 -3.105951 0.646063
H -4.064446 -1.724330 1.322203
H -3.655510 -0.888879 -1.049499
H -4.589667 -2.238442 -1.643940
H -6.585257 -1.430445 -0.380187
H -5.652665 -0.101104 0.266322
H -5.311214 0.804450 -2.063057
H -6.244257 -0.541078 -2.716466
H -7.027976 0.656307 -1.681550
H 5.843408 -0.242896 -1.805742
H 2.466865 -2.981020 -0.495526
H 0.925631 -3.935249 1.314670

2b-c300, ΔG = 1.9283 kcal/mol, population = 0.44 %
C 3.784297 -0.386583 2.067713
C 3.572309 1.102747 1.807128
C 2.269080 1.371177 1.115723
C 1.569479 0.431790 0.422283
C 2.129326 -0.894476 0.264208
C 3.539290 -1.159560 0.784051

C 0.194571 0.690578 -0.172250
N 1.807167 2.673172 1.192446
C 0.722212 3.064155 0.480200
C -0.025429 2.172117 -0.224239
C 0.238990 4.477419 0.393476
C -0.880418 4.403015 -0.672081
C -1.074300 2.875834 -0.887869
O -2.026050 2.415317 -1.521215
O -2.078139 4.961368 -0.151454
C -0.445155 5.033289 -1.970899
C -1.117958 5.972858 -2.620867
C -0.918824 -0.046243 0.636421
C -1.802537 -0.954188 -0.211791
C -2.655848 -1.891968 0.647302
C -3.345968 -2.994768 -0.159408
C -2.407932 -3.920852 -0.942489
C -1.333110 -4.600198 -0.092213
C -0.519059 -5.629967 -0.876225
C 0.547694 -6.352029 -0.047284
C 1.687114 -5.448077 0.425141
O 1.523310 -1.811966 -0.289957
O 3.733282 -2.550087 0.967654
O -2.722492 -0.231339 -1.043371
O -3.628732 -1.125455 1.373798
H 4.797522 -0.566715 2.426880
H 3.087494 -0.737490 2.832822
H 4.381741 1.495380 1.181126
H 3.603788 1.664342 2.743020
H 4.229164 -0.787883 0.009927
H 0.186991 0.285945 -1.188793
H 2.336187 3.351579 1.721171
H -0.186299 4.801635 1.344897
H 1.040307 5.167771 0.128017
H -2.807382 4.640730 -0.701354
H 0.488750 4.648771 -2.371005
H -0.749564 6.377356 -3.554804
H -2.047221 6.375176 -2.237304
H -1.534304 0.682062 1.169104
H -0.439836 -0.664007 1.397511
H -1.143594 -1.560829 -0.839296
H -2.013619 -2.344625 1.403567
H -3.935836 -3.588444 0.545272
H -4.053469 -2.525378 -0.848333
H -3.016599 -4.689843 -1.428644

H -1.926055 -3.364638 -1.752619
H -0.660264 -3.844589 0.319058
H -1.807168 -5.092999 0.765055
H -1.204537 -6.371950 -1.299131
H -0.038040 -5.135115 -1.728258
H 0.070402 -6.821473 0.819742
H 0.966646 -7.167453 -0.644464
H 1.328130 -4.653816 1.081975
H 2.440840 -6.016683 0.973776
H 2.179942 -4.976424 -0.429924
H 3.044883 -2.981850 0.433461
H -2.387865 0.664807 -1.244917
H -3.994936 -0.507734 0.723148

2b-c503, ΔG = 1.9290 kcal/mol, population = 0.43 %
C 5.410408 0.385437 0.544968
C 4.752703 1.762352 0.563490
C 3.259315 1.674946 0.469702
C 2.587900 0.579413 0.010561
C 3.335840 -0.547812 -0.485143
C 4.849926 -0.424989 -0.611021
C 1.071448 0.478245 0.032705
N 2.566326 2.806095 0.840830
C 1.230960 2.914850 0.594457
C 0.501092 1.862176 0.145568
C 0.455841 4.182097 0.772976
C -0.915113 3.843272 0.147223
C -0.848386 2.301291 -0.086347
O -1.835207 1.646448 -0.395090
O -1.956943 4.130393 1.069458
C -1.094487 4.529206 -1.182645
C -2.145874 5.262234 -1.522850
C 0.612243 -0.443932 1.192427
C -0.708271 -1.189006 0.978917
C -0.697597 -2.085393 -0.269482
C -1.967934 -2.909378 -0.435101
C -3.240350 -2.073127 -0.576280
C -4.471140 -2.897023 -0.964719
C -4.889231 -3.924933 0.089024
C -6.214457 -4.628976 -0.218414
C -6.180740 -5.496108 -1.477712
O 2.807086 -1.609682 -0.842089
O 5.443567 -1.707887 -0.675448
O -0.995854 -1.965552 2.152422

O 0.403234 -3.005210 -0.207240
H 6.490841 0.484306 0.442161
H 5.206715 -0.142445 1.479601
H 5.111478 2.363590 -0.279943
H 5.023816 2.307061 1.470077
H 5.036500 0.119373 -1.550204
H 0.756807 0.036376 -0.913993
H 3.079964 3.604265 1.185771
H 0.327994 4.415033 1.831752
H 0.946088 5.032197 0.296857
H -2.732066 3.622811 0.788315
H -0.284405 4.369189 -1.888657
H -2.214946 5.718953 -2.501754
H -2.961254 5.438823 -0.832716
H 0.529576 0.150877 2.103853
H 1.389742 -1.187536 1.378221
H -1.524265 -0.474283 0.885759
H -0.590233 -1.444767 -1.150879
H -1.841014 -3.527329 -1.329018
H -2.052762 -3.591108 0.412776
H -3.446504 -1.548291 0.361339
H -3.077962 -1.299761 -1.333683
H -5.310948 -2.215933 -1.140667
H -4.277176 -3.398092 -1.918066
H -4.105293 -4.680416 0.203392
H -4.970764 -3.418510 1.057044
H -6.487777 -5.254151 0.637007
H -7.005819 -3.877450 -0.314149
H -7.125566 -6.026314 -1.615853
H -6.003722 -4.900467 -2.375336
H -5.384877 -6.243475 -1.413903
H 4.734812 -2.310770 -0.955232
H -0.376156 -2.708067 2.149615
H 1.220196 -2.521206 -0.425159

2b-c319, ΔG = 1.9302 kcal/mol, population = 0.43 %
C 2.768402 0.338179 3.428112
C 2.570060 1.753071 2.889880
C 1.604675 1.786595 1.742974
C 1.279279 0.699024 0.991946
C 1.944985 -0.561805 1.245991
C 3.072933 -0.599051 2.273056
C 0.212039 0.725642 -0.090226
N 1.061593 3.025707 1.452949

C 0.297865 3.203357 0.347306
C -0.071021 2.155613 -0.438253
C -0.211151 4.531203 -0.117519
C -0.843427 4.205553 -1.491636
C -0.840272 2.651610 -1.532781
O -1.453260 2.013801 -2.390964
O -2.183270 4.675444 -1.528234
C 0.002759 4.730565 -2.623875
C -0.439289 5.504871 -3.605179
C -1.070915 -0.039760 0.362187
C -1.511355 -1.135212 -0.602585
C -2.559246 -2.062560 0.019974
C -2.822926 -3.323070 -0.807564
C -1.600820 -4.216110 -1.051852
C -0.878820 -4.663797 0.220161
C 0.235912 -5.673682 -0.054196
C 0.961359 -6.166709 1.201646
C 1.778806 -5.087665 1.913011
O 1.652792 -1.600937 0.653562
O 3.276692 -1.927325 2.719259
O -2.100189 -0.624171 -1.807735
O -3.781825 -1.337502 0.223483
H 3.583689 0.320022 4.151334
H 1.861358 -0.002706 3.933218
H 3.526548 2.164130 2.547268
H 2.212030 2.417198 3.679199
H 3.975582 -0.239564 1.754078
H 0.611812 0.209902 -0.968486
H 1.308213 3.817126 2.029564
H -0.981919 4.909319 0.556377
H 0.583237 5.276039 -0.175225
H -2.630904 4.211369 -2.250212
H 1.041906 4.414972 -2.597557
H 0.223682 5.840803 -4.392066
H -1.469139 5.836702 -3.647725
H -1.887861 0.667292 0.522411
H -0.870557 -0.506959 1.327539
H -0.626886 -1.728019 -0.852185
H -2.217378 -2.348019 1.015429
H -3.592386 -3.897613 -0.283345
H -3.251411 -3.021104 -1.767106
H -1.931090 -5.101336 -1.604543
H -0.889375 -3.703179 -1.706556
H -0.460366 -3.792770 0.729099

H -1.604869 -5.107911 0.911616
H -0.190807 -6.534134 -0.580315
H 0.966247 -5.226171 -0.738714
H 0.227181 -6.586974 1.897552
H 1.626430 -6.990147 0.924720
H 1.148338 -4.271333 2.269754
H 2.309208 -5.497902 2.774865
H 2.522658 -4.661952 1.233361
H 2.868511 -2.494678 2.043115
H -1.774855 0.276090 -2.006157
H -3.918838 -0.844966 -0.599719

2b-c43, ΔG = 1.9628 kcal/mol, population = 0.41 %
C 5.002215 0.236892 0.947486
C 3.985082 1.168320 1.600653
C 2.648301 1.102315 0.924427
C 2.457666 0.642627 -0.343732
C 3.605339 0.240671 -1.127043
C 5.000690 0.472872 -0.551828
C 1.075267 0.476486 -0.955433
N 1.582767 1.570018 1.671169
C 0.351819 1.707721 1.111618
C 0.093401 1.273816 -0.147915
C -0.819188 2.325228 1.809086
C -1.898415 2.385843 0.704070
C -1.271394 1.576588 -0.473757
O -1.919214 1.254523 -1.462112
O -3.076485 1.718769 1.140278
C -2.150943 3.800622 0.254265
C -3.345983 4.369780 0.174529
C 0.630965 -1.005171 -1.028717
C 0.791596 -1.776389 0.282742
C -0.409061 -2.687005 0.580560
C -1.603777 -1.883277 1.076447
C -2.909920 -2.679372 1.158446
C -3.553261 -2.984038 -0.198951
C -4.070186 -1.745513 -0.931166
C -4.754070 -2.067777 -2.259387
C -5.263117 -0.822257 -2.982912
O 3.509441 -0.262792 -2.247558
O 5.939541 -0.356763 -1.210944
O 2.011222 -2.546744 0.304336
O -0.059077 -3.633807 1.596003
H 5.996869 0.417697 1.355044

H 4.739333 -0.804930 1.144354
H 4.340312 2.204368 1.560096
H 3.863130 0.923570 2.657822
H 5.240819 1.530715 -0.743978
H 1.097250 0.850545 -1.982128
H 1.747894 1.889723 2.614357
H -1.164533 1.684667 2.622602
H -0.576951 3.302485 2.228468
H -3.573778 1.471040 0.347083
H -1.259115 4.350938 -0.032288
H -3.457217 5.390147 -0.169104
H -4.245413 3.839814 0.461541
H 1.203389 -1.509612 -1.809148
H -0.410575 -1.021357 -1.351976
H 0.899829 -1.093580 1.127200
H -0.664921 -3.229115 -0.340557
H -1.340662 -1.498316 2.065743
H -1.747424 -1.011141 0.438589
H -2.722186 -3.613331 1.694121
H -3.625318 -2.114159 1.764075
H -2.840180 -3.511874 -0.841254
H -4.387270 -3.676431 -0.044184
H -4.776647 -1.215409 -0.281035
H -3.250254 -1.047799 -1.121554
H -4.048885 -2.603145 -2.904217
H -5.587718 -2.755191 -2.080366
H -5.990753 -0.283962 -2.369591
H -4.442343 -0.134311 -3.201019
H -5.747043 -1.076755 -3.928431
H 5.511279 -0.614935 -2.044928
H 2.137894 -2.945475 -0.565855
H 0.870080 -3.854741 1.432017

2b-c480, ΔG = 2.0118 kcal/mol, population = 0.38 %
C 3.458371 -0.619200 2.237678
C 3.394844 0.886233 1.995561
C 2.225643 1.267578 1.136742
C 1.569715 0.400804 0.316537
C 2.067368 -0.952124 0.183382
C 3.354531 -1.339920 0.906737
C 0.323296 0.776891 -0.471921
N 1.845835 2.594713 1.203755
C 0.853732 3.076105 0.414819
C 0.144540 2.266153 -0.416098

C 0.424242 4.509050 0.401488
C -0.642491 4.549205 -0.716882
C -0.860839 3.049265 -1.065771
O -1.805659 2.674503 -1.762091
O -1.852165 5.090849 -0.205519
C -0.139432 5.269307 -1.942145
C -0.775266 6.258854 -2.554220
C -0.944516 0.037644 0.053813
C -1.625688 -0.825773 -0.996835
C -2.886924 -1.511730 -0.463141
C -2.645084 -2.469857 0.699846
C -1.503040 -3.466668 0.499283
C -1.357369 -4.435392 1.672698
C -0.064804 -5.258641 1.635204
C 0.136406 -6.101526 0.370764
C -0.969653 -7.127308 0.126388
O 1.509631 -1.803854 -0.510313
O 3.409045 -2.745992 1.071075
O -1.977913 -0.090394 -2.182269
O -3.501678 -2.243129 -1.535593
H 4.390381 -0.882833 2.737492
H 2.630717 -0.932247 2.878805
H 4.311628 1.228956 1.502392
H 3.332512 1.424517 2.943633
H 4.182722 -1.009978 0.259714
H 0.473131 0.469057 -1.509819
H 2.337380 3.215262 1.830294
H -0.034399 4.787407 1.351865
H 1.262903 5.182805 0.221134
H -2.563222 4.818687 -0.803530
H 0.809312 4.904894 -2.325626
H -0.360883 6.725372 -3.438639
H -1.719304 6.640706 -2.186554
H -1.656593 0.769462 0.445812
H -0.663460 -0.595187 0.894951
H -0.924894 -1.593597 -1.325268
H -3.580154 -0.725252 -0.132459
H -2.457477 -1.879408 1.601207
H -3.580468 -3.008497 0.880446
H -1.667806 -4.020158 -0.428153
H -0.560407 -2.929457 0.371723
H -1.374056 -3.865202 2.607870
H -2.226644 -5.099540 1.709899
H 0.786134 -4.576949 1.740022

H -0.039409 -5.919278 2.508815
H 0.220973 -5.443306 -0.498840
H 1.096665 -6.620020 0.450443
H -0.745859 -7.747614 -0.744194
H -1.086472 -7.790445 0.988199
H -1.933084 -6.645542 -0.052415
H 2.803408 -3.107622 0.401729
H -2.042585 0.864387 -1.982261
H -3.448971 -1.656623 -2.304224

2b-c266, ΔG = 2.0193 kcal/mol, population = 0.37 %
C 4.108578 -0.344976 1.423967
C 3.733600 1.134572 1.459666
C 2.343443 1.373942 0.951477
C 1.656900 0.496240 0.170056
C 2.310952 -0.717594 -0.273608
C 3.789722 -0.908306 0.050523
C 0.208846 0.707654 -0.242149
N 1.781837 2.590983 1.294900
C 0.604975 2.986089 0.751009
C -0.136876 2.150885 -0.026617
C 0.010112 4.346409 0.936098
C -1.174381 4.347666 -0.058640
C -1.289070 2.857965 -0.484973
O -2.263379 2.425624 -1.103757
O -2.364250 4.747586 0.603603
C -0.862088 5.179284 -1.277826
C -1.632318 6.148506 -1.752100
C -0.739509 -0.264347 0.524918
C -1.689561 -1.047094 -0.377997
C -2.287006 -2.260453 0.339892
C -3.014223 -3.231085 -0.591487
C -2.160322 -3.808683 -1.730032
C -0.793063 -4.360158 -1.308310
C -0.853118 -5.480431 -0.269525
C 0.526724 -5.984759 0.166604
C 1.338227 -4.951485 0.950926
O 1.731050 -1.589360 -0.922108
O 4.134639 -2.278370 -0.042689
O -2.805790 -0.272744 -0.835703
O -3.167595 -1.815006 1.383446
H 5.168535 -0.471368 1.644340
H 3.539277 -0.896198 2.176301
H 4.429122 1.715141 0.842712

H 3.817780 1.528260 2.474769
H 4.346718 -0.332687 -0.705617
H 0.128233 0.470685 -1.307361
H 2.305123 3.224317 1.881919
H -0.369718 4.468487 1.952038
H 0.732871 5.140527 0.746649
H -3.108472 4.453501 0.058917
H 0.066654 4.919357 -1.777816
H -1.349843 6.701686 -2.638536
H -2.558805 6.427712 -1.266251
H -1.308831 0.283778 1.278437
H -0.124013 -0.985912 1.063535
H -1.111735 -1.402214 -1.236272
H -1.476600 -2.786271 0.845587
H -3.403623 -4.040151 0.031541
H -3.881684 -2.719818 -1.017102
H -2.735266 -4.602103 -2.218140
H -2.005095 -3.038884 -2.491211
H -0.280254 -4.737600 -2.199458
H -0.172925 -3.542215 -0.936038
H -1.397208 -5.140689 0.617779
H -1.432168 -6.314509 -0.680169
H 0.399324 -6.880000 0.782533
H 1.091330 -6.298811 -0.718176
H 0.798074 -4.635549 1.847966
H 2.299249 -5.362107 1.268096
H 1.539992 -4.058561 0.357718
H 3.442756 -2.683508 -0.592559
H -2.542410 0.655202 -0.997266
H -3.694304 -1.107470 0.982865

2b-c600, ΔG = 2.0256 kcal/mol, population = 0.37 %
C 5.131367 0.773050 -0.228158
C 4.311280 2.035693 0.024545
C 2.866824 1.726698 0.277787
C 2.266168 0.555411 -0.079214
C 3.021269 -0.434482 -0.804671
C 4.430986 -0.085339 -1.267740
C 0.829329 0.226402 0.286554
N 2.129873 2.728617 0.870871
C 0.772541 2.640200 0.949259
C 0.112334 1.502386 0.614877
C -0.109620 3.767137 1.385133
C -1.525611 3.250135 1.045978

C -1.302057 1.740754 0.715183
O -2.232420 0.954884 0.590438
O -2.372667 3.363273 2.180214
C -2.077365 3.934695 -0.178270
C -3.268495 4.512812 -0.252639
C 0.796918 -0.790931 1.456520
C -0.429209 -1.705904 1.517329
C -0.637610 -2.502090 0.222141
C -1.853097 -3.414313 0.287060
C -2.165346 -4.135487 -1.027696
C -2.361571 -3.219771 -2.243464
C -3.407906 -2.114113 -2.067878
C -4.816942 -2.620376 -1.760228
C -5.838745 -1.488866 -1.660275
O 2.571694 -1.551573 -1.095396
O 5.165241 -1.264057 -1.540310
O -0.290305 -2.590682 2.639981
O 0.523465 -3.297791 -0.061972
H 6.130963 1.035599 -0.573951
H 5.233995 0.198369 0.695384
H 4.368642 2.702957 -0.843105
H 4.714086 2.594216 0.872147
H 4.313541 0.502002 -2.192204
H 0.365703 -0.229060 -0.589399
H 2.591983 3.588206 1.129753
H -0.028459 3.928842 2.461672
H 0.138921 4.702685 0.882608
H -3.116588 2.759590 2.040267
H -1.421772 3.912102 -1.044273
H -3.607279 4.977525 -1.169650
H -3.934964 4.550716 0.599847
H 0.876450 -0.248807 2.400656
H 1.681715 -1.427167 1.389457
H -1.328040 -1.119015 1.705411
H -0.792453 -1.786167 -0.587747
H -1.700638 -4.151958 1.078058
H -2.707842 -2.805309 0.594246
H -1.355885 -4.835088 -1.248438
H -3.061849 -4.743575 -0.879080
H -1.404543 -2.760559 -2.508487
H -2.641753 -3.841142 -3.100574
H -3.095586 -1.427218 -1.274716
H -3.440775 -1.516042 -2.984933
H -5.123605 -3.326722 -2.539434

H -4.811937 -3.183754 -0.822923
H -6.837346 -1.868169 -1.432476
H -5.561973 -0.782586 -0.872901
H -5.898997 -0.930520 -2.598244
H 4.501595 -1.958448 -1.686440
H 0.380764 -3.241454 2.390721
H 1.213462 -2.711477 -0.420057

2b-c548, ΔG = 2.0262 kcal/mol, population = 0.37 %
C 4.999419 0.148563 -0.705421
C 4.417914 1.554746 -0.582186
C 2.987009 1.534126 -0.136689
C 2.179143 0.438536 -0.224190
C 2.678621 -0.762326 -0.844628
C 4.050445 -0.725319 -1.507809
C 0.771462 0.412129 0.344737
N 2.487484 2.724061 0.346847
C 1.152672 2.876038 0.573471
C 0.295841 1.824899 0.512410
C 0.506262 4.188471 0.887243
C -1.001376 3.864189 0.789882
C -1.043213 2.305732 0.717334
O -2.084767 1.673944 0.838901
O -1.673527 4.299859 1.962392
C -1.596726 4.421210 -0.478147
C -2.688945 5.170766 -0.538335
C 0.751691 -0.383145 1.675235
C -0.574548 -1.053574 2.043588
C -1.091602 -1.991971 0.944690
C -2.386924 -2.690267 1.335356
C -3.009516 -3.523460 0.212612
C -3.466109 -2.701837 -0.995152
C -4.256520 -3.504347 -2.032200
C -3.456496 -4.614677 -2.715896
C -4.249759 -5.325992 -3.810723
O 2.035697 -1.820167 -0.888777
O 4.560979 -2.037036 -1.656215
O -0.402073 -1.763029 3.280613
O -0.108448 -2.994535 0.644499
H 5.973779 0.184826 -1.192258
H 5.133777 -0.291843 0.285384
H 4.466593 2.070158 -1.548323
H 5.002675 2.154094 0.118828
H 3.899955 -0.270492 -2.499615

H 0.134948 -0.096714 -0.380421
H 3.101495 3.523597 0.407873
H 0.746339 4.506496 1.903484
H 0.821675 4.976314 0.202099
H -2.504351 3.805248 2.013063
H -1.066213 4.148060 -1.386076
H -3.068309 5.529592 -1.486481
H -3.228986 5.459163 0.354692
H 1.044855 0.281715 2.489788
H 1.513429 -1.163686 1.624108
H -1.338215 -0.299530 2.233447
H -1.280914 -1.388839 0.053534
H -2.191613 -3.326345 2.201545
H -3.097056 -1.923728 1.661672
H -2.291461 -4.283166 -0.101886
H -3.873730 -4.060686 0.617709
H -4.085330 -1.870050 -0.641710
H -2.599430 -2.249200 -1.487788
H -5.141127 -3.941557 -1.554123
H -4.631285 -2.819134 -2.800293
H -2.543691 -4.184987 -3.143169
H -3.130761 -5.347612 -1.972972
H -4.555298 -4.625329 -4.592322
H -3.662015 -6.116729 -4.282171
H -5.156015 -5.782185 -3.403350
H 3.789734 -2.625877 -1.607457
H 0.125024 -2.547381 3.073941
H 0.599084 -2.585544 0.114979

2b-c310, ΔG = 2.0419 kcal/mol, population = 0.36 %
C 4.476492 -0.338255 0.220455
C 4.122147 1.143021 0.325335
C 2.639368 1.367077 0.345229
C 1.726546 0.458175 -0.094388
C 2.191140 -0.775775 -0.692638
C 3.689021 -0.960213 -0.919048
C 0.224798 0.656127 0.036957
N 2.227910 2.599059 0.821132
C 0.925513 2.967744 0.749712
C -0.037246 2.102292 0.331129
C 0.417001 4.327261 1.112542
C -1.068175 4.276039 0.682660
C -1.294313 2.777826 0.335365
O -2.417345 2.311186 0.134447

O -1.896741 4.635406 1.779201
C -1.315099 5.109753 -0.549094
C -2.250403 6.043111 -0.658809
C -0.381828 -0.292260 1.118586
C -1.529780 -1.153963 0.602366
C -1.849722 -2.318894 1.542867
C -2.819506 -3.337191 0.938599
C -2.365883 -3.969102 -0.382007
C -1.007170 -4.670894 -0.310529
C -0.614340 -5.395285 -1.601218
C -0.368364 -4.463784 -2.790326
C 0.119957 -5.208127 -4.031972
O 1.427295 -1.674388 -1.048017
O 3.987756 -2.335572 -1.071579
O -2.754560 -0.423130 0.444170
O -2.371300 -1.810364 2.780810
H 5.545957 -0.459399 0.048520
H 4.223479 -0.852856 1.150611
H 4.541053 1.692368 -0.525524
H 4.562128 1.580989 1.223803
H 3.929244 -0.422680 -1.849994
H -0.230879 0.392472 -0.922348
H 2.921874 3.254073 1.151355
H 0.478597 4.491258 2.189865
H 0.980320 5.118979 0.617371
H -2.790297 4.320645 1.580969
H -0.663014 4.883606 -1.387951
H -2.378224 6.598698 -1.578870
H -2.907644 6.289813 0.165608
H -0.711435 0.289968 1.981575
H 0.405478 -0.957659 1.475368
H -1.220597 -1.564215 -0.362903
H -0.913757 -2.815461 1.801025
H -2.974069 -4.121072 1.686127
H -3.784315 -2.844356 0.791894
H -3.122171 -4.697240 -0.692979
H -2.348315 -3.204998 -1.162582
H -0.224804 -3.945133 -0.069734
H -1.026739 -5.390115 0.515506
H 0.297148 -5.974494 -1.417925
H -1.392074 -6.122242 -1.863623
H -1.287473 -3.923161 -3.033692
H 0.364986 -3.705595 -2.499379
H 0.286732 -4.525725 -4.868470

H 1.061151 -5.727823 -3.832679
H -0.608822 -5.957202 -4.353450
H 3.142356 -2.757413 -1.302292
H -2.577678 0.520638 0.258979
H -3.021163 -1.140007 2.522099

2b-c225, ΔG = 2.0501 kcal/mol, population = 0.35 %
C 5.274064 0.989930 0.327036
C 4.325205 2.164386 0.546861
C 2.888377 1.737087 0.545842
C 2.441806 0.560522 0.017233
C 3.377833 -0.306507 -0.652711
C 4.805180 0.183107 -0.871127
C 0.995431 0.103532 0.132681
N 1.997759 2.635286 1.089774
C 0.654748 2.442815 0.970795
C 0.141050 1.293602 0.463188
C -0.365822 3.459508 1.375340
C -1.683902 2.863266 0.835114
C -1.291620 1.411188 0.419910
O -2.132116 0.566834 0.140619
O -2.656426 2.805995 1.868778
C -2.161248 3.603600 -0.387654
C -3.386162 4.084282 -0.552464
C 0.867560 -1.021614 1.193513
C -0.245573 -2.046725 0.958697
C -0.098283 -2.803022 -0.370676
C -1.151266 -3.887274 -0.572892
C -2.602324 -3.400377 -0.569006
C -2.925683 -2.390770 -1.673278
C -4.420155 -2.092061 -1.824010
C -5.059920 -1.428170 -0.602953
C -6.524997 -1.060754 -0.832622
O 3.076276 -1.426016 -1.089118
O 5.666262 -0.910670 -1.124404
O -0.259833 -2.970843 2.058492
O 1.185440 -3.445902 -0.428062
H 6.288974 1.350323 0.160320
H 5.285806 0.343630 1.207842
H 4.458785 2.910614 -0.244861
H 4.547883 2.669019 1.489444
H 4.771957 0.843057 -1.752388
H 0.696343 -0.295335 -0.838179
H 2.349676 3.498956 1.477131

H -0.420749 3.541955 2.462451
H -0.143271 4.448047 0.971902
H -3.313705 2.146975 1.601428
H -1.417796 3.711121 -1.172522
H -3.667044 4.596935 -1.463447
H -4.139646 3.991815 0.219464
H 0.712416 -0.566090 2.173286
H 1.814850 -1.561030 1.251114
H -1.212486 -1.546770 0.971893
H -0.175698 -2.081409 -1.188151
H -0.934637 -4.372187 -1.529267
H -1.016965 -4.643240 0.204140
H -3.252262 -4.273715 -0.688911
H -2.845939 -2.977184 0.408264
H -2.397845 -1.452906 -1.482379
H -2.544683 -2.779701 -2.624172
H -4.563327 -1.438058 -2.691344
H -4.955643 -3.021989 -2.051102
H -4.988147 -2.096293 0.260066
H -4.487371 -0.532537 -0.345668
H -6.628004 -0.363347 -1.668537
H -7.121274 -1.946931 -1.067143
H -6.963120 -0.589409 0.050145
H 5.089209 -1.635062 -1.418464
H 0.514029 -3.540222 1.945795
H 1.849178 -2.767758 -0.647188

2b-c514, ΔG = 2.0513 kcal/mol, population = 0.35 %
C 3.285584 -0.431351 2.378007
C 2.931485 1.053347 2.325950
C 1.767460 1.317565 1.419335
C 1.344995 0.458245 0.452997
C 2.103302 -0.747346 0.195306
C 3.404937 -0.966743 0.961983
C 0.068551 0.682098 -0.340778
N 1.138353 2.539568 1.581627
C 0.240330 2.974731 0.663550
C -0.240640 2.151182 -0.310080
C -0.275359 4.376903 0.598037
C -1.003516 4.414949 -0.765761
C -1.065081 2.920463 -1.187763
O -1.757881 2.538980 -2.133231
O -2.322216 4.913022 -0.596622
C -0.203865 5.180279 -1.790391

C -0.675720 6.171896 -2.533244
C -1.067708 -0.212760 0.235076
C -2.044919 -0.755319 -0.801750
C -2.935528 -1.874321 -0.255202
C -2.196945 -3.088342 0.294990
C -1.165437 -3.715957 -0.644464
C -0.471678 -4.948048 -0.054728
C 0.396774 -4.644175 1.169231
C 1.201483 -5.842776 1.683162
C 2.318759 -6.294558 0.741710
O 1.756030 -1.593428 -0.629270
O 3.744679 -2.341823 0.951796
O -2.967130 0.231568 -1.284271
O -3.819243 -2.312829 -1.299080
H 4.222801 -0.577443 2.914787
H 2.504930 -0.986242 2.904055
H 3.788626 1.634237 1.966564
H 2.699521 1.429338 3.324763
H 4.177474 -0.390093 0.429372
H 0.250033 0.364113 -1.370786
H 1.473671 3.170748 2.295314
H -0.994080 4.564805 1.397643
H 0.524930 5.112758 0.680520
H -2.830663 4.653538 -1.378383
H 0.824848 4.849743 -1.902539
H -0.047348 6.673415 -3.257920
H -1.695722 6.521216 -2.433608
H -1.612726 0.333582 1.009237
H -0.588886 -1.061242 0.721627
H -1.473379 -1.153199 -1.648125
H -3.537346 -1.439902 0.554947
H -1.725161 -2.798069 1.235152
H -2.952375 -3.835680 0.557401
H -1.660162 -3.991709 -1.579086
H -0.397759 -2.981122 -0.898845
H -1.221250 -5.702037 0.213839
H 0.147739 -5.396768 -0.835840
H 1.084256 -3.827469 0.924679
H -0.236555 -4.277263 1.981387
H 1.640368 -5.582652 2.651109
H 0.520293 -6.679838 1.872041
H 2.882167 -7.126664 1.169667
H 1.929835 -6.623834 -0.223574
H 3.022160 -5.478461 0.555186

H 3.255624 -2.722355 0.202597
H -2.486310 0.950960 -1.737925
H -4.109543 -1.504814 -1.745910

2b-c204, ΔG = 2.0538 kcal/mol, population = 0.35 %
C 5.274751 0.996609 0.344779
C 4.324011 2.171074 0.556275
C 2.887168 1.744116 0.540533
C 2.445603 0.568179 0.006359
C 3.388065 -0.298414 -0.655091
C 4.817638 0.191066 -0.858784
C 0.998032 0.111435 0.106928
N 1.991401 2.642019 1.076444
C 0.649600 2.449959 0.944003
C 0.140696 1.301383 0.430167
C -0.374711 3.466438 1.339550
C -1.687494 2.871098 0.785639
C -1.291456 1.419303 0.372926
O -2.129351 0.575406 0.084623
O -2.670313 2.813043 1.809479
C -2.152439 3.612734 -0.441100
C -3.375610 4.093639 -0.617770
C 0.859322 -1.014767 1.165279
C -0.251621 -2.039403 0.918340
C -0.091210 -2.794421 -0.410225
C -1.142252 -3.878343 -0.623978
C -2.593219 -3.391264 -0.634216
C -2.905333 -2.380559 -1.740718
C -4.398173 -2.081425 -1.906094
C -5.049884 -1.418306 -0.690932
C -6.512515 -1.050348 -0.934847
O 3.090628 -1.417405 -1.095621
O 5.680926 -0.902684 -1.104533
O -0.276991 -2.964640 2.016999
O 1.192944 -3.437426 -0.455388
H 6.291351 1.356924 0.188527
H 5.277572 0.349453 1.225029
H 4.465636 2.918027 -0.233362
H 4.537398 2.674750 1.501515
H 4.793351 0.851885 -1.739685
H 0.708522 -0.286342 -0.867277
H 2.339668 3.505201 1.468161
H -0.440370 3.547662 2.426157
H -0.147961 4.455380 0.939455

H -3.324866 2.154214 1.535066
H -1.401102 3.721047 -1.218314
H -3.647284 4.607238 -1.531011
H -4.136854 4.000426 0.146415
H 0.694490 -0.560208 2.143916
H 1.805875 -1.554446 1.231789
H -1.218524 -1.539274 0.922411
H -0.160335 -2.072017 -1.227743
H -0.916098 -4.362406 -1.578575
H -1.015847 -4.635038 0.153668
H -3.242009 -4.264408 -0.761502
H -2.846600 -2.968981 0.340961
H -2.379277 -1.442968 -1.543657
H -2.514902 -2.768674 -2.688109
H -4.532585 -1.426734 -2.774310
H -4.931567 -3.011065 -2.139205
H -4.986855 -2.087123 0.172233
H -4.479681 -0.523034 -0.427287
H -6.607055 -0.352240 -1.771178
H -7.106648 -1.936186 -1.175995
H -6.959273 -0.579606 -0.056095
H 5.106674 -1.626639 -1.405079
H 0.497703 -3.534218 1.911235
H 1.858905 -2.759132 -0.667178

2b-c307, ΔG = 2.1015 kcal/mol, population = 0.32 %
C 2.723305 -0.681491 2.243984
C 2.249486 0.755706 2.433680
C 1.281494 1.174017 1.369064
C 1.104629 0.507181 0.187446
C 2.006388 -0.565623 -0.142375
C 3.165147 -0.867405 0.802420
C -0.013136 0.893449 -0.774251
N 0.588253 2.338131 1.614190
C -0.071356 2.972500 0.601364
C -0.292765 2.354286 -0.584751
C -0.585422 4.375142 0.681208
C -1.005146 4.671848 -0.777106
C -0.951594 3.274208 -1.472547
O -1.434899 3.073361 -2.577883
O -2.336139 5.164636 -0.812778
C -0.014944 5.585131 -1.453875
C -0.330174 6.694207 -2.108951
C -1.314031 0.091280 -0.525032

C -1.155704 -1.419808 -0.625357
C -2.483970 -2.172594 -0.394785
C -2.282426 -3.515925 0.309112
C -1.392243 -4.519938 -0.423479
C -1.081658 -5.775123 0.399415
C -0.309006 -5.521449 1.700261
C 1.017616 -4.783338 1.515327
C 1.796523 -4.627155 2.819454
O 1.913794 -1.249055 -1.173149
O 3.660544 -2.171553 0.571651
O -0.664615 -1.802936 -1.915049
O -3.197937 -2.303213 -1.629664
H 3.550190 -0.900056 2.919104
H 1.914616 -1.381931 2.464083
H 3.103851 1.442009 2.406749
H 1.779926 0.884404 3.411403
H 3.949468 -0.128578 0.574893
H 0.316986 0.707360 -1.797577
H 0.737278 2.817144 2.491042
H -1.461666 4.431225 1.329798
H 0.164667 5.068727 1.063106
H -2.668392 5.020373 -1.710766
H 1.019857 5.260692 -1.388530
H 0.432758 7.298485 -2.582696
H -1.353989 7.038743 -2.182095
H -2.067747 0.422065 -1.244255
H -1.683687 0.335527 0.475029
H -0.450438 -1.752965 0.141899
H -3.117186 -1.555751 0.247003
H -1.863472 -3.293020 1.295295
H -3.266463 -3.961683 0.487378
H -1.880687 -4.818059 -1.355323
H -0.457931 -4.040315 -0.717914
H -2.016685 -6.293469 0.637976
H -0.500453 -6.462735 -0.224195
H -0.932493 -4.960330 2.403903
H -0.113113 -6.485766 2.181134
H 1.629731 -5.319642 0.781886
H 0.837669 -3.792662 1.089946
H 1.213026 -4.069058 3.557103
H 2.034804 -5.601110 3.255308
H 2.731714 -4.088601 2.658038
H 3.337446 -2.423404 -0.309429
H 0.297640 -1.645093 -1.903699

H -2.522930 -2.444773 -2.309656

2b-c72, ΔG = 2.1480 kcal/mol, population = 0.30 %

C 3.933710 -0.179276 2.249865
C 2.679320 0.638285 2.543426
C 1.805091 0.777696 1.332973
C 2.237460 0.599617 0.054251
C 3.635616 0.315853 -0.179422
C 4.601077 0.369319 1.002020
C 1.298440 0.618641 -1.143017
N 0.491971 1.127717 1.588989
C -0.351817 1.437256 0.570163
C 0.011665 1.273244 -0.728008
C -1.734534 1.974250 0.766649
C -2.157383 2.373074 -0.665015
C -1.067748 1.715209 -1.567368
O -1.200404 1.615140 -2.780594
O -3.426909 1.823413 -0.980909
C -2.098296 3.868156 -0.851821
C -3.077140 4.608158 -1.354607
C 1.029956 -0.790923 -1.728799
C 0.557415 -1.809402 -0.694341
C -0.364869 -2.877662 -1.288563
C -0.940672 -3.828081 -0.238449
C -1.658828 -3.149987 0.933382
C -2.824862 -2.246635 0.531854
C -3.492961 -1.570383 1.727505
C -4.691050 -0.703678 1.341713
C -5.288191 0.052170 2.527196
O 4.089111 0.046081 -1.293596
O 5.786577 -0.338858 0.689582
O 1.657740 -2.422648 0.001870
O 0.302691 -3.616715 -2.324388
H 4.621264 -0.135421 3.094582
H 3.667689 -1.224785 2.079578
H 2.954152 1.641572 2.888931
H 2.102060 0.179852 3.349222
H 4.836562 1.433149 1.163431
H 1.763595 1.204203 -1.940674
H 0.192483 1.245984 2.545641
H -2.400879 1.196400 1.141600
H -1.756293 2.806211 1.471296
H -3.479730 1.772657 -1.946615
H -1.159224 4.326172 -0.554037

H -2.964807 5.678368 -1.471625
H -4.021815 4.170674 -1.651922
H 1.930722 -1.158055 -2.222865
H 0.271012 -0.681321 -2.504915
H -0.010929 -1.290395 0.077986
H -1.176493 -2.357847 -1.800443
H -1.627717 -4.508468 -0.749538
H -0.121249 -4.437399 0.154542
H -2.031632 -3.929984 1.604191
H -0.939816 -2.571788 1.521387
H -2.482949 -1.469498 -0.158301
H -3.566008 -2.836500 -0.019108
H -3.811012 -2.330713 2.449848
H -2.749823 -0.952932 2.246970
H -4.386717 0.009124 0.569851
H -5.458401 -1.338874 0.886770
H -4.550326 0.723998 2.974495
H -6.146428 0.656217 2.224837
H -5.622929 -0.636409 3.307770
H 5.792809 -0.409645 -0.280441
H 2.369427 -2.572108 -0.631293
H 0.941981 -4.202052 -1.898796

2b-c94, ΔG = 2.1511 kcal/mol, population = 0.30 %
C 5.015664 0.390598 1.372103
C 3.865641 1.274550 1.845118
C 2.657227 1.139316 0.967533
C 2.690237 0.661954 -0.307792
C 3.966459 0.319147 -0.896690
C 5.240198 0.622766 -0.110733
C 1.432312 0.425436 -1.130329
N 1.467387 1.562480 1.529402
C 0.333421 1.629580 0.783516
C 0.292350 1.166577 -0.492205
C -0.958592 2.195186 1.282999
C -1.843101 2.219368 0.016769
C -1.024150 1.390525 -1.021848
O -1.511552 1.004394 -2.076887
O -3.078676 1.565985 0.269178
C -2.011842 3.621904 -0.508307
C -3.173168 4.179353 -0.823074
C 1.094392 -1.078336 -1.275846
C 0.979360 -1.823858 0.054964
C -0.202127 -2.802830 0.100895

C -1.536798 -2.076864 0.162762
C -2.753637 -2.998731 0.142571
C -4.067589 -2.219513 0.177458
C -5.304757 -3.115647 0.138982
C -6.631891 -2.359982 0.263071
C -6.907030 -1.392134 -0.888392
O 4.075649 -0.187555 -2.014619
O 6.311365 -0.159966 -0.604836
O 2.198937 -2.519267 0.388800
O -0.082569 -3.631964 1.262172
H 5.923542 0.619749 1.930100
H 4.773315 -0.661932 1.535539
H 4.173902 2.326370 1.846972
H 3.589425 1.030636 2.873112
H 5.455480 1.690843 -0.273097
H 1.598647 0.807910 -2.140851
H 1.466512 1.896475 2.482023
H -1.405666 1.533458 2.027117
H -0.828020 3.177760 1.737985
H -3.423313 1.267337 -0.585058
H -1.084175 4.172003 -0.639197
H -3.221533 5.189980 -1.207804
H -4.108829 3.649823 -0.695271
H 1.862220 -1.553986 -1.887960
H 0.162102 -1.152708 -1.835963
H 0.845599 -1.123642 0.881367
H -0.166832 -3.435108 -0.799428
H -1.551204 -1.464957 1.070830
H -1.605514 -1.387114 -0.680186
H -2.722469 -3.617686 -0.761316
H -2.709969 -3.685086 0.992129
H -4.097676 -1.603129 1.083424
H -4.086329 -1.522724 -0.665811
H -5.304145 -3.691156 -0.794226
H -5.236020 -3.847587 0.950736
H -7.447243 -3.087368 0.321036
H -6.643515 -1.810920 1.210961
H -7.892997 -0.933006 -0.789236
H -6.171173 -0.586585 -0.923808
H -6.875823 -1.912080 -1.850017
H 6.036411 -0.436489 -1.495650
H 2.523995 -2.963908 -0.404394
H 0.866168 -3.802295 1.360024

2b-c85, ΔG = 2.2019 kcal/mol, population = 0.27 %

C 5.043297 0.233663 1.355737
C 3.957413 1.200251 1.820222
C 2.738305 1.136401 0.950101
C 2.730154 0.638119 -0.317491
C 3.974774 0.192730 -0.905351
C 5.271964 0.422335 -0.132880
C 1.453938 0.482011 -1.130988
N 1.584754 1.654263 1.509080
C 0.458842 1.801029 0.762895
C 0.379981 1.325683 -0.506534
C -0.783656 2.474664 1.253291
C -1.662557 2.555426 -0.014422
C -0.912189 1.651942 -1.042282
O -1.426889 1.296692 -2.095259
O -2.946803 2.005188 0.241124
C -1.718825 3.962493 -0.552324
C -2.832492 4.610774 -0.865217
C 0.997857 -0.992981 -1.240166
C 0.849254 -1.703563 0.106075
C -0.403735 -2.586682 0.186132
C -1.676832 -1.759556 0.279728
C -2.956391 -2.592890 0.289458
C -4.212075 -1.725611 0.387692
C -5.518998 -2.515277 0.493919
C -5.861218 -3.345579 -0.744101
C -7.211663 -4.050417 -0.627750
O 4.040065 -0.343464 -2.012840
O 6.283316 -0.441493 -0.617796
O 2.017093 -2.486522 0.432067
O -0.320911 -3.420518 1.347061
H 5.968442 0.412054 1.903798
H 4.733237 -0.797484 1.539929
H 4.336109 2.228762 1.806373
H 3.671341 0.989524 2.852712
H 5.558974 1.469781 -0.317380
H 1.645798 0.827197 -2.150293
H 1.610805 1.999628 2.457312
H -1.283150 1.859974 2.004386
H -0.575366 3.448993 1.696797
H -3.311793 1.728318 -0.611964
H -0.750084 4.433834 -0.692954
H -2.800270 5.619023 -1.257793
H -3.807485 4.160459 -0.727877

H 1.715882 -1.538233 -1.854764
H 0.052349 -1.006865 -1.783257
H 0.785906 -0.981716 0.922140
H -0.441556 -3.221612 -0.712183
H -1.622544 -1.152128 1.189250
H -1.713970 -1.062796 -0.560216
H -2.989788 -3.201061 -0.619337
H -2.931373 -3.290938 1.130900
H -4.119695 -1.074154 1.262869
H -4.261441 -1.060174 -0.482203
H -5.472160 -3.175349 1.368072
H -6.340365 -1.815795 0.683650
H -5.862798 -2.691022 -1.622649
H -5.080291 -4.090696 -0.918992
H -7.434673 -4.639210 -1.520217
H -7.226748 -4.727460 0.230624
H -8.021259 -3.328155 -0.492954
H 5.982331 -0.717987 -1.500145
H 2.296012 -2.963922 -0.359672
H 0.614941 -3.658859 1.425960

2b-c318, ΔG = 2.2126 kcal/mol, population = 0.27 %
C 4.624364 -0.239692 0.882592
C 4.173681 1.218762 0.888774
C 2.693675 1.350231 0.687415
C 1.907243 0.377417 0.152826
C 2.522396 -0.842840 -0.327580
C 4.045699 -0.936317 -0.336213
C 0.392739 0.486055 0.068724
N 2.146455 2.568706 1.048195
C 0.863801 2.867858 0.730928
C 0.020719 1.934841 0.206634
C 0.256783 4.224409 0.900863
C -1.094101 4.095525 0.160000
C -1.224235 2.567808 -0.090962
O -2.280453 2.057695 -0.470549
O -2.152760 4.531477 0.999028
C -1.056772 4.806510 -1.170039
C -1.946869 5.701545 -1.575377
C -0.281646 -0.428359 1.136294
C -1.464653 -1.246079 0.618303
C -1.808715 -2.404213 1.557120
C -2.891029 -3.336122 1.006530
C -2.590316 -3.968732 -0.355215

C -1.302841 -4.794728 -0.397603
C -1.141185 -5.615491 -1.682115
C -1.093346 -4.797512 -2.978303
C 0.073425 -3.812850 -3.045613
O 1.869801 -1.797542 -0.750060
O 4.446936 -2.292150 -0.401344
O -2.667356 -0.483446 0.484214
O -2.208840 -1.891115 2.838956
H 5.712601 -0.298753 0.867517
H 4.270998 -0.746985 1.783543
H 4.679424 1.773203 0.089864
H 4.452069 1.703587 1.826921
H 4.381894 -0.405011 -1.240636
H 0.085914 0.122985 -0.916651
H 2.747321 3.276791 1.444652
H 0.073094 4.437570 1.955433
H 0.894554 5.013284 0.501091
H -2.972761 4.159940 0.643089
H -0.228869 4.522170 -1.813323
H -1.864443 6.169068 -2.548083
H -2.775188 6.003541 -0.946890
H -0.590571 0.171146 1.994548
H 0.468508 -1.132279 1.498991
H -1.178635 -1.665781 -0.352857
H -0.896222 -2.971798 1.743984
H -3.045580 -4.125058 1.748629
H -3.824260 -2.770739 0.943705
H -3.432226 -4.615784 -0.622899
H -2.558934 -3.189381 -1.120537
H -0.435651 -4.141292 -0.269947
H -1.293289 -5.478902 0.457632
H -0.224288 -6.210288 -1.607924
H -1.968096 -6.330954 -1.746577
H -1.027285 -5.491684 -3.821626
H -2.035369 -4.256971 -3.108336
H 1.028956 -4.331925 -2.928289
H 0.092989 -3.292984 -4.006225
H 0.019487 -3.056565 -2.261635
H 3.671390 -2.773821 -0.736055
H -2.485696 0.372294 0.046944
H -2.861343 -1.201720 2.650627

2b-c174, ΔG = 2.2339 kcal/mol, population = 0.26 %
C 5.044684 0.223022 1.239733

C 3.987490 1.214303 1.717445
C 2.733807 1.138692 0.898445
C 2.669748 0.604701 -0.352846
C 3.885997 0.129755 -0.975863
C 5.215292 0.364921 -0.261512
C 1.361275 0.440694 -1.112365
N 1.609023 1.683296 1.489247
C 0.449569 1.806218 0.792220
C 0.312385 1.293662 -0.457473
C -0.770526 2.490253 1.322971
C -1.725501 2.502381 0.109151
C -1.008973 1.590065 -0.935881
O -1.568324 1.202776 -1.954305
O -2.968979 1.913090 0.464839
C -1.871068 3.886548 -0.467202
C -3.026992 4.483394 -0.724551
C 0.895917 -1.034095 -1.184423
C 0.792602 -1.721854 0.176961
C -0.442917 -2.624620 0.303847
C -1.726035 -1.815408 0.412069
C -2.996206 -2.662357 0.442951
C -4.257775 -1.804568 0.538255
C -5.559670 -2.606244 0.637099
C -5.843049 -3.541238 -0.544959
C -5.926894 -2.826707 -1.893586
O 3.902892 -0.435702 -2.070629
O 6.196584 -0.525384 -0.760385
O 1.981045 -2.479049 0.488064
O -0.317904 -3.434938 1.477590
H 5.992528 0.408723 1.744995
H 4.733555 -0.798889 1.468013
H 4.375241 2.237743 1.658353
H 3.740425 1.036859 2.766289
H 5.506806 1.402842 -0.488355
H 1.512653 0.771830 -2.143260
H 1.678187 2.055912 2.424857
H -1.216765 1.909861 2.132572
H -0.546308 3.486834 1.705455
H -3.383819 1.605587 -0.354125
H -0.932416 4.386850 -0.688624
H -3.059492 5.477935 -1.150600
H -3.972842 4.003456 -0.506803
H 1.587594 -1.593160 -1.816491
H -0.068930 -1.049202 -1.692028

H 0.736068 -0.984366 0.979881
H -0.491451 -3.276982 -0.581323
H -1.665515 -1.200479 1.316089
H -1.785082 -1.125655 -0.432057
H -3.031152 -3.279109 -0.460248
H -2.958759 -3.353031 1.290079
H -4.178930 -1.156802 1.417233
H -4.300729 -1.132424 -0.324768
H -5.541777 -3.198022 1.558596
H -6.395633 -1.905603 0.737983
H -5.075810 -4.319375 -0.592996
H -6.786698 -4.061314 -0.354727
H -6.203613 -3.520840 -2.690015
H -6.675989 -2.030274 -1.868871
H -4.972801 -2.373105 -2.169692
H 5.858596 -0.821326 -1.622788
H 2.253193 -2.960358 -0.303591
H 0.623563 -3.655981 1.537851

2b-c59, ΔG = 2.2496 kcal/mol, population = 0.25 %
C 5.028413 0.242106 1.212321
C 3.961671 1.191031 1.750316
C 2.688346 1.107064 0.962988
C 2.601279 0.600828 -0.297701
C 3.810515 0.179813 -0.968470
C 5.152914 0.437676 -0.287631
C 1.275224 0.415913 -1.020397
N 1.566370 1.606033 1.597745
C 0.390581 1.732643 0.929501
C 0.229758 1.240541 -0.326085
C -0.821542 2.404164 1.494797
C -1.781629 2.466160 0.285878
C -1.102365 1.541316 -0.772470
O -1.689814 1.158848 -1.776553
O -3.049887 1.933055 0.635250
C -1.863644 3.862213 -0.277621
C -2.992224 4.505679 -0.543619
C 0.827853 -1.062471 -1.131583
C 0.651593 -1.789188 0.195135
C -0.268355 -3.013547 0.087965
C -1.721562 -2.703605 -0.259280
C -2.429804 -1.725627 0.680119
C -3.870476 -1.420744 0.262636
C -4.814978 -2.623150 0.312487

C -6.286764 -2.263898 0.084254
C -6.576916 -1.684425 -1.300983
O 3.811543 -0.362983 -2.075656
O 6.145903 -0.404996 -0.843135
O 1.913744 -2.212242 0.756047
O -0.248701 -3.723450 1.333521
H 5.985564 0.433353 1.697635
H 4.745983 -0.793658 1.414470
H 4.322739 2.225363 1.714149
H 3.746629 0.974458 2.799008
H 5.405662 1.490712 -0.488445
H 1.393577 0.771197 -2.047871
H 1.654090 1.964832 2.537154
H -1.268030 1.796309 2.284092
H -0.588844 3.384224 1.913177
H -3.458894 1.610539 -0.181041
H -0.903422 4.327764 -0.481350
H -2.980922 5.505211 -0.958982
H -3.958072 4.059470 -0.343157
H 1.544237 -1.608512 -1.749032
H -0.117447 -1.063746 -1.674114
H 0.242744 -1.117408 0.950025
H 0.141225 -3.664762 -0.698480
H -1.766122 -2.321764 -1.282616
H -2.252735 -3.658013 -0.271585
H -2.418258 -2.125638 1.697542
H -1.882387 -0.781665 0.713403
H -4.264261 -0.639056 0.920050
H -3.860707 -0.996051 -0.746039
H -4.514372 -3.368076 -0.431256
H -4.714419 -3.107459 1.290030
H -6.895556 -3.161221 0.230675
H -6.604213 -1.549314 0.851546
H -6.249079 -2.371073 -2.086559
H -7.645807 -1.506780 -1.437722
H -6.062336 -0.734394 -1.456414
H 5.787663 -0.689696 -1.701314
H 2.431059 -2.633517 0.057401
H 0.669788 -3.688339 1.638285

2b-c20, ΔG = 2.2546 kcal/mol, population = 0.25 %
C 4.206005 -0.024493 2.126507
C 2.969789 0.809426 2.450792
C 2.019116 0.875433 1.292195

C 2.372134 0.621811 0.001749
C 3.755264 0.331723 -0.303007
C 4.791996 0.452186 0.810584
C 1.360761 0.565567 -1.134569
N 0.723555 1.238457 1.609946
C -0.191235 1.465317 0.631724
C 0.084863 1.208616 -0.672663
C -1.562438 2.010292 0.881869
C -2.085824 2.296843 -0.544082
C -1.057695 1.571201 -1.464946
O -1.275685 1.368090 -2.652758
O -3.373018 1.731373 -0.730582
C -2.040028 3.772950 -0.850210
C -3.047484 4.473411 -1.353008
C 1.102932 -0.870775 -1.652355
C 0.833243 -1.905843 -0.566291
C -0.035474 -3.068873 -1.070605
C -1.499847 -2.705742 -1.306070
C -2.269719 -2.257091 -0.063109
C -3.758793 -2.047550 -0.345708
C -4.619364 -1.911714 0.913219
C -4.268620 -0.718022 1.802571
C -5.202714 -0.581771 3.003372
O 4.140129 0.006014 -1.427919
O 5.958550 -0.272216 0.465561
O 2.060945 -2.440097 -0.023401
O 0.013622 -4.145907 -0.128458
H 4.944399 0.066916 2.923110
H 3.932697 -1.077591 2.031594
H 3.261344 1.831660 2.718392
H 2.446840 0.399381 3.317330
H 5.032733 1.523573 0.896224
H 1.759925 1.129495 -1.982408
H 0.485212 1.412488 2.575196
H -2.196252 1.264214 1.362163
H -1.539772 2.895057 1.519315
H -3.480568 1.581926 -1.681372
H -1.086397 4.250065 -0.642497
H -2.944418 5.530949 -1.559674
H -4.006356 4.015643 -1.561175
H 1.960277 -1.198956 -2.243633
H 0.256100 -0.820172 -2.336780
H 0.341721 -1.448911 0.293314
H 0.393412 -3.411281 -2.023976

H -1.556436 -1.934719 -2.078810
H -1.988057 -3.590501 -1.725371
H -2.151304 -3.010580 0.719871
H -1.841136 -1.332053 0.328528
H -3.887399 -1.160489 -0.974474
H -4.129161 -2.897308 -0.928994
H -5.670843 -1.830542 0.616524
H -4.538169 -2.833523 1.501534
H -3.238764 -0.817534 2.159004
H -4.300861 0.194075 1.200448
H -6.238372 -0.445848 2.680344
H -5.167370 -1.475500 3.632244
H -4.932362 0.274454 3.625477
H 5.907969 -0.393815 -0.498018
H 2.680626 -2.581418 -0.750721
H 0.917680 -4.141000 0.220176

2b-c470, ΔG = 2.2841 kcal/mol, population = 0.24 %
C 2.963320 -0.958050 2.680277
C 1.801574 0.021314 2.810095
C 1.322125 0.503293 1.473080
C 2.065680 0.451931 0.330799
C 3.433725 -0.013703 0.410162
C 4.020648 -0.346353 1.780867
C 1.527138 0.877924 -1.032043
N 0.047340 1.029225 1.462609
C -0.477458 1.569356 0.326339
C 0.195628 1.546936 -0.846599
C -1.831922 2.198861 0.252896
C -2.045370 2.405061 -1.271672
C -0.613055 2.165479 -1.870568
O -0.306091 2.403951 -3.028173
O -2.907375 1.409854 -1.815961
C -2.567021 3.768221 -1.597394
C -3.589951 4.021734 -2.403144
C 1.445399 -0.276611 -2.066207
C 0.863400 -1.605366 -1.588732
C -0.568572 -1.551040 -1.061290
C -1.061072 -2.909262 -0.570140
C -2.469109 -2.877422 0.030654
C -2.666507 -1.901868 1.195441
C -1.720947 -2.124667 2.376695
C -2.033266 -1.247544 3.593694
C -3.327555 -1.622768 4.316327

O 4.161623 -0.129936 -0.577629
O 5.137630 -1.203298 1.632726
O 0.840949 -2.535657 -2.688469
O -1.457553 -0.989038 -2.044297
H 3.383853 -1.179621 3.661127
H 2.617397 -1.896352 2.239928
H 2.105513 0.892031 3.402081
H 0.967944 -0.439719 3.342985
H 4.343753 0.612344 2.217042
H 2.230158 1.597713 -1.463354
H -0.472560 1.071105 2.326614
H -2.610443 1.555407 0.664618
H -1.849652 3.139624 0.807261
H -2.422478 0.562343 -1.879749
H -2.024783 4.583285 -1.127576
H -3.907243 5.038176 -2.599167
H -4.138413 3.220914 -2.880908
H 2.458276 -0.477253 -2.418115
H 0.865466 0.076220 -2.918964
H 1.487964 -2.008854 -0.784144
H -0.593093 -0.850981 -0.232490
H -1.035975 -3.618628 -1.399659
H -0.343126 -3.273544 0.170149
H -3.185048 -2.632126 -0.757448
H -2.716990 -3.887823 0.370463
H -2.558112 -0.872080 0.841038
H -3.701787 -1.988534 1.533782
H -1.756013 -3.178187 2.676780
H -0.690939 -1.938480 2.059540
H -1.202064 -1.310218 4.302184
H -2.093825 -0.199239 3.279635
H -4.199454 -1.510491 3.669597
H -3.294148 -2.662234 4.652800
H -3.484595 -0.991820 5.193484
H 5.433340 -1.082528 0.714405
H 1.672211 -2.457588 -3.170779
H -1.315773 -1.461148 -2.875949

2b-c148, ΔG = 2.2892 kcal/mol, population = 0.24 %
C 2.935082 -0.292154 2.926106
C 2.505016 1.160910 2.745604
C 1.579387 1.338041 1.578829
C 1.426720 0.428142 0.577739
C 2.262005 -0.756085 0.572651

C 3.398554 -0.840173 1.586967
C 0.416129 0.588523 -0.548153
N 0.881775 2.532117 1.555174
C 0.106605 2.862497 0.493720
C -0.094918 2.001040 -0.539735
C -0.615506 4.166277 0.364021
C -1.208349 4.098590 -1.061646
C -0.983047 2.617474 -1.476316
O -1.551693 2.122211 -2.450930
O -2.600337 4.373448 -1.022667
C -0.451897 4.991675 -2.013831
C -1.007792 5.904590 -2.798265
C -0.760012 -0.427006 -0.440505
C -0.929883 -1.311406 -1.669048
C -2.205405 -2.157204 -1.595452
C -2.278693 -3.105355 -0.405545
C -1.104000 -4.074156 -0.258204
C -1.260737 -5.026450 0.932265
C -1.263298 -4.343166 2.306668
C -0.004252 -3.528793 2.627181
C 1.279527 -4.356971 2.635195
O 2.113506 -1.678852 -0.229031
O 3.867036 -2.169960 1.691041
O -0.953619 -0.569623 -2.900462
O -2.330690 -2.919928 -2.804515
H 3.740115 -0.360206 3.657641
H 2.097799 -0.893383 3.288368
H 3.383811 1.795572 2.582926
H 2.016253 1.533955 3.648242
H 4.204979 -0.196735 1.202436
H 0.929574 0.395188 -1.492883
H 0.994469 3.181801 2.319927
H -1.426612 4.232780 1.091335
H 0.047379 5.018566 0.516820
H -2.985955 4.007052 -1.831689
H 0.622296 4.830836 -2.031129
H -0.405664 6.509288 -3.464112
H -2.075256 6.084962 -2.790293
H -1.688611 0.118866 -0.250045
H -0.599759 -1.067765 0.425585
H -0.066359 -1.972573 -1.749786
H -3.052860 -1.459239 -1.531899
H -2.385532 -2.499271 0.496415
H -3.207691 -3.677377 -0.494673

H -1.009819 -4.660074 -1.175360
H -0.168897 -3.518184 -0.155776
H -2.194365 -5.587687 0.819294
H -0.457676 -5.767690 0.898445
H -2.138301 -3.692340 2.390460
H -1.387246 -5.113240 3.075594
H 0.100903 -2.708286 1.911954
H -0.135855 -3.057589 3.605798
H 1.497230 -4.767065 1.646838
H 2.136861 -3.751276 2.930029
H 1.196072 -5.196812 3.330791
H 3.488360 -2.639660 0.928209
H -1.295165 0.333633 -2.743080
H -2.091172 -2.307223 -3.515003

2b-c46, ΔG = 2.3042 kcal/mol, population = 0.23 %
C 4.921150 0.279103 1.547739
C 3.771521 1.158647 2.030444
C 2.589368 1.093890 1.110449
C 2.652671 0.683064 -0.186132
C 3.940234 0.344943 -0.752131
C 5.194847 0.581347 0.085992
C 1.418206 0.522076 -1.061744
N 1.391104 1.510858 1.658828
C 0.278547 1.628081 0.888856
C 0.265878 1.232480 -0.409852
C -1.020078 2.178175 1.387329
C -1.906659 2.201347 0.122934
C -1.040901 1.473652 -0.954466
O -1.495277 1.151806 -2.045399
O -3.084640 1.437618 0.350686
C -2.193808 3.606688 -0.334488
C -3.400520 4.085616 -0.605204
C 1.060608 -0.960006 -1.329642
C 0.850092 -1.794020 -0.067609
C -0.272270 -2.832059 -0.215865
C -1.649403 -2.185999 -0.222591
C -2.788093 -3.168774 -0.493733
C -4.164545 -2.501035 -0.569594
C -4.642683 -1.905354 0.757246
C -6.084675 -1.387712 0.723871
C -6.290627 -0.166374 -0.173245
O 4.075324 -0.105941 -1.891236
O 6.265204 -0.197803 -0.415837

O 2.065711 -2.451409 0.348893
O -0.211823 -3.753866 0.878428
H 5.814920 0.463421 2.143833
H 4.656425 -0.775812 1.650024
H 4.097797 2.202795 2.098981
H 3.457930 0.864840 3.034319
H 5.435986 1.651454 -0.015298
H 1.623115 0.972501 -2.036856
H 1.367525 1.796272 2.626768
H -1.461941 1.515935 2.133960
H -0.895548 3.161950 1.842162
H -3.420806 1.162818 -0.514540
H -1.314567 4.233170 -0.456497
H -3.533359 5.105241 -0.943413
H -4.290409 3.480251 -0.487146
H 1.850399 -1.412790 -1.931555
H 0.159524 -0.970020 -1.942933
H 0.590805 -1.156755 0.780376
H -0.116352 -3.380250 -1.157585
H -1.791039 -1.691134 0.741789
H -1.675062 -1.402855 -0.983170
H -2.587988 -3.681591 -1.440403
H -2.799165 -3.939898 0.281569
H -4.140221 -1.725978 -1.342257
H -4.898926 -3.242510 -0.902460
H -4.561302 -2.676551 1.531167
H -3.979677 -1.090304 1.060803
H -6.747511 -2.196607 0.397280
H -6.392001 -1.133311 1.742795
H -5.668978 0.668333 0.160181
H -6.035596 -0.379428 -1.213239
H -7.330509 0.166992 -0.152046
H 6.011334 -0.424042 -1.326869
H 2.501264 -2.810872 -0.434396
H 0.732326 -3.876365 1.058802

2b-c184, ΔG = 2.3074 kcal/mol, population = 0.23 %
C 2.573809 -0.660380 2.982123
C 1.452112 0.370062 2.932946
C 1.050775 0.696854 1.525556
C 1.812544 0.436374 0.426065
C 3.168914 -0.037936 0.628839
C 3.685197 -0.204946 2.058141
C 1.340748 0.704073 -1.004296

N -0.177392 1.316650 1.402195
C -0.659611 1.681949 0.189003
C 0.011372 1.412016 -0.962260
C -1.994253 2.330607 0.002560
C -2.041108 2.603231 -1.515664
C -0.830202 1.792545 -2.061316
O -0.739457 1.495644 -3.251510
O -3.237916 2.081799 -2.070956
C -1.829423 4.064030 -1.825567
C -2.611184 4.790026 -2.613023
C 1.356204 -0.561566 -1.899743
C 0.508164 -1.752655 -1.452871
C -0.999761 -1.590756 -1.691648
C -1.822958 -2.780675 -1.206328
C -1.625914 -3.140488 0.268396
C -1.998146 -2.029954 1.251378
C -1.712758 -2.401877 2.705018
C -2.187521 -1.353511 3.709827
C -1.805464 -1.687178 5.150078
O 3.950364 -0.268029 -0.294843
O 4.777967 -1.104573 2.074081
O 0.968060 -2.939611 -2.118691
O -1.252586 -1.451500 -3.102865
H 2.946867 -0.766459 4.000638
H 2.206532 -1.635112 2.652267
H 1.766376 1.296076 3.427797
H 0.575839 0.010960 3.476022
H 4.021523 0.794653 2.377673
H 2.070010 1.377458 -1.468348
H -0.719623 1.502300 2.232837
H -2.797679 1.642356 0.272260
H -2.099197 3.229466 0.611316
H -3.062873 1.913328 -3.008869
H -0.953177 4.505987 -1.359773
H -2.397937 5.834350 -2.801430
H -3.491473 4.368200 -3.081304
H 2.385515 -0.907996 -1.941550
H 1.079121 -0.264655 -2.912042
H 0.681466 -1.929462 -0.390646
H -1.340600 -0.692216 -1.178548
H -2.875373 -2.547927 -1.391540
H -1.573706 -3.644013 -1.826761
H -2.233580 -4.022858 0.490004
H -0.588809 -3.443698 0.439154

H -1.448041 -1.116354 1.013728
H -3.059461 -1.784479 1.133292
H -2.189181 -3.360821 2.937787
H -0.634904 -2.559411 2.827039
H -1.771093 -0.377168 3.441172
H -3.273980 -1.245955 3.630709
H -0.719875 -1.753242 5.261997
H -2.167436 -0.928114 5.846643
H -2.227647 -2.648551 5.453663
H 5.135447 -1.091051 1.170296
H 0.621645 -2.892008 -3.020951
H -1.041835 -0.540515 -3.359062

2b-c576, ΔG = 2.3218 kcal/mol, population = 0.22 %
C 5.393967 0.878202 0.247039
C 4.523747 2.108682 0.487297
C 3.063750 1.769918 0.501115
C 2.542596 0.630442 -0.038421
C 3.413010 -0.284845 -0.732118
C 4.867448 0.112891 -0.954812
C 1.075830 0.257019 0.093227
N 2.231755 2.710327 1.068118
C 0.878708 2.602373 0.951490
C 0.294261 1.492129 0.433803
C -0.077510 3.679588 1.356505
C -1.427544 3.173240 0.802383
C -1.126735 1.703350 0.371879
O -2.014936 0.919475 0.063932
O -2.407253 3.168965 1.830831
C -1.847709 3.957041 -0.414454
C -3.037066 4.519108 -0.582078
C 0.905203 -0.863344 1.152040
C -0.301646 -1.786249 0.966493
C -0.318761 -2.484037 -0.401342
C -1.469935 -3.467974 -0.566447
C -2.852323 -2.847023 -0.372863
C -3.982059 -3.796130 -0.770626
C -5.383399 -3.264764 -0.450326
C -5.740096 -1.922449 -1.100196
C -5.673340 -1.934584 -2.627127
O 3.031142 -1.374036 -1.181928
O 5.655170 -1.032597 -1.220375
O -0.311106 -2.755757 2.026038
O 0.902945 -3.211986 -0.601875

H 6.428488 1.175206 0.075926
H 5.370820 0.223794 1.121587
H 4.693460 2.851678 -0.300408
H 4.788939 2.590454 1.430743
H 4.873466 0.779685 -1.831549
H 0.744517 -0.120311 -0.875300
H 2.636429 3.546779 1.463645
H -0.134986 3.760019 2.443622
H 0.210984 4.653869 0.960104
H -3.111608 2.567469 1.548877
H -1.093160 4.025115 -1.193076
H -3.276488 5.059012 -1.489175
H -3.801648 4.468623 0.182887
H 0.843367 -0.406730 2.141553
H 1.803770 -1.483113 1.152818
H -1.222048 -1.215467 1.073712
H -0.418992 -1.716147 -1.175089
H -1.396290 -3.888770 -1.573464
H -1.333568 -4.295989 0.133944
H -2.984669 -2.555883 0.673311
H -2.919827 -1.925252 -0.957592
H -3.902527 -4.022178 -1.838706
H -3.845245 -4.750008 -0.250496
H -6.122080 -4.012778 -0.758378
H -5.481783 -3.165426 0.636022
H -6.751470 -1.646342 -0.787083
H -5.081881 -1.138513 -0.714387
H -6.311980 -2.719816 -3.041186
H -6.005019 -0.980493 -3.042364
H -4.657029 -2.112786 -2.984484
H 5.032720 -1.714218 -1.523470
H 0.405855 -3.376656 1.836521
H 1.612768 -2.576188 -0.803988

2b-c97, ΔG = 2.3243 kcal/mol, population = 0.22 %
C 5.217744 0.338843 0.986494
C 4.172676 1.288958 1.563510
C 2.847183 1.150141 0.877845
C 2.674651 0.568616 -0.341665
C 3.836258 0.145260 -1.088664
C 5.221000 0.462739 -0.526243
C 1.299194 0.333329 -0.948020
N 1.769513 1.682994 1.555313
C 0.555051 1.786156 0.955468

C 0.312114 1.229404 -0.258993
C -0.611117 2.505661 1.555946
C -1.678765 2.447727 0.440598
C -1.038258 1.524556 -0.645167
O -1.670044 1.121924 -1.614445
O -2.851815 1.814811 0.940582
C -1.944013 3.805792 -0.150987
C -3.144373 4.350973 -0.294502
C 0.844486 -1.150148 -0.901405
C 0.460244 -1.680919 0.471571
C -0.191124 -3.068794 0.421064
C -1.505986 -3.133860 -0.349173
C -2.538403 -2.070012 0.024550
C -3.885981 -2.296256 -0.658360
C -4.874115 -1.154535 -0.418615
C -6.249237 -1.359098 -1.061546
C -7.051084 -2.515094 -0.462314
O 3.765163 -0.441559 -2.171338
O 6.187984 -0.387065 -1.116047
O 1.576894 -1.730203 1.384928
O -0.439775 -3.508534 1.764016
H 6.204532 0.574158 1.385234
H 4.981394 -0.692526 1.259171
H 4.508496 2.327243 1.459893
H 4.039484 1.112009 2.633218
H 5.433090 1.509380 -0.796079
H 1.351984 0.592420 -2.008480
H 1.918191 2.094968 2.464877
H -0.976515 1.982468 2.441546
H -0.352475 3.523829 1.849672
H -3.355023 1.495000 0.177839
H -1.056542 4.334039 -0.488407
H -3.263998 5.329485 -0.741545
H -4.040623 3.844109 0.040417
H 1.630601 -1.773580 -1.333786
H -0.022462 -1.237594 -1.556989
H -0.239720 -0.999704 0.955024
H 0.523877 -3.758409 -0.050484
H -1.290804 -3.074540 -1.419165
H -1.926002 -4.129990 -0.180139
H -2.676433 -2.056407 1.109225
H -2.168550 -1.081981 -0.256714
H -3.732038 -2.410210 -1.737850
H -4.309529 -3.241764 -0.307104

H -4.998639 -1.001515 0.659693
H -4.436122 -0.232136 -0.815420
H -6.826146 -0.435218 -0.958047
H -6.121219 -1.520749 -2.137318
H -8.045233 -2.577573 -0.910135
H -6.558688 -3.476323 -0.621010
H -7.178148 -2.381583 0.615598
H 5.774610 -0.714543 -1.933117
H 2.314583 -2.169450 0.941965
H 0.333794 -3.232634 2.276740

2b-c402, ΔG = 2.3255 kcal/mol, population = 0.22 %
C 5.397489 0.866127 0.192826
C 4.514159 2.088523 0.425758
C 3.058121 1.734386 0.460685
C 2.541545 0.580769 -0.053439
C 3.416750 -0.337616 -0.736904
C 4.862276 0.076069 -0.988802
C 1.077015 0.199711 0.095176
N 2.223804 2.678445 1.017244
C 0.871384 2.562177 0.905439
C 0.290810 1.439415 0.410591
C -0.087907 3.643221 1.292504
C -1.432324 3.127897 0.732673
C -1.131388 1.644700 0.352900
O -2.020433 0.849198 0.080391
O -2.432840 3.163161 1.739049
C -1.822066 3.873237 -0.518869
C -3.005378 4.432487 -0.731906
C 0.913694 -0.895550 1.181175
C -0.267303 -1.854183 1.002520
C -0.220184 -2.623521 -0.326231
C -1.325553 -3.665704 -0.465346
C -2.756342 -3.133791 -0.353218
C -3.133166 -2.112209 -1.428827
C -4.577679 -1.613258 -1.333732
C -5.643355 -2.674086 -1.614479
C -7.061642 -2.106564 -1.588071
O 3.046783 -1.442280 -1.158013
O 5.661198 -1.062108 -1.250458
O -0.290791 -2.766088 2.112133
O 1.029165 -3.322170 -0.448004
H 6.425157 1.174039 0.001029
H 5.396749 0.225126 1.077743

H 4.665630 2.821873 -0.374800
H 4.784981 2.587248 1.358498
H 4.844361 0.730260 -1.874834
H 0.743007 -0.202618 -0.862523
H 2.624463 3.523893 1.397318
H -0.154783 3.732507 2.378356
H 0.202803 4.613977 0.889330
H -3.123296 2.538379 1.473191
H -1.049408 3.914753 -1.281508
H -3.221915 4.943543 -1.661219
H -3.787523 4.408037 0.016386
H 0.822671 -0.414412 2.156617
H 1.826273 -1.493979 1.215073
H -1.202962 -1.299462 1.050674
H -0.310575 -1.902271 -1.142467
H -1.193499 -4.148899 -1.437829
H -1.163740 -4.434055 0.294016
H -3.432863 -3.990211 -0.408049
H -2.913113 -2.689160 0.634652
H -2.469301 -1.249044 -1.353435
H -2.966753 -2.554961 -2.418344
H -4.745674 -1.188124 -0.337267
H -4.712948 -0.788293 -2.041518
H -5.446621 -3.126125 -2.592909
H -5.566690 -3.482864 -0.882710
H -7.290127 -1.671290 -0.611472
H -7.182988 -1.319105 -2.336783
H -7.807025 -2.878615 -1.791411
H 5.043370 -1.757062 -1.532081
H 0.446231 -3.378457 1.980754
H 1.716716 -2.676056 -0.690439

2b-c10, ΔG = 2.3412 kcal/mol, population = 0.22 %
C 5.107918 0.483489 0.985574
C 4.015275 1.353236 1.599766
C 2.693129 1.159058 0.921354
C 2.542565 0.608113 -0.315901
C 3.720083 0.278108 -1.085488
C 5.089897 0.654006 -0.522351
C 1.177962 0.321576 -0.924902
N 1.594171 1.608419 1.623882
C 0.370585 1.661664 1.036023
C 0.149761 1.134190 -0.195241
C -0.829591 2.295552 1.665511

C -1.896208 2.226558 0.550266
C -1.214585 1.374636 -0.568549
O -1.824395 0.980890 -1.555171
O -3.037799 1.524613 1.029561
C -2.226319 3.590035 0.005257
C -3.451533 4.081515 -0.122190
C 0.806575 -1.186106 -0.941710
C 0.428620 -1.789708 0.401716
C -0.065099 -3.238121 0.288566
C -1.354535 -3.421886 -0.506234
C -2.512596 -2.515556 -0.086474
C -3.849828 -2.951960 -0.688577
C -5.001153 -1.990833 -0.374059
C -4.969485 -0.702277 -1.198222
C -6.008629 0.323133 -0.746762
O 3.672368 -0.273222 -2.187906
O 6.097769 -0.121168 -1.145592
O 1.507963 -1.748399 1.359458
O -0.283007 -3.755353 1.608817
H 6.083054 0.763868 1.383972
H 4.935424 -0.567907 1.228025
H 4.289500 2.411757 1.523977
H 3.903351 1.137732 2.664556
H 5.240773 1.718892 -0.760098
H 1.209575 0.627962 -1.973617
H 1.726569 1.994015 2.547534
H -1.164814 1.720457 2.530582
H -0.624524 3.314043 1.998255
H -3.553612 1.251485 0.257682
H -1.365710 4.170878 -0.314430
H -3.617299 5.067132 -0.537690
H -4.323761 3.523290 0.194109
H 1.635235 -1.746302 -1.381568
H -0.044060 -1.301826 -1.614546
H -0.356182 -1.197128 0.871551
H 0.729183 -3.825729 -0.194536
H -1.140512 -3.282316 -1.568920
H -1.649130 -4.469403 -0.391489
H -2.598959 -2.516342 1.003595
H -2.298887 -1.486429 -0.382531
H -3.748709 -3.051886 -1.775680
H -4.092139 -3.950396 -0.311217
H -5.958322 -2.493688 -0.545462
H -4.974591 -1.739776 0.692859

H -3.970529 -0.260246 -1.157899
H -5.135458 -0.950961 -2.251610
H -7.020773 -0.082033 -0.824407
H -5.854754 0.608568 0.298209
H -5.965570 1.231538 -1.351716
H 5.696881 -0.445598 -1.970036
H 2.310145 -2.076553 0.933132
H 0.439655 -3.403211 2.148696

2b-c15, ΔG = 2.3488 kcal/mol, population = 0.21 %
C 5.003090 0.312516 0.941369
C 3.935911 1.185917 1.594551
C 2.611307 1.063549 0.904405
C 2.450358 0.585373 -0.360188
C 3.619202 0.245612 -1.137654
C 4.997550 0.556025 -0.557066
C 1.078842 0.350142 -0.973709
N 1.519194 1.496064 1.632586
C 0.302466 1.625603 1.041177
C 0.075434 1.167614 -0.216631
C -0.880461 2.272695 1.689293
C -1.917182 2.339933 0.544737
C -1.268612 1.494246 -0.596831
O -1.883096 1.178481 -1.608076
O -3.127802 1.716209 0.953174
C -2.112969 3.751761 0.058012
C -3.288395 4.348869 -0.084568
C 0.659418 -1.142267 -1.014888
C 0.538286 -1.830092 0.338233
C -0.228621 -3.159341 0.264911
C -1.716644 -3.033067 -0.049172
C -2.503743 -2.122261 0.898268
C -4.012079 -2.399686 0.886261
C -4.698329 -2.248904 -0.474998
C -4.664366 -0.829936 -1.039734
C -5.384376 -0.702603 -2.380516
O 3.557216 -0.270458 -2.256151
O 5.983793 -0.218823 -1.214240
O 1.826799 -2.079650 0.942808
O -0.101211 -3.839100 1.520523
H 5.985110 0.540904 1.355598
H 4.792662 -0.742629 1.131019
H 4.242801 2.237964 1.571456
H 3.814553 0.921521 2.647282

H 5.181133 1.625767 -0.744077
H 1.109418 0.680800 -2.014938
H 1.660824 1.834639 2.573128
H -1.268097 1.649131 2.496912
H -0.635294 3.250558 2.105537
H -3.601303 1.450059 0.151681
H -1.195711 4.273273 -0.201076
H -3.358363 5.364343 -0.452767
H -4.212643 3.847629 0.173752
H 1.367091 -1.694048 -1.638660
H -0.305921 -1.187228 -1.521320
H 0.033382 -1.180339 1.053481
H 0.240461 -3.772214 -0.518704
H -1.834833 -2.705783 -1.084082
H -2.130666 -4.045109 -0.004437
H -2.136020 -2.269582 1.916227
H -2.319310 -1.073708 0.653399
H -4.177846 -3.418449 1.252266
H -4.499294 -1.731626 1.604438
H -4.242673 -2.933263 -1.198226
H -5.742157 -2.565919 -0.378147
H -5.127585 -0.151934 -0.312605
H -3.629758 -0.503069 -1.161752
H -6.436972 -0.984627 -2.292686
H -5.342408 0.320720 -2.759815
H -4.928185 -1.354604 -3.130199
H 5.570408 -0.504025 -2.047101
H 2.410551 -2.459902 0.273555
H 0.797937 -3.648901 1.826895

2b-c82, ΔG = 2.3563 kcal/mol, population = 0.21 %
C 4.773904 0.189755 1.710168
C 3.627928 1.097936 2.146359
C 2.488266 1.071680 1.172276
C 2.599207 0.661522 -0.121204
C 3.900846 0.289779 -0.630040
C 5.122168 0.491486 0.264095
C 1.402001 0.536356 -1.051940
N 1.278645 1.524234 1.664758
C 0.210711 1.685172 0.840905
C 0.247200 1.291691 -0.458460
C -1.091199 2.282069 1.274803
C -1.877325 2.406641 -0.048739
C -1.019431 1.591963 -1.066467

O -1.445366 1.274849 -2.169958
O -3.154302 1.798052 0.079090
C -1.948730 3.839068 -0.512201
C -3.060576 4.465581 -0.872394
C 1.004328 -0.933079 -1.331517
C 0.671065 -1.742527 -0.081204
C -0.422144 -2.793919 -0.314196
C -1.792562 -2.169993 -0.531856
C -2.895036 -3.198922 -0.780094
C -4.233861 -2.572557 -1.185041
C -4.812876 -1.566020 -0.185080
C -5.067111 -2.133029 1.211600
C -5.713346 -1.113376 2.148045
O 4.074819 -0.162940 -1.763284
O 6.193813 -0.313357 -0.192505
O 1.840924 -2.381570 0.473119
O -0.494037 -3.652681 0.830158
H 5.644326 0.345371 2.347544
H 4.475145 -0.857745 1.792918
H 3.979084 2.132119 2.238752
H 3.261004 0.805988 3.132578
H 5.395281 1.555245 0.179362
H 1.668395 0.973127 -2.018340
H 1.217621 1.807149 2.631901
H -1.618526 1.606804 1.951158
H -0.955064 3.235350 1.787013
H -3.451376 1.572984 -0.814752
H -0.991193 4.350486 -0.554381
H -3.038226 5.494716 -1.207099
H -4.025179 3.975557 -0.832568
H 1.813057 -1.425359 -1.874420
H 0.146928 -0.911214 -2.003618
H 0.321828 -1.089015 0.720894
H -0.147620 -3.391827 -1.196732
H -2.031008 -1.558565 0.343273
H -1.744847 -1.488519 -1.384358
H -2.572501 -3.876787 -1.577277
H -3.022200 -3.816421 0.111008
H -4.110538 -2.072612 -2.151774
H -4.961979 -3.374928 -1.344923
H -4.146696 -0.702771 -0.099444
H -5.756070 -1.179928 -0.586293
H -5.707304 -3.018523 1.131558
H -4.124772 -2.475640 1.648365

H -5.078650 -0.230280 2.259780
H -6.678155 -0.778924 1.757509
H -5.882132 -1.531515 3.142828
H 5.976013 -0.529161 -1.115332
H 2.346483 -2.772966 -0.250678
H 0.422593 -3.768434 1.121915

2b-c119, ΔG = 2.3613 kcal/mol, population = 0.21 %
C 4.473627 0.493392 2.437204
C 3.097033 1.107803 2.675466
C 2.164114 0.853719 1.530179
C 2.566605 0.486357 0.282348
C 3.982849 0.422382 -0.014370
C 4.966790 0.888964 1.056437
C 1.567485 0.084711 -0.793125
N 0.819650 1.068162 1.793310
C -0.063387 1.172718 0.769122
C 0.280130 0.794148 -0.493014
C -1.441028 1.743240 0.885816
C -1.858307 1.940057 -0.593101
C -0.767890 1.169026 -1.385552
O -0.878605 0.925357 -2.588784
O -3.121134 1.337598 -0.828236
C -1.810865 3.396610 -0.981119
C -2.799797 4.055343 -1.568993
C 1.473209 -1.463155 -0.818501
C 0.460250 -2.111143 -1.774958
C -0.974069 -2.233132 -1.227523
C -1.078982 -3.172940 -0.035345
C -2.479386 -3.260831 0.576772
C -2.969819 -1.954307 1.204592
C -4.243745 -2.140953 2.030037
C -4.766232 -0.859265 2.686605
C -5.352405 0.150732 1.699423
O 4.427423 0.034254 -1.094566
O 6.254211 0.362568 0.795171
O 0.441192 -1.487191 -3.068021
O -1.820930 -2.741649 -2.268652
H 5.176014 0.834284 3.197613
H 4.415502 -0.596178 2.495305
H 3.184916 2.191528 2.813996
H 2.652498 0.713928 3.592126
H 4.993805 1.988258 0.991165
H 1.945478 0.412259 -1.763187

H 0.547092 1.355030 2.722658
H -2.115755 1.029560 1.360758
H -1.459627 2.666890 1.464285
H -3.204777 1.202801 -1.783149
H -0.872900 3.895747 -0.755585
H -2.696033 5.100679 -1.829669
H -3.744080 3.577477 -1.797589
H 1.278286 -1.812298 0.196022
H 2.467891 -1.823397 -1.083583
H 0.802895 -3.130171 -1.972603
H -1.334388 -1.243205 -0.937148
H -0.751525 -4.166073 -0.357927
H -0.384390 -2.844732 0.741009
H -3.192852 -3.592703 -0.182192
H -2.463883 -4.038064 1.347538
H -2.183764 -1.550934 1.855105
H -3.136741 -1.206975 0.425205
H -5.029797 -2.565262 1.394198
H -4.045317 -2.885565 2.808154
H -5.533122 -1.126048 3.420142
H -3.953196 -0.391596 3.253430
H -5.735923 1.031400 2.220244
H -4.614345 0.490941 0.972302
H -6.183453 -0.293796 1.144200
H 6.246459 0.115409 -0.145001
H 0.080119 -0.580911 -2.981175
H -1.486262 -2.350862 -3.089224

2b-c26, ΔG = 2.3626 kcal/mol, population = 0.21 %
C 5.076326 0.422292 1.136911
C 3.978790 1.311652 1.713252
C 2.688170 1.167179 0.964109
C 2.589779 0.668096 -0.299160
C 3.799834 0.319491 -1.011193
C 5.146488 0.635278 -0.363984
C 1.255803 0.429377 -0.990853
N 1.562741 1.605998 1.635412
C 0.356428 1.654218 1.012433
C 0.181023 1.166633 -0.242554
C -0.876472 2.220839 1.640944
C -1.925456 2.136499 0.511379
C -1.193280 1.348704 -0.622131
O -1.779041 0.947090 -1.619798
O -3.038894 1.373105 0.957620

C -2.309441 3.499204 -0.000914
C -3.553400 3.941373 -0.126659
C 0.907422 -1.074032 -1.109318
C 0.818475 -1.806232 0.229388
C -0.316632 -2.838084 0.276221
C -1.686287 -2.178542 0.281869
C -2.855119 -3.163302 0.288238
C -4.215791 -2.471747 0.149175
C -4.481775 -1.908595 -1.250154
C -5.835348 -1.203629 -1.392448
C -5.900679 0.152669 -0.690111
O 3.796592 -0.199036 -2.129355
O 6.163619 -0.152050 -0.955423
O 2.065438 -2.442209 0.580748
O -0.184839 -3.629852 1.462430
H 6.036068 0.659717 1.595866
H 4.854460 -0.628259 1.338457
H 4.285380 2.363219 1.672575
H 3.807459 1.078952 2.766317
H 5.340649 1.701677 -0.561033
H 1.322276 0.812868 -2.012734
H 1.658698 1.955418 2.577702
H -1.197726 1.609754 2.486285
H -0.716086 3.237599 2.002524
H -3.516730 1.068055 0.173175
H -1.471948 4.125187 -0.296155
H -3.757967 4.930327 -0.516163
H -4.402917 3.337297 0.165957
H 1.655153 -1.560494 -1.737580
H -0.040037 -1.146409 -1.642502
H 0.644745 -1.102161 1.045348
H -0.228111 -3.491478 -0.605365
H -1.753388 -1.524430 1.157293
H -1.765219 -1.533977 -0.592837
H -2.729694 -3.880568 -0.531072
H -2.830764 -3.742289 1.213749
H -5.009108 -3.187084 0.389045
H -4.286077 -1.675547 0.897212
H -3.689203 -1.209094 -1.534355
H -4.431580 -2.735531 -1.966179
H -6.052288 -1.059510 -2.454752
H -6.624353 -1.856349 -1.003988
H -6.885506 0.611554 -0.797682
H -5.696296 0.067711 0.379210

H -5.174443 0.844838 -1.127789
H 5.799008 -0.441437 -1.809216
H 2.405333 -2.897164 -0.200406
H 0.767293 -3.765920 1.577632

2b-c120, ΔG = 2.3676 kcal/mol, population = 0.21 %
C 4.461564 0.501894 2.449166
C 3.083897 1.116268 2.681202
C 2.154843 0.858526 1.533601
C 2.561549 0.487662 0.288160
C 3.978789 0.423545 -0.003755
C 4.958979 0.893712 1.068891
C 1.566158 0.082530 -0.789466
N 0.809452 1.073245 1.791704
C -0.070292 1.174320 0.764333
C 0.277507 0.792196 -0.495559
C -1.448600 1.744436 0.874859
C -1.860791 1.937377 -0.605983
C -0.767757 1.164059 -1.392566
O -0.874364 0.917139 -2.595520
O -3.122931 1.334645 -0.843963
C -1.811554 3.392924 -0.997676
C -2.798248 4.050348 -1.590761
C 1.472610 -1.465418 -0.810904
C 0.463395 -2.116407 -1.769264
C -0.972767 -2.237627 -1.226548
C -1.081449 -3.174768 -0.032604
C -2.483969 -3.261815 0.574786
C -2.977207 -1.953991 1.197681
C -4.254171 -2.139048 2.018761
C -4.779452 -0.855935 2.670310
C -5.362561 0.151375 1.678581
O 4.427089 0.032518 -1.081375
O 6.247537 0.367228 0.813372
O 0.448713 -1.495741 -3.063980
O -1.815908 -2.748823 -2.269429
H 5.161328 0.845298 3.210870
H 4.403767 -0.587536 2.510219
H 3.170769 2.200423 2.817033
H 2.636537 0.724691 3.597481
H 4.985619 1.992838 1.000440
H 1.947208 0.407597 -1.759179
H 0.533753 1.362812 2.719287
H -2.124574 1.031477 1.349147

H -1.469759 2.669463 1.451043
H -3.202897 1.196684 -1.798749
H -0.874264 3.892422 -0.770144
H -2.693311 5.094979 -1.853787
H -3.741804 3.572035 -1.821400
H 1.274177 -1.811838 0.203890
H 2.468389 -1.825995 -1.071396
H 0.807205 -3.135788 -1.963048
H -1.334438 -1.247190 -0.939662
H -0.752469 -4.168495 -0.351795
H -0.389688 -2.844523 0.745425
H -3.194580 -3.595825 -0.185917
H -2.470867 -4.037189 1.347473
H -2.193732 -1.548769 1.850152
H -3.141472 -1.208600 0.415853
H -5.037702 -2.565064 1.380951
H -4.058500 -2.881782 2.799376
H -5.548811 -1.121150 3.401831
H -3.968565 -0.386542 3.238787
H -4.622176 0.489779 0.952963
H -6.191738 -0.294732 1.121823
H -5.747880 1.033371 2.195814
H 6.243086 0.117648 -0.126180
H 0.086905 -0.589410 -2.980713
H -1.478408 -2.360002 -3.089772

2b-c33, ΔG = 2.5157 kcal/mol, population = 0.16 %
C 4.969320 0.288831 1.066336
C 3.916960 1.206253 1.682250
C 2.604312 1.118590 0.962490
C 2.460445 0.649175 -0.307592
C 3.639252 0.261141 -1.049923
C 5.011679 0.520488 -0.433146
C 1.102605 0.465061 -0.969445
N 1.509232 1.577138 1.670718
C 0.294544 1.689284 1.072769
C 0.077524 1.236795 -0.189034
C -0.903841 2.299693 1.729029
C -1.925823 2.387593 0.574002
C -1.284157 1.518301 -0.551804
O -1.915374 1.158778 -1.537767
O -3.164035 1.814918 0.966964
C -2.061020 3.802350 0.070572
C -3.210256 4.443781 -0.092002

C 0.688681 -1.020393 -1.095847
C 0.723516 -1.803074 0.214185
C -0.360124 -2.889804 0.281523
C -1.742179 -2.298495 0.511762
C -2.869641 -3.324661 0.413014
C -4.249208 -2.750441 0.756425
C -4.661366 -1.519867 -0.058499
C -4.699765 -1.739736 -1.570568
C -5.157380 -0.496267 -2.331124
O 3.588839 -0.253650 -2.168813
O 5.984775 -0.294888 -1.059743
O 2.018788 -2.395384 0.448227
O -0.068427 -3.790009 1.355803
H 5.947341 0.486555 1.505089
H 4.715832 -0.756208 1.257828
H 4.258488 2.247367 1.650190
H 3.763601 0.962491 2.735636
H 5.240149 1.581499 -0.621249
H 1.157690 0.858384 -1.988274
H 1.638686 1.904047 2.616962
H -1.291432 1.641694 2.509005
H -0.678438 3.266139 2.181322
H -3.638923 1.583035 0.155519
H -1.121405 4.285990 -0.181134
H -3.234784 5.458135 -0.469042
H -4.157632 3.983194 0.158048
H 1.339492 -1.513020 -1.820743
H -0.315711 -1.041447 -1.519559
H 0.575396 -1.138964 1.068620
H -0.350423 -3.446485 -0.667847
H -1.748474 -1.826917 1.500328
H -1.910840 -1.502882 -0.214974
H -2.880825 -3.741369 -0.598890
H -2.656989 -4.158774 1.085468
H -4.999886 -3.536413 0.622654
H -4.266744 -2.486870 1.819231
H -5.651576 -1.193910 0.276761
H -3.981914 -0.692308 0.164591
H -3.708042 -2.031677 -1.927544
H -5.365803 -2.579974 -1.795440
H -5.195085 -0.676444 -3.407853
H -6.154599 -0.184280 -2.009217
H -4.471088 0.335806 -2.156523
H 5.584625 -0.566948 -1.903334

H 2.350095 -2.747901 -0.387542
H 0.897859 -3.861567 1.374879

2b-c367, ΔG = 2.6851 kcal/mol, population = 0.12 %
C 4.939753 1.052193 -0.378694
C 4.011280 2.224028 -0.071091
C 2.619439 1.768548 0.247130
C 2.123986 0.544650 -0.094764
C 2.935626 -0.355088 -0.875011
C 4.280289 0.138200 -1.397130
C 0.747720 0.067014 0.334303
N 1.815504 2.686804 0.886553
C 0.481075 2.456404 1.039943
C -0.074905 1.256749 0.733319
C -0.487673 3.483843 1.536068
C -1.858599 2.818724 1.280526
C -1.496148 1.340437 0.938697
O -2.342946 0.458241 0.891088
O -2.649043 2.848831 2.459520
C -2.549957 3.427564 0.087076
C -3.805235 3.854757 0.074517
C 0.874214 -0.984173 1.468003
C -0.258047 -2.011200 1.551757
C -0.466340 -2.767955 0.235652
C -1.612986 -3.770025 0.298450
C -2.123805 -4.210065 -1.082124
C -3.247127 -3.336888 -1.657913
C -2.893451 -1.867525 -1.891820
C -4.016537 -1.071899 -2.556739
C -3.656340 0.400292 -2.752843
O 2.583215 -1.506080 -1.167762
O 5.114546 -0.958917 -1.719309
O 0.018923 -2.931269 2.619461
O 0.734588 -3.475961 -0.119741
H 5.891626 1.415894 -0.765217
H 5.140671 0.481956 0.531406
H 3.959290 2.901800 -0.930927
H 4.396018 2.812709 0.764149
H 4.061948 0.719038 -2.307350
H 0.285775 -0.406736 -0.532328
H 2.202503 3.585809 1.135084
H -0.358776 3.654674 2.606436
H -0.371296 4.439443 1.023587
H -3.315902 2.152455 2.369393

H -1.943551 3.478560 -0.812713
H -4.245301 4.267166 -0.824301
H -4.426766 3.815912 0.960198
H 0.951662 -0.466638 2.426034
H 1.809534 -1.530817 1.333496
H -1.192618 -1.516115 1.812758
H -0.689165 -2.040398 -0.542480
H -1.268806 -4.634430 0.870318
H -2.435474 -3.329662 0.869774
H -1.281659 -4.239526 -1.780910
H -2.497240 -5.235460 -1.014570
H -3.575219 -3.775286 -2.606838
H -4.110939 -3.387901 -0.984486
H -2.648723 -1.382487 -0.943784
H -1.993621 -1.804009 -2.515854
H -4.262393 -1.525224 -3.523100
H -4.920982 -1.148202 -1.943374
H -2.771610 0.504414 -3.387155
H -4.471095 0.954274 -3.224593
H -3.434637 0.877855 -1.795796
H 4.516239 -1.713313 -1.849118
H 0.710822 -3.522939 2.291473
H 1.364047 -2.830964 -0.487886

2b-c24, ΔG = 2.7623 kcal/mol, population = 0.11 %
C 4.234049 0.023328 2.101095
C 2.985129 0.840739 2.419747
C 2.027574 0.873499 1.266638
C 2.372034 0.591156 -0.019595
C 3.755606 0.316365 -0.331633
C 4.800026 0.481435 0.769565
C 1.348428 0.497371 -1.141086
N 0.731550 1.237957 1.581373
C -0.173452 1.483706 0.598356
C 0.100226 1.206975 -0.703055
C -1.526634 2.077781 0.835141
C -2.018941 2.393308 -0.595859
C -1.014635 1.622896 -1.507732
O -1.227791 1.432727 -2.698499
O -3.329522 1.891976 -0.798953
C -1.898128 3.867244 -0.894710
C -2.864914 4.617808 -1.404944
C 1.046362 -0.956157 -1.584691
C 0.577324 -1.894753 -0.480328

C -0.133749 -3.144012 -1.020954
C -1.464471 -2.879931 -1.725107
C -2.445804 -1.916959 -1.048041
C -2.941505 -2.327553 0.338326
C -4.004983 -1.376417 0.883777
C -4.432064 -1.695205 2.315163
C -5.490131 -0.731809 2.850374
O 4.135789 -0.030441 -1.452262
O 5.976416 -0.230105 0.430823
O 1.667824 -2.323612 0.365306
O -0.354818 -4.061855 0.058071
H 4.977872 0.145243 2.888455
H 3.979744 -1.036785 2.033059
H 3.260945 1.871465 2.671033
H 2.473512 0.434964 3.295185
H 5.022323 1.558473 0.829085
H 1.757787 0.999790 -2.021588
H 0.498712 1.439011 2.542856
H -2.192936 1.348896 1.299691
H -1.483844 2.956388 1.479669
H -3.432578 1.749641 -1.751326
H -0.924961 4.297568 -0.675547
H -2.708188 5.669987 -1.605135
H -3.842367 4.208086 -1.626049
H 1.938976 -1.377276 -2.053251
H 0.283011 -0.900280 -2.361820
H -0.098413 -1.378920 0.202705
H 0.543676 -3.617275 -1.747314
H -1.245647 -2.504644 -2.727711
H -1.943791 -3.852905 -1.867190
H -2.003221 -0.919984 -0.986099
H -3.310110 -1.808615 -1.711512
H -3.345402 -3.344697 0.294269
H -2.100668 -2.371372 1.034054
H -3.627835 -0.349906 0.839359
H -4.884097 -1.399030 0.228941
H -4.812796 -2.721228 2.360160
H -3.550777 -1.667835 2.965132
H -6.395094 -0.764113 2.237837
H -5.773172 -0.974849 3.876940
H -5.121539 0.297510 2.839973
H 5.921066 -0.372474 -0.529642
H 2.402480 -2.595462 -0.200213
H 0.434347 -3.998542 0.616227

2b-c124, ΔG = 2.7836 kcal/mol, population = 0.10 %
C 5.024895 0.206033 1.002176
C 4.012523 1.147492 1.647971
C 2.683939 1.106757 0.953539
C 2.500134 0.649458 -0.315769
C 3.651764 0.235544 -1.086245
C 5.043316 0.450940 -0.495665
C 1.123474 0.512019 -0.949970
N 1.619140 1.598852 1.684407
C 0.399028 1.759220 1.108999
C 0.142282 1.320518 -0.150037
C -0.762818 2.411315 1.790293
C -1.806679 2.533350 0.658207
C -1.215407 1.650452 -0.485192
O -1.876029 1.317808 -1.461109
O -3.050651 1.993157 1.078231
C -1.914767 3.953830 0.164616
C -3.048953 4.629141 0.037055
C 0.658124 -0.959081 -1.063527
C 0.653963 -1.726068 0.256958
C -0.490445 -2.746328 0.345586
C -1.839663 -2.074091 0.545667
C -3.021485 -3.039647 0.472579
C -4.374830 -2.365407 0.722467
C -4.701761 -1.202934 -0.223948
C -4.666559 -1.544191 -1.717168
C -5.660717 -2.629331 -2.129393
O 3.563935 -0.263792 -2.209714
O 5.980692 -0.384718 -1.149314
O 1.913587 -2.388963 0.494694
O -0.257103 -3.630710 1.447139
H 6.016938 0.370807 1.422587
H 4.744676 -0.833019 1.189345
H 4.384124 2.178394 1.620070
H 3.872075 0.897337 2.701786
H 5.296505 1.507171 -0.679269
H 1.171818 0.901107 -1.970632
H 1.778491 1.919709 2.628048
H -1.155705 1.769072 2.580855
H -0.493190 3.370090 2.234458
H -3.558501 1.795771 0.277746
H -0.968831 4.411507 -0.110939
H -3.054308 5.645531 -0.335194

H -4.002319 4.195025 0.311076
H 1.298383 -1.481244 -1.777155
H -0.342818 -0.951108 -1.495606
H 0.549092 -1.043552 1.102997
H -0.505448 -3.329761 -0.587708
H -1.827861 -1.569459 1.517734
H -1.960750 -1.295951 -0.208650
H -3.023738 -3.522670 -0.509799
H -2.881969 -3.837529 1.205417
H -5.161316 -3.122508 0.658632
H -4.399441 -1.989753 1.750766
H -5.695608 -0.818366 0.028466
H -4.003447 -0.383934 -0.037042
H -4.874388 -0.631639 -2.283528
H -3.655397 -1.849167 -2.002926
H -5.434986 -3.585767 -1.653430
H -6.680320 -2.352419 -1.847311
H -5.644581 -2.787998 -3.209807
H 5.559172 -0.632521 -1.989894
H 2.208782 -2.788864 -0.333283
H 0.701966 -3.767162 1.468843

2b-c358, ΔG = 2.8269 kcal/mol, population = 0.10 %
C 4.789311 0.524468 2.146335
C 3.469450 1.211724 2.485320
C 2.414389 0.934424 1.457283
C 2.672666 0.485065 0.198227
C 4.047433 0.344800 -0.235879
C 5.153300 0.822728 0.702472
C 1.555088 0.070424 -0.747180
N 1.112181 1.215883 1.841496
C 0.132200 1.301802 0.907182
C 0.330533 0.845899 -0.360299
C -1.203090 1.936506 1.130179
C -1.766191 2.070754 -0.306575
C -0.788710 1.218511 -1.163851
O -1.023399 0.925491 -2.337884
O -3.067003 1.507508 -0.365838
C -1.712313 3.501710 -0.779775
C -2.743979 4.175598 -1.268804
C 1.398209 -1.469698 -0.674856
C 0.304303 -2.120267 -1.534983
C -1.097797 -2.162327 -0.904938
C -1.181363 -3.005295 0.359758

C -2.568372 -2.991496 1.010072
C -2.954792 -1.627947 1.585857
C -4.322550 -1.605379 2.275541
C -5.511009 -1.942105 1.365450
C -5.641844 -1.022007 0.151577
O 4.363186 -0.118073 -1.331936
O 6.385634 0.225759 0.344386
O 0.222214 -1.544148 -2.849324
O -2.010744 -2.716821 -1.864915
H 5.578871 0.874195 2.811297
H 4.695146 -0.556747 2.273768
H 3.612362 2.296730 2.548108
H 3.108051 0.890253 3.464569
H 5.216829 1.914564 0.571177
H 1.845471 0.329159 -1.766698
H 0.944998 1.560236 2.776180
H -1.852884 1.280477 1.711597
H -1.125149 2.890205 1.652505
H -3.270953 1.334454 -1.295858
H -0.733835 3.967620 -0.705808
H -2.635142 5.201058 -1.597580
H -3.728272 3.730861 -1.344480
H 1.251311 -1.752822 0.368080
H 2.358808 -1.887735 -0.977867
H 0.597966 -3.158273 -1.711139
H -1.413430 -1.142637 -0.674885
H -0.896578 -4.030754 0.105168
H -0.450570 -2.639833 1.084766
H -3.308121 -3.314285 0.275085
H -2.586045 -3.734385 1.814091
H -2.189957 -1.323478 2.309326
H -2.942383 -0.871742 0.798935
H -4.312389 -2.304816 3.118583
H -4.480632 -0.609368 2.703455
H -5.435143 -2.979576 1.027182
H -6.427770 -1.886286 1.960220
H -5.695662 0.025899 0.457473
H -4.789144 -1.121311 -0.522119
H -6.543950 -1.253235 -0.419050
H 6.273096 -0.067582 -0.575583
H -0.121500 -0.630580 -2.771503
H -1.718485 -2.381178 -2.725001

2b-c123, ΔG = 2.9480 kcal/mol, population = 0.08 %

C 4.792013 -0.099544 1.591682
C 3.748738 0.901492 2.079703
C 2.570395 0.978389 1.156412
C 2.589633 0.571420 -0.142203
C 3.830351 0.091881 -0.707861
C 5.102250 0.180817 0.132749
C 1.346298 0.556914 -1.019341
N 1.425435 1.526816 1.703717
C 0.350413 1.805504 0.922734
C 0.297800 1.421200 -0.379012
C -0.860173 2.542026 1.403484
C -1.653967 2.792217 0.101643
C -0.942353 1.877365 -0.943504
O -1.441773 1.621188 -2.031948
O -3.002607 2.378559 0.254770
C -1.524581 4.225302 -0.349194
C -2.540498 5.003747 -0.696076
C 0.797199 -0.864633 -1.297249
C 0.466981 -1.683900 -0.056487
C -0.545858 -2.804111 -0.328418
C -1.939823 -2.335698 -0.734950
C -2.600421 -1.344876 0.223337
C -4.027128 -0.961633 -0.183396
C -5.034256 -2.115524 -0.232845
C -5.190828 -2.872053 1.086553
C -6.280384 -3.941175 1.028268
O 3.914507 -0.370694 -1.847634
O 6.078921 -0.710765 -0.373368
O 1.647336 -2.268890 0.536735
O -0.676429 -3.610672 0.850201
H 5.699458 -0.023707 2.190823
H 4.406852 -1.117568 1.684053
H 4.191516 1.901120 2.158062
H 3.401380 0.637385 3.080617
H 5.462903 1.217454 0.039521
H 1.611182 0.972452 -1.995615
H 1.431425 1.809679 2.672708
H -1.451034 1.916938 2.075495
H -0.599464 3.458110 1.934946
H -3.332006 2.147053 -0.625977
H -0.504918 4.597544 -0.394069
H -2.377406 6.022979 -1.022104
H -3.563176 4.650951 -0.653154
H 1.517280 -1.413110 -1.908050

H -0.100374 -0.741801 -1.903442
H 0.068443 -1.045323 0.732183
H -0.140061 -3.425706 -1.140267
H -1.892496 -1.901678 -1.737055
H -2.554922 -3.234075 -0.825808
H -2.595694 -1.759713 1.233131
H -2.010933 -0.427380 0.269383
H -4.391336 -0.203467 0.516376
H -3.995041 -0.478595 -1.166031
H -6.009027 -1.710791 -0.525400
H -4.757769 -2.823924 -1.020354
H -4.240984 -3.341055 1.358191
H -5.418618 -2.157237 1.885032
H -7.253216 -3.498586 0.798221
H -6.061652 -4.679946 0.252487
H -6.371337 -4.472889 1.977987
H 5.798755 -0.905664 -1.284017
H 2.160454 -2.692420 -0.163795
H 0.216762 -3.681675 1.217425

2b-c570, ΔG = 2.9807 kcal/mol, population = 0.07 %
C 4.792627 0.922422 -0.508063
C 3.922223 2.129829 -0.167973
C 2.525346 1.729539 0.198400
C 1.971004 0.525911 -0.124007
C 2.717821 -0.404171 -0.931773
C 4.061429 0.035596 -1.501478
C 0.591863 0.104959 0.350850
N 1.777296 2.679041 0.859739
C 0.437470 2.507008 1.038153
C -0.174857 1.331304 0.746419
C -0.477946 3.577663 1.542210
C -1.880653 2.974210 1.304806
C -1.589699 1.481131 0.954949
O -2.475592 0.638477 0.900095
O -2.649453 3.037862 2.496969
C -2.560429 3.619651 0.124294
C -3.787631 4.121889 0.137015
C 0.710358 -0.927528 1.502170
C -0.459746 -1.906285 1.646655
C -0.756186 -2.655882 0.343819
C -1.913085 -3.639591 0.461312
C -2.421111 -4.157815 -0.892818
C -3.538469 -3.317857 -1.530685

C -3.212212 -1.851807 -1.837457
C -2.096024 -1.643969 -2.862817
C -1.785749 -0.166416 -3.099996
O 2.308492 -1.539418 -1.211604
O 4.839872 -1.092863 -1.853012
O -0.171567 -2.827165 2.709908
O 0.407200 -3.383096 -0.083959
H 5.742846 1.249440 -0.929703
H 5.005042 0.344098 0.394247
H 3.866841 2.809286 -1.026277
H 4.359884 2.702373 0.652577
H 3.833105 0.625933 -2.403052
H 0.092201 -0.368249 -0.494360
H 2.203794 3.564579 1.090908
H -0.328840 3.745244 2.610502
H -0.324296 4.526107 1.026133
H -3.360217 2.385981 2.411269
H -1.969301 3.634513 -0.787007
H -4.220219 4.561530 -0.752492
H -4.391568 4.120839 1.035696
H 0.844087 -0.395256 2.445788
H 1.616432 -1.516625 1.346419
H -1.361722 -1.372994 1.945562
H -1.012406 -1.914943 -0.410266
H -1.579720 -4.473128 1.083320
H -2.731129 -3.156187 1.003750
H -1.574417 -4.250623 -1.577473
H -2.809902 -5.171095 -0.760743
H -3.851478 -3.809099 -2.458790
H -4.408045 -3.344356 -0.864999
H -4.120774 -1.370297 -2.215360
H -2.963400 -1.319760 -0.914531
H -1.185117 -2.154589 -2.538483
H -2.383585 -2.116933 -3.807826
H -2.664986 0.364847 -3.473354
H -1.479037 0.323412 -2.172287
H -0.981371 -0.036857 -3.827133
H 4.208591 -1.822827 -1.964435
H 0.497393 -3.436166 2.366936
H 1.036409 -2.752801 -0.478195

2b-c84, ΔG = 2.9907 kcal/mol, population = 0.07 %
C 4.807248 0.213163 1.374612
C 3.722362 1.153312 1.890293

C 2.485664 1.099296 1.044946
C 2.449021 0.611641 -0.226565
C 3.686433 0.222481 -0.861334
C 4.998540 0.450662 -0.111470
C 1.154780 0.454704 -1.013376
N 1.348048 1.612630 1.630164
C 0.203500 1.755023 0.914280
C 0.088390 1.280269 -0.353606
C -1.024096 2.433434 1.436855
C -1.912531 2.553477 0.178965
C -1.221896 1.600955 -0.845320
O -1.788466 1.218011 -1.862182
O -3.228161 2.097183 0.447233
C -1.879682 3.955464 -0.376998
C -2.951645 4.662823 -0.707255
C 0.729028 -1.026205 -1.195171
C 0.036151 -1.666740 0.004017
C -0.322713 -3.135337 -0.272878
C -1.247786 -3.745750 0.773006
C -2.610143 -3.058818 0.908162
C -3.454301 -3.084632 -0.368428
C -4.825969 -2.420542 -0.218692
C -4.756991 -0.903470 -0.039113
C -6.134035 -0.247628 0.039887
O 3.741407 -0.264446 -1.994134
O 6.010721 -0.382665 -0.647385
O 0.807259 -1.575363 1.205788
O 0.884728 -3.928671 -0.262286
H 5.742615 0.384034 1.907746
H 4.511833 -0.826730 1.534723
H 4.090976 2.185483 1.900613
H 3.457395 0.908364 2.921224
H 5.266800 1.507184 -0.269724
H 1.333816 0.836304 -2.022462
H 1.394164 1.949043 2.580799
H -1.525463 1.805144 2.175536
H -0.799975 3.393914 1.902530
H -3.588583 1.767679 -0.389080
H -0.883826 4.367230 -0.514389
H -2.858646 5.662758 -1.111261
H -3.950947 4.268894 -0.572115
H 1.617594 -1.594324 -1.476216
H 0.035029 -1.075734 -2.037865
H -0.881594 -1.115558 0.207923

H -0.784773 -3.200622 -1.261098
H -1.393613 -4.796768 0.508542
H -0.731299 -3.727578 1.735741
H -3.167171 -3.559993 1.705731
H -2.470887 -2.029325 1.245575
H -2.919092 -2.587465 -1.183508
H -3.584798 -4.126482 -0.678957
H -5.427751 -2.641187 -1.106465
H -5.357795 -2.864538 0.630988
H -4.189655 -0.659072 0.861758
H -4.192670 -0.479185 -0.877137
H -6.708744 -0.645344 0.880527
H -6.056907 0.833670 0.174219
H -6.709623 -0.431062 -0.871170
H 5.694393 -0.627701 -1.533856
H 1.504376 -2.242269 1.128833
H 1.350738 -3.794661 -1.095461

2b-c14, ΔG = 3.0215 kcal/mol, population = 0.07 %
C 4.993946 0.495113 1.126748
C 3.837824 1.278034 1.741933
C 2.564645 1.102459 0.969579
C 2.508056 0.671837 -0.321706
C 3.742751 0.434143 -1.037162
C 5.061714 0.797101 -0.359170
C 1.195850 0.393483 -1.039809
N 1.409446 1.443439 1.647597
C 0.214597 1.483886 1.001436
C 0.082648 1.057154 -0.280447
C -1.055356 1.975952 1.624206
C -2.027216 2.027702 0.436280
C -1.282871 1.246752 -0.687463
O -1.850447 0.871655 -1.706750
O -3.238718 1.345127 0.771803
C -2.338029 3.407205 -0.092020
C -1.697786 4.536928 0.179477
C 0.926575 -1.120632 -1.211666
C 0.933451 -1.912530 0.098388
C -0.210849 -2.931119 0.194540
C -1.560080 -2.250042 0.355548
C -2.747803 -3.210451 0.406303
C -4.092026 -2.484707 0.526364
C -4.499274 -1.709761 -0.727434
C -5.801548 -0.927368 -0.557870

C -6.195206 -0.143242 -1.808786
O 3.779397 -0.024157 -2.180472
O 6.131371 0.110588 -0.982544
O 2.194362 -2.578458 0.320326
O 0.009005 -3.784084 1.323380
H 5.933127 0.766373 1.608863
H 4.839292 -0.577401 1.265896
H 4.078202 2.347109 1.769547
H 3.672571 0.970985 2.776793
H 5.188745 1.882937 -0.494159
H 1.254975 0.815325 -2.046698
H 1.475744 1.743998 2.609137
H -1.422589 1.253159 2.354922
H -0.928500 2.928496 2.136589
H -3.654856 1.079243 -0.060857
H -3.160611 3.412954 -0.801732
H -1.995903 5.467224 -0.287060
H -0.855958 4.587142 0.858883
H 1.679607 -1.534358 -1.884032
H -0.033475 -1.229226 -1.716700
H 0.839421 -1.243979 0.955885
H -0.212794 -3.539261 -0.723021
H -1.535242 -1.651906 1.272640
H -1.695479 -1.552368 -0.470265
H -2.750070 -3.833387 -0.495508
H -2.623217 -3.888558 1.253161
H -4.875368 -3.213013 0.759206
H -4.050949 -1.795488 1.377571
H -3.702988 -1.017433 -1.016304
H -4.605049 -2.410107 -1.563897
H -6.606727 -1.619284 -0.290060
H -5.701182 -0.240827 0.290200
H -6.332952 -0.812564 -2.661892
H -7.127046 0.406438 -1.660671
H -5.421552 0.579893 -2.081506
H 5.790598 -0.157052 -1.853114
H 2.461150 -3.008406 -0.502354
H 0.966133 -3.930621 1.353641

2b-c305, ΔG = 3.0334 kcal/mol, population = 0.07 %
C 3.981324 0.030322 2.480954
C 2.677365 0.795857 2.675137
C 1.869212 0.861708 1.413588
C 2.368895 0.628697 0.167635

C 3.801022 0.454221 0.019763
C 4.688328 0.579143 1.257786
C 1.504593 0.616316 -1.091577
N 0.545466 1.217149 1.587210
C -0.273097 1.401382 0.522229
C 0.131983 1.132868 -0.748146
C -1.695863 1.847046 0.646421
C -2.149141 1.979961 -0.823912
C -1.001700 1.286995 -1.613632
O -1.161542 0.883569 -2.764477
O -3.359088 1.272197 -1.036101
C -2.224976 3.424849 -1.250427
C -3.260762 3.975524 -1.868532
C 1.518427 -0.751765 -1.821544
C 1.007132 -1.974102 -1.057504
C -0.516458 -2.013378 -0.886286
C -0.990827 -3.188145 -0.043575
C -2.491686 -3.168442 0.259465
C -2.963126 -1.898028 0.967779
C -4.415452 -1.970470 1.438344
C -4.943195 -0.657209 2.026027
C -4.230817 -0.216805 3.305512
O 4.349017 0.256285 -1.065532
O 5.924648 -0.073240 1.033757
O 1.448185 -3.166582 -1.726904
O -1.150192 -2.096176 -2.176875
H 4.615356 0.132535 3.361577
H 3.777700 -1.032711 2.331697
H 2.885789 1.820568 3.003655
H 2.072049 0.336584 3.459533
H 4.858904 1.658002 1.402943
H 1.959387 1.310861 -1.806057
H 0.197067 1.371223 2.522294
H -2.299367 1.088102 1.145159
H -1.782080 2.774000 1.214737
H -3.377252 1.020546 -1.971639
H -1.338810 4.011862 -1.026664
H -3.249127 5.018876 -2.156401
H -4.153319 3.405408 -2.093061
H 2.551627 -0.955300 -2.092948
H 0.962873 -0.644772 -2.754929
H 1.467306 -2.004313 -0.067017
H -0.822085 -1.092640 -0.394063
H -0.722457 -4.118974 -0.547993

H -0.431688 -3.166275 0.897474
H -3.052597 -3.291883 -0.670803
H -2.728864 -4.036908 0.882486
H -2.303548 -1.699452 1.819212
H -2.867630 -1.043834 0.292281
H -5.045407 -2.255952 0.589163
H -4.519474 -2.770062 2.181142
H -4.855799 0.129554 1.269668
H -6.011697 -0.766577 2.234534
H -4.296600 -0.990620 4.075405
H -3.171462 -0.016119 3.133282
H -4.675332 0.695275 3.709489
H 6.022018 -0.114406 0.067425
H 0.870188 -3.268829 -2.496377
H -1.159325 -1.210998 -2.570557

2b-c103, ΔG = 3.0491 kcal/mol, population = 0.07 %
C 4.650001 0.049804 1.588156
C 3.546723 1.000463 2.042970
C 2.397163 1.025989 1.080746
C 2.476475 0.615371 -0.214719
C 3.756166 0.196589 -0.740093
C 4.994679 0.348513 0.140507
C 1.260855 0.528751 -1.125837
N 1.210980 1.522331 1.587779
C 0.148338 1.745779 0.771003
C 0.155557 1.353603 -0.529418
C -1.113838 2.417206 1.212904
C -1.884835 2.612977 -0.111278
C -1.092373 1.736331 -1.131138
O -1.545468 1.448214 -2.231699
O -3.210101 2.120291 0.008664
C -1.828360 4.049237 -0.567862
C -2.879626 4.769553 -0.934555
C 0.787251 -0.925183 -1.375626
C 0.380225 -1.681801 -0.113803
C -0.647273 -2.783884 -0.397292
C -1.249845 -3.389789 0.867884
C -1.941926 -2.384346 1.799911
C -2.970155 -1.468360 1.126368
C -4.118601 -2.204704 0.438766
C -5.122284 -1.261207 -0.223669
C -6.280806 -1.996844 -0.893942
O 3.899430 -0.263323 -1.875168

O 6.029913 -0.493267 -0.333612
O 1.511284 -2.217079 0.595105
O -0.079202 -3.807058 -1.231228
H 5.532825 0.164746 2.217097
H 4.308948 -0.985023 1.666607
H 3.940927 2.018744 2.139006
H 3.177342 0.716433 3.030787
H 5.307315 1.401527 0.060091
H 1.534049 0.935409 -2.103264
H 1.173355 1.810795 2.554577
H -1.685800 1.764071 1.874765
H -0.919607 3.350933 1.741895
H -3.512779 1.899591 -0.884297
H -0.830468 4.477553 -0.598756
H -2.767088 5.794613 -1.263641
H -3.882314 4.362255 -0.905933
H 1.568307 -1.474887 -1.903190
H -0.070025 -0.873064 -2.048976
H -0.079862 -0.982526 0.584136
H -1.441097 -2.343584 -1.002635
H -1.957988 -4.160868 0.556111
H -0.454529 -3.897146 1.422370
H -2.434606 -2.946476 2.598944
H -1.186320 -1.765272 2.291388
H -3.383727 -0.795128 1.883514
H -2.476098 -0.818788 0.398092
H -3.725512 -2.887863 -0.320803
H -4.637360 -2.831937 1.173042
H -5.512013 -0.564592 0.525833
H -4.598337 -0.649624 -0.965658
H -6.978084 -1.301108 -1.365745
H -5.915996 -2.679293 -1.666212
H -6.841414 -2.589978 -0.166601
H 5.790353 -0.697741 -1.253778
H 2.122027 -2.588565 -0.052301
H 0.513925 -4.333942 -0.680864

2b-c67, ΔG = 3.2003 kcal/mol, population = 0.05 %
C 5.076011 0.433376 1.004306
C 3.997633 1.370591 1.539288
C 2.686482 1.181876 0.837984
C 2.545958 0.564188 -0.368490
C 3.729748 0.149394 -1.085505
C 5.097902 0.512893 -0.510977

C 1.188053 0.300101 -1.004296
N 1.588472 1.709406 1.485283
C 0.378533 1.761791 0.868587
C 0.166474 1.169741 -0.334311
C -0.816718 2.460076 1.437033
C -1.847342 2.385789 0.288447
C -1.189164 1.412721 -0.739608
O -1.807486 0.949164 -1.689731
O -3.059450 1.813071 0.759411
C -2.042798 3.728418 -0.365985
C -3.218567 4.300968 -0.585860
C 0.760961 -1.190713 -0.989832
C 0.356376 -1.757125 0.362345
C -0.172008 -3.195756 0.262009
C -1.477123 -3.359877 -0.509338
C -2.671866 -2.578158 0.039020
C -3.961405 -2.900122 -0.719101
C -5.236236 -2.397436 -0.034055
C -5.318019 -0.883267 0.184009
C -5.282895 -0.070977 -1.111111
O 3.689813 -0.462627 -2.155823
O 6.092583 -0.331424 -1.061359
O 1.437020 -1.735248 1.319766
O -0.367097 -3.715774 1.583581
H 6.050140 0.707792 1.409231
H 4.865373 -0.595754 1.304698
H 4.308350 2.414152 1.413047
H 3.851999 1.218188 2.611084
H 5.290641 1.555838 -0.808496
H 1.259348 0.571325 -2.061214
H 1.715009 2.150920 2.384417
H -1.200276 1.925453 2.308081
H -0.586218 3.481447 1.742614
H -3.529687 1.466638 -0.012737
H -1.125135 4.217915 -0.680021
H -3.288302 5.264773 -1.073565
H -4.143635 3.832051 -0.275203
H 1.568929 -1.790816 -1.414794
H -0.090289 -1.282500 -1.665343
H -0.410847 -1.133338 0.821333
H 0.601936 -3.793306 -0.241945
H -1.305361 -3.096804 -1.556034
H -1.719019 -4.426983 -0.503358
H -2.801282 -2.820279 1.097256

H -2.475396 -1.504823 -0.014022
H -3.895080 -2.497147 -1.734775
H -4.041661 -3.986409 -0.831499
H -6.101907 -2.713582 -0.626152
H -5.329042 -2.896433 0.936501
H -6.245530 -0.662162 0.720216
H -4.506258 -0.562127 0.842759
H -6.051725 -0.414620 -1.808275
H -5.475396 0.988741 -0.920689
H -4.318324 -0.149640 -1.616375
H 5.701268 -0.688370 -1.876932
H 2.231211 -2.081742 0.892870
H 0.373974 -3.377036 2.106930

2b-c30, ΔG = 3.3578 kcal/mol, population = 0.04 %
C 4.878241 0.249542 1.131672
C 3.807385 1.142285 1.752281
C 2.513926 1.075755 0.996560
C 2.406301 0.651820 -0.293828
C 3.604849 0.292063 -1.019244
C 4.959136 0.533455 -0.357415
C 1.065923 0.478081 -0.990095
N 1.398105 1.504276 1.692102
C 0.199927 1.637946 1.064148
C 0.024596 1.234514 -0.219759
C -1.022953 2.217646 1.703561
C -2.014681 2.332813 0.523921
C -1.320947 1.533002 -0.621738
O -1.906839 1.223301 -1.651570
O -3.244537 1.702047 0.853705
C -2.183171 3.764424 0.084689
C -3.348897 4.372133 -0.089461
C 0.655856 -1.008678 -1.131967
C 0.788355 -1.825309 0.154673
C -0.395071 -2.779813 0.370701
C -1.629391 -2.030080 0.854085
C -2.898756 -2.883663 0.918706
C -3.478460 -3.273738 -0.447089
C -3.868547 -2.096228 -1.347748
C -4.906495 -1.154908 -0.736588
C -5.310403 -0.025646 -1.683877
O 3.583900 -0.181954 -2.156322
O 5.952220 -0.256042 -0.985454
O 2.028431 -2.561942 0.193891

O -0.053418 -3.757732 1.359098
H 5.843846 0.434338 1.602361
H 4.623977 -0.802457 1.279894
H 4.145917 2.184693 1.765022
H 3.628443 0.861753 2.792323
H 5.187422 1.601354 -0.502146
H 1.139905 0.883120 -2.002503
H 1.500534 1.791950 2.654378
H -1.425496 1.531919 2.451416
H -0.820828 3.171732 2.191684
H -3.693319 1.502570 0.019292
H -1.253417 4.291351 -0.110736
H -3.398016 5.401584 -0.420231
H -4.286503 3.866556 0.104521
H 1.264994 -1.473603 -1.909106
H -0.373622 -1.033376 -1.491894
H 0.845617 -1.172828 1.027779
H -0.604018 -3.289534 -0.580218
H -1.399036 -1.636866 1.848446
H -1.799839 -1.163202 0.214533
H -2.687866 -3.791629 1.488908
H -3.653926 -2.337572 1.488852
H -2.762986 -3.905320 -0.982150
H -4.362555 -3.897695 -0.279061
H -2.981156 -1.515880 -1.618234
H -4.262613 -2.493999 -2.288933
H -5.792513 -1.731882 -0.450235
H -4.513288 -0.726032 0.189142
H -6.040924 0.643017 -1.222168
H -4.439505 0.568032 -1.974242
H -5.756488 -0.421756 -2.599853
H 5.576319 -0.501196 -1.848152
H 2.201910 -2.918226 -0.686617
H 0.888700 -3.940316 1.223902

2b-c25, ΔG = 3.3886 kcal/mol, population = 0.04 %
C 4.998466 0.372216 0.977776
C 3.921117 1.244031 1.615800
C 2.599691 1.100306 0.923891
C 2.445518 0.600377 -0.333472
C 3.619325 0.262329 -1.103884
C 4.993486 0.593421 -0.523982
C 1.077072 0.348788 -0.948416
N 1.503326 1.537711 1.640939

C 0.287890 1.651487 1.043251
C 0.066650 1.173041 -0.207864
C -0.899119 2.304890 1.677603
C -1.928256 2.357998 0.526137
C -1.278592 1.488170 -0.596263
O -1.892440 1.150485 -1.600425
O -3.145356 1.750357 0.937139
C -2.109972 3.762397 0.012734
C -3.279349 4.366724 -0.148630
C 0.667293 -1.147123 -0.975035
C 0.472595 -1.805283 0.383668
C -0.212982 -3.176373 0.287825
C -1.662827 -3.145202 -0.184637
C -2.588434 -2.230013 0.623017
C -4.071375 -2.582854 0.459973
C -4.603209 -2.516197 -0.977537
C -4.530333 -1.132791 -1.631506
C -5.422698 -0.088894 -0.960368
O 3.565739 -0.266628 -2.216956
O 5.988305 -0.181779 -1.167755
O 1.715449 -1.967989 1.101943
O -0.184426 -3.800354 1.578503
H 5.977203 0.617011 1.390487
H 4.799609 -0.682235 1.183302
H 4.218193 2.298477 1.578946
H 3.798615 0.993139 2.671723
H 5.167687 1.662073 -0.726077
H 1.111664 0.665377 -1.993915
H 1.639888 1.892365 2.576319
H -1.291094 1.689917 2.489818
H -0.657686 3.287632 2.084440
H -3.632429 1.511169 0.135410
H -1.187014 4.271825 -0.250044
H -3.337892 5.375896 -0.535697
H -4.210445 3.879618 0.112394
H 1.409976 -1.707687 -1.548475
H -0.270401 -1.210429 -1.529236
H -0.126169 -1.166419 1.033587
H 0.368457 -3.791176 -0.414740
H -1.685802 -2.877534 -1.242748
H -2.031746 -4.173961 -0.126390
H -2.329011 -2.309254 1.681147
H -2.421982 -1.186824 0.342941
H -4.228344 -3.597978 0.839654

H -4.664459 -1.926064 1.102557
H -4.053238 -3.230425 -1.597131
H -5.644898 -2.855117 -0.979461
H -3.498605 -0.774222 -1.639854
H -4.825193 -1.226619 -2.681051
H -6.474702 -0.383271 -1.003470
H -5.171703 0.038740 0.095193
H -5.332619 0.882763 -1.452401
H 5.579877 -0.481280 -1.998063
H 2.369124 -2.345102 0.498806
H 0.671482 -3.559744 1.962851

2b-c270, ΔG = 3.5605 kcal/mol, population = 0.03 %
C 5.039753 0.379693 1.326991
C 3.925981 1.291113 1.833405
C 2.669240 1.147109 1.028674
C 2.618138 0.595718 -0.216716
C 3.853918 0.228004 -0.870645
C 5.175040 0.550518 -0.174542
C 1.313752 0.366440 -0.968036
N 1.527957 1.641728 1.620699
C 0.356921 1.695925 0.935675
C 0.230610 1.172404 -0.310899
C -0.889154 2.318329 1.480206
C -1.882513 2.221956 0.302380
C -1.111873 1.386877 -0.770451
O -1.654780 0.972583 -1.787556
O -3.030091 1.485744 0.712055
C -2.219329 3.575858 -0.261460
C -3.448572 4.030331 -0.464994
C 0.932221 -1.137810 -1.079784
C 0.229685 -1.715532 0.137294
C -0.153510 -3.190093 -0.025413
C -1.119445 -3.491028 -1.171404
C -2.308109 -2.543792 -1.375235
C -3.283055 -2.413284 -0.205634
C -4.490270 -1.545388 -0.560211
C -5.392605 -1.234466 0.632237
C -6.587636 -0.355145 0.267764
O 3.894955 -0.308934 -1.980237
O 6.208757 -0.262342 -0.699828
O 1.007895 -1.571993 1.344680
O -0.730910 -3.656547 1.202779
H 5.980989 0.622437 1.820208

H 4.803015 -0.664155 1.547241
H 4.246103 2.338379 1.789460
H 3.702135 1.079588 2.881253
H 5.388484 1.609019 -0.392998
H 1.457796 0.712973 -1.994607
H 1.582991 2.018549 2.555685
H -1.269568 1.746520 2.328766
H -0.719580 3.342851 1.814341
H -3.456687 1.148926 -0.088896
H -1.358086 4.183784 -0.524205
H -3.618216 5.012046 -0.888189
H -4.320506 3.442919 -0.205830
H 1.835327 -1.711567 -1.298131
H 0.271704 -1.253116 -1.938901
H -0.677875 -1.150539 0.345797
H 0.773248 -3.753455 -0.209092
H -0.538185 -3.516570 -2.096727
H -1.482814 -4.510964 -1.015366
H -1.950713 -1.545700 -1.641461
H -2.860910 -2.901122 -2.250759
H -3.624293 -3.405590 0.107346
H -2.771135 -1.985600 0.659262
H -4.136817 -0.607887 -1.007206
H -5.077396 -2.038571 -1.343259
H -5.747482 -2.174645 1.067099
H -4.798986 -0.742127 1.409202
H -7.210449 -0.834732 -0.491804
H -7.216685 -0.153552 1.137456
H -6.259495 0.607272 -0.134518
H 5.884276 -0.558991 -1.567157
H 1.912010 -1.859799 1.162869
H -0.195289 -3.263939 1.907303

2b-c40, ΔG = 3.7625 kcal/mol, population = 0.02 %
C 4.957685 0.273180 1.296305
C 3.870681 1.203493 1.826013
C 2.617603 1.130698 1.006263
C 2.559365 0.626903 -0.258881
C 3.787632 0.238388 -0.914439
C 5.112157 0.493378 -0.197135
C 1.253418 0.468575 -1.025324
N 1.487451 1.642230 1.607824
C 0.325754 1.765905 0.914467
C 0.195933 1.292537 -0.351120

C -0.907457 2.403962 1.471316
C -1.899224 2.360567 0.288441
C -1.139446 1.550003 -0.809474
O -1.686285 1.177669 -1.840521
O -3.054903 1.620256 0.667439
C -2.220643 3.736085 -0.229129
C -3.444123 4.210311 -0.421328
C 0.800213 -1.007972 -1.185949
C 0.135640 -1.623502 0.037194
C -0.453560 -3.007338 -0.262062
C -1.346245 -3.538048 0.858665
C -2.553389 -2.661427 1.213665
C -3.465014 -2.330673 0.030318
C -4.790849 -1.699751 0.459003
C -5.714866 -1.339526 -0.710278
C -5.270525 -0.107008 -1.498789
O 3.821391 -0.264667 -2.040045
O 6.124109 -0.332476 -0.743981
O 1.012428 -1.681797 1.174640
O 0.589005 -3.950461 -0.561826
H 5.902116 0.466323 1.805216
H 4.685067 -0.769562 1.477385
H 4.226443 2.240195 1.820697
H 3.629781 0.963989 2.864100
H 5.361047 1.551591 -0.374731
H 1.418298 0.840539 -2.040176
H 1.548710 1.986629 2.554779
H -1.304011 1.820974 2.304536
H -0.713868 3.415360 1.831773
H -3.473254 1.304146 -0.146408
H -1.352200 4.343929 -0.467521
H -3.601933 5.208031 -0.810302
H -4.322774 3.623483 -0.185171
H 1.655666 -1.612186 -1.491393
H 0.078095 -1.044161 -2.004106
H -0.684901 -0.980190 0.353887
H -1.031499 -2.930142 -1.184103
H -1.697359 -4.527815 0.553116
H -0.729578 -3.679604 1.751908
H -3.137551 -3.188441 1.974248
H -2.220643 -1.731750 1.684911
H -2.944892 -1.662461 -0.661182
H -3.675515 -3.248367 -0.530907
H -5.308357 -2.401092 1.121309

H -4.592990 -0.803282 1.057786
H -5.785188 -2.199857 -1.384014
H -6.725339 -1.166926 -0.329089
H -4.243266 -0.196523 -1.858461
H -5.907873 0.059090 -2.369646
H -5.341637 0.791198 -0.877649
H 5.792570 -0.595595 -1.619562
H 1.881737 -1.967486 0.869779
H 1.015724 -4.185487 0.271916

Table S6. Geometry data of conformers of structure **2c**.

2c-c63, ΔG = 0.0000 kcal/mol, population = 11.25 %
C -0.434610 5.423813 -0.572805
C 0.750804 4.851164 0.199713
C 0.827472 3.357839 0.089837
C 0.220039 2.636802 -0.897616
C -0.496069 3.331168 -1.936203
C -0.442415 4.853256 -1.979906
C 0.236810 1.118393 -0.944895
N 1.594263 2.727399 1.042932
C 1.849198 1.390915 0.962623
C 1.265774 0.610785 0.020022
C 2.808569 0.671820 1.859785
C 2.594482 -0.809530 1.476260
C 1.770682 -0.729972 0.155631
O 1.632228 -1.691317 -0.588242
O 3.836794 -1.447342 1.231614
C 1.750546 -1.524128 2.503329
C 2.058478 -2.686571 3.061234
C -1.164540 0.518040 -0.624309
C -1.594286 -0.552349 -1.613845
C -2.920305 -1.212146 -1.228957
C -2.887077 -1.991883 0.081653
C -1.776562 -3.036136 0.203363
C -1.801394 -3.767910 1.543900
C -0.701227 -4.819470 1.670293
C -0.661993 -5.501791 3.036404
C 0.470734 -6.518693 3.162511
O -1.138425 2.746053 -2.820162
O -1.526142 5.362730 -2.734415
O -1.744512 -0.017076 -2.943030
O -3.318648 -2.105210 -2.279994
H -0.370621 6.511108 -0.611099

H -1.371215 5.157766 -0.077194
H 1.688861 5.268407 -0.184169
H 0.695812 5.130318 1.253845
H 0.508067 5.111033 -2.473575
H 0.506649 0.812295 -1.959119
H 2.043912 3.283136 1.756068
H 3.837924 0.962579 1.642449
H 2.614381 0.876145 2.913502
H 3.653525 -2.211626 0.665548
H 0.819016 -1.024484 2.754166
H 1.397386 -3.153182 3.779441
H 2.980133 -3.203130 2.825072
H -1.140419 0.099123 0.381743
H -1.915201 1.312299 -0.610020
H -0.821974 -1.322721 -1.671678
H -3.671869 -0.414929 -1.141687
H -2.809022 -1.276651 0.905130
H -3.860437 -2.478194 0.197125
H -1.866976 -3.759319 -0.612131
H -0.798447 -2.562940 0.087824
H -1.692151 -3.035592 2.352640
H -2.779434 -4.240998 1.688789
H -0.832548 -5.578471 0.890492
H 0.267675 -4.346366 1.479678
H -0.555867 -4.738199 3.814417
H -1.622076 -5.994628 3.223343
H 0.372994 -7.308653 2.413129
H 1.442323 -6.040333 3.013597
H 0.478521 -6.991593 4.147005
H -1.819115 4.630359 -3.301483
H -1.604207 0.947889 -2.920513
H -3.165442 -1.618783 -3.102536

2c-c1, ΔG = 0.1035 kcal/mol, population = 9.44 %
C -2.327378 2.327764 1.092096
C -0.910365 2.591595 1.590321
C 0.118665 1.830084 0.808223
C -0.100120 1.292606 -0.427039
C -1.366664 1.540587 -1.080897
C -2.357517 2.494304 -0.416320
C 0.942381 0.454874 -1.159626
N 1.356122 1.733850 1.406223
C 2.418363 1.220463 0.725738
C 2.271374 0.639147 -0.488462

C 3.826760 1.277325 1.226788
C 4.599126 0.434844 0.188162
C 3.563865 0.227633 -0.961128
O 3.884740 -0.208870 -2.059414
O 5.716282 1.159281 -0.305694
C 4.966037 -0.914674 0.750669
C 6.179788 -1.447899 0.713775
C 0.526401 -1.033203 -1.267595
C 0.345773 -1.758662 0.059117
C -0.479393 -3.046022 -0.063084
C -1.914666 -2.854113 -0.541050
C -2.738362 -1.830552 0.238896
C -4.186352 -1.758085 -0.241695
C -5.028455 -0.747104 0.533298
C -6.474999 -0.659183 0.049598
C -7.298548 0.368591 0.823844
O -1.673619 1.046390 -2.168116
O -3.655511 2.290036 -0.941752
O 1.609418 -2.086728 0.679592
O -0.518206 -3.695577 1.215162
H -3.028380 3.018798 1.560059
H -2.635561 1.312843 1.350344
H -0.675751 3.659403 1.510536
H -0.818291 2.332485 2.647247
H -2.014625 3.511127 -0.664304
H 1.006759 0.818129 -2.189727
H 1.498429 2.167398 2.306687
H 4.202774 2.302043 1.224901
H 3.911797 0.888369 2.242603
H 5.947683 0.770228 -1.161710
H 4.136078 -1.454787 1.196388
H 6.371784 -2.428489 1.130256
H 7.018851 -0.918992 0.279342
H -0.405139 -1.062234 -1.828907
H 1.271339 -1.564405 -1.867449
H -0.141956 -1.106749 0.784708
H 0.035918 -3.703506 -0.778923
H -1.899714 -2.584275 -1.599625
H -2.403452 -3.831310 -0.486565
H -2.715778 -2.077606 1.303694
H -2.287137 -0.839940 0.140976
H -4.198682 -1.496093 -1.305973
H -4.643844 -2.751168 -0.164882
H -5.018211 -1.008270 1.598039

H -4.567849 0.241154 0.447982
H -6.480530 -0.403780 -1.015532
H -6.946422 -1.644566 0.130837
H -7.331221 0.122238 1.888563
H -6.864515 1.367384 0.728804
H -8.327599 0.415435 0.460706
H -3.522133 1.809839 -1.776874
H 2.148573 -2.556371 0.029335
H 0.369155 -3.595243 1.588769

2c-c85, ΔG = 0.1092 kcal/mol, population = 9.35 %
C 0.486761 5.452361 0.668282
C 1.883763 4.846456 0.768628
C 1.887247 3.382404 0.445312
C 0.896337 2.756661 -0.255771
C -0.183648 3.540418 -0.794301
C -0.137955 5.057359 -0.657746
C 0.868922 1.255678 -0.491068
N 2.991605 2.679541 0.868489
C 3.174441 1.379856 0.500570
C 2.219488 0.689936 -0.169620
C 4.438969 0.617260 0.746689
C 4.088760 -0.807116 0.261155
C 2.724671 -0.622347 -0.474017
O 2.242973 -1.490299 -1.189090
O 5.065569 -1.266159 -0.660852
C 3.880833 -1.745589 1.422806
C 4.472013 -2.924445 1.561462
C -0.248940 0.557887 0.342325
C -1.115788 -0.380742 -0.486485
C -2.096418 -1.185316 0.376332
C -2.746541 -2.327065 -0.402115
C -3.653072 -3.208127 0.453612
C -4.291184 -4.354127 -0.329550
C -5.194831 -5.241018 0.525186
C -5.831498 -6.393452 -0.250691
C -6.730710 -7.273588 0.615715
O -1.143461 3.039476 -1.398308
O -1.436205 5.604139 -0.796417
O -1.894790 0.340499 -1.456422
O -3.076867 -0.301954 0.937918
H 0.539874 6.538035 0.746930
H -0.143828 5.085312 1.481508
H 2.564162 5.350643 0.072678

H 2.297265 4.994063 1.768345
H 0.506978 5.417219 -1.475292
H 0.654073 1.082583 -1.549603
H 3.720287 3.168656 1.367765
H 5.258870 1.018419 0.148312
H 4.738400 0.651232 1.795304
H 4.651115 -1.966394 -1.185855
H 3.174443 -1.393113 2.169181
H 4.269532 -3.553361 2.418852
H 5.183722 -3.290495 0.832220
H 0.215052 -0.009916 1.151304
H -0.886089 1.307891 0.814794
H -0.463939 -1.084096 -1.015264
H -1.548975 -1.601301 1.226315
H -3.314054 -1.905929 -1.238070
H -1.949859 -2.939008 -0.838894
H -3.071162 -3.620429 1.286356
H -4.437692 -2.591664 0.900692
H -4.872239 -3.942898 -1.163009
H -3.503449 -4.968946 -0.780059
H -4.614731 -5.647768 1.361734
H -5.985637 -4.627214 0.971815
H -6.411901 -5.986978 -1.085850
H -5.040673 -7.005597 -0.697219
H -6.166534 -7.718079 1.439927
H -7.547530 -6.691168 1.050239
H -7.172720 -8.087610 0.036995
H -1.949903 4.943160 -1.288909
H -1.609538 1.274175 -1.472411
H -3.365734 0.252966 0.197828

2c-c113, ΔG = 0.2378 kcal/mol, population = 7.52 %
C 0.480354 5.549225 0.622045
C 1.829317 4.840719 0.531352
C 1.676862 3.362481 0.333350
C 0.538324 2.774643 -0.138539
C -0.564428 3.604337 -0.546597
C -0.392932 5.118064 -0.542742
C 0.367694 1.269589 -0.248250
N 2.786840 2.600906 0.619604
C 2.812965 1.269699 0.327678
C 1.710984 0.617479 -0.116060
C 4.044936 0.423984 0.417091
C 3.517511 -0.996137 0.112044

C 2.062473 -0.749095 -0.395041
O 1.403460 -1.620636 -0.945651
O 4.284532 -1.593060 -0.922588
C 3.450252 -1.836094 1.362179
C 3.974703 -3.047095 1.491995
C -0.633637 0.716361 0.811226
C -1.677987 -0.211241 0.204761
C -2.531061 -0.914577 1.267675
C -3.365629 -2.064241 0.702143
C -2.539241 -3.264458 0.238690
C -3.399022 -4.446439 -0.206566
C -2.576792 -5.647028 -0.671945
C -3.428499 -6.835574 -1.115892
C -2.594514 -8.027976 -1.581342
O -1.646980 3.141413 -0.934855
O -1.655032 5.757634 -0.503320
O -2.581973 0.505486 -0.654845
O -3.366712 0.052023 1.919647
H 0.619925 6.629978 0.604075
H -0.020943 5.288796 1.557295
H 2.407717 5.238467 -0.310510
H 2.422066 5.024593 1.429500
H 0.122325 5.368570 -1.483884
H -0.039331 1.046892 -1.239042
H 3.624560 3.059250 0.947876
H 4.774379 0.715863 -0.340612
H 4.523364 0.503554 1.394129
H 3.737513 -2.289904 -1.313673
H 2.909034 -1.379750 2.186336
H 3.880969 -3.599768 2.417976
H 4.522907 -3.516722 0.684979
H -0.075066 0.172332 1.575487
H -1.135734 1.541295 1.320230
H -1.159036 -0.968690 -0.388878
H -1.867054 -1.308145 2.041510
H -4.054797 -2.383290 1.489308
H -3.978213 -1.688153 -0.123130
H -1.883783 -2.974590 -0.587741
H -1.880070 -3.585802 1.053313
H -4.050595 -4.751866 0.619823
H -4.062164 -4.125318 -1.017751
H -1.926652 -5.340762 -1.499595
H -1.910364 -5.964506 0.138422
H -4.076173 -7.142451 -0.287789

H -4.095355 -6.517065 -1.924157
H -1.941242 -8.385571 -0.780945
H -3.226778 -8.862115 -1.893351
H -1.959904 -7.754898 -2.428633
H -2.291314 5.106981 -0.842634
H -2.244438 1.410977 -0.793748
H -3.727184 0.594859 1.202146

2c-c159, ΔG = 0.5955 kcal/mol, population = 4.11 %
C 0.002549 5.381440 -0.071296
C 1.343544 4.853256 0.430535
C 1.474290 3.372072 0.236766
C 0.715336 2.650560 -0.639772
C -0.212733 3.340744 -1.495861
C -0.247350 4.863949 -1.476444
C 0.793715 1.136885 -0.752989
N 2.453528 2.755799 0.981067
C 2.761915 1.443770 0.778346
C 2.032322 0.661884 -0.054233
C 3.939614 0.762713 1.402725
C 3.764892 -0.708886 0.965936
C 2.633092 -0.644477 -0.106699
O 2.374748 -1.586170 -0.843742
O 4.959090 -1.188429 0.366763
C 3.288133 -1.567184 2.110260
C 3.855481 -2.702674 2.493975
C -0.477977 0.442957 -0.175903
C -1.079037 -0.588474 -1.121719
C -2.206593 -1.395451 -0.467176
C -2.616758 -2.595611 -1.320791
C -3.722491 -3.458783 -0.709263
C -3.333338 -4.145941 0.600025
C -4.415432 -5.085506 1.129466
C -4.040238 -5.766244 2.445308
C -5.127468 -6.704984 2.965313
O -0.970370 2.752197 -2.281089
O -1.481092 5.335750 -1.985143
O -1.625140 0.031880 -2.297882
O -3.322263 -0.535928 -0.197343
H 0.000926 6.471243 -0.068570
H -0.804961 5.040700 0.581052
H 2.166100 5.340721 -0.105363
H 1.478832 5.089746 1.487864
H 0.575997 5.195831 -2.128864

H 0.862587 0.880026 -1.814117
H 3.016402 3.313133 1.607191
H 4.875369 1.156124 1.001636
H 3.956391 0.890561 2.486006
H 4.714035 -1.946530 -0.183672
H 2.402341 -1.195438 2.617576
H 3.454827 -3.276209 3.319909
H 4.742004 -3.086034 2.004772
H -0.216685 -0.047682 0.763917
H -1.234982 1.191653 0.065047
H -0.290190 -1.285014 -1.425759
H -1.856139 -1.740532 0.507040
H -2.937008 -2.226157 -2.299057
H -1.726651 -3.210987 -1.492130
H -4.611115 -2.842687 -0.545398
H -4.007284 -4.224582 -1.437964
H -2.405543 -4.710646 0.450111
H -3.114019 -3.394782 1.365305
H -5.346176 -4.523027 1.267542
H -4.630386 -5.851767 0.375633
H -3.108807 -6.325583 2.306906
H -3.827623 -4.999369 3.197733
H -5.338799 -7.497504 2.242460
H -4.832646 -7.179431 3.903870
H -6.060111 -6.163121 3.143640
H -1.830004 4.618083 -2.538814
H -1.361776 0.971781 -2.320851
H -3.460021 -0.037093 -1.016359

2c-c320, ΔG = 0.6583 kcal/mol, population = 3.70 %
C -0.245848 5.407471 -0.422111
C 0.956734 4.832962 0.321591
C 1.061680 3.346031 0.160222
C 0.469002 2.650712 -0.853999
C -0.271529 3.365595 -1.861755
C -0.252749 4.889023 -1.849995
C 0.517964 1.136601 -0.957044
N 1.838102 2.693955 1.091656
C 2.129208 1.369302 0.953751
C 1.570549 0.617278 -0.025888
C 3.104135 0.635316 1.822048
C 2.938983 -0.830733 1.361785
C 2.124075 -0.709126 0.039438
O 2.027865 -1.632134 -0.757636

O 4.202083 -1.418374 1.099509
C 2.103120 -1.623424 2.336868
C 2.431470 -2.810559 2.827509
C -0.864817 0.496343 -0.635698
C -1.275969 -0.560786 -1.646509
C -2.581503 -1.262493 -1.268721
C -2.539435 -2.047723 0.038362
C -1.382059 -3.039305 0.183481
C -1.377417 -3.779644 1.524321
C -2.569476 -4.710034 1.747130
C -2.476658 -5.500921 3.051870
C -3.670040 -6.427236 3.278005
O -0.909612 2.796732 -2.759155
O -1.353691 5.400900 -2.577144
O -1.451694 0.005202 -2.960197
O -2.949039 -2.162235 -2.326225
H -0.204650 6.496549 -0.421028
H -1.173275 5.104646 0.069707
H 1.882860 5.281498 -0.055734
H 0.904030 5.078064 1.384116
H 0.688075 5.186287 -2.340088
H 0.777820 0.873668 -1.985708
H 2.277224 3.230191 1.825959
H 4.126175 0.966801 1.630194
H 2.893963 0.780257 2.882619
H 4.049249 -2.148033 0.481030
H 1.157175 -1.161194 2.604643
H 1.771832 -3.334354 3.507148
H 3.368694 -3.289499 2.573604
H -0.822564 0.057084 0.361181
H -1.634949 1.270987 -0.599191
H -0.485659 -1.309830 -1.733075
H -3.355730 -0.487526 -1.182176
H -2.511927 -1.335312 0.867734
H -3.494743 -2.570429 0.123184
H -1.419562 -3.764941 -0.634344
H -0.430744 -2.513120 0.084086
H -0.455649 -4.365946 1.594986
H -1.332375 -3.047255 2.339100
H -3.500511 -4.134794 1.748084
H -2.642089 -5.409235 0.905654
H -1.552135 -6.088091 3.050518
H -2.392594 -4.800763 3.890049
H -3.760180 -7.154723 2.466982

H -3.574937 -6.981779 4.214184
H -4.603942 -5.860519 3.319227
H -1.636170 4.682536 -3.166999
H -1.337798 0.972265 -2.912080
H -2.818560 -1.665326 -3.146190

2c-c60, ΔG = 0.6827 kcal/mol, population = 3.55 %
C -0.571164 5.312130 0.975211
C 0.853327 4.767313 0.915990
C 0.885305 3.312973 0.552970
C -0.146081 2.664316 -0.062632
C -1.308984 3.409879 -0.466967
C -1.314511 4.923122 -0.290709
C -0.138910 1.172157 -0.338937
N 2.053065 2.644823 0.844416
C 2.244383 1.359810 0.430473
C 1.254256 0.648832 -0.162035
C 3.550380 0.635569 0.542712
C 3.197100 -0.794227 0.074684
C 1.772606 -0.638639 -0.541530
O 1.262893 -1.505893 -1.237571
O 4.101918 -1.226407 -0.929521
C 3.102133 -1.748571 1.238497
C 3.682549 -2.939592 1.290857
C -1.144597 0.407987 0.573197
C -2.006535 -0.571682 -0.209393
C -2.811082 -1.507433 0.697187
C -3.435209 -2.686064 -0.054989
C -2.442201 -3.565020 -0.825287
C -1.268418 -4.082040 0.008194
C -0.328548 -4.994129 -0.777663
C 0.890018 -5.437516 0.030928
C 1.861367 -6.302524 -0.769416
O -2.302013 2.878330 -0.984823
O -2.642569 5.412968 -0.277964
O -2.945419 0.114733 -1.057406
O -3.822140 -0.760111 1.388053
H -0.555677 6.396774 1.080350
H -1.098118 4.897191 1.837676
H 1.437542 5.318011 0.169848
H 1.358718 4.909749 1.873295
H -0.774688 5.332877 -1.159246
H -0.449199 1.019662 -1.376919
H 2.812390 3.150116 1.277804

H 4.294572 1.067827 -0.128429
H 3.949433 0.669740 1.557430
H 3.649814 -1.917138 -1.436122
H 2.479702 -1.404078 2.059558
H 3.557235 -3.584716 2.150772
H 4.306177 -3.302737 0.483872
H -0.585538 -0.138004 1.335552
H -1.785962 1.115024 1.102354
H -1.345192 -1.174548 -0.836501
H -2.145931 -1.886287 1.474058
H -3.970010 -3.293753 0.680839
H -4.185695 -2.293372 -0.747013
H -2.987974 -4.417221 -1.242027
H -2.050062 -3.012696 -1.684764
H -0.683995 -3.239771 0.389100
H -1.650355 -4.615341 0.886522
H -0.877602 -5.875398 -1.128761
H 0.012313 -4.465123 -1.674736
H 1.413550 -4.549450 0.397575
H 0.554525 -5.985818 0.917887
H 2.721584 -6.600921 -0.166001
H 1.374507 -7.212559 -1.130180
H 2.238107 -5.760884 -1.641249
H -3.175861 4.739990 -0.731880
H -2.716842 1.062799 -1.083030
H -4.228460 -0.200950 0.708845

2c-c145, ΔG = 0.7398 kcal/mol, population = 3.22 %
C -0.343923 5.433497 -0.532214
C 0.847558 4.854248 0.225870
C 0.923463 3.361709 0.101853
C 0.304247 2.649940 -0.885121
C -0.420426 3.353410 -1.911449
C -0.362936 4.875543 -1.944226
C 0.313073 1.132023 -0.941626
N 1.698015 2.721605 1.042451
C 1.946587 1.384522 0.950342
C 1.350873 0.613555 0.007770
C 2.910314 0.654126 1.834142
C 2.683950 -0.823273 1.442210
C 1.853908 -0.729520 0.126958
O 1.709898 -1.683437 -0.625362
O 3.919957 -1.469158 1.187834
C 1.838338 -1.538591 2.467368

C 2.137740 -2.709246 3.012419
C -1.087740 0.539887 -0.605032
C -1.535229 -0.527401 -1.589950
C -2.855914 -1.185539 -1.181867
C -2.794561 -1.986002 0.115267
C -1.694507 -3.045441 0.187469
C -1.707554 -3.820278 1.504241
C -0.619014 -4.891481 1.568640
C -0.526825 -5.622428 2.910931
C -1.759260 -6.460382 3.252775
O -1.073248 2.775768 -2.792693
O -1.450500 5.393452 -2.686986
O -1.710797 0.015423 -2.913088
O -3.286708 -2.060351 -2.235261
H -0.280134 6.521077 -0.561266
H -1.276644 5.163306 -0.031509
H 1.782350 5.275746 -0.161131
H 0.799541 5.123662 1.282969
H 0.585108 5.134977 -2.441627
H 0.568273 0.829638 -1.960771
H 2.162202 3.271593 1.750717
H 3.939457 0.939648 1.609221
H 2.726709 0.852626 2.890913
H 3.728208 -2.224413 0.612446
H 0.912892 -1.032497 2.727081
H 1.475397 -3.176859 3.728789
H 3.053571 -3.231793 2.766770
H -1.056106 0.122488 0.401509
H -1.834379 1.337785 -0.584310
H -0.765043 -1.298270 -1.666160
H -3.599492 -0.384913 -1.063663
H -2.684382 -1.286202 0.948076
H -3.770060 -2.463023 0.250307
H -1.807230 -3.742019 -0.648192
H -0.713657 -2.579198 0.069433
H -1.568717 -3.119003 2.336088
H -2.692614 -4.275287 1.647016
H -0.787243 -5.624083 0.770360
H 0.345370 -4.420042 1.356571
H 0.354002 -6.271555 2.898143
H -0.353821 -4.889624 3.706412
H -1.963889 -7.190906 2.465091
H -1.616568 -7.008379 4.186756
H -2.650179 -5.840289 3.368455

H -1.748035 4.666516 -3.258628
H -1.554875 0.977888 -2.890492
H -3.146728 -1.564299 -3.054459

2c-c189, ΔG = 0.7593 kcal/mol, population = 3.12 %
C -0.730367 5.144748 -0.129932
C 0.514943 4.602226 0.564411
C 0.724170 3.143091 0.291292
C 0.162985 2.477665 -0.761130
C -0.626621 3.215200 -1.714127
C -0.710122 4.731880 -1.590723
C 0.320304 0.980118 -0.976011
N 1.565567 2.490005 1.162567
C 1.928210 1.194746 0.945302
C 1.392445 0.464211 -0.062964
C 2.963671 0.477637 1.754580
C 2.882762 -0.968842 1.219124
C 2.006653 -0.836993 -0.065251
O 1.922808 -1.732894 -0.894028
O 4.173080 -1.442493 0.867350
C 2.162623 -1.870647 2.188938
C 2.597871 -3.056294 2.592013
C -1.020766 0.220044 -0.750862
C -1.352947 -0.751041 -1.873264
C -2.643770 -1.532076 -1.608393
C -2.585594 -2.472254 -0.409033
C -1.420842 -3.465038 -0.413077
C -1.445721 -4.442718 0.765085
C -1.313900 -3.768828 2.131810
C -1.076411 -4.751876 3.276609
C -0.967762 -4.066487 4.637526
O -1.223449 2.678141 -2.658446
O -1.846478 5.220415 -2.278428
O -1.503499 -0.076202 -3.136525
O -2.969689 -2.300958 -2.775747
H -0.764979 6.231035 -0.049002
H -1.630615 4.742473 0.340575
H 1.404207 5.143102 0.221043
H 0.452749 4.759657 1.642824
H 0.206289 5.125901 -2.058363
H 0.621860 0.817320 -2.014315
H 1.985490 3.008397 1.920747
H 3.958042 0.884975 1.563416
H 2.768437 0.552226 2.825244

H 4.045931 -2.153489 0.222140
H 1.208223 -1.488136 2.538763
H 2.017836 -3.660167 3.277632
H 3.548179 -3.454701 2.259971
H -0.962463 -0.322500 0.193089
H -1.840222 0.935527 -0.645315
H -0.530678 -1.459014 -1.996320
H -3.443086 -0.797157 -1.437722
H -2.559294 -1.865105 0.497849
H -3.531569 -3.022271 -0.376752
H -1.436358 -4.025359 -1.351122
H -0.469995 -2.926114 -0.390827
H -2.367792 -5.034616 0.736832
H -0.621127 -5.152938 0.643494
H -0.483205 -3.056784 2.095444
H -2.211665 -3.181893 2.348495
H -1.889941 -5.484824 3.299928
H -0.160717 -5.318604 3.076941
H -0.146522 -3.344624 4.650098
H -1.885928 -3.523547 4.876727
H -0.788281 -4.788067 5.437424
H -2.075419 4.535874 -2.928526
H -1.482171 0.888599 -2.990743
H -2.814894 -1.705916 -3.523543

2c-c3, ΔG = 0.7643 kcal/mol, population = 3.09 %
C -2.517703 2.282862 0.802051
C -1.154277 2.497033 1.450643
C -0.060484 1.768451 0.727590
C -0.168440 1.296745 -0.548533
C -1.363149 1.597295 -1.306063
C -2.397944 2.536526 -0.690071
C 0.926141 0.473178 -1.215793
N 1.118803 1.631285 1.427229
C 2.240451 1.165943 0.809484
C 2.199128 0.648189 -0.441347
C 3.604740 1.214524 1.422074
C 4.468226 0.433759 0.407179
C 3.531884 0.282179 -0.832397
O 3.946985 -0.084968 -1.924394
O 5.615632 1.188074 0.046156
C 4.799235 -0.942873 0.925361
C 6.014527 -1.473304 0.948903
C 0.519075 -1.013928 -1.375554

C 0.252775 -1.759074 -0.074382
C -0.545441 -3.055007 -0.263177
C -1.949961 -2.876656 -0.828773
C -2.813239 -1.831432 -0.123124
C -4.272043 -1.884934 -0.579286
C -5.132433 -0.725087 -0.072228
C -5.240467 -0.629386 1.450123
C -6.151760 0.510292 1.902460
O -1.573166 1.157999 -2.438859
O -3.640361 2.391412 -1.350986
O 1.473019 -2.080220 0.630891
O -0.661378 -3.714641 1.005786
H -3.255270 2.955184 1.239859
H -2.857350 1.259243 0.967401
H -0.904977 3.564323 1.459498
H -1.168372 2.172971 2.493409
H -2.015845 3.557854 -0.844253
H 1.066497 0.852769 -2.231855
H 1.180109 2.013525 2.359696
H 3.963425 2.242541 1.499197
H 3.616991 0.776812 2.421306
H 5.912852 0.854388 -0.812950
H 3.941525 -1.504722 1.282480
H 6.179705 -2.473933 1.327541
H 6.880841 -0.922522 0.604548
H -0.374325 -1.034621 -1.997468
H 1.301886 -1.535690 -1.934220
H -0.293877 -1.121798 0.621586
H 0.024310 -3.701118 -0.947236
H -1.877089 -2.637270 -1.892315
H -2.440452 -3.852977 -0.771982
H -2.751477 -1.981575 0.956457
H -2.418110 -0.831539 -0.321623
H -4.299128 -1.888010 -1.674347
H -4.710954 -2.837109 -0.259859
H -4.732556 0.216036 -0.462780
H -6.140796 -0.825135 -0.488742
H -5.612045 -1.580351 1.847495
H -4.247248 -0.486604 1.885068
H -7.166658 0.377146 1.518589
H -6.211164 0.568991 2.991498
H -5.783708 1.471948 1.535602
H -3.434036 1.946414 -2.190875
H 2.055882 -2.551276 0.020543

H 0.195061 -3.600269 1.442376

2c-c27, ΔG = 0.7919 kcal/mol, population = 2.95 %
C -2.032675 2.582161 1.104001
C -0.607190 2.770908 1.613591
C 0.388843 1.997783 0.801408
C 0.152930 1.543394 -0.463405
C -1.092473 1.889725 -1.111400
C -2.059791 2.828096 -0.392735
C 1.138955 0.661803 -1.219363
N 1.608987 1.786431 1.407084
C 2.637103 1.214912 0.719541
C 2.468106 0.713558 -0.527574
C 4.032577 1.112471 1.249283
C 4.746665 0.250792 0.183660
C 3.730244 0.210504 -0.998349
O 4.035915 -0.190001 -2.113774
O 5.947311 0.882952 -0.233563
C 4.951508 -1.161193 0.672148
C 6.118737 -1.790418 0.698136
C 0.625704 -0.793679 -1.333869
C 0.496141 -1.519251 0.007503
C -0.784127 -2.359866 0.110125
C -2.024205 -1.488240 0.234905
C -3.340298 -2.261315 0.220831
C -4.553044 -1.338232 0.330731
C -5.888515 -2.077059 0.279859
C -7.099467 -1.150396 0.383338
C -8.431310 -1.896083 0.322505
O -1.404107 1.479312 -2.231741
O -3.365031 2.673220 -0.919859
O 1.645039 -2.345517 0.291069
O -0.708487 -3.204686 1.263305
H -2.709137 3.270737 1.610197
H -2.375335 1.564915 1.304970
H -0.331017 3.830960 1.577362
H -0.528169 2.464962 2.658812
H -1.699637 3.850110 -0.590531
H 1.234325 1.041849 -2.240060
H 1.766105 2.153613 2.334261
H 4.504332 2.095201 1.304672
H 4.056988 0.669161 2.245741
H 6.182367 0.502412 -1.092290
H 4.040878 -1.660230 0.990491

H 6.195087 -2.810307 1.053105
H 7.032186 -1.305207 0.377483
H -0.331382 -0.770997 -1.851717
H 1.310031 -1.353638 -1.976388
H 0.480266 -0.805731 0.833359
H -0.862041 -2.985633 -0.791910
H -1.940866 -0.913635 1.163179
H -2.035215 -0.765888 -0.583231
H -3.407402 -2.840117 -0.707171
H -3.352351 -2.983853 1.041070
H -4.488707 -0.769099 1.265189
H -4.517385 -0.599089 -0.477641
H -5.950603 -2.647165 -0.654350
H -5.927232 -2.813539 1.090757
H -7.038751 -0.583767 1.318572
H -7.055676 -0.412369 -0.424660
H -9.278370 -1.210693 0.397498
H -8.529268 -2.446153 -0.617226
H -8.513472 -2.618821 1.138637
H -3.242582 2.270289 -1.796214
H 1.869270 -2.843300 -0.505687
H 0.218356 -3.480417 1.322043

2c-c30, ΔG = 0.8911 kcal/mol, population = 2.50 %
C -2.147718 2.472433 1.644877
C -0.674167 2.676352 1.981872
C 0.227621 1.925862 1.047605
C -0.155318 1.465496 -0.178678
C -1.479667 1.787180 -0.663911
C -2.350894 2.730374 0.163012
C 0.749878 0.608959 -1.055188
N 1.519049 1.744490 1.494133
C 2.468428 1.209232 0.676378
C 2.155630 0.703455 -0.540708
C 3.924353 1.156518 1.018590
C 4.521667 0.317701 -0.133155
C 3.361210 0.241209 -1.172795
O 3.532019 -0.152264 -2.319173
O 5.635070 0.988472 -0.703805
C 4.836412 -1.084886 0.321913
C 6.009551 -1.684833 0.170134
C 0.263968 -0.858898 -1.107013
C 0.330536 -1.590251 0.233711
C -0.838574 -2.562895 0.439738

C -2.151123 -1.836169 0.687731
C -3.360956 -2.766358 0.748836
C -4.679073 -2.034980 1.027254
C -5.027247 -0.921263 0.032507
C -5.135962 -1.384534 -1.420406
C -5.483592 -0.247801 -2.380018
O -1.932081 1.355798 -1.726819
O -3.709023 2.592336 -0.209977
O 1.580093 -2.292983 0.404728
O -0.566586 -3.403328 1.566296
H -2.767666 3.149154 2.232769
H -2.451684 1.448822 1.875851
H -0.418786 3.740629 1.923317
H -0.464765 2.362823 3.006566
H -2.006303 3.750479 -0.067939
H 0.703369 0.989882 -2.079034
H 1.781046 2.113221 2.396633
H 4.364116 2.155582 1.013848
H 4.095007 0.716853 2.002237
H 5.767195 0.614317 -1.587016
H 3.998441 -1.604288 0.777317
H 6.164603 -2.700697 0.510840
H 6.851806 -1.180116 -0.286575
H -0.754796 -0.857155 -1.490171
H 0.865862 -1.403832 -1.839381
H 0.313336 -0.879504 1.062205
H -0.930979 -3.186778 -0.462426
H -2.061057 -1.276108 1.624690
H -2.299357 -1.100714 -0.103600
H -3.432906 -3.315977 -0.194687
H -3.200133 -3.515917 1.527020
H -5.490880 -2.769817 1.039287
H -4.639216 -1.605381 2.033934
H -5.979886 -0.471183 0.331433
H -4.288472 -0.118086 0.098918
H -4.192988 -1.839360 -1.736370
H -5.893422 -2.172792 -1.490362
H -4.694157 0.507789 -2.387339
H -5.601314 -0.609708 -3.403766
H -6.415301 0.244490 -2.089184
H -3.695488 2.161691 -1.081542
H 1.779301 -2.767531 -0.412784
H 0.386624 -3.574050 1.537632

2c-c2, ΔG = 0.9030 kcal/mol, population = 2.45 %

C -2.284230 2.347796 1.259603
C -0.849841 2.594908 1.714590
C 0.146242 1.830889 0.893599
C -0.116207 1.308981 -0.339577
C -1.402561 1.569225 -0.948882
C -2.363057 2.526225 -0.245832
C 0.894274 0.468892 -1.112274
N 1.400680 1.714325 1.452299
C 2.436628 1.200433 0.732569
C 2.245844 0.636461 -0.483744
C 3.860869 1.239733 1.188497
C 4.598766 0.428185 0.101187
C 3.519130 0.229393 -1.008387
O 3.797975 -0.193981 -2.123195
O 5.680877 1.183223 -0.425946
C 5.014027 -0.925385 0.617152
C 6.232763 -1.437470 0.510854
C 0.461979 -1.014342 -1.218320
C 0.307811 -1.746333 0.108137
C -0.539582 -3.020312 -0.002524
C -1.987574 -2.800826 -0.427706
C -2.777656 -1.795978 0.409386
C -4.233986 -1.684304 -0.038598
C -5.056117 -0.731287 0.828685
C -6.527491 -0.615418 0.417944
C -6.739193 0.044733 -0.945725
O -1.750807 1.080749 -2.026103
O -3.679535 2.335778 -0.727458
O 1.583046 -2.096925 0.690983
O -0.543286 -3.688720 1.266385
H -2.963805 3.040606 1.755734
H -2.592930 1.333235 1.518754
H -0.607472 3.661069 1.636114
H -0.726775 2.325961 2.765795
H -2.020665 3.542171 -0.498247
H 0.928136 0.840317 -2.140915
H 1.574607 2.133793 2.354021
H 4.239743 2.263158 1.207061
H 3.976859 0.819046 2.188614
H 5.882760 0.815573 -1.298684
H 4.216043 -1.489825 1.090473
H 6.459883 -2.423259 0.896147
H 7.041643 -0.885444 0.048866

H -0.483528 -1.031295 -1.756177
H 1.186364 -1.548706 -1.840153
H -0.149922 -1.092776 0.851619
H -0.059184 -3.673751 -0.745817
H -2.003814 -2.498847 -1.477463
H -2.484121 -3.774792 -0.387517
H -2.735824 -2.085178 1.462852
H -2.314331 -0.808446 0.340856
H -4.256749 -1.354661 -1.081783
H -4.696509 -2.678159 -0.019704
H -5.000903 -1.069715 1.868893
H -4.601659 0.263407 0.797510
H -6.983442 -1.611648 0.418767
H -7.059469 -0.035501 1.178340
H -6.280677 1.036311 -0.969308
H -6.297877 -0.543144 -1.752863
H -7.802707 0.159406 -1.166869
H -3.581747 1.848781 -1.563429
H 2.099898 -2.564493 0.021368
H 0.357169 -3.603020 1.611335

2c-c5, ΔG = 0.9199 kcal/mol, population = 2.38 %
C -2.283155 2.200936 1.438052
C -0.850777 2.422017 1.915905
C 0.153675 1.771211 1.011755
C -0.109491 1.384099 -0.269722
C -1.399700 1.694269 -0.843966
C -2.370725 2.551957 -0.035897
C 0.893182 0.614296 -1.119471
N 1.411866 1.603307 1.549907
C 2.444261 1.161081 0.778161
C 2.250298 0.732041 -0.492234
C 3.869239 1.135574 1.234663
C 4.593734 0.414315 0.075898
C 3.521021 0.364949 -1.055787
O 3.796763 0.062747 -2.209276
O 5.710744 1.178954 -0.352754
C 4.946480 -1.002863 0.451381
C 6.162971 -1.525527 0.373451
C 0.476180 -0.867340 -1.282522
C 0.507628 -1.668906 0.019796
C -0.673088 -2.639100 0.162179
C -1.982762 -1.907277 0.414321
C -3.194740 -2.832648 0.513974

C -4.497034 -2.096384 0.839856
C -4.977022 -1.142434 -0.254461
C -6.314399 -0.479356 0.073084
C -6.806847 0.454134 -1.031393
O -1.745736 1.319738 -1.966524
O -3.686171 2.395396 -0.533487
O 1.748190 -2.389128 0.182001
O -0.427707 -3.519812 1.263937
H -2.972972 2.818753 2.012809
H -2.572005 1.156466 1.575429
H -0.629389 3.494629 1.958846
H -0.718223 2.037095 2.929280
H -2.047207 3.595896 -0.171014
H 0.905867 1.049719 -2.122068
H 1.585361 1.913532 2.494733
H 4.264829 2.146903 1.344683
H 3.978656 0.623879 2.191934
H 5.935780 0.874717 -1.243958
H 4.101519 -1.600812 0.780091
H 6.346404 -2.555774 0.650969
H 7.012207 -0.940884 0.042327
H -0.519542 -0.889157 -1.722263
H 1.143074 -1.341238 -2.007606
H 0.479309 -1.001999 0.883130
H -0.753648 -3.230679 -0.762570
H -1.879602 -1.335671 1.343055
H -2.140169 -1.183403 -0.385778
H -3.311654 -3.375610 -0.430884
H -3.004276 -3.583970 1.283391
H -5.281917 -2.835835 1.030438
H -4.369771 -1.538310 1.775009
H -4.232817 -0.360438 -0.427824
H -5.069454 -1.696076 -1.196530
H -7.064971 -1.255030 0.259891
H -6.215037 0.081960 1.008348
H -6.094549 1.264382 -1.201283
H -6.930240 -0.086095 -1.973952
H -7.769567 0.902911 -0.776369
H -3.582139 2.016930 -1.423310
H 1.962754 -2.822833 -0.653972
H 0.524692 -3.696449 1.245903

2c-c48, ΔG = 1.0806 kcal/mol, population = 1.81 %
C -2.053116 2.768665 1.216125

C -0.615512 2.854865 1.718481
C 0.328602 2.025041 0.899540
C 0.055537 1.568171 -0.357291
C -1.180405 1.971066 -0.992909
C -2.069502 2.991179 -0.284722
C 1.007277 0.664009 -1.133825
N 1.548647 1.773493 1.487857
C 2.549529 1.174685 0.784008
C 2.345469 0.670792 -0.456048
C 3.952373 1.048420 1.288128
C 4.632434 0.174623 0.211309
C 3.585785 0.134938 -0.945402
O 3.859543 -0.287532 -2.061509
O 5.823684 0.798001 -0.246933
C 4.849178 -1.232117 0.707797
C 6.006250 -1.879229 0.669997
C 0.439097 -0.765925 -1.306840
C 0.255925 -1.551838 -0.014679
C -0.793836 -2.665487 -0.135854
C -2.218820 -2.186935 -0.399458
C -2.762207 -1.172298 0.606864
C -4.176391 -0.683977 0.283178
C -5.256506 -1.763027 0.354562
C -6.664704 -1.215596 0.123519
C -7.744617 -2.293990 0.187862
O -1.536169 1.557471 -2.098519
O -3.383990 2.935261 -0.806368
O 1.493557 -2.137586 0.447728
O -0.799053 -3.437452 1.072172
H -2.671025 3.516839 1.712523
H -2.475337 1.786913 1.438327
H -0.267589 3.893766 1.686310
H -0.552666 2.539358 2.762148
H -1.629119 3.977758 -0.499569
H 1.110193 1.068614 -2.145194
H 1.733296 2.142720 2.409098
H 4.441003 2.023350 1.334597
H 3.987202 0.605440 2.284546
H 6.026417 0.415373 -1.112966
H 3.956320 -1.711472 1.097591
H 6.090017 -2.894614 1.035947
H 6.904939 -1.413178 0.285741
H -0.515207 -0.670400 -1.819960
H 1.097300 -1.328713 -1.975035

H -0.036436 -0.887426 0.798775
H -0.489763 -3.314027 -0.971001
H -2.269501 -1.764675 -1.406302
H -2.852161 -3.076573 -0.413923
H -2.743277 -1.606846 1.609897
H -2.100432 -0.304547 0.636930
H -4.437036 0.118539 0.980867
H -4.180339 -0.229916 -0.714006
H -5.056491 -2.544913 -0.384418
H -5.214234 -2.250410 1.335883
H -6.875300 -0.440386 0.867906
H -6.700297 -0.719770 -0.852444
H -7.573616 -3.065043 -0.568101
H -7.750835 -2.784638 1.164852
H -8.739263 -1.875742 0.018540
H -3.301964 2.481052 -1.662341
H 1.892506 -2.620611 -0.288252
H 0.128606 -3.511929 1.339107

2c-c116, ΔG = 1.0875 kcal/mol, population = 1.79 %
C -0.507908 5.153659 1.598834
C 0.905205 4.696281 1.249217
C 0.928546 3.295030 0.716323
C -0.164728 2.661208 0.198024
C -1.399919 3.388242 0.070416
C -1.430091 4.869553 0.427033
C -0.145659 1.210862 -0.256442
N 2.152449 2.667838 0.738422
C 2.322799 1.449101 0.152052
C 1.277835 0.750549 -0.354487
C 3.657205 0.795225 -0.028674
C 3.290177 -0.595769 -0.592265
C 1.777238 -0.461503 -0.948664
O 1.190508 -1.283405 -1.639575
O 4.033002 -0.862236 -1.771906
C 3.440800 -1.666821 0.458672
C 4.120693 -2.793421 0.299782
C -0.970502 0.287481 0.691688
C -1.944728 -0.609754 -0.059998
C -2.601306 -1.654721 0.845880
C -3.424266 -2.687950 0.072015
C -2.651798 -3.505178 -0.968677
C -1.461543 -4.285441 -0.410125
C -0.810571 -5.192687 -1.454622

C 0.351724 -6.036919 -0.923307
C 1.563602 -5.216192 -0.482994
O -2.445260 2.872351 -0.351953
O -2.755279 5.282615 0.704836
O -3.003894 0.150729 -0.665824
O -3.425272 -0.999271 1.821959
H -0.513879 6.218736 1.829755
H -0.872157 4.615396 2.476956
H 1.338227 5.356383 0.488937
H 1.557092 4.759067 2.122721
H -1.051933 5.403574 -0.459344
H -0.604397 1.162289 -1.248662
H 2.951683 3.165145 1.104078
H 4.261515 1.338690 -0.757304
H 4.217414 0.746443 0.906068
H 3.547427 -1.541465 -2.262545
H 2.921265 -1.464796 1.391135
H 4.175312 -3.529378 1.091192
H 4.645376 -3.013579 -0.621124
H -0.281643 -0.338320 1.262833
H -1.519667 0.888743 1.418503
H -1.389799 -1.128293 -0.846790
H -1.818838 -2.157820 1.415799
H -3.869636 -3.364379 0.807495
H -4.249903 -2.164461 -0.417933
H -3.349408 -4.209282 -1.433165
H -2.306352 -2.851768 -1.775939
H -0.715877 -3.590097 -0.017230
H -1.793644 -4.893609 0.439815
H -1.576087 -5.859192 -1.866094
H -0.455733 -4.579083 -2.291012
H -0.002503 -6.647128 -0.085166
H 0.660931 -6.739686 -1.703101
H 1.927171 -4.586895 -1.299040
H 1.327719 -4.557003 0.353710
H 2.386543 -5.861853 -0.168624
H -3.330972 4.641830 0.256236
H -2.795612 1.102274 -0.600471
H -3.958185 -0.365876 1.318926

2c-c249, ΔG = 1.1088 kcal/mol, population = 1.73 %
C -0.678121 5.235685 -0.479363
C 0.524833 4.733332 0.314594
C 0.734475 3.257500 0.150009

C 0.223670 2.529450 -0.885869
C -0.513165 3.204118 -1.922945
C -0.586697 4.725874 -1.906528
C 0.376155 1.021459 -0.994076
N 1.523752 2.655106 1.103273
C 1.903626 1.352505 0.973655
C 1.418472 0.565701 -0.017929
C 2.909122 0.687366 1.861967
C 2.862645 -0.785110 1.396215
C 2.050891 -0.723725 0.065590
O 2.021481 -1.655712 -0.726253
O 4.170202 -1.269218 1.136186
C 2.092794 -1.642132 2.368741
C 2.501838 -2.810730 2.842762
C -0.976986 0.287442 -0.754129
C -1.275139 -0.768360 -1.806520
C -2.556870 -1.550753 -1.509389
C -2.513717 -2.387646 -0.235286
C -1.313720 -3.329352 -0.110225
C -1.398184 -4.265614 1.098048
C -1.422948 -3.534420 2.442965
C -1.209224 -4.446768 3.653521
C -2.307466 -5.493229 3.844583
O -1.072385 2.605022 -2.852823
O -1.680640 5.174635 -2.684045
O -1.415642 -0.187485 -3.117279
O -2.837160 -2.420763 -2.616461
H -0.705840 6.325155 -0.472892
H -1.605463 4.871697 -0.030701
H 1.435731 5.244214 -0.018179
H 0.410651 4.964130 1.375515
H 0.356828 5.081230 -2.350621
H 0.705526 0.786928 -2.009884
H 1.904038 3.217294 1.850927
H 3.907685 1.094176 1.692681
H 2.667316 0.812760 2.918447
H 4.079218 -2.005553 0.513573
H 1.123952 -1.242592 2.653624
H 1.885164 -3.382933 3.523622
H 3.465522 -3.225312 2.575012
H -0.954764 -0.176699 0.232173
H -1.794153 1.013042 -0.736664
H -0.439131 -1.469409 -1.863232
H -3.374488 -0.821677 -1.420661

H -2.549988 -1.708441 0.618932
H -3.439157 -2.971060 -0.196784
H -1.232011 -3.924215 -1.023372
H -0.390224 -2.749055 -0.035486
H -2.283462 -4.901828 1.001624
H -0.532760 -4.937131 1.081074
H -0.641256 -2.769009 2.435406
H -2.372349 -3.002454 2.561429
H -0.241131 -4.949800 3.553583
H -1.141902 -3.829004 4.554269
H -3.288287 -5.017091 3.930357
H -2.350565 -6.189787 3.005105
H -2.140511 -6.079232 4.750968
H -1.885857 4.446574 -3.293586
H -1.365800 0.784197 -3.046941
H -2.682489 -1.885787 -3.408071

2c-c256, ΔG = 1.2299 kcal/mol, population = 1.41 %
C 0.675611 5.561580 1.338114
C 1.922472 4.691635 1.211030
C 1.597083 3.312843 0.720836
C 0.442873 2.980596 0.080078
C -0.505933 4.025261 -0.246735
C -0.124030 5.474153 0.050841
C 0.098614 1.545604 -0.285750
N 2.586986 2.364108 0.915902
C 2.506772 1.151237 0.312897
C 1.367533 0.740130 -0.307783
C 3.656668 0.201203 0.197832
C 3.043584 -1.009061 -0.541545
C 1.627889 -0.509916 -0.952095
O 0.915835 -1.148013 -1.730205
O 3.800756 -1.291039 -1.709788
C 2.891842 -2.199715 0.371395
C 3.340481 -3.419301 0.108484
C -0.943705 0.976970 0.704440
C -1.316656 -0.482351 0.453112
C -2.503561 -0.926276 1.329247
C -2.494787 -2.417517 1.671701
C -2.482286 -3.383546 0.488376
C -2.584856 -4.843346 0.933082
C -2.437421 -5.857616 -0.204115
C -3.538247 -5.788257 -1.264202
C -3.398996 -6.872959 -2.331004

O -1.592802 3.803658 -0.781439
O -1.286458 6.280266 0.105576
O -1.694009 -0.695230 -0.913312
O -3.733584 -0.526921 0.710688
H 0.955094 6.596173 1.536969
H 0.055519 5.213789 2.167879
H 2.631032 5.147694 0.510024
H 2.439824 4.617096 2.170083
H 0.515144 5.800021 -0.785231
H -0.356984 1.539498 -1.278180
H 3.453247 2.649466 1.349680
H 4.459666 0.631934 -0.402609
H 4.068488 -0.056485 1.174656
H 3.240331 -1.822286 -2.293660
H 2.359056 -1.997309 1.296081
H 3.189607 -4.231981 0.807268
H 3.881080 -3.640712 -0.803040
H -0.549629 1.062132 1.721140
H -1.843211 1.591788 0.643655
H -0.462836 -1.122057 0.694240
H -2.454924 -0.372242 2.269667
H -1.622077 -2.608494 2.306711
H -3.375995 -2.615288 2.290779
H -3.308036 -3.142933 -0.185065
H -1.565022 -3.249985 -0.091768
H -1.810318 -5.038979 1.682536
H -3.545046 -5.001415 1.437980
H -1.464053 -5.715869 -0.688428
H -2.423044 -6.867328 0.220434
H -4.513254 -5.878824 -0.772795
H -3.527696 -4.806609 -1.745582
H -2.442361 -6.786745 -2.853303
H -3.443250 -7.870079 -1.884837
H -4.193320 -6.805301 -3.077587
H -1.968274 5.781741 -0.375810
H -0.877855 -0.875168 -1.416915
H -3.608762 -0.672141 -0.238624

2c-c66, ΔG = 1.2450 kcal/mol, population = 1.37 %
C -2.054095 2.724794 0.924474
C -0.654296 2.850135 1.518496
C 0.357885 2.055850 0.748071
C 0.180602 1.637654 -0.538354
C -1.013351 2.039361 -1.247996

C -1.985937 2.999494 -0.566147
C 1.176176 0.734620 -1.255223
N 1.532337 1.785647 1.418082
C 2.579724 1.196368 0.775799
C 2.465999 0.730324 -0.491202
C 3.939838 1.036413 1.379334
C 4.687360 0.181052 0.331678
C 3.736273 0.198900 -0.904543
O 4.090784 -0.184789 -2.011348
O 5.924960 0.790179 -0.004401
C 4.829944 -1.247560 0.793178
C 5.976458 -1.911516 0.856441
C 0.618905 -0.699579 -1.421452
C 0.403753 -1.441453 -0.100605
C -0.903744 -2.245011 -0.071208
C -2.122297 -1.338482 0.011490
C -3.456339 -2.076189 -0.068748
C -4.647356 -1.122780 0.040762
C -6.011634 -1.789681 -0.150112
C -6.367823 -2.822545 0.920450
C -7.772604 -3.396124 0.742969
O -1.276456 1.659192 -2.391212
O -3.262596 2.902765 -1.171358
O 1.513642 -2.305162 0.223192
O -0.905713 -3.107654 1.071291
H -2.733023 3.428927 1.405313
H -2.444267 1.717604 1.085521
H -0.337333 3.899233 1.520939
H -0.646850 2.521455 2.559726
H -1.577922 4.010944 -0.720855
H 1.340224 1.126864 -2.262439
H 1.645232 2.121580 2.363402
H 4.436592 2.002197 1.488533
H 3.894260 0.566979 2.363089
H 6.196539 0.423616 -0.858407
H 3.892618 -1.728867 1.056898
H 6.006352 -2.941796 1.187676
H 6.918125 -1.447248 0.591009
H -0.312735 -0.633714 -1.980358
H 1.310816 -1.274191 -2.042504
H 0.368572 -0.740591 0.735383
H -0.958231 -2.855365 -0.985426
H -2.062652 -0.780140 0.951607
H -2.077228 -0.603859 -0.794711

H -3.512735 -2.616767 -1.020694
H -3.499695 -2.829001 0.720389
H -4.621186 -0.626433 1.017851
H -4.532391 -0.329089 -0.705180
H -6.785853 -1.014936 -0.160114
H -6.045571 -2.267882 -1.136128
H -5.640682 -3.638900 0.904304
H -6.283152 -2.355780 1.908012
H -7.873749 -3.889670 -0.227393
H -8.006228 -4.131174 1.516308
H -8.527805 -2.607135 0.792469
H -3.103216 2.502261 -2.043040
H 1.765326 -2.792758 -0.571657
H 0.009232 -3.411271 1.167515

2c-c259, ΔG = 1.2469 kcal/mol, population = 1.37 %
C 0.942546 5.385419 1.670532
C 2.073077 4.407705 1.361921
C 1.562770 3.121294 0.785292
C 0.333572 2.970822 0.219714
C -0.522495 4.130485 0.076095
C 0.037582 5.499237 0.457341
C -0.189153 1.619667 -0.238438
N 2.456298 2.062563 0.810358
C 2.203682 0.932981 0.102358
C 0.984336 0.705529 -0.457565
C 3.231727 -0.112271 -0.198937
C 2.432617 -1.176494 -0.984138
C 1.061637 -0.488126 -1.241458
O 0.234877 -0.963433 -2.022748
O 3.068848 -1.443174 -2.224912
C 2.189840 -2.416597 -0.159801
C 2.408645 -3.657291 -0.572544
C -1.188256 1.047827 0.796461
C -1.664547 -0.363010 0.470567
C -2.749925 -0.867785 1.445424
C -2.655370 -2.372763 1.709372
C -2.649964 -3.270641 0.473507
C -2.555282 -4.754766 0.829853
C -2.465695 -5.682221 -0.385613
C -1.167310 -5.545529 -1.184035
C -1.084049 -6.524858 -2.353265
O -1.669568 4.068358 -0.367160
O -1.021300 6.411127 0.682705

O -2.213771 -0.434704 -0.851909
O -4.046374 -0.483228 0.973012
H 1.348776 6.363353 1.928634
H 0.356205 5.028388 2.520648
H 2.771034 4.852497 0.643153
H 2.652061 4.191054 2.262238
H 0.639345 5.833928 -0.402625
H -0.730018 1.752798 -1.177510
H 3.375233 2.212444 1.201919
H 4.025756 0.291817 -0.829069
H 3.689395 -0.504053 0.710459
H 2.404373 -1.838227 -2.807780
H 1.782241 -2.232110 0.830110
H 2.192896 -4.503610 0.066458
H 2.817486 -3.867419 -1.552812
H -0.706728 1.029656 1.778378
H -2.046243 1.719436 0.857971
H -0.815245 -1.047103 0.542010
H -2.619958 -0.353929 2.400566
H -1.737199 -2.548193 2.282102
H -3.487014 -2.649694 2.365116
H -3.555775 -3.099595 -0.116423
H -1.811245 -2.996585 -0.168513
H -1.677572 -4.916780 1.466670
H -3.426018 -5.032286 1.433014
H -2.560630 -6.720476 -0.049941
H -3.318980 -5.496321 -1.048551
H -1.067087 -4.524991 -1.562626
H -0.318169 -5.703987 -0.510650
H -1.906211 -6.365437 -3.056068
H -0.147954 -6.410732 -2.904352
H -1.142973 -7.559464 -2.004739
H -1.791539 6.028280 0.229586
H -1.479061 -0.617211 -1.467529
H -4.000539 -0.547140 0.007284

2c-c177, ΔG = 1.2607 kcal/mol, population = 1.34 %
C -0.512292 5.361938 -0.241647
C 0.735654 4.786533 0.422715
C 0.859505 3.307761 0.205685
C 0.218113 2.630575 -0.791252
C -0.578854 3.364671 -1.740091
C -0.579853 4.887337 -1.682109
C 0.280155 1.119535 -0.940189

N 1.704189 2.645244 1.067172
C 1.995083 1.326186 0.884298
C 1.376201 0.586292 -0.067943
C 3.036332 0.589236 1.668003
C 2.874593 -0.867813 1.179531
C 1.933996 -0.739777 -0.058041
O 1.766502 -1.656987 -0.850156
O 4.128614 -1.391411 0.770192
C 2.183432 -1.722187 2.211946
C 2.630843 -2.891288 2.648715
C -1.086253 0.458224 -0.594326
C -1.533887 -0.563140 -1.627641
C -2.864739 -1.222863 -1.259018
C -2.831368 -2.061072 0.014358
C -1.721040 -3.110289 0.086169
C -1.828060 -3.990137 1.330319
C -0.697848 -5.014481 1.434039
C -0.833435 -5.988573 2.608247
C -0.778889 -5.322814 3.983928
O -1.249232 2.816226 -2.626731
O -1.721244 5.404044 -2.340337
O -1.684823 0.031093 -2.931528
O -3.283965 -2.063340 -2.344554
H -0.489794 6.451027 -0.207752
H -1.408660 5.026263 0.285172
H 1.633996 5.266471 0.017854
H 0.730621 4.991821 1.495121
H 0.332218 5.213411 -2.206867
H 0.505482 0.889457 -1.984709
H 2.183694 3.170754 1.783754
H 4.037015 0.949208 1.422448
H 2.891778 0.701203 2.743482
H 3.943682 -2.128172 0.169437
H 1.243946 -1.320115 2.580856
H 2.078031 -3.461177 3.383582
H 3.565179 -3.308880 2.295548
H -1.007903 -0.020004 0.382263
H -1.856446 1.227862 -0.499961
H -0.771139 -1.338042 -1.729255
H -3.604728 -0.420421 -1.130137
H -2.754549 -1.386918 0.871655
H -3.804603 -2.553376 0.103419
H -1.754132 -3.737078 -0.809416
H -0.742598 -2.622350 0.084830

H -1.832108 -3.350148 2.218243
H -2.791706 -4.513100 1.324650
H -0.657876 -5.588633 0.502211
H 0.258706 -4.486001 1.510430
H -1.774803 -6.540085 2.509099
H -0.034740 -6.733537 2.541183
H 0.147617 -4.755887 4.106861
H -1.610730 -4.631845 4.132633
H -0.821352 -6.066356 4.782758
H -2.016730 4.702078 -2.943397
H -1.625614 1.001340 -2.850176
H -3.121069 -1.545355 -3.145919

2c-c262, ΔG = 1.2701 kcal/mol, population = 1.32 %
C -0.813215 5.236763 -0.360427
C 0.445341 4.754883 0.355716
C 0.680089 3.286649 0.159404
C 0.127615 2.557785 -0.854500
C -0.679849 3.228531 -1.840672
C -0.790580 4.747741 -1.797299
C 0.307682 1.053967 -0.989830
N 1.535509 2.693390 1.059405
C 1.925969 1.397069 0.902318
C 1.399443 0.607271 -0.065195
C 2.984959 0.743147 1.734125
C 2.942113 -0.726811 1.259948
C 2.045987 -0.676933 -0.016010
O 1.973134 -1.611815 -0.801798
O 4.243626 -1.170944 0.909864
C 2.266289 -1.610960 2.276750
C 2.766635 -2.744765 2.747565
C -1.019237 0.289880 -0.705844
C -1.348387 -0.751792 -1.763765
C -2.641507 -1.510268 -1.451535
C -2.588045 -2.371590 -0.193984
C -1.420602 -3.360144 -0.129432
C -1.507093 -4.332550 1.050805
C -1.354491 -3.664179 2.418719
C -1.458117 -4.627192 3.605983
C -0.353118 -5.682789 3.658458
O -1.272711 2.630138 -2.750014
O -1.938336 5.178156 -2.504632
O -1.491300 -0.160784 -3.069342
O -2.967663 -2.352520 -2.566977

H -0.867799 6.324987 -0.336656
H -1.703870 4.843179 0.135169
H 1.322860 5.292659 -0.021276
H 0.386477 4.968897 1.424687
H 0.116617 5.133224 -2.289351
H 0.598761 0.839127 -2.021422
H 1.946931 3.257025 1.789280
H 3.966907 1.168963 1.520169
H 2.793049 0.856547 2.802102
H 4.133189 -1.920958 0.307198
H 1.290830 -1.262396 2.604045
H 2.220388 -3.337978 3.469054
H 3.738592 -3.107621 2.438166
H -0.946062 -0.194493 0.268272
H -1.847130 1.000038 -0.636594
H -0.527609 -1.468290 -1.837443
H -3.439158 -0.763822 -1.330406
H -2.573821 -1.707915 0.672851
H -3.531760 -2.923273 -0.137210
H -1.391268 -3.929236 -1.061610
H -0.472121 -2.818731 -0.068103
H -2.465086 -4.865070 1.014035
H -0.729219 -5.091129 0.930996
H -0.387484 -3.150515 2.454253
H -2.117962 -2.889778 2.533670
H -1.436621 -4.044135 4.531710
H -2.434404 -5.123510 3.580626
H -0.432902 -6.290041 4.562720
H -0.396568 -6.360136 2.803600
H 0.634709 -5.214582 3.658091
H -2.157529 4.454822 -3.114920
H -1.500853 0.811187 -2.982678
H -2.805219 -1.808774 -3.351371

2c-c402, ΔG = 1.3799 kcal/mol, population = 1.09 %
C -1.420745 3.878497 1.773747
C -0.020527 4.048985 1.192169
C 0.579272 2.740437 0.770314
C -0.142213 1.606786 0.544366
C -1.586804 1.678358 0.596755
C -2.247768 3.036142 0.821009
C 0.502329 0.267437 0.213357
N 1.947826 2.755854 0.574849
C 2.585988 1.677927 0.054855

C 1.936953 0.503010 -0.157169
C 4.023249 1.675733 -0.359996
C 4.272165 0.207344 -0.773611
C 2.857011 -0.439788 -0.712350
O 2.646764 -1.588756 -1.103553
O 4.751420 0.164808 -2.111340
C 5.174995 -0.498954 0.204866
C 6.284789 -1.148918 -0.117400
C 0.420303 -0.759060 1.382169
C -0.642828 -1.854325 1.264931
C -0.513392 -2.703857 -0.007716
C -1.597727 -3.769897 -0.133658
C -3.034715 -3.242722 -0.112492
C -3.354608 -2.226326 -1.209080
C -4.812872 -1.772495 -1.193513
C -5.133964 -0.726702 -2.260180
C -6.590429 -0.266997 -2.230183
O -2.313076 0.697654 0.428215
O -3.570634 2.857743 1.292365
O -0.562795 -2.701593 2.422439
O 0.755209 -3.379680 -0.009969
H -1.889644 4.851261 1.921590
H -1.369239 3.376909 2.743164
H -0.054735 4.708652 0.317403
H 0.642088 4.528236 1.915827
H -2.267797 3.532508 -0.162377
H -0.022699 -0.147211 -0.649499
H 2.453403 3.616896 0.725729
H 4.184417 2.333559 -1.215736
H 4.678090 2.007630 0.446883
H 4.646674 -0.744391 -2.426035
H 4.843853 -0.456975 1.238688
H 6.881969 -1.642055 0.638726
H 6.638234 -1.198875 -1.139661
H 1.392072 -1.249025 1.481368
H 0.254234 -0.222499 2.317887
H -1.628644 -1.397830 1.288295
H -0.567703 -2.044511 -0.876567
H -1.418317 -4.305120 -1.070602
H -1.462220 -4.492374 0.674717
H -3.712170 -4.097066 -0.210617
H -3.252401 -2.799526 0.863903
H -2.718890 -1.344944 -1.095314
H -3.117980 -2.662759 -2.186618

H -5.469236 -2.640467 -1.327155
H -5.048331 -1.360060 -0.205480
H -4.474838 0.136805 -2.122527
H -4.899311 -1.136922 -3.248300
H -7.270480 -1.107107 -2.394790
H -6.840662 0.176045 -1.262498
H -6.791245 0.480450 -3.000915
H -3.816089 1.953696 1.031632
H 0.205945 -3.272553 2.281015
H 1.419821 -2.773964 -0.387619

2c-c70, ΔG = 1.4320 kcal/mol, population = 1.00 %
C -2.289093 2.349711 0.900745
C -0.910435 2.558939 1.519910
C 0.168990 1.858434 0.749360
C 0.039190 1.445585 -0.544428
C -1.173564 1.766472 -1.263627
C -2.218862 2.649619 -0.584960
C 1.110453 0.634653 -1.261978
N 1.355406 1.673738 1.427922
C 2.453920 1.185089 0.786745
C 2.390740 0.730074 -0.487946
C 3.817503 1.131054 1.400742
C 4.639730 0.348635 0.351976
C 3.704935 0.311867 -0.895592
O 4.101134 -0.023250 -2.003943
O 5.833764 1.050368 0.041638
C 4.877793 -1.072645 0.797326
C 6.069818 -1.647756 0.883618
C 0.681956 -0.841741 -1.445747
C 0.544939 -1.619657 -0.133447
C -0.726695 -2.477238 -0.072222
C -1.973974 -1.619082 0.065973
C -3.286721 -2.397377 0.059327
C -4.496542 -1.472532 0.188629
C -5.831456 -2.215891 0.203179
C -7.059108 -1.300135 0.246162
C -7.161371 -0.453567 1.515556
O -1.396653 1.378108 -2.412473
O -3.477981 2.475573 -1.208985
O 1.700548 -2.441289 0.134752
O -0.654453 -3.354573 1.056545
H -3.021102 2.999069 1.380722
H -2.613907 1.316845 1.040808

H -0.668531 3.627333 1.553861
H -0.896601 2.204863 2.552713
H -1.878376 3.688132 -0.721529
H 1.247231 1.049489 -2.264056
H 1.432906 2.001087 2.379686
H 4.233130 2.133042 1.522188
H 3.803073 0.650566 2.379925
H 6.143272 0.715447 -0.812526
H 3.972025 -1.626633 1.026867
H 6.170852 -2.677478 1.202237
H 6.978875 -1.107608 0.650132
H -0.254371 -0.855063 -2.001182
H 1.421545 -1.339174 -2.078196
H 0.512269 -0.937368 0.717526
H -0.789052 -3.076536 -0.993223
H -1.889221 -1.050826 0.997891
H -1.994895 -0.890878 -0.746940
H -3.363392 -2.973280 -0.869626
H -3.288801 -3.121865 0.877872
H -4.390723 -0.881781 1.103537
H -4.492098 -0.754103 -0.639358
H -5.892737 -2.849501 -0.687990
H -5.857560 -2.893675 1.064494
H -7.045883 -0.642870 -0.630080
H -7.959625 -1.914770 0.153850
H -8.088290 0.123870 1.529480
H -7.148697 -1.086225 2.407552
H -6.333194 0.252664 1.598790
H -3.281439 2.097798 -2.083137
H 1.939964 -2.908870 -0.675731
H 0.274465 -3.622839 1.116993

2c-c244, ΔG = 1.4634 kcal/mol, population = 0.95 %
C -1.412579 4.868799 -0.521453
C -0.158100 4.577504 0.296447
C 0.281552 3.149217 0.168521
C -0.098047 2.323121 -0.850750
C -0.906314 2.858018 -1.916497
C -1.216988 4.350240 -1.934025
C 0.289675 0.852266 -0.917787
N 1.146085 2.704983 1.141929
C 1.713979 1.468973 1.060944
C 1.354969 0.581052 0.102166
C 2.804635 1.000836 1.971972

C 3.031182 -0.462116 1.531594
C 2.163204 -0.601308 0.239441
O 2.237718 -1.574724 -0.497802
O 4.397884 -0.660941 1.196063
C 2.525339 -1.431805 2.564894
C 3.216158 -2.447536 3.063316
C -0.948095 -0.074464 -0.728027
C -1.067455 -1.153457 -1.797312
C -2.354147 -1.974366 -1.655036
C -2.460862 -2.797316 -0.377624
C -1.296805 -3.753472 -0.112921
C -1.550573 -4.697037 1.068263
C -1.819470 -4.004354 2.409031
C -0.697823 -3.072252 2.863771
C -0.940919 -2.483832 4.251802
O -1.342385 2.168321 -2.849716
O -2.353187 4.606716 -2.737803
O -1.048699 -0.600458 -3.126116
O -2.466089 -2.859994 -2.778305
H -1.611232 5.940218 -0.540005
H -2.278084 4.372365 -0.076214
H 0.666432 5.218020 -0.037112
H -0.322314 4.808149 1.350880
H -0.332494 4.839456 -2.372401
H 0.695221 0.655450 -1.913418
H 1.430936 3.341698 1.872081
H 3.717473 1.578366 1.815657
H 2.524115 1.093012 3.022350
H 4.440899 -1.448978 0.635290
H 1.503069 -1.256764 2.882642
H 2.777588 -3.112775 3.795983
H 4.239554 -2.639332 2.766382
H -0.893200 -0.546022 0.253777
H -1.858463 0.530176 -0.728990
H -0.206308 -1.822212 -1.739363
H -3.194705 -1.266183 -1.681935
H -2.584797 -2.108597 0.461250
H -3.392002 -3.369913 -0.436307
H -1.116340 -4.351211 -1.009187
H -0.379042 -3.187134 0.063729
H -2.401062 -5.343854 0.828182
H -0.683961 -5.356981 1.180385
H -2.756612 -3.440732 2.358367
H -1.973069 -4.772806 3.173922

H 0.249721 -3.619797 2.855386
H -0.581532 -2.258471 2.142750
H -0.128475 -1.820797 4.557755
H -1.868354 -1.905533 4.274603
H -1.024343 -3.273807 5.002572
H -2.425437 3.847863 -3.340011
H -1.249944 0.353846 -3.075301
H -2.204337 -2.328326 -3.544230

2c-c4, ΔG = 1.4790 kcal/mol, population = 0.92 %
C -2.183170 2.309276 1.262822
C -0.747285 2.537196 1.727670
C 0.245731 1.802874 0.876429
C -0.022732 1.333323 -0.375988
C -1.308281 1.617013 -0.973651
C -2.263727 2.550762 -0.234233
C 0.970852 0.489031 -1.160249
N 1.502129 1.651904 1.424902
C 2.535863 1.186645 0.670307
C 2.335104 0.680022 -0.569419
C 3.970512 1.236784 1.096535
C 4.694782 0.489795 -0.034449
C 3.610206 0.354836 -1.145386
O 3.886869 0.028099 -2.293620
O 5.785132 1.277343 -0.518987
C 5.168354 -0.904461 0.302148
C 4.812090 -1.647806 1.342269
C 0.545340 -1.000538 -1.209956
C 0.306583 -1.660204 0.141154
C -0.438408 -2.996965 0.030244
C -1.864121 -2.885198 -0.502576
C -2.748889 -1.862468 0.210171
C -4.191253 -1.872449 -0.290680
C -5.069556 -0.836306 0.408209
C -6.510037 -0.811325 -0.100376
C -7.368691 0.241658 0.598356
O -1.660866 1.160277 -2.063296
O -3.581751 2.384168 -0.721529
O 1.523458 -1.866880 0.888226
O -0.493602 -3.604196 1.328271
H -2.861459 2.981603 1.787996
H -2.492979 1.284330 1.478325
H -0.504255 3.605380 1.691134
H -0.624765 2.227194 2.767574

H -1.918931 3.575063 -0.445519
H 0.973479 0.834351 -2.197520
H 1.680687 2.027131 2.345030
H 4.327152 2.267495 1.134255
H 4.121449 0.798904 2.082788
H 5.962732 0.984493 -1.425059
H 5.840340 -1.316134 -0.445774
H 5.198077 -2.652348 1.460954
H 4.123331 -1.302661 2.102544
H -0.371756 -1.050485 -1.794939
H 1.301914 -1.567000 -1.762398
H -0.279517 -1.000809 0.780741
H 0.136226 -3.650079 -0.642380
H -1.824978 -2.658059 -1.570692
H -2.316788 -3.877843 -0.421413
H -2.732943 -2.054517 1.286472
H -2.343345 -0.857453 0.068605
H -4.199112 -1.682528 -1.370246
H -4.619463 -2.871741 -0.151485
H -5.071219 -1.033842 1.486699
H -4.626798 0.155947 0.275699
H -6.505433 -0.621535 -1.179143
H -6.960319 -1.800715 0.034504
H -7.410832 0.060124 1.675688
H -6.957118 1.243164 0.447933
H -8.393215 0.240981 0.219732
H -3.485248 1.935635 -1.579047
H 2.202030 -2.223451 0.300126
H 0.366768 -3.424917 1.735263

2c-c56, ΔG = 1.5016 kcal/mol, population = 0.89 %
C -2.443048 2.360445 0.715464
C -1.086686 2.624656 1.360243
C 0.020948 1.901238 0.653972
C -0.063992 1.439265 -0.627361
C -1.258413 1.717434 -1.393747
C -2.335814 2.608336 -0.777584
C 1.035066 0.608057 -1.278344
N 1.180836 1.744295 1.381651
C 2.298966 1.221248 0.804923
C 2.281245 0.715900 -0.451627
C 3.636549 1.181093 1.475119
C 4.492863 0.352929 0.491163
C 3.605826 0.270609 -0.788946

O 4.042828 -0.112622 -1.866201
O 5.701661 1.035682 0.194804
C 4.707634 -1.048783 1.004102
C 5.890601 -1.635002 1.131046
C 0.599445 -0.867427 -1.453513
C 0.312654 -1.590383 -0.138414
C -0.865131 -2.569356 -0.221611
C -2.197923 -1.859789 -0.410941
C -3.394072 -2.810124 -0.442879
C -4.706390 -2.125758 -0.841439
C -5.099622 -0.916881 0.015280
C -5.228414 -1.212805 1.509407
C -5.705532 -0.001670 2.309687
O -1.442502 1.286038 -2.534101
O -3.572280 2.395330 -1.432565
O 1.476161 -2.280220 0.366897
O -0.924228 -3.325537 0.993661
H -3.203933 3.007510 1.151558
H -2.744546 1.324996 0.886089
H -0.862446 3.697361 1.343341
H -1.095321 2.324856 2.409973
H -2.005250 3.646503 -0.939770
H 1.220594 1.000069 -2.282157
H 1.222187 2.107277 2.322664
H 4.055731 2.183964 1.574783
H 3.578565 0.738890 2.470807
H 6.034849 0.669084 -0.637197
H 3.792557 -1.579005 1.251153
H 5.973813 -2.650299 1.497510
H 6.809786 -1.118225 0.884713
H -0.279216 -0.877981 -2.094577
H 1.383766 -1.403673 -1.994045
H 0.072720 -0.872817 0.648525
H -0.693585 -3.254940 -1.065617
H -2.314740 -1.132444 0.397794
H -2.170102 -1.290831 -1.342817
H -3.189772 -3.614936 -1.156726
H -3.499896 -3.288950 0.532121
H -4.633773 -1.802642 -1.885466
H -5.511979 -2.866605 -0.803973
H -4.376560 -0.109555 -0.132084
H -6.055484 -0.527623 -0.351276
H -5.923880 -2.046831 1.653535
H -4.265159 -1.547589 1.904659

H -5.791368 -0.232489 3.373787
H -5.009892 0.834910 2.205362
H -6.684187 0.337930 1.960537
H -3.344455 1.978801 -2.281169
H 1.877008 -2.776139 -0.358703
H -0.002067 -3.497641 1.235008

2c-c15, ΔG = 1.5468 kcal/mol, population = 0.82 %
C -1.983541 2.410442 1.762997
C -0.497319 2.617396 2.042309
C 0.368276 1.861217 1.079062
C -0.063074 1.397111 -0.129445
C -1.400714 1.720554 -0.571818
C -2.249930 2.654209 0.288802
C 0.803066 0.520532 -1.021500
N 1.678050 1.679494 1.470327
C 2.598413 1.180380 0.599079
C 2.233613 0.673214 -0.602376
C 4.074717 1.185872 0.848961
C 4.632497 0.404762 -0.350964
C 3.418374 0.295931 -1.321840
O 3.542921 -0.054099 -2.489548
O 5.681039 1.151512 -0.973491
C 5.100002 -1.000637 -0.055341
C 4.860099 -1.718322 1.034814
C 0.330928 -0.956373 -1.001046
C 0.255487 -1.604465 0.374283
C -0.561618 -2.904544 0.375885
C -2.042175 -2.718757 0.054415
C -2.758066 -1.669072 0.906496
C -4.265257 -1.591178 0.654634
C -4.636154 -1.157745 -0.764337
C -6.125909 -0.867632 -0.938178
C -6.484244 -0.426666 -2.356115
O -1.882770 1.295626 -1.624047
O -3.621083 2.489961 -0.025714
O 1.553145 -1.870774 0.945622
O -0.455639 -3.515827 1.668499
H -2.579879 3.091728 2.369748
H -2.277950 1.389616 2.017294
H -0.244918 3.681409 1.968957
H -0.250348 2.309346 3.060296
H -1.932586 3.678351 0.037132
H 0.688818 0.857875 -2.054919

H 1.978298 2.053681 2.358787
H 4.467195 2.204010 0.831140
H 4.329126 0.751544 1.815256
H 5.744017 0.839474 -1.888261
H 5.656001 -1.443703 -0.876915
H 5.226640 -2.733578 1.119229
H 4.287302 -1.340987 1.871977
H -0.658453 -0.981709 -1.455118
H 0.987836 -1.547656 -1.646786
H -0.201968 -0.917742 1.085874
H -0.123591 -3.580545 -0.372670
H -2.139274 -2.476380 -1.005184
H -2.529110 -3.689494 0.188820
H -2.574880 -1.883320 1.961940
H -2.330236 -0.682293 0.711927
H -4.726083 -2.561065 0.873901
H -4.698309 -0.877617 1.363386
H -4.061742 -0.262661 -1.024101
H -4.338479 -1.928692 -1.482064
H -6.702312 -1.760871 -0.674147
H -6.423592 -0.089735 -0.227099
H -7.552271 -0.220660 -2.455153
H -5.941769 0.482082 -2.630549
H -6.223946 -1.199338 -3.084422
H -3.636360 2.080330 -0.907336
H 2.130291 -2.247240 0.268580
H 0.461963 -3.376387 1.945274

2c-c420, ΔG = 1.5663 kcal/mol, population = 0.80 %
C -0.340215 5.505029 0.139173
C 0.958435 4.852838 0.607432
C 0.992879 3.385405 0.301566
C 0.204084 2.791620 -0.641177
C -0.676124 3.601655 -1.442181
C -0.611072 5.117316 -1.304148
C 0.183039 1.294364 -0.877835
N 1.906991 2.642487 1.014369
C 2.114698 1.325777 0.726450
C 1.360559 0.672350 -0.190727
C 3.189135 0.490973 1.354712
C 2.859164 -0.931029 0.846547
C 1.840695 -0.679523 -0.305393
O 1.556585 -1.538985 -1.127744
O 4.018762 -1.567190 0.337627

C 2.149431 -1.742399 1.903955
C 2.457154 -2.988248 2.237772
C -1.137826 0.642192 -0.372331
C -1.756154 -0.296981 -1.398759
C -2.723361 -1.308131 -0.781357
C -1.993513 -2.363623 0.033965
C -2.906685 -3.398608 0.690776
C -2.185973 -4.240807 1.747760
C -0.982697 -5.024594 1.222824
C -0.327638 -5.905171 2.285936
C 0.897406 -6.654540 1.765665
O -1.470448 3.120854 -2.263292
O -1.807218 5.706933 -1.778439
O -2.485855 0.445345 -2.392066
O -3.455228 -1.965006 -1.825481
H -0.270734 6.589068 0.227425
H -1.174832 5.168850 0.758915
H 1.816685 5.323737 0.114625
H 1.097518 4.999240 1.680538
H 0.237657 5.445028 -1.925299
H 0.252262 1.118809 -1.955361
H 2.489908 3.106292 1.696027
H 4.175757 0.788853 0.995594
H 3.185726 0.571270 2.442574
H 3.716984 -2.239754 -0.290792
H 1.309732 -1.239668 2.375489
H 1.888834 -3.521255 2.988784
H 3.288532 -3.509789 1.780677
H -0.923267 0.109624 0.553395
H -1.869517 1.412657 -0.119745
H -0.960550 -0.860585 -1.900030
H -3.430257 -0.765549 -0.136482
H -1.265691 -2.856095 -0.615289
H -1.419507 -1.862157 0.817286
H -3.749620 -2.883882 1.163643
H -3.333027 -4.050871 -0.075972
H -1.852848 -3.582692 2.559188
H -2.900149 -4.940739 2.194199
H -1.296037 -5.648458 0.377125
H -0.230562 -4.332829 0.832434
H -0.039328 -5.280858 3.138581
H -1.063065 -6.621386 2.667351
H 1.659115 -5.956635 1.408626
H 1.349801 -7.273874 2.543280

H 0.630833 -7.308110 0.930780
H -2.204523 5.051696 -2.375404
H -2.132499 1.352976 -2.427912
H -3.706866 -1.267558 -2.446940

2c-c16, ΔG = 1.5700 kcal/mol, population = 0.79 %
C -2.397850 2.242903 0.728200
C -1.033021 2.575122 1.321213
C 0.076781 1.855471 0.614462
C -0.027082 1.346000 -0.647413
C -1.248339 1.562328 -1.391445
C -2.344026 2.430997 -0.775774
C 1.081855 0.529101 -1.300606
N 1.258134 1.757237 1.317407
C 2.376552 1.245524 0.730892
C 2.343849 0.700798 -0.508925
C 3.730015 1.262570 1.369622
C 4.585712 0.426898 0.391299
C 3.672932 0.283211 -0.864829
O 4.095775 -0.118076 -1.941020
O 5.769682 1.130901 0.048690
C 4.848187 -0.953600 0.939410
C 6.049284 -1.502903 1.061306
C 0.694743 -0.965006 -1.421002
C 0.484692 -1.666697 -0.078822
C -0.698917 -2.644631 -0.092669
C -2.032112 -1.915583 -0.152599
C -3.244834 -2.838378 -0.270441
C -4.557952 -2.078934 -0.483742
C -4.961138 -1.176536 0.681940
C -6.282822 -0.445216 0.452186
C -6.655078 0.486473 1.603897
O -1.443347 1.092107 -2.514670
O -3.590484 2.135469 -1.379193
O 1.674500 -2.350166 0.368773
O -0.671259 -3.438948 1.098121
H -3.166552 2.883045 1.160832
H -2.661018 1.206721 0.948443
H -0.843095 3.652247 1.252140
H -1.003783 2.321542 2.382657
H -2.064995 3.474516 -0.991922
H 1.230104 0.896119 -2.319997
H 1.312290 2.157496 2.242603
H 4.123332 2.279046 1.428040

H 3.707274 0.850184 2.379371
H 6.096036 0.746968 -0.778163
H 3.953196 -1.503407 1.215859
H 6.166993 -2.505129 1.453429
H 6.949458 -0.968220 0.784912
H -0.204694 -1.028357 -2.030249
H 1.479662 -1.485303 -1.976071
H 0.289819 -0.938114 0.710522
H -0.597800 -3.303342 -0.968729
H -2.110446 -1.294941 0.743871
H -2.028271 -1.235952 -1.007237
H -3.087403 -3.519942 -1.112792
H -3.320825 -3.461850 0.624553
H -4.476321 -1.472505 -1.393312
H -5.361035 -2.800365 -0.666660
H -5.030509 -1.776192 1.597175
H -4.180711 -0.432829 0.863276
H -6.214106 0.131491 -0.476106
H -7.080886 -1.179385 0.298827
H -7.602142 0.997263 1.416695
H -6.754262 -0.068120 2.540859
H -5.887159 1.250551 1.751150
H -3.372078 1.717413 -2.229402
H 2.048470 -2.835428 -0.377890
H 0.266347 -3.607361 1.274543

2c-c123, ΔG = 1.5863 kcal/mol, population = 0.77 %
C -0.613570 5.340412 0.697530
C 0.811732 4.798897 0.770778
C 0.873823 3.334891 0.454333
C -0.100945 2.667008 -0.229320
C -1.227676 3.397141 -0.747946
C -1.249092 4.914506 -0.614537
C -0.067695 1.168804 -0.467036
N 2.011879 2.677773 0.864524
C 2.238904 1.382966 0.502554
C 1.305941 0.655768 -0.158905
C 3.527578 0.661898 0.752215
C 3.210262 -0.782005 0.302411
C 1.854758 -0.640750 -0.455284
O 1.410980 -1.525188 -1.174613
O 4.206793 -1.257755 -0.588000
C 2.988184 -1.690650 1.485964
C 3.549700 -2.881479 1.642841

C -1.140567 0.417250 0.376503
C -1.931847 -0.583718 -0.451840
C -2.793288 -1.512384 0.408985
C -3.343131 -2.712961 -0.365111
C -2.280178 -3.610622 -1.011239
C -1.183082 -4.087851 -0.057472
C -0.246880 -5.112449 -0.699702
C 0.970924 -5.473056 0.156189
C 0.624386 -6.124026 1.495425
O -2.174420 2.847772 -1.329820
O -2.573955 5.400656 -0.725582
O -2.812096 0.080377 -1.377403
O -3.862264 -0.763796 1.004580
H -0.608277 6.427562 0.773966
H -1.208877 4.946581 1.524611
H 1.454009 5.328892 0.058127
H 1.237947 4.971311 1.760981
H -0.639291 5.302156 -1.446165
H -0.289556 0.990155 -1.523387
H 2.729272 3.195834 1.351115
H 4.328991 1.070338 0.134045
H 3.837272 0.729712 1.795995
H 3.799399 -1.960398 -1.115710
H 2.288379 -1.311620 2.225353
H 3.330315 -3.492754 2.508689
H 4.250470 -3.277740 0.919016
H -0.641429 -0.109959 1.191736
H -1.827543 1.128212 0.839168
H -1.222117 -1.191482 -1.018221
H -2.185236 -1.869596 1.241133
H -3.942481 -3.302237 0.335063
H -4.026989 -2.344049 -1.134920
H -2.784295 -4.482776 -1.439466
H -1.815563 -3.090504 -1.854541
H -0.583690 -3.235338 0.276324
H -1.645386 -4.512600 0.839048
H -0.812428 -6.022158 -0.932995
H 0.103235 -4.714244 -1.658013
H 1.614068 -6.150026 -0.414594
H 1.560599 -4.569008 0.336392
H 0.013166 -7.019268 1.349730
H 1.527659 -6.420328 2.033319
H 0.066131 -5.443555 2.141378
H -3.067671 4.713799 -1.202878

H -2.582053 1.027814 -1.410746
H -4.213626 -0.216538 0.285993

2c-c9, ΔG = 1.5958 kcal/mol, population = 0.76 %
C -2.155204 2.546071 1.632969
C -0.676936 2.681800 1.983876
C 0.198575 1.882219 1.065677
C -0.200892 1.405113 -0.148722
C -1.501488 1.781856 -0.655752
C -2.331098 2.784568 0.143741
C 0.675388 0.496313 -0.999255
N 1.489450 1.680776 1.505625
C 2.428975 1.152937 0.672229
C 2.093954 0.620198 -0.527155
C 3.894299 1.146227 0.973166
C 4.488581 0.337571 -0.200789
C 3.291462 0.191057 -1.192691
O 3.438288 -0.216364 -2.338274
O 5.530180 1.072952 -0.827736
C 4.917832 -1.036366 0.245478
C 6.100776 -1.581083 -0.004934
C 0.162031 -0.966837 -0.995363
C 0.142143 -1.647494 0.366196
C -0.724421 -2.912770 0.404926
C -2.212567 -2.697693 0.150662
C -2.865591 -1.602763 0.998117
C -4.391307 -1.728699 1.079425
C -5.120564 -1.766433 -0.267319
C -4.930475 -0.513808 -1.121063
C -5.701538 -0.570474 -2.438041
O -1.959571 1.357425 -1.718581
O -3.690273 2.711095 -0.242540
O 1.467960 -1.998990 0.823097
O -0.584758 -3.526500 1.694121
H -2.745931 3.265061 2.200402
H -2.514044 1.544756 1.881960
H -0.369946 3.732039 1.920554
H -0.493111 2.366894 3.013231
H -1.930730 3.780380 -0.102675
H 0.610151 0.831783 -2.037794
H 1.768569 2.073557 2.392885
H 4.301332 2.158946 0.968029
H 4.104982 0.703747 1.948004
H 5.623663 0.720564 -1.724769

H 4.163032 -1.585561 0.800290
H 6.340395 -2.579026 0.339230
H 6.868660 -1.043727 -0.547135
H -0.846899 -0.953671 -1.405488
H 0.773318 -1.557387 -1.684421
H -0.224346 -0.958170 1.127464
H -0.339354 -3.603722 -0.359450
H -2.364651 -2.497803 -0.911884
H -2.703136 -3.655187 0.350025
H -2.461198 -1.643466 2.012241
H -2.597507 -0.621166 0.600282
H -4.633236 -2.641434 1.634171
H -4.781343 -0.894945 1.672672
H -4.797575 -2.642902 -0.838843
H -6.190254 -1.909094 -0.080066
H -5.247991 0.361416 -0.545264
H -3.869272 -0.365496 -1.333566
H -6.775638 -0.675350 -2.262663
H -5.546670 0.334204 -3.030307
H -5.380544 -1.423103 -3.042703
H -3.692993 2.239814 -1.093041
H 1.894585 -2.519562 0.129451
H 0.347054 -3.421676 1.935120

2c-c46, ΔG = 1.6077 kcal/mol, population = 0.74 %
C -1.939151 2.739933 1.486222
C -0.451117 2.930937 1.763370
C 0.402535 2.019987 0.931779
C -0.030183 1.388481 -0.196945
C -1.357186 1.672115 -0.694293
C -2.170846 2.770547 -0.013904
C 0.823490 0.377176 -0.948704
N 1.705536 1.880851 1.360277
C 2.616393 1.213841 0.604367
C 2.248830 0.519665 -0.500871
C 4.087357 1.221962 0.877885
C 4.664227 0.322719 -0.237985
C 3.427877 0.010410 -1.142617
O 3.537454 -0.545889 -2.228536
O 5.610750 1.059950 -1.004039
C 5.225640 -0.962004 0.311132
C 6.434854 -1.438118 0.046230
C 0.293245 -1.076072 -0.810829
C -0.133035 -1.488344 0.588751

C -0.659539 -2.926638 0.655844
C -1.890783 -3.207038 -0.198059
C -3.076462 -2.270224 0.033360
C -4.305426 -2.684006 -0.777046
C -5.551141 -1.836842 -0.504902
C -5.399911 -0.359788 -0.870623
C -6.690111 0.435455 -0.682753
O -1.854909 1.085599 -1.658067
O -3.541996 2.630720 -0.335718
O 0.921096 -1.342085 1.563389
O -0.979083 -3.241289 2.018588
H -2.518148 3.525315 1.972015
H -2.278087 1.778829 1.880546
H -0.157923 3.965299 1.550128
H -0.230264 2.757975 2.818853
H -1.795277 3.726544 -0.410690
H 0.768405 0.608279 -2.016351
H 2.004317 2.379483 2.185701
H 4.499594 2.228811 0.793296
H 4.309735 0.851875 1.880118
H 5.691434 0.613197 -1.859169
H 4.552703 -1.515251 0.959910
H 6.770948 -2.376067 0.469227
H 7.128863 -0.901189 -0.588010
H -0.568031 -1.178689 -1.468845
H 1.062720 -1.760228 -1.181069
H -0.912086 -0.817780 0.950688
H 0.151031 -3.590597 0.320741
H -1.598913 -3.181443 -1.251155
H -2.195417 -4.237727 0.006896
H -3.328809 -2.259099 1.097499
H -2.796198 -1.249104 -0.232759
H -4.063823 -2.637055 -1.845449
H -4.535130 -3.732809 -0.561222
H -6.393983 -2.255591 -1.065318
H -5.818065 -1.917733 0.555441
H -4.611654 0.090766 -0.263948
H -5.067600 -0.282379 -1.911892
H -6.551642 1.487553 -0.940960
H -7.492533 0.038905 -1.310551
H -7.029403 0.390004 0.355675
H -3.566943 2.043481 -1.110705
H 1.709474 -1.786600 1.226427
H -0.272590 -2.848450 2.551038

2c-c260, ΔG = 1.6121 kcal/mol, population = 0.74 %
C -0.292865 5.500707 0.094132
C 1.044562 4.864789 0.462734
C 1.038036 3.379800 0.257321
C 0.155424 2.730941 -0.557812
C -0.801530 3.498689 -1.311435
C -0.716649 5.019692 -1.282665
C 0.110018 1.218299 -0.691998
N 2.018227 2.678865 0.921916
C 2.201027 1.347566 0.693549
C 1.355729 0.638163 -0.093572
C 3.351067 0.559811 1.240970
C 2.990982 -0.890431 0.847653
C 1.844683 -0.711818 -0.192595
O 1.487637 -1.615509 -0.935871
O 4.096696 -1.537978 0.242158
C 2.427387 -1.649050 2.024607
C 2.825946 -2.852877 2.410887
C -1.157574 0.616221 -0.014762
C -1.888052 -0.381097 -0.899008
C -3.024515 -1.096153 -0.163206
C -2.573257 -2.025745 0.958789
C -1.542681 -3.083738 0.564149
C -1.246620 -4.059602 1.702453
C -0.139375 -5.069568 1.381875
C -0.399604 -5.961399 0.162363
C -1.671969 -6.802020 0.266607
O -1.683630 2.977977 -2.009544
O -1.953413 5.589849 -1.668046
O -2.461830 0.261808 -2.053822
O -3.782168 -1.865888 -1.108967
H -0.208697 6.587277 0.100334
H -1.058132 5.217076 0.820489
H 1.846135 5.288644 -0.153106
H 1.301653 5.084026 1.500742
H 0.063223 5.295416 -2.010229
H 0.068611 0.973367 -1.756865
H 2.669137 3.182163 1.507389
H 4.286778 0.848883 0.759101
H 3.466060 0.699299 2.316722
H 3.737213 -2.247745 -0.310542
H 1.621397 -1.142474 2.547799
H 2.365000 -3.348976 3.255042

H 3.627207 -3.374476 1.902984
H -0.858369 0.133836 0.915998
H -1.849045 1.416337 0.261471
H -1.179477 -1.127142 -1.267526
H -3.678108 -0.322728 0.264138
H -2.183617 -1.419268 1.780649
H -3.469389 -2.519281 1.347881
H -1.909819 -3.626793 -0.309195
H -0.606951 -2.607869 0.261245
H -0.949015 -3.490076 2.589598
H -2.165770 -4.586965 1.977014
H 0.796310 -4.524049 1.227648
H 0.018032 -5.708401 2.257934
H -0.442554 -5.347158 -0.741576
H 0.459906 -6.626124 0.032545
H -1.771703 -7.473262 -0.589153
H -1.663325 -7.414281 1.172729
H -2.566337 -6.176463 0.299434
H -2.415391 4.900395 -2.172872
H -2.237548 1.211103 -2.042360
H -3.888929 -1.291208 -1.880445

2c-c164, ΔG = 1.6158 kcal/mol, population = 0.73 %
C -0.640601 5.448766 0.662834
C 0.788567 4.935444 0.821864
C 0.891602 3.465027 0.546779
C -0.036328 2.762771 -0.166445
C -1.149734 3.457222 -0.757128
C -1.203927 4.976680 -0.666091
C 0.033017 1.260223 -0.359791
N 2.019188 2.838580 1.028480
C 2.288991 1.541478 0.705190
C 1.401027 0.781759 0.018812
C 3.580715 0.853673 1.023996
C 3.323489 -0.600978 0.568422
C 1.989614 -0.505762 -0.234183
O 1.589782 -1.412826 -0.951638
O 4.363970 -1.040815 -0.290468
C 3.098759 -1.515477 1.746472
C 3.715747 -2.674012 1.935288
C -1.065415 0.517846 0.457994
C -1.790417 -0.531179 -0.370656
C -2.724713 -1.407714 0.471541
C -3.173325 -2.678747 -0.253904

C -2.042530 -3.619415 -0.690163
C -1.105406 -4.052010 0.440162
C -0.001502 -5.018318 0.000755
C -0.496475 -6.391430 -0.455941
C 0.647294 -7.342621 -0.804756
O -2.057709 2.873802 -1.367016
O -2.530283 5.434920 -0.852910
O -2.586236 0.075026 -1.406347
O -3.860636 -0.637432 0.887691
H -0.657338 6.537482 0.709604
H -1.269527 5.066481 1.470197
H 1.457407 5.459131 0.129196
H 1.160775 5.140627 1.827669
H -0.561912 5.353819 -1.478061
H -0.139168 1.047052 -1.418769
H 2.702251 3.382447 1.535596
H 4.403275 1.279062 0.446361
H 3.834081 0.934253 2.082046
H 4.004217 -1.767887 -0.819517
H 2.351787 -1.168606 2.454997
H 3.495390 -3.289962 2.797625
H 4.466012 -3.035525 1.243477
H -0.599203 0.033790 1.318428
H -1.790980 1.230440 0.855071
H -1.038736 -1.168667 -0.841916
H -2.204645 -1.680500 1.390356
H -3.851369 -3.212897 0.418246
H -3.761478 -2.387786 -1.129358
H -2.500239 -4.501239 -1.144682
H -1.449709 -3.150276 -1.481418
H -0.628509 -3.170647 0.876796
H -1.693874 -4.511276 1.243291
H 0.579757 -4.555873 -0.805326
H 0.695169 -5.158206 0.834192
H -1.110745 -6.830984 0.337598
H -1.150762 -6.282506 -1.325085
H 0.274357 -8.317167 -1.127358
H 1.260630 -6.935759 -1.613194
H 1.300566 -7.502806 0.057110
H -2.988356 4.726871 -1.334766
H -2.397157 1.031683 -1.434950
H -4.134405 -0.151148 0.094999

2c-c184, ΔG = 1.6196 kcal/mol, population = 0.73 %

C -3.067423 2.513746 -0.738406
C -2.024197 2.725485 0.352046
C -0.751008 1.977771 0.083795
C -0.376395 1.507568 -1.143640
C -1.244742 1.769677 -2.269466
C -2.417900 2.729544 -2.093392
C 0.917869 0.723384 -1.379251
N 0.049167 1.795534 1.188761
C 1.257923 1.178875 1.082961
C 1.697595 0.672031 -0.093847
C 2.217145 1.038031 2.222771
C 3.305662 0.114581 1.636921
C 3.016570 0.125571 0.114180
O 3.827573 -0.265191 -0.715502
O 4.593608 0.673530 1.901672
C 3.209314 -1.273699 2.220664
C 2.839131 -2.375992 1.581777
C 0.769763 -0.697283 -1.985917
C -0.122708 -1.690418 -1.240376
C -1.626487 -1.468099 -1.460997
C -2.512810 -2.445180 -0.694281
C -2.244756 -2.485083 0.813015
C -2.386434 -1.128865 1.508074
C -2.015437 -1.137291 2.992532
C -0.559167 -1.528308 3.255172
C -0.110002 -1.251957 4.687223
O -1.086812 1.249745 -3.380815
O -3.347308 2.560403 -3.145260
O 0.224604 -3.030108 -1.623129
O -1.940889 -1.610429 -2.858316
H -3.898888 3.205816 -0.606803
H -3.467686 1.498399 -0.691020
H -1.778563 3.790267 0.435439
H -2.416970 2.420097 1.323901
H -1.996155 3.746007 -2.128236
H 1.503158 1.280292 -2.119973
H -0.261967 2.150336 2.081164
H 2.652842 2.005491 2.480772
H 1.741596 0.632283 3.116505
H 5.222588 0.202195 1.336471
H 3.470483 -1.315453 3.274526
H 2.784943 -3.325114 2.099700
H 2.573620 -2.381333 0.532203
H 0.447387 -0.611971 -3.021519

H 1.771669 -1.126053 -2.005788
H 0.090778 -1.631921 -0.172495
H -1.871970 -0.456739 -1.143836
H -3.551459 -2.158240 -0.881274
H -2.375800 -3.443338 -1.115941
H -2.938141 -3.194905 1.274130
H -1.244837 -2.888179 0.985625
H -1.749448 -0.393810 1.011023
H -3.413099 -0.769147 1.385114
H -2.190638 -0.135844 3.399904
H -2.680168 -1.813310 3.541763
H -0.410047 -2.586767 3.028812
H 0.086137 -0.985068 2.559525
H 0.929418 -1.549373 4.842298
H -0.194022 -0.188296 4.926399
H -0.724783 -1.800914 5.405314
H -2.863802 2.088355 -3.844765
H -0.144226 -3.163568 -2.507509
H -1.688359 -0.786940 -3.301152

2c-c105, ΔG = 1.7351 kcal/mol, population = 0.60 %
C -0.270840 5.639930 0.139374
C 0.873071 4.866041 0.787592
C 0.862274 3.418242 0.398068
C 0.206404 2.923441 -0.687809
C -0.455359 3.842651 -1.590838
C -0.293582 5.341947 -1.348924
C 0.110714 1.432785 -0.968190
N 1.619880 2.580817 1.200242
C 1.874945 1.304721 0.816343
C 1.224913 0.737682 -0.236119
C 2.922978 0.437551 1.440472
C 2.758536 -0.904316 0.691997
C 1.787692 -0.555985 -0.472140
O 1.595276 -1.331141 -1.411033
O 4.007409 -1.324786 0.166211
C 2.096690 -1.945008 1.562111
C 2.565437 -3.166560 1.775160
C -1.279767 0.895284 -0.551425
C -1.418654 -0.617030 -0.681356
C -2.845632 -1.111197 -0.359091
C -2.855600 -2.478855 0.327290
C -2.203479 -3.620499 -0.453639
C -2.173044 -4.944236 0.317332

C -1.220015 -4.942660 1.513737
C -1.142118 -6.290775 2.229133
C -0.175540 -6.283117 3.412221
O -1.118228 3.472618 -2.560417
O -1.326108 6.053145 -2.005992
O -1.099022 -1.055734 -2.008596
O -3.650870 -1.090235 -1.543186
H -0.145555 6.709765 0.305953
H -1.225773 5.339532 0.577402
H 1.836107 5.296243 0.489179
H 0.822790 4.943452 1.875903
H 0.681528 5.618740 -1.780196
H 0.218354 1.269625 -2.042434
H 2.130181 2.981435 1.974133
H 3.920753 0.840423 1.258688
H 2.786097 0.347320 2.518803
H 3.820548 -1.961340 -0.538975
H 1.160607 -1.630361 2.014824
H 2.031115 -3.865380 2.405121
H 3.498193 -3.501799 1.339560
H -1.464861 1.167969 0.491421
H -2.039220 1.385058 -1.163316
H -0.738957 -1.098696 0.026285
H -3.308052 -0.395274 0.324410
H -2.357177 -2.353324 1.293377
H -3.895793 -2.740209 0.547283
H -2.743685 -3.761845 -1.393784
H -1.180198 -3.349685 -0.726481
H -3.184786 -5.195532 0.656464
H -1.871622 -5.744711 -0.366347
H -0.219272 -4.656702 1.170607
H -1.523360 -4.178289 2.235796
H -2.142463 -6.572122 2.575080
H -0.838237 -7.061302 1.512712
H -0.149566 -7.251799 3.915985
H 0.841427 -6.051260 3.085179
H -0.465479 -5.530294 4.150037
H -1.664705 5.445452 -2.685153
H -0.137442 -1.216647 -2.040534
H -3.058610 -1.346900 -2.265722

2c-c17, ΔG = 1.7501 kcal/mol, population = 0.58 %
C -2.055260 2.512467 1.924233
C -0.562200 2.718228 2.165216

C 0.275305 1.964820 1.175253
C -0.185837 1.523677 -0.030544
C -1.529585 1.867367 -0.437454
C -2.363372 2.776740 0.462288
C 0.646058 0.654203 -0.963301
N 1.588232 1.757243 1.541517
C 2.475778 1.208272 0.664781
C 2.081213 0.718501 -0.535421
C 3.947713 1.119759 0.920417
C 4.458178 0.279540 -0.271926
C 3.237585 0.237107 -1.241877
O 3.331148 -0.148433 -2.399486
O 5.550846 0.933214 -0.899450
C 4.767597 -1.134845 0.149686
C 5.923859 -1.750509 -0.058376
C 0.128052 -0.804294 -0.980600
C 0.343515 -1.566376 0.329067
C -0.879754 -2.408785 0.719155
C -2.003193 -1.535076 1.261267
C -3.353765 -2.245354 1.390556
C -4.065483 -2.496347 0.056218
C -4.480284 -1.216971 -0.671865
C -5.250983 -1.473795 -1.965641
C -5.641167 -0.183275 -2.684793
O -2.033787 1.471424 -1.491091
O -3.740378 2.599785 0.181232
O 1.520931 -2.399282 0.283941
O -0.520218 -3.349778 1.736137
H -2.636084 3.182751 2.557719
H -2.338402 1.486771 2.170537
H -0.309825 3.782240 2.090893
H -0.288897 2.404383 3.174601
H -2.064948 3.808660 0.218961
H 0.541282 1.037619 -1.981604
H 1.912188 2.114111 2.428610
H 4.409413 2.108607 0.899253
H 4.164902 0.665992 1.888360
H 5.629113 0.559970 -1.789411
H 3.938952 -1.650153 0.626610
H 6.075608 -2.774253 0.259529
H 6.755116 -1.251928 -0.541231
H -0.929589 -0.782483 -1.239075
H 0.627813 -1.343996 -1.789232
H 0.543392 -0.877856 1.152128

H -1.215180 -2.956626 -0.172592
H -1.677565 -1.168647 2.238746
H -2.116714 -0.656753 0.625970
H -3.209026 -3.193426 1.914894
H -4.007306 -1.638029 2.024546
H -3.427822 -3.095847 -0.602347
H -4.957473 -3.102619 0.244393
H -5.095027 -0.605569 -0.000709
H -3.599574 -0.613700 -0.907950
H -4.639452 -2.092967 -2.630696
H -6.150234 -2.058199 -1.743432
H -6.194242 -0.386090 -3.604531
H -6.270627 0.443672 -2.047493
H -4.754704 0.399300 -2.948063
H -3.773768 2.195723 -0.702734
H 1.553380 -2.841333 -0.574131
H 0.382489 -3.629333 1.522514

2c-c445, ΔG = 1.7796 kcal/mol, population = 0.56 %
C -0.262429 5.417392 0.036203
C 1.040547 4.819031 0.560065
C 1.129246 3.345217 0.298462
C 0.388213 2.697545 -0.647603
C -0.496177 3.457024 -1.492996
C -0.477864 4.977667 -1.401205
C 0.423026 1.191548 -0.838171
N 2.046401 2.655159 1.059001
C 2.309802 1.340401 0.813234
C 1.601973 0.632500 -0.100810
C 3.406136 0.570689 1.483133
C 3.162633 -0.874085 0.991769
C 2.145774 -0.697259 -0.176223
O 1.911210 -1.587881 -0.981585
O 4.365171 -1.445127 0.504389
C 2.495412 -1.709210 2.057088
C 2.907478 -2.906383 2.450334
C -0.896910 0.524133 -0.351299
C -1.460939 -0.477508 -1.344636
C -2.687940 -1.212442 -0.801398
C -2.426139 -2.075848 0.428776
C -1.286905 -3.089273 0.297558
C -1.021671 -3.870704 1.589721
C -2.203516 -4.682386 2.129681
C -2.739706 -5.736877 1.161515

C -3.852994 -6.586806 1.771372
O -1.258799 2.930922 -2.316240
O -1.674412 5.517736 -1.930060
O -1.845142 0.158371 -2.579475
O -3.227694 -2.048390 -1.836470
H -0.227582 6.505229 0.092523
H -1.104807 5.074725 0.641727
H 1.898071 5.302421 0.077991
H 1.143687 5.000609 1.631645
H 0.380304 5.310173 -2.006684
H 0.529164 0.985378 -1.906521
H 2.595882 3.159395 1.739951
H 4.386365 0.912652 1.146297
H 3.368042 0.668176 2.569030
H 4.112811 -2.158874 -0.099744
H 1.601000 -1.267176 2.487056
H 2.369408 -3.459933 3.208935
H 3.796543 -3.364259 2.035460
H -0.705394 0.027226 0.599808
H -1.650350 1.290524 -0.152276
H -0.693908 -1.213448 -1.595438
H -3.435725 -0.451190 -0.538833
H -2.229997 -1.416381 1.279191
H -3.360629 -2.592958 0.659505
H -1.507768 -3.778057 -0.521016
H -0.362026 -2.578306 0.022985
H -0.177286 -4.546589 1.418763
H -0.694188 -3.167333 2.362876
H -1.888965 -5.179289 3.053783
H -3.019848 -4.010724 2.414943
H -3.113844 -5.251575 0.255722
H -1.915034 -6.384141 0.843108
H -3.500248 -7.117804 2.659486
H -4.699462 -5.963853 2.073068
H -4.223391 -7.330573 1.062434
H -2.036152 4.831895 -2.515272
H -1.712366 1.121208 -2.501088
H -3.230645 -1.501380 -2.634655

2c-c21, ΔG = 1.8003 kcal/mol, population = 0.54 %
C -2.507201 2.338746 0.587990
C -1.180403 2.503835 1.321235
C -0.057795 1.779807 0.638878
C -0.089422 1.370732 -0.662258

C -1.236143 1.712225 -1.475816
C -2.306223 2.628625 -0.886764
C 1.041842 0.579432 -1.308298
N 1.067756 1.580633 1.409270
C 2.214592 1.100021 0.851714
C 2.253232 0.658519 -0.427919
C 3.525496 1.047375 1.571233
C 4.449660 0.323255 0.566606
C 3.597594 0.257986 -0.739164
O 4.074400 -0.073024 -1.816988
O 5.614940 1.102216 0.330724
C 4.753487 -1.083507 1.013258
C 5.972086 -1.592628 1.136254
C 0.630911 -0.882841 -1.602008
C 0.421624 -1.753267 -0.366486
C -0.607007 -2.870368 -0.602837
C -2.046892 -2.379552 -0.729675
C -2.631305 -1.735678 0.527585
C -4.057078 -1.230360 0.312442
C -4.704291 -0.708722 1.596297
C -6.042884 0.002877 1.380935
C -7.138689 -0.895044 0.805815
O -1.376594 1.325953 -2.638198
O -3.515136 2.489036 -1.610673
O 1.658595 -2.344256 0.089980
O -0.546171 -3.814330 0.471504
H -3.257011 3.010612 1.005394
H -2.874617 1.318885 0.699557
H -0.911239 3.564561 1.381988
H -1.262564 2.146578 2.349970
H -1.923261 3.655155 -0.999033
H 1.263448 1.033625 -2.278479
H 1.071411 1.908168 2.364145
H 3.907428 2.050810 1.767516
H 3.440937 0.526144 2.526039
H 5.992156 0.798483 -0.507635
H 3.874306 -1.688023 1.214247
H 6.119391 -2.617267 1.453164
H 6.857455 -1.002139 0.936101
H -0.277418 -0.852731 -2.200409
H 1.397669 -1.344503 -2.230779
H 0.094146 -1.149758 0.480524
H -0.330192 -3.377433 -1.539057
H -2.108291 -1.677851 -1.565239

H -2.665076 -3.237431 -1.009502
H -2.617162 -2.461199 1.344147
H -2.005849 -0.898778 0.850976
H -4.050183 -0.434205 -0.441263
H -4.664368 -2.038625 -0.105362
H -4.843113 -1.543865 2.292275
H -4.013316 -0.017017 2.089513
H -6.381620 0.413916 2.336714
H -5.887055 0.859472 0.716468
H -8.088082 -0.360182 0.732919
H -6.884377 -1.251962 -0.194021
H -7.296820 -1.771592 1.440405
H -3.259951 2.090992 -2.460435
H 2.121228 -2.712575 -0.674256
H 0.388744 -3.865393 0.719887

2c-c26, ΔG = 1.8731 kcal/mol, population = 0.47 %
C -2.419793 2.449384 0.899247
C -1.026431 2.699050 1.464478
C 0.029869 1.920611 0.737839
C -0.134174 1.381149 -0.504939
C -1.366429 1.638297 -1.218920
C -2.375578 2.608909 -0.609392
C 0.939662 0.543567 -1.191398
N 1.234075 1.812545 1.399080
C 2.320929 1.275099 0.780305
C 2.230167 0.694048 -0.439826
C 3.699823 1.300723 1.360936
C 4.505065 0.423671 0.377714
C 3.537474 0.248695 -0.834173
O 3.913653 -0.195878 -1.911698
O 5.680108 1.098063 -0.046325
C 4.780330 -0.935599 0.969711
C 5.970391 -1.519648 1.008517
C 0.515846 -0.934793 -1.368528
C 0.221432 -1.699971 -0.087270
C -0.547792 -3.004898 -0.342867
C -1.964180 -2.834565 -0.882118
C -2.931769 -2.076449 0.026700
C -4.330027 -1.972955 -0.587522
C -5.416767 -1.518904 0.390517
C -5.163725 -0.151393 1.023794
C -6.331498 0.333140 1.880596
O -1.628035 1.140786 -2.316424

O -3.648364 2.418136 -1.197818
O 1.427923 -2.022628 0.641785
O -0.622871 -3.755180 0.875702
H -3.136062 3.148302 1.331133
H -2.749439 1.439038 1.143499
H -0.773803 3.763226 1.391416
H -0.989992 2.444012 2.525663
H -2.007477 3.619679 -0.846420
H 1.071332 0.930752 -2.206834
H 1.333930 2.246737 2.305026
H 4.103199 2.315020 1.366200
H 3.717020 0.924419 2.384907
H 5.940479 0.703622 -0.891596
H 3.904278 -1.436837 1.370549
H 6.095544 -2.503240 1.442957
H 6.855146 -1.030323 0.620954
H -0.368914 -0.936671 -2.001212
H 1.300318 -1.458132 -1.923589
H -0.349801 -1.081931 0.606680
H 0.031082 -3.582059 -1.079578
H -1.915426 -2.349877 -1.860391
H -2.362194 -3.837321 -1.064050
H -2.991258 -2.587058 0.991496
H -2.549414 -1.073390 0.230283
H -4.296898 -1.285471 -1.440338
H -4.611170 -2.950609 -0.993235
H -6.377334 -1.494209 -0.135683
H -5.521651 -2.268652 1.183670
H -4.263085 -0.199560 1.642566
H -4.953959 0.579304 0.237294
H -6.118683 1.304545 2.332729
H -7.240742 0.435790 1.282141
H -6.545881 -0.371246 2.689047
H -3.478484 1.931447 -2.022540
H 2.046384 -2.439633 0.027110
H 0.231939 -3.630921 1.313223

2c-c7, ΔG = 1.8850 kcal/mol, population = 0.47 %
C -2.398923 2.378911 1.102664
C -0.988545 2.518749 1.668546
C 0.030902 1.764792 0.866553
C -0.177514 1.302375 -0.399936
C -1.410569 1.645215 -1.074759
C -2.359895 2.629138 -0.394093

C 0.850665 0.467072 -1.152842
N 1.254531 1.597433 1.478658
C 2.320094 1.119891 0.774978
C 2.180093 0.620705 -0.475980
C 3.725365 1.136676 1.288561
C 4.513021 0.398399 0.183592
C 3.474662 0.243830 -0.971754
O 3.793783 -0.126735 -2.094005
O 5.595467 1.204994 -0.261889
C 4.938969 -0.974150 0.635988
C 6.178906 -1.441516 0.576427
C 0.415913 -1.014202 -1.280776
C 0.433097 -1.809231 0.020315
C -0.505173 -3.024124 0.000918
C -1.989370 -2.686630 -0.097696
C -2.496223 -1.709953 0.963827
C -4.016329 -1.534764 0.961321
C -4.577856 -0.969743 -0.343761
C -6.061746 -0.616025 -0.259236
C -6.612250 -0.047197 -1.565768
O -1.711524 1.216172 -2.190211
O -3.648706 2.546736 -0.972662
O 1.762555 -2.266469 0.355753
O -0.301252 -3.789133 1.196219
H -3.069552 3.090446 1.584058
H -2.786945 1.376188 1.290608
H -0.692705 3.574076 1.683271
H -0.952408 2.172995 2.704012
H -1.939906 3.631258 -0.573087
H 0.914428 0.851507 -2.174543
H 1.391067 1.967900 2.407827
H 4.096461 2.158272 1.388472
H 3.804108 0.652794 2.263340
H 5.854758 0.872974 -1.133559
H 4.128350 -1.591584 1.010614
H 6.413877 -2.444092 0.910578
H 6.997032 -0.837338 0.204495
H -0.584789 -1.020815 -1.709325
H 1.066326 -1.513053 -2.005322
H 0.155521 -1.173776 0.861884
H -0.231978 -3.642155 -0.867053
H -2.193932 -2.299673 -1.097130
H -2.540492 -3.628726 -0.018630
H -2.172130 -2.055353 1.948134

H -2.031992 -0.732065 0.810992
H -4.495037 -2.496436 1.178288
H -4.291644 -0.864361 1.782657
H -4.016083 -0.072551 -0.619927
H -4.429882 -1.687526 -1.156598
H -6.631624 -1.507815 0.023270
H -6.210399 0.110514 0.546739
H -6.497288 -0.762983 -2.384124
H -7.673728 0.196282 -1.482113
H -6.080891 0.865564 -1.846491
H -3.519155 2.111179 -1.832386
H 2.140009 -2.696524 -0.423205
H 0.651811 -3.762181 1.365067

2c-c404, ΔG = 1.8894 kcal/mol, population = 0.46 %
C -1.576008 3.809397 1.834993
C -0.186427 4.006821 1.235829
C 0.436583 2.711127 0.806827
C -0.260762 1.559965 0.597105
C -1.705220 1.594385 0.688030
C -2.394352 2.939722 0.898669
C 0.407361 0.236484 0.248750
N 1.801371 2.758887 0.587963
C 2.456670 1.694285 0.062727
C 1.832236 0.503995 -0.138161
C 3.888323 1.723767 -0.370098
C 4.165904 0.260135 -0.782109
C 2.766636 -0.418619 -0.703244
O 2.578223 -1.572526 -1.091326
O 4.631639 0.224022 -2.124739
C 5.095154 -0.422273 0.188654
C 6.215124 -1.048999 -0.144237
C 0.360097 -0.804148 1.406798
C -0.679724 -1.921391 1.286356
C -0.550537 -2.746880 -0.001851
C -1.631518 -3.813607 -0.143523
C -3.069225 -3.286562 -0.144068
C -3.375687 -2.277063 -1.251417
C -4.843388 -1.850723 -1.270988
C -5.208461 -0.894808 -2.410833
C -4.513362 0.465724 -2.336597
O -2.409363 0.592211 0.565186
O -3.710032 2.737065 1.379614
O -0.563039 -2.787232 2.426850

O 0.722483 -3.414937 -0.018510
H -2.065068 4.772639 1.979917
H -1.502375 3.317716 2.808061
H -0.245001 4.664319 0.360772
H 0.474960 4.500967 1.950594
H -2.432250 3.423791 -0.090055
H -0.119658 -0.180347 -0.611888
H 2.289511 3.631209 0.731060
H 4.023685 2.382317 -1.229757
H 4.545133 2.073476 0.427528
H 4.545682 -0.688724 -2.434840
H 4.775020 -0.383465 1.226068
H 6.831766 -1.526146 0.606510
H 6.557573 -1.094908 -1.170420
H 1.343497 -1.273202 1.490921
H 0.191559 -0.282176 2.350332
H -1.674406 -1.487221 1.331849
H -0.609638 -2.073878 -0.859553
H -1.439221 -4.347396 -1.078748
H -1.507951 -4.537207 0.665853
H -3.743999 -4.142487 -0.246917
H -3.301688 -2.838147 0.826632
H -2.749546 -1.392548 -1.124509
H -3.113161 -2.715305 -2.221995
H -5.471825 -2.745022 -1.344023
H -5.091113 -1.378796 -0.313025
H -4.971909 -1.373244 -3.367734
H -6.291978 -0.741026 -2.406926
H -4.855789 1.124833 -3.137579
H -4.719961 0.958388 -1.383186
H -3.429665 0.372430 -2.423649
H -3.924407 1.815551 1.153187
H 0.210901 -3.343744 2.257705
H 1.380944 -2.796131 -0.385601

2c-c102, ΔG = 1.8907 kcal/mol, population = 0.46 %
C -0.276939 5.236833 1.098411
C 1.145232 4.711385 1.270351
C 1.288170 3.307586 0.763650
C 0.421385 2.714129 -0.102619
C -0.662866 3.497090 -0.658840
C -0.727055 4.987412 -0.329553
C 0.532031 1.247161 -0.486167
N 2.415432 2.631624 1.199271

C 2.773095 1.452192 0.631194
C 1.934890 0.786241 -0.208176
C 4.118991 0.820073 0.797252
C 3.989859 -0.497465 -0.000027
C 2.628133 -0.346984 -0.737943
O 2.284628 -1.118791 -1.635008
O 5.039340 -0.591930 -0.951382
C 3.907666 -1.692140 0.917414
C 4.668804 -2.773661 0.824317
C -0.523837 0.415289 0.277507
C -0.472826 -1.085061 -0.006164
C -1.672933 -1.820300 0.602090
C -1.482460 -3.338449 0.592114
C -2.410540 -4.118571 1.529922
C -3.908992 -4.071012 1.211897
C -4.281319 -4.652192 -0.151004
C -5.788068 -4.663677 -0.406772
C -6.159831 -5.240423 -1.771697
O -1.518595 3.025389 -1.408389
O -2.035645 5.477395 -0.556250
O -0.488011 -1.363032 -1.412959
O -2.874359 -1.418966 -0.075068
H -0.316752 6.302378 1.324234
H -0.955176 4.720394 1.781984
H 1.850615 5.348646 0.724581
H 1.445292 4.744255 2.319896
H -0.020520 5.483337 -1.014034
H 0.317547 1.148165 -1.552392
H 3.063928 3.112967 1.805557
H 4.898505 1.442928 0.355208
H 4.368380 0.662980 1.847528
H 4.763967 -1.237740 -1.617992
H 3.144954 -1.620864 1.687678
H 4.550577 -3.600264 1.513003
H 5.439539 -2.861749 0.069084
H -0.382841 0.566608 1.351717
H -1.510713 0.796004 0.014443
H 0.434539 -1.507490 0.436242
H -1.794683 -1.480734 1.634046
H -1.575544 -3.695686 -0.436133
H -0.450901 -3.546195 0.892802
H -2.088098 -5.165547 1.530960
H -2.254869 -3.753989 2.551275
H -4.440600 -4.630562 1.989579

H -4.264453 -3.040005 1.267932
H -3.793851 -4.077252 -0.943494
H -3.893633 -5.675244 -0.228956
H -6.283265 -5.240127 0.381984
H -6.171742 -3.641058 -0.324942
H -7.240702 -5.235269 -1.929115
H -5.702343 -4.661969 -2.578770
H -5.813419 -6.272930 -1.868018
H -2.450774 4.833409 -1.154763
H 0.431111 -1.296039 -1.730622
H -2.699368 -1.532057 -1.019297

2c-c151, ΔG = 2.0030 kcal/mol, population = 0.38 %
C -1.783241 3.565109 0.804221
C -0.279083 3.826491 0.825611
C 0.507364 2.574782 0.575839
C -0.007911 1.454269 0.000682
C -1.352975 1.490949 -0.532036
C -2.136841 2.799195 -0.457357
C 0.768760 0.150223 -0.093680
N 1.846615 2.625556 0.920039
C 2.693476 1.646858 0.514541
C 2.232728 0.480719 -0.016436
C 4.183703 1.774685 0.535483
C 4.668069 0.429523 -0.051794
C 3.350251 -0.291128 -0.458178
O 3.346252 -1.357335 -1.076007
O 5.440598 0.681287 -1.219453
C 5.391901 -0.404762 0.972741
C 6.613281 -0.899071 0.825011
C 0.312120 -0.815185 1.042602
C 0.342107 -2.319236 0.763655
C -0.590189 -2.793506 -0.363871
C -2.061773 -2.588823 -0.045655
C -2.995630 -3.027887 -1.173503
C -4.476951 -2.775333 -0.874316
C -4.863012 -1.296731 -0.814570
C -6.351442 -1.064651 -0.561354
C -6.720191 0.417812 -0.524883
O -1.884996 0.520206 -1.069708
O -3.526067 2.530892 -0.532847
O 1.661622 -2.838550 0.539684
O -0.357500 -4.194126 -0.582173
H -2.330936 4.506803 0.838655

H -2.074425 2.972832 1.675061
H -0.011566 4.563219 0.059463
H 0.024437 4.252549 1.784280
H -1.825564 3.393765 -1.330931
H 0.543032 -0.308216 -1.057948
H 2.218531 3.483794 1.301106
H 4.517079 2.598492 -0.097571
H 4.556218 1.955030 1.544903
H 5.507310 -0.151706 -1.707549
H 4.825951 -0.600924 1.878989
H 7.070098 -1.498645 1.601767
H 7.198031 -0.712484 -0.066964
H 0.921508 -0.618207 1.927556
H -0.711837 -0.557180 1.314507
H 0.012170 -2.819331 1.678517
H -0.344738 -2.249773 -1.285647
H -2.300534 -3.141764 0.869785
H -2.220103 -1.531064 0.153815
H -2.719607 -2.495911 -2.090824
H -2.842827 -4.091009 -1.372096
H -5.082326 -3.263887 -1.645177
H -4.742089 -3.258375 0.073615
H -4.287509 -0.790141 -0.034767
H -4.577858 -0.817104 -1.757751
H -6.934010 -1.568255 -1.340256
H -6.634200 -1.536426 0.385766
H -7.787309 0.563375 -0.343288
H -6.172391 0.938818 0.264682
H -6.473549 0.904490 -1.472477
H -3.600278 1.653564 -0.945581
H 2.107598 -2.352832 -0.181136
H 0.594559 -4.315425 -0.447482

2c-c58, ΔG = 2.1743 kcal/mol, population = 0.29 %
C -1.887347 2.525078 1.746828
C -0.408419 2.653630 2.099978
C 0.469649 1.919707 1.130576
C 0.073389 1.537634 -0.118157
C -1.235766 1.928453 -0.590650
C -2.075895 2.861381 0.278253
C 0.947385 0.683663 -1.024939
N 1.754242 1.670862 1.565123
C 2.690713 1.172937 0.712569
C 2.360889 0.745066 -0.529523

C 4.151895 1.089158 1.030178
C 4.720870 0.318923 -0.171140
C 3.559731 0.341167 -1.209638
O 3.729522 0.047928 -2.387352
O 5.859902 1.000467 -0.699735
C 5.055685 -1.131781 0.084528
C 4.719433 -1.866409 1.137801
C 0.422959 -0.771838 -1.133335
C 0.178854 -1.481789 0.190506
C -0.589464 -2.799617 0.031545
C -2.005543 -2.648337 -0.515224
C -2.883425 -1.628283 0.210611
C -4.312064 -1.593469 -0.327561
C -5.186216 -0.556561 0.378531
C -6.599338 -0.425272 -0.197269
C -7.455466 -1.683261 -0.046222
O -1.698901 1.562011 -1.673208
O -3.436298 2.788335 -0.105444
O 1.393034 -1.740685 0.925637
O -0.670987 -3.443114 1.310970
H -2.483407 3.196161 2.365147
H -2.233145 1.504639 1.927564
H -0.111804 3.708725 2.100521
H -0.218324 2.277690 3.107468
H -1.695161 3.878649 0.096950
H 0.900605 1.094112 -2.037212
H 2.026430 1.981169 2.486460
H 4.598038 2.084576 1.064288
H 4.337404 0.611031 1.991442
H 5.940751 0.734926 -1.627851
H 5.599781 -1.590722 -0.736297
H 4.997254 -2.911398 1.192767
H 4.156828 -1.473137 1.974920
H -0.513089 -0.735744 -1.688434
H 1.125868 -1.354806 -1.736921
H -0.392133 -0.836820 0.857444
H -0.017496 -3.443335 -0.652259
H -1.947351 -2.394448 -1.576513
H -2.476719 -3.634472 -0.466229
H -2.898458 -1.855650 1.280095
H -2.454052 -0.628258 0.111852
H -4.287061 -1.369562 -1.400745
H -4.755500 -2.589185 -0.231907
H -5.253702 -0.809124 1.443530

H -4.688680 0.416344 0.323993
H -7.103669 0.410753 0.296892
H -6.530000 -0.156206 -1.257053
H -8.469807 -1.516620 -0.415423
H -7.037858 -2.525854 -0.600808
H -7.527198 -1.980876 1.003722
H -3.432635 2.389360 -0.992449
H 2.064617 -2.087705 0.323569
H 0.190906 -3.299878 1.728645

2c-c29, ΔG = 2.1743 kcal/mol, population = 0.29 %
C -2.167859 2.529012 1.731490
C -0.686194 2.735311 2.031325
C 0.191612 1.972296 1.084596
C -0.218075 1.516135 -0.134068
C -1.547824 1.846978 -0.596191
C -2.412618 2.774745 0.254294
C 0.658692 0.647729 -1.026242
N 1.489180 1.775334 1.507322
C 2.415320 1.222673 0.674355
C 2.074301 0.721691 -0.537562
C 3.875493 1.142957 0.993072
C 4.439111 0.293439 -0.168151
C 3.261353 0.237844 -1.188989
O 3.405953 -0.158686 -2.337710
O 5.555760 0.944519 -0.754784
C 4.734386 -1.115549 0.280557
C 5.899495 -1.730368 0.126451
C 0.152376 -0.813834 -1.061286
C 0.296870 -1.562159 0.265855
C -0.931492 -2.424283 0.589054
C -2.112300 -1.568267 1.025283
C -3.421625 -2.336366 1.218530
C -3.994011 -2.960840 -0.060602
C -4.214400 -1.982031 -1.219912
C -5.123135 -0.799845 -0.884532
C -5.392689 0.095492 -2.092606
O -2.012367 1.431013 -1.659963
O -3.777923 2.606644 -0.081579
O 1.488636 -2.375060 0.302466
O -0.621570 -3.330292 1.652979
H -2.772957 3.209180 2.330789
H -2.464327 1.507360 1.978929
H -0.430409 3.798359 1.954978

H -0.454559 2.432789 3.054483
H -2.095284 3.800573 0.009065
H 0.596209 1.026780 -2.049743
H 1.772577 2.139698 2.405109
H 4.333734 2.133635 0.981746
H 4.053448 0.699702 1.973807
H 5.673347 0.562335 -1.636608
H 3.888347 -1.627302 0.729785
H 6.040413 -2.750152 0.461599
H 6.748696 -1.234952 -0.327594
H -0.888234 -0.799293 -1.380890
H 0.701685 -1.356361 -1.835088
H 0.433032 -0.863836 1.093521
H -1.188818 -3.001461 -0.310078
H -1.832650 -1.080825 1.963852
H -2.264399 -0.770871 0.297207
H -3.270019 -3.123645 1.960909
H -4.157398 -1.650926 1.646689
H -3.337886 -3.767679 -0.400050
H -4.948797 -3.435416 0.188443
H -3.252541 -1.599104 -1.576202
H -4.645043 -2.532965 -2.062549
H -6.070828 -1.176209 -0.484117
H -4.672191 -0.199187 -0.090550
H -4.457784 0.493755 -2.495126
H -5.886733 -0.461392 -2.893154
H -6.033968 0.940147 -1.830074
H -3.780367 2.187159 -0.958729
H 1.573623 -2.835189 -0.542429
H 0.299560 -3.593396 1.508331

2c-c10, ΔG = 2.2195 kcal/mol, population = 0.26 %
C -2.581368 2.420532 0.298423
C -1.271624 2.673354 1.036922
C -0.130320 1.908004 0.435276
C -0.132135 1.403494 -0.832645
C -1.259206 1.684237 -1.694605
C -2.354635 2.620833 -1.187784
C 1.000047 0.539424 -1.376037
N 0.974969 1.764943 1.245948
C 2.130727 1.235352 0.757838
C 2.191706 0.680574 -0.476159
C 3.426660 1.244583 1.505626
C 4.348629 0.390728 0.607829

C 3.539090 0.246576 -0.718063
O 4.049946 -0.165388 -1.752316
O 5.566453 1.072511 0.348173
C 4.551019 -0.983530 1.194481
C 5.727449 -1.574094 1.355921
C 0.565942 -0.936822 -1.559832
C 0.025432 -1.614899 -0.304784
C -0.800394 -2.864658 -0.638093
C -1.558460 -3.435658 0.557618
C -2.510682 -2.456730 1.253443
C -3.532240 -1.795383 0.327452
C -4.528323 -0.911005 1.074167
C -5.500732 -0.180878 0.148449
C -6.447774 0.754193 0.897712
O -1.368404 1.225401 -2.833939
O -3.544139 2.419600 -1.928652
O 1.062005 -1.929309 0.638999
O 0.039939 -3.871831 -1.231940
H -3.357445 3.096646 0.656906
H -2.919517 1.398495 0.473535
H -1.022418 3.740330 1.008424
H -1.364926 2.404377 2.091113
H -1.983733 3.644631 -1.354394
H 1.258955 0.901889 -2.375311
H 0.958324 2.170488 2.170204
H 3.825498 2.257394 1.586862
H 3.317598 0.842572 2.514196
H 5.923710 0.703147 -0.472784
H 3.633895 -1.490430 1.479351
H 5.799967 -2.568711 1.777037
H 6.651849 -1.079335 1.085557
H -0.209194 -0.954189 -2.325896
H 1.410725 -1.509650 -1.950008
H -0.626163 -0.915272 0.218849
H -1.508595 -2.592133 -1.421947
H -2.121877 -4.304180 0.204521
H -0.827975 -3.801791 1.286347
H -3.043068 -3.000602 2.039288
H -1.935328 -1.679898 1.766042
H -3.017714 -1.186049 -0.422218
H -4.074614 -2.567657 -0.229406
H -5.091622 -1.518769 1.790968
H -3.977142 -0.174913 1.671056
H -4.931952 0.393380 -0.589215

H -6.080905 -0.918829 -0.415560
H -7.131112 1.263235 0.214551
H -7.050603 0.205117 1.625945
H -5.888405 1.520266 1.441565
H -3.263529 1.975722 -2.747132
H 1.706884 -2.483590 0.181459
H 0.416540 -4.404153 -0.520669

2c-c23, ΔG = 2.2885 kcal/mol, population = 0.24 %
C -2.470093 2.367124 0.809700
C -1.088587 2.531003 1.436370
C -0.028696 1.760760 0.705371
C -0.175067 1.261937 -0.555783
C -1.378408 1.576336 -1.295735
C -2.361203 2.576520 -0.690312
C 0.891232 0.412722 -1.236834
N 1.164986 1.616243 1.379885
C 2.263731 1.120263 0.743076
C 2.185352 0.587451 -0.499402
C 3.641579 1.149015 1.325748
C 4.483275 0.385215 0.279388
C 3.502195 0.196680 -0.920261
O 3.874968 -0.205565 -2.014702
O 5.582799 1.185907 -0.134897
C 4.892937 -0.972702 0.787059
C 6.136215 -1.434779 0.800217
C 0.467880 -1.073187 -1.350179
C 0.430746 -1.837606 -0.031159
C -0.501487 -3.056523 -0.061877
C -1.981001 -2.727932 -0.233054
C -2.535030 -1.724621 0.778862
C -4.056152 -1.565492 0.723123
C -4.578330 -1.053752 -0.621075
C -6.075469 -0.727307 -0.627729
C -6.450312 0.474021 0.242114
O -1.627966 1.109240 -2.408593
O -3.619285 2.475954 -1.328738
O 1.745722 -2.280510 0.372989
O -0.347717 -3.791511 1.159541
H -3.165927 3.087742 1.238967
H -2.862440 1.368128 1.007380
H -0.798794 3.588053 1.434957
H -1.100724 2.214684 2.481654
H -1.933476 3.574326 -0.875503

H 1.004487 0.772793 -2.263181
H 1.256598 2.014310 2.303180
H 4.008873 2.172375 1.420887
H 3.670182 0.687563 2.314152
H 5.885571 0.832613 -0.983877
H 4.067874 -1.585236 1.137224
H 6.358220 -2.427345 1.171104
H 6.969432 -0.836327 0.453495
H -0.512596 -1.094253 -1.822483
H 1.151852 -1.585079 -2.033501
H 0.114994 -1.183586 0.782165
H -0.188295 -3.694492 -0.901694
H -2.143637 -2.370506 -1.250854
H -2.531607 -3.670096 -0.150877
H -2.240175 -2.034933 1.783897
H -2.076890 -0.746211 0.611154
H -4.533170 -2.525204 0.953442
H -4.356136 -0.876920 1.517689
H -4.021433 -0.155834 -0.905973
H -4.377341 -1.799483 -1.394948
H -6.385052 -0.527541 -1.658075
H -6.640346 -1.608855 -0.304691
H -7.512921 0.709940 0.150043
H -6.247369 0.289246 1.298904
H -5.878370 1.355337 -0.057298
H -3.449492 2.004813 -2.162337
H 2.156780 -2.730969 -0.376821
H 0.596647 -3.754873 1.370311

2c-c41, ΔG = 2.3532 kcal/mol, population = 0.21 %
C -2.081361 2.613598 1.887715
C -0.584585 2.780593 2.135773
C 0.238765 2.002081 1.153330
C -0.231078 1.554363 -0.046559
C -1.565580 1.923405 -0.461154
C -2.372953 2.872643 0.421337
C 0.582339 0.651951 -0.963005
N 1.548561 1.777031 1.520070
C 2.426745 1.209415 0.645658
C 2.021297 0.709946 -0.546799
C 3.898870 1.110378 0.895126
C 4.398843 0.263917 -0.297423
C 3.169288 0.213237 -1.255723
O 3.251621 -0.187247 -2.409187

O 5.482225 0.919332 -0.940241
C 4.719408 -1.146292 0.128804
C 5.875299 -1.758672 -0.090536
C 0.041837 -0.798243 -0.937288
C 0.262581 -1.531362 0.388063
C -0.946410 -2.389879 0.785673
C -2.095591 -1.533317 1.302181
C -3.444710 -2.258718 1.349187
C -4.101359 -2.415168 -0.027508
C -4.628613 -1.097091 -0.601093
C -5.136675 -1.196402 -2.042580
C -4.025010 -1.436899 -3.065682
O -2.079236 1.521165 -1.507725
O -3.753511 2.741194 0.133561
O 1.454763 -2.343841 0.362257
O -0.576119 -3.305004 1.822541
H -2.647116 3.305502 2.511508
H -2.394620 1.598381 2.141433
H -0.303965 3.837312 2.060028
H -0.324816 2.462390 3.147467
H -2.037178 3.890571 0.168325
H 0.474603 1.010672 -1.989791
H 1.879214 2.136859 2.403641
H 4.368512 2.095400 0.871198
H 4.115962 0.656245 1.862985
H 5.552721 0.542820 -1.829449
H 3.899602 -1.660820 0.621607
H 6.035872 -2.779389 0.232618
H 6.697629 -1.259498 -0.587807
H -1.018924 -0.765098 -1.182023
H 0.521774 -1.367238 -1.737972
H 0.445028 -0.824288 1.199581
H -1.262376 -2.961299 -0.098317
H -1.812155 -1.188382 2.300002
H -2.188332 -0.640320 0.683447
H -3.304037 -3.240764 1.808096
H -4.124225 -1.705408 2.004834
H -3.384035 -2.863302 -0.720519
H -4.931743 -3.124534 0.046009
H -5.435292 -0.735661 0.045256
H -3.846735 -0.333875 -0.567921
H -5.884609 -1.993938 -2.113113
H -5.655324 -0.267098 -2.296754
H -3.266376 -0.653177 -2.997273

H -3.527149 -2.395359 -2.905748
H -4.419022 -1.438224 -4.084387
H -3.795014 2.329780 -0.746644
H 1.493655 -2.805888 -0.485054
H 0.330440 -3.577298 1.616062

2c-c162, ΔG = 2.4787 kcal/mol, population = 0.17 %
C -0.816954 5.368302 0.548499
C 0.593299 4.820126 0.750276
C 0.681079 3.359318 0.423789
C -0.223424 2.703562 -0.359898
C -1.304407 3.440574 -0.962414
C -1.328532 4.957170 -0.821429
C -0.171512 1.207003 -0.605174
N 1.770425 2.691654 0.938188
C 2.032929 1.402811 0.578916
C 1.168389 0.687579 -0.181723
C 3.295517 0.679390 0.933591
C 3.034657 -0.751561 0.410103
C 1.757120 -0.593300 -0.469132
O 1.395520 -1.454025 -1.259542
O 4.117008 -1.191568 -0.394239
C 2.708250 -1.699537 1.537590
C 3.277925 -2.882182 1.722427
C -1.322895 0.472207 0.140866
C -2.024201 -0.554227 -0.733130
C -3.109183 -1.326285 0.018584
C -2.609197 -2.164967 1.195215
C -1.313434 -2.964223 1.012596
C -1.365179 -4.077629 -0.034903
C -0.014928 -4.766101 -0.257241
C 0.543856 -5.492361 0.967148
C 1.849533 -6.227020 0.668177
O -2.207991 2.898395 -1.614668
O -2.633664 5.452849 -1.053557
O -2.640660 0.068201 -1.878466
O -3.805537 -2.175023 -0.908154
H -0.815315 6.454695 0.635207
H -1.489175 4.969808 1.312129
H 1.301304 5.355738 0.107333
H 0.924849 4.979792 1.778226
H -0.640274 5.346428 -1.588437
H -0.305449 1.032329 -1.675562
H 2.438706 3.202857 1.496343

H 4.152196 1.110612 0.412668
H 3.498237 0.715061 2.004958
H 3.769600 -1.879512 -0.980993
H 1.924915 -1.356472 2.207575
H 2.981993 -3.522790 2.542716
H 4.059849 -3.243374 1.066717
H -0.917528 -0.011292 1.029535
H -2.059402 1.198974 0.492339
H -1.290809 -1.262974 -1.124609
H -3.821768 -0.586436 0.409437
H -2.479635 -1.490506 2.045652
H -3.422379 -2.842444 1.472467
H -0.486836 -2.291615 0.768497
H -1.061168 -3.394740 1.985154
H -2.114389 -4.819791 0.265003
H -1.714452 -3.663753 -0.982206
H -0.115690 -5.487753 -1.075434
H 0.711848 -4.018233 -0.592219
H 0.714254 -4.777070 1.775690
H -0.203315 -6.203862 1.336413
H 2.616565 -5.529801 0.321765
H 2.237579 -6.734870 1.554084
H 1.707041 -6.978210 -0.113335
H -3.089181 4.763764 -1.564915
H -2.588410 1.036781 -1.783146
H -3.968818 -1.623577 -1.686651

2c-c19, ΔG = 2.5107 kcal/mol, population = 0.16 %
C -1.898010 2.561499 1.955572
C -0.394831 2.769687 2.119432
C 0.392411 1.977506 1.118848
C -0.129344 1.490649 -0.043721
C -1.487324 1.828174 -0.402678
C -2.272370 2.777101 0.500553
C 0.654636 0.577345 -0.972962
N 1.724019 1.787315 1.421539
C 2.569731 1.235775 0.509670
C 2.113010 0.697501 -0.647027
C 4.059763 1.207336 0.660524
C 4.505665 0.330660 -0.519148
C 3.238793 0.255986 -1.422724
O 3.286565 -0.130067 -2.584735
O 5.575534 0.956110 -1.228474
C 4.875700 -1.091525 -0.167382

C 4.665181 -1.720739 0.982534
C 0.148330 -0.888092 -0.905233
C 0.045793 -1.489234 0.487870
C -0.653716 -2.856107 0.491771
C -2.130235 -2.818615 0.108796
C -2.982361 -1.838509 0.919443
C -4.487973 -2.094378 0.791030
C -5.052462 -1.981109 -0.629345
C -4.898370 -0.593701 -1.251168
C -5.528138 -0.491171 -2.638957
O -2.038419 1.400135 -1.419296
O -3.662322 2.604283 0.289134
O 1.325476 -1.623391 1.141423
O -0.550875 -3.426414 1.803370
H -2.447100 3.255837 2.591522
H -2.172943 1.545827 2.250293
H -0.145060 3.829564 1.994564
H -0.074422 2.491956 3.125671
H -1.977010 3.796959 0.207598
H 0.485652 0.902815 -2.002815
H 2.091494 2.178744 2.276526
H 4.477868 2.208459 0.542363
H 4.370592 0.833107 1.635274
H 5.548837 0.613853 -2.134479
H 5.325682 -1.628826 -0.997560
H 4.952834 -2.757242 1.105185
H 4.201694 -1.245013 1.837463
H -0.840025 -0.912998 -1.362228
H 0.799006 -1.512117 -1.525914
H -0.510707 -0.819269 1.142882
H -0.124759 -3.507345 -0.219000
H -2.211592 -2.604316 -0.958553
H -2.518362 -3.832926 0.243808
H -2.704113 -1.915997 1.972968
H -2.760361 -0.812558 0.616321
H -4.706459 -3.094211 1.180821
H -5.019856 -1.388611 1.437846
H -4.576559 -2.722105 -1.280309
H -6.115681 -2.242887 -0.603981
H -5.355636 0.146285 -0.584679
H -3.841144 -0.329741 -1.318742
H -5.069803 -1.204706 -3.329194
H -6.599446 -0.706758 -2.603857
H -5.401946 0.508010 -3.061966

H -3.739865 2.167573 -0.576164
H 1.968852 -1.983474 0.516497
H 0.331669 -3.185018 2.121239

2c-c53, ΔG = 2.5289 kcal/mol, population = 0.16 %
C -1.910412 2.578554 1.946317
C -0.413136 2.791571 2.146506
C 0.406924 2.028584 1.148524
C -0.084865 1.530856 -0.022623
C -1.453641 1.821678 -0.386911
C -2.246887 2.794582 0.481917
C 0.745276 0.667094 -0.962054
N 1.739439 1.882639 1.471956
C 2.611411 1.332476 0.587496
C 2.190761 0.786387 -0.579651
C 4.096887 1.300684 0.784598
C 4.565113 0.372161 -0.345757
C 3.340225 0.322236 -1.305849
O 3.432527 -0.072480 -2.462266
O 5.697976 0.918671 -1.017212
C 4.842456 -1.054530 0.071557
C 4.544885 -1.628623 1.231351
C 0.274339 -0.811254 -0.997682
C 0.029195 -1.460030 0.355686
C -0.443222 -2.914556 0.238249
C -1.760240 -3.109687 -0.504252
C -2.933298 -2.277086 0.012367
C -4.241845 -2.619115 -0.701123
C -5.466160 -1.879933 -0.155522
C -5.405400 -0.360308 -0.312063
C -6.678347 0.337914 0.161583
O -2.002952 1.341768 -1.381004
O -3.632690 2.651778 0.234338
O 1.179969 -1.425399 1.224931
O -0.585417 -3.464867 1.555302
H -2.477827 3.270463 2.568666
H -2.189920 1.561289 2.231190
H -0.167937 3.855476 2.049300
H -0.112387 2.495225 3.153606
H -1.923695 3.805997 0.190427
H 0.611292 1.042390 -1.980852
H 2.078392 2.272478 2.339287
H 4.525661 2.292954 0.634603
H 4.378099 0.965657 1.782320

H 5.681435 0.570041 -1.921304
H 5.301417 -1.644330 -0.716889
H 4.770525 -2.673065 1.405044
H 4.073380 -1.095249 2.047432
H -0.656346 -0.848122 -1.561202
H 1.012054 -1.392143 -1.559645
H -0.723900 -0.894199 0.904459
H 0.339908 -3.473220 -0.294991
H -1.597812 -2.902043 -1.564984
H -2.011254 -4.172638 -0.438869
H -3.050503 -2.442297 1.087296
H -2.717761 -1.214754 -0.119511
H -4.135937 -2.401195 -1.770393
H -4.414363 -3.697854 -0.624555
H -6.361477 -2.252340 -0.664961
H -5.593204 -2.126467 0.905310
H -4.553867 0.035501 0.245019
H -5.222388 -0.116815 -1.364774
H -7.548913 -0.001859 -0.405875
H -6.869174 0.127646 1.217493
H -6.604740 1.421373 0.046434
H -3.697136 2.157861 -0.601098
H 1.957490 -1.730179 0.736890
H 0.168503 -3.128290 2.060860

2c-c114, ΔG = 2.6054 kcal/mol, population = 0.14 %
C -0.638845 5.175076 1.217526
C 0.809805 4.703475 1.121588
C 0.909905 3.282572 0.652642
C -0.090473 2.630393 -0.009827
C -1.288564 3.347343 -0.355788
C -1.363886 4.842627 -0.074299
C -0.012498 1.160567 -0.385462
N 2.109931 2.654364 0.894213
C 2.368159 1.421188 0.371826
C 1.410264 0.706781 -0.266970
C 3.717766 0.772117 0.395270
C 3.438623 -0.630413 -0.191420
C 2.000012 -0.500967 -0.780262
O 1.533856 -1.317313 -1.562418
O 4.353767 -0.925966 -1.234195
C 3.415084 -1.678745 0.892797
C 4.093813 -2.816804 0.861263
C -0.963847 0.294385 0.494723

C -1.832226 -0.660642 -0.312400
C -2.714585 -1.538919 0.586375
C -3.380099 -2.688548 -0.173337
C -2.431824 -3.791884 -0.654466
C -1.734770 -4.548399 0.479941
C -1.031751 -5.832247 0.027414
C 0.104159 -5.635865 -0.983409
C 1.253554 -4.777496 -0.458526
O -2.261067 2.807821 -0.904214
O -2.713167 5.268294 -0.030713
O -2.704989 0.048231 -1.209535
O -3.706250 -0.719318 1.223029
H -0.674575 6.248860 1.400412
H -1.143988 4.674296 2.046771
H 1.366536 5.334001 0.418876
H 1.310232 4.798305 2.087456
H -0.844436 5.337283 -0.910386
H -0.332323 1.057014 -1.426007
H 2.843735 3.166001 1.362332
H 4.417829 1.306768 -0.249318
H 4.139250 0.741672 1.400979
H 3.933826 -1.596977 -1.792479
H 2.764467 -1.449229 1.731926
H 4.018172 -3.534596 1.667651
H 4.746633 -3.064422 0.033941
H -0.366333 -0.284874 1.201651
H -1.610017 0.941999 1.090332
H -1.181902 -1.305014 -0.910616
H -2.102590 -1.937169 1.397112
H -4.127422 -3.127793 0.493787
H -3.922505 -2.267568 -1.024796
H -3.014545 -4.506366 -1.244965
H -1.687965 -3.376516 -1.339512
H -1.015602 -3.894535 0.980063
H -2.481902 -4.809911 1.237003
H -0.629693 -6.342975 0.909240
H -1.779004 -6.506510 -0.404923
H 0.488454 -6.621403 -1.263740
H -0.290431 -5.194365 -1.903537
H 2.077359 -4.738180 -1.173994
H 0.944505 -3.748276 -0.273825
H 1.647398 -5.180016 0.478853
H -3.215647 4.601187 -0.526647
H -2.541478 1.007556 -1.130110

H -4.078745 -0.175128 0.513204

2c-c288, ΔG = 2.6167 kcal/mol, population = 0.14 %
C -0.672631 5.303044 0.047853
C 0.693932 4.787749 0.493212
C 0.822078 3.305828 0.307742
C 0.039841 2.581907 -0.544768
C -0.934232 3.256613 -1.361759
C -0.984029 4.778830 -1.343754
C 0.121251 1.072644 -0.659820
N 1.819861 2.689491 1.029822
C 2.127415 1.377861 0.816424
C 1.385686 0.600976 -0.010218
C 3.311944 0.687120 1.420651
C 3.103926 -0.785396 0.999230
C 1.995586 -0.699497 -0.092687
O 1.757257 -1.622680 -0.858857
O 4.291266 -1.320964 0.439639
C 2.556778 -1.611312 2.137930
C 3.020456 -2.797716 2.505740
C -1.115818 0.373096 -0.022696
C -1.699011 -0.705529 -0.922982
C -2.699676 -1.602435 -0.187395
C -3.047061 -2.883378 -0.946161
C -1.850365 -3.786458 -1.282952
C -0.879530 -4.067172 -0.129201
C -1.509673 -4.737626 1.091509
C -0.547846 -4.884297 2.276214
C 0.664279 -5.772486 1.995721
O -1.721799 2.652062 -2.103910
O -2.245440 5.236627 -1.794610
O -2.382615 -0.131602 -2.052173
O -3.886700 -0.854253 0.111470
H -0.682448 6.392860 0.046324
H -1.447211 4.959365 0.737509
H 1.487532 5.272905 -0.086667
H 0.876444 5.038355 1.539945
H -0.196075 5.117906 -2.034964
H 0.141694 0.815290 -1.722835
H 2.397636 3.247750 1.641671
H 4.243455 1.062593 0.993429
H 3.355982 0.823450 2.502165
H 4.024013 -2.049553 -0.140133
H 1.700732 -1.175252 2.645022

H 2.563976 -3.347781 3.318147
H 3.872100 -3.251418 2.014802
H -0.815817 -0.076460 0.925628
H -1.889641 1.106581 0.211340
H -0.876225 -1.324628 -1.288929
H -2.267916 -1.865609 0.779333
H -3.771291 -3.430121 -0.337488
H -3.561388 -2.610716 -1.872064
H -2.238079 -4.736493 -1.664075
H -1.285472 -3.342785 -2.107461
H -0.074161 -4.697403 -0.514654
H -0.395279 -3.137280 0.183738
H -2.377098 -4.159421 1.422272
H -1.892801 -5.724707 0.806711
H -0.203977 -3.888992 2.578275
H -1.099125 -5.290318 3.129757
H 1.276279 -5.895118 2.891892
H 0.351300 -6.766988 1.665425
H 1.303330 -5.348825 1.219390
H -2.617313 4.509636 -2.320546
H -2.137696 0.809527 -2.124884
H -4.086697 -0.366794 -0.701965

2c-c165, ΔG = 2.6851 kcal/mol, population = 0.12 %
C -1.736267 3.853807 2.301137
C -0.350976 4.031530 1.688362
C 0.204789 2.741347 1.164379
C -0.540883 1.634164 0.896018
C -1.981895 1.723409 1.015163
C -2.607834 3.080887 1.329117
C 0.079912 0.299089 0.495412
N 1.563085 2.747583 0.905963
C 2.132964 1.730341 0.215855
C 1.462573 0.571399 -0.037138
C 3.498741 1.809396 -0.387163
C 3.681364 0.421399 -1.036331
C 2.290226 -0.253048 -0.860468
O 2.024634 -1.342188 -1.373449
O 3.955618 0.577819 -2.422376
C 4.716123 -0.402026 -0.313572
C 5.766587 -0.974100 -0.885432
C 0.065439 -0.658653 1.720249
C 0.223978 -2.166247 1.495497
C -0.762709 -2.807727 0.512704

C -2.222479 -2.606145 0.878856
C -3.201116 -3.304830 -0.075556
C -3.038913 -2.930515 -1.552784
C -3.222676 -1.443431 -1.851309
C -3.018290 -1.089948 -3.322893
C -3.162788 0.406029 -3.598083
O -2.732446 0.762858 0.843251
O -3.918924 2.903605 1.831226
O 1.556750 -2.557471 1.119195
O -0.497652 -4.219900 0.459943
H -2.181955 4.824633 2.517143
H -1.666876 3.297468 3.239089
H -0.393386 4.748024 0.859859
H 0.345668 4.445819 2.420509
H -2.646442 3.629084 0.374425
H -0.535333 -0.136390 -0.294304
H 2.085431 3.596333 1.070712
H 3.542725 2.586946 -1.151740
H 4.258159 2.033212 0.363345
H 3.804553 -0.280236 -2.843971
H 4.538184 -0.519407 0.751566
H 6.467472 -1.560135 -0.305056
H 5.966546 -0.866412 -1.944116
H 0.846681 -0.341041 2.415027
H -0.885420 -0.513985 2.234324
H 0.070373 -2.641620 2.467961
H -0.571367 -2.382360 -0.480293
H -2.381005 -2.982864 1.894719
H -2.427664 -1.536561 0.898994
H -3.090515 -4.386647 0.027494
H -4.220132 -3.064291 0.244978
H -2.051816 -3.248792 -1.903482
H -3.763739 -3.504420 -2.139825
H -4.226676 -1.133380 -1.538068
H -2.531319 -0.851326 -1.247112
H -2.022451 -1.423361 -3.634454
H -3.733717 -1.648718 -3.935805
H -2.432467 0.980006 -3.021288
H -3.011260 0.638050 -4.654592
H -4.157701 0.761837 -3.317117
H -4.186069 2.014177 1.542910
H 1.713699 -2.259343 0.205312
H 0.465896 -4.306669 0.483059

2c-c59, ΔG = 2.7171 kcal/mol, population = 0.11 %
C -1.991784 2.662485 1.691509
C -0.500091 2.850029 1.951367
C 0.343508 1.982901 1.064498
C -0.104683 1.405025 -0.086721
C -1.441306 1.704659 -0.547426
C -2.249461 2.763155 0.198698
C 0.739571 0.434902 -0.899852
N 1.653301 1.826760 1.465966
C 2.554406 1.202878 0.662651
C 2.172124 0.563117 -0.470012
C 4.029200 1.204287 0.913433
C 4.593553 0.372776 -0.259863
C 3.342783 0.093792 -1.155986
O 3.436018 -0.408935 -2.269203
O 5.514656 1.163201 -1.004020
C 5.182864 -0.931030 0.208435
C 6.394504 -1.372791 -0.101075
C 0.212812 -1.023161 -0.820528
C -0.160093 -1.514113 0.568619
C -0.711343 -2.944959 0.565153
C -2.005793 -3.139771 -0.216954
C -3.166518 -2.232610 0.193913
C -4.467318 -2.615122 -0.512722
C -5.698887 -1.844495 -0.027296
C -5.647699 -0.325825 -0.226752
C -5.504813 0.100966 -1.687529
O -1.952931 1.162285 -1.529484
O -3.624018 2.627855 -0.108304
O 0.940273 -1.449571 1.500365
O -0.941610 -3.359271 1.918825
H -2.566646 3.420014 2.224187
H -2.318014 1.681437 2.045244
H -0.216948 3.894792 1.779175
H -0.259676 2.631539 2.993977
H -1.886577 3.739737 -0.157883
H 0.664968 0.715403 -1.954429
H 1.964332 2.287230 2.308882
H 4.435818 2.216468 0.881175
H 4.267881 0.778558 1.889552
H 5.586195 0.762447 -1.882404
H 4.529525 -1.528956 0.837296
H 6.751629 -2.326875 0.264732
H 7.069769 -0.791196 -0.715829

H -0.673608 -1.087866 -1.449305
H 0.965255 -1.686370 -1.257890
H -0.908355 -0.855267 1.008263
H 0.059077 -3.594102 0.123464
H -1.795736 -3.015915 -1.282468
H -2.302168 -4.184942 -0.086619
H -3.308770 -2.295867 1.276452
H -2.922530 -1.191346 -0.029847
H -4.344907 -2.481160 -1.592398
H -4.647887 -3.684545 -0.361336
H -6.582278 -2.235612 -0.543874
H -5.847568 -2.055675 1.037213
H -6.565638 0.105342 0.184000
H -4.828549 0.101691 0.356937
H -5.608584 1.183511 -1.793116
H -4.533051 -0.179235 -2.097773
H -6.276182 -0.363027 -2.308438
H -3.656304 2.073086 -0.906751
H 1.706076 -1.876455 1.095425
H -0.199281 -3.006254 2.430253

2c-c115, ΔG = 2.7623 kcal/mol, population = 0.11 %
C -2.276797 2.783257 0.680812
C -0.901352 2.880506 1.333230
C 0.120034 2.023290 0.645773
C -0.019506 1.530376 -0.619301
C -1.175485 1.919686 -1.397086
C -2.129253 2.963168 -0.818833
C 0.997330 0.588843 -1.254838
N 1.268591 1.784071 1.368205
C 2.339489 1.174262 0.787035
C 2.263979 0.634067 -0.452408
C 3.685108 1.074085 1.433266
C 4.478973 0.185585 0.450275
C 3.552002 0.102373 -0.802514
O 3.939404 -0.344689 -1.874537
O 5.700828 0.816753 0.094079
C 4.665669 -1.205132 1.001441
C 5.828466 -1.837548 1.084760
C 0.442038 -0.849635 -1.405998
C 0.120045 -1.557613 -0.095440
C -0.894417 -2.697457 -0.251995
C -2.277150 -2.274711 -0.738736
C -2.908395 -1.116544 0.032830

C -4.332103 -0.781735 -0.420594
C -5.363562 -1.896984 -0.205933
C -5.479607 -2.400484 1.237045
C -5.869853 -1.316573 2.241773
O -1.413442 1.472689 -2.521240
O -3.380078 2.894657 -1.477781
O 1.302954 -2.085625 0.544375
O -1.054604 -3.352251 1.014699
H -2.940691 3.546814 1.085940
H -2.725378 1.809024 0.884189
H -0.545734 3.916945 1.309741
H -0.954115 2.595289 2.386048
H -1.662687 3.941974 -1.012076
H 1.198335 0.940360 -2.270924
H 1.357112 2.181713 2.291928
H 4.158328 2.054654 1.510223
H 3.621507 0.650736 2.436832
H 5.987590 0.423483 -0.743013
H 3.747478 -1.685913 1.325015
H 5.890283 -2.841722 1.484445
H 6.752792 -1.369568 0.770081
H -0.453782 -0.784846 -2.020339
H 1.164136 -1.449455 -1.967412
H -0.275208 -0.847428 0.631483
H -0.479313 -3.417457 -0.972749
H -2.219589 -2.018871 -1.799610
H -2.913291 -3.160413 -0.676376
H -2.896962 -1.342129 1.101465
H -2.293744 -0.224227 -0.094097
H -4.661160 0.119541 0.103575
H -4.313785 -0.518406 -1.483224
H -6.342030 -1.527846 -0.531228
H -5.130766 -2.744319 -0.857239
H -6.224928 -3.200995 1.264612
H -4.535469 -2.859101 1.545196
H -5.105858 -0.539926 2.315288
H -6.805899 -0.832269 1.950036
H -6.008135 -1.737161 3.240097
H -3.208642 2.411641 -2.304302
H 1.781235 -2.620885 -0.102799
H -0.169565 -3.392788 1.404862

2c-c54, ΔG = 2.7893 kcal/mol, population = 0.10 %
C -2.331369 2.685243 0.834292

C -0.918998 2.866780 1.380948
C 0.082936 2.014771 0.659286
C -0.124628 1.469098 -0.573837
C -1.348429 1.778055 -1.277801
C -2.294572 2.816513 -0.677908
C 0.877271 0.534207 -1.239244
N 1.284098 1.841136 1.312264
C 2.327856 1.226446 0.694679
C 2.186980 0.632779 -0.514974
C 3.713678 1.179805 1.256858
C 4.459537 0.262549 0.262920
C 3.464227 0.127794 -0.930893
O 3.804386 -0.332492 -2.014677
O 5.657299 0.880854 -0.182899
C 4.684244 -1.107848 0.850089
C 5.848913 -1.741337 0.878729
C 0.372681 -0.930395 -1.334928
C -0.281928 -1.481097 -0.071866
C -0.804846 -2.909965 -0.279620
C -1.668234 -3.418401 0.869105
C -2.906937 -2.571494 1.169364
C -3.873526 -2.432211 -0.009416
C -5.162000 -1.680153 0.332954
C -4.945132 -0.205884 0.675736
C -6.248979 0.551764 0.914364
O -1.654193 1.263796 -2.356501
O -3.582513 2.688700 -1.250648
O 0.589780 -1.444482 1.064057
O 0.313810 -3.818469 -0.377605
H -3.001442 3.432038 1.260286
H -2.714733 1.698527 1.099549
H -0.608482 3.913565 1.284341
H -0.885805 2.630720 2.446691
H -1.874076 3.801175 -0.935977
H 1.018767 0.865791 -2.272301
H 1.416743 2.270914 2.215861
H 4.171573 2.170568 1.257916
H 3.723016 0.802266 2.280567
H 5.881331 0.475333 -1.033417
H 3.793164 -1.577972 1.255699
H 5.936052 -2.731106 1.308278
H 6.750115 -1.287275 0.486417
H -0.361891 -0.983617 -2.139676
H 1.222838 -1.554152 -1.624228

H -1.122647 -0.838067 0.187376
H -1.374171 -2.938411 -1.212228
H -1.973669 -4.438894 0.621695
H -1.042835 -3.481086 1.762877
H -3.440345 -3.033004 2.006313
H -2.600655 -1.583743 1.521417
H -3.380699 -1.916560 -0.840151
H -4.124821 -3.432839 -0.376631
H -5.847197 -1.744779 -0.519019
H -5.664335 -2.179695 1.169686
H -4.315011 -0.121879 1.565699
H -4.391775 0.272829 -0.137606
H -6.812737 0.114208 1.742711
H -6.060960 1.600288 1.155189
H -6.887479 0.522771 0.027358
H -3.450776 2.167424 -2.061226
H 1.189710 -2.197281 0.963504
H 0.702266 -3.739833 -1.256452

2c-c254, ΔG = 2.8156 kcal/mol, population = 0.10 %
C -3.150907 2.395559 -0.638003
C -2.081376 2.645624 0.417208
C -0.787916 1.955367 0.098891
C -0.429588 1.514214 -1.144593
C -1.345739 1.757938 -2.237351
C -2.555825 2.659557 -2.008373
C 0.911495 0.822750 -1.432894
N 0.048529 1.791657 1.179379
C 1.254437 1.178121 1.046514
C 1.686725 0.703170 -0.147015
C 2.208671 0.986850 2.183346
C 3.259888 0.031966 1.581987
C 2.995255 0.128145 0.049023
O 3.824505 -0.230274 -0.775344
O 4.575562 0.467698 1.871093
C 2.994846 -1.390504 2.014564
C 3.915584 -2.224527 2.477591
C 0.866125 -0.542662 -2.167045
C 0.159788 -1.699304 -1.460895
C -1.369124 -1.582418 -1.441949
C -2.060083 -2.717598 -0.697133
C -1.595853 -2.909584 0.749263
C -1.684579 -1.653024 1.616654
C -1.389417 -1.932212 3.090143

C -1.312631 -0.677564 3.964139
C -2.604462 0.137947 4.003045
O -1.202136 1.272569 -3.366391
O -3.508804 2.463846 -3.033877
O 0.540526 -2.938385 -2.077686
O -1.873938 -1.596019 -2.790692
H -4.009758 3.044911 -0.469584
H -3.497906 1.360534 -0.590998
H -1.878613 3.719472 0.500625
H -2.426267 2.318187 1.399546
H -2.181621 3.694741 -2.041177
H 1.465434 1.479966 -2.114000
H -0.245564 2.136688 2.081170
H 2.681863 1.932973 2.452571
H 1.718525 0.587329 3.071694
H 5.143100 0.130901 1.161563
H 1.962870 -1.713176 1.910370
H 3.658432 -3.236227 2.763900
H 4.947521 -1.919261 2.596371
H 0.448129 -0.400824 -3.160775
H 1.903737 -0.847316 -2.302677
H 0.518076 -1.764235 -0.432937
H -1.635425 -0.638109 -0.967416
H -3.133689 -2.509608 -0.711657
H -1.906593 -3.643250 -1.256056
H -2.208806 -3.695096 1.201494
H -0.568407 -3.285627 0.763613
H -0.978079 -0.899210 1.256982
H -2.680197 -1.212258 1.509292
H -2.156305 -2.605894 3.488842
H -0.439122 -2.471768 3.163401
H -1.041754 -0.971465 4.982267
H -0.494309 -0.045427 3.605465
H -2.511563 0.991281 4.677917
H -2.867001 0.524612 3.016650
H -3.441742 -0.473889 4.349614
H -3.025476 2.027538 -3.756404
H 0.036948 -2.993526 -2.902150
H -1.682633 -0.735461 -3.192618

2c-c97, ΔG = 2.9826 kcal/mol, population = 0.07 %
C -2.368981 2.496157 0.771052
C -0.997238 2.663169 1.417645
C 0.059403 1.866126 0.712206

C -0.068559 1.383411 -0.557521
C -1.239054 1.736957 -1.326604
C -2.239583 2.721675 -0.724675
C 0.966564 0.465096 -1.191208
N 1.225096 1.669864 1.421139
C 2.316893 1.122567 0.821953
C 2.246404 0.575941 -0.416278
C 3.682133 1.115177 1.433244
C 4.529945 0.343829 0.397729
C 3.562213 0.163257 -0.815970
O 3.949504 -0.242031 -1.905059
O 5.637089 1.137694 -0.009552
C 4.935158 -1.012379 0.912732
C 6.172115 -1.490510 0.906427
C 0.456626 -0.996900 -1.314177
C -0.157417 -1.581379 -0.051575
C -0.814901 -2.949268 -0.271589
C -1.996494 -2.958454 -1.240581
C -3.027243 -1.829815 -1.117819
C -3.742662 -1.706565 0.227608
C -4.845316 -0.646705 0.195018
C -5.428777 -0.304387 1.568366
C -6.102177 -1.482480 2.272687
O -1.459638 1.298692 -2.458118
O -3.487488 2.606907 -1.382824
O 0.798923 -1.702025 1.023676
O -1.253092 -3.463344 0.994217
H -3.078085 3.204985 1.198432
H -2.751483 1.489492 0.950666
H -0.699147 3.717820 1.403981
H -1.026733 2.362538 2.467071
H -1.823693 3.727081 -0.895073
H 1.143856 0.802054 -2.216147
H 1.303972 2.060440 2.348733
H 4.069671 2.129067 1.546966
H 3.678766 0.644856 2.417998
H 5.928929 0.796299 -0.867361
H 4.119247 -1.615265 1.300747
H 6.390211 -2.480604 1.285630
H 7.004140 -0.905133 0.535904
H -0.292809 -1.016632 -2.104074
H 1.285444 -1.629156 -1.647243
H -0.908259 -0.899130 0.344312
H -0.044076 -3.624492 -0.672174

H -1.596102 -2.951512 -2.257516
H -2.493246 -3.925491 -1.119280
H -2.558652 -0.872985 -1.360072
H -3.778691 -1.989993 -1.898357
H -4.158998 -2.678141 0.506895
H -3.022218 -1.456180 1.010662
H -4.446474 0.267477 -0.255239
H -5.651564 -0.984771 -0.466954
H -4.630415 0.091123 2.206291
H -6.154512 0.506396 1.451660
H -6.555644 -1.171310 3.216390
H -6.891255 -1.910656 1.648014
H -5.389390 -2.278623 2.496263
H -3.295354 2.146775 -2.217864
H 1.566131 -2.184495 0.689255
H -0.561056 -3.222490 1.627180

2c-c124, ΔG = 3.0221 kcal/mol, population = 0.07 %
C -2.099984 2.618203 1.694029
C -0.617778 2.810971 1.999296
C 0.255377 2.030041 1.062835
C -0.151660 1.575772 -0.157747
C -1.474792 1.920131 -0.628510
C -2.335208 2.859223 0.214353
C 0.719059 0.689024 -1.038409
N 1.545047 1.812767 1.498464
C 2.468596 1.236670 0.678804
C 2.130342 0.736816 -0.534111
C 3.922903 1.128267 1.014509
C 4.482636 0.265356 -0.138979
C 3.314750 0.228810 -1.171553
O 3.464269 -0.172863 -2.317965
O 5.616877 0.894333 -0.716069
C 4.747875 -1.147821 0.315946
C 5.904570 -1.781907 0.177940
C 0.188267 -0.763385 -1.077757
C 0.261654 -1.498910 0.262407
C -0.995063 -2.336382 0.538476
C -2.179728 -1.453232 0.904886
C -3.508710 -2.195444 1.063103
C -4.030826 -2.858736 -0.216990
C -4.171592 -1.920858 -1.423011
C -5.043431 -0.682295 -1.188539
C -6.485138 -1.004723 -0.797061

O -1.939021 1.504172 -1.692556
O -3.700137 2.701047 -0.128383
O 1.437229 -2.329569 0.360903
O -0.752121 -3.232522 1.627740
H -2.701001 3.307457 2.287042
H -2.407904 1.600801 1.945128
H -0.350298 3.870460 1.914665
H -0.394264 2.515222 3.026286
H -2.008108 3.881322 -0.033742
H 0.676126 1.066911 -2.063482
H 1.826037 2.175523 2.397627
H 4.401438 2.109277 1.006926
H 4.080095 0.682929 1.997888
H 5.730961 0.513998 -1.599203
H 3.888285 -1.644018 0.757113
H 6.025033 -2.802977 0.516961
H 6.766701 -1.300188 -0.266256
H -0.837393 -0.733925 -1.440958
H 0.760194 -1.323634 -1.822034
H 0.370888 -0.793730 1.088292
H -1.219812 -2.921211 -0.364462
H -1.932126 -0.946012 1.841932
H -2.289027 -0.673475 0.150944
H -3.405166 -2.956109 1.840507
H -4.251447 -1.482623 1.432043
H -3.370868 -3.685074 -0.496926
H -4.998469 -3.317450 0.004265
H -3.180250 -1.593917 -1.749718
H -4.587660 -2.493701 -2.258511
H -4.591084 -0.050893 -0.419343
H -5.043724 -0.082965 -2.103835
H -6.534423 -1.530380 0.158613
H -7.079624 -0.093514 -0.702388
H -6.962900 -1.639400 -1.548551
H -3.699418 2.269993 -1.000198
H 1.544256 -2.810183 -0.469968
H 0.168677 -3.516891 1.529075

2c-c117, ΔG = 3.1978 kcal/mol, population = 0.05 %
C -1.874045 2.579896 2.124635
C -0.370110 2.789800 2.275147
C 0.413001 2.038238 1.239576
C -0.119838 1.563485 0.076947
C -1.497616 1.866902 -0.237798

C -2.262690 2.819552 0.677094
C 0.669944 0.705965 -0.901240
N 1.753842 1.875541 1.517687
C 2.591109 1.328707 0.598016
C 2.127761 0.803718 -0.562717
C 4.081647 1.278134 0.746965
C 4.504011 0.363076 -0.411944
C 3.248542 0.339598 -1.332667
O 3.299557 -0.037702 -2.497384
O 5.620011 0.909551 -1.111148
C 4.780149 -1.072769 -0.026605
C 4.511830 -1.663681 1.131834
C 0.177793 -0.765162 -0.940109
C -0.035430 -1.425431 0.412789
C -0.539578 -2.868579 0.292259
C -1.891048 -3.028890 -0.394975
C -3.037514 -2.212117 0.202982
C -4.380867 -2.561603 -0.438034
C -5.588485 -1.887550 0.220795
C -5.592692 -0.355436 0.178334
C -5.579158 0.221755 -1.236850
O -2.079017 1.410426 -1.224654
O -3.655280 2.671009 0.474343
O 1.144316 -1.423131 1.243380
O -0.634093 -3.440202 1.604339
H -2.418405 3.261371 2.778343
H -2.143414 1.558140 2.402938
H -0.127646 3.854805 2.183244
H -0.035134 2.480217 3.267308
H -1.955366 3.838008 0.392895
H 0.508217 1.097293 -1.909891
H 2.124669 2.251028 2.378328
H 4.516465 2.268098 0.599127
H 4.390660 0.924051 1.729766
H 5.570557 0.576060 -2.019756
H 5.209208 -1.653478 -0.838302
H 4.732047 -2.713065 1.280973
H 4.070626 -1.139989 1.970798
H -0.770672 -0.781959 -1.474066
H 0.888778 -1.350204 -1.531534
H -0.758849 -0.852896 0.993262
H 0.208739 -3.431493 -0.284687
H -1.776687 -2.787800 -1.454923
H -2.146028 -4.092066 -0.352593

H -3.089338 -2.395479 1.279946
H -2.840471 -1.144820 0.076155
H -4.347870 -2.311253 -1.503406
H -4.522124 -3.646435 -0.388823
H -6.500646 -2.253061 -0.263536
H -5.644565 -2.212075 1.265454
H -6.485169 0.000166 0.702052
H -4.740002 0.037539 0.737938
H -4.638090 0.014497 -1.748998
H -6.388096 -0.200537 -1.839432
H -5.715363 1.305822 -1.221677
H -3.743395 2.199395 -0.371917
H 1.901072 -1.729122 0.724499
H 0.145265 -3.122636 2.082976

2c-c22, ΔG = 3.2373 kcal/mol, population = 0.05 %
C -2.305976 2.469606 0.864520
C -0.910375 2.717329 1.428533
C 0.145221 1.972435 0.666295
C -0.023262 1.493043 -0.599776
C -1.258604 1.775241 -1.296642
C -2.279623 2.698981 -0.635087
C 1.019814 0.630448 -1.299140
N 1.347186 1.816726 1.323625
C 2.417377 1.265692 0.692730
C 2.314888 0.735230 -0.550323
C 3.797885 1.218427 1.273796
C 4.543970 0.301927 0.292583
C 3.614156 0.273688 -0.956409
O 4.006365 -0.102188 -2.054518
O 5.814906 0.850351 -0.049166
C 4.697323 -1.132229 0.747048
C 4.083165 -1.724765 1.764331
C 0.559839 -0.844182 -1.455597
C -0.063229 -1.462760 -0.212384
C -0.693014 -2.830116 -0.501143
C -1.518001 -3.373296 0.666060
C -2.609371 -2.432859 1.189903
C -3.590404 -1.935200 0.127604
C -4.694336 -1.051011 0.703312
C -5.640744 -0.493664 -0.358467
C -6.717803 0.419361 0.223230
O -1.521147 1.320971 -2.412506
O -3.555810 2.504936 -1.215460

O 0.849670 -1.548425 0.893920
O 0.306790 -3.783783 -0.896172
H -3.029170 3.135539 1.335123
H -2.616844 1.441448 1.062811
H -0.672995 3.786654 1.391337
H -0.863180 2.425189 2.479638
H -1.937657 3.727741 -0.829291
H 1.159097 1.011127 -2.315039
H 1.449544 2.192565 2.254817
H 4.255965 2.208780 1.257998
H 3.804140 0.865928 2.304554
H 6.034225 0.517232 -0.932430
H 5.362183 -1.709671 0.110973
H 4.251461 -2.772796 1.977078
H 3.396600 -1.203929 2.419834
H -0.183588 -0.882305 -2.251850
H 1.410715 -1.446298 -1.783770
H -0.857082 -0.803091 0.135766
H -1.331701 -2.719899 -1.378844
H -1.968660 -4.314524 0.338861
H -0.834281 -3.615158 1.485757
H -3.167686 -2.959636 1.969708
H -2.148897 -1.571985 1.684074
H -3.055790 -1.366713 -0.639734
H -4.038470 -2.793469 -0.385996
H -5.269068 -1.619327 1.443510
H -4.237954 -0.216427 1.247780
H -5.056327 0.061996 -1.097907
H -6.111416 -1.325164 -0.893791
H -7.382563 0.801021 -0.554812
H -7.331343 -0.111848 0.955959
H -6.268225 1.278556 0.727953
H -3.386624 2.066456 -2.066918
H 1.716796 -1.819127 0.566782
H 0.807050 -4.024258 -0.105927

2c-c40, ΔG = 3.2662 kcal/mol, population = 0.05 %
C -2.432500 2.300115 0.889145
C -1.055214 2.511491 1.509698
C 0.015290 1.758096 0.777576
C -0.121851 1.278059 -0.492440
C -1.318391 1.598609 -1.236273
C -2.338818 2.544698 -0.606520
C 0.935279 0.405817 -1.155065

N 1.201893 1.603403 1.461274
C 2.301724 1.104642 0.834827
C 2.225659 0.562129 -0.404742
C 3.680473 1.155150 1.413635
C 4.539892 0.439643 0.348308
C 3.548015 0.209350 -0.838143
O 3.924440 -0.176993 -1.937816
O 5.581884 1.303171 -0.090487
C 5.046377 -0.889387 0.843092
C 6.306997 -1.296190 0.776790
C 0.483407 -1.074462 -1.287397
C -0.091201 -1.696336 -0.024588
C -0.699318 -3.086533 -0.248540
C -1.887986 -3.136517 -1.208121
C -2.951096 -2.038152 -1.090268
C -3.654723 -1.904714 0.260178
C -4.784240 -0.875897 0.216969
C -5.380047 -0.562141 1.588107
C -6.500549 0.474365 1.526003
O -1.545623 1.162863 -2.367381
O -3.594066 2.397455 -1.243463
O 0.881817 -1.789787 1.038198
O -1.108278 -3.622367 1.018283
H -3.158378 2.978988 1.336464
H -2.774616 1.278748 1.066571
H -0.795898 3.576366 1.497888
H -1.052630 2.202970 2.557215
H -1.958478 3.564634 -0.773409
H 1.079466 0.760312 -2.179397
H 1.285785 2.000263 2.385908
H 4.020597 2.185186 1.533769
H 3.722833 0.671800 2.391083
H 5.866661 0.983795 -0.959032
H 4.286552 -1.537025 1.270636
H 6.599126 -2.271364 1.144694
H 7.085950 -0.665473 0.367310
H -0.273859 -1.115941 -2.068703
H 1.333565 -1.668172 -1.637247
H -0.863008 -1.048120 0.387880
H 0.092584 -3.730709 -0.658990
H -1.494949 -3.126093 -2.227912
H -2.354790 -4.116813 -1.075243
H -2.515263 -1.072740 -1.358232
H -3.707246 -2.236486 -1.857508

H -4.050156 -2.877421 0.570440
H -2.933442 -1.618011 1.029362
H -4.411612 0.052026 -0.228780
H -5.576910 -1.235005 -0.449810
H -5.755387 -1.484947 2.043206
H -4.584308 -0.198926 2.247709
H -7.326045 0.121708 0.902026
H -6.901982 0.693193 2.517967
H -6.139133 1.412284 1.096264
H -3.403935 1.948924 -2.085218
H 1.666649 -2.232560 0.690006
H -0.420713 -3.359080 1.647175

2c-c50, ΔG = 3.2869 kcal/mol, population = 0.04 %
C -2.340491 2.694035 0.669124
C -0.954821 2.868157 1.282455
C 0.077500 2.016110 0.605075
C -0.075032 1.470219 -0.635505
C -1.263965 1.784688 -1.395303
C -2.231098 2.828221 -0.839341
C 0.953781 0.533309 -1.255667
N 1.249802 1.843963 1.310425
C 2.320002 1.231893 0.738646
C 2.230876 0.631824 -0.473824
C 3.684562 1.204045 1.352347
C 4.487085 0.312668 0.379469
C 3.531569 0.150459 -0.844958
O 3.917449 -0.301219 -1.916454
O 5.672928 0.975033 -0.035936
C 4.748517 -1.048428 0.971664
C 5.930603 -1.648285 1.008978
C 0.448396 -0.928325 -1.381841
C -0.276546 -1.481385 -0.162152
C -0.809249 -2.898616 -0.416468
C -1.742208 -3.407679 0.680306
C -2.987626 -2.552520 0.929102
C -3.891018 -2.390557 -0.295810
C -5.190028 -1.633379 -0.008841
C -4.980241 -0.166299 0.367775
C -6.288780 0.600176 0.544450
O -1.521753 1.272544 -2.487237
O -3.490876 2.710356 -1.473341
O 0.529051 -1.448566 1.024906
O 0.288543 -3.812514 -0.602436

H -3.026664 3.443061 1.064443
H -2.740588 1.708595 0.913732
H -0.636814 3.914349 1.205808
H -0.972492 2.626944 2.347470
H -1.792289 3.810572 -1.074753
H 1.144000 0.867612 -2.280091
H 1.343950 2.278554 2.216584
H 4.122051 2.203495 1.381518
H 3.662058 0.815964 2.371919
H 5.928784 0.584712 -0.884618
H 3.871305 -1.544534 1.376929
H 6.043448 -2.633983 1.441808
H 6.820929 -1.169615 0.621165
H -0.242137 -0.974563 -2.224601
H 1.299042 -1.569021 -1.625839
H -1.124201 -0.834530 0.063178
H -1.334808 -2.889250 -1.372608
H -2.046291 -4.422941 0.408663
H -1.167936 -3.482442 1.609455
H -3.566874 -3.018868 1.732192
H -2.690441 -1.571956 1.307568
H -3.352576 -1.867381 -1.092645
H -4.130098 -3.384145 -0.689204
H -5.829945 -1.679312 -0.896449
H -5.739108 -2.141692 0.792426
H -4.399971 -0.100986 1.292589
H -4.378362 0.318687 -0.406282
H -6.901625 0.156769 1.333902
H -6.105204 1.643487 0.810124
H -6.877055 0.589410 -0.377031
H -3.324301 2.188884 -2.277363
H 1.338446 -1.940298 0.838070
H 0.536627 -4.161467 0.262341

2c-c323, ΔG = 3.3691 kcal/mol, population = 0.04 %
C -1.711787 3.955217 1.738878
C -0.274876 4.064005 1.238253
C 0.299848 2.729663 0.866072
C -0.439353 1.612959 0.622332
C -1.883965 1.724390 0.628270
C -2.509863 3.110183 0.763164
C 0.186728 0.245177 0.363985
N 1.675315 2.701300 0.724582
C 2.284131 1.626987 0.167908

C 1.617151 0.460444 -0.058347
C 3.700425 1.643163 -0.311725
C 3.914796 0.207008 -0.833051
C 2.499296 -0.431697 -0.742610
O 2.259229 -1.549142 -1.205141
O 4.324889 0.250178 -2.193277
C 4.856743 -0.572053 0.048643
C 5.943866 -1.203246 -0.372736
C 0.042566 -0.635881 1.637409
C 0.177442 -2.157458 1.514991
C -0.738088 -2.837131 0.488822
C -2.217766 -2.587929 0.724134
C -3.136307 -3.338890 -0.247358
C -2.916122 -3.011906 -1.730116
C -3.080712 -1.536191 -2.109249
C -4.469386 -0.963593 -1.826584
C -4.628706 0.469940 -2.329463
O -2.634554 0.758552 0.496162
O -3.864009 2.990996 1.155729
O 1.526623 -2.602070 1.285789
O -0.500489 -4.254588 0.540974
H -2.156265 4.946132 1.830777
H -1.735130 3.481861 2.723457
H -0.231180 4.712215 0.355377
H 0.363535 4.526909 1.993865
H -2.452877 3.571678 -0.235363
H -0.366490 -0.229216 -0.449333
H 2.197191 3.553861 0.869285
H 3.826914 2.357499 -1.127009
H 4.392925 1.916880 0.485392
H 4.190943 -0.634880 -2.561619
H 4.575286 -0.601424 1.097397
H 6.572902 -1.751832 0.316363
H 6.245986 -1.182584 -1.412288
H 0.771327 -0.295312 2.376990
H -0.943842 -0.439363 2.058044
H -0.075592 -2.569122 2.495814
H -0.456885 -2.476229 -0.508222
H -2.458978 -2.888503 1.749287
H -2.403164 -1.516640 0.652793
H -3.006026 -4.413875 -0.105355
H -4.171995 -3.117370 0.023858
H -1.918106 -3.345550 -2.028770
H -3.620664 -3.607483 -2.320733

H -2.340356 -0.926755 -1.584080
H -2.864542 -1.426514 -3.177660
H -5.225446 -1.605061 -2.293546
H -4.663691 -0.987294 -0.751603
H -3.899389 1.126628 -1.850817
H -4.475599 0.527072 -3.410726
H -5.624910 0.862656 -2.112440
H -4.113909 2.077129 0.934972
H 1.776636 -2.359198 0.376017
H 0.454866 -4.355044 0.658800

2c-c215, ΔG = 3.5699 kcal/mol, population = 0.03 %
C -1.656759 3.832281 1.506807
C -0.173054 3.998725 1.192339
C 0.470554 2.698319 0.814809
C -0.212991 1.596069 0.401193
C -1.642071 1.699946 0.196489
C -2.314740 3.058821 0.379353
C 0.458906 0.247889 0.168220
N 1.852440 2.685008 0.858348
C 2.543739 1.650669 0.320500
C 1.926896 0.496237 -0.058479
C 4.008677 1.703563 0.024878
C 4.305118 0.304862 -0.556813
C 2.898568 -0.347691 -0.679329
O 2.729789 -1.435806 -1.234124
O 4.875472 0.440526 -1.851775
C 5.145799 -0.523945 0.380512
C 6.291524 -1.107624 0.057333
C 0.173390 -0.681265 1.383135
C 0.287289 -2.197628 1.203131
C -0.595805 -2.817371 0.110734
C -2.083104 -2.584171 0.311450
C -2.941515 -3.266692 -0.754857
C -4.445744 -3.038241 -0.573865
C -4.919248 -1.587928 -0.727610
C -4.656902 -0.988537 -2.109271
C -5.282096 0.393780 -2.284533
O -2.342700 0.747513 -0.146655
O -3.701706 2.879802 0.603631
O 1.636873 -2.657796 1.018421
O -0.351022 -4.234126 0.092727
H -2.130523 4.807010 1.622039
H -1.785602 3.281322 2.441648

H -0.038962 4.703283 0.363406
H 0.357857 4.422139 2.047634
H -2.160376 3.607281 -0.563055
H 0.018951 -0.200148 -0.724914
H 2.338944 3.530147 1.122059
H 4.228516 2.469750 -0.720383
H 4.592735 1.926736 0.918795
H 4.803107 -0.420129 -2.288687
H 4.735387 -0.634126 1.380331
H 6.841298 -1.696115 0.780475
H 6.721447 -1.007007 -0.931339
H 0.836692 -0.390087 2.201202
H -0.843086 -0.477621 1.722053
H -0.018956 -2.646004 2.152219
H -0.295771 -2.401204 -0.861477
H -2.367063 -2.953357 1.303851
H -2.265537 -1.510430 0.295393
H -2.628144 -2.916198 -1.743439
H -2.744398 -4.340411 -0.737717
H -4.982533 -3.657809 -1.300594
H -4.740246 -3.405068 0.415591
H -5.996056 -1.554122 -0.528085
H -4.447929 -0.953537 0.027973
H -3.580171 -0.916660 -2.280467
H -5.049091 -1.667285 -2.875188
H -6.366035 0.357307 -2.146560
H -4.876859 1.097525 -1.554397
H -5.085025 0.800310 -3.279147
H -3.896304 1.979029 0.292468
H 1.963553 -2.337333 0.157119
H 0.597600 -4.333579 0.259549

2c-c33, ΔG = 3.6094 kcal/mol, population = 0.03 %
C -2.330053 2.467975 1.445857
C -0.874307 2.596659 1.884173
C 0.059792 1.820764 1.003274
C -0.264673 1.355594 -0.237701
C -1.546375 1.712811 -0.804468
C -2.419107 2.712725 -0.049994
C 0.681249 0.499579 -1.070015
N 1.328417 1.635899 1.509581
C 2.321886 1.136895 0.720992
C 2.065840 0.629177 -0.507955
C 3.766670 1.134725 1.110074

C 4.440098 0.360893 -0.044773
C 3.305195 0.219998 -1.107204
O 3.521053 -0.169288 -2.247842
O 5.504039 1.128247 -0.591727
C 4.863823 -1.017060 0.393769
C 6.075785 -1.528025 0.222815
C 0.206504 -0.971391 -1.159524
C 0.248978 -1.750213 0.149320
C -0.667541 -2.980928 0.145698
C -2.160108 -2.677759 0.059480
C -2.687834 -1.727687 1.139157
C -4.219437 -1.699516 1.267973
C -4.964540 -0.730958 0.343057
C -4.786394 -0.960100 -1.158821
C -5.755376 -0.123461 -1.993197
O -1.951998 1.279934 -1.884951
O -3.752169 2.644272 -0.518172
O 1.588879 -2.184376 0.476148
O -0.435888 -3.738917 1.340495
H -2.950095 3.186932 1.981097
H -2.707409 1.468627 1.670863
H -0.565904 3.648233 1.860671
H -0.750267 2.261292 2.916031
H -2.002702 3.708459 -0.268546
H 0.668600 0.880681 -2.095101
H 1.549540 2.010208 2.420856
H 4.162992 2.150713 1.154839
H 3.922348 0.668906 2.084331
H 5.670896 0.785423 -1.481687
H 4.076120 -1.599011 0.862618
H 6.310299 -2.531617 0.554094
H 6.872007 -0.958508 -0.240077
H -0.810088 -0.958041 -1.548730
H 0.816629 -1.495889 -1.901259
H -0.032061 -1.110200 0.986713
H -0.391287 -3.596252 -0.723302
H -2.384557 -2.299454 -0.937312
H -2.681008 -3.635971 0.142340
H -2.261329 -2.039065 2.095088
H -2.321670 -0.713109 0.957351
H -4.605698 -2.714112 1.117760
H -4.473899 -1.430285 2.297317
H -6.032770 -0.794576 0.580087
H -4.663624 0.295585 0.574906

H -3.763059 -0.713302 -1.449897
H -4.931035 -2.021045 -1.388533
H -5.582682 -0.257401 -3.063243
H -6.792133 -0.399654 -1.783506
H -5.650719 0.940787 -1.767823
H -3.699817 2.178360 -1.370512
H 1.959100 -2.626764 -0.299470
H 0.516994 -3.685351 1.503928

Table S7. Geometry data of conformers of structure **2d**.

2d-c430, ΔG = 0.0000 kcal/mol, population = 19.75 %
C 4.725042 0.131124 -0.342028
C 4.094461 -0.897821 -1.278513
C 2.738093 -1.328090 -0.805318
C 1.939683 -0.582813 0.004320
C 2.434660 0.681984 0.512956
C 3.729494 1.243847 -0.066164
C 0.526175 -0.994606 0.391944
N 2.322771 -2.578847 -1.229698
C 1.214034 -3.150970 -0.704284
C 0.368228 -2.464605 0.116567
C 0.838764 -4.580940 -0.927792
C -0.279031 -4.810626 0.113443
C -0.622322 -3.375529 0.598812
O -1.625900 -3.149751 1.279794
O -1.416831 -5.385744 -0.509877
C 0.225559 -5.611973 1.288220
C -0.360047 -6.699572 1.769691
C -0.498445 -0.094687 -0.356976
C -1.794539 0.187705 0.400749
C -2.519000 1.435061 -0.125740
C -1.823318 2.735529 0.246454
C -2.473564 3.971531 -0.372495
C -1.760260 5.268257 0.005957
C -2.388197 6.510954 -0.622077
C -1.676730 7.809986 -0.245292
C -2.309551 9.045791 -0.882370
O 1.843544 1.333302 1.374385
O 4.270970 2.217651 0.805936
O -2.739869 -0.882433 0.282505
O -3.846644 1.470314 0.412652
H 5.630935 0.541463 -0.788479
H 4.999852 -0.341668 0.603951

H 3.985623 -0.473892 -2.283346
H 4.741696 -1.771121 -1.376244
H 3.450536 1.715097 -1.022328
H 0.401925 -0.808915 1.462222
H 2.944262 -3.129737 -1.804017
H 0.435126 -4.723709 -1.931788
H 1.688942 -5.252486 -0.804508
H -2.172923 -5.230610 0.074448
H 1.128302 -5.222135 1.749739
H 0.050665 -7.222456 2.623766
H -1.257052 -7.107168 1.320871
H -0.740560 -0.539241 -1.325147
H -0.005949 0.855562 -0.560653
H -1.567065 0.351294 1.462268
H -2.579846 1.353364 -1.221235
H -1.820834 2.822239 1.337908
H -0.778070 2.690046 -0.068208
H -2.477996 3.865633 -1.463355
H -3.520267 4.026822 -0.061954
H -1.760002 5.375343 1.096723
H -0.708074 5.203878 -0.294906
H -2.388143 6.403722 -1.713027
H -3.440382 6.576049 -0.321534
H -1.679377 7.917489 0.844617
H -0.624961 7.742183 -0.543248
H -1.781276 9.958495 -0.598118
H -2.292417 8.976067 -1.973273
H -3.352931 9.154469 -0.574424
H 3.534444 2.486500 1.381015
H -2.402471 -1.661628 0.768195
H -4.120219 0.541412 0.459674

2d-c344, ΔG = 0.5290 kcal/mol, population = 8.08 %
C 4.930319 -0.127859 -1.457752
C 4.035811 -1.225821 -2.028048
C 2.827992 -1.470822 -1.172619
C 2.304426 -0.556773 -0.311221
C 2.969826 0.721579 -0.154848
C 4.085403 1.089773 -1.128171
C 1.056852 -0.804948 0.524578
N 2.251120 -2.720973 -1.307279
C 1.202916 -3.090735 -0.533510
C 0.644511 -2.242099 0.371907
C 0.555955 -4.437702 -0.603507

C -0.445352 -4.407963 0.572655
C -0.438525 -2.918419 1.016440
O -1.291947 -2.468477 1.783831
O -1.742538 -4.758587 0.114736
C 0.030532 -5.260684 1.722547
C -0.690256 -6.200884 2.317757
C -0.106450 0.156083 0.132862
C -0.643366 0.988602 1.292704
C -1.910967 1.769275 0.915730
C -1.665350 2.880836 -0.091582
C -2.932437 3.635597 -0.491427
C -2.658200 4.745994 -1.506347
C -3.913299 5.461706 -2.012744
C -4.672112 6.246650 -0.940990
C -5.861221 7.020954 -1.507068
O 2.661284 1.531435 0.719963
O 4.871472 2.137426 -0.592862
O -0.930390 0.196131 2.456269
O -2.464620 2.346389 2.105640
H 5.706564 0.137473 -2.175636
H 5.419915 -0.477556 -0.545624
H 3.692346 -0.946621 -3.030716
H 4.596334 -2.155931 -2.138454
H 3.584601 1.433018 -2.047521
H 1.303800 -0.609318 1.570544
H 2.660556 -3.382528 -1.950443
H 0.010756 -4.555619 -1.541643
H 1.284653 -5.245845 -0.530921
H -2.375007 -4.429030 0.769552
H 1.039424 -5.043282 2.061423
H -0.288263 -6.771993 3.144693
H -1.695483 -6.437186 1.992211
H -0.921899 -0.428748 -0.302098
H 0.235461 0.830718 -0.651425
H 0.123883 1.700659 1.605996
H -2.635037 1.053552 0.497598
H -0.934751 3.578319 0.332136
H -1.211066 2.450773 -0.987944
H -3.652790 2.927795 -0.918117
H -3.398148 4.050464 0.403881
H -1.979265 5.481863 -1.060030
H -2.125018 4.318483 -2.362407
H -3.628656 6.150956 -2.815130
H -4.589434 4.727442 -2.466348

H -5.024183 5.565350 -0.161720
H -3.980423 6.941150 -0.451628
H -6.576585 6.345457 -1.983858
H -6.391200 7.569324 -0.725195
H -5.536458 7.743432 -2.260602
H 4.327346 2.533922 0.109009
H -1.176711 -0.714241 2.190921
H -2.342731 1.666203 2.785709

2d-c622, ΔG = 0.5773 kcal/mol, population = 7.45 %
C 4.610138 -0.635151 1.730950
C 4.284691 -1.701944 0.686250
C 2.881310 -1.569061 0.175332
C 2.177385 -0.400439 0.189115
C 2.773856 0.776819 0.768550
C 4.237607 0.732795 1.189896
C 0.781581 -0.266549 -0.398577
N 2.303832 -2.720209 -0.315238
C 0.980276 -2.754269 -0.634683
C 0.211304 -1.636249 -0.618532
C 0.249952 -4.008885 -0.995815
C -1.228624 -3.561883 -1.005269
C -1.149823 -2.006508 -0.896442
O -2.132530 -1.291925 -1.045596
O -1.834349 -3.914034 -2.241510
C -1.971026 -4.095219 0.192733
C -3.123940 -4.748534 0.142443
C 0.836996 0.569596 -1.703964
C -0.429007 1.351635 -2.067962
C -0.898587 2.283845 -0.942827
C -2.145497 3.071838 -1.320779
C -2.704907 3.939854 -0.190792
C -3.166176 3.155452 1.037568
C -3.795583 4.044090 2.110496
C -4.317998 3.281923 3.332483
C -3.228431 2.574884 4.139887
O 2.159486 1.842648 0.916330
O 4.508725 1.749397 2.136423
O -0.176762 2.095823 -3.270493
O 0.143039 3.212316 -0.603636
H 5.671108 -0.662589 1.979122
H 4.043922 -0.821372 2.646539
H 4.968097 -1.612196 -0.165685
H 4.430474 -2.700804 1.100803

H 4.814415 0.917669 0.268967
H 0.167579 0.262005 0.331766
H 2.833437 -3.579568 -0.294852
H 0.535719 -4.351087 -1.992157
H 0.449274 -4.816795 -0.290950
H -2.626483 -3.365896 -2.337341
H -1.497665 -3.889104 1.148662
H -3.610103 -5.093433 1.045932
H -3.610703 -4.969312 -0.799202
H 1.089815 -0.089590 -2.536295
H 1.653871 1.289833 -1.622034
H -1.241564 0.665189 -2.303271
H -1.133181 1.666420 -0.072990
H -1.915047 3.697187 -2.186165
H -2.907885 2.355749 -1.644191
H -1.947651 4.668839 0.110029
H -3.550214 4.514886 -0.582679
H -3.891933 2.392421 0.731013
H -2.317879 2.616706 1.466257
H -3.059527 4.787796 2.438155
H -4.621755 4.607023 1.663345
H -4.845829 3.983534 3.985580
H -5.063535 2.548881 3.005515
H -2.736284 1.792293 3.559336
H -2.459274 3.283798 4.459096
H -3.643416 2.107454 5.035522
H 3.812255 2.415334 2.012359
H 0.392067 2.836187 -3.016935
H 0.808805 2.745376 -0.068224

2d-c144, ΔG = 0.6212 kcal/mol, population = 6.91 %
C 3.757719 0.229395 -2.033583
C 3.004474 -1.059768 -2.354924
C 1.969891 -1.387785 -1.319164
C 1.413238 -0.473180 -0.479490
C 1.874585 0.899856 -0.534661
C 2.761246 1.325811 -1.699603
C 0.327696 -0.810274 0.533648
N 1.592339 -2.717495 -1.261365
C 0.703203 -3.149479 -0.334773
C 0.120688 -2.297874 0.551967
C 0.265860 -4.574377 -0.205349
C -0.618598 -4.559344 1.061427
C -0.781123 -3.044840 1.373438

O -1.608701 -2.636473 2.190264
O -1.890688 -5.118500 0.767914
C 0.070326 -5.227349 2.224995
C -0.453890 -6.199335 2.958669
C -1.001656 -0.059571 0.220381
C -1.484016 0.834442 1.352827
C -2.803572 1.537615 1.018350
C -2.728750 2.496926 -0.165872
C -1.595312 3.521981 -0.108978
C -1.594807 4.473456 -1.304059
C -0.412894 5.441358 -1.291781
C -0.389273 6.397823 -2.483181
C 0.805495 7.349607 -2.460870
O 1.564925 1.743740 0.306951
O 3.407566 2.547522 -1.397036
O -1.651477 0.122652 2.591350
O -3.242741 2.269600 2.172337
H 4.373956 0.527538 -2.881896
H 4.416713 0.075891 -1.175637
H 2.497251 -0.966577 -3.322088
H 3.700658 -1.895371 -2.448305
H 2.086911 1.466707 -2.559067
H 0.664800 -0.480548 1.519817
H 2.021862 -3.375706 -1.895069
H -0.334938 -4.874660 -1.065518
H 1.113842 -5.256199 -0.130667
H -2.500774 -4.816617 1.456360
H 1.061246 -4.842201 2.448178
H 0.093493 -6.630637 3.786841
H -1.437031 -6.601075 2.748297
H -1.779915 -0.786757 -0.027706
H -0.863812 0.551100 -0.671598
H -0.724846 1.591480 1.550665
H -3.546871 0.759698 0.792153
H -2.641414 1.906390 -1.082055
H -3.690934 3.014221 -0.230451
H -1.671553 4.098401 0.817505
H -0.630608 3.010055 -0.074598
H -1.569969 3.888367 -2.230946
H -2.532534 5.040461 -1.324604
H -0.431226 6.022979 -0.362814
H 0.520642 4.866844 -1.273285
H -0.376061 5.815558 -3.410758
H -1.319056 6.976420 -2.498281

H 0.800441 7.962832 -1.555817
H 1.747546 6.795136 -2.479388
H 0.798024 8.023391 -3.320464
H 2.911559 2.920682 -0.647776
H -1.767476 -0.833102 2.418693
H -3.072790 1.681444 2.922805

2d-c202, ΔG = 0.6802 kcal/mol, population = 6.26 %
C 4.618788 -0.027924 -1.842039
C 3.726591 -1.187469 -2.278217
C 2.631313 -1.460289 -1.290115
C 2.155215 -0.541663 -0.406213
C 2.767829 0.771242 -0.360031
C 3.751889 1.158449 -1.459500
C 1.015193 -0.818505 0.563681
N 2.111716 -2.741965 -1.321469
C 1.173462 -3.134371 -0.426679
C 0.666724 -2.278929 0.502464
C 0.600672 -4.515777 -0.376102
C -0.267802 -4.488571 0.901663
C -0.304517 -2.982198 1.282092
O -1.099813 -2.543717 2.115633
O -1.582061 -4.935971 0.606040
C 0.383807 -5.255346 2.025538
C -0.209359 -6.200698 2.741274
C -0.230355 0.070736 0.266611
C -0.674615 0.928555 1.445187
C -1.985791 1.678964 1.167904
C -1.848271 2.769783 0.118149
C -3.146712 3.527075 -0.156315
C -2.990055 4.597501 -1.235142
C -4.274793 5.378103 -1.506065
C -4.121091 6.448356 -2.586324
C -5.408104 7.230646 -2.841779
O 2.514504 1.594844 0.519723
O 4.534692 2.261836 -1.045354
O -0.837527 0.164917 2.651650
O -2.437160 2.276043 2.390829
H 5.298836 0.248687 -2.647876
H 5.220832 -0.320565 -0.978457
H 3.264009 -0.960826 -3.245679
H 4.319539 -2.092862 -2.420253
H 3.137657 1.443295 -2.328523
H 1.359388 -0.572559 1.570954

H 2.483471 -3.404348 -1.986497
H -0.034417 -4.703391 -1.243669
H 1.377716 -5.280767 -0.353194
H -2.160046 -4.610845 1.311436
H 1.409891 -4.967194 2.235919
H 0.315649 -6.706883 3.541101
H -1.229062 -6.506590 2.543871
H -1.059509 -0.567973 -0.050708
H -0.009542 0.723877 -0.577004
H 0.103992 1.663341 1.664565
H -2.733763 0.944264 0.833358
H -1.071940 3.470536 0.443683
H -1.498059 2.318525 -0.813858
H -3.921531 2.814762 -0.461846
H -3.498804 3.990497 0.768554
H -2.199928 5.296322 -0.937371
H -2.648734 4.128231 -2.165121
H -5.067659 4.680613 -1.800111
H -4.612607 5.850751 -0.576565
H -3.323777 7.140166 -2.294458
H -3.789698 5.974281 -3.516305
H -5.743454 7.739510 -1.934117
H -5.270095 7.987628 -3.616922
H -6.213500 6.565487 -3.164446
H 4.049848 2.655266 -0.299630
H -1.052436 -0.766053 2.436410
H -2.242349 1.613777 3.071663

2d-c402, ΔG = 0.9275 kcal/mol, population = 4.12 %
C 5.033961 -0.369608 1.204442
C 4.701726 -1.475950 0.204642
C 3.231399 -1.542227 -0.082315
C 2.383583 -0.482348 0.057661
C 2.895280 0.769477 0.556563
C 4.398601 0.930542 0.747511
C 0.909105 -0.546363 -0.309964
N 2.750521 -2.764981 -0.497193
C 1.411422 -2.983769 -0.616114
C 0.506675 -1.983390 -0.466894
C 0.815581 -4.328818 -0.888133
C -0.695385 -4.091406 -0.674060
C -0.816615 -2.539817 -0.554036
O -1.899896 -1.969492 -0.553886
O -1.423117 -4.531244 -1.812450

C -1.169746 -4.716527 0.612316
C -2.208018 -5.534056 0.721910
C 0.649851 0.284157 -1.594000
C -0.750547 0.884657 -1.745275
C -1.156072 1.771371 -0.559623
C -2.533159 2.393985 -0.739074
C -3.047256 3.095399 0.515979
C -4.422883 3.732695 0.316393
C -5.013267 4.364773 1.579646
C -4.226655 5.561099 2.118173
C -4.891400 6.206474 3.332923
O 2.171482 1.741942 0.812858
O 4.670157 1.981454 1.655429
O -0.799322 1.630989 -2.972075
O -0.199441 2.828065 -0.383371
H 6.114037 -0.249062 1.287930
H 4.645162 -0.627511 2.192316
H 5.225543 -1.295693 -0.740999
H 5.047184 -2.442558 0.574847
H 4.795472 1.186337 -0.248622
H 0.345143 -0.106196 0.513638
H 3.391936 -3.541004 -0.574413
H 0.996362 -4.631538 -1.921150
H 1.225248 -5.099070 -0.233536
H -2.285503 -4.092048 -1.786364
H -0.596404 -4.434322 1.490917
H -2.500354 -5.939963 1.681826
H -2.788917 -5.832065 -0.141899
H 0.856601 -0.338980 -2.465912
H 1.365780 1.108063 -1.627260
H -1.490160 0.091311 -1.845887
H -1.183119 1.149443 0.340791
H -2.495442 3.099633 -1.573130
H -3.230460 1.601952 -1.029610
H -3.104189 2.371157 1.337376
H -2.322330 3.852876 0.819673
H -4.357164 4.492306 -0.471291
H -5.115214 2.968838 -0.053679
H -6.038812 4.687310 1.369701
H -5.088159 3.601811 2.363613
H -3.213355 5.249128 2.385460
H -4.118278 6.304239 1.320442
H -5.896553 6.560637 3.088667
H -4.984049 5.490853 4.154336

H -4.316191 7.060000 3.698395
H 3.877814 2.543709 1.649548
H -0.291923 2.440213 -2.818775
H 0.592817 2.457813 0.044570

2d-c43, ΔG = 1.0040 kcal/mol, population = 3.62 %
C 5.269285 0.108369 -0.368178
C 4.678096 -1.006570 -1.227845
C 3.293890 -1.380927 -0.787724
C 2.462332 -0.549665 -0.102108
C 2.942707 0.760616 0.290458
C 4.274456 1.250851 -0.269604
C 1.035694 -0.918043 0.280425
N 2.890164 -2.662542 -1.119245
C 1.710442 -3.154169 -0.670447
C 0.840933 -2.385611 0.039943
C 1.248597 -4.559655 -0.895824
C -0.025542 -4.656280 -0.024688
C -0.284363 -3.184881 0.401476
O -1.334321 -2.832966 0.943345
O -1.113990 -5.118282 -0.811510
C 0.213405 -5.489160 1.209551
C -0.543521 -6.506700 1.596248
C -0.000894 -0.054816 -0.505287
C -0.971930 0.720477 0.377598
C -1.843315 1.685121 -0.440076
C -2.584333 2.684088 0.445844
C -3.405281 3.702758 -0.340782
C -4.138003 4.702161 0.552661
C -4.968691 5.717830 -0.229809
C -5.699655 6.723628 0.658877
C -6.530673 7.730254 -0.134882
O 2.312305 1.503410 1.043616
O 4.776690 2.308398 0.525301
O -1.872543 -0.127790 1.105704
O -2.749747 0.931546 -1.257878
H 6.205203 0.462393 -0.800693
H 5.481762 -0.265103 0.636445
H 4.628309 -0.686776 -2.275057
H 5.319508 -1.889190 -1.201554
H 4.057128 1.619810 -1.284760
H 0.905511 -0.704174 1.345301
H 3.529671 -3.262731 -1.619537
H 0.985552 -4.717297 -1.943253

H 2.011044 -5.290589 -0.624656
H -1.927784 -4.884837 -0.342263
H 1.072391 -5.186332 1.801697
H -0.318255 -7.056874 2.500741
H -1.400773 -6.829322 1.018841
H -0.560390 -0.687999 -1.197465
H 0.539030 0.668258 -1.119325
H -0.385867 1.306683 1.092655
H -1.201275 2.233336 -1.134405
H -3.232840 2.131846 1.133155
H -1.845130 3.207467 1.062020
H -2.744355 4.247237 -1.025459
H -4.129666 3.174205 -0.966345
H -4.791478 4.157203 1.243597
H -3.410093 5.234183 1.175999
H -4.316857 6.259171 -0.925365
H -5.700391 5.185519 -0.848552
H -6.348071 6.181916 1.355733
H -4.967476 7.257233 1.274195
H -5.900446 8.306524 -0.817499
H -7.290775 7.223268 -0.735239
H -7.041727 8.436189 0.523440
H 4.012729 2.636166 1.029821
H -1.606850 -1.067669 1.048620
H -3.112101 0.254824 -0.666017

2d-c425, ΔG = 1.1854 kcal/mol, population = 2.67 %
C 4.993483 -0.601344 1.402882
C 4.655054 -1.642553 0.338138
C 3.201671 -1.609171 -0.027706
C 2.410087 -0.506767 0.112537
C 2.965542 0.691950 0.689330
C 4.462331 0.753343 0.971398
C 0.955201 -0.469979 -0.328519
N 2.674098 -2.784740 -0.515356
C 1.332288 -2.920019 -0.706025
C 0.479979 -1.874260 -0.560157
C 0.675439 -4.217809 -1.054864
C -0.828615 -3.900002 -0.904137
C -0.867167 -2.349339 -0.725861
O -1.915088 -1.717033 -0.747713
O -1.520890 -4.251359 -2.094354
C -1.404569 -4.544920 0.330014
C -2.496160 -5.297407 0.354855

C 0.807110 0.420351 -1.589969
C -0.547863 1.108011 -1.783596
C -0.960686 1.971337 -0.583181
C -2.290218 2.678867 -0.798849
C -2.818045 3.368345 0.457422
C -4.163592 4.058548 0.232751
C -4.689497 4.827893 1.447714
C -5.025554 3.951676 2.655905
C -5.631526 4.746415 3.811452
O 2.286577 1.696224 0.945683
O 4.740323 1.745714 1.941280
O -0.491619 1.901703 -2.979884
O 0.045158 2.961583 -0.318749
H 6.072178 -0.550169 1.550669
H 4.534227 -0.875968 2.355390
H 5.240765 -1.455788 -0.569148
H 4.922157 -2.642583 0.683113
H 4.931438 1.027709 0.012321
H 0.377027 -0.026883 0.483422
H 3.273123 -3.594168 -0.589326
H 0.889978 -4.496451 -2.088248
H 1.008744 -5.031583 -0.409681
H -2.361538 -3.771315 -2.087450
H -0.861256 -4.335096 1.247076
H -2.861683 -5.720567 1.281657
H -3.050055 -5.523713 -0.547492
H 1.019642 -0.182397 -2.474757
H 1.569922 1.201172 -1.558521
H -1.326475 0.364806 -1.951926
H -1.067034 1.317321 0.288315
H -2.179939 3.406077 -1.607588
H -3.017444 1.936151 -1.141820
H -2.908297 2.625635 1.256042
H -2.085901 4.104967 0.800155
H -4.066253 4.751191 -0.610036
H -4.907408 3.312118 -0.070139
H -3.951758 5.582472 1.744900
H -5.589581 5.379558 1.155508
H -5.723469 3.167121 2.343363
H -4.124972 3.439216 3.004714
H -6.556884 5.241202 3.504519
H -5.863162 4.102806 4.662948
H -4.941782 5.521164 4.157107
H 3.985417 2.356702 1.914655

H 0.050911 2.674197 -2.768424
H 0.792412 2.527632 0.129940

2d-c31, ΔG = 1.1973 kcal/mol, population = 2.61 %
C 5.220531 -0.884351 -0.245372
C 4.196388 -1.038064 -1.366925
C 2.793112 -1.126999 -0.845428
C 2.398956 -0.656074 0.368771
C 3.386433 -0.064662 1.247031
C 4.777276 0.224489 0.692235
C 0.953194 -0.687085 0.844079
N 1.883855 -1.730866 -1.696153
C 0.607158 -1.963393 -1.296676
C 0.148772 -1.544709 -0.088977
C -0.404261 -2.685379 -2.129797
C -1.608147 -2.835583 -1.173788
C -1.220713 -1.952092 0.053128
O -2.025644 -1.671137 0.933071
O -2.778366 -2.300107 -1.777966
C -1.775686 -4.261208 -0.717279
C -2.906830 -4.950831 -0.776551
C 0.338360 0.729626 0.938810
C 0.440982 1.538553 -0.353024
C -0.787016 2.424941 -0.599452
C -2.003736 1.606985 -1.009694
C -3.282759 2.428811 -1.199194
C -3.764637 3.172330 0.051040
C -4.054573 2.268251 1.248869
C -4.624144 3.022312 2.449663
C -4.896193 2.115957 3.648888
O 3.146880 0.239025 2.416817
O 5.696535 0.407608 1.752782
O 1.638097 2.344909 -0.382463
O -0.502317 3.358954 -1.647024
H 6.202150 -0.654486 -0.660174
H 5.303569 -1.816194 0.319187
H 4.250493 -0.177411 -2.043586
H 4.417731 -1.921643 -1.968373
H 4.681567 1.158807 0.116738
H 0.922185 -1.110783 1.851439
H 2.205316 -2.083540 -2.585616
H -0.694220 -2.083452 -2.992929
H -0.024259 -3.639600 -2.496961
H -3.396561 -2.092683 -1.062131

H -0.879083 -4.711162 -0.300145
H -2.958057 -5.971174 -0.418799
H -3.810100 -4.524067 -1.194035
H 0.825716 1.280948 1.745252
H -0.704551 0.613272 1.231944
H 0.548416 0.878699 -1.216481
H -0.996397 2.978530 0.326734
H -1.760596 1.095149 -1.945666
H -2.178823 0.826518 -0.268739
H -3.128426 3.148697 -2.006362
H -4.074328 1.751301 -1.535217
H -3.028437 3.928778 0.341483
H -4.675118 3.724050 -0.204677
H -4.757219 1.482527 0.946964
H -3.139871 1.754118 1.559971
H -3.924301 3.813254 2.740037
H -5.549850 3.526511 2.152722
H -5.616179 1.334070 3.393056
H -3.978964 1.623923 3.983197
H -5.300304 2.678389 4.493434
H 5.149226 0.582839 2.537482
H 1.735138 2.778170 0.475328
H 0.424884 3.610982 -1.523734

2d-c256, ΔG = 1.2349 kcal/mol, population = 2.45 %
C 5.175093 -0.521283 1.177516
C 4.804267 -1.580904 0.141926
C 3.330426 -1.596870 -0.131531
C 2.512931 -0.521011 0.056938
C 3.063645 0.699762 0.590341
C 4.572678 0.812077 0.774167
C 1.032729 -0.534810 -0.291486
N 2.812283 -2.790865 -0.583672
C 1.466219 -2.972620 -0.683505
C 0.590355 -1.955556 -0.483416
C 0.831473 -4.293401 -0.984111
C -0.666521 -4.028085 -0.716488
C -0.747656 -2.477557 -0.554423
O -1.816339 -1.882040 -0.512139
O -1.440619 -4.424412 -1.839799
C -1.113525 -4.673087 0.570292
C -2.169281 -5.465566 0.695580
C 0.778917 0.339148 -1.546975
C -0.608155 0.977014 -1.663755

C -0.981197 1.833546 -0.445313
C -2.345497 2.490650 -0.591567
C -2.828274 3.180188 0.682278
C -4.205130 3.823347 0.526615
C -4.702546 4.509289 1.797553
C -6.083215 5.146760 1.645714
C -6.573421 5.827229 2.922553
O 2.369322 1.683251 0.883427
O 4.880013 1.820303 1.718459
O -0.649657 1.765199 -2.864145
O 0.001641 2.861481 -0.245297
H 6.258740 -0.433354 1.255433
H 4.787303 -0.803810 2.158971
H 5.325414 -1.382155 -0.801478
H 5.124656 -2.569362 0.474961
H 4.969424 1.094055 -0.214856
H 0.492383 -0.103148 0.552150
H 3.432598 -3.579543 -0.696416
H 0.973612 -4.562702 -2.032320
H 1.242318 -5.095544 -0.369861
H -2.290781 -3.965640 -1.775942
H -0.503852 -4.428883 1.435776
H -2.439838 -5.887763 1.654851
H -2.786722 -5.726454 -0.154690
H 0.961167 -0.261979 -2.439547
H 1.513279 1.147064 -1.563991
H -1.368966 0.206415 -1.783519
H -1.013431 1.182842 0.434652
H -2.307009 3.209396 -1.414319
H -3.063273 1.717843 -0.884173
H -2.861862 2.448434 1.497871
H -2.101027 3.940788 0.978557
H -4.172253 4.554852 -0.289044
H -4.929338 3.058863 0.222167
H -4.731123 3.779067 2.614714
H -3.981877 5.277939 2.099824
H -6.053780 5.877105 0.829989
H -6.801406 4.377848 1.341675
H -5.888058 6.621058 3.231341
H -7.560537 6.273995 2.784886
H -6.642902 5.111495 3.746015
H 4.105591 2.406641 1.737048
H -0.122493 2.557013 -2.688015
H 0.790716 2.458487 0.158271

2d-c56, ΔG = 1.3592 kcal/mol, population = 1.99 %

C 5.333517 -0.986609 -0.895455
C 4.074807 -1.140084 -1.745690
C 2.824669 -1.117553 -0.917404
C 2.740221 -0.558509 0.320712
C 3.927823 0.020647 0.912938
C 5.166820 0.196004 0.041640
C 1.440535 -0.477020 1.109163
N 1.719118 -1.712604 -1.498807
C 0.560681 -1.853153 -0.804688
C 0.409622 -1.343790 0.444960
C -0.642026 -2.567555 -1.335469
C -1.595819 -2.605570 -0.121450
C -0.902315 -1.677164 0.924410
O -1.469574 -1.311820 1.946880
O -2.854618 -2.047681 -0.474918
C -1.704459 -3.994118 0.452346
C -2.843480 -4.622298 0.710159
C 0.930511 0.978833 1.234758
C 0.777367 1.698531 -0.105236
C -0.481415 2.573227 -0.181148
C -1.748972 1.736724 -0.267840
C -3.036275 2.557813 -0.269351
C -4.282168 1.675660 -0.364087
C -5.600493 2.449573 -0.441575
C -5.944473 3.243819 0.819465
C -7.303183 3.936537 0.729626
O 3.975377 0.401107 2.083742
O 6.311536 0.379902 0.853613
O 1.941999 2.489607 -0.424834
O -0.408828 3.405880 -1.343927
H 6.204314 -0.839002 -1.534536
H 5.499879 -1.889221 -0.302522
H 4.011744 -0.321932 -2.472282
H 4.113512 -2.066444 -2.321965
H 4.986502 1.102224 -0.557845
H 1.622097 -0.835610 2.125760
H 1.812481 -2.131118 -2.412587
H -1.102753 -1.997674 -2.144361
H -0.393100 -3.557812 -1.719160
H -3.273296 -1.745209 0.344066
H -0.752301 -4.469620 0.670954
H -2.848585 -5.618221 1.134184

H -3.802071 -4.167538 0.494317
H 1.616344 1.543586 1.868698
H -0.022663 0.949956 1.762213
H 0.719428 0.980532 -0.925647
H -0.520160 3.209313 0.716316
H -1.694382 1.128317 -1.176689
H -1.776786 1.040749 0.572935
H -3.072194 3.161751 0.642156
H -3.024502 3.259182 -1.108204
H -4.191868 1.037900 -1.249463
H -4.311326 0.997816 0.497146
H -5.569099 3.131488 -1.299558
H -6.413925 1.743979 -0.642487
H -5.933737 2.566437 1.680451
H -5.170866 3.992810 1.009840
H -7.527657 4.497800 1.639304
H -7.330340 4.636988 -0.109434
H -8.105604 3.209404 0.579030
H 5.967257 0.633968 1.727043
H 2.197267 2.988705 0.361720
H 0.524364 3.652312 -1.427471

2d-c28, ΔG = 1.3730 kcal/mol, population = 1.94 %
C 5.290070 -1.094354 -1.080030
C 3.986893 -1.186052 -1.869459
C 2.780336 -1.130318 -0.980553
C 2.777115 -0.590870 0.269043
C 4.013298 -0.065950 0.810321
C 5.213148 0.078662 -0.119568
C 1.521064 -0.472412 1.120960
N 1.625660 -1.670942 -1.518264
C 0.493281 -1.769945 -0.775325
C 0.421472 -1.275091 0.486985
C -0.764003 -2.418877 -1.262047
C -1.666662 -2.427497 -0.009113
C -0.883046 -1.552358 1.019215
O -1.385996 -1.177234 2.071310
O -2.908066 -1.799540 -0.302335
C -1.824152 -3.816953 0.550636
C -2.982577 -4.388020 0.851416
C 1.083880 1.000901 1.304527
C 0.903052 1.758211 -0.010979
C -0.307401 2.701078 -0.007671
C -1.622752 1.937526 -0.034906

C -2.861767 2.829016 0.004705
C -4.159216 2.023042 -0.029431
C -5.416108 2.890187 -0.004213
C -6.712605 2.082840 -0.056185
C -7.964855 2.957388 -0.030861
O 4.133312 0.292364 1.982881
O 6.402286 0.210168 0.636816
O 2.091813 2.493132 -0.373664
O -0.250481 3.544935 -1.163466
H 6.133969 -0.968829 -1.758703
H 5.450456 -2.012145 -0.509184
H 3.920132 -0.353531 -2.579353
H 3.961156 -2.103160 -2.460782
H 5.035210 0.999518 -0.697048
H 1.732817 -0.864956 2.119140
H 1.659493 -2.075733 -2.442301
H -1.230644 -1.817776 -2.044561
H -0.578811 -3.414610 -1.667011
H -3.283071 -1.501469 0.539028
H -0.890217 -4.345287 0.720788
H -3.021954 -5.388313 1.263179
H -3.924485 -3.880929 0.684475
H 1.824073 1.518331 1.917240
H 0.156018 0.999769 1.875879
H 0.766229 1.061422 -0.840446
H -0.261178 3.325180 0.897906
H -1.640008 1.320667 -0.939523
H -1.655577 1.250525 0.812740
H -2.837601 3.441639 0.913026
H -2.840083 3.522884 -0.839580
H -4.170019 1.397161 -0.928950
H -4.179020 1.332965 0.821962
H -5.412181 3.508015 0.901244
H -5.391620 3.587017 -0.850067
H -6.714666 1.466396 -0.961416
H -6.735670 1.385872 0.788424
H -7.983970 3.639356 -0.885123
H -8.875244 2.354922 -0.064893
H -8.000156 3.564494 0.877669
H 6.110160 0.460571 1.530144
H 2.409041 2.965263 0.407064
H 0.688971 3.742761 -1.293102

2d-c58, ΔG = 1.3818 kcal/mol, population = 1.91 %

C 3.863250 0.111297 -1.786131
C 3.005416 -1.069247 -2.234788
C 1.884223 -1.348615 -1.278631
C 1.361704 -0.427098 -0.424486
C 1.950535 0.897066 -0.373018
C 2.968481 1.287978 -1.440129
C 0.186682 -0.710609 0.501618
N 1.386521 -2.638903 -1.312026
C 0.417607 -3.034217 -0.451452
C -0.137320 -2.176221 0.446743
C -0.142783 -4.420758 -0.413327
C -1.070471 -4.390931 0.821751
C -1.136223 -2.881720 1.189177
O -1.973403 -2.443484 1.980196
O -2.366929 -4.847693 0.466903
C -0.469056 -5.147757 1.979711
C -1.088606 -6.096973 2.667457
C -1.057040 0.152028 0.132076
C -1.560448 1.030355 1.268519
C -2.836383 1.790534 0.891922
C -2.691544 2.734560 -0.296210
C -1.513497 3.706394 -0.227022
C -1.446601 4.626959 -1.445687
C -0.248616 5.580831 -1.439817
C 1.108819 4.880086 -1.527531
C 2.279968 5.856679 -1.608061
O 1.648861 1.728032 0.484166
O 3.728530 2.399832 -1.006493
O -1.813346 0.295653 2.478043
O -3.276713 2.547889 2.029166
H 4.562249 0.391236 -2.574427
H 4.444435 -0.162250 -0.902299
H 2.569419 -0.864628 -3.219314
H 3.618612 -1.965488 -2.346013
H 2.380822 1.563717 -2.330456
H 0.480882 -0.442521 1.519295
H 1.794130 -3.304184 -1.952716
H -0.734573 -4.622731 -1.307826
H 0.639969 -5.177282 -0.347057
H -2.979326 -4.518326 1.140609
H 0.542533 -4.849482 2.240158
H -0.600215 -6.595975 3.494536
H -2.094134 -6.412616 2.419195
H -1.863265 -0.503207 -0.210052

H -0.807756 0.791836 -0.714156
H -0.783788 1.751316 1.526460
H -3.604904 1.042346 0.650000
H -2.610547 2.131205 -1.204688
H -3.627575 3.294061 -0.388036
H -1.586600 4.309225 0.682919
H -0.581017 3.145284 -0.150947
H -1.410491 4.015043 -2.354959
H -2.371853 5.209600 -1.505775
H -0.341653 6.274634 -2.282272
H -0.278592 6.195458 -0.532402
H 1.243386 4.225864 -0.663245
H 1.116223 4.225480 -2.406354
H 3.233515 5.327803 -1.665403
H 2.198032 6.498537 -2.489373
H 2.309630 6.505620 -0.728512
H 3.201300 2.816635 -0.304291
H -2.003099 -0.641070 2.269546
H -3.168484 1.953576 2.785925

2d-c11, ΔG = 1.4834 kcal/mol, population = 1.61 %
C 5.141450 -1.178783 -1.114563
C 3.833974 -1.226972 -1.900889
C 2.631459 -1.118245 -1.011436
C 2.650064 -0.569279 0.233469
C 3.907429 -0.093644 0.771363
C 5.110762 0.001498 -0.160154
C 1.400940 -0.397848 1.087255
N 1.456679 -1.618143 -1.543986
C 0.322078 -1.666787 -0.800474
C 0.267717 -1.157215 0.457095
C -0.957621 -2.272293 -1.284338
C -1.861073 -2.236351 -0.032584
C -1.050151 -1.374128 0.986185
O -1.543955 -0.964756 2.029632
O -3.082117 -1.574975 -0.334728
C -2.061807 -3.613674 0.543937
C -3.237722 -4.144471 0.850843
C 1.022302 1.091302 1.275887
C 0.817982 1.846598 -0.035254
C -0.304588 2.890847 0.030115
C -1.679788 2.249252 0.126490
C -2.824321 3.256585 0.243243
C -4.171812 2.601953 0.559313

C -4.687671 1.660024 -0.528919
C -6.027034 1.013166 -0.177774
C -6.517247 0.038468 -1.247152
O 4.043620 0.262333 1.943071
O 6.305171 0.091363 0.593724
O 2.037494 2.474413 -0.486528
O -0.262965 3.698834 -1.152038
H 5.988164 -1.089191 -1.795375
H 5.268208 -2.098360 -0.538163
H 3.797423 -0.398589 -2.617846
H 3.772883 -2.146960 -2.485299
H 4.966832 0.925628 -0.741918
H 1.599768 -0.798065 2.085147
H 1.473845 -2.033092 -2.463845
H -1.399950 -1.662617 -2.074382
H -0.809146 -3.278414 -1.678619
H -3.455700 -1.267897 0.504036
H -1.145185 -4.169300 0.721368
H -3.308391 -5.138252 1.273994
H -4.163581 -3.610118 0.678759
H 1.798896 1.588053 1.860261
H 0.115098 1.121447 1.878008
H 0.566296 1.154112 -0.841367
H -0.135971 3.530743 0.909986
H -1.818188 1.611018 -0.750460
H -1.704733 1.591493 0.997632
H -2.583335 3.971516 1.037066
H -2.901802 3.835144 -0.681151
H -4.084744 2.046020 1.500359
H -4.917069 3.385039 0.734313
H -4.787564 2.215265 -1.469183
H -3.955963 0.869732 -0.715496
H -5.928547 0.486160 0.777626
H -6.777587 1.794795 -0.018422
H -6.647938 0.543800 -2.207839
H -5.798030 -0.771428 -1.393520
H -7.475122 -0.409355 -0.973259
H 6.024053 0.353947 1.487100
H 2.440793 2.930518 0.263468
H 0.677737 3.818793 -1.349697

2d-c188, ΔG = 1.4891 kcal/mol, population = 1.60 %
C 4.103231 0.020440 -1.197590
C 3.312186 -1.111495 -1.850367

C 2.038655 -1.406601 -1.115349
C 1.397924 -0.516720 -0.311459
C 1.995873 0.785025 -0.084030
C 3.183915 1.202579 -0.944182
C 0.062583 -0.802383 0.361341
N 1.526890 -2.680459 -1.291714
C 0.463607 -3.106538 -0.569953
C -0.222837 -2.274551 0.263432
C -0.041150 -4.514283 -0.595724
C -1.064109 -4.537795 0.561441
C -1.235648 -3.036574 0.924333
O -2.147590 -2.653013 1.660655
O -2.303304 -5.058583 0.105467
C -0.516985 -5.268631 1.762391
C -1.141222 -6.248075 2.401816
C -1.052148 0.082938 -0.267532
C -2.086922 0.627277 0.721905
C -2.658097 1.972507 0.255850
C -1.657783 3.103779 0.434378
C -2.034328 4.396966 -0.286456
C -0.952185 5.477288 -0.186427
C 0.346964 5.124751 -0.914129
C 1.402658 6.227147 -0.845260
C 2.689554 5.863782 -1.584662
O 1.570767 1.577805 0.756564
O 3.874337 2.275630 -0.332705
O -3.205603 -0.252181 0.879296
O -3.842184 2.285724 0.998927
H 4.931160 0.320575 -1.839999
H 4.520201 -0.314440 -0.244838
H 3.055036 -0.843777 -2.881548
H 3.919236 -2.016876 -1.905455
H 2.758481 1.534397 -1.904749
H 0.148689 -0.518070 1.413795
H 2.029108 -3.332146 -1.876987
H -0.552136 -4.725885 -1.536630
H 0.763605 -5.240350 -0.475409
H -2.982469 -4.772634 0.733391
H 0.455532 -4.922336 2.100240
H -0.694320 -6.724486 3.264858
H -2.108330 -6.611602 2.078105
H -1.574074 -0.477397 -1.046360
H -0.567603 0.921921 -0.765324
H -1.612424 0.781243 1.699668

H -2.920270 1.876565 -0.808372
H -1.552159 3.293208 1.507669
H -0.682298 2.764962 0.087611
H -2.221807 4.176847 -1.343912
H -2.973008 4.780290 0.121688
H -1.343496 6.413180 -0.598583
H -0.733767 5.675309 0.869364
H 0.773892 4.206794 -0.499488
H 0.118745 4.908433 -1.964679
H 0.988384 7.151900 -1.260920
H 1.631275 6.438957 0.204774
H 3.430149 6.663837 -1.517487
H 3.137464 4.956588 -1.170989
H 2.492121 5.680278 -2.644296
H 3.247554 2.654296 0.307197
H -2.884084 -1.090333 1.267762
H -4.283185 1.435321 1.144355

2d-c432, ΔG = 1.5211 kcal/mol, population = 1.51 %
C 4.808150 -1.250431 -1.174250
C 3.682072 -2.067128 -1.805610
C 2.428952 -2.019029 -0.983079
C 2.126732 -1.021316 -0.110190
C 3.086649 0.044459 0.104760
C 4.293547 0.134282 -0.823665
C 0.803680 -0.944218 0.638598
N 1.565530 -3.090940 -1.139094
C 0.526525 -3.264586 -0.288348
C 0.170969 -2.307980 0.616852
C -0.285078 -4.518678 -0.215191
C -1.070030 -4.342824 1.103901
C -0.851247 -2.846429 1.458013
O -1.514379 -2.288182 2.335692
O -2.451596 -4.583223 0.887825
C -0.481967 -5.191816 2.203892
C -1.175110 -6.016890 2.976150
C -0.074333 0.178161 0.015646
C -1.020896 0.879370 0.986459
C -1.531233 2.223544 0.450590
C -0.451878 3.295762 0.381924
C -0.957990 4.667268 -0.073338
C -1.521977 4.692916 -1.494128
C -1.932581 6.092754 -1.947169
C -2.515010 6.130847 -3.359734

C -2.926175 7.534451 -3.800537
O 2.957218 0.893307 0.986983
O 5.304784 0.928650 -0.233592
O -2.200755 0.110463 1.254331
O -2.576000 2.698415 1.309385
H 5.649999 -1.173958 -1.862510
H 5.160355 -1.739683 -0.263059
H 3.450828 -1.677900 -2.803732
H 3.992632 -3.104919 -1.938431
H 3.928582 0.619024 -1.743492
H 1.015032 -0.657345 1.672118
H 1.823679 -3.834565 -1.771623
H -0.986794 -4.574853 -1.049212
H 0.337350 -5.413671 -0.232107
H -2.932683 -4.167357 1.617787
H 0.588371 -5.072826 2.345896
H -0.690222 -6.590890 3.755245
H -2.241485 -6.154000 2.848292
H -0.649565 -0.224335 -0.821715
H 0.604567 0.924468 -0.395548
H -0.494020 1.070853 1.930539
H -1.946051 2.045571 -0.550675
H 0.002743 3.386808 1.373210
H 0.336008 2.965833 -0.299066
H -1.720230 5.021043 0.625372
H -0.126117 5.376081 -0.010308
H -0.774954 4.291049 -2.188742
H -2.390540 4.030622 -1.561144
H -2.669391 6.497404 -1.243562
H -1.063971 6.759543 -1.897051
H -1.778889 5.726729 -4.062866
H -3.382236 5.463666 -3.407817
H -3.341184 7.531577 -4.810823
H -3.683491 7.948976 -3.129822
H -2.070218 8.214476 -3.793349
H 4.856636 1.445644 0.457283
H -1.961134 -0.676070 1.783326
H -3.065477 1.902750 1.567582

2d-c24, ΔG = 1.5248 kcal/mol, population = 1.50 %
C 5.334309 -1.234563 -0.458771
C 4.198563 -1.304721 -1.476838
C 2.856966 -1.121504 -0.834403
C 2.658940 -0.468469 0.343630

C 3.804433 0.024094 1.076277
C 5.168879 0.010972 0.392981
C 1.276092 -0.224759 0.929354
N 1.788829 -1.667581 -1.519561
C 0.553928 -1.713744 -0.956557
C 0.288264 -1.111948 0.231182
C -0.609152 -2.430865 -1.565284
C -1.711228 -2.304087 -0.490555
C -1.076312 -1.372513 0.591467
O -1.719947 -0.940679 1.540398
O -2.842211 -1.644015 -1.049868
C -2.047190 -3.634852 0.126498
C -3.269472 -4.139145 0.228556
C 0.842476 1.264690 0.863911
C 0.501427 1.787643 -0.523685
C -0.134232 3.183572 -0.505816
C -1.460261 3.275610 0.242696
C -2.493715 2.210814 -0.125354
C -3.842389 2.435508 0.555597
C -4.816367 1.277549 0.343921
C -6.192340 1.509123 0.965431
C -7.138636 0.324803 0.776507
O 3.727115 0.479435 2.219766
O 6.197445 0.120548 1.358856
O 1.641177 1.813060 -1.408851
O -0.356179 3.604663 -1.859537
H 6.297859 -1.215391 -0.968480
H 5.312486 -2.113860 0.189427
H 4.322036 -0.517385 -2.229750
H 4.223022 -2.255932 -2.011446
H 5.182872 0.893653 -0.266859
H 1.309172 -0.477731 1.991908
H 1.963615 -2.157155 -2.385001
H -0.932300 -1.939547 -2.484807
H -0.365334 -3.466706 -1.805790
H -3.370269 -1.300768 -0.314653
H -1.193549 -4.179662 0.520110
H -3.440190 -5.099707 0.697371
H -4.133003 -3.615952 -0.162581
H 1.624992 1.882432 1.311837
H -0.039403 1.370452 1.495968
H -0.193762 1.109446 -1.017922
H 0.581502 3.872542 -0.034184
H -1.260827 3.234187 1.316684

H -1.870066 4.271537 0.049294
H -2.630394 2.187967 -1.209959
H -2.124653 1.225518 0.164444
H -3.685766 2.576982 1.631110
H -4.289800 3.364695 0.185425
H -4.931067 1.087515 -0.729400
H -4.380842 0.368980 0.776796
H -6.072356 1.715667 2.034185
H -6.637305 2.408664 0.527352
H -6.727979 -0.580116 1.232341
H -8.113823 0.514132 1.230297
H -7.298976 0.116846 -0.284736
H 5.761548 0.464609 2.157527
H 2.370101 2.257735 -0.957211
H 0.423796 3.316104 -2.355114

2d-c63, ΔG = 1.5292 kcal/mol, population = 1.49 %
C 5.389522 -0.683956 -0.616116
C 4.241414 -1.022878 -1.563290
C 2.929267 -1.137746 -0.846343
C 2.665783 -0.566598 0.360613
C 3.712840 0.177624 1.028386
C 4.989501 0.492230 0.256614
C 1.301837 -0.634553 1.032765
N 1.962225 -1.879186 -1.502165
C 0.771067 -2.146716 -0.908303
C 0.447717 -1.633841 0.306654
C -0.284301 -3.013903 -1.518831
C -1.329514 -3.145924 -0.389624
C -0.853487 -2.113423 0.679479
O -1.552213 -1.796462 1.634489
O -2.612213 -2.761676 -0.865987
C -1.310949 -4.524124 0.218422
C -2.377326 -5.292474 0.394670
C 0.600267 0.744380 1.076053
C 0.486642 1.423638 -0.287106
C -0.818398 2.211046 -0.463054
C -2.023023 1.291592 -0.620366
C -3.359496 2.019593 -0.784624
C -3.790407 2.823780 0.441893
C -5.174228 3.452629 0.289719
C -5.608672 4.270015 1.505645
C -6.995873 4.889616 1.346368
O 3.608779 0.591619 2.184133

O 6.024585 0.851133 1.152662
O 1.613416 2.289282 -0.544845
O -0.723687 3.027442 -1.635966
H 6.288105 -0.441678 -1.183821
H 5.615467 -1.540621 0.023329
H 4.141955 -0.240966 -2.324943
H 4.447089 -1.953300 -2.095870
H 4.743306 1.348433 -0.391032
H 1.435585 -0.951661 2.070437
H 2.187227 -2.297636 -2.392714
H -0.737326 -2.521435 -2.381210
H 0.115174 -3.973527 -1.849433
H -3.143383 -2.527028 -0.091135
H -0.329414 -4.864959 0.536060
H -2.291241 -6.270878 0.849621
H -3.363766 -4.975074 0.080538
H 1.140680 1.403229 1.758384
H -0.387943 0.594847 1.510144
H 0.533265 0.686739 -1.091823
H -0.949035 2.858085 0.415553
H -1.843547 0.654047 -1.491563
H -2.083463 0.629467 0.245859
H -3.309810 2.679854 -1.654449
H -4.129125 1.273169 -1.005511
H -3.784385 2.170658 1.322377
H -3.062108 3.615680 0.642895
H -5.183558 4.095264 -0.598325
H -5.911998 2.663511 0.103721
H -5.593903 3.628273 2.393058
H -4.873094 5.060700 1.687806
H -7.280157 5.468126 2.228074
H -7.028944 5.559231 0.482792
H -7.754723 4.117304 1.195072
H 5.574563 1.076341 1.985042
H 1.784137 2.812235 0.249180
H 0.193358 3.338688 -1.660102

2d-c439, ΔG = 1.5901 kcal/mol, population = 1.34 %
C 4.623663 -0.677462 1.794394
C 4.360267 -1.703829 0.693567
C 2.964644 -1.595194 0.156240
C 2.220313 -0.452771 0.207041
C 2.764106 0.716791 0.850302
C 4.217854 0.701257 1.307454

C 0.833699 -0.339815 -0.406594
N 2.438558 -2.741158 -0.399644
C 1.123563 -2.806051 -0.748412
C 0.315479 -1.717060 -0.697989
C 0.445681 -4.067094 -1.182278
C -1.047104 -3.671007 -1.205566
C -1.025621 -2.120605 -1.023452
O -2.029484 -1.434141 -1.163483
O -1.612689 -3.984711 -2.470914
C -1.796226 -4.285987 -0.051739
C -2.922556 -4.977495 -0.160186
C 0.890050 0.550560 -1.675397
C -0.391588 1.308079 -2.033906
C -0.900385 2.196887 -0.890161
C -2.158160 2.966084 -1.270790
C -2.745678 3.813624 -0.140510
C -3.214160 3.017153 1.077654
C -3.884769 3.890113 2.136786
C -4.351826 3.111512 3.366161
C -5.021844 3.996546 4.415633
O 2.111985 1.755537 1.026764
O 4.430285 1.680970 2.306456
O -0.144276 2.091578 -3.212497
O 0.114394 3.138047 -0.507883
H 5.678640 -0.681489 2.068373
H 4.042034 -0.925153 2.685408
H 5.059016 -1.550349 -0.136516
H 4.531953 -2.715362 1.065161
H 4.810806 0.948014 0.411613
H 0.185996 0.137147 0.330236
H 2.998046 -3.581674 -0.407304
H 0.764590 -4.353414 -2.186157
H 0.658370 -4.899137 -0.510075
H -2.420539 -3.458856 -2.560410
H -1.352348 -4.108273 0.923852
H -3.415773 -5.382079 0.714267
H -3.379429 -5.170911 -1.122610
H 1.178956 -0.066613 -2.527987
H 1.682801 1.290855 -1.547796
H -1.182444 0.607212 -2.298891
H -1.135611 1.550895 -0.041023
H -1.930560 3.605413 -2.126571
H -2.903212 2.239562 -1.610881
H -2.005006 4.553297 0.175111

H -3.594919 4.377527 -0.540154
H -3.913408 2.236777 0.754879
H -2.365115 2.498276 1.533866
H -3.186947 4.674854 2.451465
H -4.742715 4.405880 1.689993
H -5.047525 2.326416 3.051091
H -3.493919 2.598329 3.813586
H -5.901817 4.496464 4.002197
H -5.344770 3.416013 5.282601
H -4.336814 4.771731 4.769196
H 3.716269 2.329936 2.192826
H 0.407950 2.835417 -2.933753
H 0.777795 2.670806 0.030055

2d-c281, ΔG = 1.6547 kcal/mol, population = 1.21 %
C 3.816934 0.396285 -1.970973
C 3.089484 -0.895897 -2.335347
C 2.059275 -1.276528 -1.313164
C 1.481801 -0.399078 -0.448255
C 1.914063 0.984173 -0.461074
C 2.798308 1.462865 -1.607517
C 0.399264 -0.788384 0.549392
N 1.709287 -2.614738 -1.295901
C 0.830547 -3.093074 -0.382058
C 0.228733 -2.280134 0.527807
C 0.425524 -4.530498 -0.292632
C -0.450110 -4.571793 0.980050
C -0.654762 -3.070405 1.328412
O -1.493948 -2.704184 2.153227
O -1.706276 -5.163522 0.683458
C 0.268917 -5.243484 2.123193
C -0.220996 -6.242950 2.843534
C -0.945095 -0.061445 0.245339
C -1.458728 0.791835 1.395471
C -2.793483 1.469321 1.065741
C -2.737311 2.448998 -0.102585
C -1.627914 3.498607 -0.027081
C -1.626688 4.446941 -1.224974
C -0.464002 5.439503 -1.188534
C -0.355510 6.336418 -2.425282
C -1.537364 7.288752 -2.612110
O 1.581670 1.796493 0.402665
O 3.419680 2.686389 -1.263345
O -1.619695 0.046055 2.614644

O -3.255755 2.170703 2.229603
H 4.430334 0.732185 -2.807137
H 4.475548 0.228783 -1.115421
H 2.582699 -0.781488 -3.300378
H 3.802055 -1.714433 -2.453093
H 2.125489 1.618324 -2.465675
H 0.722549 -0.475507 1.545555
H 2.152947 -3.245162 -1.947765
H -0.175841 -4.817234 -1.157076
H 1.288050 -5.196286 -0.244045
H -2.321179 -4.893276 1.380753
H 1.251368 -4.835906 2.343943
H 0.346852 -6.674693 3.657590
H -1.195093 -6.667485 2.635994
H -1.703786 -0.799875 -0.028969
H -0.813157 0.575410 -0.629037
H -0.720782 1.562523 1.619629
H -3.515760 0.676858 0.822901
H -2.632142 1.874555 -1.027026
H -3.711149 2.944265 -0.163956
H -1.732235 4.074525 0.897028
H -0.654004 3.006382 0.024449
H -1.564525 3.859539 -2.149104
H -2.579672 4.983090 -1.265584
H -0.553133 6.067139 -0.294057
H 0.469355 4.877530 -1.076471
H 0.566276 6.921802 -2.355357
H -0.250835 5.706168 -3.315247
H -2.470116 6.747515 -2.781123
H -1.674129 7.917094 -1.727586
H -1.380085 7.948371 -3.468336
H 2.913006 3.026297 -0.505475
H -1.707586 -0.908027 2.418587
H -3.080210 1.572362 2.970599

2d-c253, ΔG = 1.7514 kcal/mol, population = 1.02 %
C 4.043783 0.029664 1.236635
C 3.805213 -0.790277 -0.030772
C 2.357145 -1.138082 -0.209296
C 1.333668 -0.427739 0.335239
C 1.629267 0.687879 1.211997
C 3.065962 1.191010 1.289345
C -0.128448 -0.734273 0.056979
N 2.093580 -2.270459 -0.963214

C 0.844638 -2.796176 -0.995965
C -0.220464 -2.139450 -0.457618
C 0.515823 -4.140847 -1.562796
C -0.935203 -4.378054 -1.081628
C -1.361163 -2.995993 -0.511563
O -2.524498 -2.750937 -0.186374
O -1.761243 -4.708108 -2.188909
C -0.984175 -5.400995 0.024984
C -1.756048 -6.478970 0.028885
C -0.716191 0.324421 -0.926893
C -2.106358 0.828296 -0.558324
C -2.548061 2.031479 -1.395094
C -1.651230 3.260874 -1.325959
C -1.310523 3.773683 0.073308
C -0.552623 5.102035 0.020108
C -0.115355 5.638459 1.386062
C 0.979779 4.809969 2.059295
C 1.441954 5.400278 3.390137
O 0.765130 1.256219 1.879013
O 3.250896 1.967456 2.458005
O -3.139049 -0.143457 -0.772972
O -3.870014 2.418613 -0.985701
H 5.068089 0.402056 1.256059
H 3.895817 -0.595802 2.120176
H 4.131379 -0.224338 -0.910803
H 4.400778 -1.704564 -0.008421
H 3.211943 1.821439 0.397749
H -0.675739 -0.651898 0.999569
H 2.870033 -2.798687 -1.335059
H 0.537109 -4.117900 -2.653738
H 1.211538 -4.909831 -1.225936
H -2.679862 -4.580445 -1.912178
H -0.331276 -5.189228 0.867011
H -1.746519 -7.168174 0.863444
H -2.410519 -6.713705 -0.801232
H -0.724551 -0.079428 -1.942634
H -0.039955 1.179437 -0.934261
H -2.101943 1.121856 0.498320
H -2.589232 1.694587 -2.439965
H -0.727443 3.049556 -1.871158
H -2.157105 4.050559 -1.890206
H -2.230284 3.905067 0.650554
H -0.712435 3.029868 0.603165
H 0.333080 4.985777 -0.615996

H -1.185856 5.846760 -0.473318
H 0.245689 6.665649 1.266140
H -0.986317 5.696121 2.049668
H 0.625036 3.789619 2.217722
H 1.834075 4.732329 1.377552
H 2.233710 4.798109 3.841840
H 1.829685 6.414024 3.258111
H 0.615377 5.452647 4.103821
H 2.358714 2.235564 2.735258
H -2.911278 -1.005539 -0.372360
H -4.358387 1.591240 -0.871356

2d-c3, ΔG = 1.7696 kcal/mol, population = 0.99 %
C 5.180784 -1.235074 -0.778206
C 3.959991 -1.244332 -1.694849
C 2.677084 -1.103722 -0.930676
C 2.584521 -0.557004 0.312366
C 3.793268 -0.115006 0.976561
C 5.086340 -0.053942 0.170629
C 1.260073 -0.351010 1.034004
N 1.548907 -1.572912 -1.579933
C 0.345659 -1.598424 -0.951558
C 0.179803 -1.089778 0.296703
C -0.894127 -2.176208 -1.558398
C -1.913651 -2.123279 -0.400165
C -1.184247 -1.288449 0.698957
O -1.765771 -0.878620 1.696483
O -3.080343 -1.424954 -0.815428
C -2.210789 -3.495668 0.144362
C -3.426905 -3.986643 0.341204
C 0.907437 1.149728 1.171255
C 0.892901 1.907659 -0.157843
C -0.278515 2.891696 -0.280432
C -1.604263 2.164316 -0.435419
C -2.824135 3.082192 -0.504813
C -4.140686 2.307315 -0.627472
C -4.531202 1.530690 0.630741
C -5.787893 0.678942 0.448689
C -6.158394 -0.117528 1.698780
O 3.819960 0.236216 2.156956
O 6.200764 0.003736 1.041071
O 2.138006 2.597734 -0.396814
O -0.077821 3.727545 -1.425526
H 6.095090 -1.169620 -1.368266

H 5.221419 -2.158110 -0.194897
H 4.020608 -0.412267 -2.405970
H 3.932525 -2.160148 -2.288092
H 5.026961 0.873007 -0.421296
H 1.346428 -0.746766 2.049423
H 1.648535 -1.987495 -2.494856
H -1.245250 -1.553347 -2.383010
H -0.730680 -3.184006 -1.942262
H -3.521789 -1.106218 -0.015497
H -1.332816 -4.082151 0.400361
H -3.569081 -4.978604 0.750374
H -4.314865 -3.420520 0.089433
H 1.626230 1.620989 1.843579
H -0.063489 1.217914 1.661343
H 0.816935 1.213186 -0.996909
H -0.304353 3.518309 0.624223
H -1.554819 1.552938 -1.342679
H -1.718868 1.474360 0.399814
H -2.855134 3.714233 0.390072
H -2.717664 3.755055 -1.358338
H -4.947982 3.003887 -0.874798
H -4.066926 1.611698 -1.471240
H -3.705147 0.886414 0.947075
H -4.689052 2.235989 1.454560
H -6.623953 1.325758 0.163637
H -5.639505 -0.006687 -0.393214
H -6.340876 0.548059 2.546336
H -7.059653 -0.713558 1.541040
H -5.353709 -0.800303 1.984906
H 5.838593 0.273526 1.902506
H 2.391493 3.059894 0.412566
H 0.875327 3.897306 -1.458478

2d-c556, ΔG = 1.7809 kcal/mol, population = 0.97 %
C 4.684935 -0.717202 -1.898944
C 3.481725 -1.533903 -2.364083
C 2.360468 -1.503251 -1.367785
C 2.187468 -0.518612 -0.444563
C 3.167353 0.546510 -0.362043
C 4.219588 0.660005 -1.460898
C 1.023216 -0.475829 0.534715
N 1.478398 -2.567223 -1.434176
C 0.467511 -2.689742 -0.541000
C 0.246322 -1.757916 0.425885

C -0.498409 -3.832044 -0.535913
C -1.332115 -3.583242 0.740662
C -0.894912 -2.160440 1.188434
O -1.515009 -1.535706 2.051654
O -2.715290 -3.571925 0.419592
C -0.982001 -4.566227 1.829588
C -1.859085 -5.308455 2.491113
C 0.104129 0.758969 0.289393
C -0.081274 1.654749 1.510750
C -1.168138 2.715755 1.290693
C -0.795278 3.759875 0.250425
C -1.867260 4.832725 0.024542
C -3.237471 4.298348 -0.406196
C -3.220103 3.477026 -1.695210
C -4.611323 3.031278 -2.143776
C -4.586993 2.191455 -3.419580
O 3.176736 1.374609 0.549335
O 5.301257 1.457208 -1.017611
O -0.407844 0.914919 2.697971
O -1.423531 3.382617 2.533747
H 5.414662 -0.628251 -2.703891
H 5.171676 -1.212477 -1.055354
H 3.104555 -1.138244 -3.314101
H 3.772602 -2.569194 -2.550652
H 3.718697 1.151385 -2.310159
H 1.431417 -0.385364 1.543914
H 1.627448 -3.285800 -2.127696
H -1.150246 -3.792585 -1.410407
H 0.011101 -4.796299 -0.537019
H -3.172510 -3.117528 1.141886
H 0.077100 -4.625771 2.063567
H -1.539002 -5.989704 3.268938
H -2.918223 -5.268588 2.269799
H -0.872909 0.412408 -0.059240
H 0.519982 1.357656 -0.520446
H 0.862610 2.159013 1.731757
H -2.080171 2.188544 0.977782
H 0.136569 4.240345 0.564378
H -0.583230 3.254023 -0.692875
H -1.986055 5.415640 0.940580
H -1.500083 5.525647 -0.739400
H -3.668443 3.694527 0.398776
H -3.914219 5.148943 -0.538054
H -2.752576 4.065413 -2.493720

H -2.593332 2.589344 -1.564153
H -5.079788 2.457659 -1.336839
H -5.241027 3.914067 -2.297067
H -5.592173 1.885391 -3.717551
H -4.148983 2.752387 -4.249501
H -3.989699 1.286381 -3.280388
H 4.960352 1.948948 -0.250853
H -0.905517 0.104845 2.460424
H -1.393574 2.678335 3.199100

2d-c50, ΔG = 1.8010 kcal/mol, population = 0.94 %
C 5.261529 -0.947189 -1.161759
C 3.947376 -1.050272 -1.932004
C 2.757159 -1.066510 -1.019785
C 2.760255 -0.568023 0.246759
C 3.988435 -0.021440 0.784253
C 5.168786 0.188403 -0.158173
C 1.519067 -0.526497 1.126479
N 1.612101 -1.633763 -1.550455
C 0.507210 -1.815222 -0.782377
C 0.446530 -1.369665 0.499022
C -0.730934 -2.506046 -1.260145
C -1.587670 -2.619870 0.019570
C -0.826499 -1.733901 1.054785
O -1.321286 -1.420164 2.130561
O -2.877898 -2.068010 -0.204444
C -1.631584 -4.037488 0.528719
C -2.737378 -4.693309 0.853831
C 1.012340 0.919376 1.348992
C 0.750660 1.687250 0.053352
C -0.511965 2.558056 0.105569
C -1.781030 1.719460 0.098734
C -3.067148 2.536339 0.198256
C -4.312239 1.647925 0.210429
C -5.630626 2.408018 0.374904
C -5.979708 3.332332 -0.792936
C -7.346650 3.995743 -0.632893
O 4.116468 0.303614 1.965733
O 6.366136 0.324587 0.584482
O 1.884247 2.495718 -0.327964
O -0.534442 3.422933 -1.035798
H 6.089364 -0.773459 -1.849499
H 5.457544 -1.879456 -0.626656
H 3.842014 -0.195236 -2.609812

H 3.939691 -1.945713 -2.556229
H 4.958729 1.124267 -0.699656
H 1.772044 -0.926152 2.111893
H 1.639275 -2.004975 -2.488699
H -1.253356 -1.892493 -1.996413
H -0.511132 -3.470832 -1.718986
H -3.232662 -1.815105 0.660316
H -0.659689 -4.510388 0.639330
H -2.695347 -5.708791 1.226333
H -3.715865 -4.242299 0.746849
H 1.743212 1.465531 1.948131
H 0.104170 0.860559 1.948167
H 0.629636 0.998613 -0.785126
H -0.478148 3.168513 1.020896
H -1.791271 1.122851 -0.819606
H -1.746590 1.012885 0.930086
H -3.044733 3.137043 1.114932
H -3.112721 3.239968 -0.634584
H -4.345837 1.062605 -0.715590
H -4.216325 0.922504 1.025280
H -6.442932 1.684252 0.501773
H -5.596952 2.994453 1.300748
H -5.214248 4.106148 -0.896471
H -5.959095 2.753865 -1.723129
H -8.140505 3.246977 -0.566419
H -7.384534 4.599152 0.278073
H -7.575336 4.651089 -1.476190
H 6.082168 0.535564 1.490479
H 2.199292 2.967138 0.453899
H 0.388601 3.676027 -1.185679

2d-c367, ΔG = 1.8675 kcal/mol, population = 0.84 %
C 4.259467 0.382483 -1.132241
C 3.640009 -0.892084 -1.700749
C 2.373548 -1.275072 -0.993360
C 1.602695 -0.408123 -0.282247
C 2.041545 0.967307 -0.146254
C 3.190210 1.455383 -1.021622
C 0.300192 -0.802841 0.401049
N 2.013946 -2.606275 -1.106735
C 0.918385 -3.088252 -0.472440
C 0.111578 -2.288078 0.275731
C 0.483979 -4.517800 -0.542175
C -0.711759 -4.573495 0.434309

C -0.964389 -3.080189 0.787396
O -1.980033 -2.719965 1.385400
O -1.851622 -5.095678 -0.234021
C -0.366060 -5.323908 1.695458
C -1.070852 -6.328605 2.197368
C -0.917777 -0.026370 -0.183869
C -1.676667 0.787802 0.854078
C -2.896927 1.498808 0.260129
C -2.569346 2.531987 -0.813883
C -1.476222 3.534862 -0.442494
C -1.262779 4.594493 -1.522606
C -0.032473 5.477697 -1.286734
C -0.023801 6.244542 0.040879
C -1.212074 7.188552 0.220572
O 1.513604 1.764307 0.629730
O 3.712697 2.665930 -0.508974
O -2.112667 -0.002980 1.973045
O -3.608370 2.155773 1.320343
H 5.071114 0.725846 -1.773794
H 4.672922 0.190039 -0.139309
H 3.406468 -0.753937 -2.762676
H 4.349735 -1.719539 -1.643289
H 2.759277 1.631068 -2.019915
H 0.375875 -0.538011 1.458903
H 2.604571 -3.229951 -1.637357
H 0.146006 -4.773050 -1.547960
H 1.290425 -5.201179 -0.273363
H -2.629253 -4.838472 0.282047
H 0.526326 -4.970272 2.203994
H -0.769291 -6.817916 3.114541
H -1.960203 -6.700767 1.704683
H -1.600181 -0.730315 -0.668171
H -0.571394 0.646903 -0.967747
H -1.004890 1.538487 1.270920
H -3.550191 0.730615 -0.177747
H -2.286083 2.004747 -1.729230
H -3.495914 3.065791 -1.046741
H -1.735500 4.010794 0.506015
H -0.531222 3.011743 -0.275645
H -1.148432 4.095843 -2.491239
H -2.160486 5.215005 -1.608283
H 0.863917 4.849656 -1.332848
H 0.051320 6.195401 -2.110195
H 0.006707 5.535880 0.873619

H 0.904931 6.819906 0.101194
H -1.277384 7.898771 -0.608544
H -2.156931 6.642994 0.263129
H -1.122233 7.762365 1.145464
H 3.033042 3.000829 0.101442
H -2.189533 -0.942486 1.710700
H -3.600440 1.526049 2.056174

2d-c179, ΔG = 1.8813 kcal/mol, population = 0.82 %
C 4.021313 0.159879 -1.421086
C 3.264165 -1.058137 -1.947141
C 2.049485 -1.374591 -1.125192
C 1.412058 -0.475785 -0.327045
C 1.956328 0.862261 -0.205138
C 3.044428 1.297566 -1.179806
C 0.147698 -0.796107 0.458266
N 1.588767 -2.675477 -1.227506
C 0.529694 -3.099220 -0.495778
C -0.143353 -2.263541 0.340059
C -0.010348 -4.493955 -0.543212
C -1.089209 -4.492629 0.563846
C -1.214771 -2.990870 0.947041
O -2.143023 -2.571448 1.640595
O -2.325332 -4.949373 0.034955
C -0.637481 -5.269489 1.775206
C -1.329208 -6.242854 2.351387
C -1.062008 0.056638 -0.030173
C -1.667442 0.933442 1.055183
C -2.862204 1.750324 0.556346
C -2.546043 2.706250 -0.589266
C -1.304962 3.578738 -0.397537
C -1.073000 4.539295 -1.565625
C 0.333448 5.144509 -1.587870
C 0.689998 5.973742 -0.353868
C 2.083835 6.592510 -0.445928
O 1.562416 1.670957 0.635709
O 3.697532 2.455220 -0.695166
O -2.083510 0.179840 2.208123
O -3.390592 2.510161 1.654603
H 4.785001 0.466023 -2.136090
H 4.519620 -0.084504 -0.479924
H 2.936918 -0.879343 -2.977824
H 3.918869 -1.931240 -1.975764
H 2.529711 1.531118 -2.125168

H 0.322267 -0.545654 1.507643
H 2.080257 -3.322674 -1.826421
H -0.479474 -4.694723 -1.507945
H 0.768791 -5.240338 -0.384284
H -3.021614 -4.646287 0.635489
H 0.324976 -4.965231 2.176676
H -0.949237 -6.755528 3.225644
H -2.286957 -6.564870 1.962304
H -1.832086 -0.603382 -0.439155
H -0.738957 0.690589 -0.855254
H -0.902879 1.621524 1.417146
H -3.631493 1.041763 0.218006
H -2.435384 2.118609 -1.504907
H -3.426072 3.338897 -0.740986
H -1.393565 4.135425 0.537864
H -0.420020 2.946690 -0.293523
H -1.233732 3.999190 -2.504986
H -1.820807 5.339976 -1.541073
H 1.065140 4.334522 -1.692704
H 0.440874 5.772105 -2.479146
H -0.057669 6.763819 -0.223154
H 0.633403 5.346520 0.539764
H 2.163093 7.255248 -1.311794
H 2.324084 7.176319 0.445333
H 2.846955 5.816774 -0.552669
H 3.105612 2.817155 -0.013103
H -2.235017 -0.753800 1.961475
H -3.399699 1.899480 2.405762

2d-c371, ΔG = 1.9396 kcal/mol, population = 0.75 %
C 5.348854 -0.763161 0.852594
C 4.787407 -1.744576 -0.174917
C 3.296336 -1.636346 -0.287687
C 2.591000 -0.511949 0.027981
C 3.291392 0.637933 0.542240
C 4.815320 0.627856 0.564442
C 1.085598 -0.398592 -0.158896
N 2.640185 -2.766427 -0.724336
C 1.281336 -2.843036 -0.680639
C 0.514367 -1.773746 -0.348103
C 0.516905 -4.098708 -0.957257
C -0.916599 -3.737572 -0.510526
C -0.860181 -2.193247 -0.289647
O -1.869473 -1.524131 -0.111846

O -1.840226 -4.030486 -1.548977
C -1.264683 -4.399814 0.798102
C -2.359418 -5.114730 1.019169
C 0.772135 0.540071 -1.353840
C -0.564810 1.283833 -1.302698
C -0.731788 2.142827 -0.040141
C -2.007381 2.977092 -0.032735
C -3.296127 2.157634 -0.101998
C -4.568862 3.008388 -0.046262
C -4.796089 3.730784 1.281773
C -6.118364 4.495634 1.331730
C -6.340879 5.219844 2.658230
O 2.713067 1.656799 0.945546
O 5.300287 1.568015 1.504471
O -0.685616 2.092962 -2.483513
O 0.370577 3.055109 0.087518
H 6.438213 -0.760995 0.816111
H 5.044638 -1.059105 1.859280
H 5.220900 -1.543728 -1.161241
H 5.060386 -2.768650 0.084980
H 5.126860 0.922174 -0.451192
H 0.672206 0.039338 0.751002
H 3.179789 -3.594534 -0.930061
H 0.520026 -4.326676 -2.024766
H 0.930185 -4.956990 -0.426200
H -2.641415 -3.517324 -1.369799
H -0.544632 -4.236794 1.595181
H -2.553367 -5.553419 1.989513
H -3.087569 -5.293905 0.238096
H 0.807453 -0.041502 -2.276781
H 1.564902 1.287298 -1.429666
H -1.384460 0.569241 -1.344951
H -0.758056 1.474510 0.826603
H -1.990583 3.572169 0.882426
H -1.975572 3.679344 -0.870028
H -3.307942 1.577671 -1.027851
H -3.312785 1.430351 0.717278
H -4.546054 3.743877 -0.858735
H -5.430153 2.361763 -0.243954
H -4.771407 2.998422 2.097443
H -3.976803 4.430420 1.473480
H -6.144703 5.219758 0.510403
H -6.944081 3.798973 1.151754
H -5.545896 5.946460 2.846129

H -7.292142 5.756472 2.667330
H -6.347795 4.514122 3.493194
H 4.580968 2.209007 1.629225
H -0.071341 2.832051 -2.375601
H 1.149844 2.560512 0.398416

2d-c98, ΔG = 1.9428 kcal/mol, population = 0.74 %
C 4.535105 -1.490124 -2.102471
C 3.105663 -1.265049 -2.592158
C 2.175021 -0.925504 -1.466442
C 2.573709 -0.383530 -0.283875
C 3.988215 -0.176740 -0.039874
C 4.955771 -0.336755 -1.208511
C 1.579130 0.059685 0.780581
N 0.837418 -1.227882 -1.681154
C -0.033192 -1.233804 -0.642110
C 0.307963 -0.707443 0.566711
C -1.389431 -1.862498 -0.677889
C -1.796731 -1.896796 0.815279
C -0.714240 -1.024496 1.510214
O -0.806852 -0.676063 2.689358
O -3.069139 -1.288301 0.982311
C -1.729319 -3.298447 1.366871
C -2.713631 -3.905722 2.014878
C 1.442589 1.602122 0.688864
C 0.456545 2.302058 1.637065
C -1.000706 2.344570 1.144132
C -1.197309 3.186637 -0.108424
C -2.606621 3.093216 -0.700736
C -2.948277 1.704842 -1.243191
C -4.301280 1.640883 -1.947914
C -4.639267 0.243863 -2.468529
C -5.990544 0.177501 -3.177415
O 4.439968 0.141779 1.059717
O 6.275532 -0.509693 -0.728848
O 0.503926 1.772670 2.971030
O -1.823905 2.899786 2.180739
H 5.214855 -1.572532 -2.950705
H 4.593803 -2.420346 -1.532283
H 3.081661 -0.441092 -3.314498
H 2.740122 -2.150166 -3.115594
H 4.889155 0.600414 -1.783954
H 1.986502 -0.181611 1.763769
H 0.576388 -1.665677 -2.553277

H -2.091786 -1.238151 -1.231730
H -1.373914 -2.849145 -1.140956
H -3.164641 -1.068470 1.920010
H -0.780446 -3.803183 1.209005
H -2.594048 -4.912762 2.393074
H -3.669471 -3.424039 2.178271
H 1.194773 1.864358 -0.340182
H 2.437082 2.007868 0.880278
H 0.783295 3.339581 1.744547
H -1.333092 1.323738 0.945485
H -0.961895 4.225012 0.143408
H -0.481540 2.873514 -0.871483
H -3.342151 3.383483 0.054556
H -2.690055 3.823309 -1.512034
H -2.162514 1.390796 -1.941254
H -2.945704 0.975478 -0.429686
H -5.086124 1.966347 -1.255335
H -4.314438 2.351875 -2.782114
H -3.849806 -0.082575 -3.154456
H -4.628096 -0.461206 -1.631077
H -6.205644 -0.831246 -3.536780
H -6.800214 0.471519 -2.504231
H -6.015209 0.850855 -4.038280
H 6.259735 -0.192096 0.190094
H 0.151355 0.858555 2.960963
H -1.445066 2.579506 3.012561

2d-c293, ΔG = 1.9974 kcal/mol, population = 0.68 %
C 5.445752 -1.224566 0.337308
C 4.639489 -2.037352 -0.673762
C 3.171784 -1.747789 -0.574095
C 2.660257 -0.579923 -0.087954
C 3.560158 0.428838 0.412074
C 5.061360 0.239252 0.227569
C 1.170079 -0.273891 -0.062550
N 2.328986 -2.750964 -0.998910
C 0.984871 -2.665172 -0.797397
C 0.406029 -1.545021 -0.295418
C 0.036313 -3.787173 -1.079142
C -1.286504 -3.290410 -0.456299
C -0.999256 -1.794447 -0.114585
O -1.886401 -1.025400 0.231296
O -2.332567 -3.358033 -1.415391
C -1.602778 -4.026166 0.820096

C -2.763125 -4.606696 1.094251
C 0.838285 0.816718 -1.114638
C -0.371586 1.704680 -0.810609
C -0.254343 2.445011 0.530414
C -1.420041 3.385481 0.808807
C -2.774360 2.685271 0.905100
C -3.900751 3.638552 1.306146
C -5.256759 2.956679 1.506846
C -5.838104 2.314949 0.245893
C -7.230030 1.726704 0.471258
O 3.171715 1.467640 0.964675
O 5.775856 1.028497 1.159888
O -0.538548 2.641660 -1.886345
O 0.949339 3.229239 0.554630
H 6.513201 -1.345683 0.153049
H 5.235927 -1.572222 1.351523
H 4.966173 -1.798309 -1.692174
H 4.812636 -3.105370 -0.531915
H 5.277703 0.585186 -0.796485
H 0.928453 0.110617 0.929566
H 2.729688 -3.615677 -1.332002
H -0.093535 -3.924317 -2.154215
H 0.381695 -4.731843 -0.657291
H -3.034201 -2.763505 -1.112748
H -0.796078 -4.039841 1.547580
H -2.926896 -5.108518 2.039234
H -3.578402 -4.609684 0.381823
H 0.680484 0.336593 -2.082041
H 1.707077 1.467789 -1.231798
H -1.275590 1.099749 -0.795139
H -0.222366 1.701234 1.332891
H -1.206551 3.897169 1.751520
H -1.452673 4.151885 0.030290
H -3.014395 2.218427 -0.052001
H -2.714270 1.871979 1.636970
H -3.619277 4.147679 2.233871
H -3.998457 4.421196 0.544844
H -5.163860 2.192663 2.287771
H -5.971941 3.694796 1.885588
H -5.880728 3.066332 -0.550262
H -5.169278 1.527371 -0.111379
H -7.935370 2.497990 0.792068
H -7.624779 1.271022 -0.439479
H -7.207372 0.956347 1.246766

H 5.165587 1.735997 1.426148
H 0.170986 3.292688 -1.794720
H 1.701379 2.629816 0.707483

2d-c75, ΔG = 2.0758 kcal/mol, population = 0.59 %
C 3.948322 0.131393 -1.744967
C 3.044438 -1.019346 -2.181320
C 1.924311 -1.260767 -1.212486
C 1.457864 -0.330556 -0.335368
C 2.102211 0.967424 -0.285024
C 3.091047 1.331886 -1.386396
C 0.296281 -0.577774 0.618338
N 1.367712 -2.526226 -1.258015
C 0.386919 -2.887031 -0.396301
C -0.116150 -2.019897 0.523742
C -0.241201 -4.244668 -0.379219
C -1.172160 -4.188358 0.851738
C -1.146191 -2.688076 1.258393
O -1.949914 -2.225317 2.070083
O -2.492622 -4.550664 0.474271
C -0.632405 -5.013795 1.992498
C -1.316684 -5.942687 2.645789
C -0.901516 0.376244 0.328721
C -1.303563 1.234882 1.518987
C -2.503757 2.136367 1.205243
C -2.240083 3.199416 0.143579
C -1.023334 4.088483 0.408310
C -0.878611 5.239853 -0.592146
C -0.715927 4.816295 -2.056417
C 0.475161 3.893944 -2.318562
C 0.675088 3.595626 -3.802868
O 1.868530 1.796735 0.594627
O 3.876891 2.438616 -0.989813
O -1.617375 0.456592 2.686444
O -2.917984 2.791697 2.412726
H 4.641617 0.390994 -2.545118
H 4.535421 -0.161086 -0.871147
H 2.603952 -0.799505 -3.160615
H 3.624278 -1.936644 -2.299630
H 2.483246 1.604582 -2.262423
H 0.639379 -0.369187 1.635203
H 1.733057 -3.198472 -1.916826
H -0.836617 -4.407858 -1.279150
H 0.505391 -5.037527 -0.319241

H -3.088809 -4.209368 1.156442
H 0.392285 -4.786429 2.272398
H -0.869038 -6.494171 3.462512
H -2.336646 -6.189173 2.379158
H -1.761610 -0.210452 -0.005551
H -0.643288 1.033073 -0.501562
H -0.456986 1.860354 1.803084
H -3.322847 1.490143 0.858099
H -2.142980 2.701682 -0.823674
H -3.137913 3.821725 0.072269
H -1.103546 4.504088 1.415640
H -0.112676 3.486391 0.397848
H -1.750820 5.897326 -0.510353
H -0.011247 5.843121 -0.303798
H -1.630014 4.327217 -2.408559
H -0.605106 5.717906 -2.668094
H 1.383062 4.347275 -1.906703
H 0.336812 2.953767 -1.778913
H 0.848850 4.514937 -4.367926
H 1.530629 2.937284 -3.970212
H -0.206979 3.107828 -4.226097
H 3.396511 2.841543 -0.246062
H -1.858427 -0.456795 2.430830
H -2.848347 2.113347 3.100950

2d-c130, ΔG = 2.0814 kcal/mol, population = 0.59 %
C 3.914774 -0.048860 -1.929973
C 2.923291 -1.122996 -2.369070
C 1.835507 -1.328069 -1.356992
C 1.443101 -0.384305 -0.457794
C 2.143090 0.884256 -0.421396
C 3.152446 1.198447 -1.522247
C 0.312785 -0.592739 0.541096
N 1.229810 -2.570682 -1.382889
C 0.287057 -2.905189 -0.469173
C -0.137887 -2.025607 0.477953
C -0.380399 -4.243025 -0.419902
C -1.221181 -4.168123 0.873508
C -1.141784 -2.667316 1.270715
O -1.888926 -2.184810 2.123786
O -2.569721 -4.514546 0.597096
C -0.608207 -4.995755 1.976132
C -1.253990 -5.913930 2.681571
C -0.876657 0.380693 0.287482

C -1.234319 1.248293 1.487543
C -2.501075 2.076204 1.241657
C -2.398818 3.081392 0.100794
C -1.246657 4.081661 0.210473
C -1.240101 5.118330 -0.917582
C -1.001919 4.541602 -2.320177
C 0.308273 3.761863 -2.483552
C 1.559397 4.592362 -2.200494
O 1.945298 1.730880 0.451165
O 4.029307 2.224093 -1.098265
O -1.420332 0.489399 2.693962
O -2.838455 2.779047 2.445998
H 4.605946 0.182344 -2.740656
H 4.499608 -0.403079 -1.077827
H 2.457112 -0.837518 -3.318972
H 3.438386 -2.068743 -2.545814
H 2.560784 1.546879 -2.383815
H 0.699855 -0.380403 1.540670
H 1.540949 -3.252324 -2.059503
H -1.042628 -4.375599 -1.277282
H 0.341258 -5.060749 -0.419216
H -3.109888 -4.152977 1.314635
H 0.437129 -4.780234 2.178371
H -0.754415 -6.468809 3.465206
H -2.293583 -6.148269 2.490649
H -1.753147 -0.195391 -0.022465
H -0.633058 1.034399 -0.549562
H -0.402336 1.921275 1.697357
H -3.311429 1.370160 1.008884
H -2.330633 2.523162 -0.835336
H -3.346353 3.627776 0.056610
H -1.318257 4.598622 1.170050
H -0.290585 3.552899 0.220127
H -2.195794 5.653066 -0.917168
H -0.474841 5.868715 -0.701346
H -1.837249 3.891481 -2.595838
H -1.011541 5.366511 -3.040531
H 0.300389 2.885947 -1.828884
H 0.355852 3.373178 -3.505038
H 1.574714 5.494543 -2.818272
H 1.604314 4.905912 -1.155765
H 2.468296 4.026513 -2.409902
H 3.565507 2.677260 -0.373821
H -1.716218 -0.418192 2.476889

H -2.681917 2.142085 3.158795

2d-c145, ΔG = 2.1386 kcal/mol, population = 0.53 %
C 3.782255 1.167282 -1.527313
C 3.423755 -0.258162 -1.944046
C 2.257584 -0.799412 -1.169809
C 1.329525 -0.021141 -0.549677
C 1.484986 1.420023 -0.579523
C 2.526412 2.021479 -1.515948
C 0.121204 -0.580550 0.186423
N 2.171184 -2.180261 -1.110499
C 1.218269 -2.783510 -0.359070
C 0.272357 -2.065468 0.303125
C 1.105525 -4.264446 -0.174739
C -0.004825 -4.406027 0.893801
C -0.569306 -2.963929 1.024315
O -1.600758 -2.711783 1.650639
O -1.023377 -5.272067 0.413260
C 0.562073 -4.833577 2.223971
C 0.136986 -5.871170 2.931519
C -1.211455 -0.179785 -0.519483
C -2.147807 0.621883 0.374388
C -3.302564 1.262092 -0.409557
C -3.981429 2.406268 0.349080
C -3.047952 3.556808 0.750058
C -2.207551 4.125664 -0.394429
C -1.295252 5.270108 0.041015
C -0.390577 5.776359 -1.081724
C 0.564743 6.880177 -0.632405
O 0.800405 2.180395 0.105114
O 2.802774 3.357358 -1.139018
O -2.741416 -0.169158 1.418301
O -4.256880 0.253523 -0.767354
H 4.515673 1.587109 -2.215851
H 4.220537 1.167477 -0.526427
H 3.165714 -0.283350 -3.008951
H 4.282271 -0.919913 -1.815582
H 2.077418 2.005931 -2.521470
H 0.103498 -0.141644 1.188807
H 2.877636 -2.735113 -1.571363
H 0.784664 -4.748659 -1.098686
H 2.052716 -4.711213 0.129752
H -1.811701 -5.118079 0.953263
H 1.367959 -4.207249 2.595972

H 0.585559 -6.117078 3.885393
H -0.660064 -6.512643 2.577322
H -1.721992 -1.070263 -0.893750
H -0.975655 0.429207 -1.393547
H -1.554911 1.414965 0.832560
H -2.909633 1.642705 -1.352633
H -4.783964 2.787711 -0.289126
H -4.457410 1.994334 1.243741
H -3.653658 4.358435 1.184186
H -2.377072 3.225254 1.548219
H -1.578031 3.339301 -0.818277
H -2.868097 4.464916 -1.200786
H -1.898591 6.099793 0.427236
H -0.670926 4.928078 0.873998
H 0.185881 4.934224 -1.478669
H -1.009544 6.139237 -1.909092
H 1.216791 6.528315 0.171599
H 1.200784 7.218653 -1.453206
H 0.014683 7.746841 -0.256261
H 2.067812 3.615853 -0.555970
H -2.295548 -1.036421 1.493321
H -4.333966 -0.300610 0.025121

2d-c53, ΔG = 2.1611 kcal/mol, population = 0.51 %
C 3.149586 -0.275748 -2.922657
C 1.640607 -0.484037 -2.851307
C 1.163772 -0.744238 -1.453146
C 1.822240 -0.355358 -0.326898
C 3.139217 0.238968 -0.477553
C 3.568327 0.724880 -1.859414
C 1.291462 -0.598852 1.087599
N -0.041501 -1.416614 -1.369756
C -0.609557 -1.689770 -0.170440
C -0.029571 -1.320145 1.002916
C -1.941444 -2.354968 -0.031799
C -2.094460 -2.517605 1.494776
C -0.936695 -1.648771 2.065786
O -0.929890 -1.282107 3.240267
O -3.335033 -1.977588 1.922329
C -1.889286 -3.948296 1.924911
C -2.722813 -4.632208 2.696737
C 1.257808 0.691967 1.944664
C 0.427841 1.861985 1.414212
C -1.089192 1.691149 1.579236

C -1.900411 2.837210 0.983625
C -1.649369 3.090706 -0.505326
C -1.952007 1.898429 -1.414247
C -1.622631 2.169049 -2.880476
C -1.970900 1.007450 -3.809764
C -1.572138 1.261303 -5.261668
O 3.929480 0.369992 0.456938
O 4.963400 0.952918 -1.881415
O 0.840422 3.074506 2.063381
O -1.414279 1.622027 2.980110
H 3.435317 0.085194 -3.910685
H 3.669613 -1.220117 -2.745095
H 1.122842 0.409202 -3.219164
H 1.335409 -1.308185 -3.498633
H 3.034097 1.672241 -2.031583
H 2.003200 -1.254313 1.601745
H -0.497485 -1.713243 -2.219795
H -2.735206 -1.707669 -0.409714
H -1.984379 -3.298284 -0.577672
H -3.234184 -1.746173 2.857649
H -0.972432 -4.404781 1.563066
H -2.511905 -5.656588 2.975561
H -3.643840 -4.195355 3.061511
H 2.281948 1.046546 2.031683
H 0.930029 0.426990 2.950348
H 0.655052 2.005465 0.356829
H -1.390079 0.761996 1.097317
H -2.957114 2.606131 1.144761
H -1.682301 3.745274 1.549830
H -2.267295 3.937328 -0.818890
H -0.613215 3.407534 -0.656500
H -1.386277 1.021987 -1.088193
H -3.009464 1.628665 -1.316123
H -2.152142 3.068060 -3.215322
H -0.553556 2.395337 -2.969325
H -1.477284 0.098712 -3.451702
H -3.045734 0.808960 -3.750453
H -1.836425 0.417948 -5.902981
H -2.072014 2.150320 -5.654595
H -0.494353 1.422547 -5.348767
H 5.236571 0.955619 -0.947279
H 0.437047 3.056388 2.942975
H -1.224400 0.721346 3.286317

2d-c65, ΔG = 2.1618 kcal/mol, population = 0.51 %

C 5.328367 -1.187387 -0.368762
C 4.219011 -1.285187 -1.413577
C 2.858651 -1.119776 -0.806165
C 2.620508 -0.469799 0.365889
C 3.738883 0.045258 1.124409
C 5.118042 0.058645 0.471889
C 1.219855 -0.248845 0.917605
N 1.816352 -1.681067 -1.518909
C 0.567865 -1.744411 -0.988787
C 0.263461 -1.149061 0.192970
C -0.570239 -2.473577 -1.629539
C -1.697168 -2.373439 -0.577760
C -1.105646 -1.429982 0.518241
O -1.780774 -1.006942 1.449125
O -2.831470 -1.739001 -1.159050
C -2.014305 -3.712326 0.031978
C -3.227306 -4.241837 0.114940
C 0.764007 1.233356 0.846132
C 0.455935 1.759548 -0.548055
C -0.208241 3.142572 -0.537227
C -1.562697 3.197929 0.162303
C -2.567435 2.128186 -0.265587
C -3.938883 2.322537 0.378906
C -4.897447 1.165097 0.095747
C -6.327786 1.388397 0.594934
C -6.439798 1.522386 2.113975
O 3.626514 0.499966 2.265156
O 6.122144 0.194954 1.459808
O 1.622273 1.814881 -1.396433
O -0.387840 3.577941 -1.892469
H 6.303298 -1.151596 -0.855392
H 5.308702 -2.063688 0.283546
H 4.347573 -0.502229 -2.170277
H 4.272130 -2.240140 -1.939491
H 5.128510 0.938249 -0.191983
H 1.230623 -0.504674 1.979988
H 2.019836 -2.165444 -2.380926
H -0.881309 -1.976722 -2.550299
H -0.305722 -3.502740 -1.875852
H -3.377983 -1.402855 -0.434122
H -1.155755 -4.239429 0.438679
H -3.385542 -5.206166 0.580404
H -4.095151 -3.735846 -0.289011

H 1.522201 1.861523 1.320446
H -0.138048 1.320647 1.451929
H -0.210236 1.071719 -1.068658
H 0.474709 3.838953 -0.029215
H -1.403079 3.136791 1.241958
H -1.980110 4.191822 -0.025460
H -2.668961 2.129955 -1.354226
H -2.194403 1.140733 0.012162
H -3.809455 2.438410 1.459236
H -4.382512 3.258435 0.019983
H -4.920315 0.978933 -0.983127
H -4.498141 0.255958 0.561713
H -6.737087 2.285341 0.117693
H -6.951354 0.553053 0.262562
H -6.021631 0.644070 2.613698
H -5.903330 2.398871 2.481885
H -7.482160 1.618875 2.425218
H 5.660470 0.529708 2.247878
H 2.326975 2.272324 -0.919526
H 0.414011 3.307973 -2.362994

2d-c72, ΔG = 2.1869 kcal/mol, population = 0.49 %
C 3.829932 0.043123 -1.724173
C 2.954725 -1.119302 -2.185389
C 1.821423 -1.383126 -1.238717
C 1.304944 -0.452380 -0.390292
C 1.914400 0.861614 -0.332825
C 2.953391 1.235797 -1.385983
C 0.118500 -0.717096 0.527024
N 1.305785 -2.665794 -1.274788
C 0.321847 -3.044322 -0.423014
C -0.229983 -2.176446 0.466089
C -0.270350 -4.419228 -0.392511
C -1.183337 -4.377928 0.842748
C -1.249942 -2.867547 1.195682
O -2.096183 -2.418482 1.971479
O -2.486401 -4.849734 0.493989
C -0.668021 -5.111824 2.058801
C 0.564159 -5.557680 2.264605
C -1.108357 0.166349 0.150988
C -1.603586 1.051257 1.285935
C -2.867205 1.829468 0.906739
C -2.711385 2.761101 -0.289521
C -1.519087 3.716450 -0.233754

C -1.443165 4.622448 -1.462899
C -0.223662 5.548472 -1.479314
C 1.116624 4.816992 -1.579638
C 2.308164 5.767098 -1.676817
O 1.615710 1.698382 0.520067
O 3.728268 2.331753 -0.938814
O -1.870194 0.317968 2.493765
O -3.292281 2.602706 2.039249
H 4.543134 0.311100 -2.503839
H 4.395308 -0.243286 -0.834186
H 2.529814 -0.901546 -3.171901
H 3.553223 -2.025241 -2.296864
H 2.382936 1.525534 -2.283008
H 0.410357 -0.455652 1.547144
H 1.710016 -3.338334 -1.909885
H -0.881834 -4.587030 -1.280742
H 0.487304 -5.200608 -0.359542
H -3.101881 -4.507132 1.158405
H -1.417279 -5.222675 2.837609
H 0.827413 -6.045788 3.194295
H 1.357592 -5.451248 1.535157
H -1.923243 -0.475489 -0.195687
H -0.843532 0.802307 -0.693358
H -0.817865 1.760634 1.548076
H -3.648018 1.091631 0.672563
H -2.641681 2.148476 -1.192657
H -3.639474 3.333321 -0.383787
H -1.580147 4.330271 0.669657
H -0.594241 3.143125 -0.154885
H -1.430032 3.999998 -2.365716
H -2.355529 5.225237 -1.520471
H -0.311849 6.238699 -2.325177
H -0.227503 6.169681 -0.575884
H 1.246657 4.163125 -0.714494
H 1.099755 4.159385 -2.456088
H 2.360805 6.419480 -0.800872
H 3.249035 5.216874 -1.740555
H 2.231422 6.406386 -2.560454
H 3.198438 2.756542 -0.243329
H -2.086390 -0.611620 2.279941
H -3.195981 2.011602 2.800017

2d-c407, ΔG = 2.2578 kcal/mol, population = 0.44 %
C 5.502440 -0.704838 0.207730

C 4.807971 -1.745212 -0.668470
C 3.317167 -1.689693 -0.519166
C 2.635304 -0.572320 -0.133315
C 3.371280 0.619773 0.204581
C 4.876403 0.655944 -0.034842
C 1.116707 -0.511698 -0.061806
N 2.639593 -2.859261 -0.783889
C 1.310592 -2.972256 -0.508285
C 0.571457 -1.909805 -0.100147
C 0.557319 -4.261994 -0.595731
C -0.804818 -3.915153 0.043534
C -0.761305 -2.362295 0.196737
O -1.750300 -1.712112 0.508453
O -1.861045 -4.266752 -0.839603
C -0.942699 -4.535438 1.409958
C -1.970020 -5.272991 1.808819
C 0.569324 0.367933 -1.216727
C -0.759297 1.084062 -0.956095
C -0.720687 1.996213 0.279725
C -2.005674 2.786140 0.491710
C -3.246325 1.915388 0.692558
C -4.480928 2.703294 1.138922
C -4.980081 3.724643 0.116617
C -6.263097 4.432422 0.551112
C -6.759327 5.453261 -0.471249
O 2.834127 1.639804 0.659038
O 5.479479 1.649568 0.772396
O -1.111384 1.839730 -2.125905
O 0.348562 2.947182 0.156956
H 6.568110 -0.672354 -0.018820
H 5.388361 -0.965976 1.262423
H 5.052478 -1.571074 -1.722489
H 5.162932 -2.747910 -0.425467
H 4.998479 0.915959 -1.099133
H 0.848035 -0.049632 0.889498
H 3.166493 -3.678597 -1.049462
H 0.410448 -4.557297 -1.636279
H 1.076117 -5.073847 -0.084385
H -2.641079 -3.764026 -0.563479
H -0.122586 -4.322135 2.089881
H -2.009240 -5.680328 2.810900
H -2.794652 -5.502355 1.145741
H 0.457792 -0.251131 -2.108670
H 1.313671 1.128862 -1.460540

H -1.552979 0.351593 -0.822042
H -0.555983 1.370946 1.163179
H -1.858242 3.415112 1.374799
H -2.144445 3.457719 -0.357284
H -3.481071 1.383924 -0.234480
H -3.026302 1.146515 1.439749
H -5.289998 1.997363 1.354111
H -4.261717 3.215542 2.082845
H -4.206219 4.476024 -0.068661
H -5.152247 3.219558 -0.841347
H -7.042854 3.684606 0.730678
H -6.090187 4.930316 1.511273
H -6.969584 4.975042 -1.431636
H -7.675379 5.944288 -0.135548
H -6.009215 6.229226 -0.645617
H 4.769424 2.275540 0.991178
H -0.513713 2.599763 -2.151962
H 1.188706 2.490425 0.341388

2d-c419, ΔG = 2.2917 kcal/mol, population = 0.41 %
C 5.414540 -1.178696 0.258332
C 4.600478 -2.011354 -0.730022
C 3.130020 -1.753219 -0.593639
C 2.606512 -0.594411 -0.098336
C 3.497755 0.436159 0.372187
C 4.997559 0.276350 0.153316
C 1.111373 -0.320786 -0.034758
N 2.297911 -2.776662 -0.991173
C 0.959736 -2.721873 -0.744853
C 0.370841 -1.612378 -0.230692
C 0.030232 -3.868926 -0.985758
C -1.278071 -3.403240 -0.310631
C -1.020592 -1.895426 -0.000284
O -1.916603 -1.145084 0.363581
O -2.365141 -3.515832 -1.217741
C -1.510716 -4.131339 0.988451
C -2.637216 -4.745154 1.323900
C 0.723579 0.747904 -1.089878
C -0.497012 1.612477 -0.761603
C -0.354440 2.377817 0.563419
C -1.519710 3.315383 0.852133
C -2.870234 2.615648 0.988028
C -3.994962 3.583068 1.354535
C -5.349659 2.906732 1.589869

C -5.895214 2.099820 0.405201
C -6.080754 2.920651 -0.870884
O 3.101661 1.470928 0.926886
O 5.717137 1.083162 1.066732
O -0.720790 2.525726 -1.847467
O 0.840850 3.174025 0.545052
H 6.479370 -1.278889 0.048221
H 5.236652 -1.527044 1.278455
H 4.896463 -1.769443 -1.757055
H 4.799282 -3.074767 -0.588897
H 5.182844 0.623764 -0.876326
H 0.888983 0.070548 0.959169
H 2.708141 -3.633901 -1.332621
H -0.139826 -4.011742 -2.054521
H 0.416606 -4.803319 -0.576730
H -3.066985 -2.933730 -0.892063
H -0.670054 -4.111085 1.676358
H -2.739101 -5.241017 2.280643
H -3.485364 -4.782372 0.651746
H 0.547504 0.251400 -2.045795
H 1.573518 1.416558 -1.241860
H -1.385763 0.986991 -0.708033
H -0.295655 1.648231 1.377561
H -1.287740 3.844756 1.780624
H -1.571422 4.068134 0.061271
H -3.117568 2.110539 0.052066
H -2.803776 1.832240 1.751453
H -3.715540 4.127720 2.262302
H -4.087423 4.338289 0.567485
H -5.267187 2.243466 2.457673
H -6.082838 3.674766 1.858978
H -5.235329 1.252055 0.199547
H -6.856842 1.667389 0.697403
H -5.127563 3.289408 -1.255403
H -6.543796 2.323125 -1.659117
H -6.721367 3.787997 -0.688474
H 5.099115 1.779228 1.344971
H -0.022259 3.192673 -1.794619
H 1.603723 2.585897 0.688528

2d-c624, ΔG = 2.3268 kcal/mol, population = 0.39 %
C 4.656288 -0.820891 1.700728
C 4.317267 -1.816391 0.593035
C 2.916166 -1.633440 0.092265

C 2.228713 -0.458255 0.178545
C 2.843722 0.674598 0.823788
C 4.306554 0.582912 1.241994
C 0.833886 -0.271407 -0.396604
N 2.322854 -2.745104 -0.465730
C 0.996833 -2.743727 -0.777384
C 0.243071 -1.618590 -0.690107
C 0.246382 -3.965600 -1.203564
C -1.226731 -3.502789 -1.155804
C -1.125525 -1.955661 -0.975065
O -2.099606 -1.221756 -1.080921
O -1.870167 -3.792494 -2.388301
C -1.941083 -4.082931 0.038214
C -3.092627 -4.738701 -0.009775
C 0.900007 0.634742 -1.653257
C -0.358381 1.446394 -1.974169
C -0.814038 2.326870 -0.802316
C -2.045077 3.155782 -1.141611
C -2.601351 3.962717 0.033527
C -3.108161 3.113707 1.200033
C -3.762298 3.949970 2.300153
C -4.196017 3.148194 3.531512
C -5.284053 2.112047 3.247719
O 2.246275 1.739885 1.032106
O 4.592426 1.537672 2.246607
O -0.103079 2.244370 -3.141122
O 0.242127 3.217702 -0.411617
H 5.716035 -0.879813 1.948757
H 4.084546 -1.053253 2.602177
H 5.002531 -1.684539 -0.251988
H 4.448307 -2.840384 0.946189
H 4.887406 0.812384 0.333626
H 0.229006 0.224896 0.363434
H 2.842481 -3.610344 -0.496984
H 0.509108 -4.244834 -2.225655
H 0.449977 -4.819308 -0.556050
H -2.654325 -3.226983 -2.439736
H -1.445353 -3.913233 0.990043
H -3.555106 -5.120565 0.891317
H -3.601359 -4.925168 -0.947190
H 1.149347 0.020155 -2.520043
H 1.722705 1.342254 -1.529655
H -1.179625 0.781100 -2.238639
H -1.062498 1.668094 0.033196

H -1.796789 3.828476 -1.965665
H -2.815464 2.473407 -1.514850
H -1.832541 4.651756 0.393494
H -3.425209 4.583162 -0.334450
H -3.819193 2.374176 0.818560
H -2.280233 2.545972 1.637522
H -3.059011 4.728724 2.614007
H -4.632979 4.472290 1.886337
H -3.320262 2.649345 3.960929
H -4.556178 3.844053 4.295323
H -5.608348 1.619540 4.167033
H -6.160480 2.583238 2.793941
H -4.934693 1.335882 2.564302
H 3.907583 2.221431 2.160822
H 0.483375 2.960275 -2.859373
H 0.900757 2.710880 0.095457

2d-c1, ΔG = 2.3350 kcal/mol, population = 0.38 %
C 4.923265 -1.112248 -1.320070
C 3.577683 -1.110408 -2.040354
C 2.422667 -1.022224 -1.087950
C 2.512032 -0.531420 0.177600
C 3.801721 -0.094884 0.669602
C 4.956552 0.027026 -0.317546
C 1.312239 -0.384528 1.103916
N 1.213651 -1.477758 -1.584682
C 0.118513 -1.546424 -0.786674
C 0.134495 -1.087998 0.492079
C -1.189732 -2.130266 -1.220119
C -1.978952 -2.223945 0.104726
C -1.152461 -1.334415 1.082756
O -1.608476 -0.944754 2.150825
O -3.282236 -1.690851 -0.047423
C -1.978071 -3.636273 0.636369
C -3.055682 -4.286895 1.053276
C 0.973821 1.091413 1.426269
C 0.816346 1.990930 0.207010
C -0.071922 3.212839 0.477494
C -1.541590 2.892210 0.733830
C -2.254784 2.133551 -0.385537
C -3.749298 1.968900 -0.115319
C -4.496397 1.308256 -1.273579
C -6.001869 1.145308 -1.038927
C -6.356532 0.159897 0.076046

O 4.001464 0.211967 1.846186
O 6.189245 0.073377 0.376669
O 2.094806 2.449107 -0.288728
O 0.000159 4.105801 -0.641114
H 5.736098 -1.004442 -2.038522
H 5.065232 -2.056999 -0.789759
H 3.518433 -0.253511 -2.721379
H 3.473664 -2.005359 -2.656574
H 4.793063 0.976002 -0.852010
H 1.553076 -0.856228 2.060993
H 1.177022 -1.847977 -2.523009
H -1.706338 -1.454753 -1.904362
H -1.065266 -3.091344 -1.720304
H -3.565547 -1.375395 0.823237
H -0.999645 -4.106329 0.678567
H -2.985294 -5.297566 1.434472
H -4.038748 -3.834818 1.015424
H 1.748020 1.505723 2.075158
H 0.051386 1.086314 2.005848
H 0.395678 1.434653 -0.631476
H 0.330469 3.722422 1.365618
H -1.627295 2.336586 1.670852
H -2.052064 3.845448 0.900757
H -2.109139 2.664452 -1.329788
H -1.811737 1.142127 -0.514364
H -3.880401 1.383608 0.799817
H -4.191997 2.952168 0.083118
H -4.340753 1.909749 -2.175606
H -4.054449 0.326698 -1.467400
H -6.439807 2.124218 -0.814338
H -6.468888 0.808747 -1.969597
H -5.934918 -0.826890 -0.130769
H -5.973812 0.488626 1.044321
H -7.438686 0.047747 0.172941
H 5.957254 0.300166 1.293623
H 2.603999 2.792151 0.457602
H 0.919339 4.071431 -0.943816

2d-c285, ΔG = 2.3419 kcal/mol, population = 0.38 %
C 4.039573 0.151548 -1.535594
C 3.160696 -0.954097 -2.116501
C 1.972676 -1.239693 -1.246997
C 1.424628 -0.337777 -0.387496
C 2.062515 0.951873 -0.219202

C 3.178921 1.350964 -1.179001
C 0.185674 -0.622135 0.447080
N 1.444771 -2.513619 -1.358210
C 0.444301 -2.925162 -0.541603
C -0.134094 -2.086113 0.359303
C -0.122114 -4.309680 -0.552126
C -1.093145 -4.301921 0.650608
C -1.155953 -2.802514 1.057253
O -2.007976 -2.375358 1.838769
O -2.380991 -4.732040 0.234803
C -0.544051 -5.098806 1.807153
C -1.199075 -6.059732 2.443921
C -1.037060 0.234387 -0.002864
C -1.647752 1.085858 1.106903
C -3.024423 1.641572 0.719872
C -3.070312 2.608681 -0.458850
C -2.220009 3.883606 -0.337386
C -0.810517 3.775092 -0.921486
C -0.039938 5.093269 -0.889088
C 1.341610 4.993457 -1.534201
C 2.127171 6.302112 -1.484849
O 1.733639 1.747680 0.661646
O 3.953549 2.393171 -0.615388
O -1.796985 0.364426 2.342635
O -3.590744 2.304035 1.859160
H 4.806408 0.441199 -2.254265
H 4.541095 -0.204322 -0.632499
H 2.796402 -0.661770 -3.107921
H 3.740440 -1.868566 -2.253305
H 2.677991 1.714025 -2.090553
H 0.406556 -0.359332 1.483058
H 1.874596 -3.167684 -1.996030
H -0.682242 -4.492314 -1.470838
H 0.655667 -5.070076 -0.472954
H -3.013807 -4.423954 0.899536
H 0.460138 -4.820010 2.113753
H -0.747159 -6.587708 3.273734
H -2.198062 -6.356642 2.150262
H -1.802556 -0.431463 -0.411453
H -0.739023 0.888367 -0.821349
H -0.976507 1.912490 1.344200
H -3.654420 0.775503 0.468475
H -2.802825 2.066843 -1.370311
H -4.121069 2.886589 -0.574574

H -2.738805 4.694493 -0.857487
H -2.166685 4.185478 0.712291
H -0.229758 3.022795 -0.385031
H -0.882354 3.427433 -1.958904
H -0.622523 5.870307 -1.397819
H 0.069612 5.423373 0.150415
H 1.912718 4.206516 -1.034624
H 1.227180 4.672650 -2.575192
H 2.282696 6.626092 -0.452312
H 3.108720 6.197259 -1.952372
H 1.592831 7.101537 -2.005200
H 3.407052 2.762938 0.099052
H -1.996071 -0.576147 2.155632
H -3.353858 1.744871 2.614209

2d-c9, ΔG = 2.3626 kcal/mol, population = 0.36 %
C 5.225351 -1.247066 -0.656832
C 4.027773 -1.326621 -1.599875
C 2.724568 -1.170573 -0.874342
C 2.591169 -0.557162 0.333186
C 3.775286 -0.057345 1.000127
C 5.088210 -0.018875 0.224955
C 1.246784 -0.345415 1.015747
N 1.621901 -1.698213 -1.521550
C 0.403713 -1.711266 -0.922206
C 0.196061 -1.138618 0.291248
C -0.807187 -2.343734 -1.530267
C -1.878642 -2.198690 -0.428865
C -1.180725 -1.322385 0.660348
O -1.790531 -0.871393 1.622143
O -2.995143 -1.486657 -0.947071
C -2.251205 -3.528928 0.169827
C -3.489954 -3.984017 0.300711
C 0.855251 1.150664 1.077515
C 0.779657 1.829032 -0.289958
C -0.371824 2.836770 -0.403656
C -1.729160 2.152046 -0.408260
C -2.915484 3.112118 -0.492653
C -4.265404 2.400264 -0.350136
C -4.559979 1.914568 1.072272
C -5.893545 1.172355 1.214834
C -5.890725 -0.224171 0.593190
O 3.766503 0.360499 2.159232
O 6.178378 0.103841 1.118969

O 2.023830 2.471416 -0.640805
O -0.229718 3.578722 -1.620644
H 6.152640 -1.196945 -1.227862
H 5.266721 -2.136991 -0.024165
H 4.091312 -0.532417 -2.352655
H 4.031363 -2.273270 -2.143390
H 5.030886 0.874079 -0.417558
H 1.320658 -0.691622 2.050231
H 1.750988 -2.160720 -2.409391
H -1.121902 -1.801315 -2.423479
H -0.622858 -3.380908 -1.814262
H -3.495719 -1.141543 -0.194040
H -1.410011 -4.117524 0.525295
H -3.686242 -4.946869 0.754552
H -4.343015 -3.417621 -0.050982
H 1.573880 1.681756 1.704184
H -0.105388 1.215528 1.586781
H 0.637123 1.088457 -1.079545
H -0.313627 3.529350 0.450070
H -1.762958 1.454121 -1.251310
H -1.817901 1.550281 0.495501
H -2.824044 3.872995 0.291042
H -2.879440 3.643036 -1.446134
H -5.066397 3.081783 -0.654230
H -4.297010 1.560003 -1.051093
H -3.754770 1.261870 1.424199
H -4.561439 2.784805 1.736854
H -6.140184 1.083415 2.276704
H -6.690686 1.773039 0.763971
H -5.659325 -0.193890 -0.473546
H -5.153066 -0.861788 1.090502
H -6.861406 -0.712005 0.701548
H 5.789643 0.413925 1.954922
H 2.328031 2.981854 0.120726
H 0.721832 3.726683 -1.724652

2d-c7, ΔG = 2.3695 kcal/mol, population = 0.36 %
C 5.030483 -0.996623 -1.242356
C 3.721097 -0.984692 -2.026461
C 2.520423 -0.955044 -1.128454
C 2.540534 -0.517509 0.159429
C 3.797344 -0.082405 0.731329
C 4.995843 0.099245 -0.192615
C 1.295364 -0.428796 1.031714

N 1.344718 -1.407509 -1.702022
C 0.214060 -1.527289 -0.961651
C 0.160834 -1.123746 0.334450
C -1.061461 -2.112478 -1.482117
C -1.911301 -2.275034 -0.202169
C -1.148400 -1.415106 0.851140
O -1.661662 -1.078740 1.911290
O -3.215136 -1.756501 -0.394807
C -1.910777 -3.708555 0.269232
C -2.995383 -4.393233 0.605722
C 0.916000 1.026687 1.398542
C 0.800941 1.974585 0.211678
C -0.120254 3.170163 0.489677
C -1.594572 2.816493 0.662091
C -2.241013 2.094727 -0.520420
C -3.743488 1.895658 -0.328863
C -4.423746 1.273917 -1.548259
C -5.935476 1.077471 -1.392674
C -6.324629 0.039142 -0.338902
O 3.935292 0.176527 1.928117
O 6.192877 0.135039 0.562037
O 2.093307 2.473022 -0.202443
O -0.011133 4.110361 -0.586448
H 5.874760 -0.845744 -1.915383
H 5.163339 -1.960863 -0.745825
H 3.679658 -0.100475 -2.673024
H 3.662301 -1.854018 -2.683972
H 4.841590 1.067504 -0.694243
H 1.498289 -0.937003 1.978846
H 1.359524 -1.737744 -2.655746
H -1.556703 -1.416554 -2.161703
H -0.896168 -3.049279 -2.015461
H -3.544765 -1.481967 0.473413
H -0.927290 -4.164579 0.339100
H -2.925417 -5.418069 0.947142
H -3.983371 -3.955376 0.539317
H 1.650929 1.425115 2.100865
H -0.032810 0.982544 1.932091
H 0.430207 1.447980 -0.668549
H 0.231001 3.648096 1.416276
H -1.714590 2.220779 1.570292
H -2.128797 3.753932 0.843641
H -2.060421 2.667229 -1.433844
H -1.775120 1.116589 -0.668931

H -3.907188 1.269651 0.553504
H -4.212053 2.862584 -0.110498
H -4.236641 1.916111 -2.415583
H -3.955934 0.308651 -1.762256
H -6.400706 2.038752 -1.147696
H -6.352304 0.774352 -2.358040
H -5.876480 -0.931051 -0.567325
H -5.993542 0.331369 0.659644
H -7.408010 -0.093887 -0.298500
H 5.913233 0.318637 1.475358
H 2.560368 2.792068 0.581158
H 0.921849 4.103302 -0.845677

2d-c36, ΔG = 2.3701 kcal/mol, population = 0.36 %
C 5.177240 -1.008965 -0.380761
C 4.073185 -1.155068 -1.425005
C 2.703944 -1.097077 -0.815443
C 2.429356 -0.530499 0.391465
C 3.513090 0.039591 1.164426
C 4.878430 0.188742 0.502867
C 1.019235 -0.417575 0.952257
N 1.695168 -1.665355 -1.574006
C 0.435868 -1.781324 -1.078746
C 0.098795 -1.274790 0.134538
C -0.685343 -2.460520 -1.800647
C -1.823738 -2.475575 -0.755611
C -1.274448 -1.590306 0.406027
O -1.978816 -1.233743 1.342988
O -2.983319 -1.851261 -1.294065
C -2.084825 -3.866011 -0.240277
C -3.275612 -4.447735 -0.194460
C 0.513636 1.046117 0.965852
C 0.695517 1.779778 -0.363536
C -0.508850 2.663593 -0.718583
C -1.681964 1.828956 -1.215399
C -2.998814 2.602547 -1.334901
C -3.670994 2.918310 0.005992
C -4.182860 1.682712 0.746962
C -4.884822 2.012639 2.063748
C -5.388980 0.770086 2.795878
O 3.374172 0.434819 2.322754
O 5.879676 0.372225 1.486394
O 1.906427 2.564503 -0.376463
O -0.148354 3.582253 -1.756135

H 6.143382 -0.882074 -0.869543
H 5.229715 -1.906074 0.240818
H 4.147612 -0.348614 -2.163780
H 4.186951 -2.091922 -1.973740
H 4.807599 1.087788 -0.129154
H 1.020619 -0.762698 1.989252
H 1.927882 -2.084184 -2.462371
H -0.993659 -1.873863 -2.668036
H -0.406451 -3.456174 -2.147666
H -3.531140 -1.574612 -0.544947
H -1.204398 -4.385579 0.127367
H -3.394050 -5.448229 0.201292
H -4.163842 -3.948854 -0.561154
H 1.034460 1.599509 1.749632
H -0.538829 1.029747 1.250006
H 0.835649 1.072084 -1.182961
H -0.793028 3.231503 0.178416
H -1.396066 1.427497 -2.191619
H -1.822760 0.968751 -0.560735
H -2.815953 3.530636 -1.882341
H -3.694223 2.016389 -1.943901
H -2.976838 3.465343 0.653046
H -4.511672 3.596443 -0.173651
H -4.876013 1.137357 0.095093
H -3.357586 0.996007 0.954098
H -4.192347 2.562308 2.710372
H -5.723579 2.689360 1.868302
H -6.104005 0.217134 2.180808
H -4.563602 0.093441 3.031197
H -5.886163 1.030290 3.732950
H 5.402248 0.639101 2.290598
H 1.986534 3.018369 0.472245
H 0.773794 3.821969 -1.580286

2d-c69, ΔG = 2.3751 kcal/mol, population = 0.36 %
C 4.103153 -0.283459 -1.964106
C 3.281568 -1.524678 -2.307471
C 2.055889 -1.644340 -1.451052
C 1.456147 -0.592223 -0.830331
C 2.054543 0.723383 -0.937609
C 3.193335 0.931929 -1.930278
C 0.175702 -0.715984 -0.017133
N 1.547558 -2.924367 -1.314091
C 0.511385 -3.172049 -0.477095

C -0.120163 -2.170202 0.192119
C -0.032404 -4.537753 -0.198268
C -1.046975 -4.289006 0.942449
C -1.136608 -2.739019 1.016355
O -1.994240 -2.160386 1.686716
O -2.314515 -4.818897 0.581000
C -0.534978 -4.813946 2.260030
C -1.202690 -5.631244 3.062598
C -1.008297 0.030370 -0.708478
C -1.658031 1.097459 0.164311
C -2.634507 1.975460 -0.626220
C -3.077179 3.225381 0.140001
C -1.945837 4.177430 0.543800
C -1.114733 4.698128 -0.631222
C -0.078780 5.756083 -0.240714
C 1.033235 5.234627 0.671831
C 2.113320 6.279873 0.944716
O 1.670604 1.686592 -0.273325
O 3.908482 2.109596 -1.607386
O -2.402533 0.549430 1.263606
O -3.777312 1.195398 -1.008630
H 4.894236 -0.139645 -2.700231
H 4.571747 -0.402836 -0.984324
H 2.962279 -1.484939 -3.355111
H 3.889076 -2.425146 -2.200705
H 2.717318 1.045627 -2.917345
H 0.344296 -0.237751 0.952364
H 2.017614 -3.689417 -1.775990
H -0.555355 -4.933006 -1.070907
H 0.753944 -5.242965 0.072728
H -2.974902 -4.403309 1.153744
H 0.453294 -4.457890 2.536887
H -0.776462 -5.962326 4.000815
H -2.186102 -6.003930 2.804987
H -1.761193 -0.689535 -1.036982
H -0.634881 0.517682 -1.611075
H -0.858887 1.730103 0.557823
H -2.155273 2.272176 -1.559827
H -3.793017 3.757348 -0.493769
H -3.621365 2.905364 1.032966
H -2.385836 5.031807 1.068523
H -1.294684 3.684511 1.269380
H -0.593466 3.868133 -1.117800
H -1.792993 5.117963 -1.381989

H 0.377163 6.156711 -1.152613
H -0.583088 6.599453 0.245740
H 0.606951 4.900640 1.622031
H 1.482610 4.348826 0.212415
H 1.691886 7.164869 1.429292
H 2.896425 5.885254 1.595991
H 2.586019 6.606552 0.014463
H 3.318218 2.615895 -1.022994
H -2.183591 -0.393333 1.405033
H -4.019795 0.703934 -0.209153

2d-c456, ΔG = 2.3758 kcal/mol, population = 0.36 %
C 5.400826 -0.851085 -0.081093
C 4.595279 -1.817618 -0.946790
C 3.123627 -1.703293 -0.683125
C 2.527304 -0.576983 -0.195437
C 3.341721 0.564821 0.136626
C 4.824422 0.546109 -0.216429
C 1.023220 -0.452266 -0.005741
N 2.371883 -2.826833 -0.948536
C 1.065102 -2.895163 -0.570455
C 0.412220 -1.822379 -0.055520
C 0.245384 -4.144308 -0.652521
C -1.023788 -3.777931 0.147448
C -0.908087 -2.233577 0.340489
O -1.839761 -1.558937 0.758571
O -2.187448 -4.072205 -0.610819
C -1.017979 -4.433228 1.505354
C -2.020962 -5.136071 2.013734
C 0.435934 0.499696 -1.080213
C -0.845156 1.247579 -0.699346
C -0.693271 2.090475 0.576564
C -1.924464 2.924413 0.904670
C -3.192970 2.101943 1.126482
C -4.363878 2.925463 1.674911
C -4.798942 4.104258 0.797514
C -5.243124 3.712177 -0.611831
C -5.748860 4.903924 -1.422919
O 2.887795 1.584401 0.674922
O 5.532746 1.473265 0.584478
O -1.241391 2.073497 -1.805766
O 0.411475 2.998300 0.439883
H 6.447351 -0.855331 -0.385745
H 5.352198 -1.155761 0.966893

H 4.769947 -1.605225 -2.007596
H 4.918953 -2.845413 -0.775635
H 4.879192 0.850039 -1.274652
H 0.848418 -0.019252 0.980342
H 2.837709 -3.656060 -1.287706
H -0.024482 -4.362462 -1.687559
H 0.772288 -5.010084 -0.249727
H -2.908756 -3.544295 -0.238511
H -0.109937 -4.275983 2.080819
H -1.953226 -5.570170 3.003007
H -2.932640 -5.309471 1.455886
H 0.244652 -0.071207 -1.990630
H 1.191089 1.245611 -1.336893
H -1.656014 0.537400 -0.550294
H -0.508328 1.410633 1.414601
H -1.698311 3.496550 1.809434
H -2.075990 3.648544 0.101965
H -3.490376 1.621522 0.191661
H -2.978296 1.290109 1.828336
H -5.220922 2.259736 1.822187
H -4.095383 3.304836 2.666497
H -5.624720 4.622665 1.296609
H -3.986315 4.834458 0.725971
H -4.411602 3.242507 -1.144333
H -6.030125 2.953185 -0.542329
H -6.055984 4.604193 -2.427300
H -6.607803 5.374296 -0.936857
H -4.970016 5.664402 -1.525601
H 4.871670 2.118549 0.885210
H -0.616563 2.811040 -1.837272
H 1.239800 2.495474 0.537067

2d-c494, ΔG = 2.3801 kcal/mol, population = 0.35 %
C 5.192994 -0.299415 0.359859
C 4.667566 -1.421314 -0.533694
C 3.173369 -1.527052 -0.470388
C 2.353136 -0.489025 -0.136611
C 2.933123 0.781071 0.222025
C 4.435738 0.980585 0.060027
C 0.836057 -0.595843 -0.145376
N 2.642242 -2.764961 -0.760074
C 1.320352 -3.021216 -0.555299
C 0.449437 -2.044696 -0.195240
C 0.716321 -4.384517 -0.673348

C -0.705405 -4.187137 -0.102842
C -0.839198 -2.638291 0.039457
O -1.906677 -2.097966 0.298855
O -1.672066 -4.656985 -1.031982
C -0.838546 -4.814483 1.260875
C -1.792578 -5.664158 1.616146
C 0.256947 0.210864 -1.336783
C -1.152179 0.781889 -1.151736
C -1.280141 1.682123 0.085041
C -2.676373 2.267749 0.237780
C -2.871387 3.019062 1.553034
C -4.291416 3.561533 1.751974
C -4.776701 4.534348 0.671298
C -3.905542 5.779569 0.504097
C -4.471670 6.760090 -0.521551
O 2.262955 1.739042 0.632367
O 4.882067 2.044627 0.879423
O -1.510600 1.507802 -2.338918
O -0.340177 2.765060 0.002226
H 6.259513 -0.151113 0.190473
H 5.050578 -0.558866 1.411509
H 4.953700 -1.235836 -1.575200
H 5.114134 -2.375912 -0.251103
H 4.585563 1.238977 -1.001122
H 0.470530 -0.159718 0.785344
H 3.268456 -3.524219 -0.985744
H 0.652899 -4.692151 -1.718800
H 1.293937 -5.136655 -0.134637
H -2.516394 -4.245726 -0.796927
H -0.082236 -4.506291 1.977366
H -1.833575 -6.070116 2.618714
H -2.551839 -5.989620 0.916215
H 0.260811 -0.420984 -2.226765
H 0.924862 1.047871 -1.550612
H -1.877189 -0.026411 -1.062833
H -1.065861 1.080776 0.974150
H -2.871036 2.924160 -0.612172
H -3.399764 1.448428 0.172506
H -2.635553 2.346107 2.384381
H -2.148306 3.835078 1.608204
H -4.989688 2.719214 1.805086
H -4.341290 4.062319 2.724827
H -4.847785 4.017109 -0.290933
H -5.796581 4.848206 0.918226

H -3.802619 6.279039 1.473769
H -2.896208 5.486264 0.202729
H -5.469195 7.100116 -0.230738
H -3.835678 7.641676 -0.627648
H -4.556698 6.289989 -1.505022
H 4.096318 2.589242 1.052366
H -1.005687 2.332772 -2.315322
H 0.540536 2.423636 0.237700

2d-c378, ΔG = 2.4272 kcal/mol, population = 0.33 %
C 4.158942 0.088286 -1.567315
C 3.446475 -1.186449 -2.015785
C 2.188154 -1.433119 -1.237076
C 1.489647 -0.458757 -0.592989
C 1.999554 0.898275 -0.600844
C 3.163905 1.235085 -1.525400
C 0.183634 -0.712331 0.144739
N 1.759787 -2.748975 -1.196850
C 0.705667 -3.110540 -0.426059
C -0.022395 -2.191042 0.262159
C 0.245097 -4.522218 -0.243190
C -0.834018 -4.402816 0.859139
C -1.032975 -2.868155 1.007573
O -1.955874 -2.382961 1.665028
O -2.045226 -4.995160 0.411821
C -0.344610 -4.965298 2.169767
C -0.981348 -5.879847 2.888135
C -1.012805 -0.005552 -0.564807
C -1.766642 0.968445 0.332788
C -2.694696 1.891009 -0.464762
C -3.212454 3.080453 0.348213
C -2.124340 3.970736 0.959866
C -1.077882 4.476095 -0.035381
C -0.051901 5.406748 0.611088
C 1.106649 5.802001 -0.309491
C 0.683572 6.612141 -1.534864
O 1.520776 1.795081 0.094020
O 3.774703 2.444330 -1.115322
O -2.589642 0.310210 1.309027
O -3.789793 1.129314 -0.994091
H 4.974699 0.324352 -2.250814
H 4.583750 -0.049575 -0.570113
H 3.182475 -1.114652 -3.077030
H 4.109246 -2.048013 -1.916403

H 2.727884 1.362436 -2.528805
H 0.272724 -0.280424 1.146281
H 2.301604 -3.454741 -1.674018
H -0.209939 -4.904671 -1.158500
H 1.065630 -5.186687 0.029751
H -2.755627 -4.658497 0.976503
H 0.598402 -4.552146 2.516447
H -0.574077 -6.235002 3.825988
H -1.918784 -6.310318 2.558845
H -1.703107 -0.750374 -0.966858
H -0.630378 0.552414 -1.421153
H -1.022629 1.581865 0.846077
H -2.147585 2.260731 -1.332746
H -3.844654 3.676350 -0.316810
H -3.859718 2.699266 1.143041
H -2.608808 4.829708 1.435056
H -1.616937 3.431688 1.765803
H -0.542146 3.629653 -0.474626
H -1.584580 4.985923 -0.860824
H -0.555323 6.311746 0.971134
H 0.357921 4.910541 1.497289
H 1.834486 6.380148 0.267785
H 1.625416 4.894155 -0.635844
H 1.551850 6.923853 -2.119513
H 0.032677 6.035506 -2.194764
H 0.139849 7.513418 -1.237766
H 3.128127 2.872051 -0.527414
H -2.294848 -0.611535 1.448727
H -4.073722 0.562100 -0.261074

2d-c490, ΔG = 2.4686 kcal/mol, population = 0.30 %
C 4.880701 -1.210245 1.269385
C 4.233036 -2.092161 0.204484
C 2.812621 -1.692503 -0.061258
C 2.324569 -0.435270 0.151176
C 3.192280 0.563712 0.722955
C 4.672869 0.247655 0.905366
C 0.896878 -0.027164 -0.188572
N 1.989827 -2.687455 -0.537949
C 0.651664 -2.478729 -0.680907
C 0.088654 -1.261599 -0.473172
C -0.312082 -3.560201 -1.056701
C -1.685496 -2.890314 -0.839948
C -1.335087 -1.382504 -0.651478

O -2.194165 -0.512200 -0.681140
O -2.498874 -3.043265 -1.993339
C -2.345973 -3.386153 0.421526
C -3.597367 -3.817474 0.502657
C 0.889784 0.972999 -1.375883
C -0.234641 2.014049 -1.372159
C -0.228656 2.906053 -0.122663
C -1.345465 3.944516 -0.109290
C -2.763122 3.370325 -0.181959
C -3.106765 2.389377 0.939659
C -4.560210 1.917651 0.888150
C -4.888764 0.795615 1.877430
C -4.726795 1.191377 3.345452
O 2.800052 1.690514 1.058099
O 5.241988 1.105612 1.876189
O -0.120725 2.824171 -2.552748
O 1.019536 3.614984 -0.040388
H 5.945679 -1.429803 1.344821
H 4.425777 -1.402022 2.244049
H 4.790280 -2.013823 -0.735934
H 4.267607 -3.141151 0.502222
H 5.137337 0.436722 -0.076325
H 0.481936 0.473108 0.688095
H 2.367987 -3.615294 -0.664365
H -0.199345 -3.832921 -2.107587
H -0.172232 -4.459913 -0.456221
H -3.165545 -2.341403 -1.962754
H -1.719015 -3.348466 1.307879
H -4.014622 -4.146359 1.445711
H -4.237574 -3.866697 -0.369043
H 0.833566 0.411487 -2.310184
H 1.841777 1.506900 -1.395752
H -1.195491 1.508509 -1.440940
H -0.335107 2.271783 0.760067
H -1.234977 4.525026 0.811161
H -1.186346 4.634468 -0.941292
H -3.470145 4.205780 -0.154102
H -2.916969 2.880303 -1.148028
H -2.461580 1.510244 0.872188
H -2.898885 2.863692 1.904065
H -5.228062 2.768764 1.067387
H -4.776677 1.565354 -0.125842
H -5.918368 0.465480 1.708016
H -4.250040 -0.066314 1.658215

H -5.336045 2.068140 3.582824
H -5.036680 0.380715 4.008698
H -3.689916 1.434692 3.585469
H 4.653192 1.877485 1.919033
H 0.618859 3.427554 -2.393120
H 1.677380 2.999624 0.328071

2d-c52, ΔG = 2.4856 kcal/mol, population = 0.30 %
C 4.381549 -0.897100 -2.266860
C 2.889085 -0.843105 -2.579730
C 2.045503 -0.880067 -1.339906
C 2.471884 -0.490609 -0.107016
C 3.867238 -0.124471 0.054497
C 4.708245 0.127037 -1.194294
C 1.581061 -0.500609 1.134600
N 0.759807 -1.354326 -1.524884
C -0.094954 -1.482287 -0.481713
C 0.244057 -1.096453 0.778149
C -1.489861 -2.003629 -0.624738
C -1.996871 -2.047897 0.832952
C -0.913740 -1.246794 1.611218
O -1.137240 -0.779320 2.727373
O -3.242879 -1.380355 0.945236
C -2.031259 -3.458915 1.364528
C -3.068712 -4.007528 1.981699
C 1.505969 0.886070 1.823058
C 0.968172 2.057473 0.998952
C -0.549055 2.021278 0.775704
C -1.042651 3.134285 -0.137116
C -2.530336 3.037349 -0.485838
C -2.924018 1.727119 -1.168582
C -4.363354 1.712637 -1.678619
C -4.757476 0.383442 -2.322711
C -6.193242 0.370656 -2.843844
O 4.408145 -0.005832 1.154319
O 6.083153 0.111898 -0.863427
O 1.330833 3.291213 1.639143
O -1.227802 2.135666 2.040344
H 4.963926 -0.697258 -3.166418
H 4.654352 -1.890538 -1.902671
H 2.652398 0.080890 -3.119711
H 2.606264 -1.668561 -3.235428
H 4.425299 1.127006 -1.558579
H 2.054977 -1.151633 1.877054

H 0.473552 -1.644415 -2.448428
H -2.105066 -1.313839 -1.204517
H -1.510087 -2.974156 -1.122043
H -3.311681 -1.065890 1.859085
H -1.113064 -4.021125 1.220710
H -3.026205 -5.024935 2.348666
H -3.992639 -3.462265 2.127421
H 2.516722 1.148991 2.127281
H 0.920611 0.782066 2.737834
H 1.461042 2.074199 0.023904
H -0.798788 1.066046 0.318147
H -0.829800 4.098120 0.330676
H -0.451894 3.092876 -1.057940
H -3.125066 3.163160 0.422871
H -2.785702 3.875330 -1.142429
H -2.241885 1.534452 -2.005695
H -2.800443 0.895913 -0.469885
H -5.044083 1.924772 -0.845983
H -4.503444 2.523223 -2.403158
H -4.067333 0.165984 -3.145211
H -4.625308 -0.419617 -1.590919
H -6.343517 1.146258 -3.599595
H -6.446521 -0.590241 -3.297274
H -6.904858 0.555277 -2.034684
H 6.112840 0.186185 0.106458
H 0.717124 3.395628 2.380335
H -1.218453 1.266480 2.469351

2d-c22, ΔG = 2.5320 kcal/mol, population = 0.27 %
C 5.140578 -1.125684 -1.148238
C 3.826852 -1.197395 -1.922097
C 2.630675 -1.115574 -1.021243
C 2.650730 -0.575326 0.227448
C 3.903470 -0.076733 0.755484
C 5.094225 0.049609 -0.188386
C 1.406873 -0.434828 1.094546
N 1.460109 -1.631139 -1.548472
C 0.330468 -1.697796 -0.799229
C 0.278883 -1.200464 0.463111
C -0.946130 -2.310543 -1.281717
C -1.864136 -2.247574 -0.041686
C -1.034530 -1.427496 0.996962
O -1.517609 -1.040189 2.054076
O -3.043796 -1.517412 -0.357110

C -2.148659 -3.616639 0.515700
C -3.356513 -4.088590 0.793558
C 1.005120 1.045345 1.306373
C 0.807244 1.825050 0.008407
C -0.334747 2.848319 0.086502
C -1.700374 2.178210 0.097909
C -2.861941 3.152134 0.293305
C -4.227150 2.462323 0.375306
C -4.663609 1.786136 -0.927174
C -6.095065 1.239836 -0.895831
C -6.295210 0.063404 0.060517
O 4.044311 0.274888 1.927850
O 6.294512 0.163189 0.552743
O 2.022902 2.483480 -0.408196
O -0.268844 3.718247 -1.049376
H 5.978495 -1.015057 -1.836768
H 5.292380 -2.044950 -0.577446
H 3.767280 -0.367537 -2.635839
H 3.777991 -2.116469 -2.509036
H 4.922665 0.972570 -0.764387
H 1.619997 -0.845826 2.085117
H 1.476668 -2.038627 -2.471702
H -1.379059 -1.720218 -2.091291
H -0.793880 -3.325856 -1.650629
H -3.408903 -1.188060 0.476754
H -1.266201 -4.221090 0.706580
H -3.486710 -5.080926 1.205880
H -4.249973 -3.505735 0.608147
H 1.767083 1.541798 1.910080
H 0.089889 1.052950 1.897531
H 0.579551 1.147770 -0.817640
H -0.207250 3.443358 1.003783
H -1.812191 1.631719 -0.842246
H -1.729025 1.435229 0.897451
H -2.692141 3.717159 1.215873
H -2.870050 3.881566 -0.521334
H -4.205871 1.730671 1.189265
H -4.982734 3.205845 0.651171
H -4.580944 2.516147 -1.739876
H -3.977407 0.970633 -1.172625
H -6.781139 2.050311 -0.626092
H -6.375248 0.925610 -1.905820
H -7.327750 -0.291863 0.037199
H -5.651492 -0.775199 -0.216752

H -6.065136 0.335710 1.092447
H 6.017657 0.416217 1.450167
H 2.417378 2.910811 0.362969
H 0.675923 3.848999 -1.219676

2d-c212, ΔG = 2.5326 kcal/mol, population = 0.27 %
C 3.970902 -0.278766 -2.110655
C 3.141057 -1.532549 -2.380853
C 1.968990 -1.644539 -1.451376
C 1.406662 -0.587378 -0.805644
C 1.987294 0.731026 -0.969557
C 3.053489 0.929562 -2.041115
C 0.179503 -0.704335 0.085815
N 1.471218 -2.923745 -1.270782
C 0.494212 -3.164254 -0.363840
C -0.095073 -2.156480 0.334725
C -0.021991 -4.528496 -0.031386
C -0.950265 -4.270097 1.178636
C -1.043607 -2.719498 1.239724
O -1.851246 -2.137101 1.966444
O -2.238269 -4.810807 0.920529
C -0.336899 -4.777578 2.459196
C -0.931695 -5.600606 3.311687
C -1.044090 0.028889 -0.547034
C -1.668811 1.087048 0.356120
C -2.626734 2.003046 -0.410432
C -3.081163 3.220390 0.398436
C -1.957275 4.134970 0.899393
C -1.024597 4.654017 -0.196609
C -0.027845 5.693323 0.317229
C 0.915607 6.245582 -0.756162
C 1.913706 5.219662 -1.294517
O 1.639844 1.700323 -0.295352
O 3.777500 2.119857 -1.793237
O -2.427882 0.525787 1.437196
O -3.765757 1.247715 -0.851320
H 4.713150 -0.141218 -2.897155
H 4.500933 -0.376770 -1.160220
H 2.760762 -1.516043 -3.408580
H 3.761329 -2.426402 -2.292015
H 2.510485 1.017132 -2.995662
H 0.404240 -0.210478 1.036104
H 1.914125 -3.693308 -1.751286
H -0.606573 -4.934813 -0.858593

H 0.785153 -5.228057 0.188281
H -2.854706 -4.393330 1.539097
H 0.663443 -4.405303 2.661402
H -0.433412 -5.920205 4.217863
H -1.925191 -5.989832 3.127405
H -1.802052 -0.699470 -0.843022
H -0.717770 0.522006 -1.464359
H -0.856493 1.696651 0.761420
H -2.126903 2.338168 -1.320171
H -3.760135 3.795538 -0.238100
H -3.668499 2.866310 1.250006
H -2.415399 4.987580 1.410724
H -1.364249 3.614690 1.658045
H -0.480022 3.817372 -0.639902
H -1.622651 5.096819 -1.002038
H -0.585576 6.523163 0.763886
H 0.567193 5.252598 1.125891
H 0.320525 6.646689 -1.583821
H 1.468295 7.092513 -0.338643
H 1.411511 4.377247 -1.772739
H 2.584133 5.668715 -2.030308
H 2.527299 4.821009 -0.481581
H 3.214843 2.646172 -1.199644
H -2.138759 -0.387931 1.631455
H -4.036503 0.729862 -0.078477

2d-c89, ΔG = 2.5527 kcal/mol, population = 0.26 %
C 3.903267 0.034834 -1.742638
C 2.969972 -1.094273 -2.172747
C 1.851140 -1.309558 -1.196495
C 1.407006 -0.364765 -0.323060
C 2.079191 0.918657 -0.279729
C 3.076060 1.256413 -1.382730
C 0.240986 -0.580059 0.633169
N 1.268801 -2.563294 -1.232921
C 0.281046 -2.896693 -0.367603
C -0.203012 -2.013411 0.546294
C -0.387423 -4.236294 -0.347095
C -1.277307 -4.163266 0.902027
C -1.247032 -2.659303 1.283211
O -2.051565 -2.179856 2.085120
O -2.612454 -4.546508 0.562877
C -0.797222 -4.951306 2.097847
C 0.387780 -5.523484 2.264023

C -0.934773 0.397113 0.333043
C -1.337961 1.263316 1.517777
C -2.535420 2.164873 1.192061
C -2.267723 3.216725 0.120875
C -1.062012 4.120112 0.386506
C -0.912661 5.252980 -0.634546
C -0.715542 4.798854 -2.085852
C 0.490565 3.883907 -2.301524
C 0.714518 3.542724 -3.773162
O 1.862881 1.758125 0.594988
O 3.888243 2.344903 -0.988991
O -1.658352 0.494395 2.689399
O -2.954383 2.831006 2.391629
H 4.598734 0.276505 -2.546570
H 4.487396 -0.269947 -0.871026
H 2.527669 -0.863059 -3.148579
H 3.527369 -2.024702 -2.296045
H 2.473001 1.542672 -2.257749
H 0.588103 -0.370652 1.648396
H 1.621244 -3.248599 -1.885053
H -1.020632 -4.356017 -1.227821
H 0.326751 -5.058093 -0.339656
H -3.195460 -4.187894 1.247802
H -1.530032 -4.993583 2.898907
H 0.625499 -6.043547 3.183263
H 1.166754 -5.495347 1.512425
H -1.800820 -0.173471 -0.013999
H -0.653369 1.050190 -0.492528
H -0.491039 1.887836 1.803216
H -3.352980 1.515440 0.847210
H -2.155189 2.707108 -0.838283
H -3.169774 3.830524 0.030876
H -1.158695 4.553356 1.384820
H -0.146221 3.525533 0.398031
H -1.794762 5.900232 -0.583738
H -0.058885 5.873611 -0.342517
H -1.617058 4.291449 -2.444463
H -0.601844 5.687045 -2.716176
H 1.386740 4.362137 -1.892043
H 0.356275 2.960274 -1.733162
H 0.875234 4.447603 -4.364907
H 1.585753 2.898309 -3.910552
H -0.152113 3.023236 -4.190519
H 3.415721 2.761814 -0.247867

H -1.924374 -0.413234 2.437617
H -2.886416 2.159010 3.086283

2d-c123, ΔG = 2.5797 kcal/mol, population = 0.25 %
C 5.169364 -0.915475 -1.247384
C 3.835851 -1.033316 -1.981238
C 2.669941 -1.051463 -1.037939
C 2.703202 -0.546376 0.225439
C 3.941956 0.009911 0.727292
C 5.093776 0.225360 -0.248323
C 1.484767 -0.504441 1.136653
N 1.514624 -1.626604 -1.536713
C 0.427394 -1.802010 -0.742392
C 0.395943 -1.347088 0.536760
C -0.821030 -2.497113 -1.186800
C -1.654535 -2.590669 0.109744
C -0.866145 -1.702051 1.122352
O -1.335794 -1.378992 2.206601
O -2.943322 -2.028120 -0.096933
C -1.702939 -4.002405 0.634176
C -2.808909 -4.645202 0.983695
C 0.985933 0.942270 1.371351
C 0.700544 1.711658 0.081927
C -0.557603 2.586940 0.159523
C -1.829957 1.753982 0.184359
C -3.108585 2.579118 0.307721
C -4.359222 1.701439 0.355071
C -5.665391 2.479152 0.546902
C -5.988035 3.497784 -0.553078
C -6.112107 2.884930 -1.947899
O 4.100635 0.339251 1.903853
O 6.309726 0.377149 0.460252
O 1.829036 2.516500 -0.321820
O -0.601928 3.448199 -0.983993
H 5.976812 -0.738901 -1.958237
H 5.387749 -1.842826 -0.712408
H 3.706334 -0.183273 -2.661165
H 3.817797 -1.932749 -2.599489
H 4.859670 1.155890 -0.788989
H 1.761950 -0.904183 2.115608
H 1.520806 -2.003109 -2.473216
H -1.356164 -1.895050 -1.923433
H -0.609871 -3.469172 -1.634313
H -3.280394 -1.762294 0.770987

H -0.733428 -4.482526 0.734015
H -2.769601 -5.657055 1.366239
H -3.785110 -4.186901 0.887411
H 1.730128 1.486433 1.955615
H 0.089816 0.884800 1.988492
H 0.560137 1.023834 -0.754249
H -0.501635 3.200665 1.071613
H -1.863733 1.152807 -0.730474
H -1.780672 1.051360 1.018272
H -3.060137 3.188888 1.217452
H -3.168812 3.275884 -0.530312
H -4.412783 1.102827 -0.559565
H -4.256672 0.984254 1.176300
H -6.492728 1.764527 0.616558
H -5.627731 3.000148 1.509683
H -6.926921 3.997144 -0.295604
H -5.224997 4.281308 -0.567416
H -6.419388 3.634224 -2.680609
H -5.164901 2.461723 -2.288480
H -6.855312 2.082733 -1.957850
H 6.048493 0.585858 1.373626
H 2.160735 2.987128 0.453643
H 0.318296 3.699177 -1.153428

2d-c276, ΔG = 2.6782 kcal/mol, population = 0.21 %
C 3.524869 0.260316 -2.178756
C 2.678983 -0.978139 -2.466111
C 1.711708 -1.279098 -1.358616
C 1.277453 -0.360247 -0.453822
C 1.817117 0.984590 -0.506959
C 2.623895 1.398260 -1.732012
C 0.246124 -0.663340 0.625155
N 1.262934 -2.586953 -1.303213
C 0.431776 -2.996097 -0.314235
C -0.031913 -2.137516 0.633411
C -0.065646 -4.400175 -0.173555
C -0.839661 -4.369115 1.164047
C -0.902906 -2.855089 1.511369
O -1.643580 -2.420191 2.395029
O -2.156913 -4.863308 0.970964
C -0.087055 -5.090148 2.254251
C -0.597447 -6.039240 3.026665
C -1.059175 0.159514 0.405270
C -1.417656 1.069724 1.569503

C -2.668980 1.909993 1.292902
C -2.564427 2.823182 0.075208
C -1.334763 3.733995 0.029504
C -1.111468 4.356279 -1.348145
C 0.143977 5.227268 -1.403809
C 0.509745 5.727665 -2.804762
C 0.978390 4.618726 -3.748937
O 1.634113 1.814068 0.384285
O 3.364627 2.570864 -1.454912
O -1.622496 0.352105 2.799307
O -2.953240 2.711751 2.449906
H 4.081883 0.550641 -3.069652
H 4.245624 0.047849 -1.385559
H 2.102814 -0.833521 -3.387269
H 3.319511 -1.846167 -2.633293
H 1.888155 1.604560 -2.525216
H 0.662189 -0.367924 1.592009
H 1.603413 -3.251955 -1.982407
H -0.751475 -4.651863 -0.984331
H 0.749865 -5.124351 -0.183139
H -2.693749 -4.541783 1.709682
H 0.940484 -4.764230 2.388140
H -0.002399 -6.510936 3.798021
H -1.617331 -6.382024 2.905553
H -1.891832 -0.519957 0.202109
H -0.942274 0.768469 -0.490431
H -0.580570 1.743128 1.755939
H -3.504691 1.213924 1.133468
H -2.578125 2.190818 -0.816705
H -3.476744 3.424849 0.027651
H -1.426804 4.518101 0.786231
H -0.438370 3.166387 0.282565
H -1.031593 3.546399 -2.080155
H -1.983214 4.951952 -1.642812
H 0.009031 6.084371 -0.736023
H 0.989063 4.656693 -1.002163
H -0.350351 6.246643 -3.241959
H 1.304219 6.474716 -2.715756
H 1.834133 4.087836 -3.323615
H 0.191559 3.885206 -3.934701
H 1.281451 5.025827 -4.716123
H 2.952874 2.953652 -0.661029
H -1.747586 -0.600819 2.621309
H -2.848449 2.116701 3.206074

2d-c129, ΔG = 2.7322 kcal/mol, population = 0.20 %
C 4.374108 -1.388480 -2.180416
C 2.924975 -1.255023 -2.644889
C 1.999456 -0.942889 -1.507011
C 2.388492 -0.352391 -0.345026
C 3.793992 -0.061157 -0.135957
C 4.745434 -0.192409 -1.321420
C 1.394996 0.052184 0.735883
N 0.678354 -1.326706 -1.686717
C -0.176264 -1.343602 -0.634998
C 0.151982 -0.768603 0.554694
C -1.498554 -2.040261 -0.639739
C -1.932863 -1.990092 0.843706
C -0.850922 -1.098719 1.515257
O -0.931266 -0.745083 2.693871
O -3.192505 -1.336980 0.942744
C -1.919183 -3.359378 1.471272
C -2.942917 -3.913602 2.105598
C 1.181325 1.584796 0.641057
C 0.265223 2.244607 1.687197
C -1.221199 2.310529 1.287578
C -1.479574 3.273148 0.137360
C -2.951596 3.366224 -0.276335
C -3.558355 2.069262 -0.820708
C -2.853205 1.518979 -2.061660
C -3.655170 0.428791 -2.771672
C -2.943290 -0.141466 -3.996433
O 4.249813 0.304551 0.947181
O 6.082161 -0.278520 -0.866015
O 0.401763 1.661082 2.991008
O -1.984649 2.754944 2.417756
H 5.041097 -1.452739 -3.040241
H 4.496325 -2.299852 -1.590312
H 2.840276 -0.449928 -3.383645
H 2.598855 -2.170413 -3.141461
H 4.613038 0.725419 -1.916199
H 1.835227 -0.161982 1.711113
H 0.429212 -1.808140 -2.538972
H -2.223766 -1.502708 -1.250738
H -1.420290 -3.056427 -1.027383
H -3.321756 -1.088459 1.868955
H -0.974743 -3.888634 1.382405
H -2.860991 -4.901245 2.540666

H -3.895152 -3.407021 2.200139
H 0.816722 1.821242 -0.359903
H 2.170130 2.036924 0.727121
H 0.599938 3.276626 1.817943
H -1.560679 1.309438 1.005398
H -1.125771 4.263681 0.439254
H -0.878487 2.973650 -0.722712
H -3.538555 3.706301 0.579692
H -3.040750 4.142849 -1.042900
H -3.570704 1.299539 -0.043600
H -4.606497 2.264286 -1.070520
H -2.666002 2.337729 -2.766597
H -1.868530 1.123982 -1.789492
H -3.881580 -0.375560 -2.063920
H -4.623702 0.841036 -3.072703
H -2.747913 0.641863 -4.733294
H -1.980608 -0.582514 -3.726354
H -3.539585 -0.916614 -4.482245
H 6.065348 0.055293 0.047196
H 0.042191 0.748997 2.968215
H -1.524868 2.403214 3.194609

2d-c313, ΔG = 2.7391 kcal/mol, population = 0.19 %
C 4.617688 -0.351560 1.022323
C 4.225892 -1.302338 -0.107031
C 2.737245 -1.405170 -0.260418
C 1.862681 -0.444037 0.141137
C 2.370802 0.735146 0.814071
C 3.878504 0.963577 0.852859
C 0.359904 -0.525142 -0.087162
N 2.273648 -2.572800 -0.841541
C 0.947123 -2.840290 -0.890180
C 0.015284 -1.930619 -0.489682
C 0.380274 -4.144926 -1.352863
C -1.111533 -4.033964 -0.964947
C -1.274073 -2.538762 -0.575628
O -2.383750 -2.031441 -0.398233
O -1.928147 -4.323270 -2.089771
C -1.429500 -4.887730 0.237394
C -2.410795 -5.777239 0.296840
C -0.086533 0.544205 -1.131001
C -1.328153 1.344776 -0.734915
C -1.445271 2.644205 -1.534114
C -2.602872 3.537227 -1.081869

C -2.585081 3.952260 0.392121
C -1.326492 4.709975 0.820147
C -1.425211 5.323679 2.221299
C -1.665095 4.322537 3.357827
C -0.559366 3.278160 3.505134
O 1.634624 1.571127 1.338105
O 4.204650 1.878793 1.881871
O -2.551522 0.638428 -0.957219
O -1.576249 2.344752 -2.934203
H 5.694271 -0.180518 1.015102
H 4.351249 -0.786684 1.988535
H 4.643573 -0.948874 -1.056638
H 4.643702 -2.295321 0.067510
H 4.143473 1.394585 -0.125995
H -0.136150 -0.286972 0.858550
H 2.939552 -3.282742 -1.110158
H 0.468212 -4.242708 -2.436347
H 0.882323 -4.997018 -0.893639
H -2.807524 -3.962335 -1.906840
H -0.789285 -4.715868 1.098021
H -2.589139 -6.351495 1.196815
H -3.056532 -5.968370 -0.551017
H -0.243804 0.070528 -2.101732
H 0.732158 1.253538 -1.261356
H -1.233131 1.602981 0.325905
H -0.503405 3.186491 -1.440245
H -2.586282 4.431328 -1.712266
H -3.537270 3.012944 -1.297454
H -3.457961 4.586978 0.577259
H -2.720034 3.069114 1.021436
H -0.457908 4.047742 0.771023
H -1.134579 5.512678 0.100070
H -0.503326 5.877506 2.429675
H -2.234984 6.061343 2.224537
H -1.760587 4.880857 4.294134
H -2.625105 3.819474 3.209840
H 0.410867 3.757083 3.664882
H -0.750728 2.621618 4.357032
H -0.465931 2.650527 2.618214
H 3.374967 2.343705 2.085100
H -2.470087 -0.289404 -0.657140
H -2.267823 1.670220 -2.991555

2d-c424, ΔG = 2.7523 kcal/mol, population = 0.19 %

C 3.901304 -1.283531 -2.605843
C 2.429110 -0.991412 -2.890317
C 1.656128 -0.736257 -1.630977
C 2.211554 -0.299569 -0.468625
C 3.651750 -0.142150 -0.389777
C 4.461414 -0.217370 -1.680689
C 1.373618 0.070956 0.748514
N 0.295490 -1.003955 -1.697818
C -0.441819 -1.068665 -0.562587
C 0.049863 -0.626521 0.627788
C -1.796549 -1.695821 -0.477526
C -2.056259 -1.772774 1.045229
C -0.863299 -0.990805 1.663371
O -0.799186 -0.737544 2.868064
O -3.266248 -1.092540 1.353250
C -2.028982 -3.196484 1.537429
C -2.998946 -3.779355 2.227984
C 1.323593 1.618535 0.836259
C 0.493677 2.259635 1.957194
C -1.000200 2.459628 1.652465
C -1.258237 3.372737 0.449811
C -1.618627 2.650313 -0.852552
C -3.003506 2.003964 -0.836075
C -3.359864 1.327496 -2.158738
C -4.709564 0.611673 -2.131245
C -5.035041 -0.099782 -3.442912
O 4.246801 0.066457 0.666985
O 5.825424 -0.451913 -1.387700
O 0.656546 1.598860 3.219428
O -1.608438 3.061321 2.808115
H 4.467689 -1.305547 -3.536982
H 4.003942 -2.259435 -2.125461
H 2.339733 -0.105634 -3.529479
H 1.975699 -1.819133 -3.438073
H 4.348756 0.766264 -2.164061
H 1.882248 -0.288225 1.645288
H -0.080029 -1.381894 -2.555939
H -2.555080 -1.070478 -0.946994
H -1.818540 -2.671227 -0.964131
H -3.274250 -0.927672 2.306821
H -1.122372 -3.742532 1.292810
H -2.909462 -4.806674 2.556715
H -3.913967 -3.256592 2.476378
H 0.994497 2.018736 -0.122784

H 2.357064 1.942509 0.965053
H 0.897327 3.262505 2.120674
H -1.473564 1.489639 1.481141
H -2.085908 4.032062 0.721175
H -0.390696 4.020180 0.291603
H -1.574486 3.372520 -1.673208
H -0.871706 1.886036 -1.086013
H -3.066444 1.268357 -0.028974
H -3.753600 2.769369 -0.608907
H -3.361071 2.073830 -2.960832
H -2.575333 0.609081 -2.424117
H -4.717164 -0.112491 -1.309783
H -5.495444 1.338202 -1.900386
H -6.005129 -0.599246 -3.397618
H -5.061236 0.606717 -4.276604
H -4.280529 -0.856562 -3.674127
H 5.931072 -0.221419 -0.448880
H 0.240928 0.712721 3.166868
H -1.183912 2.645177 3.573078

2d-c97, ΔG = 2.7598 kcal/mol, population = 0.19 %
C 3.974840 0.086265 -1.692173
C 3.105533 -1.077121 -2.162752
C 1.955157 -1.330939 -1.234198
C 1.428009 -0.394517 -0.398981
C 2.031839 0.922726 -0.351783
C 3.089188 1.281577 -1.390979
C 0.240270 -0.657220 0.516829
N 1.438343 -2.613394 -1.270102
C 0.445890 -2.988231 -0.427513
C -0.111190 -2.117251 0.456920
C -0.137722 -4.365222 -0.394950
C -1.092216 -4.314411 0.818456
C -1.133583 -2.804124 1.185094
O -1.972575 -2.351216 1.966098
O -2.389932 -4.741960 0.432081
C -0.535919 -5.085878 1.989339
C -1.188867 -6.030199 2.652427
C -0.986784 0.230220 0.150522
C -1.474248 1.106173 1.296562
C -2.753126 1.870881 0.941232
C -2.618099 2.840998 -0.226311
C -1.447965 3.820963 -0.138494
C -1.450659 4.827600 -1.287789

C -0.270518 5.804707 -1.264654
C 1.115201 5.154242 -1.365999
C 1.309435 4.303689 -2.620820
O 1.714949 1.773386 0.480458
O 3.846167 2.392225 -0.950171
O -1.713551 0.365644 2.505623
O -3.188530 2.604176 2.095954
H 4.706875 0.344988 -2.457400
H 4.517667 -0.192743 -0.785924
H 2.700287 -0.863952 -3.158473
H 3.702411 -1.985981 -2.257186
H 2.535410 1.549397 -2.304118
H 0.534917 -0.399318 1.537125
H 1.847921 -3.287992 -1.899735
H -0.711828 -4.563042 -1.301800
H 0.631365 -5.133492 -0.306831
H -3.009982 -4.405825 1.095319
H 0.471834 -4.804228 2.281258
H -0.732188 -6.541224 3.490175
H -2.190982 -6.329512 2.372489
H -1.803630 -0.408974 -0.196205
H -0.726669 0.873695 -0.689772
H -0.691666 1.822489 1.548718
H -3.521829 1.126396 0.688416
H -2.538192 2.260847 -1.149977
H -3.558326 3.396419 -0.298770
H -1.487220 4.355754 0.815126
H -0.506467 3.268192 -0.143236
H -1.460880 4.286294 -2.239443
H -2.383159 5.400836 -1.258855
H -0.386014 6.513178 -2.092179
H -0.317369 6.396618 -0.344253
H 1.869617 5.946528 -1.348881
H 1.303197 4.539274 -0.481693
H 1.082894 4.877568 -3.523739
H 2.339024 3.950151 -2.694764
H 0.659095 3.426473 -2.615399
H 3.304766 2.816382 -0.262659
H -1.939772 -0.561751 2.290287
H -3.060807 1.998387 2.840734

2d-c368, ΔG = 2.7880 kcal/mol, population = 0.18 %
C 4.517216 -1.204144 1.427898
C 3.983356 -2.054412 0.277942

C 2.580903 -1.673518 -0.091333
C 2.046174 -0.435527 0.123876
C 2.840787 0.556284 0.803699
C 4.307214 0.262218 1.102709
C 0.640168 -0.045318 -0.319651
N 1.825039 -2.667883 -0.668110
C 0.497800 -2.485151 -0.909401
C -0.110567 -1.288752 -0.706508
C -0.405590 -3.574541 -1.396452
C -1.808052 -2.947987 -1.248158
C -1.510534 -1.437315 -1.007549
O -2.384848 -0.586118 -1.096213
O -2.552782 -3.100028 -2.445798
C -2.520864 -3.486784 -0.032597
C -3.760419 -3.957757 -0.027354
C 0.702014 0.994536 -1.472571
C -0.416979 2.043112 -1.500364
C -0.462504 2.904861 -0.231244
C -1.576862 3.944839 -0.223608
C -2.997269 3.383775 -0.365457
C -3.337267 2.224820 0.578917
C -3.161684 2.529867 2.066849
C -3.396681 1.311224 2.966697
C -2.353380 0.205800 2.787044
O 2.397630 1.661992 1.145773
O 4.769900 1.092004 2.151367
O -0.240601 2.880638 -2.653405
O 0.781424 3.610650 -0.086291
H 5.576624 -1.405454 1.586570
H 3.984829 -1.443836 2.351327
H 4.615785 -1.925448 -0.607827
H 4.017933 -3.113743 0.536586
H 4.850444 0.499474 0.173285
H 0.142948 0.414228 0.536289
H 2.237756 -3.579603 -0.802033
H -0.213568 -3.796096 -2.447867
H -0.282726 -4.496431 -0.826820
H -3.230783 -2.408235 -2.444056
H -1.944248 -3.446011 0.887239
H -4.216883 -4.317224 0.885955
H -4.351205 -4.009792 -0.933218
H 0.697239 0.464885 -2.426846
H 1.655226 1.524450 -1.420377
H -1.376157 1.546116 -1.630361

H -0.597196 2.246574 0.629430
H -1.487039 4.497969 0.714054
H -1.389378 4.661149 -1.026901
H -3.702259 4.205801 -0.205766
H -3.152265 3.048065 -1.394940
H -4.376589 1.926421 0.403690
H -2.736898 1.357414 0.304919
H -2.152303 2.909222 2.256474
H -3.847140 3.334483 2.353862
H -3.394665 1.635835 4.011555
H -4.395641 0.905265 2.773258
H -1.344843 0.592837 2.958757
H -2.519925 -0.611112 3.492522
H -2.379133 -0.217873 1.781918
H 4.162521 1.850321 2.171311
H 0.492895 3.475343 -2.441101
H 1.410781 2.993646 0.326116

2d-c8, ΔG = 2.7899 kcal/mol, population = 0.18 %
C 5.110998 -1.128934 -0.608203
C 3.938600 -1.176841 -1.584685
C 2.617321 -1.059267 -0.886738
C 2.448783 -0.490570 0.337760
C 3.609535 -0.005666 1.051931
C 4.943171 0.063820 0.315238
C 1.083600 -0.306385 0.984758
N 1.534156 -1.570909 -1.579943
C 0.316372 -1.664409 -0.987336
C 0.082029 -1.141741 0.243868
C -0.859466 -2.355386 -1.602422
C -1.897128 -2.373508 -0.457627
C -1.261096 -1.458342 0.635990
O -1.881134 -1.097433 1.629111
O -3.116449 -1.794086 -0.902124
C -2.071256 -3.759087 0.107127
C -3.237148 -4.366228 0.281880
C 0.629554 1.174411 1.052042
C 0.563393 1.892905 -0.288846
C -0.273205 3.179165 -0.245588
C -1.766484 2.985402 0.000315
C -2.465873 2.027805 -0.969630
C -3.978260 2.264536 -1.066272
C -4.748661 2.165324 0.254583
C -4.727747 0.777033 0.891066

C -5.518070 0.704981 2.195868
O 3.564808 0.384758 2.220356
O 6.008126 0.158806 1.242450
O 1.878758 2.226685 -0.787956
O -0.122600 3.872736 -1.491610
H 6.052540 -1.052195 -1.152420
H 5.140886 -2.042439 -0.009468
H 4.015419 -0.349860 -2.300434
H 3.964258 -2.099140 -2.167921
H 4.898872 0.979306 -0.296479
H 1.144761 -0.648749 2.021198
H 1.691333 -2.005042 -2.477714
H -1.251279 -1.779386 -2.442607
H -0.604190 -3.351645 -1.965927
H -3.596420 -1.490426 -0.118104
H -1.146215 -4.250892 0.395095
H -3.291306 -5.360895 0.705427
H -4.169067 -3.895109 -0.004121
H 1.293870 1.725329 1.722165
H -0.357918 1.182717 1.514580
H 0.149840 1.235222 -1.054654
H 0.130092 3.808992 0.561075
H -1.918370 2.665840 1.033218
H -2.221994 3.977264 -0.077947
H -2.036349 2.156134 -1.965854
H -2.269606 0.992533 -0.681957
H -4.142803 3.259598 -1.493031
H -4.402552 1.549957 -1.779553
H -4.352213 2.893383 0.969852
H -5.789117 2.456408 0.075448
H -5.143260 0.057853 0.174915
H -3.696720 0.473515 1.084220
H -5.485936 -0.298173 2.626557
H -5.110274 1.398489 2.936089
H -6.567252 0.968467 2.037655
H 5.596736 0.447868 2.075029
H 2.360785 2.684248 -0.086252
H 0.799651 3.738455 -1.754966

2d-c440, ΔG = 2.8050 kcal/mol, population = 0.17 %
C 5.300158 -1.241135 0.668275
C 4.568546 -2.033193 -0.413309
C 3.106907 -1.701128 -0.453763
C 2.583250 -0.519429 -0.015508

C 3.461127 0.462450 0.569764
C 4.967350 0.232203 0.524581
C 1.105442 -0.172207 -0.130962
N 2.281507 -2.680803 -0.958827
C 0.927639 -2.560549 -0.879670
C 0.333957 -1.424764 -0.433043
C -0.018642 -3.660777 -1.243392
C -1.375774 -3.134016 -0.728507
C -1.087927 -1.638857 -0.387702
O -1.985977 -0.843590 -0.145715
O -2.353291 -3.202541 -1.755971
C -1.791437 -3.843408 0.535307
C -2.977187 -4.401268 0.738056
C 0.901392 0.923491 -1.210551
C -0.297232 1.858456 -1.018700
C -0.266487 2.612072 0.319127
C -1.391931 3.631108 0.470152
C -2.812669 3.075957 0.345615
C -3.176638 2.034403 1.406434
C -4.610675 1.509380 1.294367
C -5.698497 2.545900 1.580846
C -7.105615 1.952540 1.533103
O 3.053270 1.510858 1.089300
O 5.613120 1.004046 1.519352
O -0.335128 2.783724 -2.117086
O 0.967834 3.336062 0.449936
H 6.376290 -1.391820 0.582828
H 4.988818 -1.581387 1.658676
H 4.996819 -1.808771 -1.396737
H 4.695366 -3.105238 -0.253546
H 5.286714 0.570495 -0.474855
H 0.782737 0.222074 0.834022
H 2.688135 -3.555365 -1.256865
H -0.063553 -3.792178 -2.326048
H 0.270182 -4.613598 -0.798029
H -3.054408 -2.577063 -1.521736
H -1.036121 -3.859271 1.316094
H -3.212844 -4.885695 1.676918
H -3.742278 -4.402551 -0.028055
H 0.809925 0.443560 -2.186494
H 1.799209 1.543448 -1.254559
H -1.222211 1.287676 -1.074959
H -0.342677 1.879151 1.126452
H -1.271252 4.102495 1.449895

H -1.241608 4.413368 -0.277298
H -3.504092 3.919873 0.408121
H -2.957657 2.640982 -0.648296
H -2.495622 1.185394 1.324845
H -3.025789 2.468251 2.402364
H -4.763352 1.094540 0.291153
H -4.735341 0.672595 1.990126
H -5.519034 2.987101 2.567494
H -5.630559 3.366652 0.861646
H -7.218761 1.151502 2.268578
H -7.867078 2.707362 1.741487
H -7.317452 1.528216 0.547965
H 4.996635 1.724119 1.732680
H 0.387916 3.410070 -1.974306
H 1.670398 2.703627 0.684254

2d-c21, ΔG = 2.9637 kcal/mol, population = 0.13 %
C 5.139008 -1.125829 -0.618142
C 3.963521 -1.205383 -1.588748
C 2.641634 -1.108685 -0.887620
C 2.468137 -0.546948 0.339588
C 3.622577 -0.035205 1.047650
C 4.949652 0.065646 0.303356
C 1.103567 -0.389892 0.995679
N 1.565843 -1.636601 -1.579816
C 0.342024 -1.722438 -0.998068
C 0.099120 -1.205275 0.233896
C -0.837502 -2.386829 -1.636197
C -1.874365 -2.428506 -0.492101
C -1.262469 -1.489483 0.593162
O -1.911877 -1.091698 1.552541
O -3.116178 -1.899840 -0.932454
C -1.993082 -3.815731 0.086593
C -3.133491 -4.467095 0.270490
C 0.656866 1.089199 1.078841
C 0.691300 1.830299 -0.255628
C -0.414468 2.888635 -0.378283
C -1.781928 2.260029 -0.598567
C -2.927789 3.270170 -0.561718
C -4.288741 2.664346 -0.923659
C -4.704458 1.452332 -0.083350
C -4.788352 1.716671 1.419697
C -5.252587 0.490796 2.204373
O 3.575275 0.348827 2.217443

O 6.017565 0.185373 1.224642
O 1.977948 2.441533 -0.491790
O -0.131283 3.749436 -1.486929
H 6.075633 -1.029612 -1.167673
H 5.193522 -2.036410 -0.016669
H 4.021305 -0.385421 -2.313951
H 4.004631 -2.133432 -2.162075
H 4.880388 0.979450 -0.307800
H 1.165081 -0.755236 2.024263
H 1.725323 -2.056771 -2.483686
H -1.223685 -1.778930 -2.456462
H -0.590728 -3.372909 -2.031599
H -3.608467 -1.637074 -0.141079
H -1.049010 -4.268522 0.376217
H -3.145896 -5.459415 0.702739
H -4.085306 -4.037520 -0.015400
H 1.289135 1.617851 1.795307
H -0.351725 1.101717 1.492072
H 0.572909 1.133920 -1.088621
H -0.426898 3.484647 0.546946
H -1.765362 1.745707 -1.565458
H -1.946784 1.494212 0.160497
H -2.972218 3.718316 0.435742
H -2.709923 4.085791 -1.254755
H -5.054368 3.442120 -0.833907
H -4.273414 2.368798 -1.978064
H -5.679744 1.100346 -0.435585
H -4.006145 0.629830 -0.262750
H -3.810681 2.031761 1.795203
H -5.470746 2.554414 1.600519
H -6.236514 0.156705 1.864358
H -4.552133 -0.337687 2.074346
H -5.322590 0.702294 3.273739
H 5.604534 0.463583 2.060047
H 2.262319 2.879461 0.320886
H 0.833286 3.839588 -1.504339

2d-c418, ΔG = 3.0089 kcal/mol, population = 0.12 %
C 4.179242 0.162586 -1.016980
C 3.386898 -0.920800 -1.746584
C 2.100419 -1.247510 -1.048118
C 1.459293 -0.403930 -0.196492
C 2.070527 0.871240 0.124890
C 3.264313 1.333899 -0.702052

C 0.110249 -0.713401 0.436287
N 1.575549 -2.500324 -1.314345
C 0.493671 -2.956635 -0.639930
C -0.194644 -2.170898 0.235820
C -0.034479 -4.350058 -0.769739
C -1.074374 -4.434164 0.369641
C -1.234013 -2.956771 0.823555
O -2.157676 -2.605893 1.561522
O -2.312282 -4.912619 -0.132962
C -0.549888 -5.243526 1.529700
C -1.191516 -6.254326 2.099286
C -0.980167 0.227975 -0.151321
C -2.019849 0.732900 0.854079
C -2.545473 2.115956 0.450906
C -1.522386 3.209119 0.729534
C -1.730875 4.482454 -0.086656
C -0.678352 5.562144 0.187755
C 0.776292 5.119276 -0.033251
C 1.078521 4.528010 -1.414254
C 0.817293 5.488763 -2.573770
O 1.652197 1.606156 1.019666
O 3.952267 2.368844 -0.026835
O -3.159566 -0.128203 0.939570
O -3.749080 2.418223 1.166921
H 5.015924 0.495424 -1.631339
H 4.583617 -0.232866 -0.082206
H 3.145528 -0.588089 -2.762579
H 3.986302 -1.827133 -1.849467
H 2.846377 1.721088 -1.644693
H 0.182823 -0.499668 1.506261
H 2.079209 -3.118979 -1.933116
H -0.535824 -4.486042 -1.729625
H 0.755223 -5.097827 -0.689551
H -2.997192 -4.653513 0.500401
H 0.421685 -4.928707 1.899806
H -0.760151 -6.788112 2.936299
H -2.157934 -6.587080 1.741937
H -1.501537 -0.274591 -0.968983
H -0.475046 1.086247 -0.593493
H -1.559058 0.817929 1.846777
H -2.771681 2.085718 -0.625177
H -1.559868 3.434032 1.800237
H -0.524352 2.816156 0.538013
H -1.720214 4.223117 -1.150246

H -2.724333 4.891784 0.116740
H -0.896241 6.431133 -0.439698
H -0.779882 5.904985 1.222829
H 1.428595 5.983702 0.130847
H 1.052286 4.382907 0.725806
H 2.127188 4.217783 -1.432310
H 0.493528 3.615205 -1.560856
H 1.103494 5.041452 -3.528224
H -0.237779 5.761880 -2.641572
H 1.390304 6.412204 -2.452476
H 3.326493 2.701014 0.639634
H -2.860817 -0.999628 1.268170
H -4.220416 1.575104 1.241165

2d-c5, ΔG = 3.0221 kcal/mol, population = 0.12 %
C 5.210507 -1.296402 -0.487745
C 4.067070 -1.341937 -1.498456
C 2.732681 -1.143454 -0.845392
C 2.552417 -0.488537 0.335208
C 3.710850 -0.018470 1.062244
C 5.071507 -0.052616 0.371302
C 1.177421 -0.231992 0.935729
N 1.653863 -1.676952 -1.522097
C 0.421658 -1.704272 -0.951588
C 0.173286 -1.100073 0.238227
C -0.757088 -2.403256 -1.552142
C -1.842531 -2.276391 -0.460683
C -1.191067 -1.342569 0.609449
O -1.820395 -0.900949 1.563255
O -2.985446 -1.623797 -1.003152
C -2.163451 -3.605466 0.168128
C -3.383333 -4.107916 0.303466
C 0.761628 1.263279 0.892613
C 0.387341 1.801182 -0.479707
C -0.138098 3.242053 -0.435600
C -1.441868 3.431237 0.334355
C -2.576030 2.486839 -0.066453
C -3.930325 2.917334 0.500470
C -5.057063 1.921598 0.204913
C -5.014747 0.667213 1.079705
C -6.023319 -0.397374 0.650244
O 3.648441 0.432843 2.208290
O 6.106559 0.033425 1.332533
O 1.478874 1.740618 -1.422480

O -0.349174 3.697455 -1.779517
H 6.170833 -1.290877 -1.003833
H 5.177998 -2.178560 0.156131
H 4.197278 -0.550927 -2.246326
H 4.073528 -2.289267 -2.040209
H 5.098487 0.833133 -0.284022
H 1.217280 -0.497589 1.995070
H 1.815896 -2.166902 -2.389774
H -1.088014 -1.897458 -2.461018
H -0.528608 -3.438876 -1.808197
H -3.518126 -1.309548 -0.258850
H -1.300856 -4.149022 0.543283
H -3.542764 -5.066084 0.781027
H -4.257010 -3.586796 -0.067375
H 1.565647 1.866606 1.321988
H -0.101879 1.378903 1.548544
H -0.378860 1.171826 -0.932019
H 0.636909 3.867000 0.031539
H -1.240355 3.336183 1.404431
H -1.754791 4.467627 0.175457
H -2.646826 2.445692 -1.156817
H -2.345710 1.474060 0.270558
H -3.847482 3.058923 1.584466
H -4.187386 3.896095 0.083334
H -6.026805 2.411182 0.340186
H -5.006867 1.629713 -0.850735
H -4.006030 0.245837 1.075574
H -5.205987 0.953340 2.119201
H -7.045083 -0.011662 0.693283
H -5.843297 -0.720366 -0.379464
H -5.972566 -1.280139 1.291462
H 5.680898 0.381753 2.134882
H 2.269461 2.104021 -1.003224
H 0.387806 3.338502 -2.294943

2d-c126, ΔG = 3.0484 kcal/mol, population = 0.11 %
C 5.055260 -0.909444 -1.352991
C 3.708555 -1.057123 -2.056265
C 2.561763 -1.072198 -1.089604
C 2.614035 -0.548003 0.164972
C 3.856997 0.028283 0.632604
C 4.983940 0.246073 -0.371183
C 1.414149 -0.505069 1.101188
N 1.402613 -1.663739 -1.559242

C 0.325588 -1.821993 -0.748223
C 0.310055 -1.343338 0.522603
C -0.929030 -2.522535 -1.165772
C -1.746262 -2.591530 0.142440
C -0.950020 -1.677231 1.125493
O -1.412305 -1.324753 2.203750
O -3.040932 -2.041986 -0.060144
C -1.778734 -3.991549 0.699012
C -2.876807 -4.636090 1.069521
C 0.923972 0.940383 1.359863
C 0.598435 1.720280 0.089191
C -0.578033 2.691301 0.257330
C -1.908476 1.964892 0.385148
C -3.095356 2.901526 0.605709
C -4.413981 2.163724 0.859616
C -4.857962 1.220887 -0.266920
C -4.996594 1.875669 -1.645639
C -6.019160 3.010712 -1.694040
O 4.037728 0.374801 1.801148
O 6.212339 0.428298 0.308064
O 1.750042 2.435920 -0.409159
O -0.643225 3.552535 -0.885510
H 5.845026 -0.733167 -2.083526
H 5.298342 -1.824833 -0.808130
H 3.554684 -0.220986 -2.748343
H 3.689122 -1.967466 -2.658165
H 4.722237 1.164043 -0.920692
H 1.711214 -0.911468 2.071953
H 1.395382 -2.055181 -2.489631
H -1.472201 -1.933557 -1.907051
H -0.724162 -3.502743 -1.598207
H -3.371817 -1.767650 0.807607
H -0.804314 -4.460653 0.802840
H -2.826285 -5.638903 1.473866
H -3.857518 -4.188440 0.969230
H 1.682504 1.481242 1.928798
H 0.045222 0.875488 2.000270
H 0.341013 1.039436 -0.725084
H -0.402614 3.300149 1.157531
H -2.054962 1.370918 -0.521174
H -1.854768 1.258590 1.216542
H -2.882861 3.544454 1.466233
H -3.196806 3.566709 -0.254156
H -4.320850 1.579715 1.781463

H -5.197928 2.902626 1.048313
H -4.154531 0.388009 -0.343032
H -5.820720 0.778986 0.012196
H -4.022754 2.249383 -1.976009
H -5.282139 1.103915 -2.366902
H -7.000872 2.666119 -1.357248
H -5.725587 3.845637 -1.054465
H -6.131042 3.397698 -2.709197
H 5.967380 0.645941 1.223908
H 2.173959 2.886717 0.332547
H 0.278004 3.726561 -1.129752

2d-c127, ΔG = 3.0767 kcal/mol, population = 0.11 %
C 4.372203 -0.731722 -1.989283
C 2.905408 -0.742259 -2.411286
C 1.977162 -0.844605 -1.237875
C 2.313186 -0.501880 0.035012
C 3.656756 -0.034381 0.303797
C 4.566903 0.282860 -0.877137
C 1.324964 -0.530017 1.192881
N 0.708718 -1.316497 -1.529522
C -0.183028 -1.565134 -0.536754
C 0.090522 -1.268098 0.759963
C -1.528482 -2.178567 -0.765252
C -2.040652 -2.440833 0.668285
C -1.022360 -1.676594 1.570802
O -1.228975 -1.482789 2.762563
O -3.333281 -1.879188 0.842248
C -1.982650 -3.907739 1.009057
C -2.984935 -4.608156 1.522342
C 0.962539 0.891386 1.690972
C 0.476563 1.822324 0.580396
C -0.569568 2.829394 1.065429
C -1.138319 3.700560 -0.052979
C -1.728151 2.938431 -1.243635
C -2.864775 1.979252 -0.888810
C -3.456497 1.290601 -2.119219
C -4.607382 0.326620 -1.813553
C -5.863766 1.011265 -1.274045
O 4.089269 0.147229 1.443024
O 5.916225 0.337504 -0.452416
O 1.570880 2.484934 -0.077466
O -0.041935 3.649579 2.122509
H 5.007735 -0.483144 -2.839485

H 4.666755 -1.719245 -1.625952
H 2.664396 0.181077 -2.950955
H 2.712054 -1.566081 -3.101061
H 4.249235 1.273141 -1.239210
H 1.785365 -1.051922 2.036030
H 0.488761 -1.574104 -2.480207
H -2.191889 -1.474592 -1.269539
H -1.469207 -3.081425 -1.374342
H -3.452302 -1.733067 1.792109
H -1.022356 -4.379123 0.819214
H -2.870568 -5.659172 1.754722
H -3.950856 -4.157952 1.713789
H 1.827022 1.334603 2.187110
H 0.187310 0.785012 2.450762
H 0.005842 1.226519 -0.202377
H -1.373196 2.256706 1.531922
H -1.905494 4.344063 0.387113
H -0.339854 4.356454 -0.413909
H -2.101194 3.672335 -1.964527
H -0.935649 2.389853 -1.761437
H -2.511816 1.209420 -0.195476
H -3.642777 2.535308 -0.357241
H -3.802648 2.052059 -2.827804
H -2.656364 0.742532 -2.629119
H -4.863024 -0.214883 -2.729527
H -4.269136 -0.423818 -1.093123
H -5.680894 1.491897 -0.310967
H -6.218803 1.780134 -1.966120
H -6.672587 0.291117 -1.132043
H 5.874909 0.435425 0.514454
H 2.145362 2.857488 0.602469
H 0.492060 4.345203 1.719177

2d-c17, ΔG = 3.0842 kcal/mol, population = 0.11 %
C 4.288406 -0.845246 -2.040902
C 2.809196 -0.908979 -2.411914
C 1.920330 -0.932107 -1.204247
C 2.289825 -0.481471 0.024951
C 3.632986 0.025635 0.210497
C 4.500794 0.258137 -1.020546
C 1.343467 -0.440201 1.217077
N 0.653730 -1.448975 -1.416371
C -0.203543 -1.626158 -0.379773
C 0.100841 -1.211931 0.877283

C -1.536382 -2.294504 -0.507949
C -1.990389 -2.454516 0.959602
C -0.973374 -1.580468 1.757466
O -1.147873 -1.287881 2.933922
O -3.300277 -1.935442 1.131828
C -1.856498 -3.884426 1.418563
C -2.810779 -4.579873 2.022030
C 0.999255 1.001914 1.670901
C 0.583117 1.945338 0.548891
C -0.270074 3.121617 1.041930
C -1.646431 2.736266 1.584358
C -2.486391 1.756095 0.757970
C -2.893267 2.236740 -0.634332
C -3.799573 1.245129 -1.360756
C -4.215273 1.710492 -2.755174
C -5.113530 0.707644 -3.477036
O 4.095762 0.307662 1.316934
O 5.861607 0.365687 -0.645472
O 1.729866 2.473916 -0.155207
O -0.436660 4.065653 -0.024022
H 4.892237 -0.656543 -2.928765
H 4.610775 -1.796269 -1.610135
H 2.536245 -0.033735 -3.013052
H 2.606960 -1.786035 -3.029539
H 4.154590 1.211026 -1.450063
H 1.837014 -0.915881 2.069437
H 0.413510 -1.793467 -2.334162
H -2.241495 -1.654864 -1.041023
H -1.469829 -3.242036 -1.043879
H -3.395863 -1.713586 2.069488
H -0.882891 -4.330579 1.235768
H -2.643158 -5.602170 2.335925
H -3.788929 -4.155233 2.209562
H 1.858520 1.431978 2.189947
H 0.199243 0.924019 2.406528
H 0.028635 1.408075 -0.221953
H 0.286706 3.610223 1.855726
H -1.507719 2.317575 2.583925
H -2.202623 3.667881 1.724104
H -1.962919 0.801359 0.668318
H -3.392311 1.534841 1.331285
H -3.404833 3.201631 -0.547264
H -2.001942 2.424855 -1.236332
H -3.285801 0.281225 -1.445991

H -4.695897 1.057716 -0.758299
H -4.731250 2.673079 -2.673646
H -3.316862 1.895019 -3.353740
H -5.394614 1.062709 -4.470959
H -4.608480 -0.254683 -3.596694
H -6.033347 0.529722 -2.913587
H 5.849218 0.549304 0.309581
H 2.359416 2.803982 0.499473
H 0.409087 4.077277 -0.495836

2d-c412, ΔG = 3.2718 kcal/mol, population = 0.08 %
C 4.005377 -1.112573 -2.504755
C 2.512154 -0.979289 -2.797595
C 1.711675 -0.798022 -1.542942
C 2.214225 -0.298031 -0.381203
C 3.627382 0.016930 -0.299223
C 4.445950 0.014245 -1.586917
C 1.329660 -0.002343 0.823648
N 0.387002 -1.204409 -1.614180
C -0.344385 -1.342011 -0.481677
C 0.099019 -0.851616 0.708204
C -1.637666 -2.088778 -0.396800
C -1.863472 -2.223592 1.128199
C -0.771865 -1.304155 1.744937
O -0.746001 -1.027750 2.945815
O -3.147285 -1.717535 1.466889
C -1.636699 -3.639405 1.592480
C -2.500409 -4.351328 2.302906
C 1.088596 1.531013 0.863888
C 0.076348 2.099682 1.865838
C -1.408195 1.997678 1.475177
C -1.743702 2.643779 0.143204
C -3.196780 2.432163 -0.279520
C -3.514265 2.983673 -1.674965
C -2.611212 2.463804 -2.800708
C -2.561925 0.940281 -2.920164
C -1.667106 0.462526 -4.061265
O 4.190746 0.305518 0.756252
O 5.825891 -0.072900 -1.287334
O 0.259300 1.581074 3.192611
O -2.184947 2.633467 2.503001
H 4.575776 -1.083014 -3.433235
H 4.207026 -2.068408 -2.015472
H 2.331364 -0.112939 -3.444034

H 2.151201 -1.854706 -3.339767
H 4.232062 0.976028 -2.080017
H 1.867301 -0.277175 1.732979
H 0.054490 -1.612958 -2.475681
H -2.453354 -1.510986 -0.834139
H -1.590454 -3.048955 -0.911184
H -3.156904 -1.565318 2.422652
H -0.675244 -4.059470 1.311083
H -2.269031 -5.362555 2.611615
H -3.466240 -3.953673 2.588263
H 0.816274 1.864543 -0.137446
H 2.056539 1.984182 1.083104
H 0.289740 3.168190 1.957870
H -1.700698 0.945796 1.424372
H -1.509825 3.712795 0.188510
H -1.092971 2.209561 -0.615184
H -3.416486 1.360395 -0.249427
H -3.862696 2.898908 0.449260
H -4.555447 2.744130 -1.914423
H -3.448721 4.076475 -1.651374
H -2.959506 2.883384 -3.750086
H -1.592721 2.841760 -2.664815
H -2.199474 0.514466 -1.981657
H -3.577437 0.554194 -3.057453
H -2.004007 0.862845 -5.020728
H -0.634462 0.788106 -3.910343
H -1.667826 -0.627195 -4.140184
H 5.903659 0.183249 -0.352504
H 0.014509 0.633332 3.194271
H -1.774098 2.371345 3.339422

2d-c78, ΔG = 3.3766 kcal/mol, population = 0.07 %
C 4.992983 -0.885957 -1.328534
C 3.650476 -1.061536 -2.033303
C 2.499743 -1.055604 -1.071549
C 2.541233 -0.491638 0.165605
C 3.778189 0.105704 0.621202
C 4.909873 0.298036 -0.382476
C 1.337619 -0.431529 1.096452
N 1.347480 -1.671164 -1.527863
C 0.273688 -1.824205 -0.712711
C 0.246900 -1.302642 0.541346
C -0.963351 -2.570779 -1.102366
C -1.753877 -2.651444 0.221829

C -1.000127 -1.657590 1.160506
O -1.476149 -1.278933 2.223638
O -3.087685 -2.207494 0.030011
C -1.672904 -4.031685 0.823802
C -2.713586 -4.732127 1.253717
C 0.829017 1.011597 1.338169
C 0.488276 1.788803 0.073280
C -0.516626 2.922855 0.313530
C -1.908009 2.473905 0.750224
C -2.593644 1.473773 -0.180351
C -4.013297 1.106230 0.262598
C -5.005422 2.274773 0.329646
C -5.150794 3.078210 -0.967544
C -5.622789 2.248827 -2.161615
O 3.950374 0.491641 1.778855
O 6.133319 0.507971 0.297763
O 1.667887 2.344349 -0.550849
O -0.656248 3.688405 -0.891159
H 5.785507 -0.727192 -2.060134
H 5.238207 -1.783300 -0.755342
H 3.495062 -0.246095 -2.749392
H 3.639192 -1.988670 -2.609245
H 4.646522 1.197162 -0.961694
H 1.638680 -0.812870 2.076505
H 1.349452 -2.095228 -2.443831
H -1.539483 -2.005541 -1.837210
H -0.734439 -3.547631 -1.530165
H -3.404810 -1.877658 0.883348
H -0.666763 -4.432755 0.907962
H -2.584512 -5.715405 1.687622
H -3.723791 -4.350790 1.174126
H 1.577686 1.566386 1.907525
H -0.053101 0.933727 1.972844
H 0.080405 1.123288 -0.688329
H -0.101777 3.571290 1.099580
H -1.848302 2.055694 1.758213
H -2.514667 3.378210 0.835520
H -2.608521 1.876650 -1.195014
H -2.009872 0.552655 -0.228301
H -4.398624 0.340442 -0.414430
H -3.963821 0.636599 1.250900
H -5.986318 1.876271 0.610456
H -4.717331 2.955809 1.135282
H -5.860649 3.892117 -0.792398

H -4.198512 3.557590 -1.212957
H -4.898147 1.476576 -2.427320
H -6.571330 1.750887 -1.942020
H -5.771756 2.877248 -3.042332
H 5.881621 0.752909 1.204873
H 2.169965 2.821307 0.123153
H 0.231676 3.740354 -1.273624

2d-c40, ΔG = 3.3948 kcal/mol, population = 0.06 %
C 5.077884 -0.826454 -1.230643
C 3.759866 -0.996589 -1.981961
C 2.580831 -1.024923 -1.056152
C 2.577692 -0.483927 0.191877
C 3.791977 0.118086 0.698650
C 4.956304 0.335755 -0.261483
C 1.344593 -0.455037 1.083552
N 1.450714 -1.648044 -1.556388
C 0.365584 -1.851445 -0.767525
C 0.298113 -1.358181 0.496476
C -0.839437 -2.628324 -1.196213
C -1.643188 -2.774132 0.114940
C -0.944783 -1.772274 1.086259
O -1.453336 -1.433574 2.147916
O -2.992768 -2.382713 -0.082190
C -1.510152 -4.164282 0.684079
C -2.525197 -4.919045 1.082104
C 0.784479 0.973052 1.300283
C 0.453833 1.729497 0.020116
C -0.572106 2.850813 0.227641
C -1.960956 2.392025 0.660460
C -2.615799 1.348826 -0.244775
C -4.036807 0.973439 0.188294
C -5.054428 2.119258 0.183675
C -5.225973 2.803952 -1.172571
C -6.323765 3.866229 -1.162797
O 3.919847 0.488340 1.867229
O 6.155143 0.535082 0.464520
O 1.634417 2.297538 -0.590284
O -0.711337 3.588926 -0.994168
H 5.892231 -0.646152 -1.932708
H 5.310957 -1.734614 -0.669600
H 3.618350 -0.165256 -2.682498
H 3.776169 -1.909438 -2.580313
H 4.710251 1.246726 -0.829819

H 1.625151 -0.823131 2.074274
H 1.485203 -2.056342 -2.478819
H -1.425492 -2.061129 -1.921780
H -0.572793 -3.583987 -1.649240
H -3.330933 -2.085389 0.775173
H -0.488800 -4.524195 0.771339
H -2.359606 -5.906781 1.492844
H -3.549509 -4.578506 0.998422
H 1.498078 1.555815 1.886820
H -0.112574 0.870581 1.910330
H 0.071242 1.048051 -0.740258
H -0.173774 3.521309 1.003652
H -1.907819 2.009714 1.683058
H -2.582985 3.288973 0.707286
H -2.619999 1.713688 -1.273655
H -2.017157 0.436203 -0.248867
H -4.397387 0.177427 -0.470149
H -3.995346 0.541828 1.194207
H -6.023582 1.721674 0.503381
H -4.779743 2.870443 0.931132
H -4.281894 3.265439 -1.475283
H -5.453258 2.046261 -1.930609
H -7.291285 3.429021 -0.902131
H -6.105322 4.646764 -0.428981
H -6.425922 4.346039 -2.138655
H 5.871952 0.765468 1.366055
H 2.107854 2.804269 0.082824
H 0.180099 3.650021 -1.366945

2d-c113, ΔG = 3.4187 kcal/mol, population = 0.06 %
C 3.491135 -0.452367 -2.731370
C 1.970437 -0.558292 -2.786694
C 1.362081 -0.752665 -1.429269
C 1.950168 -0.377056 -0.260185
C 3.308923 0.134783 -0.310959
C 3.883438 0.552299 -1.661999
C 1.297397 -0.560745 1.110232
N 0.115921 -1.352391 -1.432654
C -0.548964 -1.589379 -0.275823
C -0.036999 -1.237826 0.934212
C -1.914484 -2.196853 -0.225800
C -2.172351 -2.355565 1.287466
C -1.022040 -1.532324 1.934741
O -1.073647 -1.169333 3.109322

O -3.416730 -1.770153 1.636530
C -2.048547 -3.793564 1.724581
C -2.950306 -4.443918 2.447109
C 1.241518 0.754709 1.928190
C 0.455834 1.924987 1.332937
C -1.069150 1.789300 1.454661
C -1.841743 2.944024 0.828210
C -1.561731 3.175987 -0.659186
C -1.772534 1.943844 -1.540469
C -1.701895 2.263604 -3.033435
C -1.733669 1.033897 -3.945554
C -2.992602 0.179130 -3.803765
O 4.023422 0.245661 0.685112
O 5.287360 0.691395 -1.570105
O 0.874010 3.146732 1.961913
O -1.436213 1.739409 2.845946
H 3.884204 -0.142614 -3.699743
H 3.928223 -1.422708 -2.484014
H 1.549291 0.358815 -3.214555
H 1.666162 -1.376672 -3.441878
H 3.428366 1.525489 -1.903321
H 1.941247 -1.224394 1.697861
H -0.289323 -1.636590 -2.312132
H -2.653078 -1.514115 -0.649711
H -1.964949 -3.135954 -0.777860
H -3.368830 -1.545127 2.577597
H -1.131672 -4.286335 1.414013
H -2.795618 -5.475733 2.735255
H -3.872418 -3.971419 2.761021
H 2.266054 1.095238 2.057542
H 0.861554 0.521888 2.923532
H 0.720055 2.034419 0.279679
H -1.374888 0.862697 0.970527
H -2.905374 2.730869 0.967141
H -1.622885 3.855032 1.389301
H -2.221171 3.977236 -1.006471
H -0.541566 3.547503 -0.795281
H -1.016679 1.188756 -1.308881
H -2.739486 1.492470 -1.298809
H -2.531092 2.930326 -3.296149
H -0.782390 2.824823 -3.231004
H -1.637671 1.363287 -4.984206
H -0.852034 0.417242 -3.744069
H -2.991718 -0.643934 -4.521338

H -3.074865 -0.255010 -2.805728
H -3.891913 0.775682 -3.979267
H 5.480913 0.709443 -0.616519
H 0.451325 3.156635 2.832437
H -1.288838 0.833609 3.160587

2d-c242, ΔG = 3.4739 kcal/mol, population = 0.06 %
C 3.188137 -0.223548 -2.905409
C 1.669969 -0.302643 -2.771244
C 1.239716 -0.614706 -1.368324
C 1.998046 -0.369269 -0.264010
C 3.334909 0.163816 -0.439330
C 3.741525 0.678993 -1.816907
C 1.523085 -0.653762 1.157113
N -0.018021 -1.178589 -1.249509
C -0.487704 -1.583548 -0.039293
C 0.220636 -1.396593 1.099854
C -1.814772 -2.247349 0.157994
C -1.941976 -2.332044 1.691582
C -0.519109 -1.939605 2.209885
O -0.187059 -2.021967 3.383507
O -2.813162 -1.262636 2.108470
C -2.381795 -3.677421 2.177150
C -3.496388 -3.929775 2.850189
C 1.422697 0.620839 2.037969
C 0.775961 1.863531 1.422006
C -0.677697 1.697212 0.963309
C -1.193093 2.939440 0.244029
C -2.625259 2.811914 -0.281293
C -2.870943 1.661583 -1.263923
C -1.973357 1.688918 -2.501913
C -2.317504 0.626279 -3.550865
C -3.645154 0.866776 -4.269660
O 4.152905 0.233778 0.478229
O 5.149306 0.789113 -1.898635
O 0.866649 2.941083 2.368334
O -1.494537 1.418166 2.113373
H 3.460192 0.163088 -3.887633
H 3.627949 -1.218113 -2.800496
H 1.220904 0.655159 -3.058205
H 1.262824 -1.052357 -3.451950
H 3.284240 1.676144 -1.916661
H 2.270521 -1.285518 1.646442
H -0.543517 -1.383822 -2.086456

H -2.635101 -1.663996 -0.262489
H -1.834238 -3.233319 -0.308582
H -2.880972 -1.301970 3.073530
H -1.694298 -4.483237 1.937784
H -3.737394 -4.934909 3.170625
H -4.210357 -3.152908 3.094189
H 2.429787 0.908877 2.336610
H 0.880182 0.356762 2.947342
H 1.360755 2.189541 0.559589
H -0.722038 0.855010 0.276568
H -1.134937 3.787384 0.929512
H -0.508579 3.159606 -0.580092
H -3.304353 2.700663 0.567626
H -2.894332 3.753751 -0.770056
H -2.745540 0.701562 -0.753320
H -3.919631 1.697547 -1.569355
H -2.028604 2.679973 -2.967005
H -0.932068 1.560147 -2.195300
H -1.513590 0.586986 -4.291684
H -2.344520 -0.360493 -3.074809
H -3.821178 0.107353 -5.034261
H -4.490070 0.840518 -3.579354
H -3.647032 1.843306 -4.761022
H 5.461727 0.770898 -0.977581
H 0.226320 2.741897 3.065362
H -1.915380 0.549679 2.017160

2d-c77, ΔG = 3.5046 kcal/mol, population = 0.05 %
C 5.209457 -1.157450 -0.815710
C 3.983676 -1.230118 -1.723047
C 2.705385 -1.098599 -0.951706
C 2.603737 -0.479253 0.256406
C 3.802396 0.021016 0.892352
C 5.095936 0.060197 0.083510
C 1.276566 -0.281745 0.974647
N 1.590370 -1.654534 -1.548575
C 0.413307 -1.740313 -0.876638
C 0.245527 -1.170882 0.344638
C -0.787345 -2.468379 -1.393614
C -1.764988 -2.432872 -0.198208
C -1.073875 -1.473760 0.821712
O -1.647710 -1.064522 1.823866
O -3.002393 -1.861063 -0.603970
C -1.919094 -3.793679 0.428754

C -3.077255 -4.394823 0.665201
C 0.809267 1.197975 0.999617
C 0.331890 1.756252 -0.332887
C -0.303790 3.146710 -0.213729
C -1.555277 3.209643 0.655286
C -2.624336 2.163839 0.337162
C -3.910632 2.388647 1.130517
C -4.919034 1.239976 1.016617
C -5.370495 0.903382 -0.409472
C -6.040732 2.068504 -1.136504
O 3.823849 0.443407 2.050755
O 6.208570 0.172171 0.950734
O 1.380383 1.816144 -1.323447
O -0.650728 3.608464 -1.527310
H 6.119143 -1.098265 -1.413668
H 5.271862 -2.054179 -0.194535
H 4.015589 -0.418694 -2.459787
H 3.979128 -2.164470 -2.287253
H 5.025868 0.959646 -0.549506
H 1.413067 -0.566053 2.020998
H 1.695152 -2.121149 -2.437575
H -1.225322 -1.940372 -2.242689
H -0.542576 -3.480988 -1.716780
H -3.450860 -1.553157 0.196793
H -0.984502 -4.273554 0.705334
H -3.114850 -5.371868 1.129549
H -4.019621 -3.937044 0.392064
H 1.615968 1.820098 1.395688
H -0.013868 1.261315 1.712026
H -0.403812 1.085197 -0.776137
H 0.450623 3.824623 0.211554
H -1.259382 3.125934 1.704147
H -1.975710 4.213078 0.538561
H -2.834594 2.179276 -0.734404
H -2.246022 1.165591 0.567912
H -3.656938 2.520196 2.187700
H -4.373563 3.328380 0.813153
H -4.478716 0.345511 1.470816
H -5.801249 1.483559 1.618266
H -4.515755 0.552709 -0.994177
H -6.067020 0.060747 -0.361446
H -5.349181 2.900359 -1.284708
H -6.402859 1.762333 -2.120265
H -6.895148 2.445866 -0.567844

H 5.842216 0.483583 1.796377
H 2.145523 2.259146 -0.934063
H 0.078681 3.334966 -2.102120

2d-c66, ΔG = 3.5272 kcal/mol, population = 0.05 %
C 4.918909 -0.881401 -1.278295
C 3.579087 -1.012863 -1.997533
C 2.421765 -1.032327 -1.044601
C 2.461971 -0.529705 0.218441
C 3.703531 0.028651 0.710313
C 4.840921 0.258344 -0.279103
C 1.246644 -0.481381 1.134441
N 1.261222 -1.601789 -1.539739
C 0.181612 -1.784164 -0.737869
C 0.155456 -1.328047 0.541653
C -1.063672 -2.490922 -1.173018
C -1.859679 -2.633342 0.142408
C -1.100636 -1.697343 1.134058
O -1.581058 -1.366330 2.211275
O -3.189373 -2.168204 -0.028495
C -1.791652 -4.044677 0.669443
C -2.839363 -4.763447 1.049005
C 0.746391 0.963352 1.384218
C 0.362238 1.715539 0.111530
C -0.697145 2.794521 0.365505
C -1.290571 3.378750 -0.912993
C -1.944065 2.351958 -1.849642
C -2.966131 1.420248 -1.188670
C -4.143817 2.138076 -0.531704
C -5.142320 1.179016 0.115803
C -6.335085 1.894056 0.747169
O 3.875231 0.355041 1.886114
O 6.063155 0.428647 0.413968
O 1.506433 2.270452 -0.557019
O -0.161760 3.835358 1.202680
H 5.716733 -0.695349 -1.997624
H 5.154316 -1.806209 -0.746104
H 3.437451 -0.166200 -2.679561
H 3.561258 -1.915071 -2.611754
H 4.584730 1.184739 -0.817214
H 1.530558 -0.884493 2.110406
H 1.261437 -1.976390 -2.477137
H -1.628366 -1.876424 -1.876552
H -0.848425 -3.446242 -1.653217

H -3.511858 -1.903756 0.845432
H -0.788149 -4.454773 0.740579
H -2.718428 -5.769882 1.428706
H -3.847141 -4.373900 0.980399
H 1.506834 1.524442 1.929107
H -0.124579 0.894091 2.037703
H -0.058217 1.008125 -0.603012
H -1.491390 2.339252 0.959284
H -2.021042 4.136460 -0.620105
H -0.497016 3.898880 -1.458654
H -2.432546 2.898296 -2.662024
H -1.166433 1.745472 -2.321734
H -3.349787 0.732401 -1.948355
H -2.473190 0.787069 -0.445351
H -3.781047 2.835121 0.230372
H -4.658978 2.748664 -1.282372
H -5.496022 0.465157 -0.635345
H -4.623992 0.588309 0.878323
H -7.028508 1.186676 1.207193
H -6.007442 2.592292 1.521898
H -6.889468 2.466610 -0.001146
H 5.809209 0.633017 1.330528
H 1.978075 2.819516 0.081879
H 0.291374 4.470431 0.634609

2d-c135, ΔG = 3.5636 kcal/mol, population = 0.05 %
C 3.405481 -0.426155 -2.749704
C 1.884021 -0.536849 -2.773392
C 1.305335 -0.746316 -1.404963
C 1.919335 -0.383012 -0.245963
C 3.274562 0.133783 -0.319022
C 3.820478 0.565498 -1.677093
C 1.292269 -0.572126 1.135233
N 0.061097 -1.349715 -1.385261
C -0.573861 -1.607575 -0.216239
C -0.038213 -1.261419 0.984861
C -1.932850 -2.229144 -0.140277
C -2.142076 -2.421113 1.376951
C -1.001469 -1.569235 2.002646
O -1.043572 -1.199284 3.175338
O -3.395576 -1.892418 1.775597
C -1.948611 -3.861609 1.782147
C -2.798117 -4.556779 2.525918
C 1.236397 0.745856 1.947019

C 0.460530 1.913384 1.334087
C -1.066832 1.780615 1.422861
C -1.816023 2.953323 0.798630
C -1.537156 3.188831 -0.687801
C -1.991814 2.056392 -1.610431
C -1.739183 2.366430 -3.087713
C -2.472779 1.445387 -4.066712
C -2.081332 -0.027797 -3.963916
O 4.007363 0.240299 0.664312
O 5.226230 0.703185 -1.612853
O 0.870106 3.137047 1.964795
O -1.465428 1.719976 2.805587
H 3.775742 -0.103323 -3.722779
H 3.850718 -1.398290 -2.524841
H 1.449249 0.381609 -3.183940
H 1.572136 -1.350997 -3.429904
H 3.360857 1.541598 -1.897433
H 1.951997 -1.228046 1.713581
H -0.362424 -1.626051 -2.258343
H -2.692198 -1.544776 -0.523893
H -1.992141 -3.156522 -0.710803
H -3.317071 -1.661336 2.713298
H -1.026778 -4.314007 1.427990
H -2.595899 -5.587144 2.788665
H -3.723632 -4.123033 2.882851
H 2.260913 1.085307 2.080566
H 0.848728 0.520674 2.941143
H 0.745242 2.015766 0.285725
H -1.367124 0.860572 0.921886
H -2.884459 2.769776 0.942325
H -1.569166 3.855126 1.362679
H -2.054304 4.105454 -0.987070
H -0.472300 3.385117 -0.844649
H -1.484574 1.127201 -1.340282
H -3.062093 1.879285 -1.452982
H -2.048495 3.397728 -3.287262
H -0.661719 2.327783 -3.285084
H -3.551289 1.543246 -3.903585
H -2.284254 1.793912 -5.086407
H -2.597210 -0.627626 -4.716009
H -1.006801 -0.154943 -4.119901
H -2.338788 -0.439087 -2.986102
H 5.438264 0.716131 -0.663133
H 0.439250 3.149781 2.831109

H -1.301526 0.820599 3.129079

2d-c18, ΔG = 3.5975 kcal/mol, population = 0.05 %
C 5.065075 -1.079870 -0.694699
C 3.869762 -1.161924 -1.640698
C 2.562317 -1.087382 -0.909734
C 2.411918 -0.545662 0.330261
C 3.575686 -0.025900 1.017787
C 4.882703 0.100027 0.243002
C 1.059792 -0.403908 1.013304
N 1.475191 -1.609793 -1.588802
C 0.262104 -1.707182 -0.985047
C 0.047380 -1.216096 0.261929
C -0.936004 -2.348776 -1.612279
C -1.958594 -2.391653 -0.454708
C -1.303648 -1.506829 0.650237
O -1.918883 -1.140739 1.644178
O -3.183834 -1.796975 -0.861297
C -2.127776 -3.790487 0.078856
C -3.292800 -4.395717 0.265912
C 0.604306 1.073994 1.101237
C 0.741262 1.847999 -0.210699
C -0.455341 2.770750 -0.481889
C -1.664631 1.978605 -0.961046
C -2.946605 2.805312 -1.091304
C -3.566781 3.239916 0.242912
C -3.955302 2.092382 1.182267
C -4.955953 1.103328 0.584851
C -5.361630 0.005774 1.568001
O 3.549846 0.344510 2.192181
O 5.971204 0.225088 1.139113
O 1.970131 2.603847 -0.255330
O -0.114367 3.719029 -1.499129
H 5.988293 -0.967098 -1.263377
H 5.142817 -1.996151 -0.104537
H 3.902926 -0.332722 -2.356913
H 3.907906 -2.082280 -2.226401
H 4.785400 1.018578 -0.356780
H 1.138616 -0.773523 2.038757
H 1.617971 -2.012511 -2.503387
H -1.325239 -1.724987 -2.419134
H -0.709730 -3.333225 -2.023608
H -3.658490 -1.546806 -0.055369
H -1.199254 -4.293671 0.333745

H -3.342349 -5.400106 0.666373
H -4.229179 -3.914120 0.013711
H 1.183754 1.583540 1.873474
H -0.431665 1.081153 1.441748
H 0.826038 1.165014 -1.058156
H -0.692802 3.309931 0.445785
H -1.403747 1.548803 -1.932476
H -1.835024 1.136363 -0.289373
H -2.737611 3.692757 -1.693531
H -3.677667 2.222345 -1.656395
H -2.876929 3.907538 0.767801
H -4.458589 3.838026 0.028153
H -3.062918 1.544356 1.499560
H -4.382895 2.519978 2.095320
H -5.845832 1.647818 0.250774
H -4.529025 0.644978 -0.311463
H -4.486737 -0.556688 1.904654
H -5.839764 0.429881 2.454818
H -6.065781 -0.697001 1.116107
H 5.575515 0.489681 1.987155
H 2.109812 3.013479 0.608008
H 0.822423 3.922672 -1.357883

2d-c2, ΔG = 3.6257 kcal/mol, population = 0.04 %
C 5.108082 -1.199483 -0.766091
C 3.890885 -1.222747 -1.687287
C 2.606346 -1.068829 -0.929697
C 2.508613 -0.492732 0.298663
C 3.713396 -0.035688 0.956950
C 5.012237 0.000032 0.158863
C 1.180209 -0.270304 1.007780
N 1.479839 -1.553768 -1.571119
C 0.284541 -1.600676 -0.929965
C 0.116936 -1.066382 0.307419
C -0.940410 -2.241484 -1.501615
C -1.935669 -2.218566 -0.320949
C -1.225743 -1.325542 0.747103
O -1.797189 -0.935976 1.757888
O -3.146356 -1.596709 -0.728831
C -2.142726 -3.594483 0.255585
C -3.324033 -4.156474 0.473386
C 0.783482 1.225763 1.099313
C 0.707340 1.951389 -0.238480
C -0.145325 3.226632 -0.195287

C -1.621288 3.006384 0.119274
C -2.324230 1.975020 -0.763814
C -3.839542 1.916176 -0.555510
C -4.261314 1.614174 0.886470
C -5.748634 1.275865 1.038406
C -6.119937 -0.113771 0.519261
O 3.733262 0.354427 2.125967
O 6.121293 0.070442 1.035523
O 2.016790 2.297489 -0.742760
O -0.052448 3.888951 -1.464085
H 6.025192 -1.148044 -1.353285
H 5.143221 -2.111056 -0.164691
H 3.954543 -0.401456 -2.410772
H 3.865891 -2.147601 -2.266499
H 4.962007 0.915010 -0.452729
H 1.275108 -0.621089 2.038892
H 1.584560 -1.996240 -2.472322
H -1.337755 -1.645967 -2.325483
H -0.740920 -3.246172 -1.876340
H -3.612810 -1.311891 0.069716
H -1.227796 -4.120013 0.514585
H -3.400424 -5.146645 0.903899
H -4.247883 -3.652791 0.217994
H 1.487695 1.747127 1.751696
H -0.187757 1.265862 1.592375
H 0.298551 1.292233 -1.005186
H 0.278622 3.883191 0.578864
H -1.713482 2.728030 1.170323
H -2.119662 3.975591 0.018519
H -2.112634 2.197841 -1.812378
H -1.915035 0.981332 -0.570153
H -4.290643 2.865678 -0.865557
H -4.242352 1.151191 -1.224438
H -3.659603 0.786206 1.277576
H -4.029435 2.477083 1.516252
H -6.023931 1.341051 2.095001
H -6.345768 2.033497 0.519548
H -5.593219 -0.889783 1.083559
H -7.189298 -0.306720 0.626619
H -5.870818 -0.234921 -0.536790
H 5.755696 0.368317 1.886179
H 2.492558 2.771501 -0.047800
H 0.868634 3.788289 -1.744951

2d-c25, ΔG = 3.6345 kcal/mol, population = 0.04 %
C 5.200074 -1.281738 -0.600151
C 4.027397 -1.342261 -1.575900
C 2.710192 -1.160193 -0.884168
C 2.555534 -0.511434 0.303252
C 3.729243 -0.030387 0.998152
C 5.068673 -0.042155 0.266034
C 1.196094 -0.279413 0.948751
N 1.618981 -1.703341 -1.533165
C 0.402193 -1.743661 -0.930318
C 0.179243 -1.144858 0.267275
C -0.787242 -2.448980 -1.502813
C -1.822336 -2.378100 -0.358298
C -1.177309 -1.392358 0.665515
O -1.803586 -0.929455 1.610998
O -3.040201 -1.823748 -0.835525
C -2.002664 -3.718667 0.305325
C -3.171763 -4.302823 0.530244
C 0.758711 1.208212 0.956091
C 0.352304 1.787913 -0.390501
C -0.191383 3.219583 -0.275322
C -1.501118 3.359259 0.492998
C -2.684329 2.569144 -0.068661
C -3.981718 2.869650 0.685029
C -5.247105 2.363593 -0.014998
C -5.314480 0.851401 -0.250463
C -5.288792 0.025461 1.036304
O 3.695455 0.413354 2.148304
O 6.131284 0.057631 1.195287
O 1.434639 1.785917 -1.346368
O -0.388028 3.753106 -1.591240
H 6.144007 -1.260080 -1.145257
H 5.201354 -2.165956 0.041768
H 4.125200 -0.550458 -2.327826
H 4.029466 -2.290198 -2.116959
H 5.062540 0.844966 -0.387868
H 1.271798 -0.567416 2.000642
H 1.763283 -2.189159 -2.406328
H -1.169715 -1.917072 -2.375987
H -0.550754 -3.469655 -1.806050
H -3.515485 -1.477495 -0.066439
H -1.079435 -4.195843 0.622066
H -3.230069 -5.263778 1.025037
H -4.102447 -3.846564 0.217433

H 1.559995 1.811700 1.389930
H -0.093813 1.284949 1.631640
H -0.407726 1.161960 -0.858453
H 0.574027 3.819879 0.238494
H -1.329808 3.086865 1.537354
H -1.755861 4.423367 0.497818
H -2.811329 2.819524 -1.125281
H -2.475876 1.497659 -0.024091
H -3.918556 2.454998 1.696189
H -4.073216 3.953674 0.809992
H -6.119891 2.665743 0.573967
H -5.336759 2.872665 -0.980615
H -6.233883 0.629030 -0.799946
H -4.492607 0.543947 -0.903239
H -4.330316 0.104217 1.553020
H -6.067283 0.357458 1.728374
H -5.473254 -1.033212 0.832615
H 5.724908 0.395678 2.011896
H 2.219582 2.157665 -0.923427
H 0.354381 3.423863 -2.118655

2d-c150, ΔG = 3.6628 kcal/mol, population = 0.04 %
C 3.823755 -0.539342 -2.509365
C 2.309765 -0.625233 -2.673258
C 1.599898 -0.794938 -1.362629
C 2.110638 -0.429218 -0.154901
C 3.478910 0.055218 -0.107978
C 4.156906 0.458964 -1.414491
C 1.351175 -0.584354 1.163881
N 0.342884 -1.364244 -1.455201
C -0.415817 -1.569153 -0.352692
C 0.004534 -1.205700 0.889294
C -1.791694 -2.153130 -0.404514
C -2.195282 -2.237430 1.081431
C -1.078771 -1.429313 1.802883
O -1.230621 -1.012679 2.951119
O -3.441557 -1.589809 1.285042
C -2.174297 -3.657759 1.586876
C -3.163407 -4.237519 2.253015
C 1.295653 0.730258 1.984003
C 0.729301 1.968459 1.289160
C -0.785202 1.924020 1.051888
C -1.286180 3.125033 0.264745
C -2.752852 3.029820 -0.164321

C -3.096541 1.827461 -1.051721
C -2.232383 1.703738 -2.307733
C -2.740908 0.654278 -3.294902
C -1.844969 0.506950 -4.522798
O 4.121030 0.157591 0.937332
O 5.553258 0.570807 -1.221695
O 1.062974 3.135186 2.057111
O -1.476331 1.896765 2.314674
H 4.289015 -0.236462 -3.447485
H 4.228714 -1.515672 -2.232450
H 1.929039 0.289494 -3.142212
H 2.043771 -1.448867 -3.338592
H 3.740213 1.440994 -1.687310
H 1.917964 -1.278553 1.794352
H -0.004102 -1.639694 -2.361977
H -2.478166 -1.487872 -0.929270
H -1.802596 -3.117300 -0.914437
H -3.468834 -1.315901 2.213885
H -1.256515 -4.200855 1.380088
H -3.080450 -5.260665 2.596343
H -4.087476 -3.714111 2.463389
H 2.314226 0.971619 2.278536
H 0.739492 0.535011 2.901600
H 1.222150 2.099125 0.322938
H -1.012517 1.015189 0.494779
H -1.139129 4.023095 0.868378
H -0.649664 3.237628 -0.616425
H -3.385948 3.007962 0.725969
H -3.007556 3.947797 -0.703637
H -3.030674 0.902812 -0.469777
H -4.146290 1.912241 -1.350829
H -2.187130 2.675764 -2.812883
H -1.201718 1.458336 -2.031426
H -2.826423 -0.312208 -2.788180
H -3.755287 0.919448 -3.609946
H -2.225586 -0.252412 -5.209022
H -1.775970 1.449676 -5.071808
H -0.829716 0.220024 -4.236812
H 5.677306 0.588318 -0.256588
H 0.440369 3.151837 2.798006
H -1.409437 1.003690 2.685751

Attached figures of compounds 1-6

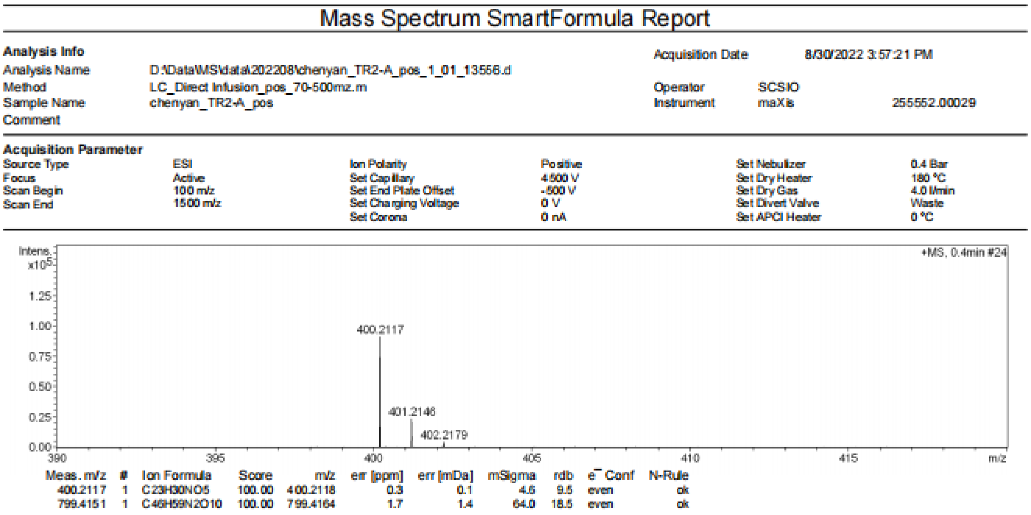

Figure S1. HRESIMS spectrum of compound **1**

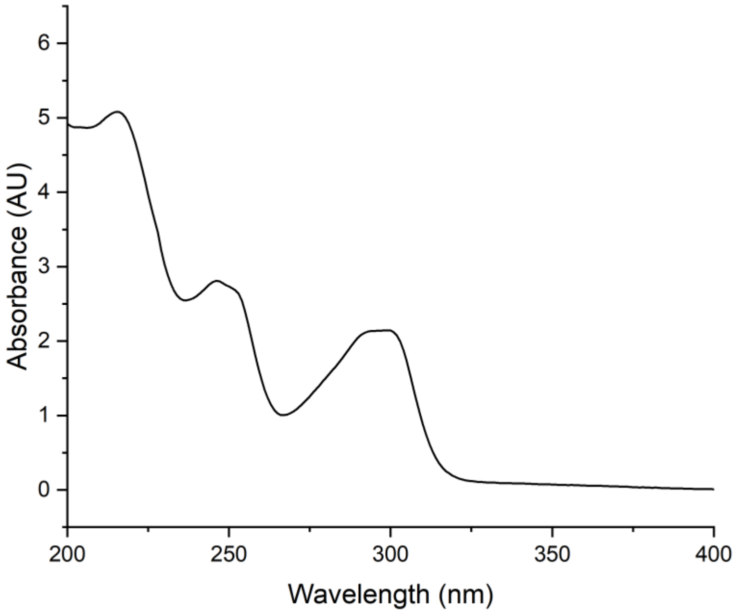

Figure S2. UV spectrum of compound **1**

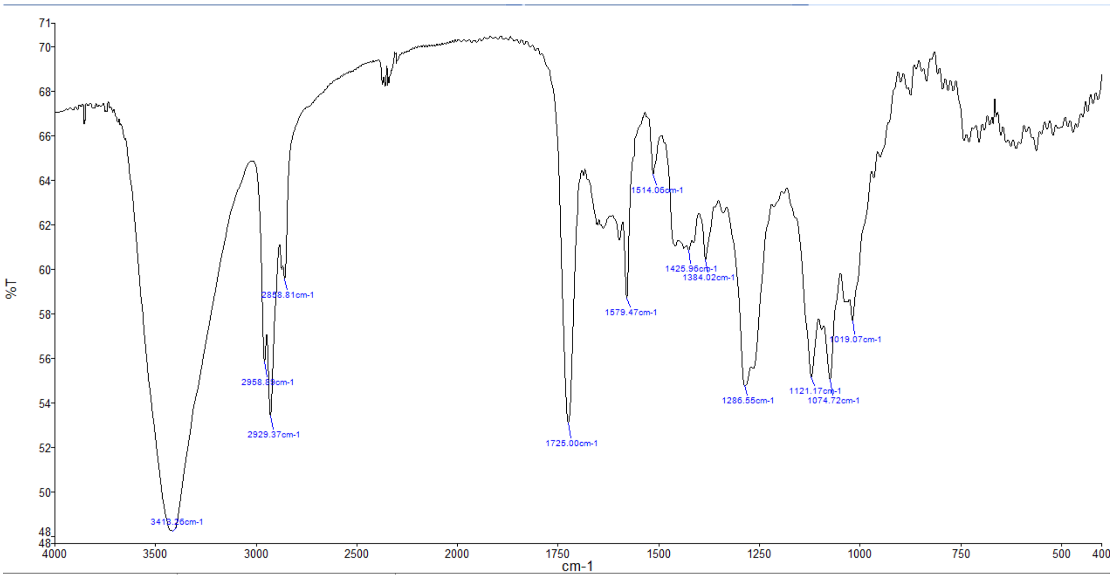

Figure S3. IR spectrum of compound **1**

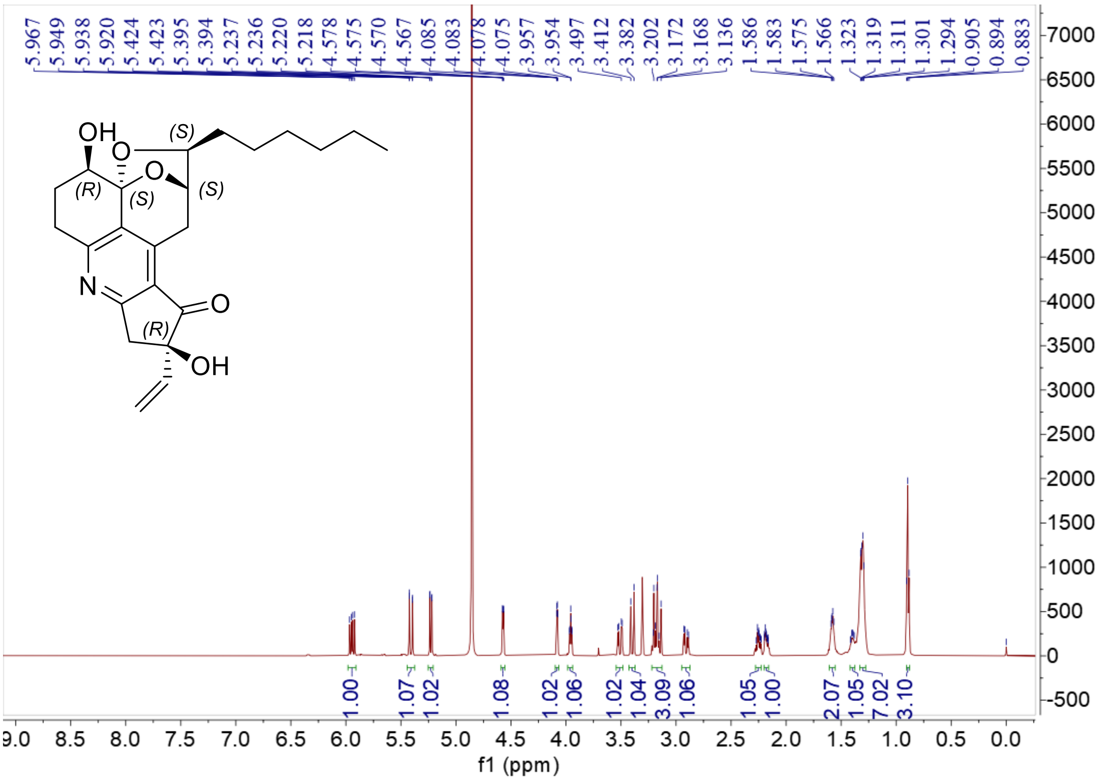

Figure S4. ^{1}H NMR spectrum (600 MHz, CD_3OD) of compound **1**

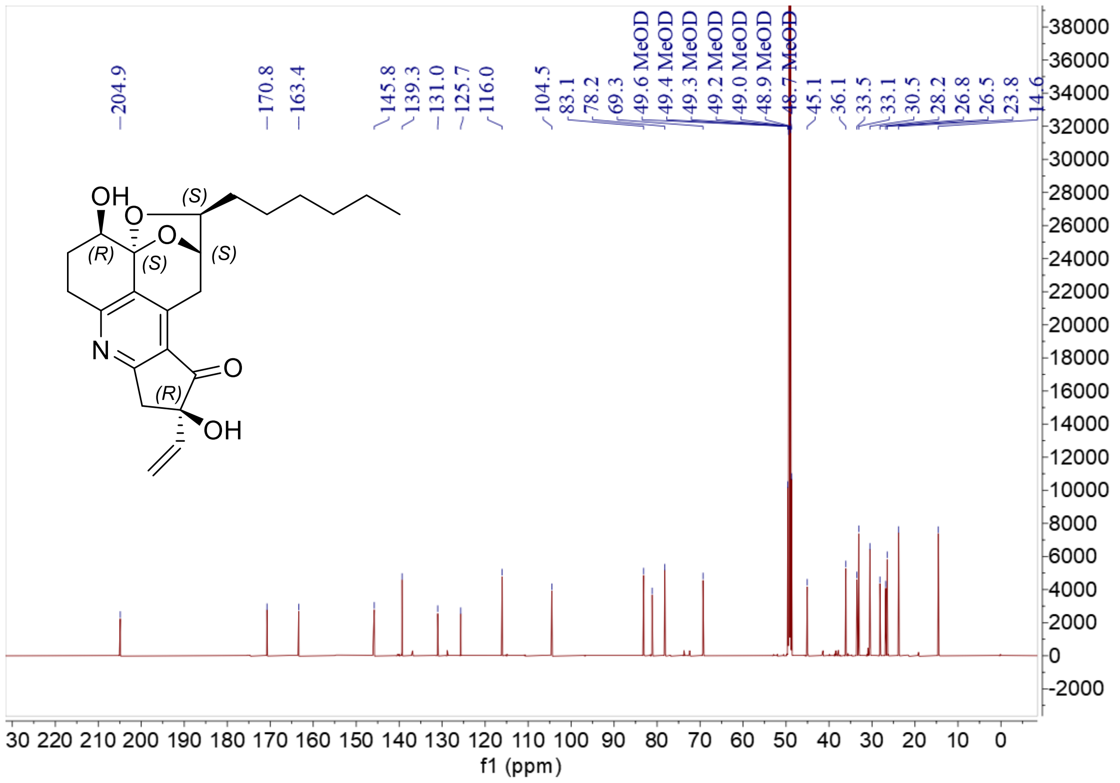

Figure S5. ^{13}C NMR spectrum (150 MHz, CD_3OD) of compound **1**

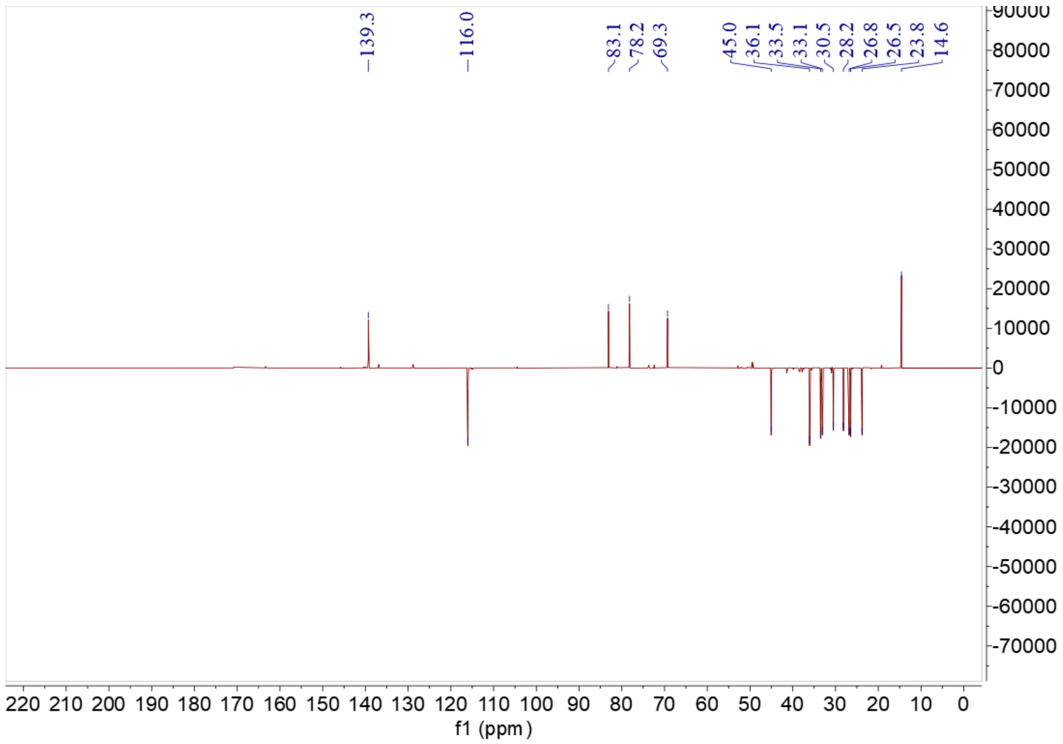

Figure S6. DEPT 135 spectrum of compound **1** recorded in CD_3OD

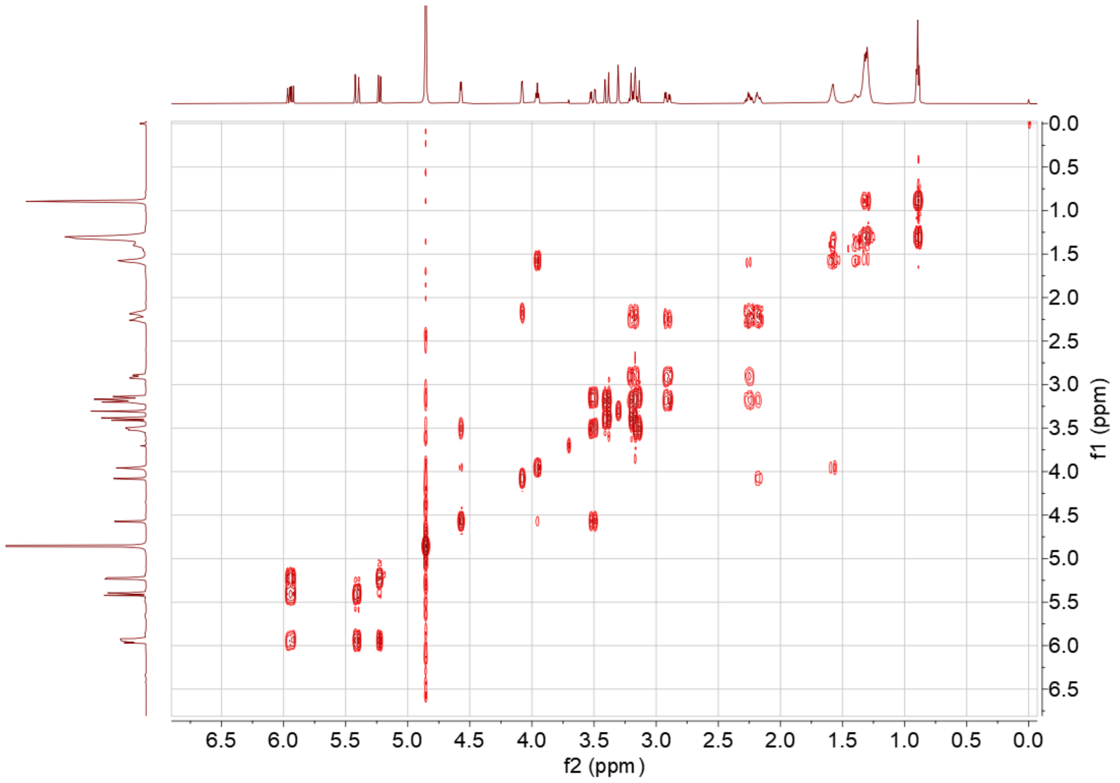

Figure S7. ^{1}H-^{1}H COSY spectrum of compound **1** recorded in CD_3OD

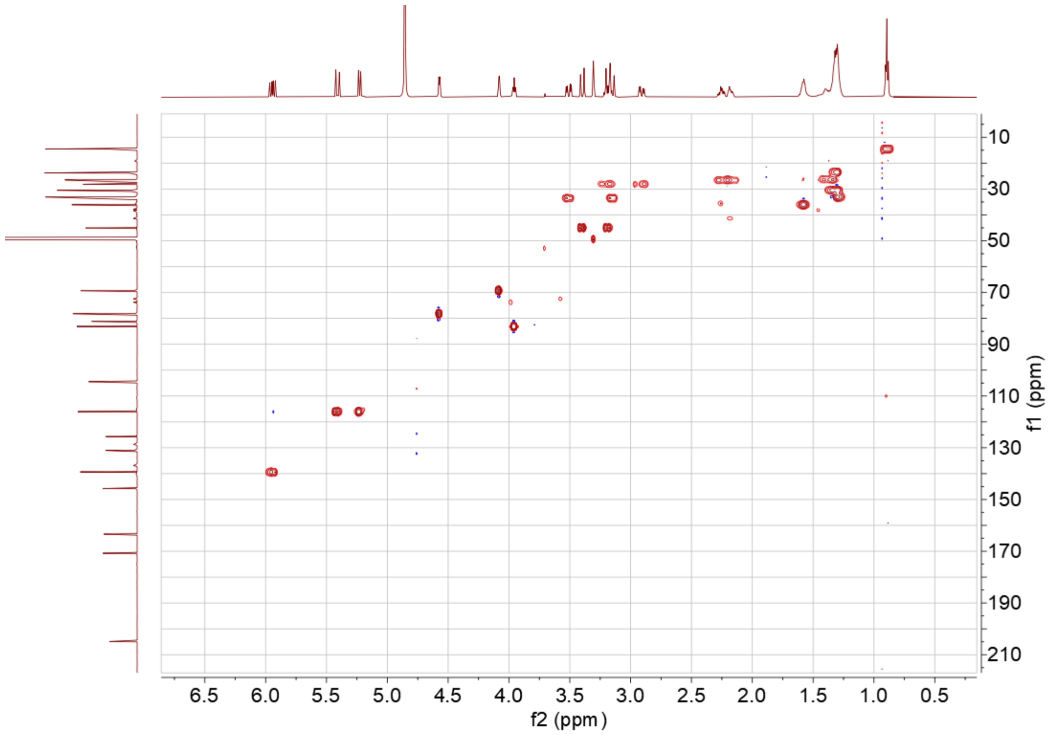

Figure S8. HSQC spectrum of compound **1** recorded in CD_3OD

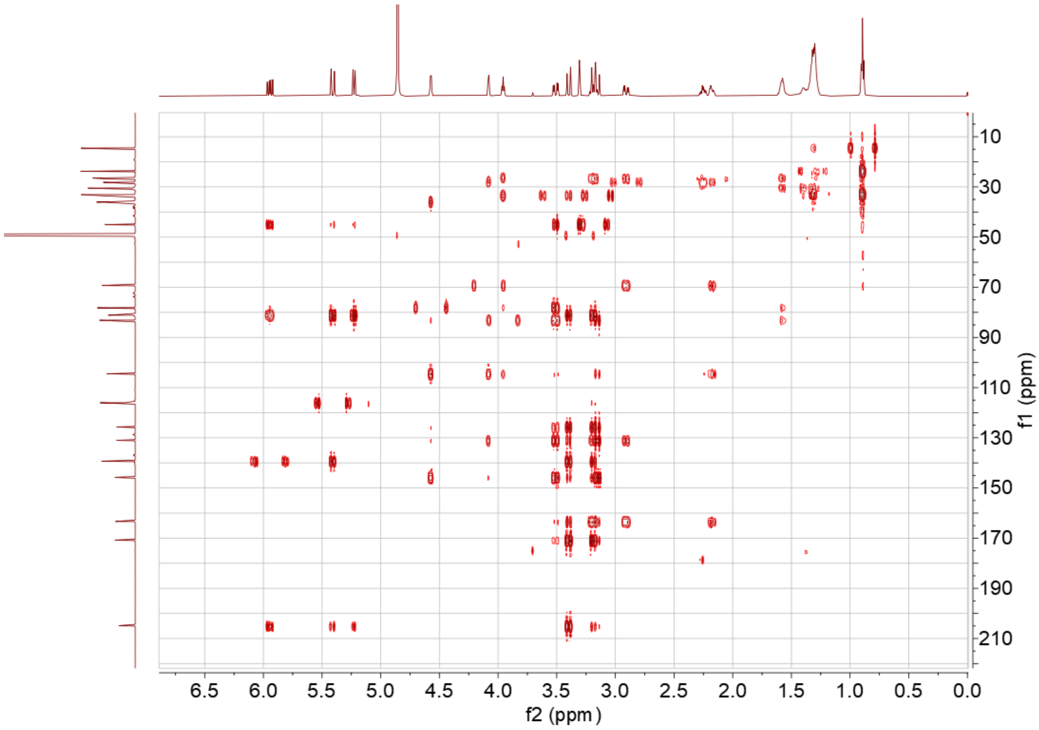

Figure S9. HMBC spectrum of compound **1** recorded in CD_3OD

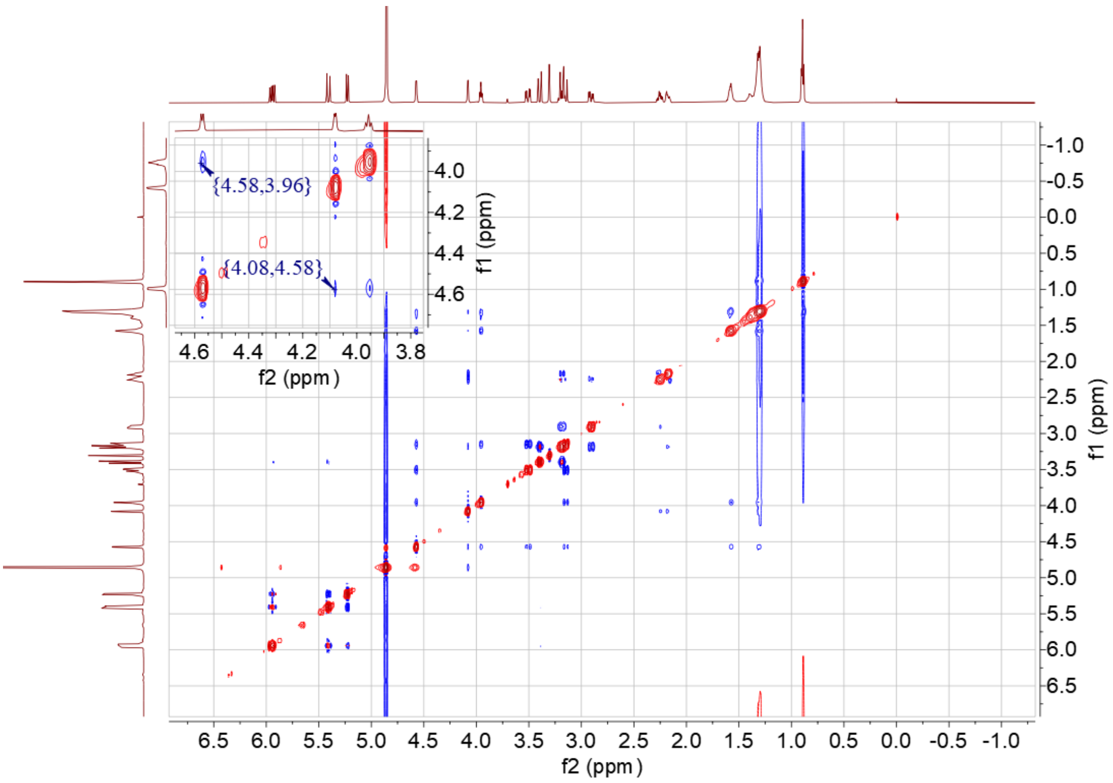

Figure S10. NOESY spectrum of compound **1** recorded in CD_3OD

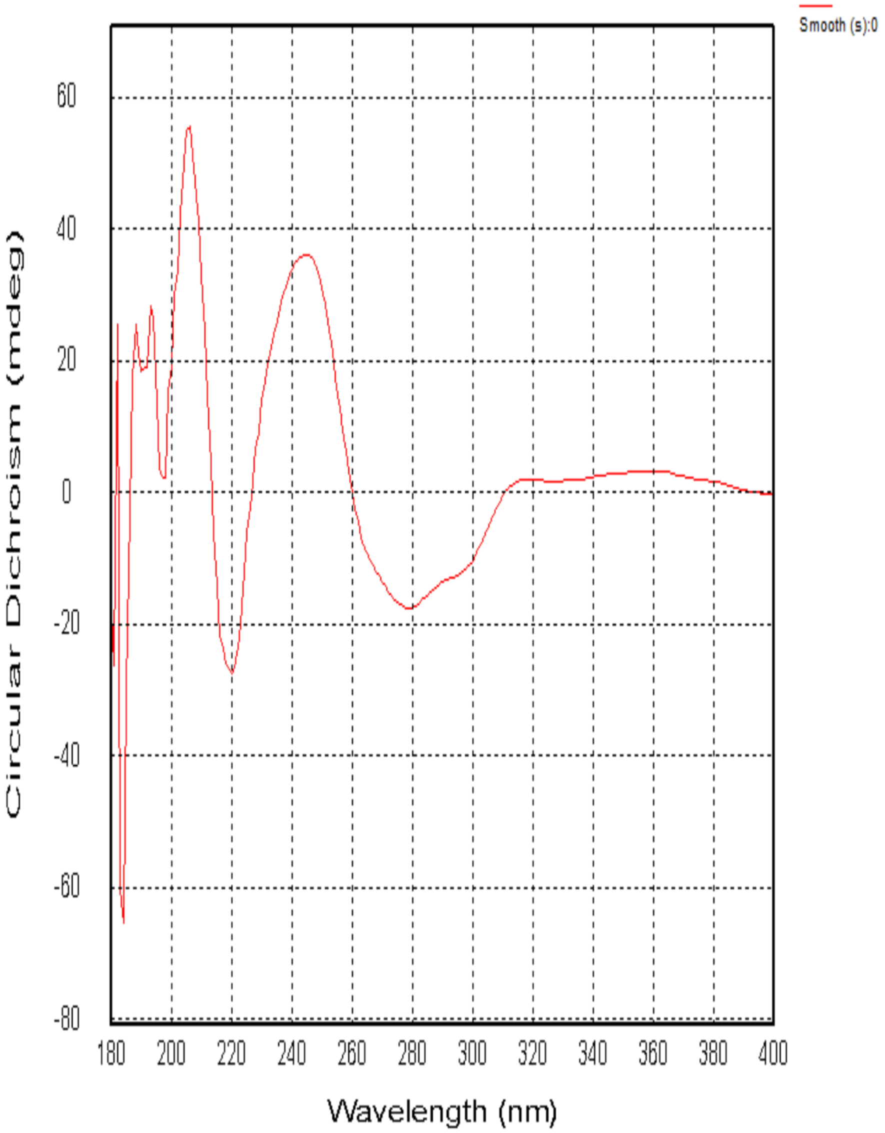

Figure S11. CD spectrum of compound **1**

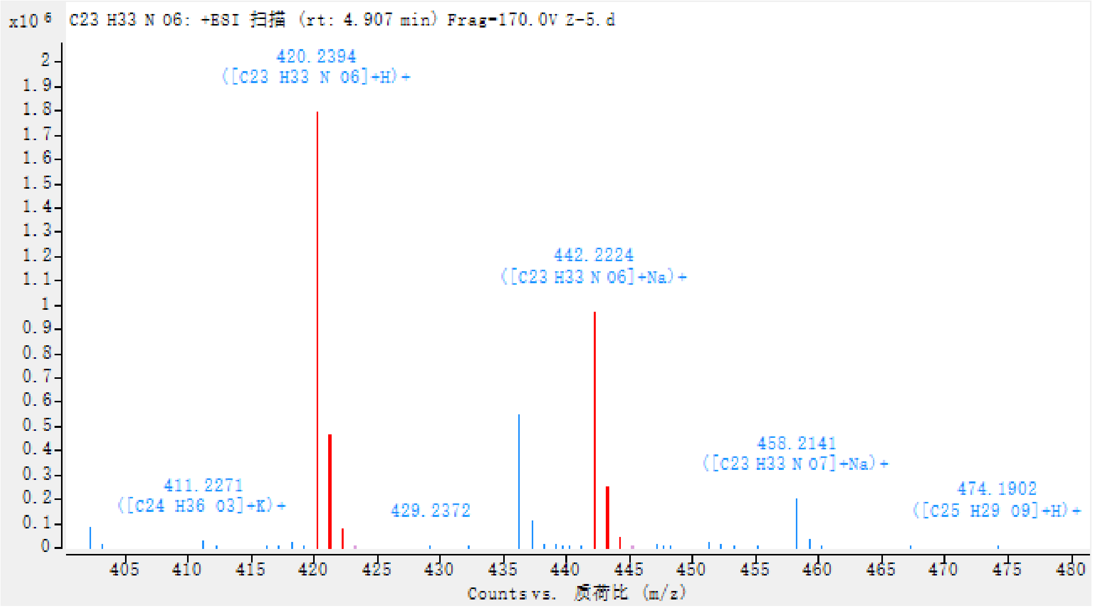

Figure S12. HRESIMS spectrum of compound **2**

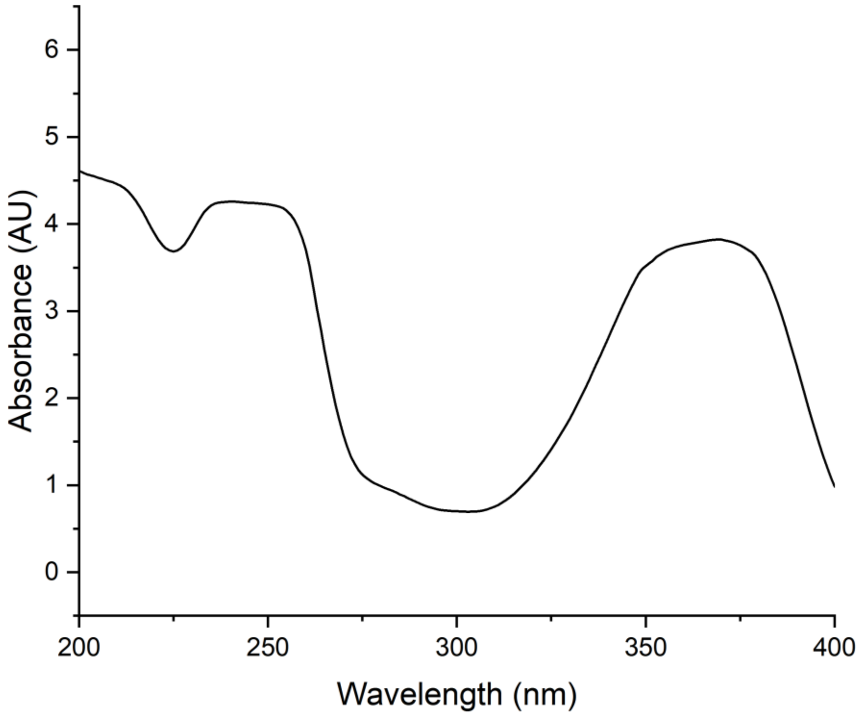

Figure S13. UV spectrum of compound **2**

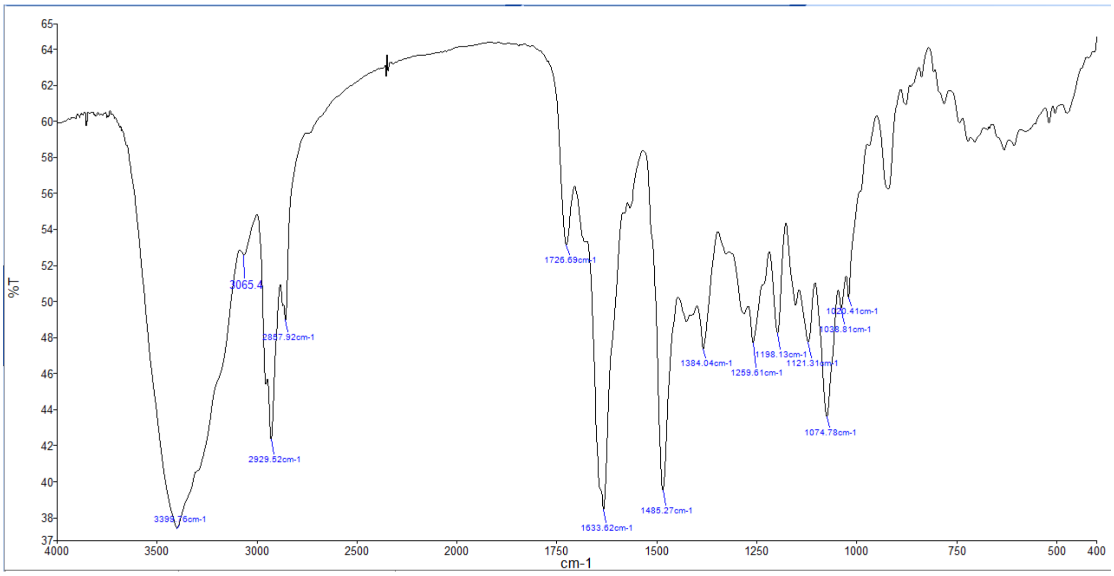

Figure S14. IR spectrum of compound **2**

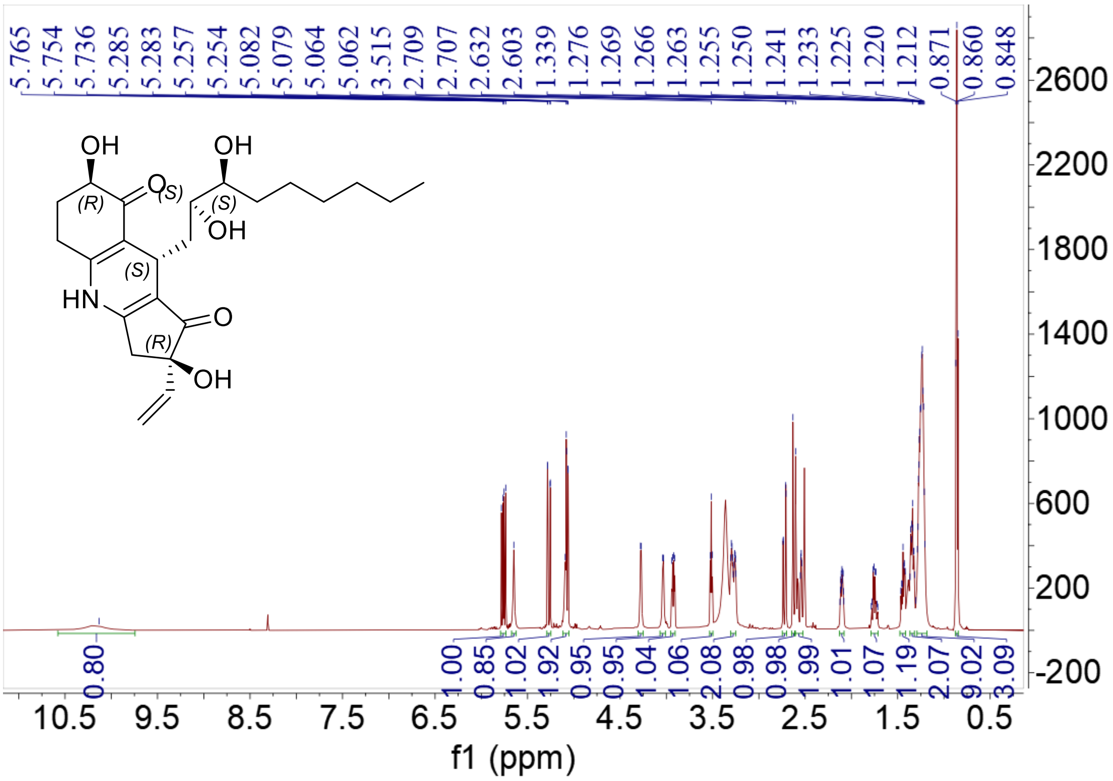

Figure S15. ^{1}H NMR spectrum (600 MHz, DMSO-d_6) of compound **2**

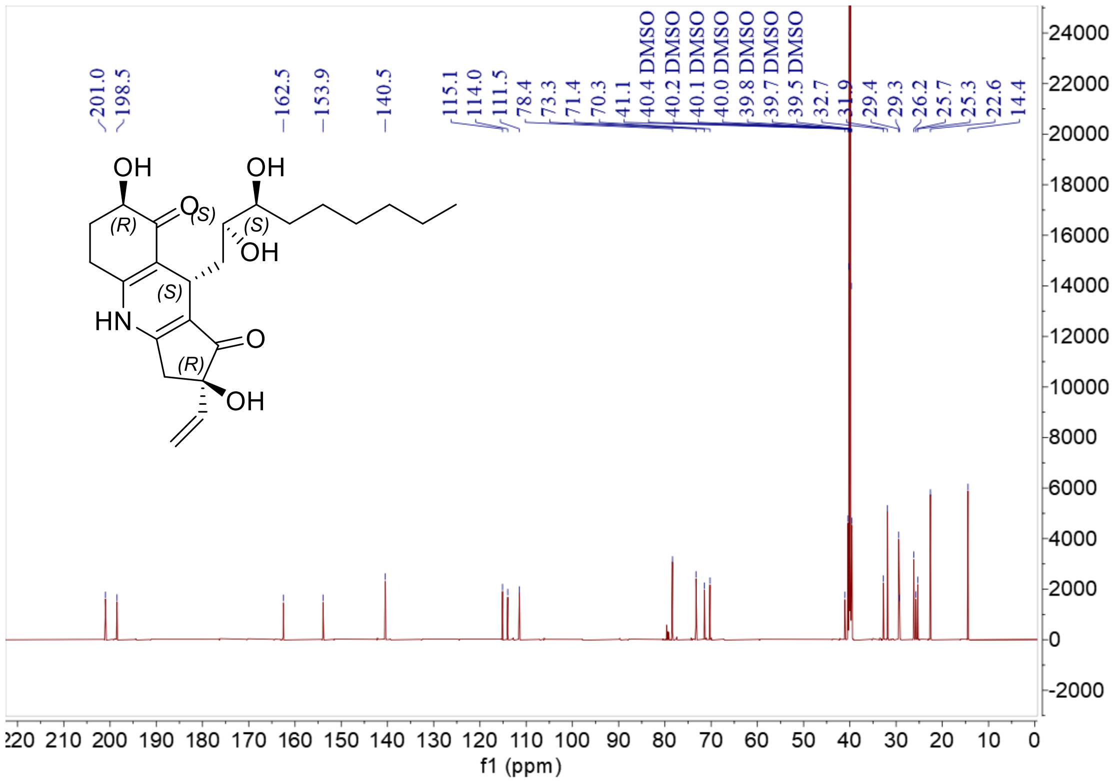

Figure S16. ^{13}C NMR spectrum (150 MHz, DMSO-d_6) of compound **2**

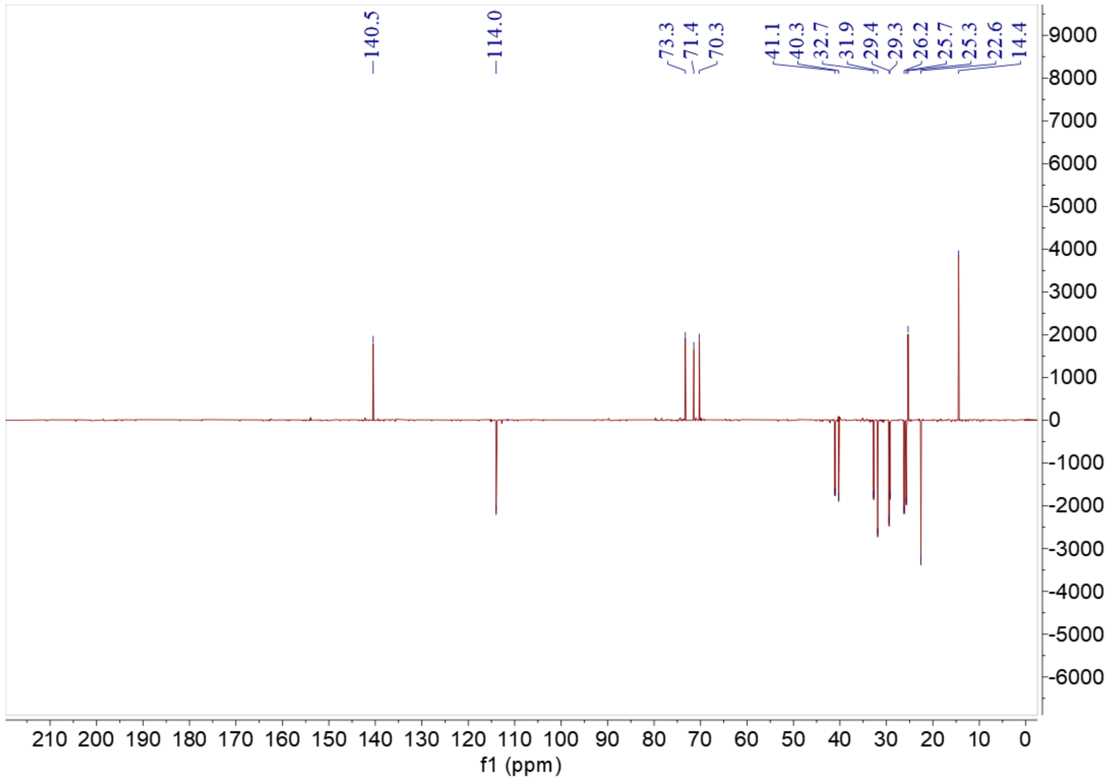

Figure S17. DEPT 135 spectrum of compound **2** recorded in DMSO-d_6

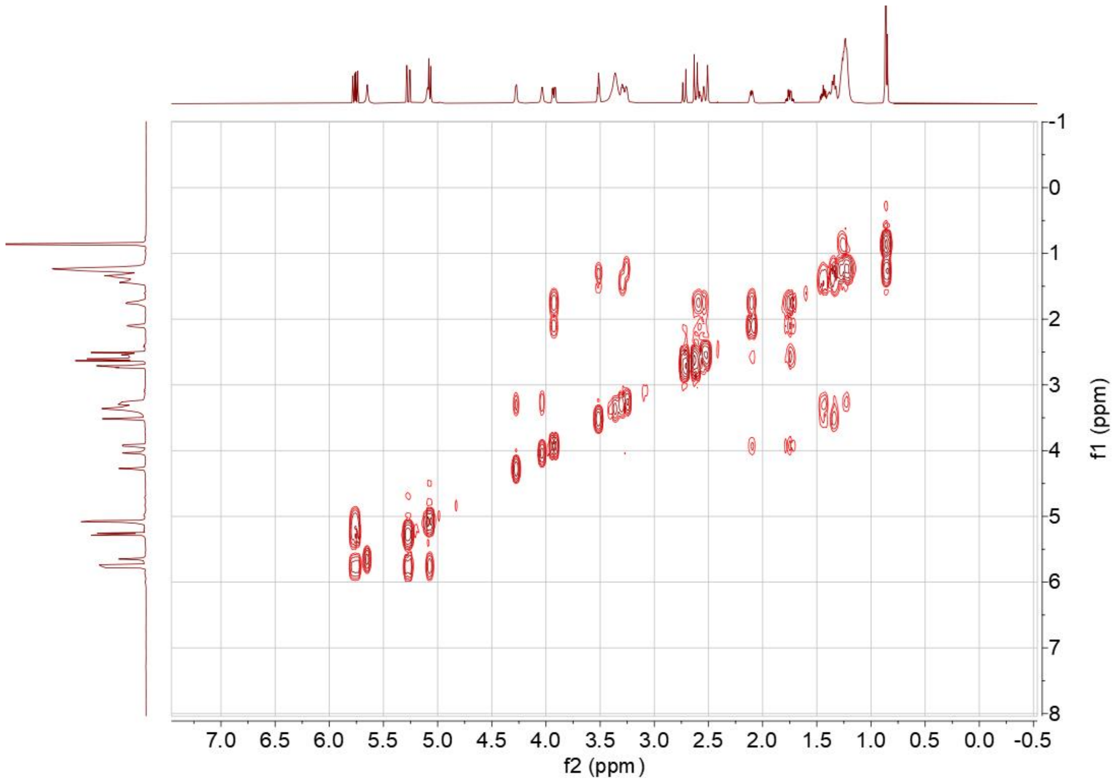

Figure S18. ^{1}H-^{1}H COSY spectrum of compound **2** recorded in DMSO-d_6

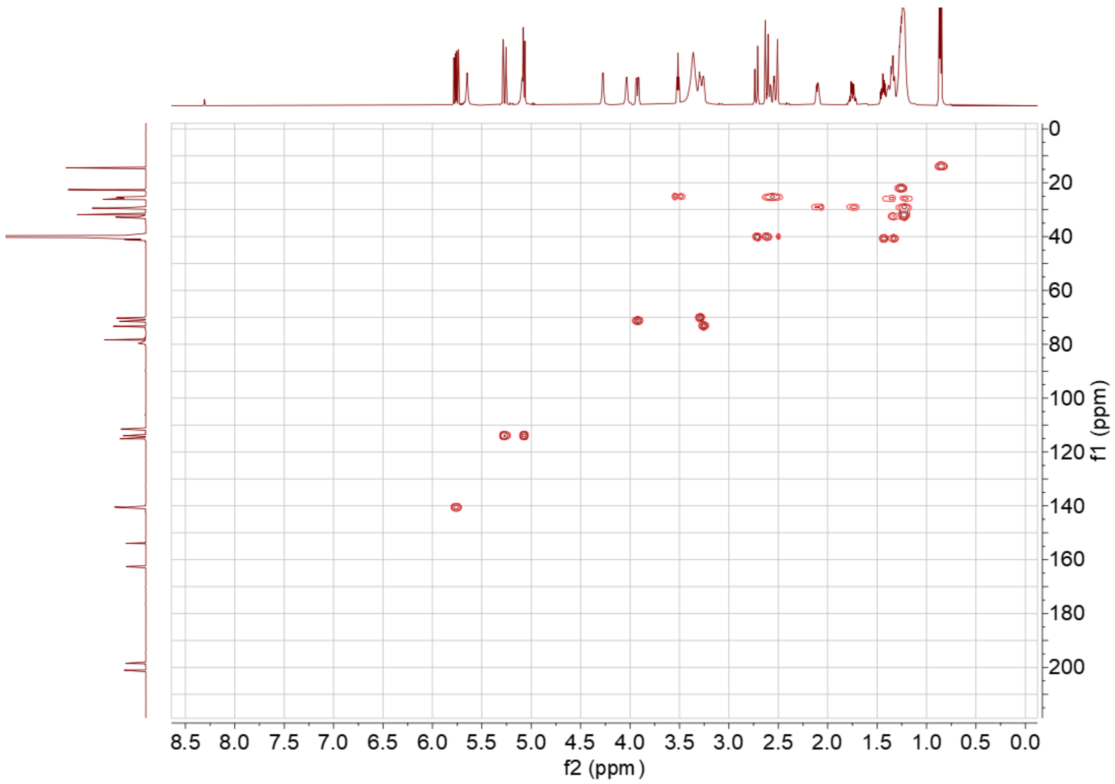

Figure S19. HSQC spectrum of compound **2** recorded in DMSO-d_6

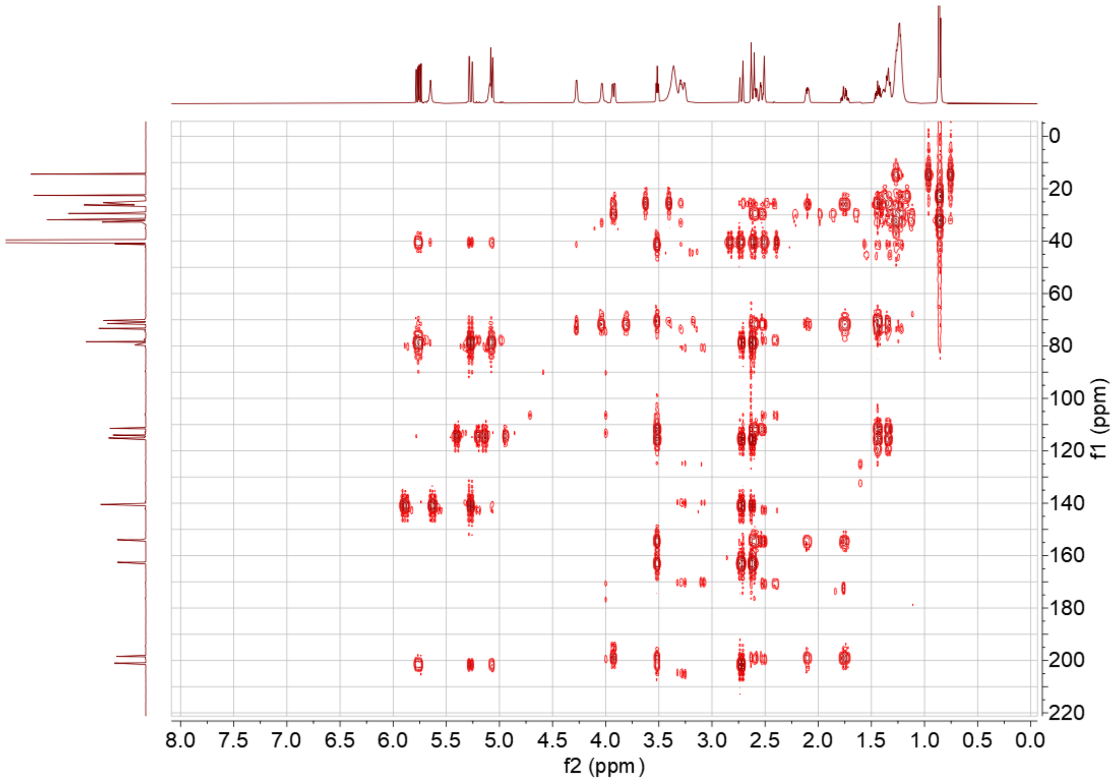

Figure S20. HMBC spectrum of compound **2** recorded in DMSO-d_6

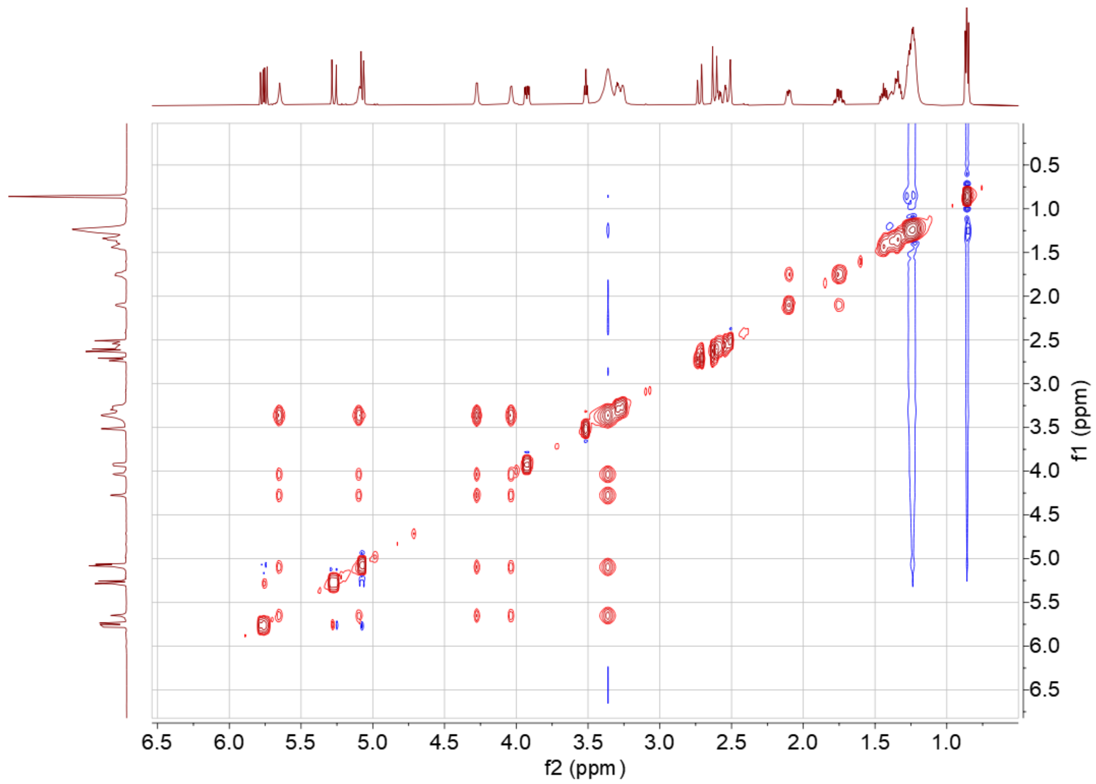

Figure S21. NOESY spectrum of compound **2** recorded in DMSO-d_6

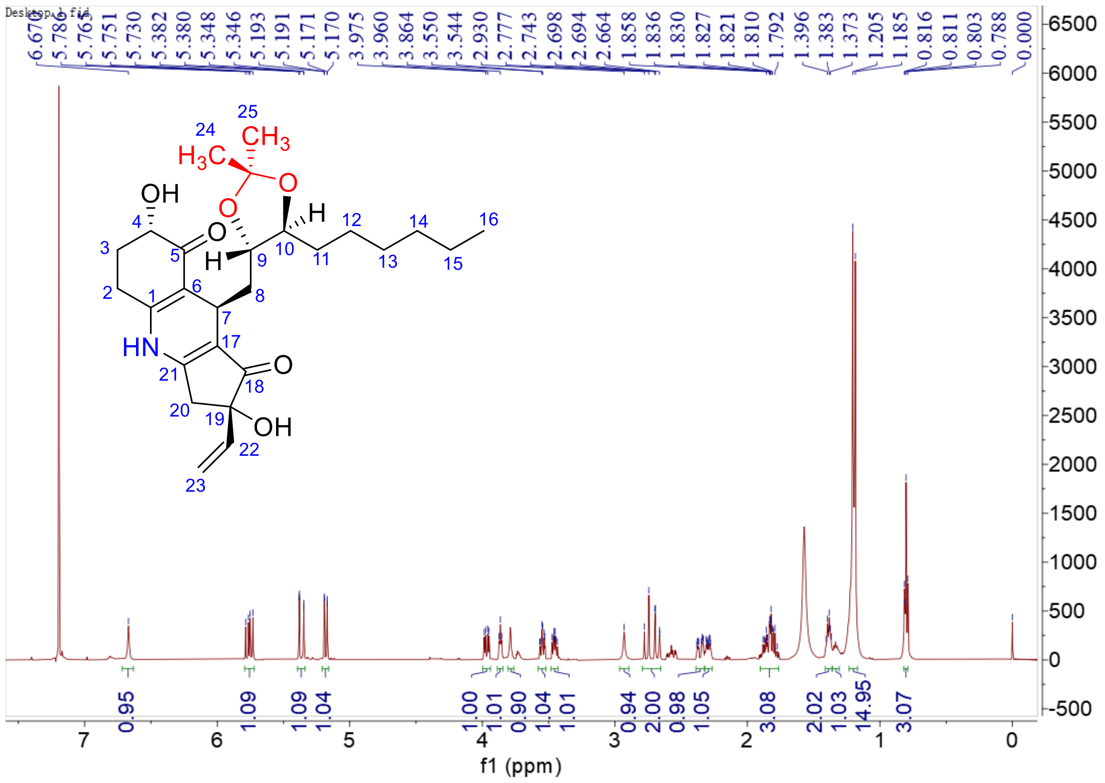

Figure S22. ^{1}H NMR spectrum (500 MHz, $CDCl_3$) of acetonylidene derivative of **2**

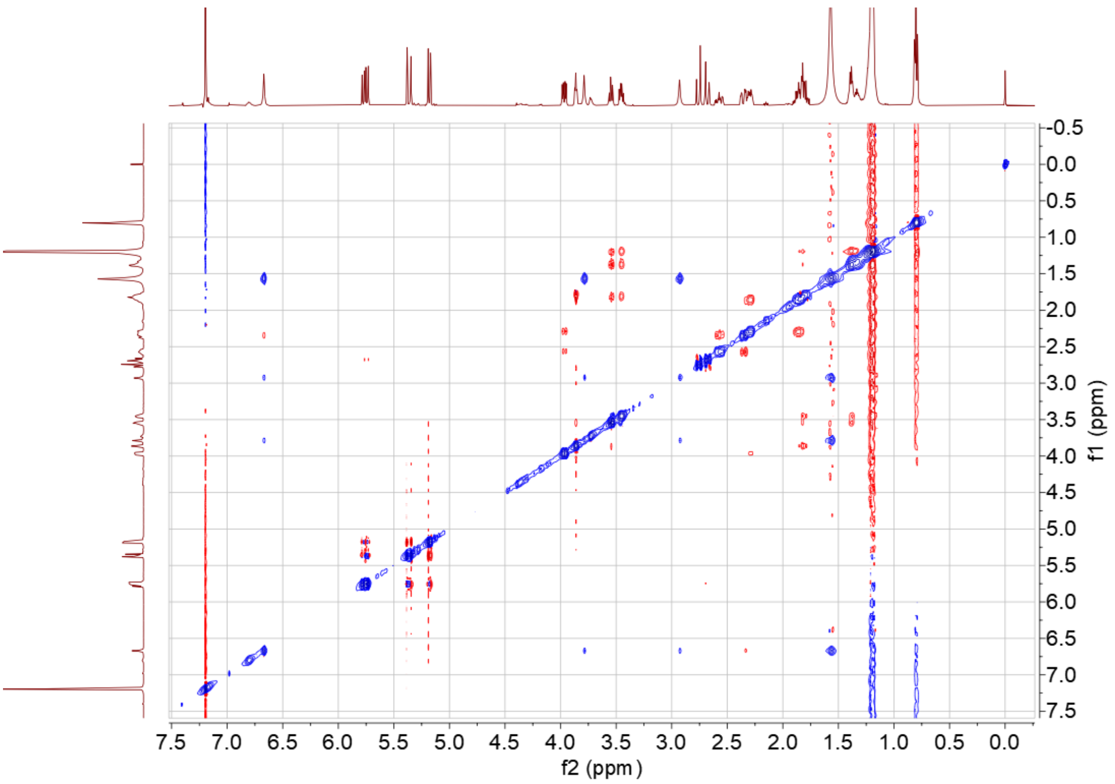

Figure S23. NOESY spectrum of acetonylidene derivative of **2** recorded in $CDCl_3$

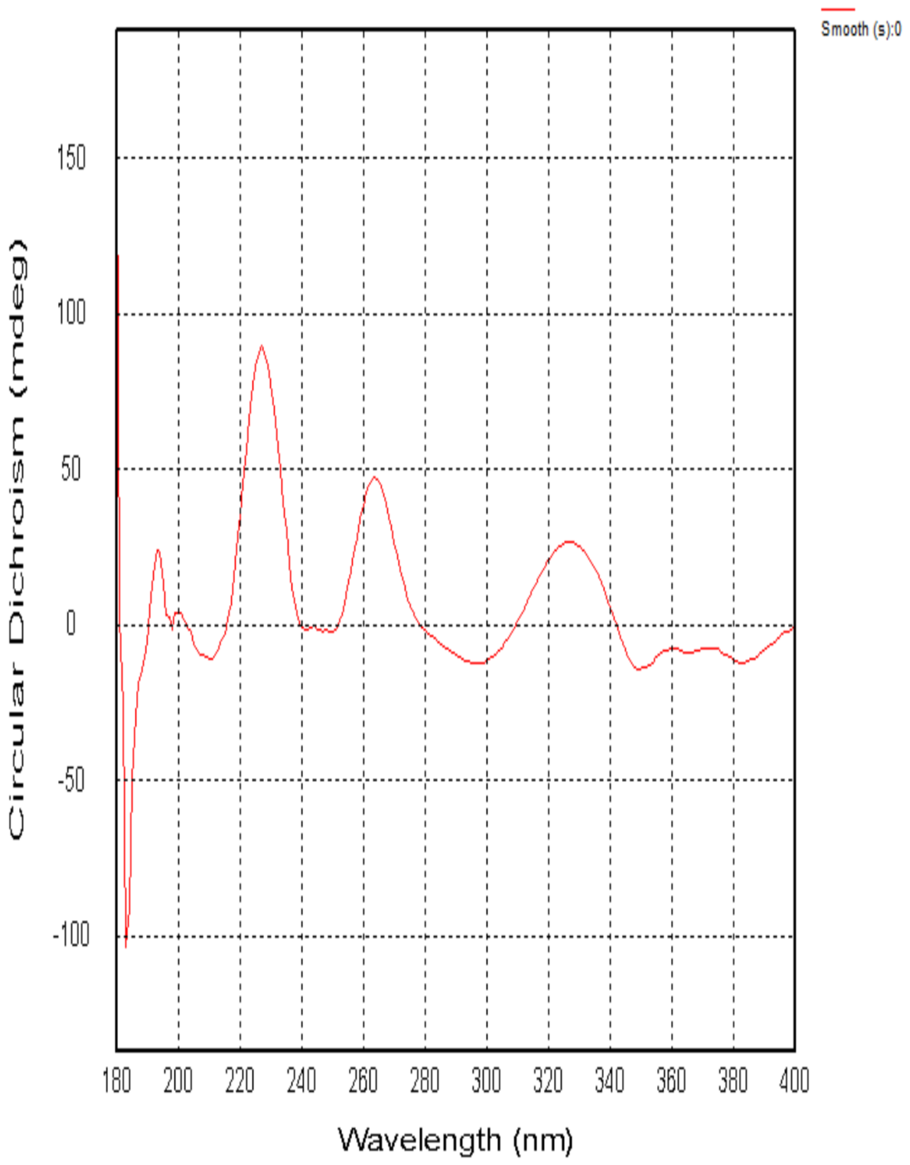

Figure S24. CD spectrum of compound **2**

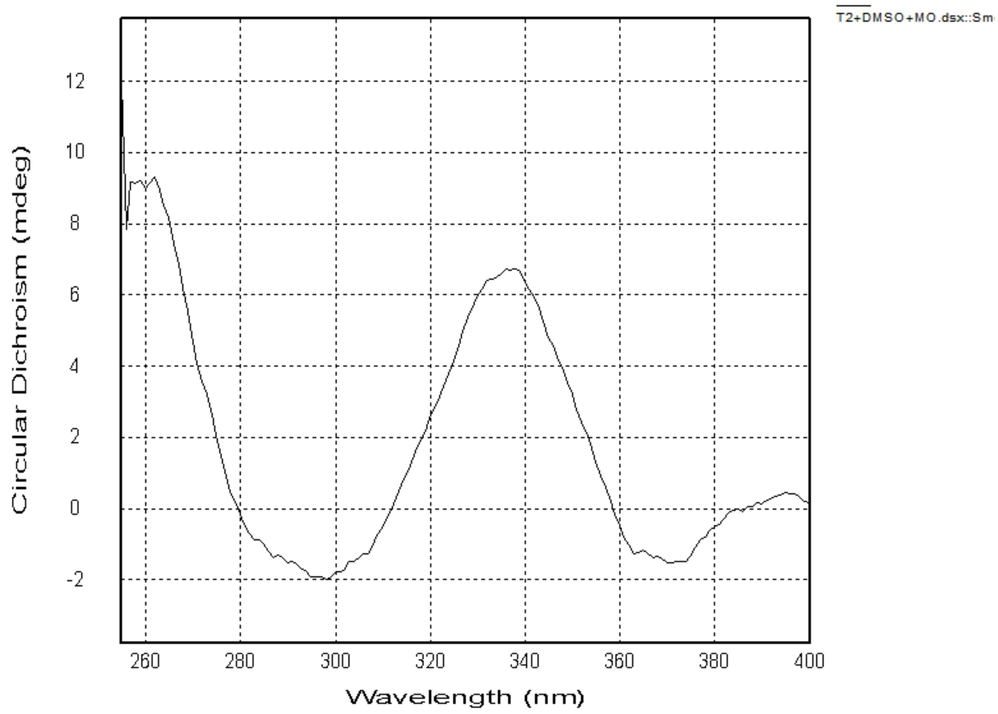

Figure S25. Mo-ICD spectrum of compound **2**

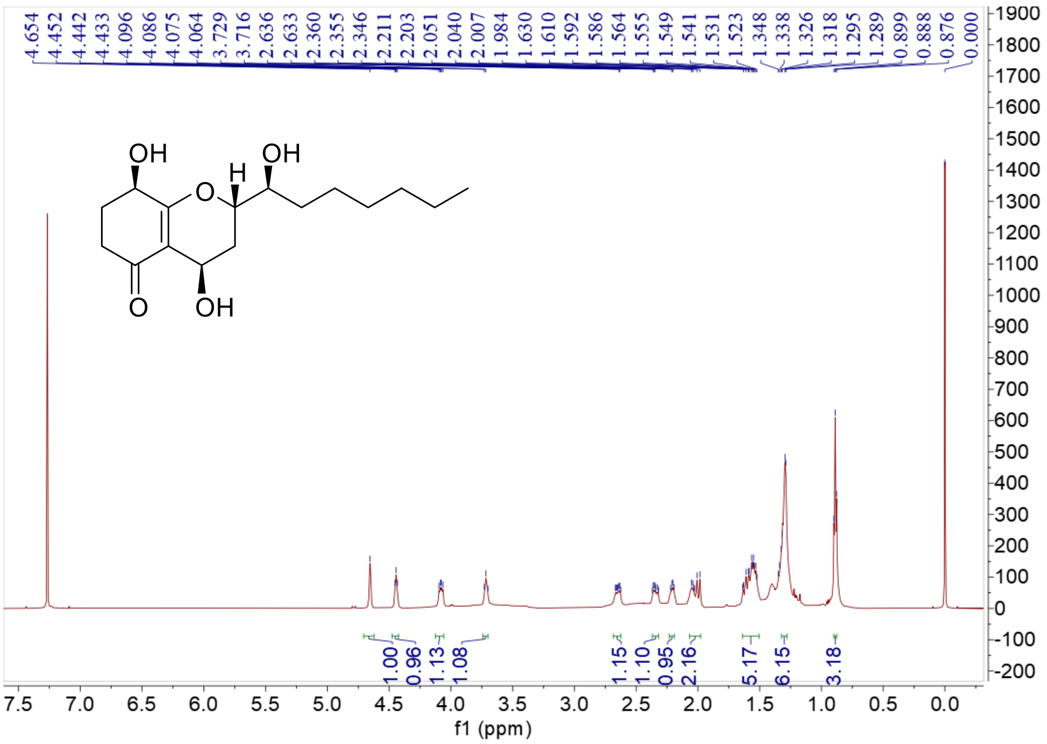

Figure S26. ^{1}H NMR spectrum (600 MHz, $CDCl_3$) of compound **3**

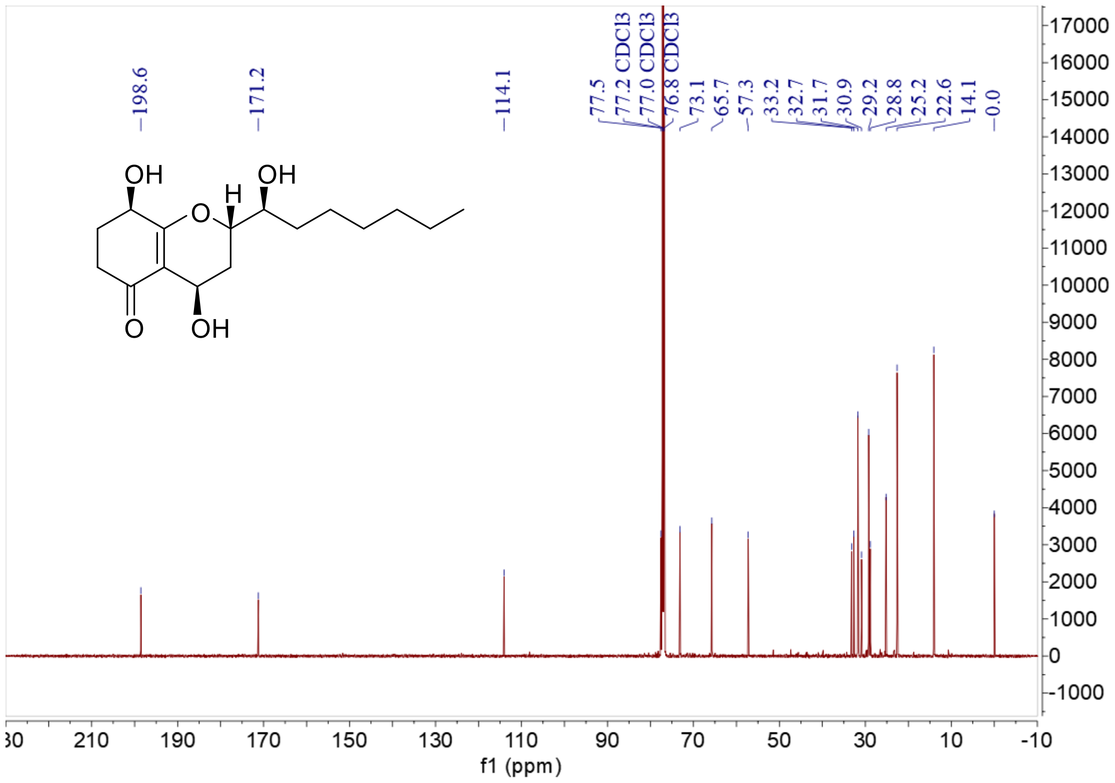

Figure S27. ^{13}C NMR spectrum (150 MHz, $CDCl_3$) of compound **3**

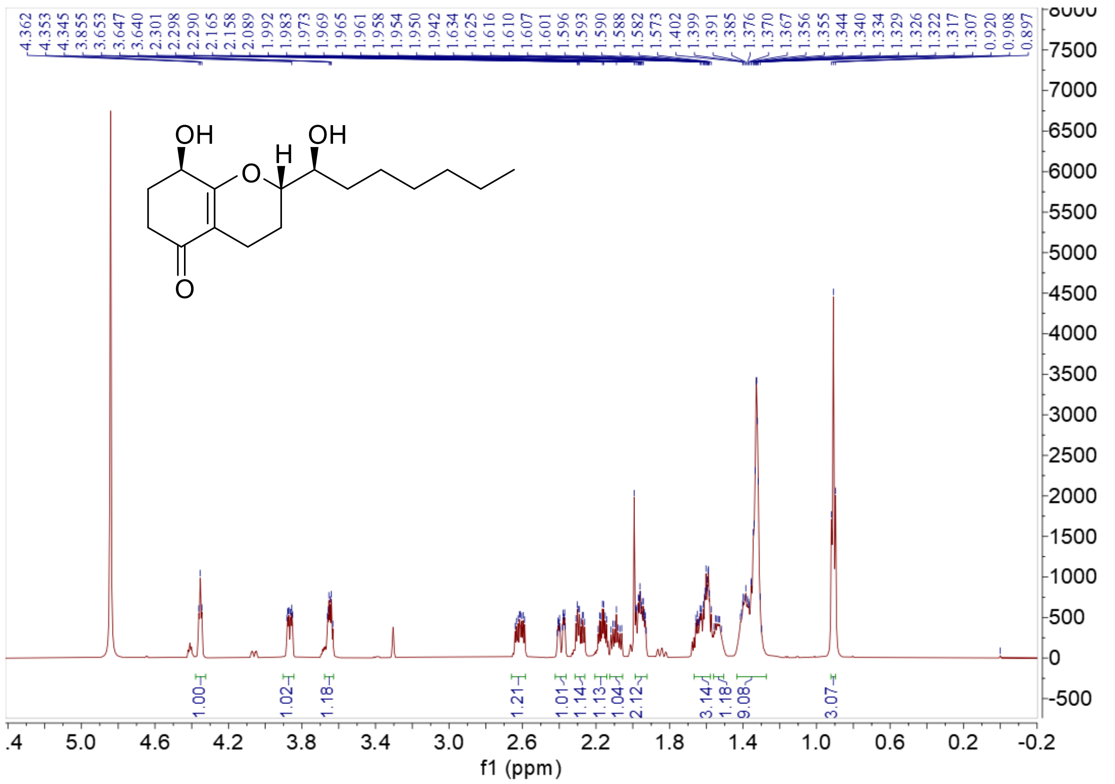

Figure S28. ^{1}H NMR spectrum (600 MHz, CD_3OD) of compound **4**

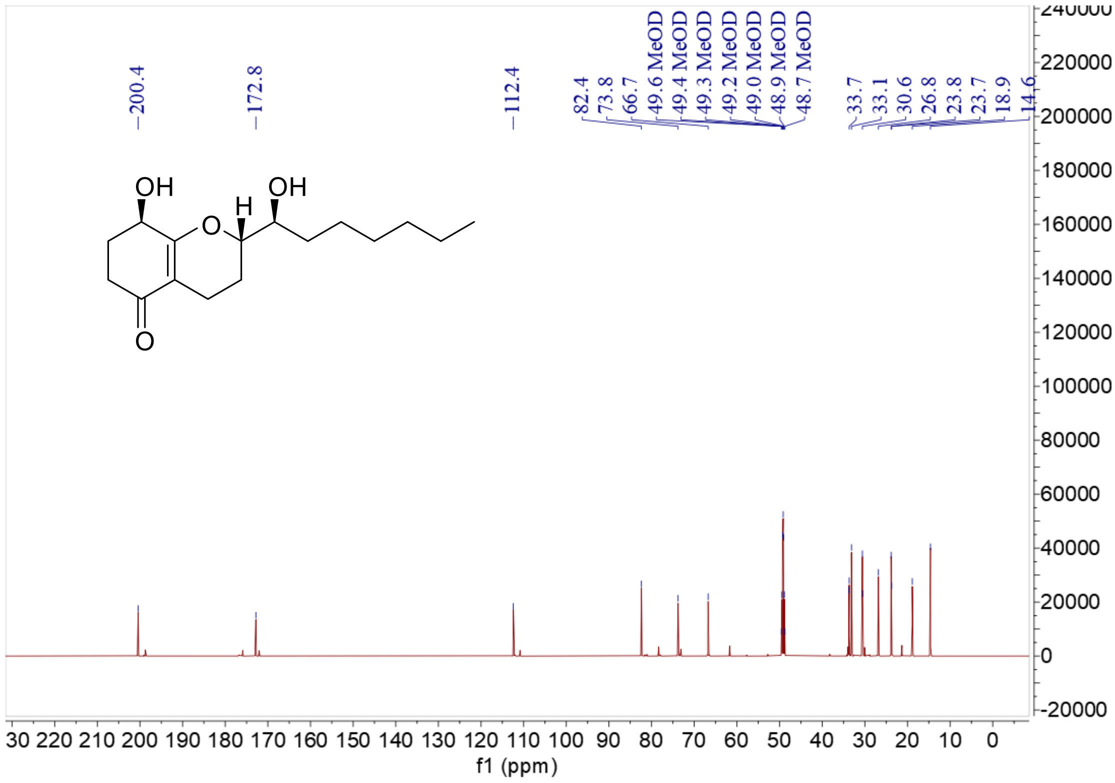

Figure S29. ^{13}C NMR spectrum (150 MHz, CD_3OD) of compound **4**

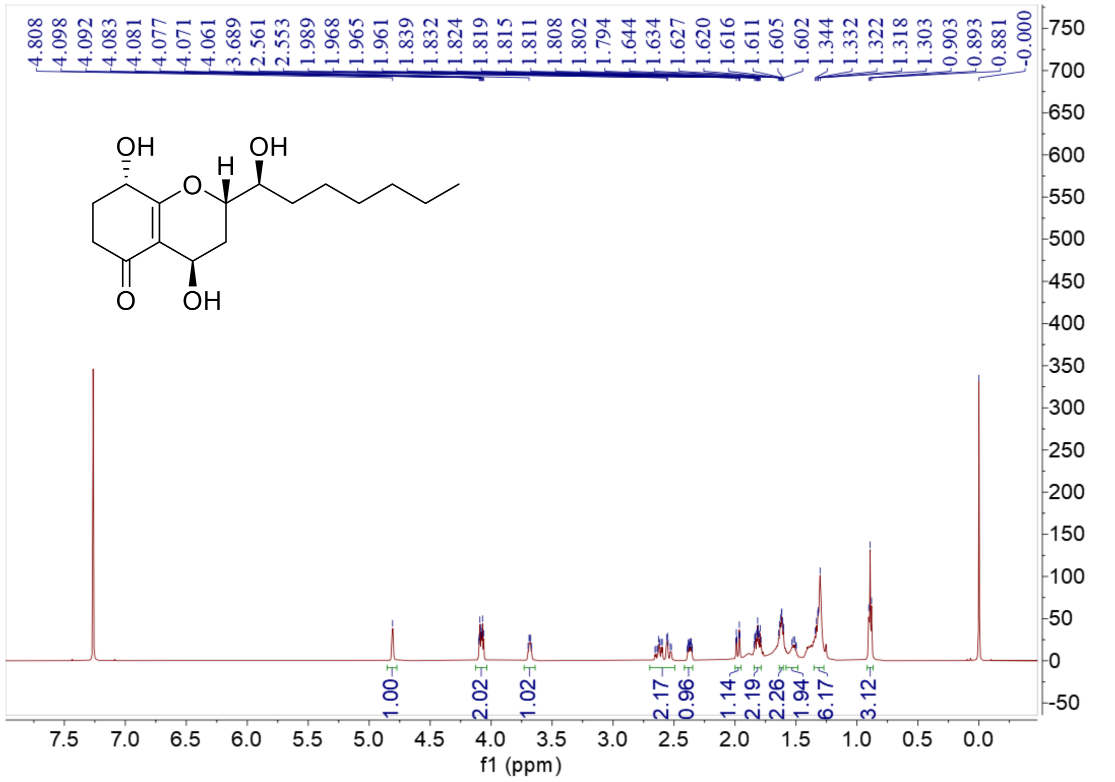

Figure S30. ^{1}H NMR spectrum (600 MHz, $CDCl_3$) of compound **5**

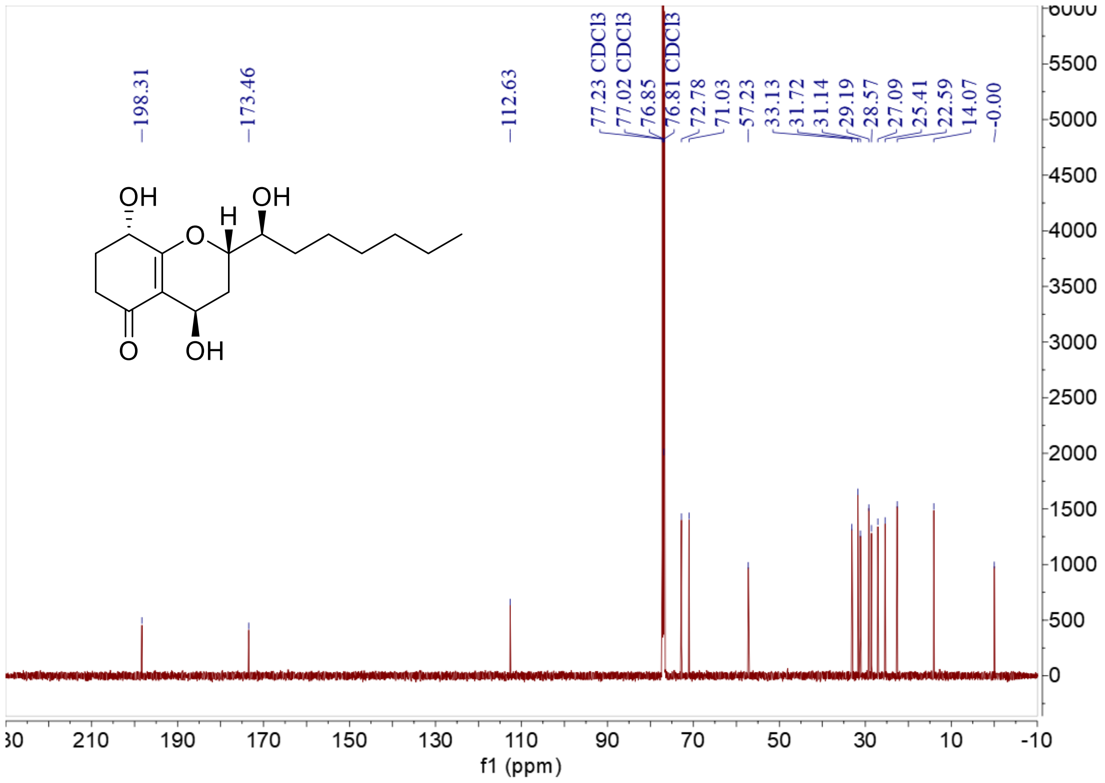

Figure S31. ^{13}C NMR spectrum (150 MHz, $CDCl_3$) of compound **5**

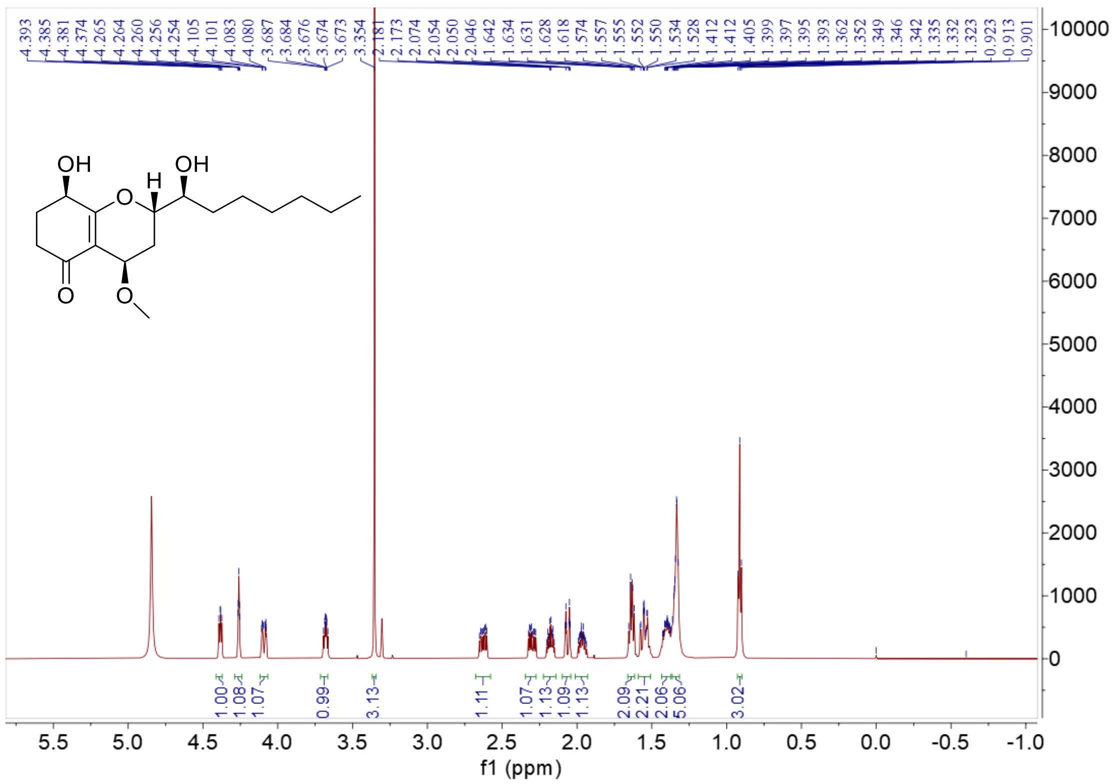

Figure S32. ^{1}H NMR spectrum (600 MHz, CD_3OD) of compound **6**

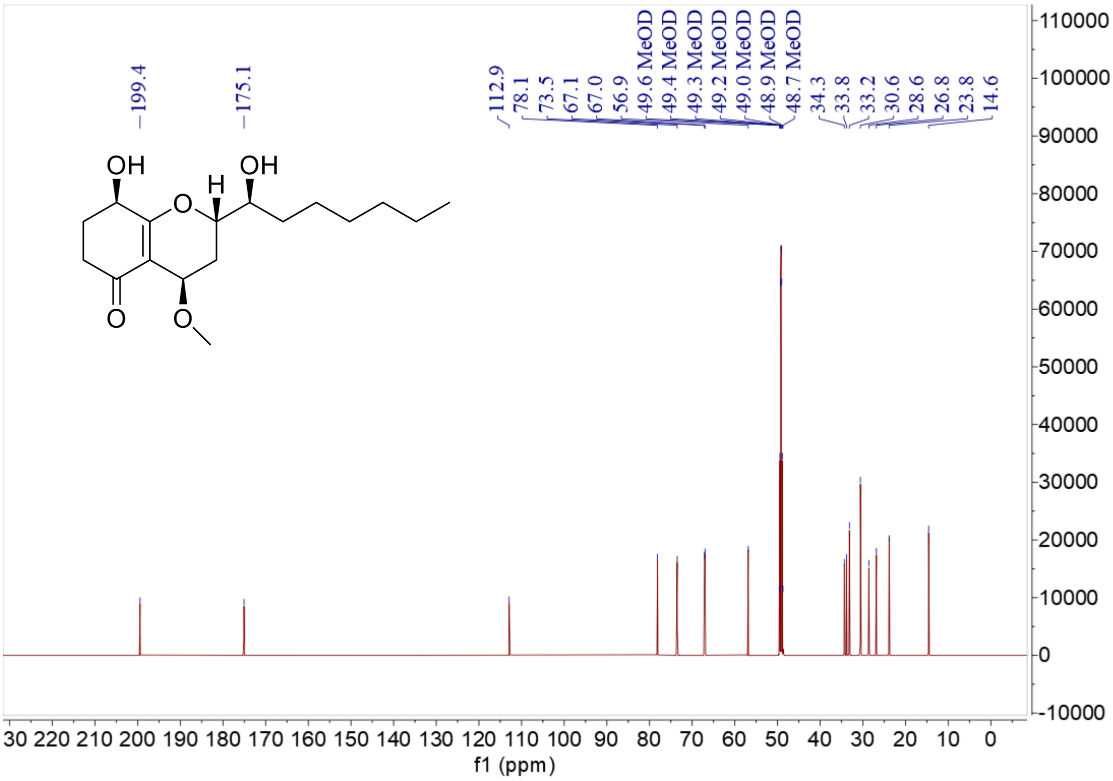

Figure S33. ^{13}C NMR spectrum (150 MHz, CD_3OD) of compound **6**

References

Bannwarth, C., Ehlert, S., Grimme, S. (2019). GFN2-xTB an accurate and broadly parametrized self- consistent tight-binding quantum chemical method with multipole electrostatics and density- dependent dispersion contributions, *J. Chem. Theor. Comput.* 15, 1652-1671. doi: 10.1021/acs.jctc.8b01176

Bruhn, T., Schaumlöffel, A., Hemberger, Y., Bringmann, G. (2013). SpecDis: quantifying the comparison of calculated and experimental electronic circular dichroism spectra, *Chirality.* 25, 243-249. doi: 10.1002/chir.22138

Chai, J. D., Head-Gordon, M. (2008). Long-range corrected hybrid density functionals with damped atom-atom dispersion corrections, *Phys. Chem. Chem. Phys.* 10, 6615-6620. doi: 10.1039/b810189b

Frisch, M. J., Trucks, G. W., Schlegel, H. B. et al. (2016). Gaussian 16, Revision A.02, Wallingford (CT), Gaussian, Inc.

Grimme, S., Ehrlich, S., Goerigk, L. (2011). Effect of the damping function in dispersion corrected density functional theory, *J. Comput Chem.* 32, 1456-1465. doi: 10.1002/jcc.21759

Li, J., Liu, J. K., Wang, W. X. (2020). GIAO ^{13}C NMR calculation with sorted training sets improves accuracy and reliability for structural assignation, *J. Org. Chem.* 85, 11350-11358. doi: 10.1021/acs.joc.0c01451

Pracht, P., Bohle, F., Grimme, S. (2020). *Phys.* Automated exploration of the low-energy chemical space with fast quantum chemical methods, *Chem. Chem. Phys*. 22, 7169-7192. doi: 10.1039/c9cp06869d

Tsuzuki, S., Uchimaru, T. (2020). Accuracy of intermolecular interaction energies, particularly those of hetero-atom containing molecules obtained by DFT calculations with Grimme's D2, D3 and D3BJ dispersion corrections, *Phys. Chem. Chem. Phys.* 22, 22508-22519. doi: 10.1039/d0cp03679j
